# Heat-shock responsive genes identified and validated in Atlantic cod (*Gadus morhua*) liver, head kidney and skeletal muscle using genomic techniques

**DOI:** 10.1186/1471-2164-11-72

**Published:** 2010-01-28

**Authors:** Tiago S Hori, A Kurt Gamperl, Luis OB Afonso, Stewart C Johnson, Sophie Hubert, Jennifer Kimball, Sharen Bowman, Matthew L Rise

**Affiliations:** 1 Ocean Sciences Centre, Memorial University of Newfoundland, St. John's, NL, A1C 5S7, Canada; 2 British Columbia Centre for Aquatic Health Sciences, Campbell River, BC, V9W 2C2, Canada; 3 Pacific Biological Station, Department for Fisheries and Oceans, Nanaimo, BC, V9T 6N7, Canada; 4 The Atlantic Genome Centre, Halifax, NS, B3H 3Z1, Canada; 5 Institute for Marine Biosciences, National Research Council of Canada, Halifax, NS, B3H 3Z1, Canada

## Abstract

**Background:**

Daily and seasonal changes in temperature are challenges that fish within aquaculture settings cannot completely avoid, and are known to elicit complex organismal and cellular stress responses. We conducted a large-scale gene discovery and transcript expression study in order to better understand the genes that are potentially involved in the physiological and cellular aspects of stress caused by heat-shock. We used suppression subtractive hybridization (SSH) cDNA library construction and characterization to identify transcripts that were dysregulated by heat-shock in liver, skeletal muscle and head kidney of Atlantic cod. These tissues were selected due to their roles in metabolic regulation, locomotion and growth, and immune function, respectively. Fish were exposed for 3 hours to an 8°C elevation in temperature, and then allowed to recover for 24 hours at the original temperature (i.e. 10°C). Tissue samples obtained before heat-shock (BHS), at the cessation of heat-shock (CS), and 3, 12, and 24 hours after the cessation of heat-shock (ACS), were used for reciprocal SSH library construction and quantitative reverse transcription - polymerase chain reaction (QPCR) analysis of gene expression using samples from a group that was transferred but not heat-shocked (CT) as controls.

**Results:**

We sequenced and characterized 4394 ESTs (1524 from liver, 1451 from head kidney and 1419 from skeletal muscle) from three "forward subtracted" libraries (enriched for genes up-regulated by heat-shock) and 1586 from the liver "reverse subtracted" library (enriched for genes down-regulated by heat-shock), for a total of 5980 ESTs. Several cDNAs encoding putative chaperones belonging to the heat-shock protein (HSP) family were found in these libraries, and "protein folding" was among the gene ontology (GO) terms with the highest proportion in the libraries. QPCR analysis of HSP90α and HSP70-1 (synonym: *HSPA1A*) mRNA expression showed significant up-regulation in all three tissues studied. These transcripts were more than 100-fold up-regulated in liver following heat-shock. We also identified HSP47, GRP78 and GRP94-like transcripts, which were significantly up-regulated in all 3 tissues studied. Toll-like receptor 22 (TLR22) transcript, found in the liver reverse SSH library, was shown by QPCR to be significantly down-regulated in the head kidney after heat-shock.

**Conclusion:**

Chaperones are an important part of the cellular response to stress, and genes identified in this work may play important roles in resistance to thermal-stress. Moreover, the transcript for one key immune response gene (TLR22) was down-regulated by heat-shock, and this down-regulation may be a component of heat-induced immunosuppression.

## Background

Temperatures are known to vary considerably at aquaculture cage-sites [1] and can approach upper critical temperatures (i.e. temperatures that are lethal) for Atlantic cod (*Gadus morhua*). These changes can occur both rapidly [e.g. increase of ~8°C in less than 12 hours during thermocline inversions, especially at depths where Atlantic cod tend to congregate (≥ 5 m)] [1] and seasonally. Fish confined to cages cannot completely avoid these temperatures and therefore are likely to be exposed to stressful conditions [1]. The stress response consists of numerous modifications to an organism's physiology and behaviour that are necessary to regain and maintain homeostasis once it has been challenged by changes in the environment, e.g. changes in temperature [2]. The cellular response to stress is the coordinated reaction to a threat of macromolecular damage and protects the cell against the potentially hazardous consequences of such events [3]. Cortisol is often regarded as a suitable indicator of stress, and one of the key hormones regulating the stress response [4-6]. Most actions of cortisol are thought to be mediated by the glucocorticoid receptor (GR), which upon binding to the hormone, moves into the nucleus and acts as a transcription factor that interacts with specific promoter regions known as glucocorticoid responsive elements (GREs) [4]. Stress can therefore have a significant impact on the transcription of specific genes. Thermal stress is also known to alter the transcription of a variety of genes including those encoding proteins that are involved in the response to oxidative stress, apoptosis, protein folding, energy metabolism, protein synthesis, membrane fluidity and immune function [7-12]. The proteins encoded by these transcripts include some of the elements that comprise and/or regulate both the organismal and cellular stress responses, and may help to protect the animal against the deleterious effects of stress. Among these are chaperones (e.g. members of the heat-shock protein gene family), anti-oxidative enzymes [e.g. catalase, superoxide dismutases (SODs), glutathione-S-transferases (GSTs)] and enzymes of carbohydrate metabolism (e.g. glycogen phosphorylase and phosphofructokinase).

Transcriptomic studies have been used to investigate the impacts of environmental stress on several organisms including fish [13-19]. Specifically, suppression subtractive hybridization (SSH) libraries have been used to identify fish genes that are responsive to diverse stimuli such as polyriboinosinic polyribocytidylic acid (pIC, a viral mimic) injection [20], formalin-killed atypical *Aeromonas salmonicida *injection [21], osmotic stress [22], cadmium [23], and pesticide exposure [24]. In order to better characterize the genes and molecular pathways involved in the Atlantic cod response to heat-shock, we constructed, sequenced, and characterized reciprocal SSH libraries enriched for transcripts dysregulated by heat-shock. Candidate heat-shock responsive cod cDNAs identified in the libraries were further investigated using real-time quantitative reverse transcription - polymerase chain reaction (QPCR). This report is the first to use high-throughput genomic techniques [SSH library construction; sequencing of expressed sequence tags (ESTs); cDNA sequence assembly, identification, and functional annotation of assembled sequences in an EST database; and QPCR for several SSH-identified genes in different tissues and at different time points post-stress] to characterize the transcriptomic response of Atlantic cod to thermal stress. This study is part of the Genome Canada funded Atlantic Cod Genomics and Broodstock Development Project (CGP, http://www.codgene.ca), which is developing many tools to study stress physiology in this species. A better understanding of changes in Atlantic cod gene transcription in response to heat-shock will potentially lead to new tools and techniques [e.g. molecular biomarkers, QPCR assays, and single nucleotide polymorphism (SNP) genotyping assays] for studying the impacts of environmental stress on cod, and for selecting individuals (broodstock) that can tolerate higher water temperatures.

## Results

### Plasma cortisol levels

**Figure 1:**
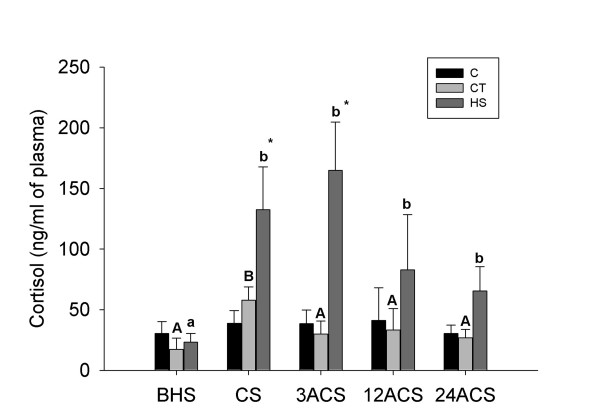
**Average (± SEM) plasma cortisol (ng/ml) levels in cod before and following a 3 hour heat-shock (transfer from 10°C to 18°C)**. Plasma cortisol levels in undisturbed control (C), control transferred (CT) and heat-shocked (HS) groups are shown before heat-shock (BHS), at the cessation of heat-shock (CS) and 3 h after the cessation of heat-shock (3ACS), 12 h after the cessation of heat-shock (12ACS) and 24 h after the cessation of heat-shock (24ACS). Different letters indicate significant differences between sampling points within the same group (p < 0.05). * indicates a significant difference between groups within a sampling point (p < 0.05). Cortisol levels in the C group did not change significantly during the experiment.

Plasma cortisol did not change significantly in the undisturbed control (C) group over the course of the experiment, averaging 30.4 - 41.2 ng ml^-1 ^(Fig. 1). In contrast, plasma cortisol levels were significantly (p < 0.05) increased in the "transferred but not heat-shocked control" (CT) and heat-shocked (HS) groups at the cessation of the 3 hour heat-shock (CS) when compared to their levels at the before heat-shock (BHS) time point (Fig. 1). At this time point (CS), average plasma cortisol levels in the heat-shocked group (HS: 132.4 ng ml^-1^) were 2.3-fold higher than for the CT group (57.8 ng ml^-1^) and 5.7-fold higher than for the HS BHS values (23.4 ng ml^-1^) (Fig. 1). Plasma cortisol in fish that were only transferred to a new tank at 10°C (CT) returned to basal levels (29.9 ng ml^-1^) at 3 hours after the cessation of heat-shock (3ACS). In contrast, cortisol in the heat-shocked fish reached its highest level at this time-point (164.8 ng ml^-1^), and remained significantly elevated at 12 (12ACS) and 24 (24ACS) hours after the cessation of heat-shock; values at these time-points were 3.5-fold and 2.8-fold higher than those measured in BHS fish, respectively.

### Characterization of SSH libraries and identification of candidate heat-shock responsive transcripts

We constructed and characterized 3 forward SSH libraries (liver, head kidney and skeletal muscle) and 1 reverse SSH library (liver). The SSH library method requires 2 μg of poly (A)^+ ^RNA (mRNA) [25], and mRNA usually only represents 1-5% of total RNA. In order to obtain adequate quantities of mRNA for SSH library construction, we isolated mRNA from pooled total RNA samples (see Methods). For SSH library construction, mRNA from heat-shocked (HS) (i.e. netted and transferred to a new tank at 18°C) fish were subtracted against mRNA from tissues of control transferred (CT) fish that were subjected to a handling stress (i.e. netted and transferred to a new tank at the same temperature) but without heat-shock. The mRNA used for SSH library construction consisted of samples taken at the cessation of the stressor and throughout the recovery period (up to 24 hours after the cessation of heat-shock). The resulting SSH libraries were enriched for transcripts that were dysregulated by the combined handling and heat-shock, but not those transcripts that were dysregulated by handling stress alone. We used three tissues to facilitate the identification of cod transcripts that were stress responsive across tissues, and therefore likely to be linked to the organism's overall sensitivity or resistance to thermal stress. We built reciprocal SSH libraries to identify transcripts that were up-regulated by heat-shock (i.e. "forward subtracted" libraries) as well as transcripts that were down-regulated by heat-shock (i.e. "reverse subtracted" libraries). We sequenced a total of 5980 expressed sequence tags (ESTs): 1524 from the liver forward library, 1451 from the head kidney forward library, 1419 from the skeletal muscle forward library, and 1586 from the liver reverse library. Although reverse SSH libraries were also made for the head kidney and skeletal muscle, initial complexity evaluation and/or sequencing showed that they were of low complexity (i.e. dominated by a few highly abundant transcripts), and they were therefore not subjected to deeper sequencing (Table 1). The ESTs were assembled into contiguous sequences (contigs) using Paracel Transcript Assembler (PTA) and annotated using AutoFACT [26]. Selected defense (i.e. stress and immune) relevant sequences are presented in Tables 2 and 3. In these tables, we do not include contigs and singletons with BLASTx hit E-values above 10^-5^, as well as many sequences that are unclassified (i.e. no BLAST hit), redundant, or with BLAST hits that have non-stress related functional annotations. A complete list of assembled sequences (i.e. contigs and singletons), ESTs contributing to contigs, EST accession numbers, associated AutoFACT hit descriptions (including BLASTx statistics) and functional annotations, can be found in Additional file 1 (Table S1) and Additional file 2 (Table S2).

**Table 1:** Statistics for ESTs generated for all SSH libraries^1^

Library Name	HK_F	**HK_R**^6^	SM_F	L_F	L_R
Tissue	Head Kidney	Head Kidney	Skeletal Muscle	Liver	Liver
Direction^2^	forward	reverse	forward	forward	reverse
CGP ID^3^	sb_gmnlkfta	sb_gmnlkrta	sb_gmnlmfta	sb_gmnllfta	sb_gmnllrta
# of ESTs	1451	93	1419	1524	1586
Average EST length^4^	297 bp	400 bp	340 bp	241 bp	227 bp
# of contigs^5^	212	8	159	200	178
# of singletons	746	35	483	612	668
# of non- redundant ESTs^7^	958	43	642	812	846
% redundancy^8^	33.9%	53.7%	54.7%	46.7%	46.6%

^1^Agarose gel electrophoresis of the muscle reverse library revealed the presence of 5 clear bands, which indicated a low-complexity library. Therefore, sequencing of this library was not attempted.

^2^The forward SSH libraries were constructed to be enriched for genes that were up-regulated by heat-shock, and the reverse SSH libraries were constructed to be enriched for genes that were down-regulated by heat-shock.

^3^The identifiers (ID) or names of the SSH libraries in the CGP EST database: http://www.codgene.ca.

^4^The ESTs were trimmed with Phred [62,63] with the trim_alt and trim_cutoff fixed at 0.06, followed by the removal of known contaminant sequences and short sequences (< 75 bp), and the average EST length was calculated based on edited sequences.

^5^Sequences generated were then clustered using Paracel Transcript Assembler (PTA), with the cluster threshold set at 100 for relatively stringent clustering.

^6^The 4 largest contigs in this library (Additional file 2, Table S2A) were annotated as haemoglobins and contained 45 sequences, which represented almost 50% of the good sequences obtained for it. Thus, this library was deemed low-complexity and not sequenced further.

^7^The number of non-redundant ESTs is the sum of the number of contigs plus the number of singletons.

^8^Percent redundancy is the proportion of redundant ESTs in each library, calculated as [1 - (Number of non-redundant ESTs/total number of ESTs)] multiplied by 100.

**Table 2:** Selected cDNAs^1 ^from all 3 forward (enriched for genes up-regulated by heat-shock) SSH libraries representing stress response related genes

				BLASTx identification^4 ^of selected contigs			
Contig ID (Accession Number)	Tissue (Library)	QPCR	# ESTs	Gene Name [Species of best BLASTx Hit]	%ID (aa length of align.)	E-value	Gene Ontology or function of putative orthologue^7^
16.C1 (ES784003)	Muscle (gmnlmfta)	Fig.5D^2^	13	Enolase 3 (beta muscle) [*Danio rerio*]	91% (159/174)	2e-81	Glycolysis (BP), Cytoplasm (CC)
76.C1 (ES784315)	Muscle (gmnlmfta)	Fig.5C^2^	8	Translationally-controlled tumor protein^5 ^[*Cyprinus carpio*]	68% (116/170)	9e-62	Calcium binding and apoptosis regulation [58]
45.C1 (ES783752)	Muscle (gmnlmfta)	Fig.4E^2^	7	Heat Shock Protein 47 [*Onchorhynchus mykiss*]	75% (132/174)	2e-76	Serine-type endopeptidase inhibitor activity (MF)
31.C1 (ES783410)	Muscle (gmnlmfta)	Fig.5B^2^	7	Chaperonin containing TCP1, subunit 5 (epsilon) (synonym: CCT 5) [*Danio rerio*]	93% (149/159)	2e-98	Protein folding (BP)*
27.C1 (ES783916)	Muscle (gmnlmfta)	Not sig.^3^	5	Phosphofructokinase, muscle a [*Danio rerio*]	88% (173/195)	4e-98	Glycolysis (BP)*
79.C1 (ES781823)	Liver (gmnllfta)	Not done	5	Cyclophilin A^6 ^[*Argopecten irradians*]	80% (132/164)	2e-75	Protein folding (BP)*
37.C1 (ES780395)	H. Kidney (gmnlkfta)	Not Done	5	Taldo1 protein^5,6 ^(synonym: transaldolase) [*Danio rerio*]	82% (120/145)	6e-63	Pentose-phosphate shunt (BP)*
65.C1 (EY973473)	H. Kidney (gmnlkfta)	Fig.4A^2^	4	Heat shock protein 90 alpha [*Paralichthys olivaceus*]	93% (93/99)	5e-47	Protein folding (BP)*, Response to stress (BP)*
62.C1 (ES781078)	Liver (gmnllfta)	Not done	4	Glutathione S-transferase pi^5 ^[*Carassius auratus*]	69% (46/66)	9e-14	Metabolic process (BP)*
24.C1 (ES781741)	Liver (gmnllfta)	Not done	4	T-complex protein 1 subunit beta^6 ^[*Salmo salar*]	88% (174/196)	2e-77	Protein folding (BP)*
41.C1 (EY973602)	H. Kidney (gmnlkfta)	Not done	3	Copper/zinc superoxide dismutase [Epinephelus coioides]	82% (125/152)	2e-71	Superoxide metabolic process (BP), Superoxide dismutase activity (MF)^8^
113.C1 (ES781350)	Liver (gmnllfta)	Fig.4B^2^	3	Heat shock protein 90 kDa beta, member 1^5,6 ^(synonym: GRP94) [*Danio rerio*]	91% (122/134)	4e-54	Protein folding (BP), Response to stress (BP)*
146.C1 (ES781990)	Liver (gmnllfta)	Fig.5A^2^	2	T-complex 1^6 ^(synonym: CCT 1) [*Pan troglodytes*]	98% (59/60)	3e-28	Assists folding of tubulin and other cytoskeleton proteins [56]
128.C1 (ES783784)	Muscle (gmnlmfta)	Fig.4C^2^	2	HSP70-1 protein^6 ^(synonym: *HSPA1A*) [*Oryzias latipes*]	92% (170/183)	7e-76	Response to stress (BP)*, ATP binding (MF)
5.C1 (EX190083)	H. Kidney (gmnlkfta)	Not done	2	TCP1-theta^6 ^[*Notothenia coriiceps*]	90% (56/62)	2e-25	Protein folding (BP), Protein binding (MF)^9^
10.C1 (ES781650)	Liver (gmnllfta)	Fig.4D^2^	2	78 kDa glucose-regulated protein^6 ^(synonym: GRP78) [*Salmo salar*]	98% (60/61)	1e-28	ATP binding (MF), Ig chain folding [39]^10^
27.C1 (ES780987)	Liver (gmnllfta)	Not sig.^3^	2	Aldolase B^5 ^[*Poecilia reticulata*]	91% (45/49)	8e-20	Glycolysis (BP)^11^
84.C1 (ES781112)	Liver (gmnllfta)	Not done	2	Pdia4 protein^6 ^[*Danio rerio*]	77% (71/92)	5e-36	Cell redox homeostasis (BP)*
172.C1 (ES781772)	Liver (gmnllfta)	Not done	2	Phosphoglucomutase 1 [*Danio rerio*]	96% (38/41)	1e-14	Carbohydrate metabolic process (BP)*
174.C1 (EX190172)	H. Kidney (gmnlkfta)	Not done	2	Glycogen phosphorylase [*Oreochromis mossambicus*]	84% (69/82)	4e-34	Carbohydrate metabolic process (BP)*

^1^ESTs from each individual forward library (e.g. liver) were assembled separately. Contigs identified in the forward library that were also found in the liver reverse library were included in this table if present as one contig of 2 or more ESTs in at least 2 forward libraries or if validated by QPCR. For genes represented by multiple contigs in a given library or across tissues (i.e. in different forward libraries), the contig with the highest number of contributing sequences is shown. Possible reasons for the presence of multiple contigs with the same gene name within a given library are provided in the discussion. All SSH contigs and singletons for the 3 libraries with all BLASTx statistics and GenBank accession numbers are presented in Additional file 1, Table S1. The GenBank accession number of one representative EST from each contig is given in this table. The names of these SSH libraries in the CGP EST database are gmnllfta (liver), gmnlkfta (head kidney), gmnlmfta (muscle).

^2^These genes showed at least one statistically significant difference between 2 groups and/or time points (e.g. CT at BHS versus CT at CS, or CT at CS versus HS at CS) in at least 1 tissue.

^3^These 2 genes were analyzed with QPCR at the individual level, but the data is not presented because there were no statistically significant differences between groups and/or time points.

^4^The BLASTx hit with the lowest E-value and a gene name (e.g. not predicted or hypothetical) is shown. BLAST statistics were collected on the 4^th ^of December, 2008 and reflect the entries in the nr protein database up to that date. aa = amino acids.

^5^These cDNAs were represented by a contig or a singleton in the liver reverse library.

^6^Synonyms for protein names were retrieved from the Swiss-Prot Protein Knowledgebase Database http://ca.expasy.org/sprot/: Cyclophilin A: Peptidyl-prolyl-isomerase A, PPIase.

Taldo1 protein: Transaldolase 1.

T-complex protein 1 subunit beta: CCT2.

Heat shock protein 90 kDa beta, member 1: HSPB1, Chaperone protein GP96, Tumor rejection antigen (gp96) 1.

T-complex 1: Chaperonin-containing T-complex polypeptide alpha subunit.

HSP70-1: HSP70, Heat Shock Protein 70 kDa 1, HSP72.

TCP1-theta: T-complex protein 1 subunit theta, CCT8.

Glucose-regulated protein 78 kDa: Heat Shock Protein 70 kDa protein 5.

Pdia4 protein: Protein disulfide isomerase-associated 4.

^7^Functional annotation associated with the cod cDNA's best BLAST hit or an annotated putative orthologue from *Homo sapiens *or *Mus musculus *(* or reference in []). Gene ontology (GO) categories: Biological Process (BP), Molecular Function (MF) and Cellular Component (CC).

^8^Other functional annotations associated with this cod EST are: Endoplasmic reticulum (CC) and Nucleotide binding (MF).

^9^Other functional annotations associated with this cod EST are: Oxireductase activity (MF), Zinc ion binding (MF) and Copper ion binding (MF).

^10^Other functional annotations associated with this cod EST are: Cellular protein (CC), Cytoplasm (CC), Metabolism (BP), ATP binding (MF), Nucleotide binding (MF).

^11^Other functional annotations associated with this cod EST are: Catalytic activity (MF), Fructose biphosphate aldolase activity (MF) and Metabolic process (BP).

**Table 3:** Selected cDNAs^1 ^from the liver reverse (enriched for genes down-regulated by heat-shock) SSH library representing immune/stress related genes

			BLASTx identification^4 ^of selected transcripts			
				
Contig or Sequence ID (Accession Number)	QPCR	# ESTs	Gene Name [Species of best BLASTx Hit]	%ID (aa length of align.)	E-value	**Gene Ontology or function of putative orthologue**^7^
154.C1 (ES783183)	Not done	3	Map4k4^5,6 ^[*Mus musculus*]	92% (24/26)	4e-05	Protein amino acid phosphorylation (BP)*
111.C1 (FL634330)	Not done	2	Glutathione peroxidase [*Xenopus tropicalis*]	55% (30/54)	1e-11	Response to oxidative stress (BP)*
121.C1 (ES782223)	Not done	2	Tetraspanin-6 [*Salmo salar*]	96% (28/29)	9e-09	G-protein coupled receptor protein signaling pathway (BP)*
129.C1 (ES782343)	Not done	2	Nuclear factor interleukin-3 regulated [*Danio rerio*]	73% (31/42)	6e-08	Immune response (BP)*
143.C1 (ES782977)	Not done	2	Chaperonin containing TCP1- subunit 3^4,6 ^[*Salmo salar*]	94% (127/134)	7e-67	Protein folding (BP)*
128.C1 (ES782638)	Fig.6B^2^	2	Nuclear protein 1^5,6 ^[*Salmo salar*]	50% (35/70)	4e-11	Cell growth (BP)*, Acute inflammatory response (BP)*
168.C1 (ES782218)	Fig.6C^2^	2	Alpha-1-microglobulin/bikunin precursor [*Oncorhynchus mykiss*]	55% (21/38)	1e-04	Serine-type endopeptidase inhibitor activity (MF), Endopepitidase (MF), Transporter activity (MF)
69.C1 (ES783083)	Fig.6D^2^	2	Immunoglobulin heavy chain, secretory form^5 ^[*Gadus morhua*]	97% (137/140)	1e-70	Immune response (BP)*
91.C1 (ES782456)	Not done	2	Lectin [*Oncorhynchus mykiss*]	48% (25/52)	2e-13	Sugar binding (MF)*
1e08 (ES782565)	Not done	1	Hepcidin precursor^5 ^[*Gadus morhua*]	98% (78/79)	1e-26	Innate immune response (BP)*
2i08 (ES782607)	Fig.6A^2^	1	TLR22^4,6 ^[*Takifugu rubripes*]	88% (39/44)	3e-29	Immune response (BP), Inflammatory response (BP)^8^
7m20 (FL634530)	Not sig.^3^	1	Interleukin-8 [*Melanogrammus aeglefinus*]	82% (63/76)	4e-36	Immune response (BP), Cytokine activity (MF)^9^
7p13 (FL634573)	Not done	1	TNFAIP3 interacting protein 1 [*Danio rerio*]	86% (74/86)	1e-34	Negative regulation of viral genome duplication (BP)

^1^ESTs from the liver reverse library were assembled separately. These cDNAs were not represented by any contig of 2 or more ESTs in any of the 3 forward libraries. For genes represented by multiple contigs in this library, the contig with the highest number of contributing sequences is shown. Possible reasons for the presence of multiple contigs with the same gene name within a given library are provided in the discussion. All SSH contigs and singletons for the liver reverse library, with all BLASTx statistics and GenBank accession numbers, are presented in the Additional file 2, Table S2. The GenBank accession number of one representative EST from each contig is given in this table. The name of this SSH library in the CGP EST database is gmnllrta. Sequence names reflect the plate number and the well number (e.g. 7p13 - plate 7, well p13).

^2^These genes showed at least one statistically significant difference between 2 groups and/or time points (e.g. CT at BHS versus CT at CS, or CT at CS versus HS at CS) in at least 1 tissue.

^3^This gene was analyzed with QPCR, but the data is not presented because there were no statistically significant differences between groups and/or time-points.

^4^Refer to footnote ^4 ^in Table 2.

^5^These cDNAs were each represented by one singleton in the liver forward library.

^6^Synonyms for protein names were retrieved from the Swiss-Prot Protein Knowledgebase Database http://ca.expasy.org/sprot/: Map4k4: Mitogen-activated protein kinase kinase kinase kinase 4. Chaperonin containing TCP1- subunit 3: CCT3. Nuclear protein 1: Protein p8, Candidate of metastasis 1. TLR22: Toll-like receptor 22.

^7^Refer to footnote ^7 ^in Table 2.

^8^Other functional annotations associated with this cod EST are: Innate immune response (BP), Receptor activity (MF), Integral part to membrane (CC).

^9^Other functional annotations associated with this cod EST are: Extracellular region (CC), Extracellular space (CC).

Haemoglobin subunit alpha-1 was the largest contig (17 contributing sequences) in the head kidney forward library (Additional file 1, Table S1A), and haemoglobin subunit beta-1 was the largest contig (25 contributing sequences) in the liver forward library (Additional file 1, Table S1B). In the skeletal muscle forward library the largest contig was parvalbumin (66 contributing sequences) (Additional file 1, Table S1C). In order to identify and validate transcripts that were up-regulated in response to heat-shock, we looked in the forward libraries for cod cDNAs that: a) had significant BLASTx hits (i.e. E-values lower than 10^-5^) against proteins with stress-relevant functional annotations; b) were in contigs in more than one forward library; and/or c) had a relatively large number of contributing sequences (more than 5). A list of selected contigs from the forward libraries, with detailed BLASTx statistics and functional annotations, is presented in Table 2. These include several cod cDNAs with BLASTx hits against putative chaperone or chaperone-related proteins, including the following: the translationally-controlled tumor protein (TCTP) (found in skeletal muscle, head kidney and liver); heat shock protein 47 (HSP47) (skeletal muscle); several putative members of the T-complex containing chaperonin system (CCT) including chaperonin containing TCP1, subunit 5 (synonym: CCT 5) (skeletal muscle and liver), T-complex protein 1 subunit beta (synonym: CCT 2) (liver), T complex 1 (synonym: CCT 1) (liver, skeletal muscle and head kidney) and TCP1-theta (synonym: CCT 8) (head kidney); heat shock protein 90 alpha (HSP90α) (head kidney and skeletal muscle); heat shock protein 90 kDa beta, member 1 (synonym: glucose regulated protein 94, GRP94) (liver and head kidney); HSP70-1 (synonym: *HSPA1A*) (skeletal muscle); and the 78 kDa glucose-regulated protein (synonym: GRP78) (liver).

We also identified several cod transcripts with significant BLASTx hits against genes and proteins involved in carbohydrate metabolism (Table 2 and Additional file 1, Table S1). These include enolase 3 (skeletal muscle), phosphofructokinase 1 (PFK) (skeletal muscle), transaldolase 1 (Taldo1) (head kidney and skeletal muscle), aldolase B (liver), phosphoglucomutase 1 (PGM1) (liver and skeletal muscle), glycogen phosphorylase (head kidney) and phosphoenolpyruvate carboxykinase (PEPCK) (liver).

In the liver reverse SSH library (i.e. enriched for transcripts down-regulated by heat-shock) the 3 largest contigs were also haemoglobins (subunits alpha-1, beta-1 and beta-2) (Additional file 2, Table S2B). Some of the defence-relevant transcripts identified in this library were: tetraspanin-6, nuclear factor interleukin-3 regulated protein, alpha 1-microglobulin/bikunin (bikunin), lectin, Immunoglobulin (Ig) heavy chain (synonym: IgM), hepcidin, interleukin-8 (IL-8), Toll-like receptor 22 (TLR22) and TNFAIP3 interacting protein 1. We also identified the cell cycle regulator nuclear protein 1 (NUPR1, synonym: p8). Stress has been shown to influence fish immune function [27], and therefore for QPCR studies we chose primarily cod cDNAs representing acute phase and immune-relevant genes as heat-shock responsive candidate transcripts from the liver reverse SSH library. Detailed information on these selected cod transcripts is presented in Table 3. For a more comprehensive list (i.e. containing all contigs and singletons identified in the reverse libraries along with AutoFACT hit descriptions, BLASTx statistics and functional annotations), refer to Additional file 2, Table S2.

### Functional (Gene Ontology) annotation of assembled ESTs from SSH libraries

**Figure 2:**
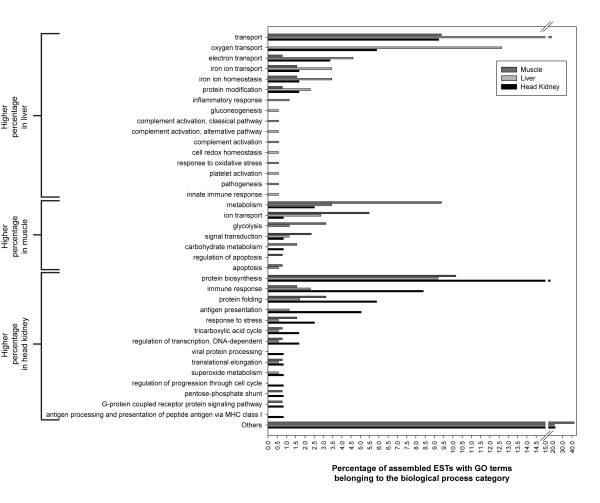
**Summary of Gene Ontology (GO) functional annotation of assembled ESTs identified in the forward SSH libraries**. GO annotations were obtained using AutoFACT [26] and GOblet [28] analysis of clusters. Numbers represent the percentage of ESTs with a particular GO annotation relative to the total number of sequences with GO annotation (all belonging to the Biological Process GO category). Light grey: liver. Dark grey: skeletal muscle. Black: head kidney.

**Figure 3:**
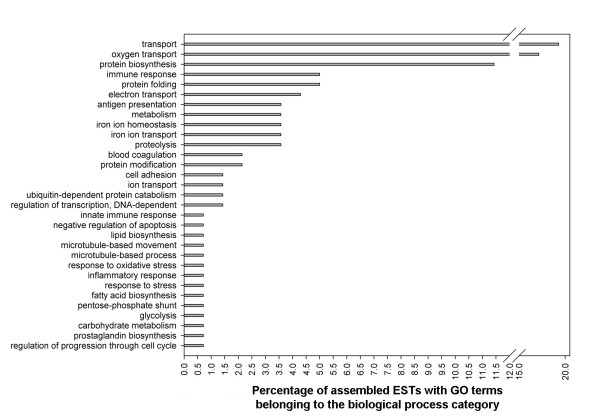
**Summary of Gene Ontology (GO) functional annotation of assembled ESTs identified in the liver reverse SSH library**. GO annotations were obtained using AutoFACT [26] and GOblet [28] analysis of clusters. Numbers represent the percentage of ESTs with a particular GO annotation relative to the total number of sequences with GO annotation (all belonging to the Biological Process GO category).

The ESTs from the 3 forward SSH libraries represented 958 (head kidney: 212 contigs and 746 singletons), 812 (liver: 200 contigs and 612 singletons) and 642 (skeletal muscle: 159 contigs and 483 singletons) putative transcripts, and ESTs from the liver reverse library represented 846 putative transcripts (178 contigs and 668 singletons) (Table 1). Using AutoFACT [26] and GOblet [28], these assembled sequences were assigned to 143, 157, 165 and 125 gene ontology (GO) terms (belonging to all 3 GO categories), respectively. A comprehensive list of assembled ESTs, and detailed BLAST statistics and functional annotations (e.g. associated GO terms) can be found at the Cod Genomics Project website http://www.codgene.ca and in Additional files 1, 2 and 3 (Tables S1-S3). Protein biosynthesis and transport were among the GO terms with the highest proportion of associated assembled ESTs in all 4 libraries (Figs. 2 and 3). We identified many sequences that were assigned to GO terms relevant to stress or immune responses (e.g. metabolism, protein folding, immune response, proteolysis, glycolysis, response to stress and signal transduction). The proportions of assembled ESTs associated with GO annotations belonging to specific biological process categories relative to the total number of assembled ESTs with biological process GO annotations within each SSH library are shown in Figs. 2 and 3.

### Expression of candidate heat-shock responsive transcripts

We used QPCR to validate and further study the effects of heat-shock on 16 SSH-identified Atlantic cod transcripts. While SSH libraries were constructed using pooled mRNA samples, QPCR was conducted using individual RNA templates to assess biological variability. Eight cod cDNA sequences selected for QPCR had significant BLASTx hits against proteins with chaperone functions (TCTP, HSP47, CCT 5, HSP90α, GRP94, CCT 1, HSP70-1 and GRP78), 3 had significant BLASTx hits against proteins involved in carbohydrate metabolism (enolase, PFK and aldolase), 3 were identified as immune-relevant transcripts (IgM, TLR22 and IL-8), 1 was most similar at the predicted amino acid level to an acute phase protein (bikunin) and 1 cDNA was identified as the transcript for a gene involved in regulation of the cell cycle (NUPR1). The numbers of contributing sequences and libraries where these transcripts were found are detailed in Tables 2 and 3.

**Figure 4:**
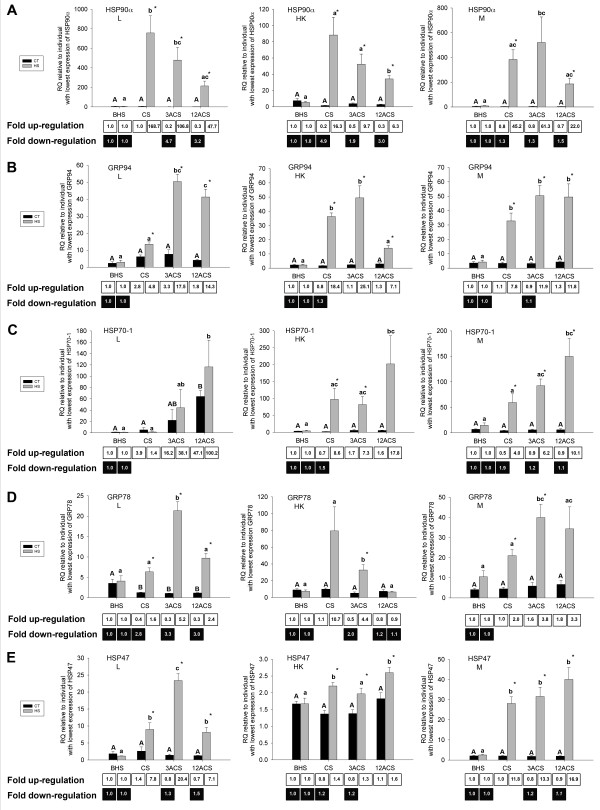
**QPCR of selected genes with stress relevant functional annotations identified in the forward SSH libraries (designed to be enriched for transcripts up-regulated by heat-shock): Part I.** The RQs (relative quantities), normalized to 18S ribosomal RNA expression and calibrated to the individual with the lowest expression of each gene of interest (see methods section), are presented as averages ± SEM. The levels of gene expression of heat-shock protein 90 alpha (HSP90α:  **A**), heat shock protein 90kDa beta, member 1 (GRP94: **B**), HSP70-1 protein (HSP70-1: **C**), 78 kDa glucose regulated protein (GRP78: **D**), heat-shock protein 47 (HSP47: **E**) are shown for the control transferred (CT) and heat-shocked (HS) groups before heat-shock  (BHS), at the cessation of heat-shock (CS), 3h after the cessation of heat-shock (3ACS), and 12h after the cessation of heat-shock (12ACS). Different letters indicate significant differences between sampling points within the same treatment (p<0.05). * indicates significant differences between CT and HS groups at a given sampling point (p<0.05).  Numbers in the boxes represent overall fold-changes. For each treatment, overall fold upregulation was calculated relative to the appropriate before heat-shock (BHS) value as (average RQ of time point)/(average RQ of appropriate group at the BHS time point), and overall fold down-regulation was calculated when necessary (i.e. if overall fold upregulation  was < 1) as the inverse of overall fold up-regulation. Each panel shows the expression for a given gene of interest in all 3 tissues studied. L: liver. HK: head kidney.  M: skeletal muscle.

**Figure 5:**
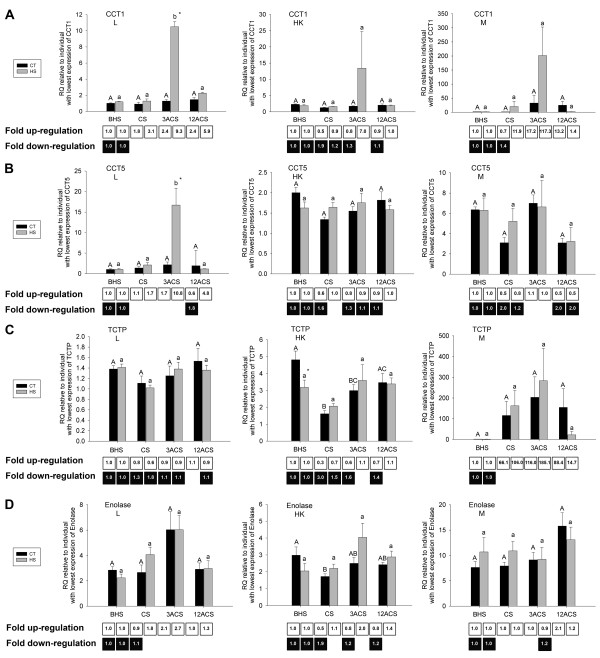
**QPCR of selected genes with stress relevant functional annotations identified in the forward SSH libraries (designed to be enriched for transcripts up-regulated by heat-shock): Part II.** The RQs (relative quantities), normalized to 18S ribosomal RNA expression and calibrated to the individual with the lowest expression of each gene of interest (see methods section), are presented as averages ± SEM. The levels of gene expression of T-complex 1 (CCT1: **A**), chaperonin containing TCP1, subunit 5 (CCT5 : **B**), translationally-controlled tumor protein (TCTP: **C**) and enolase 3 (Enolase: **D**) are shown for the control transferred (CT) and heat-shocked (HS) groups before heat-shock (BHS), at the cessation of heat-shock (CS), 3h after the cessation of heat-shock (3ACS), and 12h after the cessation of heat-shock (12ACS). Different letters indicate significant differences between sampling points within the same treatment (p<0.05). * indicates significant differences between CT and  HS groups at a given sampling point (p<0.05). Numbers in the boxes represent overall  fold-changes. For each treatment, overall fold up-regulation was calculated relative to the appropriate before heat-shock (BHS) value as (average RQ of time point)/(average RQ of appropriate group at the BHS time point), and overall fold down-regulation was calculated when necessary (i.e. if overall fold up-regulation was < 1) as the inverse of overall fold up-regulation. Each panel shows the expression for a given gene of interest in all 3 tissues studied. L: liver. HK: head kidney. M: skeletal muscle.

Of the 11 cDNAs arising from the forward libraries and analyzed with QPCR (Table 2), all of those encoding proteins with putative chaperone function with the exception of TCTP were shown to be significantly up-regulated at the mRNA level by heat-shock in at least one tissue and at least one time point post heat-shock (Fig. 4A-E and Fig. 5A-C). TCTP mRNA levels were responsive to handling alone (Fig. 5C) in the head kidney, with an average fold down-regulation of 3.0 and 1.6 at CS and at 3ACS, respectively. We also found that HSP70-1 (Fig. 4C) and GRP78 (Fig. 4D) transcripts responded significantly to handling alone in the liver. HSP70-1 mRNA was significantly up-regulated by an average of 47.1-fold at 12ACS in the CT group when compared to the values for BHS fish, while GRP78 mRNA was significantly down-regulated by an average of ~3.0-fold at all time points in the same group. These two transcripts showed no response to handling alone in the other tissues studied. Of the cDNAs with significant BLASTx hits against proteins involved in carbohydrate metabolism, enolase mRNA expression was significantly responsive to handling alone in only a single tissue (i.e. average down-regulation of 1.9-fold in the head kidney of the CT group at the CS time point, Fig. 5D), but was not significantly responsive to heat-shock. PFK and aldolase presented no significant differences between groups or time points (data not shown).

The genes with the highest significant mRNA up-regulation in response to heat-shock were HSP90α [Fig. 4A - average fold-changes of 168.7 (liver - CS) and 61.3 (skeletal muscle - 3ACS)]; HSP70-1 [Fig. 4C - average fold-changes of 100.2 (liver - 12ACS) and 17.8 (head kidney - 12ACS)]; GRP94 [Fig. 4B - average fold-changes of 25.1 (head kidney - 3ACS) and 17.5 (liver - 3ACS)]; and HSP47 [Fig. 4E - average fold-changes of 20.4 (liver - 3ACS) and 16.9 (skeletal muscle - 12ACS)]. Of the 7 transcripts significantly up-regulated by heat-shock that were identified in the forward libraries (i.e. HSP90α, GRP94, HSP70-1, GRP78, HSP47, CCT1 and CCT5 - Fig. 4A-E and Fig. 5A-B), only two transcripts (CCT-1 and CCT-5, Fig. 5A and 5B) were not significantly up-regulated (i.e. in at least one heat-shock time point relative to the BHS time point) in all 3 tissues tested. Within the tissues, HSP90α mRNA presented the greatest fold up-regulation in liver (average 168.7-fold up-regulation at CS) and skeletal muscle (average 61.3-fold up-regulation at 3ACS), while GRP94 mRNA presented the highest fold up-regulation in the head kidney (average 25.1-fold up-regulation at 3ACS). The transcripts with significant up-regulation in response to heat-shock also showed differences in timing of expression in the three tissues studied. As previously stated, HSP90α mRNA expression in the liver peaked relative to the BHS time point at CS (Fig. 4A). However, many stress-relevant transcripts had significant maximum fold up-regulation at 3ACS relative the BHS time point. These were: HSP90α in the skeletal muscle (Fig. 4A, 61.3 fold-change); GRP94 in all tissues [Fig. 4B - average fold-changes of 17.5 (liver), 25.1 (head kidney), and 11.9 (skeletal muscle)]; GRP78 in all tissues [Fig. 4D - average fold-changes of 5.2 (liver), 4.4 (head kidney), and 3.8 (skeletal muscle)]; HSP47 in the liver (Fig. 4E - average fold-change of 20.4); and CCT1 (Fig. 5A - average fold-change of 9.3) and CCT5 (Fig. 5B - average fold-change of 10.8) both in the liver. Transcripts for HSP90α in the head kidney (Fig. 4A - average fold-change of 6.3), HSP47 in the skeletal muscle and head kidney [Fig. 4E - average fold-changes of 16.9 (skeletal muscle) and 1.6 (head kidney)], and HSP70-1 in all tissues [Fig. 4C - average fold-change of 100.2 (liver), 17.8 (head kidney) and 10.1 (skeletal muscle)] had maximum significant mRNA fold up-regulation at 12ACS relative to the BHS time point. The timing and magnitude of the changes in expression of transcripts for HSP90α (Fig. 4A), HSP47 (Fig. 4E), and GRP78 (Fig. 4D) also varied between different tissues. For example, HSP90α transcript was maximally up-regulated at CS in the liver but at 3ACS in the skeletal muscle relative to the BHS time point.

**Figure 6:**
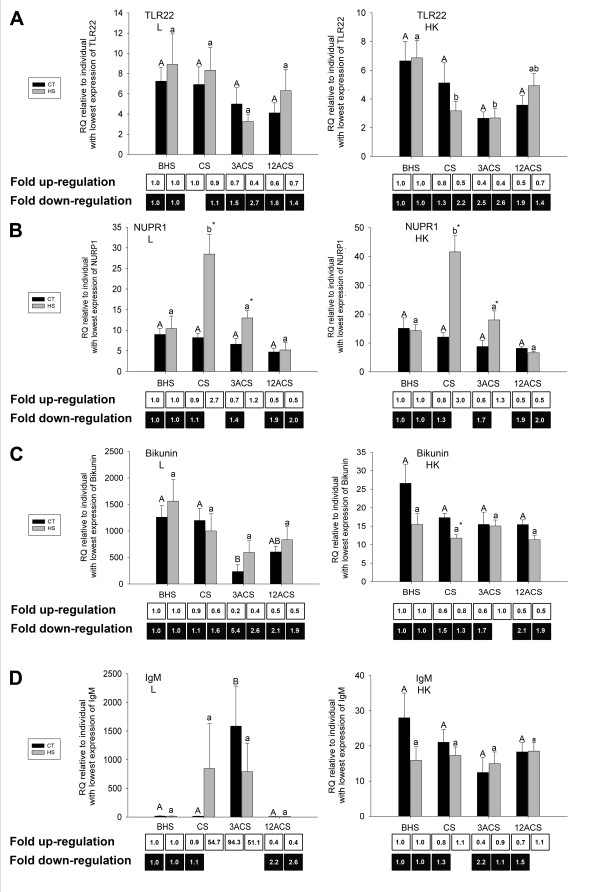
**QPCR of selected transcripts with immune relevant functional annotations identified in the reverse liver SSH library (designed to be enriched for transcipts down-regulated by heat-shock).** The RQs (relative quantities), normalized to 18S ribosomal RNA expression and calibrated to the individual with the lowest expression of each gene of interest (see methods section),  are presented as averages ± SEM.  The levels of gene expression of TLR22 (Toll-like receptor 22: **A**), nuclear protein 1 (NUPR1: **B**), alpha 1-microglobulin/bikunin (Bikunin: **C**) and immunoglobulin heavy chain, secretory form (IgM: **D**) are shown for the control transferred (CT) and heat-shocked (HS) groups before heat-shock (BHS), at the cessation of heat-shock (CS), 3h after the cessation of heat-shock (3ACS), and 12h after the cessation of heat-shock (12ACS).  Different letters indicate significant differences between sampling points within the same treatment (p< 0.05). * indicates significant differences between CT and HS groups within a given sampling point (p< 0.05).  Numbers in the boxes represent overall fold-changes.  For each treatment, overall fold up-regulation was calculated relative to the appropriate before heat-shock (BHS) values as (average RQ for a time point)/(average RQ of the appropriate group at the BHS time point), and overall fold down-regulation was calculated when necessary (i.e. if overall fold upregulation was < 1) as the inverse of overall fold up-regulation.  Each panel shows the expression for a given gene of interest in both tissues studied.  L: liver.  HK: head kidney.

From the 5 cDNAs selected for QPCR studies from the reverse liver library (Table 3), NUPR1 and TLR22 were the only cod transcripts that responded significantly to heat-shock. The transcript levels for TLR22 were down-regulated by an average of 2.2- and 2.6-fold in the HS group in the head kidney at CS and 3ACS, respectively, when compared to the levels at the BHS time point (Fig. 6A). Interestingly, NUPR1 transcripts were significantly up-regulated by heat-shock at CS in both liver (average 2.7-fold change) and head kidney (average 3.0-fold change), and at 3ACS the HS group still showed significantly higher levels of NUPR1 transcript than in the CT group in both tissues (Fig. 6B). Bikunin and IgM mRNAs displayed similar responses in CT and HS groups (Fig. 6C and 6D) and IL-8 mRNA showed no significant changes (data not shown). Bikunin transcripts in the liver and head kidney were reduced by both stressors. They were significantly lower in the CT group in the liver at 3ACS when compared to levels at the BHS time point, and in the head kidney from HS fish when compared to those from the CT group at the CS time-point (Fig. 6C).

## Discussion

Thermal stress can pose a significant challenge to cod at aquaculture cage sites as summer temperatures may approach the upper critical thermal limit for this species and/or change rapidly during the day (due to thermocline inversions, i.e. when bays "turn over")[1]. Heat-stress is known to cause many physiological changes in cod including the release of stress hormones (e.g. cortisol), changes in the expression of immune relevant transcripts, alterations in oxygen consumption and heart rate, and increased mortality [1,7,29]. Therefore, a better understanding of the mechanisms mediating the response to thermal stress should provide insights into how to mitigate and/or avoid the deleterious effect of such environmental challenges. We observed a significant elevation in average plasma cortisol in both control transferred (CT) and heat-shocked (HS) cod at CS; plasma cortisol in HS fish was 2.3-fold higher than in CT fish at the CS time point, and 5.5-fold higher in HS when compared to CT at 3ACS. Further, cortisol levels in HS fish remained elevated during the recovery period. In contrast, average plasma cortisol levels in the CT group had returned to basal levels (i.e. not significantly different from the CT group at BHS) by 3ACS. These results confirm that 3 hours of exposure to 18°C was a severe stressor for these juvenile cod.

Some of the terms more frequently represented amongst the GO annotated ESTs derived from the forward libraries were protein folding, signal transduction, immune response, and response to stress (Fig. 2). The genes associated with these terms may be important in the strategies involved with coping with stress, and variability in their sequences (e.g. exonic, intronic, or regulatory region SNPs) and/or timing and magnitude of mRNA expression (i.e. expression profiles) could reveal markers for increased resistance to thermal and other stressors (research ongoing). This is the first study to use high-throughput genomic techniques to investigate the response to heat-shock in cod, and to provide expression profiles of a wide range of transcripts encoding putative chaperone proteins in tissues of fish that have distinct physiological roles. Many of the transcripts validated at the individual level using QPCR in this study had BLASTx hits that were associated with the GO terms mentioned above and exhibited differences in expression profiles between tissues. These findings indicate that the cellular response to heat-shock in cod is complex, involves several genes, and may be controlled by different cues and/or transcription regulation mechanisms in different tissues, as has been observed in human cells [30]. In all three tissues studied we showed an increase in transcript levels of HSP70-1 (a putative orthologue of the human *HSPA1A *gene as per the nomenclature proposed by Kampinga et al. [31]). Previous reports on cod and haddock (*Melanogrammus aeglefinus*) [29,32,33] did not detect an increase in HSC71/HSP70-1 protein expression in the gills and liver of cod or haddock in response to thermal stress. It could be that an elevation in HSC71/HSP70-1 protein was not detected in cod or haddock in the aforementioned studies due to the fact that the antibodies [i.e. polyclonal anti-rainbow trout (*Onchorynchus mykiss*) HSP70 (Agrisera, Sweden) and monoclonal anti-mouse (*Mus musculus*) HSP70/HSC71 (Sigma Co., St. Louis)] used were not generated against cod or haddock HSP70-1 and may not have recognized the HSP70-1 protein in these species.

Several of the cDNAs identified in this study were represented by more than one contig in a single library. There are several possible reasons for the presence of more than one contig with the same annotation in a given library. Multiple, same-named contigs may represent: a) different paralogues; b) different alleles at a given locus; or c) non-contiguous segments of a given cDNA (the last-mentioned potential cause of multiple same-named contigs may be the most likely since SSH library construction includes a restriction digest with *Rsa*I, potentially resulting in more than one contig from a given full mRNA). For example, we report two contigs that were annotated as HSP90α in the head kidney forward library (Additional file 1, Table S1A). Further analysis of these contigs using nucleotide alignments against a full-length sequence obtained from Chinook salmon (*Onchorynchus tshawytscha*) [GenBank: U89945][34] suggests that these are likely to represent non-contiguous regions of the same cod cDNA (data not shown). However, the HSP family provides important examples of differential expression (i.e. constitutive and induced expression profiles) between distinct paralogues (e.g. HSC71/HSP70-1) [35], and further studies addressing this question will be needed to determine the roles of different Atlantic cod chaperone paralogues in thermal tolerance.

Molecular chaperones play important roles in cell physiology in both unstressed and stressed situations. These proteins assist with the folding of nascent peptides and the *de-novo *folding of denatured proteins, the transport of unfolded proteins across membranes, quality control and conformational changes that affect function [36]. Sudden or chronic increases in temperature are known to induce both mRNA and protein expression of several chaperones, such as those belonging to the HSP family [35].

Among the clients that these proteins bind to are physiologically relevant proteins such as the glucocorticoid [4] and aryl hydrocarbon [37] receptors (clients of HSP90), heat-shock factor 1 [38] (client of HSP70), Immunoglobulin (Ig) heavy chain [39] (client of GRP78) and the Toll-like receptors [40] (clients of GRP94). Transcripts encoding putative orthologues of all of these chaperones were identified in our libraries, and all of them were confirmed to be heat-shock responsive mRNAs. HSP90α mRNA expression was up-regulated in the liver more than 150-fold at CS relative to the before heat-shock (BHS) time point (Fig 4A). The up-regulation of HSP90s in response to heat-shock has been demonstrated at both mRNA and protein levels in different species of fish [41,42]. For example, Cara et al. [41] detected a ~6000% increase in HSP90 proteins and a ~600% increase in HSP70 protein in fasted +10°C heat-shocked rainbow trout larvae. In our study, there was a higher maximum fold-induction of HSP90α transcripts compared to HSP70-1 transcripts in both liver and muscle following heat-shock. We observed a significant increase in HSP70-1 mRNA expression in the liver of CT fish at 12ACS, which may have been a result of fasting. Cara et al. [41] observed increased HSP70 protein expression in fasted non heat-shocked rainbow trout larvae. Among the many clients of these chaperones are heat-shock factor 1 (client of HSP70) and the glucocorticoid receptor (client of HSP90). Therefore, the increase in the levels of mRNAs encoding these chaperones may indicate that their products are essential in maintaining signal transduction during stress and are likely to be proteins involved in heat-stress tolerance.

Stress also has an impact on the fish's immune system, and temperature stress has been shown to decrease serum IgM content and increase the susceptibility of sea bass (*Dicentrachus labrax*) to nodavirus [27]. Nodaviruses belong to the family *Nodaviridae*, and are the causative agents of viral nervous necrosis (VNN). These viral pathogens also infect cod, and can cause high levels of morbidity and mortality [43]. GRP78 is essential for the appropriate folding and secretion of immunoglobulin light and heavy chains from the endoplasmic reticulum (ER) [39,44]. IgM heavy chain transcripts in liver were significantly up-regulated by handling stress but not by heat-shock in our study (Fig. 6D), and thus, GRP78 may be important for the proper folding of this immune relevant protein following exposure to only some types of stressor. GRP78 mRNA, which encodes the ER-resident member of the HSP70 family, was significantly up-regulated by heat-shock in all tissues studied. GRP94 (synonym: Gp96), the ER-resident member of the HSP90 family, is the major chaperone for the Toll-like receptors (TLRs) [40]. Yang et al. [40] demonstrated that Gp96 null mice were also macrophage-TLR null and highly susceptible to *Listeria *infections. In our study, GRP94 transcripts were significantly up-regulated in all tissues after heat-shock, with the head kidney presenting the highest up-regulation (25.1-fold) at 3ACS (Fig. 4B) relative to GRP94 mRNA levels before heat-shock. TLRs may play an important role in the defence against viral infections and have been shown to be up-regulated by the viral mimic pIC in fugu (*Takifugu rubripes*) [45].

Therefore, divergent forms of GRP gene sequences or different expression profiles of the mRNAs encoding these proteins between families and/or populations could play an important role in temperature-related immunosuppression. Given that TLR22 mRNA was significantly down-regulated in the head kidney of heat-shocked cod (Fig. 6A) when compared to its levels before heat-shock, and that this receptor in fish recognizes double-stranded RNA and induces genes of the interferon pathway [45], it is possible that its down-regulation following thermal stress results in reduced protein levels and is linked to decreased resistance to viruses in stressed fish [27] (a hypothesis we are currently testing). However, stress does not always correlate negatively with disease resistance. Weber et al. [46] have shown that a single 3 hour crowding event does not affect rainbow trout survival following a challenge with *Yersinia ruckeri *(the causative agent of enteric redmouth disease). On the other hand, the work of Fevolden et al. [47] indicates that the impact of stress on immune competence may be pathogen-specific. These authors have shown that rainbow trout strains selected for high cortisol response had lower survival rate when challenged with *A. salmonicida *(the causative agent of furunculosis), but higher survival rates when challenged with *Vibrio anguillarum *(the causative agent of vibriosis), when compared to strains selected for low cortisol response. Fast et al. [48] showed that Atlantic salmon (*Salmo salar*) subjected to long-term handling stress (i.e. once a day for 4 weeks) had reduced up-regulation of LPS (lipopolysaccharide)-induced macrophage IL-1β mRNA expression compared to control fish. In this study, chronic handling stress appeared to cause reduced immune competence as evidenced by the decreased survival of isolated macrophages from stressed fish (compared with macrophages from control, non-stressed fish) following incubation with *A. salmonicida *[48]. Clearly, the relationships between stress and immune responses in fish are complex and require further investigation.

Other cod transcripts encoding molecular chaperone-like proteins were identified in this work including several putative members of the T-complex-containing chaperones (CCT), prolyl-peptidyl-isomerase (PPIase, synonym: cyclophilin A), protein disulfide isomerase (PDI) (the two latter being classified as foldases, enzymes that catalyze reactions which accelerate protein folding and are an important part of the ER chaperone machinery) [44], and an ER-resident chaperone (HSP47) that is essential for the normal synthesis of procollagen and its stabilization during stress [49]. Collagen is an essential and ubiquitous component of the extracellular matrix and a potential target for denaturation and aggregation. We found that maximum up-regulation of HSP47 mRNA by heat-shock was at 3ACS in liver (20.4-fold), and at 12ACS in both skeletal muscle (16.9-fold) and head kidney (1.6-fold) when compared to its levels before heat-shock (Fig. 4E).

In mammals apoptosis induced by the denaturation and aggregation of proteins is one of the causes of death in heat-shocked cells [50]. Over-expression of the HSP70-1 protein (synonym: HSPA1A) plays an important role in protecting cells from apoptosis, presumably by preventing protein aggregation and inactivating the c-jun N-terminal kinase (JNK) pro-apoptotic pathway [50]. In our study, the mRNA encoding the putative cod orthologue of this particular chaperone was one of the most highly induced transcripts in the liver, with a 100.2-fold up-regulation at 12ACS (Fig. 4C) relative to the BHS time point. Although HSP70s have been shown to be anti-apoptotic in sea bream (*Sparus auratus*) primary macrophage cultures [51], previous studies have reported that HSP70-1 protein is not responsive to heat-stress in cod [29,32]. However as previously mentioned, these studies relied on anti-mouse HSC71/HSP70 or anti-rainbow trout HSP70 protein commercial antibodies, which may not efficiently cross-react with the orthologous HSP70 protein in cod. We have demonstrated that, at least at the transcriptional level, there was a significant up-regulation of a HSP70-1-like transcript in response to heat-shock. Moreover, the maximum-fold up-regulation of HSP70-1 mRNA at 12ACS in all tissues (Fig. 4C), is consistent with its reported role in acquired thermal tolerance during the recovery of mildly heat-shocked mammalian cells [52]. GRP78 is also known to protect cells against apoptosis, since it interacts with the key players in the ER stress signalling system (e.g. ATF6 and PERK) in non-stressed cells, preventing pro-apoptotic signalling [53]. Misfolded proteins in the ER interact with GRP78, which causes the activation of the pro-apoptotic ER stress signalling cascades [53]. Thus, up-regulation of the GRP78 transcripts may lead to elevated levels of this protein that would still be able to silence the pro-apoptotic ER stress signalling pathway. The up-regulation of NUPR1 (synonym: p8) mRNA may also lead to increased expression of this protein, and be an indication of increased levels of anti-apoptotic factors in both liver and head kidney. This protein has been correlated with reduced apoptosis in pancreatic cancer cells [54]. Protein aggregation is known to trigger apoptosis [3], and therefore, cell viability under thermal stress may depend on the ability to elicit a significant anti-apoptotic response through the expression of transcripts such as those encoding HSP70-1 and NUPR1. However, up-regulation of NUPR1 has also been linked to the acute phase response to pancreatitis in mammals [55]. We found that bikunin transcript, which also encodes an acute phase protein, was down-regulated by handling stress in the liver and by heat-shock in the head kidney. Thus, heat-shock may also affect the inflammatory response. In the spleen of Atlantic cod, bikunin transcript levels were not affected by saline control injection (which includes general handling stress), but were significantly suppressed by viral mimic (pIC) injection at 2 and 6 h post-injection, and significantly induced by the viral mimic at 24 h post-injection (these data relative to saline injected controls at these time points) [20]. Finally, it is worth noting that while NUPR1 was identified as a contig of 2 sequences in the reverse liver library (enriched for genes down-regulated by heat-shock), QPCR showed that this transcript was up-regulated by heat-shock in the liver and head kidney of cod. This was not surprising however, as in our hands, the SSH technique sometimes appears to be less effective at enriching for genes that are down-regulated by a stressor (i.e. in reverse subtractions) than at enriching for genes that are up-regulated by a stressor (i.e. in forward subtractions). As evidence of this, three out of four transcripts identified in a reverse spleen SSH library designed to be enriched for cod transcripts that were down-regulated by exposure to a stressor (viral mimic) could not be confirmed by QPCR as significantly down-regulated by the stressor (i.e. no statistically significant differences were detected) [20]. Therefore, the presence of NUPR1 as a contig of 2 sequences in the reverse liver library in the current study could be an artifact of the SSH technique.

The timing of up-regulation of some transcripts encoding putative chaperone proteins suggests that HSP90α may be a first line of defence against heat-stress, while HSP70-1 may be more important during recovery. Moreover, given that the mRNA expression of most of the studied chaperone genes peaked either at CS or 3ACS, we hypothesize that early time points may be crucial in the process of recovery and repair of damaged proteins. Two other transcripts, CCT 1 and CCT 5, putative members of the TCP1 complex, were significantly up-regulated by thermal stress in the liver at 3ACS (Fig. 5A and 5B). Of the 8 known mammalian members of this complex, we identified cDNAs for 6 putative orthologues in cod: CCT 1, 2, 3, 5, 6 and 8 [Tables 2 and 3, and Additional files 1 (Table S1) and 2 (Table S2)]. These chaperonins are known to form heterologous polymers that assist in the folding of actins and tubulins [56], important components of the cytoskeleton. Structural proteins seem to be among the most heat-labile proteins, and their misfolding and/or denaturation contributes greatly to protein aggregation [57].

Although we only saw a small, albeit significant, down-regulation (3.0 fold; CS-Fig. 5C) of TCTP mRNA expression in the head kidney in the CT group relative to the BHS time point, this transcript may still represent an important component of the molecular mechanism involved in thermal resistance. The product of the TCTP gene, a ubiquitously expressed protein in most mammalian cells, is known to bind to calcium and tubulin and to be responsive to stressors such as starvation and heat-stress [58]. In addition, Bonnet et al. [59] have shown that in yeast cells exposed to heat-shock there is a down-regulation of TCTP mRNA, and in rat (*Rattus norvegicus*) C6.9 glioma cells TCTP mRNA is up-regulated in response to induced programmed cell-death [60]. Down-regulation of TCTP in response to heat-shock in yeast may be one of the mechanisms that prevent heat-induced apoptosis, and it is possible that this down-regulation, which was detected in our experiments (e.g. 1.5 fold down-regulation in HS head kidney at CS), was not significant due to high variance between biological replicates (Fig. 5C). Down-regulation of TCTP may also play a role in preventing apoptosis triggered by other stressors (i.e. handling) since we found it to be significantly down-regulated by 3.0 fold at CS in the head kidney.

We saw little change in the mRNA expression of genes with carbohydrate metabolism related functional annotations (enolase, aldolase, PFK) (Fig. 5D, Table 2) with heat-shock at 18°C. However, this finding does not preclude the possibility that carbohydrate metabolism is increased when Atlantic cod are acutely exposed to elevated temperatures. This is because glycolysis had a relatively high prevalence (3.13%) amongst the biological process GO terms in the muscle forward library (Fig. 2). PFK and glycogen phosphorylase (identified in the head kidney forward library) are the rate limiting enzymes of glycolysis, and allosteric regulation of these enzymes is likely to be the main mechanism through which carbohydrate metabolism is re-organized during acute stress. Finally, the results of Perez-Casanova et al. [29] suggest that carbohydrate metabolism (based on measurements of plasma glucose) is not up-regulated significantly in cod until temperature reaches at least 20°C during acute thermal stress.

Interestingly, enolase transcript was significantly down-regulated (by 1.9-fold) in the head kidney of control transferred (CT) fish at the CS time point relative to the CT BHS time point, and GRP78 mRNA was significantly down-regulated in the liver in the CT group at all time points relative to the BHS time point. These results indicate that, even though there is a conserved general stress response, some responses at the transcriptome level are stressor specific (i.e. responsive to either heat-shock or handling stress).

## Conclusions

In conclusion, the present work adds significantly to the available data on the stress physiology of cod. We have contributed a total of 5980 ESTs (derived from all 4 SSH libraries) from three important stress-responsive tissues. Among these are several cDNAs encoding putative chaperones, which we have demonstrated to be responsive to heat-shock. Apoptosis and the aggregation of denatured proteins are likely to play a major role in heat-induced cell death in fish cells. SSH-identified transcripts (e.g. HSP90α, GRP94, GRP78, HSP70-1, HSP47) that were not only highly responsive to heat-shock, but also dysregulated in all three tissues studied, encode proteins that are known to prevent both programmed cell-death and aggregation. The functional genomics research reported herein may lead to the development of molecular markers (e.g. exonic, intronic, or regulatory SNPs associated with heat-stress responsive genes, and mRNA/protein expression profiles that correlate with thermal tolerance) that could be used for the selection of heat-resistant Atlantic cod broodstock for the aquaculture industry.

## Methods

### Heat-shock and sampling

One hundred and fifty juvenile cod (~35 g) from a single CGP family (06NL04) were divided equally into 3 × 250 L saltwater flow-through tanks (10°C, dissolved oxygen > 90% of air saturation). The tanks were then randomly assigned as control (C), control + handling stress (CT) and heat-shocked (HS), and fish were allowed to acclimate to their new environment for one week. In addition, another two tanks with the same water conditions were set up, and these tanks were adjacent to the tanks where the fish were stocked. During the one week acclimation period, the cod were fed 1.5% of their average body mass once daily. After the acclimation period, one of the two tanks that was set aside had the water flow interrupted and was heated to 18°C using a bayonet style immersion heater (8.4 A, 1000 W - Process Technologies, Tampa, FL), while the other tank was left as a 10°C flow-through tank. Fish from the CT group were quickly netted and transferred to the 10°C tank while fish belonging to the HS group were quickly netted and transferred to the 18°C tank. During this period, oxygen levels were constantly monitored in both tanks using a dissolved oxygen (DO) meter and probe (Oxyguard, HandiPolaris, Denmark), and pure oxygen was gently bubbled into the 18°C tank to maintain the DO levels above 90% of air saturation. The heat-shock lasted for 3 hours; after this period the immersion heater was turned off and water in the HS tank was quickly (within 10 minutes) brought back to 10°C by re-establishing the flow of 10°C water. Eight fish from each tank were sampled before the heat-shock (BHS; i.e. while still in their acclimation tank), at the cessation of the 3 hour heat-shock (i.e. when cold water flow was being re-established) (CS), and at 3, 12, and 24 hours after the cessation of heat-shock (ACS). For lethal sampling, fish were quickly netted from their tanks and placed in a bath containing an overdose of anaesthetic [400 mg of tricaine-methane-sulphonate (TMS) × L^-1^]. Blood, gills, head kidney, liver, and skeletal muscle samples were rapidly removed by team dissection, flash-frozen in liquid nitrogen and stored at -80°C until RNA extractions were performed. All sampling instruments were cleaned with RNase Away (Molecular BioProducts, San Diego, CA) between individuals. An aliquot of the blood was centrifuged at 5000 × *g *for 10 minutes at 10°C to separate plasma for cortisol determination.

### Plasma cortisol

Plasma cortisol levels were determined in duplicate using an enzyme-linked immunosorbent assay kit that has been previously validated for fish (Neogen Corp. Lexington, KY), and parallelism to the standard curve was confirmed using serially-diluted plasma samples [61]. Intra- and inter-assay variation was determined and never exceeded 10%. The cortisol data were analyzed statistically using MiniTab (Version 14). Data were tested for normality using the Anderson-Darling normality test. Treatment and time point were used in a general linear model with 2 crossed factors, considering all possible interactions between factors (A B A*B model). When the effects of each factor on a given variable were found to be significant (p < 0.05), two separate analyses were performed: 1) a one-way ANOVA within each group (e.g. CT) across sampling points was used to determine if values were different from their respective before heat-shock (BHS) values (p < 0.05); and 2) a one-way ANOVA within each sampling point was used to determine if the CT and HS groups were different from the undisturbed control (C) (p < 0.05). When groups were identified as significantly different through ANOVA, Tukey's multi-comparison pair-wise post-hoc test was used to test the hypothesis that means were significantly different between groups.

### RNA extractions

RNA was extracted from flash-frozen tissues using TRIzol reagent (Invitrogen, Carlsbad, CA) according to the manufacturer's instructions with modifications. Samples (~50 mg of tissue) were disrupted with disposable pestles and further homogenized using QIAshredder spin columns (QIAGEN, Mississauga, ON). The remainder of the protocol was carried out following the manufacturer's instructions. RNA samples were treated with DNase-I (QIAGEN) to degrade any residual genomic DNA and then purified from salts, proteins and nucleotides using RNeasy MinElute (QIAGEN) spin columns following the manufacturer's instructions. RNA quantity and quality were assessed using spectrophotometry and 1% agarose gel electrophoresis, respectively. Only high quality total RNA samples (260/280 ratio >1.8, with tight 18S/28S ribosomal RNA bands) were used for library construction and QPCR.

### mRNA isolation

Poly (A)^+ ^RNA (mRNA) was isolated from total RNA pools using the MicroPurist mRNA isolation kit (Ambion, Austin, TX) following the manufacturer's instructions. For each one of the tissues used for library construction [head kidney (HK), liver (L) and skeletal muscle (M)] DNase-I treated, column-cleaned total RNA samples from the CT and HS groups taken at sampling times CS, 3ACS, 12ACS, and 24ACS were pooled for mRNA isolation (n = 32 for each group within each tissue). Each one of the 32 individuals contributed an equal quantity of cleaned total RNA (HK = 10 μg; L = 8 μg; M = 10 μg) to each tissue/treatment group-specific pool. Purified mRNA quantity and quality were assessed by spectrophotometry and 1.5% agarose gel electrophoresis, respectively. Poly (A)^+ ^RNA yield ranged from 1.5 - 4% of total RNA.

### SSH library construction

Suppressive subtractive hybridization (SSH) was performed using the PCR-Select cDNA Subtraction Kit (Clontech, Mountain View, CA) and the manufacturer's instructions. Pooled HS mRNA samples were used as testers in the forward subtractions and as drivers in the reverse subtractions. Pooled CT samples were used as drivers in the forward subtractions and as testers in the reverse subtractions. "Forward subtracted" libraries were designed to be enriched for genes that were up-regulated by heat-shock, and "reverse subtracted" libraries were designed to be enriched for genes that were down-regulated by heat-shock. Two μg of mRNA were used for each first-strand cDNA synthesis. After second strand synthesis, cDNA samples were RsaI digested for 1.5 hours at 37°C. For SSH enrichment, two rounds of hybridization (6 h for the first hybridization and 16 h for the second hybridization) were performed in a hybridization oven at 68°C. All other procedures were performed according to the manufacturer's instructions.

The resulting SSH cDNA libraries were cloned into pGEM-T Easy (Promega, Madison, WI) vectors following the manufacturer's instructions. The ligation reactions were then transformed into chemically competent Max Efficiency DH5α cells (Invitrogen) using standard molecular biology techniques. Prior to sequencing, library insert size and complexity were evaluated as described in Rise et al. [20].

### DNA sequencing, sequence assembly and annotation

DNA sequencing, sequence assembly and annotation were done as described previously by Rise et al. [20]. Briefly, individual bacterial clones were inoculated into LB/glycerol/ampicillin in 384-well format and incubated overnight at 37°C. Sequencing reactions were carried out using ET terminator chemistry (GE Healthcare, Piscataway, NJ) and after removal of excess fluorescent terminators, samples were loaded onto MegaBACE (GE Healthcare) capillary sequencers. The resulting ESTs were analyzed for quality, trimmed and assembled as described by Rise et al. [20] using Phred [62,63] and Paracel Transcript Assembler (PTA). Each EST set resulting from different libraries (e.g. liver forward) was assembled separately. All ESTs were annotated by an automated pipeline using AutoFACT [26] and have been deposited in the GenBank dbEST under the accession numbers presented in Additional files 1 and 2 (Tables S1 and S2). One hundred seventy-nine ESTs were not submitted to GenBank due to low quality. Gene ontology (GO) annotation was obtained using AutoFACT [26] and GOblet [28]. Annotations presented within the text were obtained using BLASTx manually, and reflect a more updated state of the NCBI's non-redundant (nr) protein database. AutoFACT summary results are stored in the CGP EST database http://www.codgene.ca.

### cDNA synthesis and quantitative reverse transcription - polymerase chain reaction (QPCR)

Complementary DNA (cDNA) was synthesized from 1 μg of high quality, DNase-I treated, column-purified total RNA (the same individual samples that were pooled for SSH library construction) using the High Capacity Reverse Transcriptase Kit (Applied Biosystems, Foster City, CA) following the manufacturer's instructions.

Candidate stress responsive transcript levels were quantified by QPCR using Power SYBR Green I dye chemistry and the 7500 Fast Real-Time PCR System (Applied Biosystems). For QPCR studies, we used 6 individuals per treatment per time-point out of the 8 individuals that were used for SSH library construction.

The sequences of the primers used in mRNA expression analysis are presented in Table 4. Each primer set was tested for quality before use. QPCR primer quality control included running serial 1:5 dilutions (with the exception of TLR22 for which a 1:2 dilution was used) for both CT and HS samples using cDNA (a pool of 6 individuals sampled at CS) at a starting concentration of 10 ng of input total RNA in order to calculate amplification efficiency (*E *= 10^[-1/slope]^). We also performed melt-curves (+1% increases every 30 seconds from 60°C to 95°C) to verify that the primers amplified a single product and that there were no primer dimers or amplification in the no-template controls. Furthermore, random samples of each QPCR amplicon were subjected to 1.5% agarose gel electrophoresis with ethidium bromide staining and compared with a DNA size marker (100 bp ladder, Invitrogen) to confirm that the amplicons were of the expected sizes.

**Table 4:** Primers used in quantitative reverse transcription - polymerase chain reaction (QPCR)

Primer name	Sequence	Gene name of the best BLASTx hit	Amplification efficiency	Amplicon size (bp)
HSP90α Forward	5'- GAA CAA GAC CAA GCC CCT TT -3'	Heat shock protein 90 alpha	107%	133
HSP90α Reverse	5'- CTG ACC CTC CAC CGA GAA GT -3'			

GRP94 Forward	5'- AGT GTT TCT CTC GAC ACG TTC A -3'	Heat shock protein 90 kDa beta, member 1 (synonym: GRP94)	91%	97
GRP94 Reverse	5'- CAG ACG ACT TCC ATG ACA TGA T -3'			

HSP70-1 Forward	5'- GAG AAC AAG ATC ACC ATC ACG A -3'	HSP70-1 (synonym: *HSPA1A*)	97%	123
HSP70-1 Reverse	5'- GGC TGT TAC TTT CTC TCC CTG A -3'			

GRP78 Forward	5'- CTC CTT CAT TTT GGT CAG AAC C -3'	78 kDa glucose-regulated protein (synonym: GRP78)	90%	132
GRP78 Reverse	5'- CTC AAG TTC CTC CCA TTC AAA G -3'			

HSP47 Forward	5'- ATG GAA GTC AGC CAC AAC CT -3'	Heat Shock Protein 47	93%	100
HSP47 Reverse	5'- TCT TGC CCG TGA TGT TAG AC -3'			

CCT1 Forward	5'- GCA GGC GTT TGG GAT AAC TA -3'	T-complex-1	95%	127
CCT1 Reverse	5'- GCG CTT AAC CCT TCA GAG AA -3'			

CCT5 Forward	5'- CCA GGC GAG GTT GAA GAA TA -3'	Chaperonin containing TCP1, subunit	92%	91
CCT5 Reverse	5'- TAG AAC AGG GGA GTG GTG GT -3'	5 (epsilon)		

TCTP Forward	5'- ACC AAG CCA GAG AGA GTG GA -3'	Translationally-controlled tumor protein	101%	147
TCTP Reverse	5'- ATC CTC ACG GAA GTC AAG CA -3'			

Enolase Forward	5'- GGA CGG CAC TGA AAA CAA AT -3'	Enolase 3	93%	115
Enolase Reverse	5'- ACA GAG GAA CCC CCT TCT CC -3'			

PFK Forward^1^	5'- TGT TTG CCA ACT CCC CAG AGA -3'	Phosphofructokinase, muscle a	96%	121
PFK Reverse^1^	5'- TCC GGT GCT TGA AGT CTG TCA -3'			

Aldolase Forward^1^	5'- TGA CAT TGC TCA GAG GAT GG -3'	Aldolase B	91%	143
Aldolase Reverse^1^	5'- TAG CGA CGG TTC TCC TCA CT -3'			

Bikunin Forward	5'- GCC ACT GAG TTC ACA GAC G -3'	Alpha-1-microglobulin/bikunin precursor	90%	107
Bikunin Reverse	5'- CAG CTC ATG GAG GAG GAG T -3'			

IgM Forward	5'- GAG CAT CCA CTG GCT CTT TA -3'	Immunoglobulin heavy chain, secretory form	93%	97
IgM Reverse	5'- GCA GCA AGC TAT ATC CAG GT -3'			

Nupr1 Forward	5'- CTT TCT TCT CGC TGT TCT GC -3'	Nuclear protein 1	88%	101
Nupr1 Reverse	5'- GGA AGG ACC AAG AAG GAG TC -3'			

IL-8 Forward	5'- CTT CAG CAT CCA GAC AGA CC -3'	Interleukin-8	100%	133
IL-8 Reverse	5'- CAG ACA GAG AGC CGT CAG AT -3'			

TLR22 Forward	5'- TGC AGG TAA TCA CGA CTG AC -3'	TLR22	85%	93
TLR22 Reverse	5'- GAG ACT TCC AGC CAG ACC TA -3'			

18S Forward	5'- ATG GCC GTT CTT AGT TGG TG -3'	18S ribosomal RNA (normalizer gene)	109%	180
18S Reverse	5'- GGA CAT TTA AGG GCG TCT CA -3'			

^1^The primers for aldolase and PFK were designed to amplify a region that is conserved between the muscle and liver forms of these genes.

PCR amplification was performed with the 7500 Fast Real-Time PCR System (Applied Biosystems) in 13 μl reactions using 2 μl of cDNA (10 ng of input total RNA), 50 nM each of forward and reverse primer and 1× Power SYBR Green PCR Master Mix (Applied Biosystems). Expression levels of the genes of interest were normalized to 18S ribosomal RNA. The suitability of 18S as a normalizer was confirmed by calculating the standard deviation (SD) of all 18S fluorescence threshold cycle (C_T_) values for a given tissue. The highest SD found was 0.36 in the muscle, with values of 0.28 and 0.30 in the head kidney and liver, respectively. The average 18S C_T_ values were 25.66, 25.90, and 27.99 for muscle, head kidney and liver, respectively. Moreover, the average 18S C_T_ for each group (e.g. HS) was calculated and shown not to differ by more than 0.3 cycles from the average of any other group. The QPCR cycling parameters consisted of 1 cycle of 50°C for 5 minutes to activate AmpErase Uracil N-glycosylase (UNG), 1 cycle of 95°C for 10 minutes, and 40 cycles of (95°C for 15 sec and 60°C for 1 minute). On a given 96- well plate, target and normalizer genes were run in duplicate [64]. The C_T _values were determined using the 7500 Software Relative Quantification Study Application (Version 2.0) (Applied Biosystems) with automated threshold determination and walking baseline. Each data set from a tissue was analyzed as a multi-plate study. The relative starting quantity (RQ) of each transcript was determined using the comparative C_T _method for relative quantification [65], using the individual with the lowest gene of interest expression (i.e. lowest normalized expression) within a given tissue as calibrator.

Calculated amplification efficiencies (Table 4) were used to calculate RQs. Overall fold up-regulation for each group (e.g. HS at CS) was calculated as (average RQ)/(average RQ for the appropriate group at BHS). Overall fold down-regulation (if applicable) was calculated as the inverse of overall fold up-regulation.

The RQs obtained from the software were statistically analyzed using MiniTab (Version 14). Data were tested for normality using the Anderson-Darling normality test. Treatment and time point were then used in a general linear model with 2 crossed factors, considering all possible interactions between factors (A B A*B model). When the effects of each factor on a given variable were found to be significant (p < 0.05) two separate analyses were performed. An one-way ANOVA in each group (e.g. CT) was used across sampling points to determine if groups (e.g. CT) were different from their respective BHS values (p < 0.05); when values were significantly different, Tukey's multi-comparison pairwise post-hoc test was used. A t-test within each sampling point was used to determine if the HS group was different from the CT group (p < 0.05).

## Authors' contributions

TSH was involved in the conceptualization, design, and implementation of all experiments, and took the lead role in data analysis, interpretation of results, and writing of this manuscript. AKG is the first author's (TSH) Ph.D. co-supervisor, was involved in the conceptualization, design and implementation of experiments, and took an active part in data interpretation and the writing of this manuscript. LOBA was involved in the conceptualization, design, and implementation of experiments, and in the interpretation of data. SCJ was involved in the conceptualization and design of experiments, and in the interpretation of data. SH was involved in the characterization of SSH libraries (e.g. sequencing, sequence trimming and assembly, and BLAST identification and functional annotation of assembled sequences). JK was involved in the characterization of SSH libraries (e.g. sequencing, sequence trimming and assembly, and BLAST identification and functional annotation of assembled sequences). SB was involved in conceptualizing experiments and characterizing SSH libraries. MLR, the first author's (TSH) Ph.D. co-supervisor, was involved in the conceptualization, design, and implementation of SSH and QPCR-based experiments, and took an active part in the interpretation of data and the writing of this manuscript. All authors read and approved the final manuscript.

## Supplementary Material

Additional file 1 **Supplemental Table S1, assembled ESTs (contigs and singletons) in the forward heat-shock SSH libraries**. Contains 3 tables (S1 A-C) with information such as supporting annotations, statistics, and contributing EST accession numbers of contigs and singletons found in all 3 forward libraries. Click here for file

Additional file 2 **Supplemental Table S2, assembled ESTs (contigs and singletons) in the reverse heat-shock SSH libraries**. Contains 2 tables (S2A and S2B) with information such as supporting annotations, statistics, and contributing EST accession numbers of contigs and singletons found in the 2 reverse libraries that were sequenced (head kidney and liver). Click here for file

Additional file 3 **Supplemental Table S3, summary of GO annotation**. Contains 4 tables (S3 A-D) with summaries of percentages of total ESTs with GO (biological process) terms for each of 4 SSH libraries. Click here for file
