# Supplementary material for: Heat-shock responsive genes identified and validated in Atlantic cod (Gadus morhua) liver, head kidney and skeletal muscle using genomic techniques
Source: BMC Genomics. 2010 Jan 28;11:72. doi: 10.1186/1471-2164-11-72 (PMC2830189; doi:10.1186/1471-2164-11-72)
Supplement: Additional file 1 — Supplemental Table S1, assembled ESTs (contigs and singletons) in the forward heat-shock SSH libraries. Contains 3 tables (S1 A-C) with information such as supporting annotations, statistics, and contributing EST accession numbers of contigs and singletons found in all 3 forward libraries. [file 1471-2164-11-72-S1.PDF]

Supplemental Table S1A. Contigs in library gmnlkfta (forward heat-shock head kidney SSH library) with supporting annotations[1], statistics, and contributing EST accession numbers

Sequence	Count	Sub-sequences	AutoFACT Description	GO terms	E-value	Identity	Accession Number
sb_gmnlkfta.88.C1	17	sb_gmnlkfta_0007e23.t7<br>sb_gmnlkfta_0003d02.t7<br>sb_gmnlkfta_0007f06.t7<br>sb_gmnlkfta_0006g21.t7<br>sb_gmnlkfta_0006i21.t7<br>sb_gmnlkfta_0006m23.t7<br>sb_gmnlkfta_0006b13.t7<br>sb_gmnlkfta_0002f09.t7<br>sb_gmnlkfta_0002k18.t7<br>sb_gmnlkfta_0006a15.t7<br>sb_gmnlkfta_0006n17.t7<br>sb_gmnlkfta_0006o23.t7<br>sb_gmnlkfta_0006p11.t7<br>sb_gmnlkfta_0002i04.t7<br>sb_gmnlkfta_0007i14.t7<br>sb_gmnlkfta_0004i01.t7<br>sb_gmnlkfta_0004e12.t7	Cluster: Hemoglobin subunit alpha-1; n=3; Gadidae|Rep: Hemoglobin subunit alpha-1 - Gadus morhua (Atlantic cod)	GO:0020037<br>GO:0019825<br>GO:0015671<br>GO:0006810<br>GO:0005833<br>GO:0005506<br>GO:0005344	3e-48	93% (94/101)	EY973521<br>ES780139<br>EY973528<br>EX190153<br>EX190192<br>EX190276<br>EX190039<br>ES779912<br>ES780010<br>EX190018<br>EX190291<br>EX190319<br>EX190328<br>ES779964<br>EY973584<br>ES780417<br>ES780364
sb_gmnlkfta.33.C1	10	sb_gmnlkfta_0003o13.t7<br>sb_gmnlkfta_0003h22.t7<br>sb_gmnlkfta_0007e22.t7<br>sb_gmnlkfta_0007d20.t7<br>sb_gmnlkfta_0007n08.t7<br>sb_gmnlkfta_0003h24.t7<br>sb_gmnlkfta_0007g03.t7<br>sb_gmnlkfta_0006p15.t7<br>sb_gmnlkfta_0006g23.t7<br>sb_gmnlkfta_0007h04.t7	LSU rRNA; Xenopus borealis		1e-168	97% (323/331)	ES780279<br>ES780197<br>EY973520<br>EY973501<br>EY973662<br>ES780199<br>EY973541<br>EX190332<br>EX190155<br>EY973557
sb_gmnlkfta.1.C1	9	sb_gmnlkfta_0006l08.t7<br>sb_gmnlkfta_0003e04.t7<br>sb_gmnlkfta_0003g04.t7<br>sb_gmnlkfta_0004h13.t7<br>sb_gmnlkfta_0006e16.t7<br>sb_gmnlkfta_0007i05.t7<br>sb_gmnlkfta_0006k24.t7<br>sb_gmnlkfta_0006a24.t7<br>sb_gmnlkfta_0007f05.t7	Cluster: Ribosomal protein L21; n=5; Clupeocephala|Rep: Ribosomal protein L21 - Danio rerio (Zebrafish) (Brachydanio rerio)		3e-50	85% (91/106)	EX190243<br>ES780152<br>ES780176<br>ES780408<br>EX190105<br>EY973576<br>EX190236<br>EX190027<br>EY973527
sb_gmnlkfta.16.C1	9	sb_gmnlkfta_0007j08.t7<br>sb_gmnlkfta_0002f16.t7<br>sb_gmnlkfta_0007h08.t7<br>sb_gmnlkfta_0002l13.t7<br>sb_gmnlkfta_0002j11.t7<br>sb_gmnlkfta_0002m16.t7<br>sb_gmnlkfta_0001h05.t7<br>sb_gmnlkfta_0002a02.t7<br>sb_gmnlkfta_0007n17.t7	Cluster: PREDICTED: similar to ribosomal protein L10a; n=1; Pan troglodytes|Rep: PREDICTED: similar to ribosomal protein L10a - Pan troglodytes		5e-71	78% (138/176)	EY973597<br>ES779919<br>EY973561<br>ES780024<br>ES779985<br>ES780047<br>ES779806<br>ES779815<br>EY973670

sb_gmnlkfta.76.C1	7	sb_gmnlkfta_0004n13.t7<br>sb_gmnlkfta_0002k17.t7<br>sb_gmnlkfta_0004h18.t7<br>sb_gmnlkfta_0004l13.t7<br>sb_gmnlkfta_0006c11.t7<br>sb_gmnlkfta_0006m07.t7<br>sb_gmnlkfta_0006a19.t7	Cluster: Ribosomal protein S8; n=22; Euteleostomi|Rep: Ribosomal protein S8 - Mus musculus (Mouse)		2e-47	95% (89/93)	ES780515<br>ES780009<br>ES780413<br>ES780476<br>EX190059<br>EX190263<br>EX190022
sb_gmnlkfta.85.C1	7	sb_gmnlkfta_0002g08.t7<br>sb_gmnlkfta_0007j07.t7<br>sb_gmnlkfta_0002a15.t7<br>sb_gmnlkfta_0007f17.t7<br>sb_gmnlkfta_0003a15.t7<br>sb_gmnlkfta_0007o03.t7<br>sb_gmnlkfta_0007g01.t7	Cluster: Translationally-controlled tumor protein; n=6; Otophysi|Rep: Translationally-controlled tumor protein - Danio rerio (Zebrafish) (Brachydanio rerio)		6e-53	65% (79/121)	ES779932<br>EY973596<br>ES779823<br>EY973535<br>ES780109<br>EY973676<br>EY973539
sb_gmnlkfta.104.C1	6	sb_gmnlkfta_0006d20.t7<br>sb_gmnlkfta_0006c16.t7<br>sb_gmnlkfta_0006j20.t7<br>sb_gmnlkfta_0006o16.t7<br>sb_gmnlkfta_0004c20.t7<br>sb_gmnlkfta_0004c14.t7	unclassified				EX190087<br>EX190064<br>EX190214<br>EX190314<br>ES780336<br>ES780332
sb_gmnlkfta.20.C1	6	sb_gmnlkfta_0007i09.t7<br>sb_gmnlkfta_0004i03.t7<br>sb_gmnlkfta_0006i09.t7<br>sb_gmnlkfta_0006m19.t7<br>sb_gmnlkfta_0006o19.t7<br>sb_gmnlkfta_0007f10.t7	Cluster: 40S ribosomal protein S27; n=8; Tetrapoda|Rep: 40S ribosomal protein S27 - Homo sapiens (Human)		1e-36	86% (73/84)	EY973580<br>ES780418<br>EX190183<br>EX190272<br>EX190316<br>EY973531
sb_gmnlkfta.37.C1	5	sb_gmnlkfta_0004g09.t7<br>sb_gmnlkfta_0002f01.t7<br>sb_gmnlkfta_0003l16.t7<br>sb_gmnlkfta_0006p13.t7<br>sb_gmnlkfta_0006d17.t7	Cluster: Taldo1 protein; n=4; Danio rerio|Rep: Taldo1 protein - Danio rerio (Zebrafish) (Brachydanio rerio)		2e-63	82% (120/145)	ES780395<br>ES779906<br>ES780245<br>EX190330<br>EX190084
sb_gmnlkfta.64.C1	5	sb_gmnlkfta_0004f10.t7<br>sb_gmnlkfta_0003g09.t7<br>sb_gmnlkfta_0007e06.t7<br>sb_gmnlkfta_0004b11.t7<br>sb_gmnlkfta_0001c04.t7	unclassified				ES780378<br>ES780180<br>EY973509<br>ES780314<br>ES779749
sb_gmnlkfta.7.C1	5	sb_gmnlkfta_0006e09.t7<br>sb_gmnlkfta_0001c08.t7<br>sb_gmnlkfta_0006m09.t7<br>sb_gmnlkfta_0006k11.t7<br>sb_gmnlkfta_0006k15.t7	IGHA2; similar to Ig alpha-2 chain C region		4e-06	27% (38/138)	EX190099<br>ES779753<br>EX190264<br>EX190226<br>EX190229

sb_gmnlkfta.78.C1	5	sb_gmnlkfta_0002d09.t7<br>sb_gmnlkfta_0004b12.t7<br>sb_gmnlkfta_0007j11.t7<br>sb_gmnlkfta_0004o18.t7<br>sb_gmnlkfta_0007f04.t7	LSU rRNA; Squalus acanthias		1e-131	99%<br>(236/237)	ES779873<br>ES780315<br>EY973599<br>ES780536<br>EY973526
sb_gmnlkfta.82.C1	5	sb_gmnlkfta_0007l15.t7<br>sb_gmnlkfta_0002m18.t7<br>sb_gmnlkfta_0003b13.t7<br>sb_gmnlkfta_0002m12.t7<br>sb_gmnlkfta_0007p11.t7	unclassified				EY973639<br>ES780049<br>ES780117<br>ES780044<br>EY973688
sb_gmnlkfta.94.C1	5	sb_gmnlkfta_0006j05.t7<br>sb_gmnlkfta_0004o17.t7<br>sb_gmnlkfta_0006d03.t7<br>sb_gmnlkfta_0006n05.t7<br>sb_gmnlkfta_0004a07.t7	Cluster: PREDICTED: similar to NCK associated protein 1 like; n=1; Danio rerio|Rep: PREDICTED: similar to NCK associated protein 1 like - Danio rerio		1e-28	61%<br>(65/106)	EX190200<br>ES780535<br>EX190074<br>EX190280<br>ES780298
sb_gmnlkfta.103.C1	4	sb_gmnlkfta_0006l23.t7<br>sb_gmnlkfta_0006f19.t7<br>sb_gmnlkfta_0003o15.t7<br>sb_gmnlkfta_0002a04.t7	eif4a3; eukaryotic translation initiation factor 4A, isoform 3; K03257 translation initiation factor eIF-4A		1e-56	100%<br>(107/107)	EX190257<br>EX190129<br>ES780281<br>ES779816
sb_gmnlkfta.109.C1	4	sb_gmnlkfta_0007d13.t7<br>sb_gmnlkfta_0006i05.t7<br>sb_gmnlkfta_0006m06.t7<br>sb_gmnlkfta_0006k06.t7	Unassigned protein				EY973495<br>EX190179<br>EX190262<br>EX190222
sb_gmnlkfta.11.C1	4	sb_gmnlkfta_0004k15.t7<br>sb_gmnlkfta_0003k24.t7<br>sb_gmnlkfta_0003l22.t7<br>sb_gmnlkfta_0004l15.t7	unclassified				ES780457<br>ES780235<br>ES780248<br>ES780478
sb_gmnlkfta.136.C1	4	sb_gmnlkfta_0004d24.t7<br>sb_gmnlkfta_0004e10.t7<br>sb_gmnlkfta_0006a07.t7<br>sb_gmnlkfta_0006o15.t7	Cluster: Homolog of Homo sapiens "UDP-glucuronosyltransferase 2A1 precursor, microsomal; n=1; Takifugu rubripes|Rep: Homolog of Homo sapiens "UDP-glucuronosyltransferase 2A1 precursor, microsomal - Takifugu rubripes		7e-57	81%<br>(70/86)	ES780355<br>ES780362<br>EX190010<br>EX190313
sb_gmnlkfta.142.C1	4	sb_gmnlkfta_0006l13.t7<br>sb_gmnlkfta_0006b11.t7<br>sb_gmnlkfta_0006j09.t7	Cluster: Replication factor C subunit RFC4; n=3; Danio rerio|Rep: Replication factor C subunit RFC4 - Danio rerio (Zebrafish)		5e-50	71%	EX190248<br>EX190037<br>EX190204

		sb_gmnlkfta_0006j13.t7	(Brachydanio rerio)			(96/134)	EX190208
sb_gmnlkfta.143.C1	4	sb_gmnlkfta_0004o06.t7<br>sb_gmnlkfta_0007b10.t7<br>sb_gmnlkfta_0006j16.t7<br>sb_gmnlkfta_0006d24.t7	Cluster: Proteasome subunit beta type-1-A; n=6; Clupeocephala|Rep: Proteasome subunit beta type-1-A - Carassius auratus (Goldfish)	GO:0016787<br>GO:0008233<br>GO:0006511<br>GO:0005839<br>GO:0005829<br>GO:0005737<br>GO:0005634<br>GO:0004298	2e-50	90%<br>(93/103)	ES780527<br>EY973462<br>EX190211<br>EX190091
sb_gmnlkfta.203.C1	4	sb_gmnlkfta_0002k03.t7<br>sb_gmnlkfta_0002m06.t7<br>sb_gmnlkfta_0003e14.t7<br>sb_gmnlkfta_0004k02.t7	unclassified				ES779998<br>ES780039<br>ES780156<br>ES780446
sb_gmnlkfta.24.C1	4	sb_gmnlkfta_0006p05.t7<br>sb_gmnlkfta_0006l24.t7<br>sb_gmnlkfta_0007j10.t7<br>sb_gmnlkfta_0007p08.t7	Unassigned protein				EX190323<br>EX190258<br>EY973598<br>EY973685
sb_gmnlkfta.46.C1	4	sb_gmnlkfta_0001f04.t7<br>sb_gmnlkfta_0002l09.t7<br>sb_gmnlkfta_0004l22.t7<br>sb_gmnlkfta_0002k10.t7	unclassified				ES779782<br>ES780020<br>ES780485<br>ES780004
sb_gmnlkfta.47.C1	4	sb_gmnlkfta_0004e02.t7<br>sb_gmnlkfta_0004m02.t7<br>sb_gmnlkfta_0004o22.t7<br>sb_gmnlkfta_0002f24.t7	Cluster: Small nuclear ribonucleoprotein Sm D3; n=16; Gnathostomata|Rep: Small nuclear ribonucleoprotein Sm D3 - Homo sapiens (Human)		8e-37	88%<br>(82/93)	ES780356<br>ES780488<br>ES780539<br>ES779926
sb_gmnlkfta.51.C1	4	sb_gmnlkfta_0004j10.t7<br>sb_gmnlkfta_0007m18.t7<br>sb_gmnlkfta_0007p07.t7<br>sb_gmnlkfta_0007i12.t7	Cluster: Proline-serine-threonine phosphatase-interacting protein 2.; n=2; Gallus gallus|Rep: Proline-serine-threonine phosphatase-interacting protein 2. - Gallus gallus		2e-64	55%<br>(124/222)	ES780435<br>EY973654<br>EY973684<br>EY973583
sb_gmnlkfta.61.C1	4	sb_gmnlkfta_0002g18.t7<br>sb_gmnlkfta_0002p06.t7<br>sb_gmnlkfta_0006d02.t7<br>sb_gmnlkfta_0007b19.t7	Cluster: Signal sequence receptor, delta; n=2; Danio rerio|Rep: Signal sequence receptor, delta - Danio rerio (Zebrafish) (Brachydanio rerio)		2e-67	87%<br>(123/141)	ES779940<br>ES780091<br>EX190073<br>EY973466

sb_gmnlkfta.62.C1	4	sb_gmnlkfta_0006f13.t7<br>sb_gmnlkfta_0006a23.t7<br>sb_gmnlkfta_0003g16.t7<br>sb_gmnlkfta_0006d11.t7	unclassified				EX190123<br>EX190026<br>ES780184<br>EX190079
sb_gmnlkfta.65.C1	4	sb_gmnlkfta_0007c08.t7<br>sb_gmnlkfta_0007a19.t7<br>sb_gmnlkfta_0004n10.t7<br>sb_gmnlkfta_0007d10.t7	Cluster: HSP90AA1 protein; n=6; Euteleostomi|Rep: HSP90AA1 protein - Homo sapiens (Human)		3e-47	93% (93/99)	EY973473<br>EY973458<br>ES780512<br>EY973494
sb_gmnlkfta.67.C1	4	sb_gmnlkfta_0001h06.t7<br>sb_gmnlkfta_0007a11.t7<br>sb_gmnlkfta_0007f22.t7<br>sb_gmnlkfta_0007o05.t7	Cluster: Homolog of Homo sapiens "PePtidylProlyl isomerase A-like; n=1; Takifugu rubripes|Rep: Homolog of Homo sapiens "PePtidylProlyl isomerase A-like - Takifugu rubripes		3e-71	82% (129/156)	ES779807<br>EY973455<br>EY973538<br>EY973678
sb_gmnlkfta.106.C1	3	sb_gmnlkfta_0004n24.t7<br>sb_gmnlkfta_0006i04.t7<br>sb_gmnlkfta_0007n13.t7	unclassified				ES780525<br>EX190178<br>EY973666
sb_gmnlkfta.110.C1	3	sb_gmnlkfta_0002g12.t7<br>sb_gmnlkfta_0003k19.t7<br>sb_gmnlkfta_0002a21.t7	unclassified				ES779936<br>ES780233<br>ES779829
sb_gmnlkfta.114.C1	3	sb_gmnlkfta_0003c13.t7<br>sb_gmnlkfta_0004h02.t7<br>sb_gmnlkfta_0007l05.t7	Cluster: Zgc:114195; n=2; Danio rerio|Rep: Zgc:114195 - Danio rerio (Zebrafish) (Brachydanio rerio)		1e-84	75% (143/189)	ES780129<br>ES780404<br>EY973632
sb_gmnlkfta.115.C1	3	sb_gmnlkfta_0002m13.t7<br>sb_gmnlkfta_0003m04.t7<br>sb_gmnlkfta_0002a09.t7	unclassified				ES780045<br>ES780251<br>ES779819
sb_gmnlkfta.121.C1	3	sb_gmnlkfta_0003a06.t7<br>sb_gmnlkfta_0004b21.t7<br>sb_gmnlkfta_0004m14.t7	Cluster: Heat shock cognate 71 kDa protein; n=25; Clupeocephala|Rep: Heat shock cognate 71 kDa protein - Danio rerio (Zebrafish) (Brachydanio rerio)		3e-36	68% (77/113)	ES780105<br>ES780321<br>ES780498
sb_gmnlkfta.123.C1	3	sb_gmnlkfta_0002h21.t7<br>sb_gmnlkfta_0003l18.t7	Cluster: Ribosomal protein L12; n=7; Euteleostomi|Rep: Ribosomal protein L12 - Danio rerio (Zebrafish) (Brachydanio rerio)		9e-42	94% (86/91)	ES779958<br>ES780246<br>ES780426

		sb_gmnlkfta_0004i22.t7					
sb_gmnlkfta.137.C1	3	sb_gmnlkfta_0001e09.t7<br>sb_gmnlkfta_0002i05.t7<br>sb_gmnlkfta_0006e08.t7	unclassified				ES779776<br>ES779965<br>EX190098
sb_gmnlkfta.144.C1	3	sb_gmnlkfta_0002e15.t7<br>sb_gmnlkfta_0006l12.t7<br>sb_gmnlkfta_0003a05.t7	Cluster: Ribosomal protein S13; n=7; Euteleostomi|Rep: Ribosomal protein S13 - Siniperca chuatsi (Chinese perch)	GO:0006412<br>GO:0005840<br>GO:0005622<br>GO:0003735	3e-42	94%<br>(86/91)	ES779897<br>EX190247<br>ES780104
sb_gmnlkfta.145.C1	3	sb_gmnlkfta_0006j12.t7<br>sb_gmnlkfta_0006l07.t7<br>sb_gmnlkfta_0006l10.t7	Cluster: Zgc:92063; n=1; Danio rerio|Rep: Zgc:92063 - Danio rerio (Zebrafish) (Brachydanio rerio)		2e-43	86%<br>(84/97)	EX190207<br>EX190242<br>EX190245
sb_gmnlkfta.150.C1	3	sb_gmnlkfta_0003i18.t7<br>sb_gmnlkfta_0002i03.t7<br>sb_gmnlkfta_0002k19.t7	unclassified				ES780211<br>ES779963<br>ES780011
sb_gmnlkfta.155.C1	3	sb_gmnlkfta_0007k19.t7<br>sb_gmnlkfta_0004k18.t7<br>sb_gmnlkfta_0006g13.t7	Cluster: Mitochondrial carrier-like 2; n=1; Oreochromis mossambicus|Rep: Mitochondrial carrier-like 2 - Oreochromis mossambicus (Mozambique tilapia) (Tilapia mossambica)	GO:0016020<br>GO:0006810<br>GO:0005488	5e-30	90%<br>(60/66)	EY973625<br>ES780460<br>EX190145
sb_gmnlkfta.157.C1	3	sb_gmnlkfta_0004l03.t7<br>sb_gmnlkfta_0007m05.t7<br>sb_gmnlkfta_0002j21.t7	Cluster: Cystatin-F precursor (Leukocystatin) (Cystatin-7) (Cystatin-like metastasis-associated protein) (CMAP).; n=1; Takifugu rubripes|Rep: Cystatin-F precursor (Leukocystatin) (Cystatin-7) (Cystatin-like metastasis-associated protein) (CMAP). - Takifugu rubripes		3e-23	47%<br>(56/118)	ES780467<br>EY973648<br>ES779993
sb_gmnlkfta.160.C1	3	sb_gmnlkfta_0006f24.t7<br>sb_gmnlkfta_0006b17.t7<br>sb_gmnlkfta_0006h13.t7	unclassified				EX190134<br>EX190043<br>EX190165
sb_gmnlkfta.168.C1	3	sb_gmnlkfta_0002m20.t7<br>sb_gmnlkfta_0004m22.t7<br>sb_gmnlkfta_0007b15.t7	unclassified				ES780051<br>ES780503<br>EY973464
sb_gmnlkfta.169.C1	3	sb_gmnlkfta_0002b20.t7<br>sb_gmnlkfta_0002i02.t7	Cluster: Peptidyl-prolyl cis-trans isomerase; n=1; Tetraodon nigroviridis|Rep: Peptidyl-prolyl cis-trans isomerase - Tetraodon	GO:0006457	5e-59	72%	ES779843<br>ES779962<br>ES780018

		sb_gmnlkfta_0002l04.t7	nigroviridis (Green puffer)	GO:0003755		(109/151)	
sb_gmnlkfta.175.C1	3	sb_gmnlkfta_0002f18.t7<br>sb_gmnlkfta_0006j15.t7<br>sb_gmnlkfta_0003a11.t7	unclassified				ES779921<br>EX190210<br>ES780108
sb_gmnlkfta.176.C1	3	sb_gmnlkfta_0006g20.t7<br>sb_gmnlkfta_0006i22.t7<br>sb_gmnlkfta_0006m20.t7	Cluster: Zgc:77929; n=1; Danio rerio|Rep: Zgc:77929 - Danio rerio (Zebrafish) (Brachydanio rerio)		4e-06	59% (25/42)	EX190152<br>EX190193<br>EX190273
sb_gmnlkfta.18.C1	3	sb_gmnlkfta_0002e07.t7<br>sb_gmnlkfta_0004b19.t7<br>sb_gmnlkfta_0006l06.t7	unclassified				ES779891<br>ES780320<br>EX190241
sb_gmnlkfta.180.C1	3	sb_gmnlkfta_0002i06.t7<br>sb_gmnlkfta_0002m01.t7<br>sb_gmnlkfta_0004d06.t7	unclassified				ES779966<br>ES780034<br>ES780342
sb_gmnlkfta.198.C1	3	sb_gmnlkfta_0006b02.t7<br>sb_gmnlkfta_0003n17.t7<br>sb_gmnlkfta_0006n11.t7	Cluster: Actin-related protein 2/3 complex subunit 5; n=5; Danio rerio|Rep: Actin-related protein 2/3 complex subunit 5 - Danio rerio (Zebrafish) (Brachydanio rerio)		1e-39	82% (83/101)	EX190028<br>ES780271<br>EX190285
sb_gmnlkfta.211.C1	3	sb_gmnlkfta_0006f03.t7<br>sb_gmnlkfta_0006f05.t7<br>sb_gmnlkfta_0006d05.t7	unclassified				EX190115<br>EX190117<br>EX190075
sb_gmnlkfta.217.C1	3	sb_gmnlkfta_0004d07.t7<br>sb_gmnlkfta_0001h10.t7<br>sb_gmnlkfta_0007i01.t7	HMG14_17 multi-domain protein		1e-06	42% (27/64)	ES780343<br>ES779811<br>EY973573
sb_gmnlkfta.23.C1	3	sb_gmnlkfta_0006d14.t7<br>sb_gmnlkfta_0006f15.t7<br>sb_gmnlkfta_0006h17.t7	Cluster: Torsin B precursor (Torsin family 1 member B).; n=1; Takifugu rubripes|Rep: Torsin B precursor (Torsin family 1 member B). - Takifugu rubripes		1e-80	79% (140/177)	EX190082<br>EX190125<br>EX190169
sb_gmnlkfta.27.C2	3	sb_gmnlkfta_0002f20.t7<br>sb_gmnlkfta_0003k13.t7	Cluster: PREDICTED: similar to bloodthirsty; n=3; Danio rerio|Rep: PREDICTED: similar to bloodthirsty - Danio rerio		7e-22	59% (57/96)	ES779923<br>ES780229<br>ES780127

		sb_gmnlkfta_0003c11.t7						
sb_gmnlkfta.41.C1	3	sb_gmnlkfta_0007j14.t7<br>sb_gmnlkfta_0002c21.t7<br>sb_gmnlkfta_0002l24.t7	Cluster: Superoxide dismutase [Cu-Zn]; n=4; Perciformes|Rep: Superoxide dismutase [Cu-Zn] - Epinephelus coioides (Orange-spotted grouper)	GO:0016491<br>GO:0008270<br>GO:0006801<br>GO:0005507<br>GO:0004785<br>GO:0004784	1e-71	82% (125/152)	EY973602<br>ES779862<br>ES780033
sb_gmnlkfta.45.C1	3	sb_gmnlkfta_0006b19.t7<br>sb_gmnlkfta_0007g09.t7<br>sb_gmnlkfta_0002e22.t7	unclassified				EX190045<br>EY973546<br>ES779903
sb_gmnlkfta.49.C1	3	sb_gmnlkfta_0004f11.t7<br>sb_gmnlkfta_0002g07.t7<br>sb_gmnlkfta_0004e11.t7	unclassified				ES780379<br>ES779931<br>ES780363
sb_gmnlkfta.50.C1	3	sb_gmnlkfta_0003k10.t7<br>sb_gmnlkfta_0002n23.t7<br>sb_gmnlkfta_0003n23.t7	Cluster: PREDICTED: similar to formin-like 1,; n=1; Danio rerio|Rep: PREDICTED: similar to formin-like 1, - Danio rerio		4e-14	40% (44/109)	ES780226<br>ES780070<br>ES780274
sb_gmnlkfta.54.C1	3	sb_gmnlkfta_0002l16.t7<br>sb_gmnlkfta_0006b12.t7<br>sb_gmnlkfta_0003c09.t7	unclassified				ES780027<br>EX190038<br>ES780126
sb_gmnlkfta.55.C1	3	sb_gmnlkfta_0006e18.t7<br>sb_gmnlkfta_0006g18.t7<br>sb_gmnlkfta_0006k16.t7	unclassified				EX190107<br>EX190150<br>EX190230
sb_gmnlkfta.69.C1	3	sb_gmnlkfta_0004o19.t7<br>sb_gmnlkfta_0006k13.t7<br>sb_gmnlkfta_0006k19.t7	unclassified				ES780537<br>EX190227<br>EX190232
sb_gmnlkfta.71.C1	3	sb_gmnlkfta_0006i14.t7<br>sb_gmnlkfta_0007e17.t7<br>sb_gmnlkfta_0007h23.t7	unclassified				EX190186<br>EY973517<br>EY973571

sb_gmnlkfta.73.C1	3	sb_gmnlkfta_0004d12.t7<br>sb_gmnlkfta_0007k23.t7<br>sb_gmnlkfta_0004c12.t7	unclassified				ES780347<br>EY973626<br>ES780331
sb_gmnlkfta.8.C1	3	sb_gmnlkfta_0002g21.t7<br>sb_gmnlkfta_0006a06.t7<br>sb_gmnlkfta_0002e05.t7	Cluster: Goose-type lysozyme 1; n=2; Gadus morhua|Rep: Goose-type lysozyme 1 - Gadus morhua (Atlantic cod)	GO:0009253<br>GO:0003796	3e-85	96% (154/160)	ES779942<br>EX190009<br>ES779889
sb_gmnlkfta.84.C1	3	sb_gmnlkfta_0004o20.t7<br>sb_gmnlkfta_0006k10.t7<br>sb_gmnlkfta_0004c05.t7	unclassified				ES780538<br>EX190225<br>ES780327
sb_gmnlkfta.86.C1	3	sb_gmnlkfta_0002b18.t7<br>sb_gmnlkfta_0006b08.t7<br>sb_gmnlkfta_0006b09.t7	Cluster: Stromal cell-derived factor 1a; n=1; Oryzias latipes|Rep: Stromal cell-derived factor 1a - Oryzias latipes (Medaka fish) (Japanese ricefish)	GO:0006955<br>GO:0005576<br>GO:0005125	7e-13	61% (33/54)	ES779841<br>EX190034<br>EX190035
sb_gmnlkfta.92.C1	3	sb_gmnlkfta_0004k23.t7<br>sb_gmnlkfta_0003i09.t7<br>sb_gmnlkfta_0006f16.t7	Cluster: Hemoglobin subunit beta-2; n=3; Gadidae|Rep: Hemoglobin subunit beta-2 - Gadus morhua (Atlantic cod)	GO:0020037<br>GO:0019825<br>GO:0015671<br>GO:0006810<br>GO:0005833<br>GO:0005506<br>GO:0005344	1e-29	100% (64/64)	ES780463<br>ES780205<br>EX190126
sb_gmnlkfta.98.C1	3	sb_gmnlkfta_0002f10.t7<br>sb_gmnlkfta_0006e20.t7<br>sb_gmnlkfta_0004n14.t7	unclassified				ES779913<br>EX190109<br>ES780516
sb_gmnlkfta.0.C1	2	sb_gmnlkfta_0006g06.t7<br>sb_gmnlkfta_0006i06.t7	Cluster: Glutathione S-transferase; n=1; Oplegnathus fasciatus|Rep: Glutathione S-transferase - Oplegnathus fasciatus	GO:0008152<br>GO:0004364	9e-26	61% (58/94)	EX190138<br>EX190180
sb_gmnlkfta.1.C2	2	sb_gmnlkfta_0002p15.t7<br>sb_gmnlkfta_0006n07.t7	Cluster: Ribosomal protein L21; n=5; Clupeocephala|Rep: Ribosomal protein L21 - Danio rerio (Zebrafish) (Brachydanio rerio)		2e-10	76% (29/38)	ES780095<br>EX190282
sb_gmnlkfta.10.C1	2	sb_gmnlkfta_0007g07.t7<br>sb_gmnlkfta_0003e09.t7	LOC100027952; similar to IKK-a kinase		1e-21	83% (47/56)	EY973544<br>ES780153

sb_gmnlkfta.100.C1	2	sb_gmnlkfta_0007i17.t7<br>sb_gmnlkfta_0004h08.t7	unclassified				EY973587<br>ES780407
sb_gmnlkfta.101.C1	2	sb_gmnlkfta_0001e01.t7<br>sb_gmnlkfta_0003l14.t7	Cluster: Heat shock protein HSP 90-alpha (HSP 86) (Renal carcinoma antigen NY- REN-38).; n=1; Canis lupus familiaris|Rep: Heat shock protein HSP 90-alpha (HSP 86) (Renal carcinoma antigen NY- REN-38). - Canis familiaris		1e-23	96% (49/51)	ES779769<br>ES780244
sb_gmnlkfta.102.C1	2	sb_gmnlkfta_0002g16.t7<br>sb_gmnlkfta_0002h06.t7	Cluster: PREDICTED: similar to Eukaryotic translation initiation factor 1 (eIF1) (Protein translation factor SUI1 homolog) (Sui1iso1) (A121); n=1; Macaca mulatta|Rep: PREDICTED: similar to Eukaryotic translation initiation factor 1 (eIF1) (Protein translation factor SUI1 homolog) (Sui1iso1) (A121) - Macaca mulatta		6e-24	85% (55/64)	ES779939<br>ES779948
sb_gmnlkfta.105.C1	2	sb_gmnlkfta_0006f17.t7<br>sb_gmnlkfta_0006h15.t7	Cluster: TATA-binding protein-associated 55 kd factor (TAF7 RNA polymerase II, TATA box binding protein (TBP)-associated factor); n=3; Danio rerio|Rep: TATA-binding protein-associated 55 kd factor (TAF7 RNA polymerase II, TATA box binding protein (TBP)-associated factor) - Danio rerio (Zebrafish) (Brachydanio rerio)		2e-13	94% (37/39)	EX190127<br>EX190167
sb_gmnlkfta.107.C1	2	sb_gmnlkfta_0004j07.t7<br>sb_gmnlkfta_0004f06.t7	unclassified				ES780433<br>ES780375
sb_gmnlkfta.108.C1	2	sb_gmnlkfta_0006e04.t7<br>sb_gmnlkfta_0001b11.t7	Cluster: Homolog of Paralichthys olivaceus "Ornithine decarboxylase antizyme large isoform.; n=1; Takifugu rubripes|Rep: Homolog of Paralichthys olivaceus "Ornithine decarboxylase antizyme large isoform. - Takifugu rubripes		6e-06	85% (23/27)	EX190094<br>ES779744
sb_gmnlkfta.111.C1	2	sb_gmnlkfta_0002i11.t7<br>sb_gmnlkfta_0006i08.t7	Cluster: PREDICTED: similar to p23; n=1; Equus caballus|Rep: PREDICTED: similar to p23 - Equus caballus		1e-33	52% (62/117)	ES779969<br>EX190182
sb_gmnlkfta.112.C1	2	sb_gmnlkfta_0002n16.t7<br>sb_gmnlkfta_0007k13.t7	unclassified				ES780064<br>EY973620
sb_gmnlkfta.113.C1	2	sb_gmnlkfta_0002d14.t7<br>sb_gmnlkfta_0007b03.t7	unclassified				ES779877<br>EY973459

sb_gmnlkfta.116.C1	2	sb_gmnlkfta_0006o11.t7<br>sb_gmnlkfta_0004g02.t7	Cluster: IgD mRNA for immunoglobulin D; n=1; Takifugu rubripes|Rep: IgD mRNA for immunoglobulin D - Takifugu rubripes		4e-17	32% (58/178)	EX190309<br>ES780389
sb_gmnlkfta.117.C1	2	sb_gmnlkfta_0002m09.t7<br>sb_gmnlkfta_0002n09.t7	unclassified				ES780041<br>ES780058
sb_gmnlkfta.118.C1	2	sb_gmnlkfta_0004k11.t7<br>sb_gmnlkfta_0004m11.t7	Cluster: Hemoglobin subunit beta-1; n=2; Gadidae|Rep: Hemoglobin subunit beta-1 - Gadus morhua (Atlantic cod)	GO:0020037<br>GO:0019825<br>GO:0015671<br>GO:0006810<br>GO:0005833<br>GO:0005506<br>GO:0005344	2e-53	98% (78/79)	ES780453<br>ES780495
sb_gmnlkfta.119.C1	2	sb_gmnlkfta_0007o18.t7<br>sb_gmnlkfta_0007c14.t7	Cluster: PREDICTED: similar to follicle stimulating hormone receptor; n=1; Ornithorhynchus anatinus|Rep: PREDICTED: similar to follicle stimulating hormone receptor - Ornithorhynchus anatinus		2e-32	92% (69/75)	EY973681<br>EY973478
sb_gmnlkfta.12.C1	2	sb_gmnlkfta_0006n20.t7<br>sb_gmnlkfta_0006b10.t7	Cluster: Complement receptor-like protein 1 precursor; n=2; Oncorhynchus mykiss|Rep: Complement receptor-like protein 1 precursor - Oncorhynchus mykiss (Rainbow trout) (Salmo gairdneri)		5e-14	44% (33/75)	EX190294<br>EX190036
sb_gmnlkfta.120.C1	2	sb_gmnlkfta_0004h17.t7<br>sb_gmnlkfta_0002h07.t7	Cluster: Splicing factor 3B subunit 5; n=9; Euteleostomi|Rep: Splicing factor 3B subunit 5 - Homo sapiens (Human)		5e-43	92% (79/85)	ES780412<br>ES779949
sb_gmnlkfta.122.C1	2	sb_gmnlkfta_0004l07.t7<br>sb_gmnlkfta_0004k07.t7	Cluster: MHC class Ia antigen; n=2; Gadus morhua|Rep: MHC class Ia antigen - Gadus morhua (Atlantic cod)	GO:0019882<br>GO:0016020<br>GO:0006955	3e-25	100% (48/48)	ES780470<br>ES780449
sb_gmnlkfta.124.C1	2	sb_gmnlkfta_0004c07.t7<br>sb_gmnlkfta_0003d05.t7	Cluster: Carbonic anhydrase; n=3; Cyprinidae|Rep: Carbonic anhydrase - Danio rerio (Zebrafish) (Brachydanio rerio)		7e-30	87% (61/70)	ES780328<br>ES780141
sb_gmnlkfta.125.C1	2	sb_gmnlkfta_0002c12.t7<br>sb_gmnlkfta_0004g24.t7	unclassified				ES779855<br>ES780402

sb_gmnlkfta.126.C1	2	sb_gmnlkfta_0004o14.t7<br>sb_gmnlkfta_0002c01.t7	unclassified				ES780532<br>ES779847
sb_gmnlkfta.127.C1	2	sb_gmnlkfta_0004e09.t7<br>sb_gmnlkfta_0004f09.t7	Cluster: MHC class Ia antigen; n=5; Gadus morhua|Rep: MHC class Ia antigen - Gadus morhua (Atlantic cod)	GO:0019882<br>GO:0016020<br>GO:0006955	1e-15	86%<br>(38/44)	ES780361<br>ES780377
sb_gmnlkfta.128.C1	2	sb_gmnlkfta_0006b24.t7<br>sb_gmnlkfta_0006j22.t7	Cluster: Cathepsin D; n=1; Gadus morhua|Rep: Cathepsin D - Gadus morhua (Atlantic cod)	GO:0004194<br>GO:0004190	2e-34	98%<br>(70/71)	EX190050<br>EX190216
sb_gmnlkfta.129.C1	2	sb_gmnlkfta_0002g24.t7<br>sb_gmnlkfta_0002n07.t7	unclassified				ES779944<br>ES780056
sb_gmnlkfta.13.C1	2	sb_gmnlkfta_0003k12.t7<br>sb_gmnlkfta_0003f15.t7	Cluster: Bloom syndrome protein homolog; n=4; Gallus gallus|Rep: Bloom syndrome protein homolog - Gallus gallus (Chicken)		8e-13	62%<br>(28/45)	ES780228<br>ES780171
sb_gmnlkfta.131.C1	2	sb_gmnlkfta_0002m14.t7<br>sb_gmnlkfta_0002l11.t7	YccV-like domain containing protein		2e-06	33%<br>(18/53)	ES780046<br>ES780022
sb_gmnlkfta.132.C1	2	sb_gmnlkfta_0006i18.t7<br>sb_gmnlkfta_0007l02.t7	unclassified				EX190190<br>EY973629
sb_gmnlkfta.133.C1	2	sb_gmnlkfta_0007a01.t7<br>sb_gmnlkfta_0006g16.t7	unclassified				EY973451<br>EX190148
sb_gmnlkfta.134.C1	2	sb_gmnlkfta_0006m13.t7<br>sb_gmnlkfta_0006a13.t7	unclassified				EX190268<br>EX190016
sb_gmnlkfta.135.C1	2	sb_gmnlkfta_0001c03.t7<br>sb_gmnlkfta_0004b10.t7	unclassified				ES779748<br>ES780313

sb_gmnlkfta.138.C1	2	sb_gmnlkfta_0001c02.t7<br>sb_gmnlkfta_0002n10.t7	unclassified					ES779747<br>ES780059
sb_gmnlkfta.139.C1	2	sb_gmnlkfta_0007c07.t7<br>sb_gmnlkfta_0007j18.t7	unclassified					EY973472<br>EY973606
sb_gmnlkfta.14.C1	2	sb_gmnlkfta_0002j05.t7<br>sb_gmnlkfta_0004j11.t7	unclassified					ES779980<br>ES780436
sb_gmnlkfta.140.C1	2	sb_gmnlkfta_0006o14.t7<br>sb_gmnlkfta_0006d22.t7	Cluster: 40S ribosomal protein S11; n=23; Euteleostomi|Rep: 40S ribosomal protein S11 - Homo sapiens (Human)		9e-24	87%<br>(51/58)		EX190312<br>EX190089
sb_gmnlkfta.141.C1	2	sb_gmnlkfta_0001f09.t7<br>sb_gmnlkfta_0001g08.t7	unclassified					ES779787<br>ES779798
sb_gmnlkfta.146.C1	2	sb_gmnlkfta_0003n05.t7<br>sb_gmnlkfta_0006h19.t7	Unassigned protein		2e-53	58%<br>(102/173)		ES780266<br>EX190171
sb_gmnlkfta.147.C1	2	sb_gmnlkfta_0002d19.t7<br>sb_gmnlkfta_0004o08.t7	Cluster: Plastin-2; n=4; Clupeocephala|Rep: Plastin-2 - Danio rerio (Zebrafish) (Brachydanio rerio)		4e-73	76%<br>(142/186)		ES779882<br>ES780528
sb_gmnlkfta.149.C1	2	sb_gmnlkfta_0006e21.t7<br>sb_gmnlkfta_0006o21.t7	Cluster: Alpha-aminoadipic semialdehyde dehydrogenase; n=13; Eutheria|Rep: Alpha-aminoadipic semialdehyde dehydrogenase - Homo sapiens (Human)		1e-83	84%<br>(139/164)		EX190110<br>EX190317
sb_gmnlkfta.15.C1	2	sb_gmnlkfta_0002j24.t7<br>sb_gmnlkfta_0006p20.t7	Cluster: Coronin-1A (Coronin-like protein p57) (Coronin-like protein A) (Clipin-A) (Tryptophan aspartate-containing coat protein) (TACO).; n=1; Takifugu rubripes|Rep: Coronin-1A (Coronin-like protein p57) (Coronin-like protein A) (Clipin-A) (Tryptophan aspartate-containing coat protein) (TACO). - Takifugu rubripes		1e-13	53%<br>(37/69)		ES779996<br>EX190337
sb_gmnlkfta.151.C1	2	sb_gmnlkfta_0006a05.t7<br>sb_gmnlkfta_0002k05.t7	unclassified					EX190008<br>ES780000

sb_gmnlkfta.152.C1	2	sb_gmnlkfta_0002j20.t7<br>sb_gmnlkfta_0003m13.t7	unclassified				ES779992<br>ES780257
sb_gmnlkfta.153.C1	2	sb_gmnlkfta_0002e20.t7<br>sb_gmnlkfta_0002a10.t7	unclassified				ES779901<br>ES779820
sb_gmnlkfta.154.C1	2	sb_gmnlkfta_0006m16.t7<br>sb_gmnlkfta_0006p18.t7	Cluster: GTP binding protein NGB; n=3; Danio rerio|Rep: GTP binding protein NGB - Danio rerio (Zebrafish) (Brachydanio rerio)		3e-48	96% (92/95)	EX190271<br>EX190335
sb_gmnlkfta.156.C1	2	sb_gmnlkfta_0002h17.t7<br>sb_gmnlkfta_0003e21.t7	unclassified				ES779956<br>ES780161
sb_gmnlkfta.158.C1	2	sb_gmnlkfta_0006f04.t7<br>sb_gmnlkfta_0007m17.t7	Cluster: Zgc:86905; n=1; Danio rerio|Rep: Zgc:86905 - Danio rerio (Zebrafish) (Brachydanio rerio)		4e-46	89% (52/58)	EX190116<br>EY973653
sb_gmnlkfta.159.C1	2	sb_gmnlkfta_0001c05.t7<br>sb_gmnlkfta_0006i03.t7	PREDICTED: similar to slit-robo rho gtpase activating protein 1,3 [Nasonia vitripennis]		1e-14	53% (42/78)	ES779750<br>EX190177
sb_gmnlkfta.161.C1	2	sb_gmnlkfta_0003d23.t7<br>sb_gmnlkfta_0006g22.t7	unclassified				ES780150<br>EX190154
sb_gmnlkfta.162.C1	2	sb_gmnlkfta_0006o12.t7<br>sb_gmnlkfta_0006c14.t7	Unassigned protein				EX190310<br>EX190062
sb_gmnlkfta.163.C1	2	sb_gmnlkfta_0003k07.t7<br>sb_gmnlkfta_0002h24.t7	unclassified				ES780223<br>ES779960
sb_gmnlkfta.164.C1	2	sb_gmnlkfta_0006e24.t7<br>sb_gmnlkfta_0004l19.t7	Cluster: PREDICTED: similar to Ribosomal protein L8 isoform 1; n=2; Eutheria|Rep: PREDICTED: similar to Ribosomal protein L8 isoform 1 - Pan troglodytes		1e-45	91% (87/95)	EX190113<br>ES780482

sb_gmnlkfta.165.C1	2	sb_gmnlkfta_0007e14.t7<br>sb_gmnlkfta_0002a01.t7	Cluster: Zgc:55347; n=2; Danio rerio|Rep: Zgc:55347 - Danio rerio (Zebrafish) (Brachydanio rerio)		7e-13	60%<br>(37/61)	EY973515<br>ES779814
sb_gmnlkfta.166.C1	2	sb_gmnlkfta_0001b01.t7<br>sb_gmnlkfta_0007m15.t7	unclassified				ES779735<br>EY973652
sb_gmnlkfta.167.C1	2	sb_gmnlkfta_0001a03.t7<br>sb_gmnlkfta_0006e19.t7	Cluster: Cathepsin L; n=1; Oryzias latipes|Rep: Cathepsin L - Oryzias latipes (Medaka fish) (Japanese ricefish)	GO:0008234<br>GO:0008233<br>GO:0006508<br>GO:0004197	9e-54	78%<br>(90/114)	ES779726<br>EX190108
sb_gmnlkfta.17.C1	2	sb_gmnlkfta_0007c24.t7<br>sb_gmnlkfta_0004n02.t7	Cluster: Ribosomal protein L32; n=4; Euteleostei|Rep: Ribosomal protein L32 - Epinephelus coioides (Orange-spotted grouper)	GO:0005840<br>GO:0005622<br>GO:0003735	9e-47	90%<br>(89/98)	EY973486<br>ES780505
sb_gmnlkfta.170.C1	2	sb_gmnlkfta_0002i19.t7<br>sb_gmnlkfta_0004d04.t7	Cluster: PREDICTED: similar to guanine nucleotide binding protein (G protein), beta polypeptide 2-like 1; n=1; Ornithorhynchus anatinus|Rep: PREDICTED: similar to guanine nucleotide binding protein (G protein), beta polypeptide 2-like 1 - Ornithorhynchus anatinus		1e-28	66%<br>(70/105)	ES779974<br>ES780340
sb_gmnlkfta.171.C1	2	sb_gmnlkfta_0004a16.t7<br>sb_gmnlkfta_0004m16.t7	LOC513641; similar to hematopoietic protein 1		4e-24	67%<br>(42/62)	ES780303<br>ES780499
sb_gmnlkfta.172.C1	2	sb_gmnlkfta_0001c07.t7<br>sb_gmnlkfta_0007g13.t7	Cluster: Tgm2b protein; n=3; Danio rerio|Rep: Tgm2b protein - Danio rerio (Zebrafish) (Brachydanio rerio)		1e-09	72%<br>(29/40)	ES779752<br>EY973548
sb_gmnlkfta.173.C1	2	sb_gmnlkfta_0002c05.t7<br>sb_gmnlkfta_0003l04.t7	unclassified				ES779850<br>ES780238
sb_gmnlkfta.174.C1	2	sb_gmnlkfta_0006h20.t7<br>sb_gmnlkfta_0006n18.t7	Cluster: Glycogen phosphorylase, liver form (EC 2.4.1.1).; n=1; Takifugu rubripes|Rep: Glycogen phosphorylase, liver form (EC 2.4.1.1). - Takifugu rubripes		3e-37	86%<br>(72/83)	EX190172<br>EX190292
sb_gmnlkfta.177.C1	2	sb_gmnlkfta_0001b03.t7<br>sb_gmnlkfta_0002h11.t7	Cluster: Zgc:85675; n=1; Danio rerio|Rep: Zgc:85675 - Danio rerio (Zebrafish) (Brachydanio rerio)		1e-84	85%<br>(152/178)	ES779737<br>ES779952

sb_gmnlkfta.178.C1	2	sb_gmnlkfta_0007i03.t7<br>sb_gmnlkfta_0007d08.t7	unclassified				EY973574<br>EY973492
sb_gmnlkfta.179.C1	2	sb_gmnlkfta_0002e19.t7<br>sb_gmnlkfta_0006h11.t7	Cluster: Cytidine deaminase; n=1; Danio rerio|Rep: Cytidine deaminase - Danio rerio (Zebrafish) (Brachydanio rerio)		7e-22	68%<br>(31/45)	ES779900<br>EX190164
sb_gmnlkfta.181.C1	2	sb_gmnlkfta_0006l14.t7<br>sb_gmnlkfta_0006n14.t7	Cluster: PREDICTED: similar to sec61-like protein; n=1; Equus caballus|Rep: PREDICTED: similar to sec61-like protein - Equus caballus		4e-22	100%<br>(52/52)	EX190249<br>EX190288
sb_gmnlkfta.184.C1	2	sb_gmnlkfta_0002e18.t7<br>sb_gmnlkfta_0003i07.t7	unclassified				ES779899<br>ES780204
sb_gmnlkfta.185.C1	2	sb_gmnlkfta_0006d08.t7<br>sb_gmnlkfta_0007g14.t7	unclassified				EX190076<br>EY973549
sb_gmnlkfta.186.C1	2	sb_gmnlkfta_0007o02.t7<br>sb_gmnlkfta_0002a18.t7	I-set domain containing protein		1e-04	32%<br>(22/67)	EY973675<br>ES779826
sb_gmnlkfta.187.C1	2	sb_gmnlkfta_0004p16.t7<br>sb_gmnlkfta_0001c12.t7	unclassified				ES780549<br>ES779757
sb_gmnlkfta.188.C1	2	sb_gmnlkfta_0002n13.t7<br>sb_gmnlkfta_0007h17.t7	unclassified				ES780061<br>EY973567
sb_gmnlkfta.189.C1	2	sb_gmnlkfta_0006c13.t7<br>sb_gmnlkfta_0006g19.t7	Cluster: Homolog of Scombridae gen "Cytochrome c oxidase subunit VIb.; n=1; Takifugu rubripes|Rep: Homolog of Scombridae gen "Cytochrome c oxidase subunit VIb. - Takifugu rubripes		4e-11	93%<br>(30/32)	EX190061<br>EX190151
sb_gmnlkfta.19.C1	2	sb_gmnlkfta_0006h07.t7<br>sb_gmnlkfta_0003o18.t7	unclassified				EX190161<br>ES780282

sb_gmnlkfta.190.C1	2	sb_gmnlkfta_0004b06.t7<br>sb_gmnlkfta_0004f24.t7	unclassified				ES780309<br>ES780387
sb_gmnlkfta.191.C1	2	sb_gmnlkfta_0006m05.t7<br>sb_gmnlkfta_0006k05.t7	Cluster: PREDICTED: similar to alpha-NAC, muscle-specific form gp220; n=1; Monodelphis domestica|Rep: PREDICTED: similar to alpha-NAC, muscle-specific form gp220 - Monodelphis domestica		6e-33	84%<br>(74/88)	EX190261<br>EX190221
sb_gmnlkfta.193.C1	2	sb_gmnlkfta_0004c11.t7<br>sb_gmnlkfta_0002g13.t7	unclassified				ES780330<br>ES779937
sb_gmnlkfta.194.C1	2	sb_gmnlkfta_0004b07.t7<br>sb_gmnlkfta_0006o08.t7	Cluster: Putative ribosomal protein L26e; n=1; Diaphorina citri|Rep: Putative ribosomal protein L26e - Diaphorina citri (Asian citrus psyllid)	GO:0015934<br>GO:0006412<br>GO:0005840<br>GO:0005622<br>GO:0003735	3e-24	86%<br>(39/45)	ES780310<br>EX190306
sb_gmnlkfta.195.C1	2	sb_gmnlkfta_0006f07.t7<br>sb_gmnlkfta_0003f08.t7	Unassigned protein				EX190119<br>ES780167
sb_gmnlkfta.196.C1	2	sb_gmnlkfta_0006m03.t7<br>sb_gmnlkfta_0006o05.t7	unclassified				EX190260<br>EX190303
sb_gmnlkfta.197.C1	2	sb_gmnlkfta_0002m05.t7<br>sb_gmnlkfta_0002o24.t7	Cluster: MGC82998 protein; n=3; Xenopus|Rep: MGC82998 protein - Xenopus laevis (African clawed frog)	GO:0016491<br>GO:0008152<br>GO:0006099<br>GO:0005739<br>GO:0004449	4e-46	88%<br>(80/90)	ES780038<br>ES780087
sb_gmnlkfta.199.C1	2	sb_gmnlkfta_0004e18.t7<br>sb_gmnlkfta_0002c18.t7	unclassified				ES780368<br>ES779859
sb_gmnlkfta.2.C1	2	sb_gmnlkfta_0003l06.t7<br>sb_gmnlkfta_0007i11.t7	unclassified				ES780239<br>EY973582
sb_gmnlkfta.200.C1	2	sb_gmnlkfta_0007f08.t7	unclassified				EY973529<br>ES779997

		sb_gmnlkfta_0002k01.t7					
sb_gmnlkfta.201.C1	2	sb_gmnlkfta_0006f22.t7 sb_gmnlkfta_0007h14.t7	Cluster: ATP synthase B chain, mitochondrial precursor (EC 3.6.3.14).; n=1; Takifugu rubripes|Rep: ATP synthase B chain, mitochondrial precursor (EC 3.6.3.14). - Takifugu rubripes		5e-12	79% (35/44)	EX190132 EY973564
sb_gmnlkfta.202.C1	2	sb_gmnlkfta_0003o09.t7 sb_gmnlkfta_0007o04.t7	unclassified				ES780278 EY973677
sb_gmnlkfta.205.C1	2	sb_gmnlkfta_0001e12.t7 sb_gmnlkfta_0004p10.t7	Cluster: Zgc:153988; n=1; Danio rerio|Rep: Zgc:153988 - Danio rerio (Zebrafish) (Brachydanio rerio)		8e-23	46% (47/102)	ES779778 ES780547
sb_gmnlkfta.206.C1	2	sb_gmnlkfta_0004l18.t7 sb_gmnlkfta_0007n12.t7	unclassified				ES780481 EY973665
sb_gmnlkfta.207.C1	2	sb_gmnlkfta_0002c10.t7 sb_gmnlkfta_0004b09.t7	unclassified				ES779853 ES780312
sb_gmnlkfta.208.C1	2	sb_gmnlkfta_0006j14.t7 sb_gmnlkfta_0006l16.t7	Cluster: Probable ribosome biogenesis protein RLP24; n=3; Danio rerio|Rep: Probable ribosome biogenesis protein RLP24 - Danio rerio (Zebrafish) (Brachydanio rerio)		5e-60	72% (95/131)	EX190209 EX190251
sb_gmnlkfta.209.C1	2	sb_gmnlkfta_0004i08.t7 sb_gmnlkfta_0001f06.t7	Cluster: 60S ribosomal protein L36; n=1; Tetraodon nigroviridis|Rep: 60S ribosomal protein L36 - Tetraodon nigroviridis (Green puffer)	GO:0006412 GO:0005840 GO:0005622 GO:0003735	1e-19	88% (44/50)	ES780422 ES779784
sb_gmnlkfta.21.C1	2	sb_gmnlkfta_0006j17.t7 sb_gmnlkfta_0006n09.t7	Cluster: Glutathione S-transferase pi; n=2; Cyprinidae|Rep: Glutathione S-transferase pi - Danio rerio (Zebrafish) (Brachydanio rerio)		4e-16	57% (42/73)	EX190212 EX190284
sb_gmnlkfta.210.C1	2	sb_gmnlkfta_0007h13.t7 sb_gmnlkfta_0007k01.t7	DUF1777 multi-domain protein		9e-09	46% (33/71)	EY973563 EY973610
sb_gmnlkfta.212.C1	2	sb_gmnlkfta_0003h11.t7 sb_gmnlkfta_0007c18.t7	unclassified				ES780193 EY973481

sb_gmnlkfta.213.C1	2	sb_gmnlkfta_0006e17.t7<br>sb_gmnlkfta_0006o09.t7	unclassified				EX190106<br>EX190307
sb_gmnlkfta.214.C1	2	sb_gmnlkfta_0001f05.t7<br>sb_gmnlkfta_0002j13.t7	Cluster: PREDICTED: similar to actin related protein 2/3 complex, subunit 4; n=1; Rattus norvegicus|Rep: PREDICTED: similar to actin related protein 2/3 complex, subunit 4 - Rattus norvegicus		7e-08	87% (28/32)	ES779783<br>ES779987
sb_gmnlkfta.215.C1	2	sb_gmnlkfta_0006e15.t7<br>sb_gmnlkfta_0006g15.t7	unclassified				EX190104<br>EX190147
sb_gmnlkfta.216.C1	2	sb_gmnlkfta_0004h14.t7<br>sb_gmnlkfta_0007g16.t7	Cluster: Novel protein similar to vertebrate SNAP-associated protein; n=1; Danio rerio|Rep: Novel protein similar to vertebrate SNAP-associated protein - Danio rerio (Zebrafish) (Brachydanio rerio)		6e-31	90% (70/77)	ES780409<br>EY973551
sb_gmnlkfta.22.C1	2	sb_gmnlkfta_0006o03.t7<br>sb_gmnlkfta_0006c24.t7	unclassified				EX190301<br>EX190071
sb_gmnlkfta.25.C1	2	sb_gmnlkfta_0004f22.t7<br>sb_gmnlkfta_0004n22.t7	unclassified				ES780386<br>ES780523
sb_gmnlkfta.27.C1	2	sb_gmnlkfta_0003h08.t7<br>sb_gmnlkfta_0003g08.t7	Cluster: Bloodthirsty; n=2; Notothenioidei|Rep: Bloodthirsty - Chaenocephalus aceratus (White crocodile fish)	GO:0008270<br>GO:0005622<br>GO:0005515	3e-21	47% (64/136)	ES780192<br>ES780179
sb_gmnlkfta.28.C1	2	sb_gmnlkfta_0006i11.t7<br>sb_gmnlkfta_0006a11.t7	Unassigned protein				EX190184<br>EX190014
sb_gmnlkfta.29.C1	2	sb_gmnlkfta_0002f05.t7<br>sb_gmnlkfta_0007h06.t7	Cluster: Beta-galactosidase; n=3; Danio rerio|Rep: Beta-galactosidase - Danio rerio (Zebrafish) (Brachydanio rerio)		6e-12	54% (28/51)	ES779910<br>EY973559
sb_gmnlkfta.3.C1	2	sb_gmnlkfta_0004b18.t7<br>sb_gmnlkfta_0002l05.t7	Cluster: 40S ribosomal protein S24; n=15; Euteleostomi|Rep: 40S ribosomal protein S24 - Oryzias latipes (Medaka fish) (Japanese	GO:0006412<br>GO:0005840<br>GO:0005622	2e-35	98% (57/58)	ES780319<br>ES780019

			ricefish)	GO:0003735<br>GO:0000166			
sb_gmnlkfta.30.C1	2	sb_gmnlkfta_0002e04.t7<br>sb_gmnlkfta_0003i15.t7	unclassified				ES779888<br>ES780208
sb_gmnlkfta.31.C1	2	sb_gmnlkfta_0006f11.t7<br>sb_gmnlkfta_0006e23.t7	Cluster: Replication protein A 70 kDa DNA-binding subunit; n=4; Danio rerio|Rep: Replication protein A 70 kDa DNA-binding subunit - Danio rerio (Zebrafish) (Brachydanio rerio)		2e-16	79%<br>(39/49)	EX190121<br>EX190112
sb_gmnlkfta.32.C1	2	sb_gmnlkfta_0004g20.t7<br>sb_gmnlkfta_0007k15.t7	DUF758 domain containing protein		5e-06	72%<br>(18/25)	ES780399<br>EY973622
sb_gmnlkfta.34.C1	2	sb_gmnlkfta_0006h03.t7<br>sb_gmnlkfta_0007d15.t7	Cluster: PREDICTED: similar to alpha-cardiac actin; n=1; Ornithorhynchus anatinus|Rep: PREDICTED: similar to alpha-cardiac actin - Ornithorhynchus anatinus		2e-08	90%<br>(27/30)	EX190157<br>EY973497
sb_gmnlkfta.35.C1	2	sb_gmnlkfta_0006k17.t7<br>sb_gmnlkfta_0006m15.t7	caspc; caspase c		3e-09	54%<br>(20/37)	EX190231<br>EX190270
sb_gmnlkfta.36.C1	2	sb_gmnlkfta_0004n09.t7<br>sb_gmnlkfta_0004m10.t7	unclassified				ES780511<br>ES780494
sb_gmnlkfta.38.C1	2	sb_gmnlkfta_0002f19.t7<br>sb_gmnlkfta_0004k14.t7	Zona_pellucida domain containing protein		1e-04	27%<br>(15/55)	ES779922<br>ES780456
sb_gmnlkfta.39.C1	2	sb_gmnlkfta_0003j06.t7<br>sb_gmnlkfta_0003h06.t7	unclassified				ES780215<br>ES780190
sb_gmnlkfta.4.C1	2	sb_gmnlkfta_0006a08.t7<br>sb_gmnlkfta_0006c08.t7	unclassified				EX190011<br>EX190057
sb_gmnlkfta.40.C1	2	sb_gmnlkfta_0007i06.t7<br>sb_gmnlkfta_0001e05.t7	Cluster: 60S ribosomal protein L22; n=1; Gadus morhua|Rep: 60S ribosomal protein L22 - Gadus morhua (Atlantic cod)	GO:0006412<br>GO:0005840<br>GO:0005622	2e-23	96%<br>(54/56)	EY973577<br>ES779773

				GO:0003735<br>GO:0003723			
sb_gmnlkfta.42.C1	2	sb_gmnlkfta_0006g04.t7<br>sb_gmnlkfta_0006k22.t7	Cluster: Homolog of Carassius auratus "Ig heavy chain V region 5A precursor.; n=4; Takifugu rubripes|Rep: Homolog of Carassius auratus "Ig heavy chain V region 5A precursor. - Takifugu rubripes		1e-25	80%<br>(38/47)	EX190136<br>EX190235
sb_gmnlkfta.43.C1	2	sb_gmnlkfta_0007f13.t7<br>sb_gmnlkfta_0007f19.t7	unclassified				EY973532<br>EY973536
sb_gmnlkfta.44.C1	2	sb_gmnlkfta_0002d18.t7<br>sb_gmnlkfta_0002l21.t7	unclassified				ES779881<br>ES780030
sb_gmnlkfta.5.C1	2	sb_gmnlkfta_0006d15.t7<br>sb_gmnlkfta_0006j11.t7	Cluster: TCP1-theta; n=1; Notothenia coriiceps|Rep: TCP1-theta - Notothenia coriiceps (yellowbelly rockcod)	GO:0044267<br>GO:0006457<br>GO:0005737<br>GO:0005524<br>GO:0005515<br>GO:0000166	1e-25	90%<br>(56/62)	EX190083<br>EX190206
sb_gmnlkfta.52.C1	2	sb_gmnlkfta_0006n22.t7<br>sb_gmnlkfta_0002o06.t7	unclassified				EX190296<br>ES780074
sb_gmnlkfta.53.C1	2	sb_gmnlkfta_0004a21.t7<br>sb_gmnlkfta_0003n12.t7	unclassified				ES780306<br>ES780269
sb_gmnlkfta.56.C1	2	sb_gmnlkfta_0002i15.t7<br>sb_gmnlkfta_0007n22.t7	unclassified				ES779971<br>EY973674
sb_gmnlkfta.57.C1	2	sb_gmnlkfta_0004j03.t7<br>sb_gmnlkfta_0001f12.t7	unclassified				ES780429<br>ES779790
sb_gmnlkfta.58.C1	2	sb_gmnlkfta_0007k17.t7<br>sb_gmnlkfta_0002h14.t7	unclassified				EY973623<br>ES779953

sb_gmnlkfta.59.C1	2	sb_gmnlkfta_0001g01.t7<br>sb_gmnlkfta_0007c11.t7	unclassified					ES779791<br>EY973476
sb_gmnlkfta.6.C1	2	sb_gmnlkfta_0004d16.t7<br>sb_gmnlkfta_0002k22.t7	Unassigned protein					ES780350<br>ES780014
sb_gmnlkfta.60.C1	2	sb_gmnlkfta_0004l06.t7<br>sb_gmnlkfta_0004p20.t7	unclassified					ES780469<br>ES780552
sb_gmnlkfta.63.C1	2	sb_gmnlkfta_0001d12.t7<br>sb_gmnlkfta_0002i07.t7	Cluster: Solute carrier family 35 member B1; n=1; Danio rerio|Rep: Solute carrier family 35 member B1 - Danio rerio (Zebrafish) (Brachydanio rerio)		4e-20	91%<br>(43/47)		ES779768<br>ES779967
sb_gmnlkfta.66.C1	2	sb_gmnlkfta_0006e07.t7<br>sb_gmnlkfta_0002m11.t7	Cluster: 60S ribosomal protein L7a; n=51; Euteleostomi|Rep: 60S ribosomal protein L7a - Mus musculus (Mouse)		5e-21	93%<br>(45/48)		EX190097<br>ES780043
sb_gmnlkfta.68.C1	2	sb_gmnlkfta_0002f12.t7<br>sb_gmnlkfta_0002k20.t7	unclassified					ES779915<br>ES780012
sb_gmnlkfta.70.C1	2	sb_gmnlkfta_0002k24.t7<br>sb_gmnlkfta_0002p22.t7	unclassified					ES780016<br>ES780101
sb_gmnlkfta.72.C1	2	sb_gmnlkfta_0002h04.t7<br>sb_gmnlkfta_0002b01.t7	unclassified					ES779946<br>ES779831
sb_gmnlkfta.74.C1	2	sb_gmnlkfta_0006c21.t7<br>sb_gmnlkfta_0006k21.t7	Cluster: PREDICTED: similar to ribosomal protein S17; n=2; Laurasiatheria|Rep: PREDICTED: similar to ribosomal protein S17 - Equus caballus		4e-32	95%<br>(68/71)		EX190068<br>EX190234
sb_gmnlkfta.75.C1	2	sb_gmnlkfta_0006h02.t7<br>sb_gmnlkfta_0006d23.t7	unclassified					EX190156<br>EX190090

sb_gmnlkfta.77.C1	2	sb_gmnlkfta_0007c21.t7<br>sb_gmnlkfta_0007g15.t7	unclassified				EY973484<br>EY973550
sb_gmnlkfta.79.C1	2	sb_gmnlkfta_0006c06.t7<br>sb_gmnlkfta_0006e06.t7	Cluster: NADH dehydrogenase [ubiquinone] 1 beta subcomplex subunit 8, mitochondrial precursor; n=4; Laurasiatheria|Rep: NADH dehydrogenase [ubiquinone] 1 beta subcomplex subunit 8, mitochondrial precursor - Bos taurus (Bovine)		4e-62	66%<br>(107/160)	EX190055<br>EX190096
sb_gmnlkfta.80.C1	2	sb_gmnlkfta_0006i15.t7<br>sb_gmnlkfta_0006o13.t7	Cluster: Eukaryotic elongation factor 1 alpha; n=1; Gadus morhua|Rep: Eukaryotic elongation factor 1 alpha - Gadus morhua (Atlantic cod)		8e-48	97%<br>(93/95)	EX190187<br>EX190311
sb_gmnlkfta.81.C1	2	sb_gmnlkfta_0004i16.t7<br>sb_gmnlkfta_0002k15.t7	Cluster: PREDICTED: similar to Actin, cytoplasmic 2 (Gamma-actin); n=1; Rattus norvegicus|Rep: PREDICTED: similar to Actin, cytoplasmic 2 (Gamma-actin) - Rattus norvegicus		5e-25	96%<br>(55/57)	ES780425<br>ES780007
sb_gmnlkfta.83.C1	2	sb_gmnlkfta_0004d13.t7<br>sb_gmnlkfta_0003p10.t7	Cluster: Ferritin heavy subunit; n=3; Clupeocephala|Rep: Ferritin heavy subunit - Ictalurus punctatus (Channel catfish)	GO:0046872<br>GO:0016491<br>GO:0008199<br>GO:0006879<br>GO:0006826<br>GO:0005506<br>GO:0005488	6e-69	89%<br>(73/82)	ES780348<br>ES780288
sb_gmnlkfta.87.C1	2	sb_gmnlkfta_0007e07.t7<br>sb_gmnlkfta_0002k23.t7	unclassified				EY973510<br>ES780015
sb_gmnlkfta.89.C1	2	sb_gmnlkfta_0007f14.t7<br>sb_gmnlkfta_0002o23.t7	Cluster: Affixin; n=3; Danio rerio|Rep: Affixin - Danio rerio (Zebrafish) (Brachydanio rerio)		3e-58	89%<br>(115/129)	EY973533<br>ES780086
sb_gmnlkfta.9.C1	2	sb_gmnlkfta_0002j10.t7<br>sb_gmnlkfta_0003d12.t7	unclassified				ES779984<br>ES780145
sb_gmnlkfta.90.C1	2	sb_gmnlkfta_0002d16.t7<br>sb_gmnlkfta_0002l14.t7	unclassified				ES779879<br>ES780025

sb_gmnlkfta.91.C1	2	sb_gmnlkfta_0003n09.t7<br>sb_gmnlkfta_0006c04.t7	Cluster: CC chemokine type 2; n=1; Gadus morhua|Rep: CC chemokine type 2 - Gadus morhua (Atlantic cod)		6e-14	63% (38/60)	ES780268<br>EX190053
sb_gmnlkfta.93.C1	2	sb_gmnlkfta_0004i15.t7<br>sb_gmnlkfta_0002p17.t7	Cluster: Zgc:91957; n=1; Danio rerio|Rep: Zgc:91957 - Danio rerio (Zebrafish) (Brachydanio rerio)		2e-76	83% (139/166)	ES780424<br>ES780097
sb_gmnlkfta.95.C1	2	sb_gmnlkfta_0001g04.t7<br>sb_gmnlkfta_0002n21.t7	unclassified				ES779794<br>ES780068
sb_gmnlkfta.96.C1	2	sb_gmnlkfta_0004m20.t7<br>sb_gmnlkfta_0007c22.t7	Cluster: Wu:fc26c03 protein; n=4; Danio rerio|Rep: Wu:fc26c03 protein - Danio rerio (Zebrafish) (Brachydanio rerio)		6e-18	94% (32/34)	ES780501<br>EY973485
sb_gmnlkfta.97.C1	2	sb_gmnlkfta_0004j06.t7<br>sb_gmnlkfta_0007e05.t7	Cluster: FK506 binding protein 4; n=3; Danio rerio|Rep: FK506 binding protein 4 - Danio rerio (Zebrafish) (Brachydanio rerio)		2e-56	83% (108/130)	ES780432<br>EY973508
sb_gmnlkfta.99.C1	2	sb_gmnlkfta_0001h08.t7<br>sb_gmnlkfta_0006e05.t7	unclassified				ES779809<br>EX190095
sb_gmnlkfta_0001a01.t7	1		unclassified				ES779724
sb_gmnlkfta_0001a02.t7	1		Cluster: Hemoglobin subunit beta-2; n=3; Gadidae|Rep: Hemoglobin subunit beta-2 - Arctogadus glacialis (Arctic cod)		2e-12	100% (22/22)	ES779725
sb_gmnlkfta_0001a04.t7	1		unclassified				ES779727
sb_gmnlkfta_0001a05.t7	1		Cluster: Cytochrome b-245, beta polypeptide; n=2; Danio rerio|Rep: Cytochrome b-245, beta polypeptide - Brachydanio rerio (Zebrafish) (Danio rerio)		9e-29	83% (57/68)	ES779728
sb_gmnlkfta_0001a06.t7	1		Cluster: PREDICTED: similar to Ribosomal protein L8 isoform 1; n=2; Eutheria|Rep: PREDICTED: similar to Ribosomal protein L8		2e-25	100%	ES779729

			isoform 1 - Pan troglodytes			(53/53)	
sb_gmnlkfta_0001a08.t7	1		unclassified				ES779730
sb_gmnlkfta_0001a09.t7	1		Cluster: Elongation factor-1 alpha; n=57; Euteleostomi|Rep: Elongation factor-1 alpha - Leptodoras sp. 1-GM-2003		1e-75	86% (137/159)	ES779731
sb_gmnlkfta_0001a10.t7	1		Cluster: Homolog of Gallus gallus "Inner centromere protein.; n=1; Takifugu rubripes|Rep: Homolog of Gallus gallus "Inner centromere protein. - Takifugu rubripes		1e-26	55% (63/114)	ES779732
sb_gmnlkfta_0001a11.t7	1		unclassified				ES779733
sb_gmnlkfta_0001a12.t7	1		unclassified				ES779734
sb_gmnlkfta_0001b02.t7	1		Cluster: CC chemokine type 3; n=1; Gadus morhua|Rep: CC chemokine type 3 - Gadus morhua (Atlantic cod)		1e-19	64% (49/76)	ES779736
sb_gmnlkfta_0001b04.t7	1		unclassified				ES779738
sb_gmnlkfta_0001b05.t7	1		unclassified				ES779739
sb_gmnlkfta_0001b06.t7	1		Cluster: MGC84123 protein; n=2; Xenopus|Rep: MGC84123 protein - Xenopus laevis (African clawed frog)	GO:0004175 GO:0004298 GO:0005737 GO:0005829 GO:0005839 GO:0006511 GO:0008233 GO:0016787 GO:0043234	3e-43	91% (85/93)	ES779740
sb_gmnlkfta_0001b07.t7	1		LSU rRNA; Hydrolagus colliei		1e-34	98% (74/75)	

sb_gmnlkfta_0001b08.t7	1		Cluster: Homolog of Gallus gallus "Ig lambda chain C region.; n=5; Takifugu rubripes|Rep: Homolog of Gallus gallus "Ig lambda chain C region. - Takifugu rubripes		2e-14	53% (37/69)	ES779741
sb_gmnlkfta_0001b09.t7	1		Cluster: RH30-like protein; n=1; Takifugu rubripes|Rep: RH30-like protein - Fugu rubripes (Japanese pufferfish) (Takifugu rubripes)	GO:0016020 GO:0016021	1e-27	61% (52/84)	ES779742
sb_gmnlkfta_0001b10.t7	1		unclassified				ES779743
sb_gmnlkfta_0001b12.t7	1		Cluster: Ribosomal protein L5a; n=4; Otophysi|Rep: Ribosomal protein L5a - Brachydanio rerio (Zebrafish) (Danio rerio)		2e-08	93% (28/30)	ES779745
sb_gmnlkfta_0001c01.t7	1		unclassified				ES779746
sb_gmnlkfta_0001c06.t7	1		LOC683250; similar to Eukaryotic translation initiation factor 4E type 3 (eIF4E type 3) (eIF-4E type 3) (mRNA cap-binding protein type 3) (Eukaryotic translation initiation factor 4E-like 3) (Eukaryotic translation initiation factor 4E homologous protein) (mR...		1e-64	89% (115/129)	ES779751
sb_gmnlkfta_0001c09.t7	1		LOC567493; similar to Peroxisomal acyl-coenzyme A thioester hydrolase 1 (Peroxisomal long-chain acyl-coA thioesterase 1) (Peroxisomal acyl-CoA thioesterase 2) (PTE-2)		6e-51	70% (90/128)	ES779754
sb_gmnlkfta_0001c10.t7	1		unclassified				ES779755
sb_gmnlkfta_0001c11.t7	1		Cluster: PREDICTED: decapping enzyme hDcp2; n=3; Mus musculus|Rep: PREDICTED: decapping enzyme hDcp2 - Mus musculus		9e-09	81% (27/33)	ES779756
sb_gmnlkfta_0001d01.t7	1		unclassified				ES779758
sb_gmnlkfta_0001d02.t7	1		unclassified				ES779759

sb_gmnlkfta_0001d03.t7	1		unclassified				ES779760
sb_gmnlkfta_0001d04.t7	1		unclassified				ES779761
sb_gmnlkfta_0001d05.t7	1		unclassified				ES779762
sb_gmnlkfta_0001d07.t7	1		Cluster: Heat shock protein 90 beta; n=7; Euteleostomi|Rep: Heat shock protein 90 beta - Paralichthys olivaceus (Japanese flounder)		3e-34	78% (75/95)	ES779763
sb_gmnlkfta_0001d08.t7	1		unclassified				ES779764
sb_gmnlkfta_0001d09.t7	1		Cluster: Ribosomal protein L3; n=8; Euteleostomi|Rep: Ribosomal protein L3 - Brachydanio rerio (Zebrafish) (Danio rerio)	GO:0003735 GO:0005622 GO:0005840 GO:0006412 GO:0030529	3e-45	90% (89/98)	ES779765
sb_gmnlkfta_0001d10.t7	1		unclassified				ES779766
sb_gmnlkfta_0001d11.t7	1		unclassified				ES779767
sb_gmnlkfta_0001e02.t7	1		unclassified				ES779770
sb_gmnlkfta_0001e03.t7	1		unclassified				ES779771
sb_gmnlkfta_0001e04.t7	1		unclassified				ES779772

sb_gmnlkfta_0001e07.t7	1		unclassified				ES779774
sb_gmnlkfta_0001e08.t7	1		Cluster: PREDICTED: similar to glutathione S-transferase M isoform 1; n=2; Danio rerio|Rep: PREDICTED: similar to glutathione S-transferase M isoform 1 - Danio rerio		5e-73	77% (124/160)	ES779775
sb_gmnlkfta_0001e11.t7	1		unclassified				ES779777
sb_gmnlkfta_0001f01.t7	1		unclassified				ES779779
sb_gmnlkfta_0001f02.t7	1		unclassified				ES779780
sb_gmnlkfta_0001f03.t7	1		Unassigned protein				ES779781
sb_gmnlkfta_0001f07.t7	1		unclassified				ES779785
sb_gmnlkfta_0001f08.t7	1		unclassified				ES779786
sb_gmnlkfta_0001f10.t7	1		unclassified				ES779788
sb_gmnlkfta_0001f11.t7	1		unclassified				ES779789
sb_gmnlkfta_0001g02.t7	1		Cluster: CC chemokine type 3; n=1; Gadus morhua|Rep: CC chemokine type 3 - Gadus morhua (Atlantic cod)	GO:0005576 GO:0006955 GO:0008009	6e-29	84% (62/73)	ES779792

sb_gmnlkfta_0001g03.t7	1		unclassified				ES779793
sb_gmnlkfta_0001g05.t7	1		Cluster: PREDICTED: similar to Electron-transfer-flavoprotein, alpha polypeptide, partial; n=1; Danio rerio|Rep: PREDICTED: similar to Electron-transfer-flavoprotein, alpha polypeptide, partial - Danio rerio		6e-06	47% (24/51)	ES779795
sb_gmnlkfta_0001g06.t7	1		unclassified				ES779796
sb_gmnlkfta_0001g07.t7	1		Cluster: 14-3-3 protein epsilon; n=19; Euteleostomi|Rep: 14-3-3 protein epsilon - Homo sapiens (Human)		2e-49	76% (101/132)	ES779797
sb_gmnlkfta_0001g09.t7	1		Cluster: Homolog of Oncorhynchus mykiss "Complement factor B/C2-B.; n=1; Takifugu rubripes|Rep: Homolog of Oncorhynchus mykiss "Complement factor B/C2-B. - Takifugu rubripes		4e-11	51% (30/58)	ES779799
sb_gmnlkfta_0001g10.t7	1		Cluster: Homolog of Homo sapiens "GDP-mannose pyrophosphorylase A; n=1; Takifugu rubripes|Rep: Homolog of Homo sapiens "GDP-mannose pyrophosphorylase A - Takifugu rubripes		7e-69	90% (127/140)	ES779800
sb_gmnlkfta_0001g11.t7	1		unclassified				ES779801
sb_gmnlkfta_0001g12.t7	1		unclassified				ES779802
sb_gmnlkfta_0001h02.t7	1		Cluster: Homolog of Homo sapiens "Kunitz-type protease inhibitor 2 precursor; n=1; Takifugu rubripes|Rep: Homolog of Homo sapiens "Kunitz-type protease inhibitor 2 precursor - Takifugu rubripes		1e-25	47% (56/118)	ES779803
sb_gmnlkfta_0001h03.t7	1		unclassified				ES779804
sb_gmnlkfta_0001h04.t7	1		unclassified				ES779805

sb_gmnlkfta_0001h07.t7	1		Cluster: Metaxin 2; n=2; Takifugu rubripes|Rep: Metaxin 2 - Fugu rubripes (Japanese pufferfish) (Takifugu rubripes)		2e-27	91% (52/57)	ES779808
sb_gmnlkfta_0001h09.t7	1		unclassified				ES779810
sb_gmnlkfta_0001h11.t7	1		unclassified				ES779812
sb_gmnlkfta_0001h12.t7	1		unclassified				ES779813
sb_gmnlkfta_0002a05.t7	1		unclassified				ES779817
sb_gmnlkfta_0002a06.t7	1		unclassified				ES779818
sb_gmnlkfta_0002a12.t7	1		Unassigned protein				ES779821
sb_gmnlkfta_0002a14.t7	1		unclassified				ES779822
sb_gmnlkfta_0002a16.t7	1		unclassified				ES779824
sb_gmnlkfta_0002a17.t7	1		unclassified				ES779825
sb_gmnlkfta_0002a19.t7	1		unclassified				ES779827
sb_gmnlkfta_0002a20.t7	1		LOC573882; similar to 5-lipoxygenase activating protein (FLAP)		1e-24	63%	ES779828

			(MK-886-binding protein)			(52/82)	
sb_gmnlkfta_0002a23.t7	1		unclassified				ES779830
sb_gmnlkfta_0002b05.t7	1		unclassified				ES779832
sb_gmnlkfta_0002b08.t7	1		Cluster: Dolichyl-diphosphooligosaccharide-protein glycosyltransferase; n=2; Danio rerio|Rep: Dolichyl-diphosphooligosaccharide-protein glycosyltransferase - Brachydanio rerio (Zebrafish) (Danio rerio)		1e-68	91% (123/134)	ES779833
sb_gmnlkfta_0002b10.t7	1		unclassified				ES779834
sb_gmnlkfta_0002b11.t7	1		unclassified				ES779835
sb_gmnlkfta_0002b12.t7	1		unclassified				ES779836
sb_gmnlkfta_0002b13.t7	1		Cluster: Krt18 protein; n=3; Danio rerio|Rep: Krt18 protein - Brachydanio rerio (Zebrafish) (Danio rerio)		2e-10	100% (20/20)	ES779837
sb_gmnlkfta_0002b14.t7	1		unclassified				ES779838
sb_gmnlkfta_0002b15.t7	1		unclassified				ES779839
sb_gmnlkfta_0002b17.t7	1		Cluster: 26S proteasome non-ATPase regulatory subunit 7; n=2; Sophophora|Rep: 26S proteasome non-ATPase regulatory subunit 7 - Drosophila melanogaster (Fruit fly)		6e-30	92% (46/50)	ES779840
sb_gmnlkfta_0002b19.t7	1		unclassified				ES779842

sb_gmnlkfta_0002b21.t7	1		Cluster: Ribosomal protein L18; n=3; Euteleostomi|Rep: Ribosomal protein L18 - Pagrus major (Red sea bream) (Chrysophrys major)	GO:0003735<br>GO:0005622<br>GO:0005840<br>GO:0006412<br>GO:0030529	6e-50	84% (96/113)	ES779844
sb_gmnlkfta_0002b22.t7	1		unclassified				ES779845
sb_gmnlkfta_0002b23.t7	1		Cluster: Proteasome subunit alpha type 2; n=32; Euteleostomi|Rep: Proteasome subunit alpha type 2 - Homo sapiens (Human)	GO:0004175<br>GO:0004298<br>GO:0005829<br>GO:0005839<br>GO:0006511<br>GO:0008233<br>GO:0016787<br>GO:0043234	9e-17	97% (39/40)	ES779846
sb_gmnlkfta_0002c03.t7	1		unclassified				ES779848
sb_gmnlkfta_0002c04.t7	1		Cluster: Ribosomal protein L10; n=7; Euteleostomi|Rep: Ribosomal protein L10 - Brachydanio rerio (Zebrafish) (Danio rerio)		2e-27	98% (54/55)	ES779849
sb_gmnlkfta_0002c08.t7	1		unclassified				ES779851
sb_gmnlkfta_0002c09.t7	1		unclassified				ES779852
sb_gmnlkfta_0002c11.t7	1		ZWILCH, LOC415552; Zwilch, kinetochore associated, homolog (Drosophila)		8e-39	50% (73/146)	ES779854
sb_gmnlkfta_0002c13.t7	1		unclassified				ES779856
sb_gmnlkfta_0002c14.t7	1		Cluster: Actin related protein 2/3 complex, subunit 5B; n=2; Clupeocephala|Rep: Actin related protein 2/3 complex, subunit 5B - Brachydanio rerio (Zebrafish) (Danio rerio)		2e-16	100% (42/42)	ES779857

sb_gmnlkfta_0002c17.t7	1		unclassified				ES779858
sb_gmnlkfta_0002c19.t7	1		unclassified				ES779860
sb_gmnlkfta_0002c20.t7	1		unclassified				ES779861
sb_gmnlkfta_0002c22.t7	1		Cluster: UPI0000D8D10D related cluster; n=1; Danio rerio|Rep: UPI0000D8D10D UniRef100 entry - Danio rerio		2e-12	57% (36/63)	ES779863
sb_gmnlkfta_0002c23.t7	1		unclassified				ES779864
sb_gmnlkfta_0002c24.t7	1		unclassified				ES779865
sb_gmnlkfta_0002d01.t7	1		unclassified				ES779866
sb_gmnlkfta_0002d03.t7	1		Cluster: Zgc:77429 protein; n=1; Danio rerio|Rep: Zgc:77429 protein - Brachydanio rerio (Zebrafish) (Danio rerio)		9e-27	67% (53/79)	ES779867
sb_gmnlkfta_0002d04.t7	1		unclassified				ES779868
sb_gmnlkfta_0002d05.t7	1		Cluster: Homolog of Homo sapiens "Feline leukemia virus subgroup C receptor-related protein 2; n=1; Takifugu rubripes|Rep: Homolog of Homo sapiens "Feline leukemia virus subgroup C receptor-related protein 2 - Takifugu rubripes		4e-38	80% (81/101)	ES779869
sb_gmnlkfta_0002d06.t7	1		Cluster: PREDICTED: similar to 40S ribosomal protein S2; n=1; Gallus gallus|Rep: PREDICTED: similar to 40S ribosomal protein S2 - Gallus gallus		2e-25	96% (55/57)	ES779870

sb_gmnlkfta_0002d07.t7	1		unclassified				ES779871
sb_gmnlkfta_0002d08.t7	1		Cluster: 60S ribosomal protein L27; n=11; Euteleostomi|Rep: 60S ribosomal protein L27 - Brachydanio rerio (Zebrafish) (Danio rerio)		8e-10	100% (31/31)	ES779872
sb_gmnlkfta_0002d10.t7	1		unclassified				ES779874
sb_gmnlkfta_0002d11.t7	1		Cluster: PREDICTED: similar to ribosomal protein L39; n=1; Bos taurus|Rep: PREDICTED: similar to ribosomal protein L39 - Bos taurus		2e-13	97% (35/36)	ES779875
sb_gmnlkfta_0002d12.t7	1		Unassigned protein				ES779876
sb_gmnlkfta_0002d15.t7	1		unclassified				ES779878
sb_gmnlkfta_0002d17.t7	1		unclassified				ES779880
sb_gmnlkfta_0002d20.t7	1		mobkl1a; MOB1, Mps One Binder kinase activator-like 1A (yeast) [KO:K06685]		4e-07	100% (25/25)	ES779883
sb_gmnlkfta_0002d21.t7	1		unclassified				ES779884
sb_gmnlkfta_0002d23.t7	1		unclassified				ES779885
sb_gmnlkfta_0002e01.t7	1		unclassified				ES779886

sb_gmnlkfta_0002e02.t7	1		Cluster: Homolog of Homo sapiens "WD-repeat protein 5; n=5; Euteleostomi|Rep: Homolog of Homo sapiens "WD-repeat protein 5 - Takifugu rubripes		2e-24	89% (42/47)	ES779887
sb_gmnlkfta_0002e06.t7	1		Cluster: Heat shock 70kDa protein 8; n=1; Danio rerio|Rep: Heat shock 70kDa protein 8 - Brachydanio rerio (Zebrafish) (Danio rerio)		4e-08	90% (28/31)	ES779890
sb_gmnlkfta_0002e08.t7	1		unclassified				ES779892
sb_gmnlkfta_0002e10.t7	1		unclassified				ES779893
sb_gmnlkfta_0002e11.t7	1		unclassified				ES779894
sb_gmnlkfta_0002e12.t7	1		unclassified				ES779895
sb_gmnlkfta_0002e13.t7	1		unclassified				ES779896
sb_gmnlkfta_0002e16.t7	1		unclassified				ES779898
sb_gmnlkfta_0002e21.t7	1		unclassified				ES779902
sb_gmnlkfta_0002e23.t7	1		unclassified				ES779904
sb_gmnlkfta_0002e24.t7	1		Cluster: Hemoglobin subunit alpha-1; n=3; Gadidae|Rep: Hemoglobin subunit alpha-1 - Arctogadus glacialis (Arctic cod)	GO:0005344 GO:0005506 GO:0005833 GO:0006810 GO:0015671 GO:0019825 GO:0020037	2e-11	94% (18/19)	ES779905

				GO:0046872			
sb_gmnlkfta_0002f02.t7	1		Cluster: Homolog of Homo sapiens "Elongation of very long chain fatty acids protein 1; n=1; Takifugu rubripes|Rep: Homolog of Homo sapiens "Elongation of very long chain fatty acids protein 1 - Takifugu rubripes		3e-23	79% (53/67)	ES779907
sb_gmnlkfta_0002f03.t7	1		unclassified				ES779908
sb_gmnlkfta_0002f04.t7	1		Cluster: CXC chemokine receptor; n=1; Oncorhynchus mykiss|Rep: CXC chemokine receptor - Oncorhynchus mykiss (Rainbow trout) (Salmo gairdneri)	GO:0001584 GO:0004871 GO:0004872 GO:0004930 GO:0007165 GO:0007186 GO:0016021 GO:0016493 GO:0016494	4e-13	45% (38/83)	ES779909
sb_gmnlkfta_0002f06.t7	1		Cluster: DUTP pyrophosphatase; n=2; Danio rerio|Rep: DUTP pyrophosphatase - Brachydanio rerio (Zebrafish) (Danio rerio)		2e-36	82% (55/67)	ES779911
sb_gmnlkfta_0002f07.t7	1		LSU rRNA; Hydrolagus colliei		1e-175	98% (325/331)	EX189995
sb_gmnlkfta_0002f11.t7	1		unclassified				ES779914
sb_gmnlkfta_0002f13.t7	1		unclassified				ES779916
sb_gmnlkfta_0002f14.t7	1		putative protein involved in 60S ribosome subunit biogenesis.		2e-31	67% (36/53)	ES779917
sb_gmnlkfta_0002f15.t7	1		unclassified				ES779918
sb_gmnlkfta_0002f17.t7	1		unclassified				ES779920

sb_gmnlkfta_0002f21.t7	1		LSU rRNA; Petromyzon marinus		6e-58	99% (113/114)	ES779924
sb_gmnlkfta_0002f23.t7	1		unclassified				ES779925
sb_gmnlkfta_0002g01.t7	1		unclassified				ES779927
sb_gmnlkfta_0002g02.t7	1		unclassified				ES779928
sb_gmnlkfta_0002g03.t7	1		unclassified				ES779929
sb_gmnlkfta_0002g06.t7	1		Unassigned protein				ES779930
sb_gmnlkfta_0002g09.t7	1		Cluster: DUTP pyrophosphatase; n=2; Danio rerio|Rep: DUTP pyrophosphatase - Brachydanio rerio (Zebrafish) (Danio rerio)		6e-32	90% (30/33)	ES779933
sb_gmnlkfta_0002g10.t7	1		unclassified				ES779934
sb_gmnlkfta_0002g11.t7	1		unclassified				ES779935
sb_gmnlkfta_0002g15.t7	1		Cluster: PREDICTED: similar to Proteasome subunit alpha type 6 (Proteasome iota chain) (Macropain iota chain) (Multicatalytic endopeptidase complex iota chain) isoform 4; n=3; Eutheria|Rep: PREDICTED: similar to Proteasome subunit alpha type 6 (Proteasome iota chain) (Macropain iota chain) (Multicatalytic endopeptidase complex iota chain) isoform 4 - Canis familiaris		9e-12	91% (31/34)	ES779938
sb_gmnlkfta_0002g19.t7	1		Cluster: Homolog of Homo sapiens "Pre-mRNA cleavage comPlex II Protein Pcf11; n=1; Takifugu rubripes|Rep: Homolog of Homo sapiens "Pre-mRNA cleavage comPlex II Protein Pcf11 - Takifugu rubripes		4e-14	92% (39/42)	ES779941

sb_gmnlkfta_0002g23.t7	1		unclassified				ES779943
sb_gmnlkfta_0002h01.t7	1		unclassified				ES779945
sb_gmnlkfta_0002h05.t7	1		Cluster: Proteasome subunit alpha type 1 (EC 3.4.25.1) (Proteasome component C2) (Macropain subunit C2) (Multicatalytic endopeptidase complex subunit C2) (Proteasome nu chain) (30 kDa prosomal protein) (PROS-30).; n=1; Xenopus tropicalis|Rep: Proteasome subunit alpha type 1 (EC 3.4.25.1) (Proteasome component C2) (Macropain subunit C2) (Multicatalytic endopeptidase complex subunit C2) (Proteasome nu chain) (30 kDa prosomal protein) (PROS-30). - Xenopus tropicalis		3e-40	88% (69/78)	ES779947
sb_gmnlkfta_0002h08.t7	1		unclassified				ES779950
sb_gmnlkfta_0002h10.t7	1		unclassified				ES779951
sb_gmnlkfta_0002h15.t7	1		unclassified				ES779954
sb_gmnlkfta_0002h16.t7	1		unclassified				ES779955
sb_gmnlkfta_0002h19.t7	1		unclassified				ES779957
sb_gmnlkfta_0002h22.t7	1		unclassified				ES779959
sb_gmnlkfta_0002i01.t7	1		unclassified				ES779961
sb_gmnlkfta_0002i09.t7	1		Cluster: Ribosomal protein L8; n=3; Gnathostomata|Rep: Ribosomal protein L8 - Scyliorhinus canicula (Spotted dogfish) (Spotted		2e-25	59% (65/109)	ES779968

			catshark)				
sb_gmnlkfta_0002i14.t7	1		Cluster: Ribosomal protein L17; n=3; Euteleostomi|Rep: Ribosomal protein L17 - Siniperca chuatsi (Chinese perch)	GO:0003735 GO:0005622 GO:0005840 GO:0006412 GO:0015934 GO:0030529	5e-31	93% (59/63)	ES779970
sb_gmnlkfta_0002i16.t7	1		Cluster: Ras-related protein Rab-7; n=13; Amniota|Rep: Ras-related protein Rab-7 - Homo sapiens (Human)		4e-92	97% (162/166)	ES779972
sb_gmnlkfta_0002i17.t7	1		Cluster: Hemoglobin subunit beta; n=3; Gadidae|Rep: Hemoglobin subunit beta - Gadus morhua (Atlantic cod)		1e-10	88% (31/35)	ES779973
sb_gmnlkfta_0002i21.t7	1		Cluster: PREDICTED: similar to BSAC; n=1; Danio rerio|Rep: PREDICTED: similar to BSAC - Danio rerio		2e-24	44% (51/114)	ES779975
sb_gmnlkfta_0002i24.t7	1		Cluster: Sodium-dicarboxylate cotransporter; n=1; Pseudopleuronectes americanus|Rep: Sodium-dicarboxylate cotransporter - Pseudopleuronectes americanus (Winter flounder) (Pleuronectesamericanus)		2e-09	55% (29/52)	ES779976
sb_gmnlkfta_0002j01.t7	1		unclassified				ES779977
sb_gmnlkfta_0002j02.t7	1		LOC419218; similar to Protein KIAA1404		8e-48	68% (86/125)	ES779978
sb_gmnlkfta_0002j04.t7	1		unclassified				ES779979
sb_gmnlkfta_0002j07.t7	1		unclassified				ES779981
sb_gmnlkfta_0002j08.t7	1		CBX3; chromobox homolog 3 (HP1 gamma homolog, Drosophila)		1e-55	77% (63/81)	ES779982

sb_gmnlkfta_0002j09.t7	1		Cluster: 40S ribosomal protein S3a; n=49; Eukaryota|Rep: 40S ribosomal protein S3a - Homo sapiens (Human)		4e-77	89% (141/157)	ES779983
sb_gmnlkfta_0002j12.t7	1		Cluster: PREDICTED: similar to putative polyprotein; n=1; Danio rerio|Rep: PREDICTED: similar to putative polyprotein - Danio rerio		1e-05	56% (21/37)	ES779986
sb_gmnlkfta_0002j14.t7	1		unclassified				ES779988
sb_gmnlkfta_0002j15.t7	1		unclassified				ES779989
sb_gmnlkfta_0002j16.t7	1		Cluster: Roadblock-related dynein light chain; n=1; Ciona intestinalis|Rep: Roadblock-related dynein light chain - Ciona intestinalis (Transparent sea squirt)		6e-18	79% (43/54)	ES779990
sb_gmnlkfta_0002j18.t7	1		LOC480432; similar to Cytochrome p450 46A1 (Cholesterol 24-hydroxylase)		6e-33	61% (65/105)	ES779991
sb_gmnlkfta_0002j22.t7	1		Exonuc_X-T domain containing protein		5e-07	30% (33/108)	ES779994
sb_gmnlkfta_0002j23.t7	1		Cluster: Swelling dependent chloride channel, ICln; n=4; Danio rerio|Rep: Swelling dependent chloride channel, ICln - Brachydanio rerio (Zebrafish) (Danio rerio)		4e-05	66% (22/33)	ES779995
sb_gmnlkfta_0002k04.t7	1		Cluster: UPI0000D8D10D related cluster; n=1; Danio rerio|Rep: UPI0000D8D10D UniRef100 entry - Danio rerio		6e-12	55% (35/63)	ES779999
sb_gmnlkfta_0002k07.t7	1		unclassified				ES780001
sb_gmnlkfta_0002k08.t7	1		Cluster: Zgc:77429 protein; n=1; Danio rerio|Rep: Zgc:77429 protein - Brachydanio rerio (Zebrafish) (Danio rerio)		1e-27	64% (54/84)	ES780002

sb_gmnlkfta_0002k09.t7	1		Cluster: Bactericidal permeability increasing protein/lipopolysaccharide binding protein variant b; n=2; Gadus morhua|Rep: Bactericidal permeability increasing protein/lipopolysaccharide binding protein variant b - Gadus morhua (Atlantic cod)	GO:0008289	2e-11	66% (22/33)	ES780003
sb_gmnlkfta_0002k12.t7	1		Cluster: Similar to Tetraodon protein product CAG00085; n=1; Oncorhynchus mykiss|Rep: Similar to Tetraodon protein product CAG00085 - Oncorhynchus mykiss (Rainbow trout) (Salmo gairdneri)		5e-16	49% (37/75)	ES780005
sb_gmnlkfta_0002k13.t7	1		unclassified				ES780006
sb_gmnlkfta_0002k16.t7	1		unclassified				ES780008
sb_gmnlkfta_0002k21.t7	1		Cluster: Zgc:76977; n=4; Clupeocephala|Rep: Zgc:76977 - Brachydanio rerio (Zebrafish) (Danio rerio)		3e-84	83% (152/181)	ES780013
sb_gmnlkfta_0002l03.t7	1		Cluster: PREDICTED: similar to zinc finger protein 291; n=1; Danio rerio|Rep: PREDICTED: similar to zinc finger protein 291 - Danio rerio		2e-20	56% (36/64)	ES780017
sb_gmnlkfta_0002l10.t7	1		unclassified				ES780021
sb_gmnlkfta_0002l12.t7	1		LOC689197; similar to antigenic determinant of rec-A protein		3e-70	90% (119/132)	ES780023
sb_gmnlkfta_0002l15.t7	1		DUF625 domain containing protein		2e-08	53% (16/30)	ES780026
sb_gmnlkfta_0002l17.t7	1		Cluster: UPI0000E4EE3A related cluster; n=3; Danio rerio|Rep: UPI0000E4EE3A UniRef100 entry - Danio rerio		1e-29	86% (62/72)	ES780028
sb_gmnlkfta_0002l20.t7	1		unclassified				ES780029

sb_gmnlkfta_0002l22.t7	1		Cluster: PREDICTED: similar to Chain A, Tubulin-Colchicine-Vinblastine: Stathmin-Like Domain Complex; n=1; Gallus gallus|Rep: PREDICTED: similar to Chain A, Tubulin-Colchicine-Vinblastine: Stathmin-Like Domain Complex - Gallus gallus		4e-08	68% (22/32)	ES780031
sb_gmnlkfta_0002l23.t7	1		Cluster: mortality factor 4 like 1 isoform 1; n=2; Gallus gallus|Rep: mortality factor 4 like 1 isoform 1 - Gallus gallus		4e-51	85% (98/114)	ES780032
sb_gmnlkfta_0002m02.t7	1		unclassified				ES780035
sb_gmnlkfta_0002m03.t7	1		MGC109340; similar to Microsomal signal peptidase 23 kDa subunit (SPase 22 kDa subunit) (SPC22/23) [EC:3.4.-.-] [KO:K01423]		9e-09	38% (40/104)	ES780036
sb_gmnlkfta_0002m04.t7	1		Cluster: Sodium/potassium-transporting ATPase subunit beta-233; n=1; Anguilla anguilla|Rep: Sodium/potassium-transporting ATPase subunit beta-233 - Anguilla anguilla (European freshwater eel)	GO:0005391 GO:0005890 GO:0006810 GO:0006811 GO:0006813 GO:0006814 GO:0016020 GO:0016021 GO:0030955 GO:0031402	4e-30	70% (59/84)	ES780037
sb_gmnlkfta_0002m07.t7	1		LOC615842; similar to AMME syndrome candidate gene 1 protein		2e-55	89% (107/120)	ES780040
sb_gmnlkfta_0002m10.t7	1		Cluster: Zgc:86609; n=2; Danio rerio|Rep: Zgc:86609 - Brachydanio rerio (Zebrafish) (Danio rerio)		4e-49	90% (93/103)	ES780042
sb_gmnlkfta_0002m17.t7	1		unclassified				ES780048
sb_gmnlkfta_0002m19.t7	1		unclassified				ES780050
sb_gmnlkfta_0002m21.t7	1		unclassified				ES780052

sb_gmnlkfta_0002m22.t7	1		unclassified				ES780053
sb_gmnlkfta_0002m23.t7	1		unclassified				ES780054
sb_gmnlkfta_0002n06.t7	1		unclassified				ES780055
sb_gmnlkfta_0002n08.t7	1		Unassigned protein	GO:0002474<br>GO:0006955<br>GO:0042612			ES780057
sb_gmnlkfta_0002n12.t7	1		unclassified				ES780060
sb_gmnlkfta_0002n14.t7	1		unclassified				ES780062
sb_gmnlkfta_0002n15.t7	1		Cluster: 70kD heat shock protein; n=4; Takifugu rubripes|Rep: 70kD heat shock protein - Fugu rubripes (Japanese pufferfish) (Takifugu rubripes)	GO:0000166<br>GO:0005524<br>GO:0006950	2e-50	82% (97/117)	ES780063
sb_gmnlkfta_0002n17.t7	1		unclassified				ES780065
sb_gmnlkfta_0002n18.t7	1		Cluster: Transcription elongation factor SPT4; n=3; Clupeocephala|Rep: Transcription elongation factor SPT4 - Brachydanio rerio (Zebrafish) (Danio rerio)		9e-60	94% (110/117)	ES780066
sb_gmnlkfta_0002n19.t7	1		unclassified				ES780067
sb_gmnlkfta_0002n22.t7	1		unclassified				ES780069

sb_gmnlkfta_0002n24.t7	1		unclassified				ES780071
sb_gmnlkfta_0002o01.t7	1		unclassified				ES780072
sb_gmnlkfta_0002o05.t7	1		Cluster: Ribosomal protein S11; n=1; Pelodiscus sinensis|Rep: Ribosomal protein S11 - Trionyx sinensis (Chinese softshell turtle) (Pelodiscus sinensis)	GO:0003735 GO:0005622 GO:0005840 GO:0006412 GO:0030529	8e-08	100% (28/28)	ES780073
sb_gmnlkfta_0002o09.t7	1		unclassified				ES780075
sb_gmnlkfta_0002o10.t7	1		Cluster: Homolog of Oncorhynchus mykiss "Complement factor B/C2-B.; n=1; Takifugu rubripes|Rep: Homolog of Oncorhynchus mykiss "Complement factor B/C2-B. - Takifugu rubripes		2e-07	52% (31/59)	ES780076
sb_gmnlkfta_0002o11.t7	1		unclassified				ES780077
sb_gmnlkfta_0002o13.t7	1		unclassified				ES780078
sb_gmnlkfta_0002o14.t7	1		unclassified				ES780079
sb_gmnlkfta_0002o15.t7	1		cytochrome c oxidase subunit VIb precursor [Scombridae gen. sp.]		9e-21	86% (44/51)	ES780080
sb_gmnlkfta_0002o17.t7	1		unclassified				ES780081
sb_gmnlkfta_0002o18.t7	1		SPCS1, LOC415899; signal peptidase complex subunit 1 homolog (S. cerevisiae) [EC:3.4.-.-] [KO:K01423]		1e-25	77% (54/70)	ES780082

sb_gmnlkfta_0002o19.t7	1		Cluster: PREDICTED: similar to 60S ribosomal protein L17 (L23) (Amino acid starvation-induced protein) (ASI), partial; n=1; Gallus gallus|Rep: PREDICTED: similar to 60S ribosomal protein L17 (L23) (Amino acid starvation-induced protein) (ASI), partial - Gallus gallus		6e-19	100% (46/46)	ES780083
sb_gmnlkfta_0002o20.t7	1		unclassified				ES780084
sb_gmnlkfta_0002o21.t7	1		unclassified				ES780085
sb_gmnlkfta_0002p02.t7	1		unclassified				ES780088
sb_gmnlkfta_0002p03.t7	1		unclassified				ES780089
sb_gmnlkfta_0002p04.t7	1		unclassified				ES780090
sb_gmnlkfta_0002p11.t7	1		rap1a; RAP1A, member of RAS oncogene family [KO:K04353]		1e-13	100% (22/22)	ES780092
sb_gmnlkfta_0002p12.t7	1		Cluster: EIF4G-related protein NAT1A; n=3; Danio rerio|Rep: EIF4G-related protein NAT1A - Brachydanio rerio (Zebrafish) (Danio rerio)		3e-67	72% (111/154)	ES780093
sb_gmnlkfta_0002p13.t7	1		unclassified				ES780094
sb_gmnlkfta_0002p16.t7	1		Cluster: Cth protein; n=3; Danio rerio|Rep: Cth protein - Brachydanio rerio (Zebrafish) (Danio rerio)		3e-38	75% (72/95)	ES780096
sb_gmnlkfta_0002p19.t7	1		Cluster: Aspartyl-tRNA synthetase; n=5; Xenopus|Rep: Aspartyl-tRNA synthetase - Xenopus tropicalis (Western clawed frog) (Silurana tropicalis)	GO:0000166 GO:0003676 GO:0004812 GO:0004815 GO:0005524	2e-36	87% (74/85)	ES780098

				GO:0005737 GO:0006412 GO:0006418 GO:0006422 GO:0016874				
sb_gmnlkfta_0002p20.t7	1		unclassified				ES780099
sb_gmnlkfta_0002p21.t7	1		unclassified				ES780100
sb_gmnlkfta_0003a01.t7	1		unclassified				ES780102
sb_gmnlkfta_0003a03.t7	1		unclassified				ES780103
sb_gmnlkfta_0003a09.t7	1		unclassified				ES780106
sb_gmnlkfta_0003a10.t7	1		unclassified				ES780107
sb_gmnlkfta_0003a16.t7	1		LOC475043; similar to Ribonuclease UK114 (14.5 kDa translational inhibitor protein) (p14.5) (UK114 antigen homolog)		8e-22	75% (51/68)	ES780110
sb_gmnlkfta_0003a17.t7	1		unclassified				ES780111
sb_gmnlkfta_0003a19.t7	1		unclassified				ES780112
sb_gmnlkfta_0003a20.t7	1		unclassified				ES780113
sb_gmnlkfta_0003a23.t7	1		Unassigned protein				ES780114

sb_gmnlkfta_0003b11.t7	1		unclassified				ES780115
sb_gmnlkfta_0003b12.t7	1		Cluster: Homolog of Homo sapiens "Probable polypeptide N-acetylgalactosaminyltransferase 8; n=1; Takifugu rubripes|Rep: Homolog of Homo sapiens "Probable polypeptide N-acetylgalactosaminyltransferase 8 - Takifugu rubripes		2e-07	92% (24/26)	ES780116
sb_gmnlkfta_0003b15.t7	1		SSU rRNA; Phallusia mammilata		3e-28	95% (71/74)	EX189996
sb_gmnlkfta_0003b17.t7	1		unclassified				ES780118
sb_gmnlkfta_0003b19.t7	1		unclassified				ES780119
sb_gmnlkfta_0003b23.t7	1		unclassified				ES780120
sb_gmnlkfta_0003c01.t7	1		Cluster: PREDICTED: similar to cytidine deaminase; n=2; Danio rerio|Rep: PREDICTED: similar to cytidine deaminase - Danio rerio		7e-14	50% (28/56)	ES780121
sb_gmnlkfta_0003c02.t7	1		unclassified				ES780122
sb_gmnlkfta_0003c03.t7	1		unclassified				ES780123
sb_gmnlkfta_0003c05.t7	1		Cluster: Cathepsin D; n=1; Oncorhynchus mykiss|Rep: Cathepsin D - Oncorhynchus mykiss (Rainbow trout) (Salmo gairdneri)	GO:0004190 GO:0004194 GO:0006508	1e-18	74% (44/59)	ES780124
sb_gmnlkfta_0003c07.t7	1		Cluster: Peptidyl prolyl isomerase H; n=1; Danio rerio|Rep: Peptidyl prolyl isomerase H - Brachydanio rerio (Zebrafish) (Danio rerio)		2e-05	56% (23/41)	ES780125

sb_gmnlkfta_0003c12.t7	1		unclassified				ES780128
sb_gmnlkfta_0003c14.t7	1		Cluster: Chaperonin containing TCP1, subunit 6A; n=4; Euteleostomi|Rep: Chaperonin containing TCP1, subunit 6A - Brachydanio rerio (Zebrafish) (Danio rerio)		5e-15	97% (40/41)	ES780130
sb_gmnlkfta_0003c17.t7	1		unclassified				ES780131
sb_gmnlkfta_0003c19.t7	1		LOC607030; similar to myosin, light polypeptide 6, alkali, smooth muscle and non-muscle		5e-41	82% (78/94)	ES780132
sb_gmnlkfta_0003c20.t7	1		Cluster: Eukaryotic translation elongation factor 1 gamma; n=1; Latimeria chalumnae|Rep: Eukaryotic translation elongation factor 1 gamma - Latimeria chalumnae (Latimeria) (Coelacanth)		1e-13	82% (23/28)	ES780133
sb_gmnlkfta_0003c21.t7	1		unclassified				ES780134
sb_gmnlkfta_0003c22.t7	1		Cluster: Ribosomal protein L13; n=1; Protopterus dolloi|Rep: Ribosomal protein L13 - Protopterus dolloi (Slender lungfish)	GO:0003735 GO:0005622 GO:0005840 GO:0006412 GO:0030529	3e-30	53% (40/75)	ES780135
sb_gmnlkfta_0003c23.t7	1		unclassified				ES780136
sb_gmnlkfta_0003c24.t7	1		unclassified				ES780137
sb_gmnlkfta_0003d01.t7	1		LOC733940; novel protein		7e-22	81% (48/59)	ES780138
sb_gmnlkfta_0003d04.t7	1		unclassified				ES780140

sb_gmnlkfta_0003d06.t7	1		unclassified				ES780142
sb_gmnlkfta_0003d09.t7	1		MGC89650; MGC89650 protein [EC:3.1.3.2 3.1.3.48] [KO:K01078 K01104]		1e-17	65% (41/63)	ES780143
sb_gmnlkfta_0003d11.t7	1		ube2l3l; ubiquitin-conjugating enzyme E2L 3, like		1e-06	61% (16/26)	ES780144
sb_gmnlkfta_0003d13.t7	1		unclassified				ES780146
sb_gmnlkfta_0003d15.t7	1		unclassified				ES780147
sb_gmnlkfta_0003d17.t7	1		unclassified				ES780148
sb_gmnlkfta_0003d20.t7	1		unclassified				ES780149
sb_gmnlkfta_0003e01.t7	1		LOC568848; similar to cytidine deaminase		1e-08	50% (26/52)	ES780151
sb_gmnlkfta_0003e12.t7	1		Cluster: 40S ribosomal protein S17; n=22; Euteleostomi|Rep: 40S ribosomal protein S17 - Gallus gallus (Chicken)		7e-12	78% (26/33)	ES780154
sb_gmnlkfta_0003e13.t7	1		unclassified				ES780155
sb_gmnlkfta_0003e15.t7	1		unclassified				ES780157
sb_gmnlkfta_0003e17.t7	1		unclassified				ES780158

sb_gmnlkfta_0003e18.t7	1		Cluster: 60S acidic ribosomal protein P0; n=6; Euteleostomi|Rep: 60S acidic ribosomal protein P0 - Brachydanio rerio (Zebrafish) (Danio rerio)		6e-71	88% (133/151)	ES780159
sb_gmnlkfta_0003e20.t7	1		LOC615677; similar to Copine-1 (Copine I)		7e-45	50% (50/100)	ES780160
sb_gmnlkfta_0003e22.t7	1		LOC555695; similar to microtubule aggregate protein homolog		4e-39	42% (95/226)	ES780162
sb_gmnlkfta_0003e23.t7	1		unclassified				ES780163
sb_gmnlkfta_0003f01.t7	1		Cluster: ATPase family AAA domain-containing protein 1-B; n=2; Danio rerio|Rep: ATPase family AAA domain-containing protein 1-B - Brachydanio rerio (Zebrafish) (Danio rerio)		9e-45	91% (88/96)	ES780164
sb_gmnlkfta_0003f02.t7	1		unclassified				ES780165
sb_gmnlkfta_0003f03.t7	1		unclassified				ES780166
sb_gmnlkfta_0003f05.t7	1		SSU rRNA; Hyla chrysoscelis		0.0	93% (521/557)	EX189997
sb_gmnlkfta_0003f11.t7	1		unclassified				ES780168
sb_gmnlkfta_0003f12.t7	1		Cluster: Putative iodothyronine deiodinase type 1; n=1; Sparus aurata|Rep: Putative iodothyronine deiodinase type 1 - Sparus aurata (Gilthead sea bream)	GO:0004800	2e-33	58% (70/120)	ES780169
sb_gmnlkfta_0003f14.t7	1		Unassigned protein				ES780170

sb_gmnlkfta_0003f18.t7	1		unclassified				ES780172
sb_gmnlkfta_0003f19.t7	1		Unassigned protein	GO:0006464			ES780173
sb_gmnlkfta_0003f20.t7	1		unclassified				ES780174
sb_gmnlkfta_0003f21.t7	1		unclassified				ES780175
sb_gmnlkfta_0003g05.t7	1		unclassified				ES780177
sb_gmnlkfta_0003g07.t7	1		unclassified				ES780178
sb_gmnlkfta_0003g10.t7	1		Unassigned protein				ES780181
sb_gmnlkfta_0003g13.t7	1		Unassigned protein				ES780182
sb_gmnlkfta_0003g15.t7	1		unclassified				ES780183
sb_gmnlkfta_0003g20.t7	1		Cluster: Homolog of Danio rerio "MID1 interacting protein 1; n=1; Takifugu rubripes|Rep: Homolog of Danio rerio "MID1 interacting protein 1 - Takifugu rubripes		1e-37	80% (76/94)	ES780185
sb_gmnlkfta_0003g24.t7	1		unclassified				ES780186

sb_gmnlkfta_0003h01.t7	1		unclassified				ES780187
sb_gmnlkfta_0003h03.t7	1		unclassified				ES780188
sb_gmnlkfta_0003h05.t7	1		unclassified				ES780189
sb_gmnlkfta_0003h07.t7	1		Cluster: Barrier-to-autointegration factor; n=2; Clupeocephala|Rep: Barrier-to-autointegration factor - Brachydanio rerio (Zebrafish) (Danio rerio)		2e-19	89% (41/46)	ES780191
sb_gmnlkfta_0003h14.t7	1		Cluster: Ribosomal protein L7; n=4; Danio rerio|Rep: Ribosomal protein L7 - Brachydanio rerio (Zebrafish) (Danio rerio)		1e-46	81% (59/72)	ES780194
sb_gmnlkfta_0003h15.t7	1		barrier-to-autointegration factor [Danio rerio] sp|Q6P026|BAF_BRARE Barrier-to-autointegration factor gb|AAH65864.1| Zgc:77767 [Danio rerio]		5e-08	55% (27/49)	ES780195
sb_gmnlkfta_0003h17.t7	1		unclassified				ES780196
sb_gmnlkfta_0003h23.t7	1		unclassified				ES780198
sb_gmnlkfta_0003i01.t7	1		unclassified				ES780200
sb_gmnlkfta_0003i03.t7	1		unclassified				ES780201
sb_gmnlkfta_0003i04.t7	1		unclassified				ES780202

sb_gmnlkfta_0003i05.t7	1		Cluster: Actin related protein 2/3 complex, subunit 5B; n=2; Clupeocephala|Rep: Actin related protein 2/3 complex, subunit 5B - Brachydanio rerio (Zebrafish) (Danio rerio)		5e-15	97% (41/42)	ES780203
sb_gmnlkfta_0003i11.t7	1		LSU rRNA; Hydrolagus colliei		1e-88	97% (174/178)	EX189998
sb_gmnlkfta_0003i12.t7	1		Cluster: Novel protein similar to human breast cancer metastasis-suppressor 1; n=2; Danio rerio|Rep: Novel protein similar to human breast cancer metastasis-suppressor 1 - Brachydanio rerio (Zebrafish) (Danio rerio)		2e-29	80% (49/61)	ES780206
sb_gmnlkfta_0003i14.t7	1		Unassigned protein		1e-04	33% (37/109)	ES780207
sb_gmnlkfta_0003i16.t7	1		unclassified				ES780209
sb_gmnlkfta_0003i17.t7	1		unclassified				ES780210
sb_gmnlkfta_0003i19.t7	1		unclassified				ES780212
sb_gmnlkfta_0003j03.t7	1		Cluster: Hemoglobin subunit alpha-1; n=3; Gadidae|Rep: Hemoglobin subunit alpha-1 - Arctogadus glacialis (Arctic cod)	GO:0005344 GO:0005506 GO:0005833 GO:0006810 GO:0015671 GO:0019825 GO:0020037 GO:0046872	1e-07	100% (24/24)	ES780213
sb_gmnlkfta_0003j05.t7	1		unclassified				ES780214
sb_gmnlkfta_0003j07.t7	1		unclassified				ES780216

sb_gmnlkfta_0003j13.t7	1		unclassified				ES780217
sb_gmnlkfta_0003j15.t7	1		Cluster: 40S ribosomal protein S11; n=18; Euteleostomi|Rep: 40S ribosomal protein S11 - Xenopus laevis (African clawed frog)		4e-27	95% (58/61)	ES780218
sb_gmnlkfta_0003j18.t7	1		Cluster: 60S ribosomal protein L27; n=12; Amniota|Rep: 60S ribosomal protein L27 - Homo sapiens (Human)		3e-14	85% (41/48)	ES780219
sb_gmnlkfta_0003k03.t7	1		Cluster: LOC553404 protein; n=2; Danio rerio|Rep: LOC553404 protein - Brachydanio rerio (Zebrafish) (Danio rerio)		4e-31	48% (73/150)	ES780220
sb_gmnlkfta_0003k04.t7	1		Cluster: Homolog of Brachydanio rerio "Similar to DEAD (Asp-Glu-Ala-Asp) box polypeptide 48.; n=1; Takifugu rubripes|Rep: Homolog of Brachydanio rerio "Similar to DEAD (Asp-Glu-Ala-Asp) box polypeptide 48. - Takifugu rubripes		4e-36	97% (46/47)	ES780221
sb_gmnlkfta_0003k05.t7	1		unclassified				ES780222
sb_gmnlkfta_0003k08.t7	1		Cluster: N-acetylglucosamine-1-phosphate transferase, gamma subunit; n=2; Danio rerio|Rep: N-acetylglucosamine-1-phosphate transferase, gamma subunit - Brachydanio rerio (Zebrafish) (Danio rerio)		2e-08	42% (28/66)	ES780224
sb_gmnlkfta_0003k09.t7	1		unclassified				ES780225
sb_gmnlkfta_0003k11.t7	1		unclassified				ES780227
sb_gmnlkfta_0003k14.t7	1		unclassified				ES780230
sb_gmnlkfta_0003k15.t7	1		unclassified				ES780231

sb_gmnlkfta_0003k17.t7	1		unclassified				ES780232
sb_gmnlkfta_0003k23.t7	1		unclassified				ES780234
sb_gmnlkfta_0003l01.t7	1		unclassified				ES780236
sb_gmnlkfta_0003l02.t7	1		Unassigned protein				ES780237
sb_gmnlkfta_0003l07.t7	1		Cluster: Hsp90 co-chaperone Cdc37; n=3; Tetraodontidae|Rep: Hsp90 co-chaperone Cdc37 - Tetraodon fluviatilis (Puffer fish)	GO:0000074<br>GO:0005737<br>GO:0006457	3e-44	55% (87/157)	ES780240
sb_gmnlkfta_0003l08.t7	1		unclassified				ES780241
sb_gmnlkfta_0003l10.t7	1		unclassified				ES780242
sb_gmnlkfta_0003l12.t7	1		unclassified				ES780243
sb_gmnlkfta_0003l21.t7	1		unclassified				ES780247
sb_gmnlkfta_0003l23.t7	1		unclassified				ES780249
sb_gmnlkfta_0003l24.t7	1		unclassified				ES780250

sb_gmnlkfta_0003m05.t7	1		unclassified				ES780252
sb_gmnlkfta_0003m09.t7	1		unclassified				ES780253
sb_gmnlkfta_0003m10.t7	1		Unassigned protein				ES780254
sb_gmnlkfta_0003m11.t7	1		unclassified				ES780255
sb_gmnlkfta_0003m12.t7	1		unclassified				ES780256
sb_gmnlkfta_0003m14.t7	1		unclassified				ES780258
sb_gmnlkfta_0003m15.t7	1		Cluster: TATA binding protein associated factor 9; n=4; Percomorpha|Rep: TATA binding protein associated factor 9 - Sander vitreus	GO:0005669 GO:0006352	7e-55	95% (104/109)	ES780259
sb_gmnlkfta_0003m17.t7	1		Unassigned protein				ES780260
sb_gmnlkfta_0003m18.t7	1		unclassified				ES780261
sb_gmnlkfta_0003m20.t7	1		unclassified				ES780262
sb_gmnlkfta_0003m23.t7	1		unclassified				ES780263

sb_gmnlkfta_0003m24.t7	1		SSU rRNA; Apteryx australis		7e-38	95% (91/95)	EX189999
sb_gmnlkfta_0003n01.t7	1		unclassified				ES780264
sb_gmnlkfta_0003n02.t7	1		unclassified				ES780265
sb_gmnlkfta_0003n08.t7	1		unclassified				ES780267
sb_gmnlkfta_0003n15.t7	1		Cluster: MGC80188 protein; n=3; Xenopus|Rep: MGC80188 protein - Xenopus laevis (African clawed frog)		2e-05	95% (20/21)	ES780270
sb_gmnlkfta_0003n18.t7	1		unclassified				ES780272
sb_gmnlkfta_0003n21.t7	1		unclassified				ES780273
sb_gmnlkfta_0003n24.t7	1		unclassified				ES780275
sb_gmnlkfta_0003o02.t7	1		unclassified				ES780276
sb_gmnlkfta_0003o06.t7	1		Cluster: PREDICTED: similar to heat shock protein 90-alpha; n=1; Danio rerio|Rep: PREDICTED: similar to heat shock protein 90-alpha - Danio rerio		9e-30	92% (66/71)	ES780277
sb_gmnlkfta_0003o14.t7	1		unclassified				ES780280

sb_gmnlkfta_0003o23.t7	1		Cluster: PHD finger protein 6; n=2; Danio rerio|Rep: PHD finger protein 6 - Brachydanio rerio (Zebrafish) (Danio rerio)		5e-19	92% (26/28)	ES780283
sb_gmnlkfta_0003o24.t7	1		Cluster: MHC class I; n=6; Gadus morhua|Rep: MHC class I - Gadus morhua (Atlantic cod)	GO:0006955 GO:0016020 GO:0019882 GO:0042612	2e-22	68% (48/70)	ES780284
sb_gmnlkfta_0003p06.t7	1		unclassified				ES780285
sb_gmnlkfta_0003p07.t7	1		unclassified				ES780286
sb_gmnlkfta_0003p08.t7	1		unclassified				ES780287
sb_gmnlkfta_0003p11.t7	1		unclassified				ES780289
sb_gmnlkfta_0003p14.t7	1		unclassified				ES780290
sb_gmnlkfta_0003p17.t7	1		unclassified				ES780291
sb_gmnlkfta_0003p20.t7	1		Unassigned protein				ES780292
sb_gmnlkfta_0003p24.t7	1		Cluster: 40S ribosomal protein S9; n=17; Gnathostomata|Rep: 40S ribosomal protein S9 - Homo sapiens (Human)		7e-86	90% (159/175)	ES780293
sb_gmnlkfta_0004a01.t7	1		Unassigned protein				ES780294

sb_gmnlkfta_0004a03.t7	1		Cluster: Receptor-type tyrosine-protein phosphatase be; n=5; Eutheria|Rep: Receptor-type tyrosine-protein phosphatase be - Homo sapiens		2e-21	94% (47/50)	ES780295
sb_gmnlkfta_0004a05.t7	1		Cluster: PREDICTED: similar to adaptor-related protein complex 1, mu 1 subunit isoform 10; n=9; Canis familiaris|Rep: PREDICTED: similar to adaptor-related protein complex 1, mu 1 subunit isoform 10 - Canis familiaris		6e-07	52% (30/57)	ES780296
sb_gmnlkfta_0004a06.t7	1		unclassified				ES780297
sb_gmnlkfta_0004a08.t7	1		unclassified				ES780299
sb_gmnlkfta_0004a09.t7	1		unclassified				ES780300
sb_gmnlkfta_0004a13.t7	1		unclassified				ES780301
sb_gmnlkfta_0004a15.t7	1		Cluster: Small inducible cytokine SCYA104; n=1; Paralabidochromis chilotes|Rep: Small inducible cytokine SCYA104 - Paralabidochromis chilotes	GO:0005125 GO:0005576 GO:0005615 GO:0006955 GO:0008009	4e-11	39% (27/68)	ES780302
sb_gmnlkfta_0004a19.t7	1		Cluster: Ribophorin I; n=3; Danio rerio|Rep: Ribophorin I - Brachydanio rerio (Zebrafish) (Danio rerio)		1e-46	80% (93/115)	ES780304
sb_gmnlkfta_0004a20.t7	1		Cluster: Phosphoglucose isomerase; n=1; Mugil cephalus|Rep: Phosphoglucose isomerase - Mugil cephalus (Flathead mullet) (Mugil japonicus)		2e-42	94% (79/84)	ES780305
sb_gmnlkfta_0004a22.t7	1		Cluster: Cytosolic malate dehydrogenase A; n=4; Danio rerio|Rep: Cytosolic malate dehydrogenase A - Brachydanio rerio (Zebrafish) (Danio rerio)	GO:0006099 GO:0006100 GO:0006108 GO:0016491 GO:0016615	4e-44	95% (91/95)	ES780307

sb_gmnlkfta_0004a23.t7	1		unclassified				ES780308
sb_gmnlkfta_0004b08.t7	1		unclassified				ES780311
sb_gmnlkfta_0004b13.t7	1		Cluster: Phospholipid hydroperoxide glutathione peroxidase A; n=2; Danio rerio|Rep: Phospholipid hydroperoxide glutathione peroxidase A - Brachydanio rerio (Zebrafish) (Danio rerio)		2e-61	71% (42/59)	ES780316
sb_gmnlkfta_0004b14.t7	1		LSU rRNA; Hydrolagus colliei		6e-47	96% (108/112)	EX190000
sb_gmnlkfta_0004b15.t7	1		unclassified				ES780317
sb_gmnlkfta_0004b17.t7	1		unclassified				ES780318
sb_gmnlkfta_0004b24.t7	1		unclassified				ES780322
sb_gmnlkfta_0004c01.t7	1		unclassified				ES780323
sb_gmnlkfta_0004c02.t7	1		unclassified				ES780324
sb_gmnlkfta_0004c03.t7	1		Cluster: PREDICTED: similar to ring finger protein 157; n=2; Danio rerio|Rep: PREDICTED: similar to ring finger protein 157 - Danio rerio		4e-07	63% (28/44)	ES780325
sb_gmnlkfta_0004c04.t7	1		unclassified				ES780326

sb_gmnlkfta_0004c09.t7	1		unclassified				ES780329
sb_gmnlkfta_0004c15.t7	1		Cluster: Hemoglobin subunit alpha-1; n=3; Gadidae|Rep: Hemoglobin subunit alpha-1 - Arctogadus glacialis (Arctic cod)	GO:0005344 GO:0005506 GO:0005833 GO:0006810 GO:0015671 GO:0019825 GO:0020037 GO:0046872	4e-27	87% (34/39)	ES780333
sb_gmnlkfta_0004c16.t7	1		unclassified				ES780334
sb_gmnlkfta_0004c18.t7	1		unclassified				ES780335
sb_gmnlkfta_0004c19.t7	1		LSU rRNA; Hydrolagus colliei		1e-141	96% (290/302)	EX190001
sb_gmnlkfta_0004c21.t7	1		SSU rRNA; Hyla chrysoscelis		0.0	94% (586/622)	EX190002
sb_gmnlkfta_0004c24.t7	1		unclassified				ES780337
sb_gmnlkfta_0004d02.t7	1		Cluster: StAR-related lipid transfer protein 3; n=1; Salvelinus fontinalis|Rep: StAR-related lipid transfer protein 3 - Salvelinus fontinalis (Brook trout) (Brook char)		1e-28	86% (51/59)	ES780338
sb_gmnlkfta_0004d03.t7	1		Cluster: Methionyl aminopeptidase 2; n=4; Danio rerio|Rep: Methionyl aminopeptidase 2 - Brachydanio rerio (Zebrafish) (Danio rerio)		2e-47	96% (90/93)	ES780339
sb_gmnlkfta_0004d05.t7	1		Cluster: 60S ribosomal protein L30; n=1; Platichthys flesus|Rep: 60S ribosomal protein L30 - Platichthys flesus (European flounder)	GO:0003735 GO:0005622 GO:0005840 GO:0006412 GO:0030529	9e-21	98% (51/52)	ES780341

sb_gmnlkfta_0004d08.t7	1		Unassigned protein				ES780344
sb_gmnlkfta_0004d09.t7	1		LOC565377; similar to putative transmembrane protein TA-2		2e-52	67% (102/152)	ES780345
sb_gmnlkfta_0004d10.t7	1		Cluster: Erythrocyte carbonic anhydrase; n=1; Oncorhynchus mykiss|Rep: Erythrocyte carbonic anhydrase - Oncorhynchus mykiss (Rainbow trout) (Salmo gairdneri)	GO:0004089 GO:0006730 GO:0008270 GO:0016829	6e-58	86% (105/121)	ES780346
sb_gmnlkfta_0004d14.t7	1		Unassigned protein		1e-04	88% (22/25)	ES780349
sb_gmnlkfta_0004d17.t7	1		LOC556249; similar to Vacuolar protein sorting 13A (Chorein) (Chorea-acanthocytosis protein)		1e-56	56% (122/216)	ES780351
sb_gmnlkfta_0004d18.t7	1		LSU rRNA; Hydrolagus colliei		1e-163	97% (322/331)	EX190003
sb_gmnlkfta_0004d20.t7	1		Cluster: Ribosomal protein L5b; n=1; Ictalurus punctatus|Rep: Ribosomal protein L5b - Ictalurus punctatus (Channel catfish)		3e-07	96% (26/27)	ES780352
sb_gmnlkfta_0004d21.t7	1		Cluster: RAD21 homolog; n=2; Danio rerio|Rep: RAD21 homolog - Brachydanio rerio (Zebrafish) (Danio rerio)		2e-18	79% (42/53)	ES780353
sb_gmnlkfta_0004d22.t7	1		Cluster: Actin; n=2; Coelomata|Rep: Actin - Hydroides elegans (calcareous tube worm)		3e-05	75% (21/28)	ES780354
sb_gmnlkfta_0004e03.t7	1		unclassified				ES780357
sb_gmnlkfta_0004e04.t7	1		unclassified				ES780358

sb_gmnlkfta_0004e05.t7	1		PREDICTED: similar to ribosomal protein L36 [Rattus norvegicus]		9e-06	100% (24/24)	ES780359
sb_gmnlkfta_0004e08.t7	1		Cluster: PREDICTED: similar to CUB and zona pellucida-like domains 1; n=1; Danio rerio|Rep: PREDICTED: similar to CUB and zona pellucida-like domains 1 - Danio rerio		8e-23	50% (54/108)	ES780360
sb_gmnlkfta_0004e14.t7	1		LOC417871; similar to 40S ribosomal protein S16	GO:0003735 GO:0005622 GO:0005840 GO:0006412 GO:0030529	1e-05	100% (24/24)	ES780365
sb_gmnlkfta_0004e15.t7	1		unclassified				ES780366
sb_gmnlkfta_0004e17.t7	1		unclassified				ES780367
sb_gmnlkfta_0004e19.t7	1		LSU rRNA; Anguilla rostrata		2e-65	92% (166/179)	EX190004
sb_gmnlkfta_0004e20.t7	1		Cluster: 60S ribosomal protein L9; n=4; Otophysi|Rep: 60S ribosomal protein L9 - Ictalurus punctatus (Channel catfish)		5e-09	88% (30/34)	ES780369
sb_gmnlkfta_0004e21.t7	1		Cluster: GTP-binding protein SAR1a; n=1; Homo sapiens|Rep: GTP-binding protein SAR1a - Homo sapiens		2e-89	90% (162/180)	ES780370
sb_gmnlkfta_0004e22.t7	1		Uqcrq; ubiquinol-cytochrome c reductase, complex III subunit VII [EC:1.10.2.2] [KO:K00418]	GO:0006118 GO:0008121	1e-04	81% (13/16)	ES780371
sb_gmnlkfta_0004f02.t7	1		unclassified				ES780372
sb_gmnlkfta_0004f04.t7	1		unclassified				ES780373

sb_gmnlkfta_0004f05.t7	1		Cluster: PREDICTED: ribosomal protein L35; n=1; Bos taurus|Rep: PREDICTED: ribosomal protein L35 - Bos taurus		1e-14	72% (42/58)	ES780374
sb_gmnlkfta_0004f08.t7	1		unclassified				ES780376
sb_gmnlkfta_0004f12.t7	1		Cluster: Elongation factor 1-alpha; n=1; Trichophyton rubrum|Rep: Elongation factor 1-alpha - Trichophyton rubrum	GO:0000166 GO:0003746 GO:0003924 GO:0005525 GO:0005737 GO:0006412 GO:0006414	8e-23	76% (36/47)	ES780380
sb_gmnlkfta_0004f13.t7	1		Cluster: Toll-like receptor 3; n=2; Paralichthys olivaceus|Rep: Toll-like receptor 3 - Paralichthys olivaceus (Japanese flounder)	GO:0004872 GO:0004888 GO:0005515 GO:0016020	4e-06	62% (25/40)	ES780381
sb_gmnlkfta_0004f16.t7	1		unclassified				ES780382
sb_gmnlkfta_0004f18.t7	1		Cluster: Basic leucine zipper and W2 domain-containing protein 1-A; n=4; Clupeocephala|Rep: Basic leucine zipper and W2 domain-containing protein 1-A - Brachydanio rerio (Zebrafish) (Danio rerio)		5e-31	55% (77/139)	ES780383
sb_gmnlkfta_0004f20.t7	1		unclassified				ES780384
sb_gmnlkfta_0004f21.t7	1		unclassified				ES780385
sb_gmnlkfta_0004g01.t7	1		unclassified				ES780388
sb_gmnlkfta_0004g03.t7	1		unclassified				ES780390

sb_gmnlkfta_0004g04.t7	1		unclassified				ES780391
sb_gmnlkfta_0004g05.t7	1		Cluster: Ceruloplasmin; n=6; Danio rerio|Rep: Ceruloplasmin - Brachydanio rerio (Zebrafish) (Danio rerio)		4e-60	63% (65/103)	ES780392
sb_gmnlkfta_0004g06.t7	1		unclassified				ES780393
sb_gmnlkfta_0004g08.t7	1		unclassified				ES780394
sb_gmnlkfta_0004g10.t7	1		unclassified				ES780396
sb_gmnlkfta_0004g12.t7	1		Cluster: Homolog of Homo sapiens "NADH-ubiquinone oxidoreductase SGDH subunit, mitochondrial precursor; n=1; Takifugu rubripes|Rep: Homolog of Homo sapiens "NADH-ubiquinone oxidoreductase SGDH subunit, mitochondrial precursor - Takifugu rubripes		2e-23	72% (32/44)	ES780397
sb_gmnlkfta_0004g16.t7	1		Cluster: T-complex protein 1 subunit alpha; n=3; Archosauria|Rep: T-complex protein 1 subunit alpha - Paleosuchus palpebrosus (Cuvier's dwarf caiman) (Red caiman)		4e-48	83% (94/113)	ES780398
sb_gmnlkfta_0004g19.t7	1		LSU rRNA; Anguilla rostrata		2e-59	91% (166/181)	EX190005
sb_gmnlkfta_0004g21.t7	1		unclassified				ES780400
sb_gmnlkfta_0004g22.t7	1		unclassified				ES780401
sb_gmnlkfta_0004h01.t7	1		Cluster: Annexin max4; n=1; Oryzias latipes|Rep: Annexin max4 - Oryzias latipes (Medaka fish) (Japanese ricefish)	GO:0005509 GO:0005544	1e-33	62% (52/83)	ES780403

sb_gmnlkfta_0004h04.t7	1		unclassified				ES780405
sb_gmnlkfta_0004h06.t7	1		Unassigned protein				ES780406
sb_gmnlkfta_0004h15.t7	1		Cluster: Protein tyrosine kinase; n=1; Ephydatia fluviatilis|Rep: Protein tyrosine kinase - Ephydatia fluviatilis		6e-21	49% (32/65)	ES780410
sb_gmnlkfta_0004h16.t7	1		unclassified				ES780411
sb_gmnlkfta_0004h20.t7	1		unclassified				ES780414
sb_gmnlkfta_0004h22.t7	1		Cluster: Ribosomal protein L17; n=3; Euteleostomi|Rep: Ribosomal protein L17 - Siniperca chuatsi (Chinese perch)	GO:0003735 GO:0005622 GO:0005840 GO:0006412 GO:0015934 GO:0030529	1e-13	100% (31/31)	ES780415
sb_gmnlkfta_0004h24.t7	1		unclassified				ES780416
sb_gmnlkfta_0004i05.t7	1		Cluster: PREDICTED: similar to hect domain and RLD 4 (predicted); n=1; Danio rerio|Rep: PREDICTED: similar to hect domain and RLD 4 (predicted) - Danio rerio		3e-17	46% (41/89)	ES780419
sb_gmnlkfta_0004i06.t7	1		unclassified				ES780420
sb_gmnlkfta_0004i07.t7	1		Cluster: Ubiquitin.; n=5; Amniota|Rep: Ubiquitin. - Canis familiaris		1e-38	97% (81/83)	ES780421
sb_gmnlkfta_0004i09.t7	1		unclassified				ES780423

sb_gmnlkfta_0004i24.t7	1		unclassified				ES780427
sb_gmnlkfta_0004j02.t7	1		Cluster: Homolog of Brachydanio rerio "Protein tyrosine phosphatase, non-receptor type 6.; n=1; Takifugu rubripes|Rep: Homolog of Brachydanio rerio "Protein tyrosine phosphatase, non-receptor type 6. - Takifugu rubripes		1e-49	70% (95/135)	ES780428
sb_gmnlkfta_0004j04.t7	1		unclassified				ES780430
sb_gmnlkfta_0004j05.t7	1		unclassified				ES780431
sb_gmnlkfta_0004j08.t7	1		unclassified				ES780434
sb_gmnlkfta_0004j12.t7	1		LOC568934; similar to Zinc finger protein 185 (LIM-domain protein ZNF185) (P1-A)		4e-10	72% (24/33)	ES780437
sb_gmnlkfta_0004j13.t7	1		Cluster: Hypoxanthine-guanine phosphoribosyltransferase; n=15; Euteleostomi|Rep: Hypoxanthine-guanine phosphoribosyltransferase - Gallus gallus (Chicken)		4e-72	88% (134/152)	ES780438
sb_gmnlkfta_0004j14.t7	1		Cluster: Hemoglobin subunit alpha-1; n=3; Gadidae|Rep: Hemoglobin subunit alpha-1 - Arctogadus glacialis (Arctic cod)	GO:0005344 GO:0005506 GO:0005833 GO:0006810 GO:0015671 GO:0019825 GO:0020037 GO:0046872	8e-30	94% (64/68)	ES780439
sb_gmnlkfta_0004j16.t7	1		unclassified				ES780440
sb_gmnlkfta_0004j18.t7	1		unclassified				ES780441

sb_gmnlkfta_0004j20.t7	1		Cluster: O-sialoglycoprotein endopeptidase; n=2; Xenopus|Rep: O-sialoglycoprotein endopeptidase - Xenopus tropicalis (Western clawed frog) (Silurana tropicalis)		9e-44	90% (86/95)	ES780442
sb_gmnlkfta_0004j21.t7	1		unclassified				ES780443
sb_gmnlkfta_0004j22.t7	1		unclassified				ES780444
sb_gmnlkfta_0004j24.t7	1		unclassified				ES780445
sb_gmnlkfta_0004k04.t7	1		Cluster: Samsn1 protein; n=5; Danio rerio|Rep: Samsn1 protein - Brachydanio rerio (Zebrafish) (Danio rerio)		3e-51	57% (105/183)	ES780447
sb_gmnlkfta_0004k05.t7	1		Cluster: PREDICTED: similar to ribosomal protein L10a isoform 2; n=1; Canis familiaris|Rep: PREDICTED: similar to ribosomal protein L10a isoform 2 - Canis familiaris		2e-15	78% (26/33)	ES780448
sb_gmnlkfta_0004k08.t7	1		unclassified				ES780450
sb_gmnlkfta_0004k09.t7	1		unclassified				ES780451
sb_gmnlkfta_0004k10.t7	1		unclassified				ES780452
sb_gmnlkfta_0004k12.t7	1		Cluster: Ribosomal protein L6; n=1; Ictalurus punctatus|Rep: Ribosomal protein L6 - Ictalurus punctatus (Channel catfish)	GO:0003735 GO:0005622 GO:0005840 GO:0006412 GO:0030529	3e-18	76% (43/56)	ES780454
sb_gmnlkfta_0004k13.t7	1		Cluster: PREDICTED: similar to chaperone protein GP96 isoform 10; n=3; Danio rerio|Rep: PREDICTED: similar to chaperone protein GP96 isoform 10 - Danio rerio		2e-70	82% (136/165)	ES780455

sb_gmnlkfta_0004k16.t7	1		unclassified				ES780458
sb_gmnlkfta_0004k17.t7	1		Cluster: Ribosomal protein L5a; n=4; Otophysi|Rep: Ribosomal protein L5a - Brachydanio rerio (Zebrafish) (Danio rerio)		4e-11	72% (35/48)	ES780459
sb_gmnlkfta_0004k21.t7	1		Cluster: Homolog of Brachydanio rerio "Hydroxyacyl glutathione hydrolase (Zgc:73161 protein).; n=1; Takifugu rubripes|Rep: Homolog of Brachydanio rerio "Hydroxyacyl glutathione hydrolase (Zgc:73161 protein). - Takifugu rubripes		2e-45	82% (79/96)	ES780461
sb_gmnlkfta_0004k22.t7	1		Cluster: PREDICTED: similar to Ribosomal protein L8 isoform 1; n=2; Eutheria|Rep: PREDICTED: similar to Ribosomal protein L8 isoform 1 - Pan troglodytes		7e-16	97% (35/36)	ES780462
sb_gmnlkfta_0004k24.t7	1		unclassified				ES780464
sb_gmnlkfta_0004l01.t7	1		unclassified				ES780465
sb_gmnlkfta_0004l02.t7	1		unclassified				ES780466
sb_gmnlkfta_0004l05.t7	1		Cluster: UPI0000D8E5FE related cluster; n=1; Danio rerio|Rep: UPI0000D8E5FE UniRef100 entry - Danio rerio		2e-06	35% (25/70)	ES780468
sb_gmnlkfta_0004l08.t7	1		unclassified				ES780471
sb_gmnlkfta_0004l09.t7	1		unclassified				ES780472
sb_gmnlkfta_0004l10.t7	1		unclassified				ES780473

sb_gmnlkfta_0004l11.t7	1		unclassified				ES780474
sb_gmnlkfta_0004l12.t7	1		unclassified				ES780475
sb_gmnlkfta_0004l14.t7	1		unclassified				ES780477
sb_gmnlkfta_0004l16.t7	1		unclassified				ES780479
sb_gmnlkfta_0004l17.t7	1		unclassified				ES780480
sb_gmnlkfta_0004l20.t7	1		Cluster: Homolog of Homo sapiens "Leukocyte formin; n=1; Takifugu rubripes|Rep: Homolog of Homo sapiens "Leukocyte formin - Takifugu rubripes		2e-19	81% (49/60)	ES780483
sb_gmnlkfta_0004l21.t7	1		unclassified				ES780484
sb_gmnlkfta_0004l24.t7	1		LSU rRNA; Hydrolagus colliei		4e-50	97% (106/109)	ES780486
sb_gmnlkfta_0004m01.t7	1		Cluster: LSM12 homolog; n=4; Clupeocephala|Rep: LSM12 homolog - Brachydanio rerio (Zebrafish) (Danio rerio)		6e-48	95% (71/74)	ES780487
sb_gmnlkfta_0004m03.t7	1		LOC572803; similar to lipoic acid synthetase isoform 1 precursor		1e-24	85% (52/61)	ES780489
sb_gmnlkfta_0004m04.t7	1		unclassified				ES780490

sb_gmnlkfta_0004m05.t7	1		Cluster: Homolog of Gallus gallus "Ig mu chain C region.; n=1; Takifugu rubripes|Rep: Homolog of Gallus gallus "Ig mu chain C region. - Takifugu rubripes		4e-08	33% (29/86)	ES780491
sb_gmnlkfta_0004m06.t7	1		Cluster: Ribophorin I; n=3; Danio rerio|Rep: Ribophorin I - Brachydanio rerio (Zebrafish) (Danio rerio)		8e-35	93% (75/80)	ES780492
sb_gmnlkfta_0004m07.t7	1		unclassified				ES780493
sb_gmnlkfta_0004m12.t7	1		Cluster: Cathepsin S-like; n=1; Oncorhynchus mykiss|Rep: Cathepsin S-like - Oncorhynchus mykiss (Rainbow trout) (Salmo gairdneri)	GO:0004197 GO:0006508 GO:0008234	6e-32	55% (49/88)	ES780496
sb_gmnlkfta_0004m13.t7	1		unclassified				ES780497
sb_gmnlkfta_0004m18.t7	1		Cluster: Simple type II keratin K8a; n=3; Oncorhynchus|Rep: Simple type II keratin K8a - Oncorhynchus mykiss (Rainbow trout) (Salmo gairdneri)	GO:0005198 GO:0005882	1e-26	97% (42/43)	ES780500
sb_gmnlkfta_0004m21.t7	1		Cluster: Homolog of Paralichthys olivaceus "Complement component C3.; n=1; Takifugu rubripes|Rep: Homolog of Paralichthys olivaceus "Complement component C3. - Takifugu rubripes	GO:0004866 GO:0005515 GO:0005576	2e-46	52% (100/191)	ES780502
sb_gmnlkfta_0004m23.t7	1		Cluster: Homolog of Homo sapiens "Maltase-glucoamylase, intestinal; n=1; Takifugu rubripes|Rep: Homolog of Homo sapiens "Maltase-glucoamylase, intestinal - Takifugu rubripes		1e-18	51% (25/49)	ES780504
sb_gmnlkfta_0004n03.t7	1		unclassified				ES780506
sb_gmnlkfta_0004n04.t7	1		Cluster: Homolog of Homo sapiens "Glioma tumor suppressor candidate region gene 2 protein; n=1; Takifugu rubripes|Rep: Homolog of Homo sapiens "Glioma tumor suppressor candidate region gene 2 protein - Takifugu rubripes		8e-33	45% (85/185)	ES780507

sb_gmnlkfta_0004n05.t7	1		unclassified				ES780508
sb_gmnlkfta_0004n06.t7	1		unclassified				ES780509
sb_gmnlkfta_0004n08.t7	1		unclassified				ES780510
sb_gmnlkfta_0004n11.t7	1		Cluster: Annexin max2; n=1; Oryzias latipes|Rep: Annexin max2 - Oryzias latipes (Medaka fish) (Japanese ricefish)	GO:0005509<br>GO:0005544<br>GO:0050819	6e-79	82% (152/185)	ES780513
sb_gmnlkfta_0004n12.t7	1		Cluster: Homolog of Brachydanio rerio "Sulfotransferase family 1, cytosolic sulfotransferase 3.; n=2; Takifugu rubripes|Rep: Homolog of Brachydanio rerio "Sulfotransferase family 1, cytosolic sulfotransferase 3. - Takifugu rubripes	GO:0008146<br>GO:0016740	2e-28	76% (56/73)	ES780514
sb_gmnlkfta_0004n15.t7	1		Unassigned protein				ES780517
sb_gmnlkfta_0004n16.t7	1		unclassified				ES780518
sb_gmnlkfta_0004n18.t7	1		unclassified				ES780519
sb_gmnlkfta_0004n19.t7	1		Cluster: Pantophysin; n=13; Theragra chalcogramma|Rep: Pantophysin - Theragra chalcogramma (Alaska pollock)	GO:0005215<br>GO:0006810<br>GO:0008021<br>GO:0016020<br>GO:0016021	3e-18	83% (31/37)	ES780520
sb_gmnlkfta_0004n20.t7	1		Cluster: Transaldolase; n=1; Ctenopharyngodon idella|Rep: Transaldolase - Ctenopharyngodon idella (Grass carp)	GO:0003824<br>GO:0004801<br>GO:0005737<br>GO:0005975<br>GO:0006098<br>GO:0008152	9e-27	73% (57/78)	ES780521

sb_gmnlkfta_0004n21.t7	1		unclassified				ES780522
sb_gmnlkfta_0004n23.t7	1		unclassified				ES780524
sb_gmnlkfta_0004o04.t7	1		Ms4a4d; membrane-spanning 4-domains, subfamily A, member 4D		2e-06	34% (32/92)	ES780526
sb_gmnlkfta_0004o09.t7	1		Cluster: Novel protein; n=3; Danio rerio|Rep: Novel protein - Brachydanio rerio (Zebrafish) (Danio rerio)		3e-20	43% (40/93)	ES780529
sb_gmnlkfta_0004o11.t7	1		unclassified				ES780530
sb_gmnlkfta_0004o12.t7	1		unclassified				ES780531
sb_gmnlkfta_0004o15.t7	1		TFIIF_alpha domain containing protein		7e-05	38% (15/39)	ES780533
sb_gmnlkfta_0004o16.t7	1		Cluster: PREDICTED: similar to Sodium/hydrogen exchanger 2 (Na(+)/H(+) exchanger 2) (NHE-2) (H7); n=1; Danio rerio|Rep: PREDICTED: similar to Sodium/hydrogen exchanger 2 (Na(+)/H(+) exchanger 2) (NHE-2) (H7) - Danio rerio		3e-19	73% (30/41)	ES780534
sb_gmnlkfta_0004o23.t7	1		Cluster: 60S ribosomal protein L9; n=4; Otophysi|Rep: 60S ribosomal protein L9 - Ictalurus punctatus (Channel catfish)		8e-20	87% (51/58)	ES780540
sb_gmnlkfta_0004o24.t7	1		unclassified				ES780541
sb_gmnlkfta_0004p01.t7	1		Cluster: Aspartate aminotransferase; n=2; Euteleostei|Rep: Aspartate aminotransferase - Oncorhynchus tschawytscha (Chinook salmon) (King salmon)		3e-06	96% (25/26)	ES780542

sb_gmnlkfta_0004p02.t7	1		unclassified				ES780543
sb_gmnlkfta_0004p04.t7	1		unclassified				ES780544
sb_gmnlkfta_0004p08.t7	1		Unassigned protein				ES780545
sb_gmnlkfta_0004p09.t7	1		unclassified				ES780546
sb_gmnlkfta_0004p14.t7	1		unclassified				ES780548
sb_gmnlkfta_0004p18.t7	1		unclassified				ES780550
sb_gmnlkfta_0004p19.t7	1		Cluster: Glycogen phosphorylase; n=4; Euteleostomi|Rep: Glycogen phosphorylase - Oreochromis mossambicus (Mozambique tilapia) (Tilapia mossambica)		2e-15	96% (25/26)	ES780551
sb_gmnlkfta_0004p21.t7	1		unclassified				ES780553
sb_gmnlkfta_0004p22.t7	1		Unassigned protein		9e-06	56% (17/30)	ES780554
sb_gmnlkfta_0004p23.t7	1		Cluster: Simple type II keratin K8a; n=3; Oncorhynchus|Rep: Simple type II keratin K8a - Oncorhynchus mykiss (Rainbow trout) (Salmo gairdneri)	GO:0005198 GO:0005882	2e-23	96% (48/50)	ES780555
sb_gmnlkfta_0004p24.t7	1		Cluster: Protein disulfide isomerase associated 4; n=3; Danio rerio|Rep: Protein disulfide isomerase associated 4 - Brachydanio rerio (Zebrafish) (Danio rerio)		4e-60	71% (79/111)	ES780556

sb_gmnlkfta_0006a01.t7	1		Cluster: Leukemia inhibitory factor receptor; n=1; Carassius auratus|Rep: Leukemia inhibitory factor receptor - Carassius auratus (Goldfish)	GO:0004872	1e-10	32% (24/73)	EX190006
sb_gmnlkfta_0006a04.t7	1		unclassified				EX190007
sb_gmnlkfta_0006a09.t7	1		unclassified				EX190012
sb_gmnlkfta_0006a10.t7	1		Cluster: PREDICTED: tubulin, alpha, ubiquitous isoform 8; n=5; Deuterostomia|Rep: PREDICTED: tubulin, alpha, ubiquitous isoform 8 - Macaca mulatta		6e-67	94% (123/130)	EX190013
sb_gmnlkfta_0006a12.t7	1		ppp6c; protein phosphatase 6, catalytic subunit [EC:3.1.3.16]; K01090 protein phosphatase		1e-129	98% (215/218)	EX190015
sb_gmnlkfta_0006a14.t7	1		Cluster: GTP binding protein NGB; n=3; Danio rerio|Rep: GTP binding protein NGB - Danio rerio (Zebrafish) (Brachydanio rerio)		3e-48	96% (92/95)	EX190017
sb_gmnlkfta_0006a16.t7	1		Cluster: Heat shock protein 90 beta; n=7; Euteleostomi|Rep: Heat shock protein 90 beta - Paralichthys olivaceus (Japanese flounder)	GO:0000166 GO:0005524 GO:0006457 GO:0051082 GO:0006950	8e-46	93% (92/98)	EX190019
sb_gmnlkfta_0006a17.t7	1		unclassified				EX190020
sb_gmnlkfta_0006a18.t7	1		unclassified				EX190021
sb_gmnlkfta_0006a20.t7	1		Cluster: Homolog of Brachydanio rerio "Eukaryotic translation elongation factor 2, like.; n=1; Takifugu rubripes|Rep: Homolog of Brachydanio rerio "Eukaryotic translation elongation factor 2, like. - Takifugu rubripes		7e-30	70% (60/85)	EX190023
sb_gmnlkfta_0006a21.t7	1		Cluster: PREDICTED: similar to Guanine nucleotide-binding protein G(k), alpha subunit (G(i) alpha-3); n=1; Canis lupus		9e-20	95%	EX190024

			familiaris|Rep: PREDICTED: similar to Guanine nucleotide-binding protein G(k), alpha subunit (G(i) alpha-3) - Canis familiaris			(46/48)	
sb_gmnlkfta_0006a22.t7	1		unclassified				EX190025
sb_gmnlkfta_0006b03.t7	1		Cluster: PREDICTED: similar to Actin, cytoplasmic 2 (Gamma-actin); n=1; Rattus norvegicus|Rep: PREDICTED: similar to Actin, cytoplasmic 2 (Gamma-actin) - Rattus norvegicus		8e-32	94% (68/72)	EX190029
sb_gmnlkfta_0006b04.t7	1		unclassified				EX190030
sb_gmnlkfta_0006b05.t7	1		unclassified				EX190031
sb_gmnlkfta_0006b06.t7	1		unclassified				EX190032
sb_gmnlkfta_0006b07.t7	1		Cluster: 26 proteasome complex subunit DSS1; n=11; Euteleostomi|Rep: 26 proteasome complex subunit DSS1 - Homo sapiens (Human)		6e-12	61% (39/63)	EX190033
sb_gmnlkfta_0006b14.t7	1		unclassified				EX190040
sb_gmnlkfta_0006b15.t7	1		Unassigned protein				EX190041
sb_gmnlkfta_0006b16.t7	1		unclassified				EX190042
sb_gmnlkfta_0006b18.t7	1		Cluster: PREDICTED: similar to mitogen-activated protein kinase 13; n=1; Monodelphis domestica|Rep: PREDICTED: similar to mitogen-activated protein kinase 13 - Monodelphis domestica		1e-23	92% (49/53)	EX190044
sb_gmnlkfta_0006b20.t7	1		Cluster: Heat shock protein 90 beta; n=7; Euteleostomi|Rep: Heat	GO:0000166 GO:0005524	8e-46	93%	EX190046

			shock protein 90 beta - Paralichthys olivaceus (Japanese flounder)	GO:0006457 GO:0051082 GO:0006950		(92/98)	
sb_gmnlkfta_0006b21.t7	1		unclassified				EX190047
sb_gmnlkfta_0006b22.t7	1		unclassified				EX190048
sb_gmnlkfta_0006b23.t7	1		unclassified				EX190049
sb_gmnlkfta_0006c02.t7	1		Unassigned protein				EX190051
sb_gmnlkfta_0006c03.t7	1		unclassified				EX190052
sb_gmnlkfta_0006c05.t7	1		unclassified				EX190054
sb_gmnlkfta_0006c07.t7	1		Cluster: Sodium potassium ATPase beta subunit; n=1; Rhabdosargus sarba|Rep: Sodium potassium ATPase beta subunit - Rhabdosargus sarba (goldlined seabream)	GO:0005391 GO:0006813 GO:0006814 GO:0016020	3e-22	63% (51/80)	EX190056
sb_gmnlkfta_0006c09.t7	1		unclassified				EX190058
sb_gmnlkfta_0006c12.t7	1		Cluster: 60S ribosomal protein L40; n=3; Eukaryota|Rep: 60S ribosomal protein L40 - Cryptosporidium parvum Iowa II	GO:0003735 GO:0005622 GO:0005840 GO:0006412 GO:0006464 GO:0004252 GO:0006508 GO:0016032 GO:0019082	4e-24	98% (56/57)	EX190060
sb_gmnlkfta_0006c15.t7	1		Cluster: PREDICTED: similar to Ribosomal protein L8 isoform 1; n=2; Eutheria|Rep: PREDICTED: similar to Ribosomal protein L8		1e-25	98%	EX190063

			isoform 1 - Pan troglodytes			(53/54)	
sb_gmnlkfta_0006c17.t7	1		Cluster: Cathepsin L; n=1; Oryzias latipes|Rep: Cathepsin L - Oryzias latipes (Medaka fish) (Japanese ricefish)	GO:0004197 GO:0006508 GO:0008233 GO:0008234 GO:0016787	9e-54	78% (90/114)	EX190065
sb_gmnlkfta_0006c18.t7	1		unclassified				EX190066
sb_gmnlkfta_0006c19.t7	1		MGC139072; similar to ATP synthase mitochondrial F1 complex assembly factor 2		6e-18	68% (37/54)	EX190067
sb_gmnlkfta_0006c22.t7	1		Cluster: PREDICTED: similar to Tubulin beta-6 chain (Beta-tubulin class-VI); n=2; Danio rerio|Rep: PREDICTED: similar to Tubulin beta-6 chain (Beta-tubulin class-VI) - Danio rerio		2e-13	65% (31/47)	EX190069
sb_gmnlkfta_0006c23.t7	1		Cluster: Novel protein similar to sorting nexin 9; n=2; Danio rerio|Rep: Novel protein similar to sorting nexin 9 - Danio rerio (Zebrafish) (Brachydanio rerio)		1e-08	84% (28/33)	EX190070
sb_gmnlkfta_0006d01.t7	1		unclassified				EX190072
sb_gmnlkfta_0006d09.t7	1		Cluster: Alpha-aminoadipic semialdehyde dehydrogenase; n=12; Eutheria|Rep: Alpha-aminoadipic semialdehyde dehydrogenase - Homo sapiens (Human)		2e-16	87% (41/47)	EX190077
sb_gmnlkfta_0006d10.t7	1		Cluster: PREDICTED: similar to large subunit ribosomal protein L36a isoform 3; n=1; Macaca mulatta|Rep: PREDICTED: similar to large subunit ribosomal protein L36a isoform 3 - Macaca mulatta		6e-46	91% (88/96)	EX190078
sb_gmnlkfta_0006d12.t7	1		unclassified				EX190080
sb_gmnlkfta_0006d13.t7	1		unclassified				EX190081

sb_gmnlkfta_0006d18.t7	1		Cluster: Homolog of Oreochromis niloticus "Carbonyl reductase-like 20beta-hydroxysteroid dehydrogenase.; n=1; Takifugu rubripes|Rep: Homolog of Oreochromis niloticus "Carbonyl reductase-like 20beta-hydroxysteroid dehydrogenase. - Takifugu rubripes		3e-10	70% (29/41)	EX190085
sb_gmnlkfta_0006d19.t7	1		Cluster: PREDICTED: similar to Chain A, Structure Of Wdr5; n=1; Monodelphis domestica|Rep: PREDICTED: similar to Chain A, Structure Of Wdr5 - Monodelphis domestica		8e-31	94% (63/67)	EX190086
sb_gmnlkfta_0006d21.t7	1		unclassified				EX190088
sb_gmnlkfta_0006e02.t7	1		unclassified				EX190092
sb_gmnlkfta_0006e03.t7	1		snap29-prov; synaptosomal-associated protein, 29kDa; K08509 synaptosomal-associated protein, 29kDa		4e-33	56% (75/132)	EX190093
sb_gmnlkfta_0006e11.t7	1		unclassified				EX190100
sb_gmnlkfta_0006e12.t7	1		unclassified				EX190101
sb_gmnlkfta_0006e13.t7	1		unclassified				EX190102
sb_gmnlkfta_0006e14.t7	1		unclassified				EX190103
sb_gmnlkfta_0006e22.t7	1		unclassified				EX190111
sb_gmnlkfta_0006f02.t7	1		Cluster: transforming acidic coiled coil 3; n=2; Takifugu rubripes|Rep: transforming acidic coiled coil 3 - Takifugu rubripes		1e-25	67% (62/92)	EX190114

sb_gmnlkfta_0006f06.t7	1		unclassified				EX190118
sb_gmnlkfta_0006f08.t7	1		RCJMB04_10g15, CLPTM1L; CLPTM1-like		2e-40	85% (74/87)	EX190120
sb_gmnlkfta_0006f12.t7	1		unclassified				EX190122
sb_gmnlkfta_0006f14.t7	1		Cluster: Taldo1 protein; n=4; Danio rerio|Rep: Taldo1 protein - Danio rerio (Zebrafish) (Brachydanio rerio)		1e-53	81% (103/127)	EX190124
sb_gmnlkfta_0006f18.t7	1		Cluster: Calreticulin; n=3; Oncorhynchus mykiss|Rep: Calreticulin - Oncorhynchus mykiss (Rainbow trout) (Salmo gairdneri)	GO:0005509 GO:0005783 GO:0006457 GO:0051082	2e-12	89% (33/37)	EX190128
sb_gmnlkfta_0006f20.t7	1		unclassified				EX190130
sb_gmnlkfta_0006f21.t7	1		unclassified				EX190131
sb_gmnlkfta_0006f23.t7	1		unclassified				EX190133
sb_gmnlkfta_0006g02.t7	1		Rsl1; regulator of sex limited protein 1; K09228 KRAB domain-containing zinc finger protein		3e-10	41% (31/75)	EX190135
sb_gmnlkfta_0006g05.t7	1		Cluster: 40S ribosomal protein S8; n=2; Gnathostomata|Rep: 40S ribosomal protein S8 - Ictalurus punctatus (Channel catfish)	GO:0005622 GO:0030529	9e-10	78% (26/33)	EX190137
sb_gmnlkfta_0006g07.t7	1		unclassified				EX190139

sb_gmnlkfta_0006g08.t7	1		unclassified				EX190140
sb_gmnlkfta_0006g09.t7	1		Cluster: Homolog of Brachydanio rerio "Eukaryotic translation elongation factor 2, like.; n=1; Takifugu rubripes|Rep: Homolog of Brachydanio rerio "Eukaryotic translation elongation factor 2, like. - Takifugu rubripes		2e-27	53% (79/149)	EX190141
sb_gmnlkfta_0006g10.t7	1		Cluster: GTP binding protein NGB; n=3; Danio rerio|Rep: GTP binding protein NGB - Danio rerio (Zebrafish) (Brachydanio rerio)		3e-48	96% (92/95)	EX190142
sb_gmnlkfta_0006g11.t7	1		unclassified				EX190143
sb_gmnlkfta_0006g12.t7	1		Cluster: Homolog of Homo sapiens "inositol polyphosphate-5-phosphatase, 145kDa; n=1; Takifugu rubripes|Rep: Homolog of Homo sapiens "inositol polyphosphate-5-phosphatase, 145kDa - Takifugu rubripes		1e-21	71% (48/67)	EX190144
sb_gmnlkfta_0006g14.t7	1		Cluster: Cytochrome c oxidase subunit VIc; n=1; Thunnus obesus|Rep: Cytochrome c oxidase subunit VIc - Thunnus obesus (Bigeye tuna)	GO:0004129 GO:0006118	3e-15	93% (41/44)	EX190146
sb_gmnlkfta_0006g17.t7	1		Cluster: Homolog of Homo sapiens "NADH-ubiquinone oxidoreductase subunit B14.7; n=1; Takifugu rubripes|Rep: Homolog of Homo sapiens "NADH-ubiquinone oxidoreductase subunit B14.7 - Takifugu rubripes		5e-40	71% (73/102)	EX190149
sb_gmnlkfta_0006h04.t7	1		Unassigned protein		6e-13	46% (38/82)	EX190158
sb_gmnlkfta_0006h05.t7	1		Unassigned protein		3e-11	46% (27/58)	EX190159
sb_gmnlkfta_0006h06.t7	1		Cluster: Lipocalin-type prostaglandin D synthase-like protein; n=2; Danio rerio|Rep: Lipocalin-type prostaglandin D synthase-like protein - Danio rerio (Zebrafish) (Brachydanio rerio)		2e-09	59% (29/49)	EX190160

sb_gmnlkfta_0006h08.t7	1		RCJMB04_10g15, CLPTM1L; CLPTM1-like		2e-40	85% (74/87)	EX190162
sb_gmnlkfta_0006h10.t7	1		unclassified				EX190163
sb_gmnlkfta_0006h14.t7	1		Cluster: Glutathione S-transferase pi; n=2; Cyprinidae|Rep: Glutathione S-transferase pi - Brachydanio rerio (Zebrafish) (Danio rerio)		5e-29	77% (61/79)	EX190166
sb_gmnlkfta_0006h16.t7	1		Cluster: Novel protein; n=2; Danio rerio|Rep: Novel protein - Danio rerio (Zebrafish) (Brachydanio rerio)		2e-16	57% (47/82)	EX190168
sb_gmnlkfta_0006h18.t7	1		Cluster: SERPINE1 mRNA binding protein 1; n=2; Danio rerio|Rep: SERPINE1 mRNA binding protein 1 - Danio rerio (Zebrafish) (Brachydanio rerio)		1e-19	90% (45/50)	EX190170
sb_gmnlkfta_0006h22.t7	1		Cluster: GTP binding protein NGB; n=3; Danio rerio|Rep: GTP binding protein NGB - Danio rerio (Zebrafish) (Brachydanio rerio)		3e-48	96% (92/95)	EX190173
sb_gmnlkfta_0006h24.t7	1		Cluster: Chaperone protein GP96 (Tumor rejection antigen (Gp96) 1) (Heat shock protein 90kDa beta (Grp94), member 1); n=2; Clupeocephala|Rep: Chaperone protein GP96 (Tumor rejection antigen (Gp96) 1) (Heat shock protein 90kDa beta (Grp94), member 1) - Danio rerio (Zebrafish) (Brachydanio rerio)		5e-20	90% (49/54)	EX190174
sb_gmnlkfta_0006i01.t7	1		Cluster: 60S ribosomal protein L7; n=1; Neurospora crassa|Rep: 60S ribosomal protein L7 - Neurospora crassa	GO:0003735 GO:0005622 GO:0005840 GO:0006412 GO:0015934 GO:0030528 GO:0030529	2e-06	51% (28/54)	EX190175
sb_gmnlkfta_0006i02.t7	1		Mobkl2a; MOB1, Mps One Binder kinase activator-like 2A (yeast)		2e-27	91% (54/59)	EX190176
sb_gmnlkfta_0006i07.t7	1		unclassified				EX190181

sb_gmnlkfta_0006i12.t7	1		Cluster: Homolog of Homo sapiens "inositol polyphosphate-5-phosphatase, 145kDa; n=1; Takifugu rubripes|Rep: Homolog of Homo sapiens "inositol polyphosphate-5-phosphatase, 145kDa - Takifugu rubripes		1e-21	71% (48/67)	EX190185
sb_gmnlkfta_0006i16.t7	1		unclassified				EX190188
sb_gmnlkfta_0006i17.t7	1		Cluster: PREDICTED: similar to mitogen-activated protein kinase 13; n=1; Monodelphis domestica|Rep: PREDICTED: similar to mitogen-activated protein kinase 13 - Monodelphis domestica		1e-23	92% (49/53)	EX190189
sb_gmnlkfta_0006i20.t7	1		Cluster: Novel protein similar to vertebrate phospholipase A2, group IV family; n=1; Danio rerio|Rep: Novel protein similar to vertebrate phospholipase A2, group IV family - Danio rerio (Zebrafish) (Brachydanio rerio)		8e-18	65% (45/69)	EX190191
sb_gmnlkfta_0006i23.t7	1		Cluster: Interferon regulatory factor 1 type 2; n=2; Scophthalmus maximus|Rep: Interferon regulatory factor 1 type 2 - Scophthalmus maximus (Turbot)	GO:0003700 GO:0005634 GO:0006355	1e-50	56% (97/171)	EX190194
sb_gmnlkfta_0006i24.t7	1		Cluster: NOP56; n=5; Danio rerio|Rep: NOP56 - Danio rerio (Zebrafish) (Brachydanio rerio)		2e-99	85% (184/214)	EX190195
sb_gmnlkfta_0006j01.t7	1		unclassified				EX190196
sb_gmnlkfta_0006j02.t7	1		unclassified				EX190197
sb_gmnlkfta_0006j03.t7	1		COX8 domain containing protein	GO:0004129 GO:0006118	6e-06	48% (14/29)	EX190198
sb_gmnlkfta_0006j04.t7	1		Cluster: Extracellular signal-regulated kinase 3; n=1; Danio rerio|Rep: Extracellular signal-regulated kinase 3 - Danio rerio (Zebrafish) (Brachydanio rerio)		1e-06	96% (24/25)	EX190199
sb_gmnlkfta_0006j06.t7	1		unclassified				EX190201

sb_gmnlkfta_0006j07.t7	1		Cluster: Novel protein similar to human oligophrenin 1; n=1; Danio rerio|Rep: Novel protein similar to human oligophrenin 1 - Danio rerio (Zebrafish) (Brachydanio rerio)		1e-100	81% (147/180)	EX190202
sb_gmnlkfta_0006j08.t7	1		unclassified				EX190203
sb_gmnlkfta_0006j10.t7	1		Cluster: PREDICTED: similar to Proteasome subunit alpha type 4 (Proteasome component C9) (Macropain subunit C9) (Multicatalytic endopeptidase complex subunit C9) (Proteasome subunit L); n=1; Monodelphis domestica|Rep: PREDICTED: similar to Proteasome subunit alpha type 4 (Proteasome component C9) (Macropain subunit C9) (Multicatalytic endopeptidase complex subunit C9) (Proteasome subunit L) - Monodelphis domestica		3e-13	94% (34/36)	EX190205
sb_gmnlkfta_0006j19.t7	1		unclassified				EX190213
sb_gmnlkfta_0006j21.t7	1		Cluster: Novel protein; n=3; Danio rerio|Rep: Novel protein - Danio rerio (Zebrafish) (Brachydanio rerio)		2e-23	50% (54/108)	EX190215
sb_gmnlkfta_0006j23.t7	1		unclassified				EX190217
sb_gmnlkfta_0006j24.t7	1		Cluster: T-complex protein 1 subunit alpha; n=9; Euteleostomi|Rep: T-complex protein 1 subunit alpha - Ambystoma mexicanum (Axolotl)	GO:0000166 GO:0005515 GO:0005524 GO:0006457 GO:0044267 GO:0051082	2e-15	84% (42/50)	EX190218
sb_gmnlkfta_0006k01.t7	1		unclassified				EX190219
sb_gmnlkfta_0006k03.t7	1		unclassified				EX190220
sb_gmnlkfta_0006k08.t7	1		Cluster: Serine/threonine-protein phosphatase 2A catalytic subunit alpha isoform; n=63; Bilateria|Rep: Serine/threonine-protein phosphatase 2A catalytic subunit alpha isoform - Homo sapiens		2e-53	69% (107/153)	EX190223

			(Human)				
sb_gmnlkfta_0006k09.t7	1		unclassified				EX190224
sb_gmnlkfta_0006k14.t7	1		unclassified				EX190228
sb_gmnlkfta_0006k20.t7	1		Cluster: Novel protein similar to vertebrate phospholipase A2, group IV family; n=1; Danio rerio|Rep: Novel protein similar to vertebrate phospholipase A2, group IV family - Danio rerio (Zebrafish) (Brachydanio rerio)		8e-18	65% (45/69)	EX190233
sb_gmnlkfta_0006l02.t7	1		unclassified				EX190237
sb_gmnlkfta_0006l03.t7	1		unclassified				EX190238
sb_gmnlkfta_0006l04.t7	1		Cluster: Homolog of Homo sapiens "PREDICTED "similar to immunoglobulin heavy-chain-2 light-chain-2 VH segment; n=1; Takifugu rubripes|Rep: Homolog of Homo sapiens "PREDICTED "similar to immunoglobulin heavy-chain-2 light-chain-2 VH segment - Takifugu rubripes		3e-20	63% (41/65)	EX190239
sb_gmnlkfta_0006l05.t7	1		unclassified				EX190240
sb_gmnlkfta_0006l09.t7	1		unclassified				EX190244
sb_gmnlkfta_0006l11.t7	1		unclassified				EX190246
sb_gmnlkfta_0006l15.t7	1		unclassified				EX190250

sb_gmnlkfta_0006l17.t7	1		Cluster: Ribosomal protein L23; n=1; Siniperca chuatsi|Rep: Ribosomal protein L23 - Siniperca chuatsi (Chinese perch)	GO:0003735<br>GO:0005622<br>GO:0005840<br>GO:0006412	5e-12	80% (38/47)	EX190252
sb_gmnlkfta_0006l18.t7	1		Cluster: Homolog of Brachydanio rerio "Brain glycogen phosphorylase Pygb.; n=1; Takifugu rubripes|Rep: Homolog of Brachydanio rerio "Brain glycogen phosphorylase Pygb. - Takifugu rubripes		2e-45	82% (87/105)	EX190253
sb_gmnlkfta_0006l20.t7	1		unclassified				EX190254
sb_gmnlkfta_0006l21.t7	1		Cluster: Novel protein; n=5; Danio rerio|Rep: Novel protein - Danio rerio (Zebrafish) (Brachydanio rerio)		7e-05	56% (21/37)	EX190255
sb_gmnlkfta_0006l22.t7	1		unclassified				EX190256
sb_gmnlkfta_0006m02.t7	1		unclassified				EX190259
sb_gmnlkfta_0006m10.t7	1		Cluster: Proteasome subunit alpha type-7; n=11; Euteleostomi|Rep: Proteasome subunit alpha type-7 - Carassius auratus (Goldfish)	GO:0004175<br>GO:0004298<br>GO:0005634<br>GO:0005737<br>GO:0005829<br>GO:0005839<br>GO:0006511<br>GO:0008233<br>GO:0016787<br>GO:0043234	4e-62	100% (78/78)	EX190265
sb_gmnlkfta_0006m11.t7	1		Cluster: Riboflavin kinase; n=7; Eutheria|Rep: Riboflavin kinase - Homo sapiens (Human)		2e-50	66% (98/148)	EX190266
sb_gmnlkfta_0006m12.t7	1		unclassified				EX190267
sb_gmnlkfta_0006m14.t7	1		Cluster: GTP binding protein NGB; n=3; Danio rerio|Rep: GTP binding protein NGB - Danio rerio (Zebrafish) (Brachydanio rerio)		3e-48	96% (92/95)	EX190269

sb_gmnlkfta_0006m21.t7	1		unclassified				EX190274
sb_gmnlkfta_0006m22.t7	1		SSU rRNA; Oncorhynchus kisutch		1e-42	96% (95/98)	EX190275
sb_gmnlkfta_0006m24.t7	1		Cluster: Ribosomal protein L21; n=7; Euteleostomi|Rep: Ribosomal protein L21 - Danio rerio (Zebrafish) (Brachydanio rerio)		1e-34	83% (68/81)	EX190277
sb_gmnlkfta_0006n02.t7	1		unclassified				EX190278
sb_gmnlkfta_0006n04.t7	1		unclassified				EX190279
sb_gmnlkfta_0006n06.t7	1		Cluster: Protein phosphatase 2 (Formerly 2A), regulatory subunit B (PR 52), beta isoform; n=2; Danio rerio|Rep: Protein phosphatase 2 (Formerly 2A), regulatory subunit B (PR 52), beta isoform - Danio rerio (Zebrafish) (Brachydanio rerio)		6e-05	70% (21/30)	EX190281
sb_gmnlkfta_0006n08.t7	1		Cluster: Homolog of Brachydanio rerio "Glutamate-cysteine ligase, catalytic subunit.; n=1; Takifugu rubripes|Rep: Homolog of Brachydanio rerio "Glutamate-cysteine ligase, catalytic subunit. - Takifugu rubripes		1e-36	93% (71/76)	EX190283
sb_gmnlkfta_0006n12.t7	1		unclassified				EX190286
sb_gmnlkfta_0006n13.t7	1		Cluster: Toll-like-receptor; n=2; Oncorhynchus mykiss|Rep: Toll-like-receptor - Oncorhynchus mykiss (Rainbow trout) (Salmo gairdneri)	GO:0004872 GO:0004888 GO:0005515 GO:0016020	3e-26	36% (80/221)	EX190287
sb_gmnlkfta_0006n15.t7	1		Cluster: NADH dehydrogenase (Ubiquinone) Fe-S protein 6; n=2; Danio rerio|Rep: NADH dehydrogenase (Ubiquinone) Fe-S protein 6 - Danio rerio (Zebrafish) (Brachydanio rerio)		2e-45	79% (86/108)	EX190289
sb_gmnlkfta_0006n16.t7	1		unclassified				EX190290

sb_gmnlkfta_0006n19.t7	1		Cluster: Bactericidal permeability increasing protein/lipopolysaccharide binding protein variant b; n=2; Gadus morhua|Rep: Bactericidal permeability increasing protein/lipopolysaccharide binding protein variant b - Gadus morhua (Atlantic cod)	GO:0008289	8e-44	85% (91/107)	EX190293
sb_gmnlkfta_0006n21.t7	1		SSU72; SSU72 RNA polymerase II CTD phosphatase homolog (S. cerevisiae)		1e-19	93% (46/49)	EX190295
sb_gmnlkfta_0006n23.t7	1		unclassified				EX190297
sb_gmnlkfta_0006n24.t7	1		unclassified				EX190298
sb_gmnlkfta_0006o01.t7	1		unclassified				EX190299
sb_gmnlkfta_0006o02.t7	1		unclassified				EX190300
sb_gmnlkfta_0006o04.t7	1		unclassified				EX190302
sb_gmnlkfta_0006o06.t7	1		Cluster: Transcribed locus, weakly similar to NP_004137.2 NADH dehydrogenase (ubiquinone) 1 beta subcomplex, 7, 18kDa [Homo sapiens]; n=1; Takifugu rubripes|Rep: Transcribed locus, weakly similar to NP_004137.2 NADH dehydrogenase (ubiquinone) 1 beta subcomplex, 7, 18kDa [Homo sapiens] - Takifugu rubripes		9e-43	76% (72/94)	EX190304
sb_gmnlkfta_0006o07.t7	1		Cluster: PREDICTED: similar to Guanine nucleotide-binding protein G(k), alpha subunit (G(i) alpha-3); n=1; Canis lupus familiaris|Rep: PREDICTED: similar to Guanine nucleotide-binding protein G(k), alpha subunit (G(i) alpha-3) - Canis familiaris		9e-20	95% (46/48)	EX190305
sb_gmnlkfta_0006o10.t7	1		unclassified				EX190308

sb_gmnlkfta_0006o17.t7	1		Cluster: Transglutaminase; n=1; Oreochromis niloticus|Rep: Transglutaminase - Oreochromis niloticus (Nile tilapia) (Tilapia nilotica)	GO:0003810 GO:0018149	8e-13	50% (41/81)	EX190315
sb_gmnlkfta_0006o22.t7	1		Unassigned protein		7e-07	48% (30/62)	EX190318
sb_gmnlkfta_0006p02.t7	1		unclassified				EX190320
sb_gmnlkfta_0006p03.t7	1		Cluster: MHC class I precursor; n=3; Gadus morhua|Rep: MHC class I precursor - Gadus morhua (Atlantic cod)	GO:0006955 GO:0016020 GO:0019882 GO:0042612	2e-19	94% (47/50)	EX190321
sb_gmnlkfta_0006p04.t7	1		unclassified				EX190322
sb_gmnlkfta_0006p06.t7	1		unclassified				EX190324
sb_gmnlkfta_0006p08.t7	1		LSU rRNA; Hydrolagus colliei		1e-171	97% (322/329)	EX190325
sb_gmnlkfta_0006p09.t7	1		Pros45 [EC:3.4.25.1]; K03066 26S proteasome regulatory subunit T6		5e-14	92% (38/41)	EX190326
sb_gmnlkfta_0006p10.t7	1		Cluster: PREDICTED: similar to Sulfide:quinone oxidoreductase, mitochondrial precursor; n=3; Eutheria|Rep: PREDICTED: similar to Sulfide:quinone oxidoreductase, mitochondrial precursor - Rattus norvegicus		6e-36	66% (68/103)	EX190327
sb_gmnlkfta_0006p12.t7	1		unclassified				EX190329
sb_gmnlkfta_0006p14.t7	1		Cluster: Glutathione S-transferase pi; n=2; Cyprinidae|Rep: Glutathione S-transferase pi - Brachydanio rerio (Zebrafish) (Danio rerio)		5e-29	77% (61/79)	EX190331

sb_gmnlkfta_0006p16.t7	1		unclassified				EX190333
sb_gmnlkfta_0006p17.t7	1		unclassified				EX190334
sb_gmnlkfta_0006p19.t7	1		Cluster: Homolog of Brachydanio rerio "Heat shock protein 90-alpha.; n=1; Takifugu rubripes|Rep: Homolog of Brachydanio rerio "Heat shock protein 90-alpha. - Takifugu rubripes		3e-81	89% (150/167)	EX190336
sb_gmnlkfta_0006p21.t7	1		Cluster: Ferritin, heavy subunit; n=4; Euteleostei|Rep: Ferritin, heavy subunit - Salmo salar (Atlantic salmon)	GO:0004322 GO:0005488 GO:0005506 GO:0006826 GO:0006879 GO:0008199 GO:0016491 GO:0046872 GO:0046914	4e-23	70% (40/57)	EX190338
sb_gmnlkfta_0006p23.t7	1		unclassified				EX190339
sb_gmnlkfta_0006p24.t7	1		unclassified				EX190340
sb_gmnlkfta_0007a02.t7	1		unclassified				EY973452
sb_gmnlkfta_0007a03.t7	1		unclassified				EY973453
sb_gmnlkfta_0007a10.t7	1		unclassified				EY973454
sb_gmnlkfta_0007a12.t7	1		Cluster: Zgc:56011; n=2; Danio rerio|Rep: Zgc:56011 - Danio rerio (Zebrafish) (Brachydanio rerio)		6e-08	77% (27/35)	

sb_gmnlkfta_0007a13.t7	1		unclassified				EY973456
sb_gmnlkfta_0007a15.t7	1		unclassified				EY973457
sb_gmnlkfta_0007a18.t7	1		Cluster: PREDICTED: similar to predicted protein; n=2; Danio rerio|Rep: PREDICTED: similar to predicted protein - Danio rerio		3e-05	54% (24/44)	
sb_gmnlkfta_0007b06.t7	1		unclassified				EY973460
sb_gmnlkfta_0007b07.t7	1		Cluster: Bridging integrator 3; n=5; Eutheria|Rep: Bridging integrator 3 - Mus musculus (Mouse)		3e-29	78% (63/80)	EY973461
sb_gmnlkfta_0007b11.t7	1		unclassified				EY973463
sb_gmnlkfta_0007b14.t7	1		Cluster: Hemoglobin subunit alpha-1; n=3; Gadidae|Rep: Hemoglobin subunit alpha-1 - Gadus morhua (Atlantic cod)	GO:0005344 GO:0005506 GO:0005833 GO:0006810 GO:0015671 GO:0019825 GO:0020037	2e-14	88% (39/44)	
sb_gmnlkfta_0007b17.t7	1		unclassified				EY973465
sb_gmnlkfta_0007b21.t7	1		unclassified				
sb_gmnlkfta_0007b22.t7	1		Cluster: PREDICTED: similar to ATP-binding cassette sub-family E member 1 (RNase L inhibitor) (Ribonuclease 4 inhibitor) (RNS4I) isoform 4; n=1; Canis lupus familiaris|Rep: PREDICTED: similar to ATP-binding cassette sub-family E member 1 (RNase L inhibitor) (Ribonuclease 4 inhibitor) (RNS4I) isoform 4 - Canis familiaris		6e-21	88% (48/54)	EY973467

sb_gmnlkfta_0007b23.t7	1		unclassified				
sb_gmnlkfta_0007b24.t7	1		unclassified				
sb_gmnlkfta_0007c01.t7	1		unclassified				EY973468
sb_gmnlkfta_0007c02.t7	1		unclassified				EY973469
sb_gmnlkfta_0007c03.t7	1		HSP90 domain containing protein		3e-07	58% (17/29)	EY973470
sb_gmnlkfta_0007c05.t7	1		unclassified				
sb_gmnlkfta_0007c06.t7	1		unclassified				EY973471
sb_gmnlkfta_0007c09.t7	1		unclassified				EY973474
sb_gmnlkfta_0007c10.t7	1		unclassified				EY973475
sb_gmnlkfta_0007c13.t7	1		unclassified				EY973477
sb_gmnlkfta_0007c16.t7	1		unclassified				EY973479
sb_gmnlkfta_0007c17.t7	1		unclassified				EY973480

sb_gmnlkfta_0007c19.t7	1		Cluster: PREDICTED: similar to alpha-cardiac actin; n=1; Ornithorhynchus anatinus|Rep: PREDICTED: similar to alpha-cardiac actin - Ornithorhynchus anatinus		9e-09	80% (29/36)	EY973482
sb_gmnlkfta_0007c20.t7	1		unclassified				EY973483
sb_gmnlkfta_0007d01.t7	1		unclassified				EY973487
sb_gmnlkfta_0007d02.t7	1		Cluster: PREDICTED: similar to RAB11B, member RAS oncogene family; n=1; Canis lupus familiaris|Rep: PREDICTED: similar to RAB11B, member RAS oncogene family - Canis familiaris		5e-11	84% (21/25)	EY973488
sb_gmnlkfta_0007d03.t7	1		unclassified				EY973489
sb_gmnlkfta_0007d04.t7	1		Cluster: RING finger protein 145; n=2; Danio rerio|Rep: RING finger protein 145 - Danio rerio (Zebrafish) (Brachydanio rerio)		1e-04	59% (28/47)	EY973490
sb_gmnlkfta_0007d05.t7	1		unclassified				EY973491
sb_gmnlkfta_0007d09.t7	1		LSU rRNA; Squalus acanthias		3e-50	98% (107/109)	EY973493
sb_gmnlkfta_0007d12.t7	1		Cluster: Elongation factor 1-gamma; n=6; Clupeocephala|Rep: Elongation factor 1-gamma - Danio rerio (Zebrafish) (Brachydanio rerio)		2e-05	91% (22/24)	
sb_gmnlkfta_0007d14.t7	1		unclassified				EY973496
sb_gmnlkfta_0007d16.t7	1		Cluster: NADH dehydrogenase (Ubiquinone) 1 alpha subcomplex 4, like; n=1; Danio rerio|Rep: NADH dehydrogenase (Ubiquinone) 1 alpha subcomplex 4, like - Danio rerio (Zebrafish) (Brachydanio		1e-10	77% (28/36)	EY973498

			rerio)				
sb_gmnlkfta_0007d17.t7	1		unclassified				EY973499
sb_gmnlkfta_0007d18.t7	1		unclassified				
sb_gmnlkfta_0007d19.t7	1		unclassified				EY973500
sb_gmnlkfta_0007d21.t7	1		TRAP-delta domain containing protein	GO:0005783	2e-06	54% (20/37)	
sb_gmnlkfta_0007d22.t7	1		pbef1; pre-B-cell colony enhancing factor 1		1e-06	88% (22/25)	EY973502
sb_gmnlkfta_0007d23.t7	1		unclassified				EY973503
sb_gmnlkfta_0007d24.t7	1		unclassified				EY973504
sb_gmnlkfta_0007e01.t7	1		unclassified				EY973505
sb_gmnlkfta_0007e02.t7	1		unclassified				EY973506
sb_gmnlkfta_0007e04.t7	1		Unassigned protein				EY973507
sb_gmnlkfta_0007e09.t7	1		unclassified				EY973511

sb_gmnlkfta_0007e10.t7	1		Unassigned protein				EY973512
sb_gmnlkfta_0007e11.t7	1		Cluster: 40S ribosomal protein S3a; n=2; Percomorpha|Rep: 40S ribosomal protein S3a - Siniperca chuatsi (Chinese perch)	GO:0003735 GO:0005622 GO:0005840 GO:0006412 GO:0030529	1e-07	86% (25/29)	EY973513
sb_gmnlkfta_0007e13.t7	1		unclassified				EY973514
sb_gmnlkfta_0007e16.t7	1		unclassified				EY973516
sb_gmnlkfta_0007e19.t7	1		unclassified				EY973518
sb_gmnlkfta_0007e21.t7	1		unclassified				EY973519
sb_gmnlkfta_0007e24.t7	1		unclassified				EY973522
sb_gmnlkfta_0007f01.t7	1		unclassified				EY973523
sb_gmnlkfta_0007f02.t7	1		Cluster: High mobility group protein; n=3; Percomorpha|Rep: High mobility group protein - Pagrus major (Red sea bream) (Chrysophrys major)	GO:0000785 GO:0003677 GO:0005634 GO:0006355	1e-24	89% (52/58)	EY973524
sb_gmnlkfta_0007f03.t7	1		RPS19BP1, S19BP; ribosomal protein S19 binding protein 1		1e-09	37% (27/72)	EY973525
sb_gmnlkfta_0007f09.t7	1		unclassified				EY973530

sb_gmnlkfta_0007f15.t7	1		Cluster: RBM39 protein; n=3; Eutheria|Rep: RBM39 protein - Homo sapiens (Human)		3e-42	50% (93/186)	EY973534
sb_gmnlkfta_0007f20.t7	1		unclassified				EY973537
sb_gmnlkfta_0007g02.t7	1		Cluster: ADP,ATP translocase; n=2; Pleuronectoidei|Rep: ADP,ATP translocase - Platichthys flesus (European flounder)	GO:0005215 GO:0005488 GO:0005743 GO:0006810 GO:0016020 GO:0016021	8e-10	96% (31/32)	EY973540
sb_gmnlkfta_0007g04.t7	1		Gal-bind_lectin domain containing protein		1e-05	29% (17/57)	EY973542
sb_gmnlkfta_0007g05.t7	1		unclassified				EY973543
sb_gmnlkfta_0007g08.t7	1		Cluster: Homolog of Melanotaenia fluviatilis "DEAD box RNA helicase Vasa.; n=1; Takifugu rubripes|Rep: Homolog of Melanotaenia fluviatilis "DEAD box RNA helicase Vasa. - Takifugu rubripes		3e-30	83% (66/79)	EY973545
sb_gmnlkfta_0007g12.t7	1		Cluster: PREDICTED: similar to vacuolar protein sorting 35; n=1; Macaca mulatta|Rep: PREDICTED: similar to vacuolar protein sorting 35 - Macaca mulatta		5e-42	94% (85/90)	EY973547
sb_gmnlkfta_0007g19.t7	1		unclassified				EY973552
sb_gmnlkfta_0007g20.t7	1		Cluster: Vacuolar-sorting protein SNF8; n=5; Clupeocephala|Rep: Vacuolar-sorting protein SNF8 - Danio rerio (Zebrafish) (Brachydanio rerio)		8e-22	90% (48/53)	EY973553
sb_gmnlkfta_0007g22.t7	1		unclassified				EY973554

sb_gmnlkfta_0007g24.t7	1		Cluster: Dap3 protein; n=1; Danio rerio|Rep: Dap3 protein - Danio rerio (Zebrafish) (Brachydanio rerio)		2e-05	84% (22/26)	EY973555
sb_gmnlkfta_0007h01.t7	1		unclassified				EY973556
sb_gmnlkfta_0007h02.t7	1		unclassified				
sb_gmnlkfta_0007h05.t7	1		unclassified				EY973558
sb_gmnlkfta_0007h07.t7	1		ZNF593; zinc finger protein 593	GO:0003676 GO:0005622 GO:0008270	3e-35	64% (68/105)	EY973560
sb_gmnlkfta_0007h09.t7	1		unclassified				EY973562
sb_gmnlkfta_0007h11.t7	1		unclassified				
sb_gmnlkfta_0007h12.t7	1		unclassified				
sb_gmnlkfta_0007h15.t7	1		Cluster: PREDICTED: similar to ATP-binding cassette sub-family E member 1 (RNase L inhibitor) (Ribonuclease 4 inhibitor) (RNS4I) isoform 4; n=1; Canis lupus familiaris|Rep: PREDICTED: similar to ATP-binding cassette sub-family E member 1 (RNase L inhibitor) (Ribonuclease 4 inhibitor) (RNS4I) isoform 4 - Canis familiaris		6e-38	87% (78/89)	EY973565
sb_gmnlkfta_0007h16.t7	1		unclassified				EY973566
sb_gmnlkfta_0007h18.t7	1		Cluster: Homolog of Brachydanio rerio "Similar to upstream transcription factor 1.; n=1; Takifugu rubripes|Rep: Homolog of Brachydanio rerio "Similar to upstream transcription factor 1. - Takifugu rubripes		5e-28	78% (60/76)	EY973568

sb_gmnlkfta_0007h19.t7	1		unclassified				
sb_gmnlkfta_0007h20.t7	1		unclassified				EY973569
sb_gmnlkfta_0007h22.t7	1		unclassified				EY973570
sb_gmnlkfta_0007h24.t7	1		Cluster: 14-3-3 protein epsilon; n=18; Tetrapoda|Rep: 14-3-3 protein epsilon - Homo sapiens (Human)		1e-13	92% (38/41)	EY973572
sb_gmnlkfta_0007i04.t7	1		LOC701834; similar to THAP domain containing 6		4e-12	35% (33/92)	EY973575
sb_gmnlkfta_0007i07.t7	1		RCJMB04_17p9, DKC1; dyskeratosis congenita 1, dyskerin [EC:5.4.99.-]; K01855		4e-07	68% (26/38)	EY973578
sb_gmnlkfta_0007i08.t7	1		unclassified				EY973579
sb_gmnlkfta_0007i10.t7	1		unclassified				EY973581
sb_gmnlkfta_0007i13.t7	1		LSU rRNA; Squalus acanthias		2e-70	98% (137/139)	
sb_gmnlkfta_0007i15.t7	1		unclassified				EY973585
sb_gmnlkfta_0007i16.t7	1		unclassified				EY973586
sb_gmnlkfta_0007i19.t7	1		unclassified				

sb_gmnlkfta_0007i20.t7	1		unclassified				EY973588
sb_gmnlkfta_0007i22.t7	1		unclassified				EY973589
sb_gmnlkfta_0007j01.t7	1		unclassified				EY973590
sb_gmnlkfta_0007j02.t7	1		LOC100027726; similar to MRNA turnover 4 homolog (S. cerevisiae)		2e-34	83% (70/84)	EY973591
sb_gmnlkfta_0007j03.t7	1		unclassified				EY973592
sb_gmnlkfta_0007j04.t7	1		Cluster: Eukaryotic translation initiation factor 4E; n=5; Xenopus|Rep: Eukaryotic translation initiation factor 4E - Xenopus laevis (African clawed frog)	GO:0003723 GO:0003743 GO:0005737 GO:0006412 GO:0006413 GO:0006417	8e-45	80% (81/101)	EY973593
sb_gmnlkfta_0007j05.t7	1		Cluster: Putative flavin-monooxygenase; n=1; Takifugu rubripes|Rep: Putative flavin-monooxygenase - Fugu rubripes (Japanese pufferfish) (Takifugu rubripes)	GO:0004497 GO:0004499 GO:0005792 GO:0006118 GO:0016491 GO:0031227 GO:0050660 GO:0050661	2e-51	77% (95/123)	EY973594
sb_gmnlkfta_0007j06.t7	1		unclassified				EY973595
sb_gmnlkfta_0007j12.t7	1		Cluster: Ribosomal protein L37; n=4; Euteleostomi|Rep: Ribosomal protein L37 - Tetraodon nigroviridis (Green puffer)	GO:0003723 GO:0003735 GO:0005622 GO:0005840 GO:0006412 GO:0008270 GO:0019843 GO:0030529	9e-11	96% (29/30)	EY973600

				GO:0046872			
sb_gmnlkfta_0007j13.t7	1		Cluster: PREDICTED: similar to elongation factor-2; n=1; Pan troglodytes|Rep: PREDICTED: similar to elongation factor-2 - Pan troglodytes		6e-09	65% (27/41)	EY973601
sb_gmnlkfta_0007j15.t7	1		unclassified				EY973603
sb_gmnlkfta_0007j16.t7	1		unclassified				EY973604
sb_gmnlkfta_0007j17.t7	1		unclassified				EY973605
sb_gmnlkfta_0007j19.t7	1		unclassified				EY973607
sb_gmnlkfta_0007j20.t7	1		Cluster: Macrophage migration inhibitory factor; n=1; Danio rerio|Rep: Macrophage migration inhibitory factor - Danio rerio (Zebrafish) (Brachydanio rerio)		1e-27	73% (63/86)	EY973608
sb_gmnlkfta_0007j23.t7	1		unclassified				
sb_gmnlkfta_0007j24.t7	1		Cluster: RAD21 homolog; n=4; Danio rerio|Rep: RAD21 homolog - Danio rerio (Zebrafish) (Brachydanio rerio)		5e-07	88% (22/25)	EY973609
sb_gmnlkfta_0007k02.t7	1		unclassified				EY973611
sb_gmnlkfta_0007k03.t7	1		unclassified				EY973612
sb_gmnlkfta_0007k04.t7	1		Cluster: UPI0000D8D2B5 related cluster; n=1; Danio rerio|Rep: UPI0000D8D2B5 UniRef100 entry - Danio rerio		1e-07	50% (28/55)	EY973613

sb_gmnlkfta_0007k06.t7	1		unclassified				EY973614
sb_gmnlkfta_0007k07.t7	1		Cluster: Homolog of Homo sapiens "Inter-alpha (globulIn) InhIbItor H3; n=1; Takifugu rubripes|Rep: Homolog of Homo sapiens "Inter-alpha (globulIn) InhIbItor H3 - Takifugu rubripes		4e-53	65% (95/144)	EY973615
sb_gmnlkfta_0007k08.t7	1		unclassified				
sb_gmnlkfta_0007k09.t7	1		Cluster: Zgc:103624; n=5; Danio rerio|Rep: Zgc:103624 - Danio rerio (Zebrafish) (Brachydanio rerio)		2e-12	92% (35/38)	EY973616
sb_gmnlkfta_0007k10.t7	1		unclassified				EY973617
sb_gmnlkfta_0007k11.t7	1		unclassified				EY973618
sb_gmnlkfta_0007k12.t7	1		Cluster: 26S proteasome non-ATPase regulatory subunit 14; n=19; Coelomata|Rep: 26S proteasome non-ATPase regulatory subunit 14 - Homo sapiens (Human)		4e-29	100% (65/65)	EY973619
sb_gmnlkfta_0007k14.t7	1		Cluster: Transmembrane protein 4; n=3; Danio rerio|Rep: Transmembrane protein 4 - Danio rerio (Zebrafish) (Brachydanio rerio)		1e-50	66% (95/143)	EY973621
sb_gmnlkfta_0007k18.t7	1		Cluster: UPI0000D8D559 related cluster; n=5; Danio rerio|Rep: UPI0000D8D559 UniRef100 entry - Danio rerio		1e-08	54% (23/42)	EY973624
sb_gmnlkfta_0007k24.t7	1		unclassified				EY973627
sb_gmnlkfta_0007l01.t7	1		Cluster: Transmembrane phosphatase with tensin homology; n=2; Danio rerio|Rep: Transmembrane phosphatase with tensin homology - Danio rerio (Zebrafish) (Brachydanio rerio)		1e-08	54% (27/50)	EY973628

sb_gmnlkfta_0007l03.t7	1		unclassified				EY973630
sb_gmnlkfta_0007l04.t7	1		Cluster: Eukaryotic initiation factor 4A-II; n=21; Theria|Rep: Eukaryotic initiation factor 4A-II - Homo sapiens (Human)		9e-21	87% (28/32)	EY973631
sb_gmnlkfta_0007l07.t7	1		unclassified				EY973633
sb_gmnlkfta_0007l08.t7	1		Cluster: Thioredoxin domain containing 9; n=1; Danio rerio|Rep: Thioredoxin domain containing 9 - Danio rerio (Zebrafish) (Brachydanio rerio)		2e-60	80% (99/123)	EY973634
sb_gmnlkfta_0007l09.t7	1		Cluster: Novel protein containing a short chain dehydrogenase domain; n=4; Danio rerio|Rep: Novel protein containing a short chain dehydrogenase domain - Danio rerio (Zebrafish) (Brachydanio rerio)		1e-17	91% (43/47)	EY973635
sb_gmnlkfta_0007l10.t7	1		unclassified				EY973636
sb_gmnlkfta_0007l12.t7	1		unclassified				EY973637
sb_gmnlkfta_0007l13.t7	1		Cluster: PREDICTED: similar to HSPC005; n=1; Equus caballus|Rep: PREDICTED: similar to HSPC005 - Equus caballus		4e-09	100% (28/28)	EY973638
sb_gmnlkfta_0007l16.t7	1		Cluster: Chaperonin containing TCP1, subunit 5; n=4; Clupeocephala|Rep: Chaperonin containing TCP1, subunit 5 - Danio rerio (Zebrafish) (Brachydanio rerio)		7e-59	89% (111/124)	EY973640
sb_gmnlkfta_0007l17.t7	1		unclassified				EY973641
sb_gmnlkfta_0007l18.t7	1		Cluster: MHC class Ia antigen; n=2; Gadus morhua|Rep: MHC class Ia antigen - Gadus morhua (Atlantic cod)	GO:0006955 GO:0016020 GO:0019882 GO:0042612	3e-13	85% (35/41)	EY973642

sb_gmnlkfta_0007l19.t7	1		unclassified				EY973643
sb_gmnlkfta_0007l21.t7	1		unclassified				
sb_gmnlkfta_0007l22.t7	1		unclassified				
sb_gmnlkfta_0007l23.t7	1		unclassified				
sb_gmnlkfta_0007l24.t7	1		Cluster: PREDICTED: similar to 40S ribosomal protein S12; n=1; Pan troglodytes|Rep: PREDICTED: similar to 40S ribosomal protein S12 - Pan troglodytes		3e-08	96% (30/31)	EY973644
sb_gmnlkfta_0007m01.t7	1		unclassified				
sb_gmnlkfta_0007m02.t7	1		unclassified				EY973645
sb_gmnlkfta_0007m03.t7	1		Unassigned protein				EY973646
sb_gmnlkfta_0007m04.t7	1		unclassified				EY973647
sb_gmnlkfta_0007m07.t7	1		unclassified				
sb_gmnlkfta_0007m09.t7	1		Cluster: Homolog of Homo sapiens "N-myc and STAT interactor; n=1; Takifugu rubripes|Rep: Homolog of Homo sapiens "N-myc and STAT interactor - Takifugu rubripes		2e-11	43% (38/87)	EY973649

sb_gmnlkfta_0007m11.t7	1		MGC89675; MGC89675 protein; K02885 large subunit ribosomal protein L19e		2e-22	100% (50/50)	EY973650
sb_gmnlkfta_0007m13.t7	1		Cluster: Zgc:110598; n=3; Danio rerio|Rep: Zgc:110598 - Danio rerio (Zebrafish) (Brachydanio rerio)		3e-52	87% (98/112)	EY973651
sb_gmnlkfta_0007m14.t7	1		unclassified				
sb_gmnlkfta_0007m16.t7	1		unclassified				
sb_gmnlkfta_0007m19.t7	1		unclassified				EY973655
sb_gmnlkfta_0007m20.t7	1		Cluster: AP-3 complex subunit sigma-1; n=27; Euteleostomi|Rep: AP-3 complex subunit sigma-1 - Homo sapiens (Human)		2e-35	93% (70/75)	EY973656
sb_gmnlkfta_0007m24.t7	1		unclassified				EY973657
sb_gmnlkfta_0007n02.t7	1		unclassified				EY973658
sb_gmnlkfta_0007n03.t7	1		Cluster: Hemoglobin subunit beta; n=3; Gadidae|Rep: Hemoglobin subunit beta - Gadus morhua (Atlantic cod)	GO:0005344 GO:0005506 GO:0005833 GO:0006810 GO:0015671 GO:0019825 GO:0020037	7e-06	100% (24/24)	
sb_gmnlkfta_0007n04.t7	1		Cluster: PREDICTED: similar to predicted protein; n=2; Danio rerio|Rep: PREDICTED: similar to predicted protein - Danio rerio		1e-07	47% (37/78)	EY973659
sb_gmnlkfta_0007n05.t7	1		unclassified				EY973660

sb_gmnlkfta_0007n06.t7	1		unclassified				EY973661
sb_gmnlkfta_0007n10.t7	1		Cluster: MHC class Ia antigen; n=5; Gadus morhua|Rep: MHC class Ia antigen - Gadus morhua (Atlantic cod)	GO:0006955 GO:0016020 GO:0019882 GO:0042612	2e-60	92% (110/119)	EY973663
sb_gmnlkfta_0007n11.t7	1		unclassified				EY973664
sb_gmnlkfta_0007n14.t7	1		unclassified				EY973667
sb_gmnlkfta_0007n15.t7	1		unclassified				EY973668
sb_gmnlkfta_0007n16.t7	1		unclassified				EY973669
sb_gmnlkfta_0007n18.t7	1		unclassified				EY973671
sb_gmnlkfta_0007n19.t7	1		unclassified				EY973672
sb_gmnlkfta_0007n20.t7	1		unclassified				EY973673
sb_gmnlkfta_0007n21.t7	1		unclassified				
sb_gmnlkfta_0007o06.t7	1		unclassified				EY973679

sb_gmnlkfta_0007o13.t7	1		unclassified				EY973680
sb_gmnlkfta_0007o14.t7	1		unclassified				
sb_gmnlkfta_0007o16.t7	1		unclassified				
sb_gmnlkfta_0007o19.t7	1		unclassified				EY973682
sb_gmnlkfta_0007o24.t7	1		unclassified				EY973683
sb_gmnlkfta_0007p09.t7	1		unclassified				EY973686
sb_gmnlkfta_0007p10.t7	1		unclassified				EY973687
sb_gmnlkfta_0007p14.t7	1		unclassified				
sb_gmnlkfta_0007p18.t7	1		unclassified				EY973689
sb_gmnlkfta_0007p19.t7	1		unclassified				EY973690

[1]Annotations presented in the supplemental table were generated with AutoFACT [18], while annotations presented in the manuscript are recent BLASTx hits that reflect a more updated state of the NCBI's nr protein database.

Supplemental Table S1B. Contigs in library gmnllfta (forward heat-shock liver SSH library) with supporting annotations[1], statistics, and contributing EST accession numbers

Sequence	Count	Sub-sequences	AutoFACT Description	GO terms	E-value	Identity	Accession Number
sb_gmnllfta.2.C1	25	sb_gmnllfta_0005c24.t7<br>sb_gmnllfta_0002h08.t7<br>sb_gmnllfta_0002c10.t7<br>sb_gmnllfta_0003i24.t7<br>sb_gmnllfta_0002l11.t7<br>sb_gmnllfta_0003p08.t7<br>sb_gmnllfta_0004p07.t7<br>sb_gmnllfta_0006d18.t7<br>sb_gmnllfta_0006i18.t7<br>sb_gmnllfta_0003e02.t7<br>sb_gmnllfta_0001d08.t7<br>sb_gmnllfta_0002a24.t7<br>sb_gmnllfta_0002c18.t7<br>sb_gmnllfta_0007i17.t7<br>sb_gmnllfta_0005c23.t7<br>sb_gmnllfta_0002g15.t7<br>sb_gmnllfta_0003k18.t7<br>sb_gmnllfta_0007g02.t7<br>sb_gmnllfta_0007o17.t7<br>sb_gmnllfta_0005i09.t7<br>sb_gmnllfta_0001b03.t7<br>sb_gmnllfta_0001e02.t7<br>sb_gmnllfta_0004l21.t7<br>sb_gmnllfta_0006a15.t7<br>sb_gmnllfta_0006p08.t7	Cluster: Hemoglobin subunit beta-1; n=2; Gadidae|Rep: Hemoglobin subunit beta-1 - Gadus morhua (Atlantic cod)	GO:0020037<br>GO:0019825<br>GO:0015671<br>GO:0006810<br>GO:0005833<br>GO:0005506<br>GO:0005344	7e-66	99% (121/122)	ES781619<br>ES781020<br>ES781706<br>ES781794<br>ES780811<br>ES781858<br>ES781315<br>ES780830<br>ES781504<br>ES781949<br>ES781060<br>ES780950<br>ES781580<br>FL634203<br>ES781489<br>ES781213<br>ES781242<br>FL634159<br>FL634302<br>ES780932<br>ES781639<br>ES781598<br>ES781295<br>ES780910<br>ES781376
sb_gmnllfta.3.C1	24	sb_gmnllfta_0004d16.t7<br>sb_gmnllfta_0005h02.t7<br>sb_gmnllfta_0002l12.t7<br>sb_gmnllfta_0006f16.t7<br>sb_gmnllfta_0005b01.t7<br>sb_gmnllfta_0005h13.t7<br>sb_gmnllfta_0007j04.t7<br>sb_gmnllfta_0005l13.t7<br>sb_gmnllfta_0006e21.t7<br>sb_gmnllfta_0002m15.t7<br>sb_gmnllfta_0003c06.t7<br>sb_gmnllfta_0003e20.t7<br>sb_gmnllfta_0004d19.t7<br>sb_gmnllfta_0005e09.t7<br>sb_gmnllfta_0006g19.t7<br>sb_gmnllfta_0006k14.t7<br>sb_gmnllfta_0006m06.t7<br>sb_gmnllfta_0007a06.t7<br>sb_gmnllfta_0007c11.t7<br>sb_gmnllfta_0007d09.t7<br>sb_gmnllfta_0007j20.t7<br>sb_gmnllfta_0007e21.t7<br>sb_gmnllfta_0001e06.t7<br>sb_gmnllfta_0007c24.t7	Cluster: Hemoglobin subunit alpha-1; n=3; Gadidae|Rep: Hemoglobin subunit alpha-1 - Gadus morhua (Atlantic cod)	GO:0020037<br>GO:0019825<br>GO:0015671<br>GO:0006810<br>GO:0005833<br>GO:0005506<br>GO:0005344	6e-55	94% (106/112)	ES780876<br>ES781429<br>ES780873<br>ES780909<br>ES780916<br>ES781368<br>FL634210<br>ES781819<br>ES780826<br>ES781767<br>ES781053<br>ES781536<br>ES781252<br>ES781264<br>ES781641<br>ES781525<br>ES781402<br>FL634065<br>FL634100<br>FL634116<br>FL634223<br>FL634140<br>ES781517<br>FL634110
sb_gmnllfta.28.C1	10	sb_gmnllfta_0006k22.t7<br>sb_gmnllfta_0006c20.t7<br>sb_gmnllfta_0006n18.t7<br>sb_gmnllfta_0003p24.t7<br>sb_gmnllfta_0004a01.t7	Cluster: Hemoglobin subunit beta; n=3; Gadidae|Rep: Hemoglobin subunit beta - Gadus morhua (Atlantic cod)	GO:0020037<br>GO:0019825<br>GO:0015671<br>GO:0006810<br>GO:0005833	3e-45	95% (88/92)	ES781033<br>ES781936<br>ES781401<br>ES781720<br>ES781423

		sb_gmnllfta_0005d17.t7<br>sb_gmnllfta_0005e20.t7<br>sb_gmnllfta_0005h07.t7<br>sb_gmnllfta_0007f02.t7<br>sb_gmnllfta_0007f08.t7		GO:0005506<br>GO:0005344			ES781015<br>ES781347<br>ES781675<br>FL634143<br>FL634148
sb_gmnllfta.65.C1	10	sb_gmnllfta_0006f20.t7<br>sb_gmnllfta_0007m08.t7<br>sb_gmnllfta_0003e10.t7<br>sb_gmnllfta_0004b21.t7<br>sb_gmnllfta_0003m11.t7<br>sb_gmnllfta_0006c09.t7<br>sb_gmnllfta_0006n14.t7<br>sb_gmnllfta_0007g09.t7<br>sb_gmnllfta_0004g21.t7<br>sb_gmnllfta_0005g11.t7	Cluster: Protein phosphatase 2 (Formerly 2A), regulatory subunit B (PR 52), beta isoform; n=2; Danio rerio|Rep: Protein phosphatase 2 (Formerly 2A), regulatory subunit B (PR 52), beta isoform - Danio rerio (Zebrafish) (Brachydanio rerio)		5e-05	60% (24/40)	ES781643<br>FL634261<br>ES780933<br>ES781128<br>ES781736<br>ES781854<br>ES781802<br>FL634165<br>ES780796<br>ES781602
sb_gmnllfta.11.C1	7	sb_gmnllfta_0005e08.t7<br>sb_gmnllfta_0006p22.t7<br>sb_gmnllfta_0005b02.t7<br>sb_gmnllfta_0007m20.t7<br>sb_gmnllfta_0006l06.t7<br>sb_gmnllfta_0005b22.t7<br>sb_gmnllfta_0005j12.t7	Cluster: Serotransferrin; n=1; Gadus morhua|Rep: Serotransferrin - Gadus morhua (Atlantic cod)	GO:0008199<br>GO:0006879<br>GO:0006826<br>GO:0006811<br>GO:0006810<br>GO:0005576<br>GO:0005506	2e-58	93% (113/121)	ES781328<br>ES781391<br>ES780858<br>FL634273<br>ES781256<br>ES781392<br>ES780979
sb_gmnllfta.126.C1	7	sb_gmnllfta_0002f05.t7<br>sb_gmnllfta_0006c13.t7<br>sb_gmnllfta_0005a09.t7<br>sb_gmnllfta_0006i15.t7<br>sb_gmnllfta_0007h05.t7<br>sb_gmnllfta_0002j12.t7<br>sb_gmnllfta_0007c10.t7	unclassified				ES781136<br>ES781288<br>ES781705<br>ES781692<br>FL634181<br>ES781873<br>FL634099
sb_gmnllfta.22.C1	7	sb_gmnllfta_0007a20.t7<br>sb_gmnllfta_0002o14.t7<br>sb_gmnllfta_0004o14.t7<br>sb_gmnllfta_0005b15.t7<br>sb_gmnllfta_0005d16.t7<br>sb_gmnllfta_0006n05.t7<br>sb_gmnllfta_0004i19.t7	unclassified				FL634074<br>ES781467<br>ES781263<br>ES781826<br>ES781089<br>ES780963<br>ES781680
sb_gmnllfta.73.C1	7	sb_gmnllfta_0007n20.t7<br>sb_gmnllfta_0001a01.t7<br>sb_gmnllfta_0005m08.t7<br>sb_gmnllfta_0001h02.t7<br>sb_gmnllfta_0007f09.t7<br>sb_gmnllfta_0007k16.t7<br>sb_gmnllfta_0007p18.t7	zgc:73262; zgc:73262; K02978 small subunit ribosomal protein S27e		4e-33	87% (67/77)	FL634288<br>ES780946<br>ES781630<br>ES781028<br>FL634149<br>FL634236<br>FL634316
sb_gmnllfta.0.C1	6	sb_gmnllfta_0006g06.t7<br>sb_gmnllfta_0004o20.t7	Cluster: Complement C3 precursor [Contains: Complement C3 beta chain; Complement C3 alpha chain; C3a anaphylatoxin; Complement		8e-61	54%	ES780831<br>ES781791

		sb_gmnllfta_0006i16.t7<br>sb_gmnllfta_0002g23.t7<br>sb_gmnllfta_0003o20.t7<br>sb_gmnllfta_0002b15.t7	C3b alpha' chain; Complement C3c alpha' chain fragment 1; Complement C3dg fragment; Complement C3g fragment; Complement C3d fragment; Com; n=1; Takifugu rubripes|Rep: Complement C3 precursor [Contains: Complement C3 beta chain; Complement C3 alpha chain; C3a anaphylatoxin; Complement C3b alpha' chain; Complement C3c alpha' chain fragment 1; Complement C3dg fragment; Complement C3g fragment; Complement C3d fragment; Com - Takifugu rubripes			(125/228)	ES781782<br>ES781948<br>ES780897<br>ES780928
sb_gmnllfta.180.C1	6	sb_gmnllfta_0004h13.t7<br>sb_gmnllfta_0003h13.t7<br>sb_gmnllfta_0004o22.t7<br>sb_gmnllfta_0006a16.t7<br>sb_gmnllfta_0003k01.t7<br>sb_gmnllfta_0006a10.t7	Cluster: Ubiquitin/40S ribosomal protein S27a fusion protein; n=16; Metazoa|Rep: Ubiquitin/40S ribosomal protein S27a fusion protein - Argas monolakensis	GO:0006412<br>GO:0005840<br>GO:0005622<br>GO:0003735	7e-29	82%<br>(65/79)	ES781204<br>ES781496<br>ES781746<br>ES780869<br>ES781752<br>ES780960
sb_gmnllfta.56.C1	6	sb_gmnllfta_0006a13.t7<br>sb_gmnllfta_0007d10.t7<br>sb_gmnllfta_0007p06.t7<br>sb_gmnllfta_0003o01.t7<br>sb_gmnllfta_0006c01.t7<br>sb_gmnllfta_0005b12.t7	Cluster: UTP--glucose-1-phosphate uridylyltransferase 2 (EC 2.7.7.9) (UDP- glucose pyrophosphorylase 2) (UDPGP 2) (UGPase 2).; n=1; Takifugu rubripes|Rep: UTP--glucose-1-phosphate uridylyltransferase 2 (EC 2.7.7.9) (UDP- glucose pyrophosphorylase 2) (UDPGP 2) (UGPase 2). - Takifugu rubripes		5e-35	94%<br>(73/77)	ES781039<br>FL634117<br>FL634309<br>ES781301<br>ES781460<br>ES781978
sb_gmnllfta.7.C1	6	sb_gmnllfta_0006l12.t7<br>sb_gmnllfta_0003f13.t7<br>sb_gmnllfta_0005m11.t7<br>sb_gmnllfta_0005g14.t7<br>sb_gmnllfta_0004n20.t7<br>sb_gmnllfta_0006b10.t7	Cluster: Novel protein; n=2; Danio rerio|Rep: Novel protein - Danio rerio (Zebrafish) (Brachydanio rerio)		8e-09	30%<br>(44/146)	ES781569<br>ES781648<br>ES780978<br>ES781498<br>ES781052<br>ES781480
sb_gmnllfta.83.C1	6	sb_gmnllfta_0005a17.t7<br>sb_gmnllfta_0006k20.t7<br>sb_gmnllfta_0006j02.t7<br>sb_gmnllfta_0005k19.t7<br>sb_gmnllfta_0005o05.t7<br>sb_gmnllfta_0005f07.t7	Cluster: similar to splicing factor, arginine/serine-rich 2 (Sfrs2), mRNA; n=1; Rattus norvegicus|Rep: similar to splicing factor, arginine/serine-rich 2 (Sfrs2), mRNA - Rattus norvegicus		2e-06	61%<br>(21/34)	ES781351<br>ES780981<br>ES781544<br>ES781674<br>ES781618<br>ES781483
sb_gmnllfta.108.C1	5	sb_gmnllfta_0007n15.t7<br>sb_gmnllfta_0002b24.t7<br>sb_gmnllfta_0004b24.t7<br>sb_gmnllfta_0006d15.t7<br>sb_gmnllfta_0006c24.t7	unclassified				FL634283<br>ES781487<br>ES781400<br>ES781237<br>ES781694
sb_gmnllfta.118.C1	5	sb_gmnllfta_0005e01.t7<br>sb_gmnllfta_0006h14.t7<br>sb_gmnllfta_0007g11.t7<br>sb_gmnllfta_0005m06.t7<br>sb_gmnllfta_0003b01.t7	Cluster: Ribosomal protein S6; n=5; Amniota|Rep: Ribosomal protein S6 - Struthio camelus (Ostrich)	GO:0005840<br>GO:0005622<br>GO:0003735	1e-77	93%<br>(140/149)	ES780851<br>ES781012<br>FL634167<br>ES781942<br>ES781946

sb_gmnllfta.138.C1	5	sb_gmnllfta_0006h04.t7<br>sb_gmnllfta_0004m23.t7<br>sb_gmnllfta_0006j12.t7<br>sb_gmnllfta_0007f14.t7<br>sb_gmnllfta_0007k12.t7	unclassified				ES781834<br>ES781488<br>ES781179<br>FL634151<br>FL634233
sb_gmnllfta.46.C1	5	sb_gmnllfta_0007g13.t7<br>sb_gmnllfta_0001g10.t7<br>sb_gmnllfta_0005l04.t7<br>sb_gmnllfta_0001a09.t7<br>sb_gmnllfta_0006o23.t7	Cluster: Ribosomal protein L18; n=5; Euteleostomi|Rep: Ribosomal protein L18 - Pagrus major (Red sea bream) (Chrysophrys major)	GO:0006412<br>GO:0005840<br>GO:0005622<br>GO:0003735	8e-45	86%<br>(88/102)	FL634169<br>ES780894<br>ES780864<br>ES781338<br>ES781518
sb_gmnllfta.49.C1	5	sb_gmnllfta_0007l07.t7<br>sb_gmnllfta_0006f09.t7<br>sb_gmnllfta_0005d09.t7<br>sb_gmnllfta_0002c17.t7<br>sb_gmnllfta_0002k11.t7	unclassified				FL634246<br>ES781568<br>ES781412<br>ES781875<br>ES780877
sb_gmnllfta.5.C1	5	sb_gmnllfta_0003o16.t7<br>sb_gmnllfta_0002h18.t7<br>sb_gmnllfta_0005l07.t7<br>sb_gmnllfta_0004o16.t7<br>sb_gmnllfta_0006a22.t7	Cluster: Ribosomal L1 domain-containing protein 1 (Cellular senescence- inhibited gene protein) (Protein PBK1) (CATX-11).; n=1; Takifugu rubripes|Rep: Ribosomal L1 domain-containing protein 1 (Cellular senescence- inhibited gene protein) (Protein PBK1) (CATX-11). - Takifugu rubripes		6e-74	66%<br>(133/199)	ES781646<br>ES781690<br>ES780920<br>ES781375<br>ES781654
sb_gmnllfta.6.C1	5	sb_gmnllfta_0003n01.t7<br>sb_gmnllfta_0002n06.t7<br>sb_gmnllfta_0007e04.t7<br>sb_gmnllfta_0004m08.t7<br>sb_gmnllfta_0007i06.t7	Cluster: Alpha-2-antiplasmin precursor (Alpha-2-plasmin inhibitor) (Alpha-2-PI) (Alpha-2-AP).; n=1; Takifugu rubripes|Rep: Alpha-2-antiplasmin precursor (Alpha-2-plasmin inhibitor) (Alpha-2-PI) (Alpha-2-AP). - Takifugu rubripes		7e-49	53%<br>(93/174)	ES781450<br>ES781422<br>FL634127<br>ES781514<br>FL634196
sb_gmnllfta.79.C1	5	sb_gmnllfta_0004h08.t7<br>sb_gmnllfta_0005g22.t7<br>sb_gmnllfta_0005d12.t7<br>sb_gmnllfta_0006n08.t7<br>sb_gmnllfta_0003i21.t7	Cluster: Homolog of Homo sapiens "PePtidylProlyl isomerase A-like; n=1; Takifugu rubripes|Rep: Homolog of Homo sapiens "PePtidylProlyl isomerase A-like - Takifugu rubripes		4e-75	82%<br>(135/164)	ES781823<br>ES781066<br>ES780835<br>ES781334<br>ES781909
sb_gmnllfta.110.C1	4	sb_gmnllfta_0006a20.t7<br>sb_gmnllfta_0005n23.t7<br>sb_gmnllfta_0007f20.t7<br>sb_gmnllfta_0005f16.t7	Cluster: Hemopexin-like protein; n=1; Gillichthys mirabilis|Rep: Hemopexin-like protein - Gillichthys mirabilis (Long-jawed mudsucker)	GO:0003824	1e-46	54%<br>(92/169)	ES781542<br>ES780855<br>FL634155<br>ES781325
sb_gmnllfta.130.C1	4	sb_gmnllfta_0006e13.t7<br>sb_gmnllfta_0004g17.t7	Cluster: Fetuin-B; n=1; Carassius auratus gibelio|Rep: Fetuin-B -		2e-06	25%	ES781481<br>ES781608

		sb_gmnllfta_0005i11.t7<br>sb_gmnllfta_0002g17.t7	Carassius auratus gibelio			(42/165)	ES781414<br>ES781113
sb_gmnllfta.158.C1	4	sb_gmnllfta_0004n18.t7<br>sb_gmnllfta_0005h03.t7<br>sb_gmnllfta_0006f23.t7<br>sb_gmnllfta_0007d21.t7	LSU rRNA; Xenopus borealis		1e-160	97%<br>(311/319)	ES781814<br>ES781437<br>ES781543<br>FL634124
sb_gmnllfta.17.C1	4	sb_gmnllfta_0006h22.t7<br>sb_gmnllfta_0005j05.t7<br>sb_gmnllfta_0004m11.t7<br>sb_gmnllfta_0002m11.t7	unclassified				ES781407<br>ES781947<br>ES780840<br>ES781871
sb_gmnllfta.18.C1	4	sb_gmnllfta_0003l08.t7<br>sb_gmnllfta_0006h03.t7<br>sb_gmnllfta_0005p01.t7<br>sb_gmnllfta_0007c05.t7	unclassified				ES781645<br>ES781989<br>ES781313<br>FL634096
sb_gmnllfta.24.C1	4	sb_gmnllfta_0007a14.t7<br>sb_gmnllfta_0002h19.t7<br>sb_gmnllfta_0007p08.t7<br>sb_gmnllfta_0007m13.t7	Cluster: Chaperonin containing TCP1, subunit 2; n=4; Clupeocephala|Rep: Chaperonin containing TCP1, subunit 2 - Danio rerio (Zebrafish) (Brachydanio rerio)		1e-78	76%<br>(150/196)	FL634071<br>ES781741<br>FL634310<br>FL634266
sb_gmnllfta.30.C1	4	sb_gmnllfta_0003k08.t7<br>sb_gmnllfta_0006h09.t7<br>sb_gmnllfta_0006j21.t7<br>sb_gmnllfta_0006l21.t7	unclassified				ES781603<br>ES781455<br>ES781702<br>ES781032
sb_gmnllfta.32.C1	4	sb_gmnllfta_0005h06.t7<br>sb_gmnllfta_0006j15.t7<br>sb_gmnllfta_0004f14.t7<br>sb_gmnllfta_0006b11.t7	unclassified				ES781620<br>ES781335<br>ES781219<br>ES781524
sb_gmnllfta.38.C1	4	sb_gmnllfta_0002h22.t7<br>sb_gmnllfta_0004h22.t7<br>sb_gmnllfta_0006e20.t7<br>sb_gmnllfta_0004n24.t7	Cluster: Protein kinase C substrate 80K-H; n=2; Danio rerio|Rep: Protein kinase C substrate 80K-H - Danio rerio (Zebrafish) (Brachydanio rerio)		1e-13	65%<br>(34/52)	ES781049<br>ES781803<br>ES780809<br>ES780786
sb_gmnllfta.43.C1	4	sb_gmnllfta_0005l11.t7<br>sb_gmnllfta_0001g08.t7<br>sb_gmnllfta_0007m17.t7	Cluster: Keratin; n=1; Carassius auratus|Rep: Keratin - Carassius auratus (Goldfish)	GO:0005198	5e-48	67%<br>(102/152)	ES781712<br>ES781451<br>FL634270<br>FL634212

		sb_gmnllfta_0007j06.t7					
sb_gmnllfta.54.C1	4	sb_gmnllfta_0002h23.t7<br>sb_gmnllfta_0006i05.t7<br>sb_gmnllfta_0007n16.t7<br>sb_gmnllfta_0003k11.t7	Cluster: titin isoform novex-1; n=2; Homo sapiens|Rep: titin isoform novex-1 - Homo sapiens		1e-100	99% (183/184)	ES781069<br>ES781030<br>FL634284<br>ES781086
sb_gmnllfta.58.C1	4	sb_gmnllfta_0005p04.t7<br>sb_gmnllfta_0006n20.t7<br>sb_gmnllfta_0005g23.t7<br>sb_gmnllfta_0007a02.t7	unclassified				ES781207<br>ES781312<br>ES781055<br>FL634062
sb_gmnllfta.59.C1	4	sb_gmnllfta_0006i12.t7<br>sb_gmnllfta_0004b17.t7<br>sb_gmnllfta_0003b17.t7<br>sb_gmnllfta_0005c20.t7	Cluster: Zgc:136684; n=3; Danio rerio|Rep: Zgc:136684 - Danio rerio (Zebrafish) (Brachydanio rerio)		2e-55	81% (95/117)	ES781859<br>ES781897<br>ES780991<br>ES781420
sb_gmnllfta.62.C1	4	sb_gmnllfta_0004o18.t7<br>sb_gmnllfta_0002e09.t7<br>sb_gmnllfta_0006j13.t7<br>sb_gmnllfta_0006a04.t7	Cluster: Glutathione S-transferase pi; n=2; Cyprinidae|Rep: Glutathione S-transferase pi - Danio rerio (Zebrafish) (Brachydanio rerio)		2e-13	57% (38/66)	ES781078<br>ES781552<br>ES781233<br>ES781860
sb_gmnllfta.63.C1	4	sb_gmnllfta_0005j17.t7<br>sb_gmnllfta_0005p06.t7<br>sb_gmnllfta_0006h23.t7<br>sb_gmnllfta_0005n17.t7	Cluster: Ferritin, heavy subunit; n=2; Salmonidae|Rep: Ferritin, heavy subunit - Salmo salar (Atlantic salmon)	GO:0046872<br>GO:0016491<br>GO:0008199<br>GO:0006879<br>GO:0006826<br>GO:0005506<br>GO:0005488<br>GO:0004322	6e-85	88% (148/167)	ES780935<br>ES781162<br>ES781459<br>ES781649
sb_gmnllfta.68.C1	4	sb_gmnllfta_0007i04.t7<br>sb_gmnllfta_0006g22.t7<br>sb_gmnllfta_0002n11.t7<br>sb_gmnllfta_0005p07.t7	unclassified				FL634194<br>ES781156<br>ES781198<br>ES781100
sb_gmnllfta.71.C1	4	sb_gmnllfta_0007i05.t7<br>sb_gmnllfta_0005a05.t7<br>sb_gmnllfta_0005b24.t7<br>sb_gmnllfta_0005h20.t7	Cluster: Novel protein; n=5; Danio rerio|Rep: Novel protein - Danio rerio (Zebrafish) (Brachydanio rerio)		3e-42	51% (78/151)	FL634195<br>ES781540<br>ES781180<br>ES781903
sb_gmnllfta.77.C1	4	sb_gmnllfta_0005c02.t7<br>sb_gmnllfta_0006d21.t7<br>sb_gmnllfta_0006f07.t7	Cluster: Cytokeratin; n=1; Stizostedion vitreum vitreum|Rep:	GO:0005198	3e-06	51%	ES781904<br>ES781879<br>ES781862

		sb_gmnllfta_0007k14.t7	Cytokeratin - Stizostedion vitreum vitreum			(32/62)	FL634235
sb_gmnllfta.80.C1	4	sb_gmnllfta_0004c16.t7<br>sb_gmnllfta_0002d10.t7<br>sb_gmnllfta_0002j20.t7<br>sb_gmnllfta_0003c16.t7	unclassified				ES780813<br>ES781764<br>ES781394<br>ES781713
sb_gmnllfta.85.C1	4	sb_gmnllfta_0007h13.t7<br>sb_gmnllfta_0005b17.t7<br>sb_gmnllfta_0005d18.t7<br>sb_gmnllfta_0005h17.t7	TMA7 domain containing protein		1e-27	80%<br>(51/63)	FL634186<br>ES781703<br>ES781396<br>ES781170
sb_gmnllfta.86.C1	4	sb_gmnllfta_0003a01.t7<br>sb_gmnllfta_0002c08.t7<br>sb_gmnllfta_0002i11.t7<br>sb_gmnllfta_0007o20.t7	Cluster: Complement component C3; n=1; Paralichthys olivaceus|Rep: Complement component C3 - Paralichthys olivaceus (Japanese flounder)	GO:0005515<br>GO:0004866	6e-35	54%<br>(72/132)	ES781119<br>ES781215<br>ES781109<br>FL634304
sb_gmnllfta.92.C1	4	sb_gmnllfta_0006p24.t7<br>sb_gmnllfta_0005j14.t7<br>sb_gmnllfta_0005c01.t7<br>sb_gmnllfta_0001g01.t7	LOC708771; similar to Immunoglobulin lambda-like polypeptide 1 precursor (Immunoglobulin-related protein 14.1) (Immunoglobulin omega polypeptide) (Ig lambda-5) (CD179b antigen); K06554 immunoglobulin lambda-like polypeptide 1		7e-13	38%<br>(27/70)	ES781181<br>ES780842<br>ES781969<br>ES781883
sb_gmnllfta.1.C1	3	sb_gmnllfta_0004a23.t7<br>sb_gmnllfta_0004d10.t7<br>sb_gmnllfta_0005g24.t7	unclassified				ES781906<br>ES781082<br>ES780900
sb_gmnllfta.106.C1	3	sb_gmnllfta_0007e14.t7<br>sb_gmnllfta_0005p09.t7<br>sb_gmnllfta_0005p23.t7	unclassified				FL634135<br>ES780801<br>ES780807
sb_gmnllfta.113.C1	3	sb_gmnllfta_0006p09.t7<br>sb_gmnllfta_0005j18.t7<br>sb_gmnllfta_0007n19.t7	Cluster: Chaperone protein GP96 (Tumor rejection antigen (Gp96) 1) (Heat shock protein 90kDa beta (Grp94), member 1); n=1; Danio rerio|Rep: Chaperone protein GP96 (Tumor rejection antigen (Gp96) 1) (Heat shock protein 90kDa beta (Grp94), member 1) - Danio rerio (Zebrafish) (Brachydanio rerio)		1e-54	79%<br>(107/134)	ES781350<br>ES781184<br>FL634287
sb_gmnllfta.120.C1	3	sb_gmnllfta_0004k16.t7<br>sb_gmnllfta_0006l20.t7<br>sb_gmnllfta_0005l21.t7	unclassified				ES781821<br>ES781094<br>ES781129

sb_gmnllfta.121.C1	3	sb_gmnllfta_0006g15.t7<br>sb_gmnllfta_0007p21.t7<br>sb_gmnllfta_0002d15.t7	Cluster: Eukaryotic elongation factor 1 alpha; n=1; Gadus morhua|Rep: Eukaryotic elongation factor 1 alpha - Gadus morhua (Atlantic cod)		2e-37	98% (61/62)	ES781916<br>FL634318<br>ES781887
sb_gmnllfta.122.C1	3	sb_gmnllfta_0004b18.t7<br>sb_gmnllfta_0003m05.t7<br>sb_gmnllfta_0005k23.t7	LSU rRNA; Hydrolagus colliei		3e-91	99% (169/170)	ES781554<br>ES780856<br>ES781138
sb_gmnllfta.129.C1	3	sb_gmnllfta_0004k10.t7<br>sb_gmnllfta_0002k10.t7<br>sb_gmnllfta_0003g16.t7	Cluster: Warm-temperature-acclimation-related-65kDa-protein-like-protein; n=2; Takifugu rubripes|Rep: Warm-temperature-acclimation-related-65kDa-protein-like- protein - Fugu rubripes (Japanese pufferfish) (Takifugu rubripes)	GO:0003824	2e-13	70% (34/48)	ES781928<br>ES780931<br>ES781268
sb_gmnllfta.131.C1	3	sb_gmnllfta_0005p21.t7<br>sb_gmnllfta_0007o05.t7<br>sb_gmnllfta_0004a19.t7	Cluster: PREDICTED: similar to Actin, cytoplasmic 2 (Gamma-actin); n=1; Rattus norvegicus|Rep: PREDICTED: similar to Actin, cytoplasmic 2 (Gamma-actin) - Rattus norvegicus		1e-23	96% (53/55)	ES780941<br>FL634292<br>ES780924
sb_gmnllfta.143.C1	3	sb_gmnllfta_0006k10.t7<br>sb_gmnllfta_0006d08.t7<br>sb_gmnllfta_0005o21.t7	Cluster: Cytochrome c oxidase, subunit Va; n=3; Tetrapoda|Rep: Cytochrome c oxidase, subunit Va - Xenopus tropicalis (Western clawed frog) (Silurana tropicalis)	GO:0004129	2e-47	71% (100/139)	ES781586<br>ES781960<br>ES781952
sb_gmnllfta.148.C1	3	sb_gmnllfta_0003k16.t7<br>sb_gmnllfta_0007p11.t7<br>sb_gmnllfta_0003d04.t7	Cluster: UPI0000ECBEDF related cluster; n=1; Gallus gallus|Rep: UPI0000ECBEDF UniRef100 entry - Gallus gallus		1e-09	28% (52/182)	ES780925<br>FL634313<br>ES781065
sb_gmnllfta.149.C1	3	sb_gmnllfta_0004k24.t7<br>sb_gmnllfta_0005k13.t7<br>sb_gmnllfta_0006b22.t7	Cluster: Zgc:153670; n=2; Danio rerio|Rep: Zgc:153670 - Danio rerio (Zebrafish) (Brachydanio rerio)		1e-11	50% (41/81)	ES781127<br>ES781727<br>ES780827
sb_gmnllfta.151.C1	3	sb_gmnllfta_0005f15.t7<br>sb_gmnllfta_0006d13.t7<br>sb_gmnllfta_0007k07.t7	Cluster: NADH dehydrogenase (Ubiquinone) flavoprotein 1; n=3; Clupeocephala|Rep: NADH dehydrogenase (Ubiquinone) flavoprotein 1 - Danio rerio (Zebrafish) (Brachydanio rerio)		9e-32	89% (62/69)	ES781374<br>ES781339<br>FL634230
sb_gmnllfta.160.C1	3	sb_gmnllfta_0002e16.t7<br>sb_gmnllfta_0006l23.t7<br>sb_gmnllfta_0007m04.t7	ifrd2; interferon-related developmental regulator 2		3e-21	89% (53/59)	ES780926<br>ES780984<br>FL634259

sb_gmnllfta.161.C1	3	sb_gmnllfta_0005p24.t7<br>sb_gmnllfta_0004f23.t7<br>sb_gmnllfta_0003f23.t7	Cluster: Lypla3 protein; n=5; Danio rerio|Rep: Lypla3 protein - Danio rerio (Zebrafish) (Brachydanio rerio)		3e-42	66% (74/111)	ES780962<br>ES781964<br>ES781071
sb_gmnllfta.178.C1	3	sb_gmnllfta_0006i06.t7<br>sb_gmnllfta_0002e15.t7<br>sb_gmnllfta_0005d06.t7	Cluster: 60S ribosomal protein L3 (HIV-1 TAR RNA-binding protein B) (TARBP-B).; n=3; Tetrapoda|Rep: 60S ribosomal protein L3 (HIV-1 TAR RNA-binding protein B) (TARBP-B). - Xenopus tropicalis		4e-47	100% (53/53)	ES780965<br>ES780837<br>ES781747
sb_gmnllfta.185.C1	3	sb_gmnllfta_0005b21.t7<br>sb_gmnllfta_0005j19.t7<br>sb_gmnllfta_0007p10.t7	unclassified				ES781294<br>ES781251<br>FL634312
sb_gmnllfta.187.C1	3	sb_gmnllfta_0005g19.t7<br>sb_gmnllfta_0002g19.t7<br>sb_gmnllfta_0007n18.t7	Unassigned protein				ES781679<br>ES780794<br>FL634286
sb_gmnllfta.194.C1	3	sb_gmnllfta_0006c10.t7<br>sb_gmnllfta_0006l10.t7<br>sb_gmnllfta_0007o07.t7	unclassified				ES781337<br>ES781660<br>FL634294
sb_gmnllfta.20.C1	3	sb_gmnllfta_0004h20.t7<br>sb_gmnllfta_0003h20.t7<br>sb_gmnllfta_0002b07.t7	unclassified				ES781688<br>ES780788<br>ES781954
sb_gmnllfta.26.C1	3	sb_gmnllfta_0005j20.t7<br>sb_gmnllfta_0006f17.t7<br>sb_gmnllfta_0006j11.t7	Cluster: Fragile X mental retardation, autosomal homolog 1; n=3; Danio rerio|Rep: Fragile X mental retardation, autosomal homolog 1 - Danio rerio (Zebrafish) (Brachydanio rerio)		9e-98	82% (179/216)	ES781667<br>ES780892<br>ES781135
sb_gmnllfta.33.C1	3	sb_gmnllfta_0003a20.t7<br>sb_gmnllfta_0007g12.t7<br>sb_gmnllfta_0007o15.t7	Cluster: Annexin max4; n=1; Oryzias latipes|Rep: Annexin max4 - Oryzias latipes (Medaka fish) (Japanese ricefish)	GO:0005509	4e-33	82% (48/58)	ES781075<br>FL634168<br>FL634300
sb_gmnllfta.35.C1	3	sb_gmnllfta_0005o16.t7<br>sb_gmnllfta_0003e21.t7<br>sb_gmnllfta_0006j16.t7	Cluster: PREDICTED: similar to ribosomal protein S17; n=2; Laurasiatheria|Rep: PREDICTED: similar to ribosomal protein S17 - Equus caballus		9e-31	91% (66/72)	ES781196<br>ES781484<br>ES781287

sb_gmnllfta.37.C1	3	sb_gmnllfta_0005j23.t7<br>sb_gmnllfta_0002m23.t7<br>sb_gmnllfta_0006g18.t7	Cluster: PREDICTED: similar to ribosomal protein L37; n=1; Monodelphis domestica|Rep: PREDICTED: similar to ribosomal protein L37 - Monodelphis domestica		2e-20	95% (43/45)	ES781578<br>ES781397<br>ES781655
sb_gmnllfta.4.C1	3	sb_gmnllfta_0006j23.t7<br>sb_gmnllfta_0005c22.t7<br>sb_gmnllfta_0007p05.t7	Cluster: Ribosomal protein L15; n=5; Actinopteri|Rep: Ribosomal protein L15 - Acipenser guldenstadti (Caspian sturgeon) (Russian sturgeon)	GO:0006412<br>GO:0005840<br>GO:0005622<br>GO:0003735	3e-63	92% (113/122)	ES781829<br>ES781532<br>FL634308
sb_gmnllfta.42.C1	3	sb_gmnllfta_0006p18.t7<br>sb_gmnllfta_0005m23.t7<br>sb_gmnllfta_0003m16.t7	unclassified				ES781448<br>ES781671<br>ES781890
sb_gmnllfta.51.C1	3	sb_gmnllfta_0003o12.t7<br>sb_gmnllfta_0003p12.t7<br>sb_gmnllfta_0007o11.t7	unclassified				ES781475<br>ES781285<br>FL634296
sb_gmnllfta.53.C1	3	sb_gmnllfta_0006a17.t7<br>sb_gmnllfta_0007o14.t7<br>sb_gmnllfta_0003p21.t7	unclassified				ES780800<br>FL634299<br>ES781933
sb_gmnllfta.61.C1	3	sb_gmnllfta_0005k18.t7<br>sb_gmnllfta_0005m15.t7<br>sb_gmnllfta_0002i23.t7	Cluster: Zgc:101851; n=2; Danio rerio|Rep: Zgc:101851 - Danio rerio (Zebrafish) (Brachydanio rerio)		4e-44	68% (85/124)	ES781621<br>ES780871<br>ES781812
sb_gmnllfta.64.C1	3	sb_gmnllfta_0005d23.t7<br>sb_gmnllfta_0005g08.t7<br>sb_gmnllfta_0005d20.t7	unclassified				ES781425<br>ES781088<br>ES781493
sb_gmnllfta.75.C1	3	sb_gmnllfta_0006o13.t7<br>sb_gmnllfta_0004d21.t7<br>sb_gmnllfta_0007o02.t7	unclassified				ES780906<br>ES781629<br>FL634291
sb_gmnllfta.78.C1	3	sb_gmnllfta_0007g01.t7<br>sb_gmnllfta_0007l01.t7<br>sb_gmnllfta_0007e01.t7	unclassified				FL634158<br>FL634241<br>FL634125

sb_gmnllfta.89.C1	3	sb_gmnllfta_0002j13.t7<br>sb_gmnllfta_0004d18.t7<br>sb_gmnllfta_0006j04.t7	Cluster: Probable Bax inhibitor 1; n=1; Paralichthys olivaceus|Rep: Probable Bax inhibitor 1 - Paralichthys olivaceus (Japanese flounder)	GO:0016021<br>GO:0016020<br>GO:0006915	1e-54	83% (107/128)	ES781893<br>ES781186<br>ES781462
sb_gmnllfta.90.C1	3	sb_gmnllfta_0007g10.t7<br>sb_gmnllfta_0007l02.t7<br>sb_gmnllfta_0005p14.t7	unclassified				FL634166<br>FL634242<br>ES781565
sb_gmnllfta.93.C1	3	sb_gmnllfta_0004a16.t7<br>sb_gmnllfta_0006h02.t7<br>sb_gmnllfta_0006h08.t7	Cluster: 60S ribosomal protein L7a; n=9; Amniota|Rep: 60S ribosomal protein L7a - Gallus gallus (Chicken)		3e-26	93% (57/61)	ES781197<br>ES781937<br>ES781433
sb_gmnllfta.96.C1	3	sb_gmnllfta_0005f12.t7<br>sb_gmnllfta_0005n05.t7<br>sb_gmnllfta_0006i20.t7	Cluster: Liver angiotensinogen; n=1; Rhabdosargus sarba|Rep: Liver angiotensinogen - Rhabdosargus sarba (goldlined seabream)		6e-29	70% (63/90)	ES781214<br>ES781182<br>ES781378
sb_gmnllfta.97.C1	3	sb_gmnllfta_0007d02.t7<br>sb_gmnllfta_0003j23.t7<br>sb_gmnllfta_0007h18.t7	Cluster: Ubiquitin carboxyl-terminal hydrolase 48 (EC 3.1.2.15) (Ubiquitin thioesterase 48) (Ubiquitin-specific-processing protease 48) (Deubiquitinating enzyme 48).; n=1; Takifugu rubripes|Rep: Ubiquitin carboxyl-terminal hydrolase 48 (EC 3.1.2.15) (Ubiquitin thioesterase 48) (Ubiquitin-specific-processing protease 48) (Deubiquitinating enzyme 48). - Takifugu rubripes		7e-37	91% (62/68)	FL634111<br>ES781123<br>FL634189
sb_gmnllfta.10.C1	2	sb_gmnllfta_0002o10.t7<br>sb_gmnllfta_0003p20.t7	Cluster: 78 kDa glucose-regulated protein precursor; n=1; Aplysia californica|Rep: 78 kDa glucose-regulated protein precursor - Aplysia californica (California sea hare)	GO:0005783<br>GO:0005524<br>GO:0000166	4e-28	96% (59/61)	ES781650<br>ES781994
sb_gmnllfta.100.C1	2	sb_gmnllfta_0006n12.t7<br>sb_gmnllfta_0005b16.t7	unclassified				ES781941<br>ES781739
sb_gmnllfta.101.C1	2	sb_gmnllfta_0002h10.t7<br>sb_gmnllfta_0007m15.t7	Cluster: PREDICTED: similar to ADP-ribosylation factor 1; n=1; Strongylocentrotus purpuratus|Rep: PREDICTED: similar to ADP-ribosylation factor 1 - Strongylocentrotus purpuratus		2e-11	76% (33/43)	ES781607<br>FL634268
sb_gmnllfta.102.C1	2	sb_gmnllfta_0006j24.t7<br>sb_gmnllfta_0003c19.t7	Unassigned protein		2e-05	56% (21/37)	ES781991<br>ES781440

sb_gmnllfta.103.C1	2	sb_gmnllfta_0002i12.t7<br>sb_gmnllfta_0004i12.t7	Cluster: N-acetylmuramoyl-L-alanine amidase precursor (EC 3.5.1.28) (Peptidoglycan recognition protein long) (PGRP-L) (Peptidoglycan recognition protein 2).; n=1; Takifugu rubripes|Rep: N-acetylmuramoyl-L-alanine amidase precursor (EC 3.5.1.28) (Peptidoglycan recognition protein long) (PGRP-L) (Peptidoglycan recognition protein 2). - Takifugu rubripes		6e-20	86% (37/43)	ES781201<br>ES781644
sb_gmnllfta.104.C1	2	sb_gmnllfta_0004k01.t7<br>sb_gmnllfta_0004n22.t7	unclassified				ES780853<br>ES780999
sb_gmnllfta.107.C1	2	sb_gmnllfta_0002h01.t7<br>sb_gmnllfta_0005h18.t7	unclassified				ES781185<br>ES780790
sb_gmnllfta.109.C1	2	sb_gmnllfta_0002c22.t7<br>sb_gmnllfta_0005k08.t7	Unassigned protein		1e-25	73% (34/46)	ES781254<br>ES781108
sb_gmnllfta.111.C1	2	sb_gmnllfta_0006l08.t7<br>sb_gmnllfta_0005f13.t7	unclassified				ES780957<br>ES781205
sb_gmnllfta.112.C1	2	sb_gmnllfta_0004g04.t7<br>sb_gmnllfta_0007i09.t7	unclassified				ES781143<br>FL634197
sb_gmnllfta.114.C1	2	sb_gmnllfta_0005a15.t7<br>sb_gmnllfta_0002f16.t7	unclassified				ES781330<br>ES781600
sb_gmnllfta.115.C1	2	sb_gmnllfta_0005p10.t7<br>sb_gmnllfta_0006p20.t7	Cluster: Heat shock protein 90Ae.; n=1; Takifugu rubripes|Rep: Heat shock protein 90Ae. - Takifugu rubripes		4e-37	88% (78/88)	ES781479<br>ES781278
sb_gmnllfta.116.C1	2	sb_gmnllfta_0007d05.t7<br>sb_gmnllfta_0007p09.t7	unclassified				FL634113<br>FL634311
sb_gmnllfta.117.C1	2	sb_gmnllfta_0001c12.t7<br>sb_gmnllfta_0001d11.t7	Cluster: Translationally-controlled tumor protein; n=6; Otophysi|Rep: Translationally-controlled tumor protein - Danio rerio (Zebrafish)		7e-28	62% (54/87)	ES781592<br>ES781590

			(Brachydanio rerio)				
sb_gmnllfta.119.C1	2	sb_gmnllfta_0007j13.t7<br>sb_gmnllfta_0007e02.t7	unclassified				FL634217<br>FL634126
sb_gmnllfta.12.C1	2	sb_gmnllfta_0003l03.t7<br>sb_gmnllfta_0007h11.t7	unclassified				ES781676<br>FL634185
sb_gmnllfta.123.C1	2	sb_gmnllfta_0002l23.t7<br>sb_gmnllfta_0004b07.t7	Cluster: ATP-dependent RNA helicase DHX8 (EC 3.6.1.-) (DEAH box protein 8) (RNA helicase HRH1).; n=1; Gallus gallus|Rep: ATP-dependent RNA helicase DHX8 (EC 3.6.1.-) (DEAH box protein 8) (RNA helicase HRH1). - Gallus gallus		8e-19	93%<br>(44/47)	ES781539<br>ES780922
sb_gmnllfta.124.C1	2	sb_gmnllfta_0005d24.t7<br>sb_gmnllfta_0006a23.t7	Cluster: Ribosomal protein S30; n=4; Percomorpha|Rep: Ribosomal protein S30 - Solea senegalensis (Sole)	GO:0006412<br>GO:0005840<br>GO:0005622<br>GO:0003735	6e-10	96%<br>(32/33)	ES781556<br>ES781642
sb_gmnllfta.125.C1	2	sb_gmnllfta_0007j17.t7<br>sb_gmnllfta_0006n23.t7	unclassified				FL634220<br>ES781379
sb_gmnllfta.127.C1	2	sb_gmnllfta_0006p23.t7<br>sb_gmnllfta_0001d01.t7	Cluster: Beta2-microglobulin precursor; n=3; Gadus morhua|Rep: Beta2-microglobulin precursor - Gadus morhua (Atlantic cod)		6e-32	100%<br>(64/64)	ES781346<br>ES781227
sb_gmnllfta.128.C1	2	sb_gmnllfta_0002c20.t7<br>sb_gmnllfta_0006b24.t7	Cluster: hydroxysteroid (17-beta) dehydrogenase 6; n=1; Takifugu rubripes|Rep: hydroxysteroid (17-beta) dehydrogenase 6 - Takifugu rubripes		1e-35	63%<br>(71/112)	ES781116<br>ES780964
sb_gmnllfta.13.C1	2	sb_gmnllfta_0002g09.t7<br>sb_gmnllfta_0007j02.t7	unclassified				ES781930<br>FL634208
sb_gmnllfta.132.C1	2	sb_gmnllfta_0003a24.t7<br>sb_gmnllfta_0006g14.t7	Cluster: Serotransferrin; n=1; Gadus morhua|Rep: Serotransferrin - Gadus morhua (Atlantic cod)	GO:0008199<br>GO:0006879<br>GO:0006826<br>GO:0006811<br>GO:0006810<br>GO:0005576<br>GO:0005506	1e-72	96%<br>(133/138)	ES780857<br>ES781856

sb_gmnllfta.133.C1	2	sb_gmnllfta_0002g03.t7<br>sb_gmnllfta_0007b06.t7	unclassified				ES781419<br>FL634081
sb_gmnllfta.134.C1	2	sb_gmnllfta_0007b18.t7<br>sb_gmnllfta_0006b21.t7	ApoC-I domain containing protein		1e-04	35%<br>(20/56)	FL634091<br>ES780942
sb_gmnllfta.135.C1	2	sb_gmnllfta_0005g06.t7<br>sb_gmnllfta_0006m20.t7	Cluster: PREDICTED: similar to 6-phosphofructokinase, liver type (Phosphofructokinase 1) (Phosphohexokinase) (Phosphofructo-1-kinase isozyme B) (PFK-B); n=2; Danio rerio|Rep: PREDICTED: similar to 6-phosphofructokinase, liver type (Phosphofructokinase 1) (Phosphohexokinase) (Phosphofructo-1-kinase isozyme B) (PFK-B) - Danio rerio		9e-26	71%<br>(50/70)	ES781395<br>ES781832
sb_gmnllfta.136.C1	2	sb_gmnllfta_0004c17.t7<br>sb_gmnllfta_0005d15.t7	Cluster: CWF19-like 1, cell cycle control; n=1; Takifugu rubripes|Rep: CWF19-like 1, cell cycle control - Takifugu rubripes		4e-53	89%<br>(98/110)	ES780845<br>ES780992
sb_gmnllfta.137.C1	2	sb_gmnllfta_0007e17.t7<br>sb_gmnllfta_0007f13.t7	Cluster: Zgc:92161; n=2; Danio rerio|Rep: Zgc:92161 - Danio rerio (Zebrafish) (Brachydanio rerio)		9e-13	58%<br>(32/55)	FL634138<br>FL634150
sb_gmnllfta.139.C1	2	sb_gmnllfta_0006d17.t7<br>sb_gmnllfta_0006p19.t7	Cluster: Homolog of Homo sapiens "Organic-cation transporter like 3; n=1; Takifugu rubripes|Rep: Homolog of Homo sapiens "Organic-cation transporter like 3 - Takifugu rubripes		3e-09	51%<br>(29/56)	ES781132<br>ES781418
sb_gmnllfta.14.C1	2	sb_gmnllfta_0004l12.t7<br>sb_gmnllfta_0005j08.t7	Cluster: Zgc:92606; n=3; Clupeocephala|Rep: Zgc:92606 - Danio rerio (Zebrafish) (Brachydanio rerio)		4e-14	79%<br>(39/49)	ES781977<br>ES781548
sb_gmnllfta.140.C1	2	sb_gmnllfta_0003e01.t7<br>sb_gmnllfta_0003f01.t7	Cluster: Heat shock cognate 71 kDa protein; n=25; Clupeocephala|Rep: Heat shock cognate 71 kDa protein - Danio rerio (Zebrafish) (Brachydanio rerio)		3e-45	78%<br>(93/118)	ES781842<br>ES781187
sb_gmnllfta.141.C1	2	sb_gmnllfta_0007n24.t7<br>sb_gmnllfta_0002o23.t7	unclassified				FL634289<br>ES781008
sb_gmnllfta.142.C1	2	sb_gmnllfta_0005j04.t7<br>sb_gmnllfta_0006l11.t7	Cluster: PREDICTED: CDC-like kinase 4 isoform 1; n=3; Danio rerio|Rep: PREDICTED: CDC-like kinase 4 isoform 1 - Danio rerio		1e-10	76%<br>(29/38)	ES781970<br>ES781616

sb_gmnllfta.144.C1	2	sb_gmnllfta_0006f14.t7<br>sb_gmnllfta_0006j06.t7	unclassified				ES780802<br>ES781503
sb_gmnllfta.145.C1	2	sb_gmnllfta_0007c08.t7<br>sb_gmnllfta_0004k06.t7	Unassigned protein				FL634098<br>ES781004
sb_gmnllfta.146.C1	2	sb_gmnllfta_0006c21.t7<br>sb_gmnllfta_0002a22.t7	Cluster: PREDICTED: similar to T-complex protein 1, alpha subunit (TCP-1-alpha) (CCT-alpha) isoform 7; n=1; Canis lupus familiaris|Rep: PREDICTED: similar to T-complex protein 1, alpha subunit (TCP-1-alpha) (CCT-alpha) isoform 7 - Canis familiaris		4e-28	70% (72/102)	ES781990<br>ES780862
sb_gmnllfta.147.C1	2	sb_gmnllfta_0006d11.t7<br>sb_gmnllfta_0006b07.t7	Cluster: Skin mucus lectin; n=2; Lophiomus setigerus|Rep: Skin mucus lectin - Lophiomus setigerus		3e-19	44% (50/112)	ES781302<br>ES781098
sb_gmnllfta.15.C1	2	sb_gmnllfta_0006m12.t7<br>sb_gmnllfta_0007l16.t7	unclassified				ES781133<br>FL634253
sb_gmnllfta.150.C1	2	sb_gmnllfta_0004k11.t7<br>sb_gmnllfta_0002j18.t7	Cluster: Protein disulfide isomerase-related protein; n=3; Danio rerio|Rep: Protein disulfide isomerase-related protein - Danio rerio (Zebrafish) (Brachydanio rerio)		1e-26	75% (60/80)	ES781974<br>ES781490
sb_gmnllfta.152.C1	2	sb_gmnllfta_0007f16.t7<br>sb_gmnllfta_0005k21.t7	unclassified				FL634152<br>ES781192
sb_gmnllfta.153.C1	2	sb_gmnllfta_0002n18.t7<br>sb_gmnllfta_0004h02.t7	Cluster: Cyclin-dependent kinases regulatory subunit 2; n=1; Xenopus laevis|Rep: Cyclin-dependent kinases regulatory subunit 2 - Xenopus laevis (African clawed frog)	GO:0016538<br>GO:0007049	2e-08	58% (17/29)	ES781054<br>ES781575
sb_gmnllfta.154.C1	2	sb_gmnllfta_0006i08.t7<br>sb_gmnllfta_0006o19.t7	unclassified				ES781286<br>ES781316
sb_gmnllfta.155.C1	2	sb_gmnllfta_0006j10.t7<br>sb_gmnllfta_0004p24.t7	unclassified				ES781148<br>ES780834

sb_gmnllfta.156.C1	2	sb_gmnllfta_0003j12.t7<br>sb_gmnllfta_0007d13.t7	Cluster: Superoxide dismutase [Cu-Zn]; n=4; Perciformes|Rep: Superoxide dismutase [Cu-Zn] - Epinephelus coioides (Orange-spotted grouper)	GO:0016491<br>GO:0008270<br>GO:0006801<br>GO:0005507<br>GO:0004785<br>GO:0004784	2e-64	83% (113/136)	ES781733<br>FL634118
sb_gmnllfta.157.C1	2	sb_gmnllfta_0005k10.t7<br>sb_gmnllfta_0006i03.t7	unclassified				ES781817<br>ES780938
sb_gmnllfta.157.C2	2	sb_gmnllfta_0005o23.t7<br>sb_gmnllfta_0005a13.t7	unclassified				ES781907<br>ES781111
sb_gmnllfta.159.C1	2	sb_gmnllfta_0005d05.t7<br>sb_gmnllfta_0003n21.t7	unclassified				ES781813<br>ES781973
sb_gmnllfta.16.C1	2	sb_gmnllfta_0007i11.t7<br>sb_gmnllfta_0007l05.t7	Cluster: Differentially regulated trout protein 1 precursor; n=2; Salmonidae|Rep: Differentially regulated trout protein 1 precursor - Salvelinus fontinalis (Brook trout) (Brook char)		6e-19	54% (40/73)	FL634199<br>FL634244
sb_gmnllfta.162.C1	2	sb_gmnllfta_0003j02.t7<br>sb_gmnllfta_0005l09.t7	unclassified				ES781072<br>ES781220
sb_gmnllfta.163.C1	2	sb_gmnllfta_0004g03.t7<br>sb_gmnllfta_0007b07.t7	Cluster: Scaffolding protein B; n=35; Enterobacteria phage phiX174 sensu lato|Rep: Scaffolding protein B - Bacteriophage phi-X174	GO:0019069	2e-40	98% (63/64)	ES781292<br>FL634082
sb_gmnllfta.164.C1	2	sb_gmnllfta_0005f14.t7<br>sb_gmnllfta_0006h13.t7	unclassified				ES781355<br>ES780865
sb_gmnllfta.165.C1	2	sb_gmnllfta_0002f10.t7<br>sb_gmnllfta_0002f22.t7	unclassified				ES781411<br>ES780918

sb_gmnllfta.166.C1	2	sb_gmnllfta_0001g04.t7<br>sb_gmnllfta_0001f09.t7	unclassified				ES781778<br>ES781289
sb_gmnllfta.167.C1	2	sb_gmnllfta_0004e06.t7<br>sb_gmnllfta_0006e14.t7	Cluster: Homolog of Homo sapiens "Apolipoprotein B-100 precursor; n=1; Takifugu rubripes|Rep: Homolog of Homo sapiens "Apolipoprotein B-100 precursor - Takifugu rubripes		1e-65	52% (127/243)	ES780787<br>ES781615
sb_gmnllfta.168.C1	2	sb_gmnllfta_0006o18.t7<br>sb_gmnllfta_0007e16.t7	unclassified				ES781257<br>FL634137
sb_gmnllfta.169.C1	2	sb_gmnllfta_0005h10.t7<br>sb_gmnllfta_0007o06.t7	Cluster: Zgc:136978; n=1; Danio rerio|Rep: Zgc:136978 - Danio rerio (Zebrafish) (Brachydanio rerio)		5e-31	53% (42/79)	ES781319<br>FL634293
sb_gmnllfta.170.C1	2	sb_gmnllfta_0004i07.t7<br>sb_gmnllfta_0003i07.t7	unclassified				ES781343<br>ES781627
sb_gmnllfta.171.C1	2	sb_gmnllfta_0006f05.t7<br>sb_gmnllfta_0006h16.t7	Cluster: Ribosomal protein Sa; n=2; Percomorpha|Rep: Ribosomal protein Sa - Solea senegalensis (Sole)	GO:0015935<br>GO:0006412<br>GO:0005840<br>GO:0005622<br>GO:0003735	1e-21	97% (46/47)	ES781993<br>ES781037
sb_gmnllfta.172.C1	2	sb_gmnllfta_0006l18.t7<br>sb_gmnllfta_0005i03.t7	Cluster: Phosphoglucomutase 1; n=2; Danio rerio|Rep: Phosphoglucomutase 1 - Danio rerio (Zebrafish) (Brachydanio rerio)		5e-15	92% (38/41)	ES781772<br>ES781293
sb_gmnllfta.173.C1	2	sb_gmnllfta_0003o24.t7<br>sb_gmnllfta_0004o24.t7	unclassified				ES780952<br>ES781838
sb_gmnllfta.174.C1	2	sb_gmnllfta_0003m09.t7<br>sb_gmnllfta_0006o08.t7	Unassigned protein				ES781203<br>ES781476
sb_gmnllfta.175.C1	2	sb_gmnllfta_0005m24.t7<br>sb_gmnllfta_0007h15.t7	Cluster: Type-4 ice-structuring protein precursor; n=1; Gadus morhua|Rep: Type-4 ice-structuring protein precursor - Gadus morhua (Atlantic cod)	GO:0050825<br>GO:0042309<br>GO:0042157<br>GO:0008289<br>GO:0006869	3e-06	100% (26/26)	ES781538<br>FL634188

				GO:0005576			
sb_gmnllfta.176.C1	2	sb_gmnllfta_0002h15.t7<br>sb_gmnllfta_0007e15.t7	unclassified				ES781501<br>FL634136
sb_gmnllfta.177.C1	2	sb_gmnllfta_0005f05.t7<br>sb_gmnllfta_0005k01.t7	Cluster: Mitochondrial ribosomal protein S23 (S23mt) (MRP-S23).; n=3; Takifugu rubripes|Rep: Mitochondrial ribosomal protein S23 (S23mt) (MRP-S23). - Takifugu rubripes		3e-24	56%<br>(51/91)	ES781443<br>ES780954
sb_gmnllfta.179.C1	2	sb_gmnllfta_0007f07.t7<br>sb_gmnllfta_0006l15.t7	unclassified				FL634147<br>ES781431
sb_gmnllfta.181.C1	2	sb_gmnllfta_0005g13.t7<br>sb_gmnllfta_0004p12.t7	unclassified				ES781638<br>ES781570
sb_gmnllfta.182.C1	2	sb_gmnllfta_0005f22.t7<br>sb_gmnllfta_0004i04.t7	unclassified				ES781805<br>ES781275
sb_gmnllfta.183.C1	2	sb_gmnllfta_0003e13.t7<br>sb_gmnllfta_0004e13.t7	Cluster: Transforming protein RhoA precursor; n=49; Euteleostomi|Rep: Transforming protein RhoA precursor - Homo sapiens (Human)		1e-64	95%<br>(117/122)	ES780843<br>ES781726
sb_gmnllfta.184.C1	2	sb_gmnllfta_0004c13.t7<br>sb_gmnllfta_0007b22.t7	unclassified				ES781085<br>FL634094
sb_gmnllfta.186.C1	2	sb_gmnllfta_0006h15.t7<br>sb_gmnllfta_0006m23.t7	unclassified				ES780959<br>ES781721
sb_gmnllfta.188.C1	2	sb_gmnllfta_0002j14.t7<br>sb_gmnllfta_0002k01.t7	Cluster: Ribosomal protein S2; n=5; Euteleostomi|Rep: Ribosomal protein S2 - Danio rerio (Zebrafish) (Brachydanio rerio)		1e-17	97%<br>(44/45)	ES781725<br>ES781844
sb_gmnllfta.189.C1	2	sb_gmnllfta_0007b08.t7<br>sb_gmnllfta_0007p13.t7	Unassigned protein				FL634083<br>FL634314

sb_gmnllfta.19.C1	2	sb_gmnllfta_0006b12.t7<br>sb_gmnllfta_0007l14.t7	Cluster: Putative iodothyronine deiodinase type 1; n=1; Sparus aurata|Rep: Putative iodothyronine deiodinase type 1 - Sparus aurata (Gilthead sea bream)	GO:0016491<br>GO:0008430<br>GO:0004800	7e-28	58% (60/102)	ES781454<br>FL634251
sb_gmnllfta.190.C1	2	sb_gmnllfta_0004j12.t7<br>sb_gmnllfta_0007c15.t7	Cluster: La ribonucleoprotein domain family member 2 isoform 1; n=1; Takifugu rubripes|Rep: La ribonucleoprotein domain family member 2 isoform 1 - Takifugu rubripes		1e-10	73% (31/42)	ES780836<br>FL634103
sb_gmnllfta.191.C1	2	sb_gmnllfta_0003g13.t7<br>sb_gmnllfta_0002n15.t7	LSU rRNA; Homo sapiens		9e-64	92% (173/188)	ES781217<br>ES781265
sb_gmnllfta.192.C1	2	sb_gmnllfta_0006n15.t7<br>sb_gmnllfta_0004g19.t7	Unassigned protein				ES781779<br>ES781910
sb_gmnllfta.193.C1	2	sb_gmnllfta_0003l17.t7<br>sb_gmnllfta_0004l17.t7	unclassified				ES780818<br>ES781704
sb_gmnllfta.195.C1	2	sb_gmnllfta_0002l16.t7<br>sb_gmnllfta_0004l16.t7	unclassified				ES780977<br>ES781740
sb_gmnllfta.196.C1	2	sb_gmnllfta_0005p16.t7<br>sb_gmnllfta_0007h19.t7	Cluster: Hemoglobin subunit alpha-2; n=3; Gadidae|Rep: Hemoglobin subunit alpha-2 - Gadus morhua (Atlantic cod)	GO:0020037<br>GO:0019825<br>GO:0015671<br>GO:0006810<br>GO:0005833<br>GO:0005506<br>GO:0005344	5e-29	100% (63/63)	ES781664<br>FL634190
sb_gmnllfta.197.C1	2	sb_gmnllfta_0003f20.t7<br>sb_gmnllfta_0004f20.t7	Cluster: Fc11b09; n=4; Danio rerio|Rep: Fc11b09 - Danio rerio (Zebrafish) (Brachydanio rerio)		8e-82	93% (147/157)	ES781003<br>ES781902
sb_gmnllfta.198.C1	2	sb_gmnllfta_0002i20.t7<br>sb_gmnllfta_0006c17.t7	Cluster: Zgc:66080; n=3; Danio rerio|Rep: Zgc:66080 - Danio rerio (Zebrafish) (Brachydanio rerio)		8e-17	80% (40/50)	ES781748<br>ES781175
sb_gmnllfta.199.C1	2	sb_gmnllfta_0005f24.t7	Cluster: PREDICTED: similar to protein tyrosine phosphatase, receptor type, B; n=1; Ornithorhynchus anatinus|Rep: PREDICTED: similar to protein tyrosine phosphatase, receptor type, B -		4e-29	77%	ES781950<br>ES781336

		sb_gmnllfta_0002p17.t7	Ornithorhynchus anatinus			(60/77)	
sb_gmnllfta.200.C1	2	sb_gmnllfta_0003a11.t7<br>sb_gmnllfta_0004a11.t7	Cluster: Eukaryotic translation initiation factor 3, subunit 6 48kDa; n=2; Danio rerio|Rep: Eukaryotic translation initiation factor 3, subunit 6 48kDa - Danio rerio (Zebrafish) (Brachydanio rerio)		1e-69	88%<br>(67/76)	ES781631<br>ES781358
sb_gmnllfta.21.C1	2	sb_gmnllfta_0007f18.t7<br>sb_gmnllfta_0006o12.t7	Cluster: Flavin-containing monooxygenase FMO1; n=1; Oncorhynchus mykiss|Rep: Flavin-containing monooxygenase FMO1 - Oncorhynchus mykiss (Rainbow trout) (Salmo gairdneri)	GO:0050660<br>GO:0031227<br>GO:0016491<br>GO:0006118<br>GO:0005792<br>GO:0004499<br>GO:0004497	5e-16	60%<br>(42/70)	FL634153<br>ES780895
sb_gmnllfta.23.C1	2	sb_gmnllfta_0006d14.t7<br>sb_gmnllfta_0006l04.t7	LOC784446; similar to 60S ribosomal protein L5		1e-09	82%<br>(28/34)	ES781177<br>ES781382
sb_gmnllfta.25.C1	2	sb_gmnllfta_0005b10.t7<br>sb_gmnllfta_0002d07.t7	unclassified				ES781874<br>ES780797
sb_gmnllfta.27.C1	2	sb_gmnllfta_0006i07.t7<br>sb_gmnllfta_0002g21.t7	Cluster: Fructose-bisphosphate aldolase; n=1; Poecilia reticulata|Rep: Fructose-bisphosphate aldolase - Poecilia reticulata (Guppy)	GO:0008152<br>GO:0006096<br>GO:0004332<br>GO:0003824	3e-20	91%<br>(45/49)	ES780987<br>ES781908
sb_gmnllfta.29.C1	2	sb_gmnllfta_0006b19.t7<br>sb_gmnllfta_0007l06.t7	DUF1777 multi-domain protein		6e-13	35%<br>(34/96)	ES781934<br>FL634245
sb_gmnllfta.31.C1	2	sb_gmnllfta_0004p01.t7<br>sb_gmnllfta_0004k19.t7	Cluster: Cation-transporting ATPase; n=2; Tetraodontidae|Rep: Cation-transporting ATPase - Tetraodon nigroviridis (Green puffer)	GO:0016787<br>GO:0016021<br>GO:0016020<br>GO:0015672<br>GO:0015662<br>GO:0015077<br>GO:0008152<br>GO:0006812<br>GO:0006810<br>GO:0005524<br>GO:0003824<br>GO:0000166	1e-41	75%<br>(91/121)	ES781102<br>ES781485
sb_gmnllfta.34.C1	2	sb_gmnllfta_0007j08.t7<br>sb_gmnllfta_0005k16.t7	unclassified				FL634214<br>ES781923

sb_gmnllfta.36.C1	2	sb_gmnllfta_0005f23.t7 sb_gmnllfta_0007p03.t7	Cluster: Fructose-bisphosphate aldolase; n=3; Actinopterygii|Rep: Fructose-bisphosphate aldolase - Polypterus senegalus (Senegal bichir)	GO:0008152 GO:0006096 GO:0004332 GO:0003824	8e-35	97% (69/71)	ES781797 FL634307
sb_gmnllfta.39.C1	2	sb_gmnllfta_0003d18.t7 sb_gmnllfta_0004h06.t7	Cluster: Si:ch211-20b12.1; n=4; Danio rerio|Rep: Si:ch211-20b12.1 - Danio rerio (Zebrafish) (Brachydanio rerio)		8e-26	64% (64/99)	ES781491 ES781535
sb_gmnllfta.40.C1	2	sb_gmnllfta_0005m13.t7 sb_gmnllfta_0006e16.t7	Cluster: Homolog of Brachydanio rerio "Eukaryotic translation elongation factor 1 beta 2.; n=1; Takifugu rubripes|Rep: Homolog of Brachydanio rerio "Eukaryotic translation elongation factor 1 beta 2. - Takifugu rubripes		4e-29	89% (60/67)	ES781084 ES781591
sb_gmnllfta.41.C1	2	sb_gmnllfta_0004l04.t7 sb_gmnllfta_0002k12.t7	Cluster: Zgc:110709; n=1; Danio rerio|Rep: Zgc:110709 - Danio rerio (Zebrafish) (Brachydanio rerio)		9e-45	72% (85/117)	ES780947 ES780815
sb_gmnllfta.44.C1	2	sb_gmnllfta_0006e09.t7 sb_gmnllfta_0007a18.t7	UQ_con domain containing protein		8e-05	24% (12/49)	ES780893 FL634073
sb_gmnllfta.45.C1	2	sb_gmnllfta_0004b14.t7 sb_gmnllfta_0007c07.t7	Cluster: X-box binding protein 1B; n=4; Danio rerio|Rep: X-box binding protein 1B - Danio rerio (Zebrafish) (Brachydanio rerio)		1e-11	47% (39/82)	ES781986 FL634097
sb_gmnllfta.47.C1	2	sb_gmnllfta_0005i19.t7 sb_gmnllfta_0004i08.t7	Cluster: 40S ribosomal protein S10; n=1; Oreochromis mossambicus|Rep: 40S ribosomal protein S10 - Oreochromis mossambicus (Mozambique tilapia) (Tilapia mossambica)		2e-14	66% (39/59)	ES781911 ES781087
sb_gmnllfta.48.C1	2	sb_gmnllfta_0006j20.t7 sb_gmnllfta_0007i20.t7	Cluster: Zgc:92639; n=2; Danio rerio|Rep: Zgc:92639 - Danio rerio (Zebrafish) (Brachydanio rerio)		2e-37	75% (76/101)	ES781719 FL634206
sb_gmnllfta.50.C1	2	sb_gmnllfta_0002j11.t7 sb_gmnllfta_0006m10.t7	Cluster: PREDICTED: similar to PAM; n=2; Danio rerio|Rep: PREDICTED: similar to PAM - Danio rerio		3e-15	66% (36/54)	ES781920 ES781235
sb_gmnllfta.52.C1	2	sb_gmnllfta_0002k18.t7 sb_gmnllfta_0006m19.t7	unclassified				ES781348 ES781097

sb_gmnllfta.55.C1	2	sb_gmnllfta_0004i18.t7<br>sb_gmnllfta_0002i18.t7	Cluster: Eukaryotic elongation factor 1 alpha; n=1; Gadus morhua|Rep: Eukaryotic elongation factor 1 alpha - Gadus morhua (Atlantic cod)		5e-24	100% (49/49)	ES781751<br>ES780953
sb_gmnllfta.60.C1	2	sb_gmnllfta_0007b16.t7<br>sb_gmnllfta_0007l12.t7	unclassified				FL634089<br>FL634249
sb_gmnllfta.66.C1	2	sb_gmnllfta_0005n06.t7<br>sb_gmnllfta_0005o24.t7	Unassigned protein				ES781117<br>ES781754
sb_gmnllfta.67.C1	2	sb_gmnllfta_0007k18.t7<br>sb_gmnllfta_0006g23.t7	Cluster: NADH dehydrogenase [ubiquinone] 1 alpha subcomplex subunit 6; n=2; Bos taurus|Rep: NADH dehydrogenase [ubiquinone] 1 alpha subcomplex subunit 6 - Bos taurus (Bovine)		4e-25	77% (52/67)	FL634238<br>ES781103
sb_gmnllfta.69.C1	2	sb_gmnllfta_0005d10.t7<br>sb_gmnllfta_0002l07.t7	Cluster: Complement component C4; n=2; Takifugu rubripes|Rep: Complement component C4 - Fugu rubripes (Japanese pufferfish) (Takifugu rubripes)	GO:0005515<br>GO:0004866	2e-20	38% (60/155)	ES780884<br>ES781807
sb_gmnllfta.70.C1	2	sb_gmnllfta_0006d24.t7<br>sb_gmnllfta_0004e24.t7	Cluster: Myeloperoxidase; n=1; Siniperca chuatsi|Rep: Myeloperoxidase - Siniperca chuatsi (Chinese perch)	GO:0006979<br>GO:0006118<br>GO:0004601	2e-09	42% (37/87)	ES781774<br>ES781297
sb_gmnllfta.72.C1	2	sb_gmnllfta_0006o11.t7<br>sb_gmnllfta_0004a07.t7	Cluster: COP9 signalosome complex subunit 4; n=25; Euteleostomi|Rep: COP9 signalosome complex subunit 4 - Homo sapiens (Human)		2e-11	97% (34/35)	ES780803<br>ES781583
sb_gmnllfta.74.C1	2	sb_gmnllfta_0007b17.t7<br>sb_gmnllfta_0007h01.t7	Cluster: Antithrombin precursor; n=2; Salmonidae|Rep: Antithrombin precursor - Salmo salar (Atlantic salmon)		1e-21	70% (48/68)	FL634090<br>FL634177
sb_gmnllfta.76.C1	2	sb_gmnllfta_0005l22.t7<br>sb_gmnllfta_0007a04.t7	unclassified				ES781190<br>FL634063
sb_gmnllfta.8.C1	2	sb_gmnllfta_0005h21.t7<br>sb_gmnllfta_0007m12.t7	RPS15A, LOC427675; ribosomal protein S15a; K02957 small subunit ribosomal protein S15Ae	GO:0006412<br>GO:0005840<br>GO:0005622<br>GO:0003735	7e-52	97% (99/102)	ES781852<br>FL634265

sb_gmnllfta.81.C1	2	sb_gmnllfta_0002i09.t7<br>sb_gmnllfta_0005d19.t7	Cluster: Cathepsin S; n=1; Fundulus heteroclitus|Rep: Cathepsin S - Fundulus heteroclitus (Killifish) (Mummichog)	GO:0008234<br>GO:0008233<br>GO:0006508<br>GO:0004197	2e-12	60%<br>(32/53)	ES781816<br>ES781341
sb_gmnllfta.82.C1	2	sb_gmnllfta_0004h23.t7<br>sb_gmnllfta_0007p17.t7	unclassified				ES781796<br>FL634315
sb_gmnllfta.84.C1	2	sb_gmnllfta_0004o13.t7<br>sb_gmnllfta_0006k17.t7	Cluster: Protein disulfide-isomerase A4 precursor (EC 5.3.4.1) (Protein ERp-72) (ERp72).; n=1; Takifugu rubripes|Rep: Protein disulfide-isomerase A4 precursor (EC 5.3.4.1) (Protein ERp-72) (ERp72). - Takifugu rubripes		4e-37	78%<br>(72/92)	ES781112<br>ES781456
sb_gmnllfta.87.C1	2	sb_gmnllfta_0005m17.t7<br>sb_gmnllfta_0007j11.t7	unclassified				ES780846<br>FL634216
sb_gmnllfta.88.C1	2	sb_gmnllfta_0005h22.t7<br>sb_gmnllfta_0006p16.t7	Cluster: Ribosomal protein L7; n=4; Danio rerio|Rep: Ribosomal protein L7 - Danio rerio (Zebrafish) (Brachydanio rerio)		7e-60	81%<br>(107/131)	ES781957<br>ES781737
sb_gmnllfta.9.C1	2	sb_gmnllfta_0005b11.t7<br>sb_gmnllfta_0006j03.t7	Cluster: ADP-ribosylation factor-like 6 interacting protein 1; n=1; Danio rerio|Rep: ADP-ribosylation factor-like 6 interacting protein 1 - Danio rerio (Zebrafish) (Brachydanio rerio)		7e-09	87%<br>(28/32)	ES781892<br>ES781595
sb_gmnllfta.91.C1	2	sb_gmnllfta_0006m15.t7<br>sb_gmnllfta_0004n10.t7	Cluster: PREDICTED: similar to Translocon-associated protein delta subunit precursor (TRAP-delta) (Signal sequence receptor delta subunit) (SSR-delta) isoform 1; n=2; Catarrhini|Rep: PREDICTED: similar to Translocon-associated protein delta subunit precursor (TRAP-delta) (Signal sequence receptor delta subunit) (SSR-delta) isoform 1 - Macaca mulatta		2e-37	68%<br>(43/63)	ES781284<br>ES781635
sb_gmnllfta.94.C1	2	sb_gmnllfta_0006j07.t7<br>sb_gmnllfta_0006p06.t7	Cluster: Zgc:153093; n=2; Danio rerio|Rep: Zgc:153093 - Danio rerio (Zebrafish) (Brachydanio rerio)		1e-90	81%<br>(156/191)	ES781522<br>ES781077
sb_gmnllfta.95.C1	2	sb_gmnllfta_0004m16.t7<br>sb_gmnllfta_0005k15.t7	Cluster: Aldehyde dehydrogenase 7 family, member A1; n=3; Xenopus|Rep: Aldehyde dehydrogenase 7 family, member A1 - Xenopus tropicalis (Western clawed frog) (Silurana tropicalis)	GO:0008152	2e-63	80%<br>(120/149)	ES780995<br>ES781872

sb_gmnllfta.98.C1	2	sb_gmnllfta_0002l04.t7<br>sb_gmnllfta_0005l19.t7	Cluster: DEAD (Asp-Glu-Ala-Asp) box polypeptide 39a; n=12; Euteleostomi|Rep: DEAD (Asp-Glu-Ala-Asp) box polypeptide 39a - Danio rerio (Zebrafish) (Brachydanio rerio)		9e-44	96% (85/88)	ES781745<br>ES781585
sb_gmnllfta.99.C1	2	sb_gmnllfta_0007g08.t7<br>sb_gmnllfta_0007k02.t7	Cluster: Homolog of Danio rerio "MID1 interacting protein 1; n=1; Takifugu rubripes|Rep: Homolog of Danio rerio "MID1 interacting protein 1 - Takifugu rubripes		8e-28	77% (60/77)	FL634164<br>FL634226
sb_gmnllfta_0001a02.t7	1		Cluster: Hemoglobin subunit alpha-1; n=3; Gadidae|Rep: Hemoglobin subunit alpha-1 - Gadus morhua (Atlantic cod)	GO:0005344<br>GO:0005506<br>GO:0005833<br>GO:0006810<br>GO:0015671<br>GO:0019825<br>GO:0020037<br>GO:0046872	1e-51	94% (99/105)	ES780832
sb_gmnllfta_0001a03.t7	1		Cluster: Ubiquitin-like protein 5; n=6; Euteleostomi|Rep: Ubiquitin-like protein 5 - Brachydanio rerio (Zebrafish) (Danio rerio)		4e-15	92% (36/39)	ES780804
sb_gmnllfta_0001a04.t7	1		unclassified				ES780968
sb_gmnllfta_0001a08.t7	1		unclassified				ES781384
sb_gmnllfta_0001b02.t7	1		unclassified				ES781658
sb_gmnllfta_0001b05.t7	1		Cluster: Plastin-2; n=2; Danio rerio|Rep: Plastin-2 - Brachydanio rerio (Zebrafish) (Danio rerio)		2e-98	85% (132/154)	ES781519
sb_gmnllfta_0001b06.t7	1		Cluster: Novel protein similar to human NADH dehydrogenase (Ubiquinone) 1 beta subcomplex, 11, 17.3kDa; n=2; Danio rerio|Rep: Novel protein similar to human NADH dehydrogenase (Ubiquinone) 1 beta subcomplex, 11, 17.3kDa - Brachydanio rerio (Zebrafish) (Danio rerio)		9e-31	64% (61/94)	ES781461
sb_gmnllfta_0001b10.t7	1		Cluster: Glutathione S-transferase M; n=3; Cyprinidae|Rep: Glutathione S-transferase M - Brachydanio rerio (Zebrafish) (Danio rerio)		4e-10	61% (32/52)	ES781176

sb_gmnllfta_0001b11.t7	1		LOC512908; similar to HCF, C1, VCAF, CFF=VP16 accessory protein host cell factor		2e-23	55% (64/116)	ES781238
sb_gmnllfta_0001b12.t7	1		unclassified				ES781155
sb_gmnllfta_0001c01.t7	1		NDUFA5; NADH dehydrogenase (ubiquinone) 1 alpha subcomplex, 5, 13kDa		3e-19	67% (41/61)	ES781161
sb_gmnllfta_0001c02.t7	1		unclassified				ES781232
sb_gmnllfta_0001c04.t7	1		unclassified				ES781365
sb_gmnllfta_0001c05.t7	1		Cluster: Protein phosphatase 1 regulatory subunit 1B; n=6; Eutheria|Rep: Protein phosphatase 1 regulatory subunit 1B - Homo sapiens (Human)		5e-06	63% (19/30)	ES781380
sb_gmnllfta_0001c06.t7	1		Cluster: Tubulin alpha 1; n=5; Euteleostomi|Rep: Tubulin alpha 1 - Thamnaconus modestus	GO:0003924 GO:0005198 GO:0005525 GO:0005874 GO:0007017 GO:0007018 GO:0043234 GO:0051258	2e-15	84% (42/50)	ES781311
sb_gmnllfta_0001c08.t7	1		Cluster: Complement component C3; n=1; Gadus morhua|Rep: Complement component C3 - Gadus morhua (Atlantic cod)		1e-65	96% (74/77)	ES781010
sb_gmnllfta_0001c10.t7	1		Cluster: Cathepsin L; n=1; Fundulus heteroclitus|Rep: Cathepsin L - Fundulus heteroclitus (Killifish) (Mummichog)	GO:0004197 GO:0006508 GO:0008233 GO:0008234 GO:0016787	2e-38	91% (41/45)	ES781613
sb_gmnllfta_0001c11.t7	1		Cluster: Homolog of Brachydanio rerio "Putative heparin-binding growth factor 1 (HBGF-1) (Fibroblast growth factor 1) (FGF-1) (Acidic fibroblast growth factor) (aFGF).; n=1; Takifugu rubripes|Rep: Homolog of Brachydanio rerio "Putative heparin-binding growth factor 1 (HBGF-1) (Fibroblast growth factor 1) (FGF-		2e-10	63% (23/36)	ES781665

			1) (Acidic fibroblast growth factor) (aFGF). - Takifugu rubripes				
sb_gmnllfta_0001d02.t7	1		unclassified				ES781157
sb_gmnllfta_0001d03.t7	1		unclassified				ES781101
sb_gmnllfta_0001d04.t7	1		unclassified				ES781260
sb_gmnllfta_0001d05.t7	1		Cluster: LOC398863 protein; n=5; Xenopus|Rep: LOC398863 protein - Xenopus laevis (African clawed frog)	GO:0000166 GO:0005524	7e-45	83% (66/79)	ES781309
sb_gmnllfta_0001d07.t7	1		unclassified				ES781367
sb_gmnllfta_0001d09.t7	1		unclassified				ES781040
sb_gmnllfta_0001d10.t7	1		unclassified				ES781567
sb_gmnllfta_0001e01.t7	1		unclassified				ES781656
sb_gmnllfta_0001e04.t7	1		unclassified				ES781406
sb_gmnllfta_0001e05.t7	1		unclassified				ES781458
sb_gmnllfta_0001e07.t7	1		unclassified				ES781508

sb_gmnllfta_0001e11.t7	1		Cluster: Ras-related protein M-Ras precursor; n=12; Tetrapoda|Rep: Ras-related protein M-Ras precursor - Homo sapiens (Human)		5e-05	52% (12/23)	ES781152
sb_gmnllfta_0001e12.t7	1		unclassified				ES781236
sb_gmnllfta_0001f01.t7	1		Unassigned protein				ES780829
sb_gmnllfta_0001f04.t7	1		unclassified				ES781034
sb_gmnllfta_0001f05.t7	1		unclassified				ES781093
sb_gmnllfta_0001f06.t7	1		unclassified				ES780980
sb_gmnllfta_0001f07.t7	1		Cluster: Secernin-3; n=1; Danio rerio|Rep: Secernin-3 - Brachydanio rerio (Zebrafish) (Danio rerio)		3e-11	61% (35/57)	ES780970
sb_gmnllfta_0001f10.t7	1		LSU rRNA; Oncorhynchus mykiss		1e-138	92% (347/376)	ES781938
sb_gmnllfta_0001f12.t7	1		Cluster: Homolog of Gallus gallus "Fibrinogen alpha/alpha-E chain precursor [Contains "Fibrinopeptide A].; n=1; Takifugu rubripes|Rep: Homolog of Gallus gallus "Fibrinogen alpha/alpha-E chain precursor [Contains "Fibrinopeptide A]. - Takifugu rubripes		2e-15	55% (35/63)	ES781915
sb_gmnllfta_0001g03.t7	1		Cluster: 40S ribosomal protein S8; n=2; Gillichthys mirabilis|Rep: 40S ribosomal protein S8 - Gillichthys mirabilis (Long-jawed mudsucker)	GO:0005622	2e-17	65% (45/69)	ES781935
sb_gmnllfta_0001g05.t7	1		Cluster: Glucose phosphate isomerase a; n=4; Danio rerio|Rep: Glucose phosphate isomerase a - Brachydanio rerio (Zebrafish) (Danio rerio)		1e-55	80% (105/131)	ES781830

sb_gmnllfta_0001g06.t7	1		Cluster: PREDICTED: similar to ribosomal protein L26e; n=1; Strongylocentrotus purpuratus|Rep: PREDICTED: similar to ribosomal protein L26e - Strongylocentrotus purpuratus		7e-21	68% (42/61)	ES781718
sb_gmnllfta_0001g07.t7	1		Cluster: High mobility group protein; n=3; Percomorpha|Rep: High mobility group protein - Pagrus major (Red sea bream) (Chrysophrys major)	GO:0000785 GO:0003677 GO:0005634 GO:0006355	8e-76	88% (136/153)	ES781701
sb_gmnllfta_0001g09.t7	1		unclassified				ES781436
sb_gmnllfta_0001g12.t7	1		unclassified				ES780868
sb_gmnllfta_0001h03.t7	1		unclassified				ES781096
sb_gmnllfta_0001h04.t7	1		Cluster: Heat shock protein HSP 90-beta; n=26; Euteleostomi|Rep: Heat shock protein HSP 90-beta - Homo sapiens (Human)		3e-17	97% (33/34)	ES780939
sb_gmnllfta_0001h06.t7	1		unclassified				ES780810
sb_gmnllfta_0001h09.t7	1		Cluster: PREDICTED: similar to NAP isoform 2; n=2; Theria|Rep: PREDICTED: similar to NAP isoform 2 - Monodelphis domestica		4e-13	81% (26/32)	ES781149
sb_gmnllfta_0001h10.t7	1		unclassified				ES781801
sb_gmnllfta_0001h11.t7	1		Cluster: Antithrombin precursor; n=2; Salmonidae|Rep: Antithrombin precursor - Salmo salar (Atlantic salmon)	GO:0004867	9e-07	75% (24/32)	ES781780
sb_gmnllfta_0002a08.t7	1		unclassified				ES780838

sb_gmnllfta_0002a10.t7	1		Cluster: Prmt1 protein; n=2; Danio rerio|Rep: Prmt1 protein - Brachydanio rerio (Zebrafish) (Danio rerio)		2e-58	92% (97/105)	ES781495
sb_gmnllfta_0002a12.t7	1		Cluster: MHC class Ia antigen; n=2; Gadus morhua|Rep: MHC class Ia antigen - Gadus morhua (Atlantic cod)	GO:0006955 GO:0016020 GO:0019882 GO:0042612	2e-11	96% (30/31)	ES781474
sb_gmnllfta_0002a14.t7	1		unclassified				ES781549
sb_gmnllfta_0002a15.t7	1		unclassified				ES781604
sb_gmnllfta_0002a19.t7	1		Cluster: Glucose transporter 2; n=1; Gadus morhua|Rep: Glucose transporter 2 - Gadus morhua (Atlantic cod)	GO:0005215 GO:0005351 GO:0005355 GO:0006810 GO:0008643 GO:0016020 GO:0016021	5e-16	100% (42/42)	ES781951
sb_gmnllfta_0002a20.t7	1		unclassified				ES780899
sb_gmnllfta_0002b06.t7	1		unclassified				ES781967
sb_gmnllfta_0002b10.t7	1		Cluster: Hydroxyproline-rich glycoprotein; n=3; Oryza sativa|Rep: Hydroxyproline-rich glycoprotein - Oryza sativa (Rice)		4e-06	51% (25/49)	ES780971
sb_gmnllfta_0002b14.t7	1		LSU rRNA; Latimeria chalumnae		8e-74	95% (184/193)	ES780880
sb_gmnllfta_0002b17.t7	1		Cluster: Homolog of Homo sapiens "Maltase-glucoamylase, intestinal; n=1; Takifugu rubripes|Rep: Homolog of Homo sapiens "Maltase-glucoamylase, intestinal - Takifugu rubripes		2e-35	55% (71/127)	ES780817

sb_gmnllfta_0002c13.t7	1		unclassified				ES781759
sb_gmnllfta_0002c14.t7	1		unclassified				ES781925
sb_gmnllfta_0002c19.t7	1		Cluster: Long neurotoxin homolog precursor; n=3; Naja|Rep: Long neurotoxin homolog precursor - Naja atra (Chinese cobra)	GO:0005576<br>GO:0007268<br>GO:0009405<br>GO:0030550<br>GO:0045211	5e-06	36% (31/86)	ES781560
sb_gmnllfta_0002d03.t7	1		unclassified				ES781048
sb_gmnllfta_0002d08.t7	1		Cluster: PREDICTED: similar to Rh type C glycoprotein1; n=2; Danio rerio|Rep: PREDICTED: similar to Rh type C glycoprotein1 - Danio rerio		9e-09	54% (35/64)	ES781165
sb_gmnllfta_0002d12.t7	1		unclassified				ES781732
sb_gmnllfta_0002d13.t7	1		unclassified				ES781710
sb_gmnllfta_0002d16.t7	1		unclassified				ES781976
sb_gmnllfta_0002d18.t7	1		unclassified				ES781669
sb_gmnllfta_0002d19.t7	1		Cluster: Hemoglobin subunit alpha-1; n=3; Gadidae|Rep: Hemoglobin subunit alpha-1 - Gadus morhua (Atlantic cod)	GO:0005344<br>GO:0005506<br>GO:0005833<br>GO:0006810<br>GO:0015671<br>GO:0019825<br>GO:0020037<br>GO:0046872	3e-13	51% (49/95)	ES781626

sb_gmnllfta_0002e01.t7	1		unclassified				ES781750
sb_gmnllfta_0002e13.t7	1		unclassified				ES780974
sb_gmnllfta_0002e14.t7	1		unclassified				ES780822
sb_gmnllfta_0002e19.t7	1		unclassified				ES781195
sb_gmnllfta_0002e24.t7	1		unclassified				ES781428
sb_gmnllfta_0002f02.t7	1		unclassified				ES781296
sb_gmnllfta_0002f06.t7	1		unclassified				ES781245
sb_gmnllfta_0002f07.t7	1		unclassified				ES781194
sb_gmnllfta_0002f09.t7	1		Unassigned protein				ES780883
sb_gmnllfta_0002f11.t7	1		Cluster: Phosphoenolpyruvate carboxykinase; n=1; Acanthopagrus schlegelii|Rep: Phosphoenolpyruvate carboxykinase - Acanthopagrus schlegeli (Black porgy)	GO:0004611 GO:0005525 GO:0006094 GO:0016301	2e-07	81% (27/33)	ES781470
sb_gmnllfta_0002f14.t7	1		unclassified				ES781637

sb_gmnllfta_0002f17.t7	1		Unassigned protein		7e-10	70% (31/44)	ES781553
sb_gmnllfta_0002f19.t7	1		unclassified				ES781853
sb_gmnllfta_0002f21.t7	1		Unassigned protein				ES780861
sb_gmnllfta_0002g11.t7	1		Cluster: Homolog of Cyprinus carpio "Alpha-2-macroglobulin-1.; n=2; Takifugu rubripes|Rep: Homolog of Cyprinus carpio "Alpha-2-macroglobulin-1. - Takifugu rubripes		9e-49	66% (91/136)	ES781327
sb_gmnllfta_0002g18.t7	1		Cluster: Ribosomal protein S2; n=43; Euteleostomi|Rep: Ribosomal protein S2 - Brachydanio rerio (Zebrafish) (Danio rerio)		2e-62	92% (76/82)	ES780852
sb_gmnllfta_0002h09.t7	1		Cluster: 60S ribosomal protein L8; n=35; Vertebrata|Rep: 60S ribosomal protein L8 - Homo sapiens (Human)		1e-25	90% (49/54)	ES781081
sb_gmnllfta_0002h24.t7	1		unclassified				ES780911
sb_gmnllfta_0002i04.t7	1		Unassigned protein				ES781439
sb_gmnllfta_0002i05.t7	1		unclassified				ES781426
sb_gmnllfta_0002i13.t7	1		Cluster: Homolog of Homo sapiens "Beta-2-glycoprotein I precursor; n=1; Takifugu rubripes|Rep: Homolog of Homo sapiens "Beta-2-glycoprotein I precursor - Takifugu rubripes		9e-57	60% (99/163)	ES781216
sb_gmnllfta_0002i15.t7	1		unclassified				ES781359

sb_gmnllfta_0002i17.t7	1		Unassigned protein	GO:0005506<br>GO:0005529<br>GO:0020037<br>GO:0046872<br>GO:0046914	1e-04	29% (40/135)	ES781321
sb_gmnllfta_0002i21.t7	1		unclassified				ES781682
sb_gmnllfta_0002j01.t7	1		Cluster: Apolipoprotein B; n=1; Salmo salar|Rep: Apolipoprotein B - Salmo salar (Atlantic salmon)		2e-05	37% (23/61)	ES780791
sb_gmnllfta_0002j06.t7	1		unclassified				ES780956
sb_gmnllfta_0002j08.t7	1		unclassified				ES781269
sb_gmnllfta_0002j21.t7	1		unclassified				ES781342
sb_gmnllfta_0002j22.t7	1		Cluster: 60S ribosomal protein L11; n=2; Percomorpha|Rep: 60S ribosomal protein L11 - Gillichthys mirabilis (Long-jawed mudsucker)	GO:0003735<br>GO:0005622<br>GO:0005840<br>GO:0006412<br>GO:0030529	3e-44	100% (89/89)	ES781274
sb_gmnllfta_0002j24.t7	1		Cluster: PREDICTED: similar to hect domain and RLD 5; n=2; Bos taurus|Rep: PREDICTED: similar to hect domain and RLD 5 - Bos taurus		2e-06	30% (29/95)	ES781140
sb_gmnllfta_0002k05.t7	1		unclassified				ES781795
sb_gmnllfta_0002k07.t7	1		unclassified				ES781742
sb_gmnllfta_0002k09.t7	1		Cluster: PREDICTED: similar to Rh type C glycoprotein1; n=2; Danio rerio|Rep: PREDICTED: similar to Rh type C glycoprotein1 -		3e-08	56%	ES781466

			Danio rerio			(30/53)	
sb_gmnllfta_0002k16.t7	1		unclassified				ES781024
sb_gmnllfta_0002k17.t7	1		unclassified				ES781079
sb_gmnllfta_0002k19.t7	1		Cluster: Homolog of Homo sapiens "chloride channel 6 isoform ClC-6a; n=1; Takifugu rubripes|Rep: Homolog of Homo sapiens "chloride channel 6 isoform ClC-6a - Takifugu rubripes		5e-11	44% (36/81)	ES781389
sb_gmnllfta_0002k24.t7	1		Cluster: 39S ribosomal protein L35, mitochondrial precursor; n=4; Catarrhini|Rep: 39S ribosomal protein L35, mitochondrial precursor - Homo sapiens (Human)		4e-12	80% (29/36)	ES781574
sb_gmnllfta_0002l02.t7	1		unclassified				ES781839
sb_gmnllfta_0002l06.t7	1		Cluster: Hemoglobin subunit beta-2; n=3; Gadidae|Rep: Hemoglobin subunit beta-2 - Gadus morhua (Atlantic cod)	GO:0005344 GO:0005506 GO:0005833 GO:0006810 GO:0015671 GO:0019825 GO:0020037 GO:0046872	2e-06	85% (23/27)	ES781792
sb_gmnllfta_0002l24.t7	1		unclassified				ES781673
sb_gmnllfta_0002m04.t7	1		unclassified				ES781000
sb_gmnllfta_0002m13.t7	1		unclassified				ES781987
sb_gmnllfta_0002m14.t7	1		Cluster: Homolog of Brachydanio rerio "Protein disulfide isomerase related protein (Calcium-binding protein, intestinal-related).; n=1; Takifugu rubripes|Rep: Homolog of Brachydanio rerio "Protein disulfide isomerase related protein (Calcium-binding protein,		2e-29	72% (62/86)	ES781820

			intestinal-related). - Takifugu rubripes					
sb_gmnllfta_0002m16.t7	1		Cluster: Guanine nucleotide-binding protein subunit beta 2-like 1; n=50; Craniata|Rep: Guanine nucleotide-binding protein subunit beta 2-like 1 - Homo sapiens (Human)		5e-67	89% (122/137)	ES781714
sb_gmnllfta_0002m17.t7	1		Cluster: Mitochondrial import inner membrane translocase subunit Tim8 A; n=3; Xenopus|Rep: Mitochondrial import inner membrane translocase subunit Tim8 A - Xenopus laevis (African clawed frog)	GO:0005739 GO:0006457 GO:0006605 GO:0006626 GO:0006810 GO:0008270 GO:0015031 GO:0016020 GO:0042719 GO:0045039 GO:0046872	5e-33	72% (68/94)	ES781729
sb_gmnllfta_0002m21.t7	1		unclassified				ES781271
sb_gmnllfta_0002n14.t7	1		unclassified				ES781324
sb_gmnllfta_0002n21.t7	1		Cluster: Homolog of Homo sapiens "Dual specificity protein kinase CLK4; n=1; Takifugu rubripes|Rep: Homolog of Homo sapiens "Dual specificity protein kinase CLK4 - Takifugu rubripes		1e-48	83% (61/73)	ES781784
sb_gmnllfta_0002o13.t7	1		unclassified				ES781610
sb_gmnllfta_0002o21.t7	1		Cluster: Hepcidin; n=1; Pseudosciaena crocea|Rep: Hepcidin - Pseudosciaena crocea (Croceine croaker)		8e-11	51% (19/37)	ES781045
sb_gmnllfta_0002p07.t7	1		unclassified				ES781405
sb_gmnllfta_0002p10.t7	1		MGC82841; MGC82841 protein; K02962 small subunit ribosomal protein S17e		2e-21	72% (53/73)	ES781174
sb_gmnllfta_0002p16.t7	1		Cluster: Dci protein; n=5; Danio rerio|Rep: Dci protein - Brachydanio		3e-10	74%	ES781383

			rerio (Zebrafish) (Danio rerio)			(29/39)	
sb_gmnllfta_0002p19.t7	1		unclassified				ES781031
sb_gmnllfta_0003a04.t7	1		unclassified				ES781393
sb_gmnllfta_0003a16.t7	1		unclassified				ES781499
sb_gmnllfta_0003a18.t7	1		Cluster: Plasminogen; n=1; Oryzias latipes|Rep: Plasminogen - Oryzias latipes (Medaka fish) (Japanese ricefish)	GO:0004252 GO:0004283 GO:0005509 GO:0006508 GO:0007596	7e-12	85% (29/34)	ES781790
sb_gmnllfta_0003a21.t7	1		unclassified				ES781047
sb_gmnllfta_0003b09.t7	1		unclassified				ES781511
sb_gmnllfta_0003b12.t7	1		unclassified				ES780874
sb_gmnllfta_0003b15.t7	1		unclassified				ES781019
sb_gmnllfta_0003b18.t7	1		Cluster: PREDICTED: similar to ribosomal protein L37; n=1; Monodelphis domestica|Rep: PREDICTED: similar to ribosomal protein L37 - Monodelphis domestica		7e-41	94% (75/79)	ES781277
sb_gmnllfta_0003c10.t7	1		unclassified				ES781895
sb_gmnllfta_0003c17.t7	1		unclassified				ES781728

sb_gmnllfta_0003c24.t7	1		Cluster: Homolog of Homo sapiens "Alanine--glyoxylate aminotransferase 2, mitochondrial precursor; n=1; Takifugu rubripes|Rep: Homolog of Homo sapiens "Alanine--glyoxylate aminotransferase 2, mitochondrial precursor - Takifugu rubripes		1e-17	93% (27/29)	ES781247
sb_gmnllfta_0003d07.t7	1		Cluster: Splicing factor, arginine/serine-rich 11; n=3; Cyprinidae|Rep: Splicing factor, arginine/serine-rich 11 - Brachydanio rerio (Zebrafish) (Danio rerio)		5e-19	69% (52/75)	ES780998
sb_gmnllfta_0003d10.t7	1		Cluster: 60S ribosomal protein L35; n=17; Tetrapoda|Rep: 60S ribosomal protein L35 - Rattus norvegicus (Rat)		1e-14	63% (37/58)	ES781984
sb_gmnllfta_0003d14.t7	1		unclassified				ES781724
sb_gmnllfta_0003d15.t7	1		unclassified				ES781716
sb_gmnllfta_0003d19.t7	1		unclassified				ES781530
sb_gmnllfta_0003e06.t7	1		unclassified				ES781685
sb_gmnllfta_0003e11.t7	1		unclassified				ES780878
sb_gmnllfta_0003e12.t7	1		Cluster: Zgc:86609; n=2; Danio rerio|Rep: Zgc:86609 - Brachydanio rerio (Zebrafish) (Danio rerio)		9e-45	90% (68/75)	ES780814
sb_gmnllfta_0003e16.t7	1		unclassified				ES781025
sb_gmnllfta_0003e19.t7	1		unclassified				ES781390

sb_gmnllfta_0003f04.t7	1		unclassified				ES781290
sb_gmnllfta_0003f08.t7	1		Cluster: PREDICTED: similar to ribosomal protein L24; n=1; Mus musculus|Rep: PREDICTED: similar to ribosomal protein L24 - Mus musculus		2e-16	70% (31/44)	ES781022
sb_gmnllfta_0003f17.t7	1		unclassified				ES781464
sb_gmnllfta_0003g05.t7	1		Cluster: Lipoprotein lipase; n=3; Danio rerio|Rep: Lipoprotein lipase - Brachydanio rerio (Zebrafish) (Danio rerio)		9e-25	42% (60/140)	ES781424
sb_gmnllfta_0003g11.t7	1		Ribosomal_L38e domain containing protein		9e-06	76% (16/21)	ES781107
sb_gmnllfta_0003h02.t7	1		Cluster: PREDICTED: similar to ribosomal protein S23; n=2; Homo/Pan/Gorilla group|Rep: PREDICTED: similar to ribosomal protein S23 - Homo sapiens		2e-34	100% (72/72)	ES781298
sb_gmnllfta_0003h03.t7	1		pdia4; protein disulfide isomerase associated 4	GO:0005509 GO:0005783 GO:0016853 GO:0045454	1e-42	78% (80/102)	ES781276
sb_gmnllfta_0003h04.t7	1		unclassified				ES781131
sb_gmnllfta_0003h09.t7	1		Cluster: Succinate dehydrogenase [ubiquinone] flavoprotein subunit A, mitochondrial precursor; n=3; Xenopus|Rep: Succinate dehydrogenase [ubiquinone] flavoprotein subunit A, mitochondrial precursor - Xenopus laevis (African clawed frog)	GO:0005739 GO:0006099 GO:0006118 GO:0006810 GO:0008177 GO:0016491 GO:0016627 GO:0050660	2e-96	83% (113/136)	ES780881
sb_gmnllfta_0003h11.t7	1		PON2; paraoxonase 2		4e-42	55% (92/165)	ES781471

sb_gmnllfta_0003h14.t7	1		Cluster: Annexin A2; n=1; Monopterus albus|Rep: Annexin A2 - Monopterus albus (Swamp eel)	GO:0004859 GO:0005509 GO:0005544 GO:0008092	5e-21	76% (43/56)	ES781636
sb_gmnllfta_0003i04.t7	1		unclassified				ES781561
sb_gmnllfta_0003i09.t7	1		NAP1L4; nucleosome assembly protein 1-like 4		3e-27	57% (62/108)	ES781929
sb_gmnllfta_0003i13.t7	1		Cluster: PREDICTED: similar to complement C3-H1, partial; n=2; Danio rerio|Rep: PREDICTED: similar to complement C3-H1, partial - Danio rerio		3e-16	57% (40/69)	ES781356
sb_gmnllfta_0003j04.t7	1		Cluster: Ribosomal protein L9-like protein; n=4; Eutheria|Rep: Ribosomal protein L9-like protein - Bos taurus (Bovine)		3e-11	49% (41/83)	ES780903
sb_gmnllfta_0003j05.t7	1		unclassified				ES780913
sb_gmnllfta_0003j11.t7	1		Cluster: PREDICTED: similar to Ribosomal protein L8 isoform 1; n=2; Eutheria|Rep: PREDICTED: similar to Ribosomal protein L8 isoform 1 - Pan troglodytes		7e-42	84% (86/102)	ES781824
sb_gmnllfta_0003j20.t7	1		Cluster: Hemoglobin subunit beta-2; n=3; Gadidae|Rep: Hemoglobin subunit beta-2 - Gadus morhua (Atlantic cod)	GO:0005344 GO:0005506 GO:0005833 GO:0006810 GO:0015671 GO:0019825 GO:0020037 GO:0046872	2e-27	81% (64/79)	ES781188
sb_gmnllfta_0003k14.t7	1		Cluster: PREDICTED: similar to Ribosomal protein L5b isoform 2; n=1; Danio rerio|Rep: PREDICTED: similar to Ribosomal protein L5b isoform 2 - Danio rerio		3e-14	87% (34/39)	ES780823
sb_gmnllfta_0003k24.t7	1		LOC489932; similar to androgen receptor coactivator ARA55		5e-09	64% (22/34)	ES781430

sb_gmnllfta_0003l09.t7	1		unclassified				ES781634
sb_gmnllfta_0003l11.t7	1		Cluster: Pol-like protein; n=1; Danio rerio|Rep: Pol-like protein - Brachydanio rerio (Zebrafish) (Danio rerio)		1e-39	38% (84/220)	ES780997
sb_gmnllfta_0003l21.t7	1		unclassified				ES781584
sb_gmnllfta_0003m15.t7	1		LSU rRNA; Neoceratodus forsteri	GO:0016491 GO:0050381	2e-71	91% (191/209)	ES781979
sb_gmnllfta_0003m20.t7	1		unclassified				ES781118
sb_gmnllfta_0003m23.t7	1		unclassified				ES781183
sb_gmnllfta_0003n02.t7	1		unclassified				ES781537
sb_gmnllfta_0003n22.t7	1		unclassified				ES781912
sb_gmnllfta_0003n23.t7	1		Cluster: Vitamin K-dependent gamma-glutamyl carboxylase; n=1; Opsanus tau|Rep: Vitamin K-dependent gamma-glutamyl carboxylase - Opsanus tau (Oyster toadfish)	GO:0008488 GO:0017187	2e-17	74% (43/58)	ES781840
sb_gmnllfta_0003o11.t7	1		Cluster: Homolog of Homo sapiens "BCSC-1 isoform c/f; n=3; Takifugu rubripes|Rep: Homolog of Homo sapiens "BCSC-1 isoform c/f - Takifugu rubripes		8e-63	62% (118/189)	ES781513
sb_gmnllfta_0003o13.t7	1		unclassified				ES781410

sb_gmnllfta_0003o14.t7	1		Unassigned protein		9e-05	45% (17/37)	ES781550
sb_gmnllfta_0003o15.t7	1		unclassified				ES781605
sb_gmnllfta_0003p11.t7	1		Cluster: 40S ribosomal protein S24; n=6; Euteleostomi|Rep: 40S ribosomal protein S24 - Oryzias latipes (Medaka fish) (Japanese ricefish)	GO:0000166 GO:0003735 GO:0005622 GO:0005840 GO:0006412 GO:0030529	3e-08	100% (21/21)	ES781333
sb_gmnllfta_0003p15.t7	1		Cluster: Homolog of Oncorhynchus mykiss "CD209-like protein.; n=1; Takifugu rubripes|Rep: Homolog of Oncorhynchus mykiss "CD209-like protein. - Takifugu rubripes		9e-16	40% (34/84)	ES781134
sb_gmnllfta_0003p17.t7	1		Cluster: PREDICTED: similar to reverse transcriptase-like; n=1; Strongylocentrotus purpuratus|Rep: PREDICTED: similar to reverse transcriptase-like - Strongylocentrotus purpuratus		1e-05	30% (34/112)	ES781234
sb_gmnllfta_0004a10.t7	1		Cluster: FK506 binding protein 5; n=1; Danio rerio|Rep: FK506 binding protein 5 - Brachydanio rerio (Zebrafish) (Danio rerio)		2e-58	63% (111/175)	ES781371
sb_gmnllfta_0004a21.t7	1		Ribosomal_S19e domain containing protein		1e-07	73% (19/26)	ES781953
sb_gmnllfta_0004a22.t7	1		unclassified				ES781847
sb_gmnllfta_0004b06.t7	1		unclassified				ES780898
sb_gmnllfta_0004b10.t7	1		LSU rRNA; Hydrolagus colliei		3e-95	94% (224/237)	ES781730
sb_gmnllfta_0004b11.t7	1		Cluster: Malate dehydrogenase 1b, NAD; n=3; Danio rerio|Rep: Malate dehydrogenase 1b, NAD - Brachydanio rerio (Zebrafish)		4e-28	84% (53/63)	ES781711

			(Danio rerio)				
sb_gmnllfta_0004b15.t7	1		Cluster: 3'-phosphoadenosine 5'-phosphosulfate synthase 2; n=1; Danio rerio|Rep: 3'-phosphoadenosine 5'-phosphosulfate synthase 2 - Brachydanio rerio (Zebrafish) (Danio rerio)		4e-35	83% (55/66)	ES781919
sb_gmnllfta_0004b20.t7	1		unclassified				ES781141
sb_gmnllfta_0004c06.t7	1		Unassigned protein				ES781943
sb_gmnllfta_0004c09.t7	1		unclassified				ES781653
sb_gmnllfta_0004c14.t7	1		Unassigned protein				ES780937
sb_gmnllfta_0004c15.t7	1		DUF1356 domain containing protein		1e-04	30% (15/49)	ES780872
sb_gmnllfta_0004c22.t7	1		unclassified				ES781625
sb_gmnllfta_0004c23.t7	1		Cluster: UPF0027 protein C22orf28; n=22; Euteleostomi|Rep: UPF0027 protein C22orf28 - Homo sapiens (Human)		2e-09	100% (30/30)	ES781672
sb_gmnllfta_0004d11.t7	1		Cluster: Complement component C3-3; n=1; Oncorhynchus mykiss|Rep: Complement component C3-3 - Oncorhynchus mykiss (Rainbow trout) (Salmo gairdneri)	GO:0004866 GO:0005576 GO:0006954 GO:0006956	2e-09	51% (21/41)	ES781023
sb_gmnllfta_0004d13.t7	1		unclassified				ES780988
sb_gmnllfta_0004d23.t7	1		unclassified				ES781576

sb_gmnllfta_0004e17.t7	1		unclassified				ES781982
sb_gmnllfta_0004e20.t7	1		Ribosomal_L41 domain containing protein		6e-10	92% (23/25)	ES781246
sb_gmnllfta_0004e21.t7	1		Cluster: Zgc:86896; n=3; Danio rerio|Rep: Zgc:86896 - Brachydanio rerio (Zebrafish) (Danio rerio)		4e-30	73% (62/84)	ES781193
sb_gmnllfta_0004e23.t7	1		PREDICTED: p8 protein (candidate of metastasis 1) [Macaca mulatta]		5e-09	63% (26/41)	ES781137
sb_gmnllfta_0004f10.t7	1		unclassified				ES781318
sb_gmnllfta_0004f15.t7	1		unclassified				ES781200
sb_gmnllfta_0004f21.t7	1		Cluster: 40S ribosomal protein S2-3; n=5; Arabidopsis thaliana|Rep: 40S ribosomal protein S2-3 - Arabidopsis thaliana (Mouse-ear cress)		1e-22	77% (55/71)	ES781851
sb_gmnllfta_0004f22.t7	1		Cluster: Novel protein similar to human angiopoietin-like ANGPTL; n=3; Xenopus tropicalis|Rep: Novel protein similar to human angiopoietin-like ANGPTL - Xenopus tropicalis (Western clawed frog) (Silurana tropicalis)	GO:0005102 GO:0007165	2e-24	45% (50/110)	ES781958
sb_gmnllfta_0004g05.t7	1		Cluster: PREDICTED: adaptor-related protein complex 2, mu 1 subunit isoform 9; n=18; Eutheria|Rep: PREDICTED: adaptor-related protein complex 2, mu 1 subunit isoform 9 - Pan troglodytes		8e-43	96% (83/86)	ES781122
sb_gmnllfta_0004g09.t7	1		Cluster: Homolog of Homo sapiens "Fibrinogen beta chain precursor; n=1; Takifugu rubripes|Rep: Homolog of Homo sapiens "Fibrinogen beta chain precursor - Takifugu rubripes		4e-17	86% (37/43)	ES780934
sb_gmnllfta_0004g13.t7	1		LSU rRNA; Homo sapiens		2e-56	88% (220/248)	ES781509

sb_gmnllfta_0004g18.t7	1		Cluster: similar to ribosomal protein S2 (predicted) (RGD1559516_predicted), mRNA; n=1; Rattus norvegicus|Rep: similar to ribosomal protein S2 (predicted) (RGD1559516_predicted), mRNA - Rattus norvegicus		2e-35	91% (52/57)	ES781843
sb_gmnllfta_0004g22.t7	1		unclassified				ES780904
sb_gmnllfta_0004h05.t7	1		Unassigned protein		1e-04	66% (16/24)	ES781444
sb_gmnllfta_0004h09.t7	1		Cluster: Succinate dehydrogenase complex, subunit A, flavoprotein; n=5; Euteleostomi|Rep: Succinate dehydrogenase complex, subunit A, flavoprotein - Brachydanio rerio (Zebrafish) (Danio rerio)		2e-27	88% (52/59)	ES781763
sb_gmnllfta_0004h14.t7	1		Cluster: Annexin A2; n=1; Monopterus albus|Rep: Annexin A2 - Monopterus albus (Swamp eel)	GO:0004859 GO:0005509 GO:0005544 GO:0008092	1e-14	38% (71/185)	ES781354
sb_gmnllfta_0004h15.t7	1		Cluster: Homolog of Danio rerio "MID1 interacting protein 1; n=1; Takifugu rubripes|Rep: Homolog of Danio rerio "MID1 interacting protein 1 - Takifugu rubripes		4e-11	47% (38/80)	ES781373
sb_gmnllfta_0004h16.t7	1		unclassified				ES781326
sb_gmnllfta_0004i09.t7	1		NAP1L4; nucleosome assembly protein 1-like 4		4e-21	60% (52/86)	ES781016
sb_gmnllfta_0004j01.t7	1		unclassified				ES781901
sb_gmnllfta_0004j09.t7	1		unclassified				ES781413
sb_gmnllfta_0004j13.t7	1		Cluster: Hemoglobin subunit beta-1; n=1; Boreogadus saida|Rep: Hemoglobin subunit beta-1 - Boreogadus saida (Polar cod)	GO:0005344 GO:0005506 GO:0005833	4e-17	73% (30/41)	ES780825

				GO:0006810<br>GO:0015671<br>GO:0019825<br>GO:0020037<br>GO:0046872			
sb_gmnllfta_0004j15.t7	1		Cluster: PREDICTED: similar to ZNF367; n=2; Gallus gallus|Rep: PREDICTED: similar to ZNF367 - Gallus gallus		2e-30	71% (64/89)	ES780993
sb_gmnllfta_0004j20.t7	1		unclassified				ES781492
sb_gmnllfta_0004j24.t7	1		Cluster: Serum lectin isoform 1 precursor; n=3; Verasper variegatus|Rep: Serum lectin isoform 1 precursor - Verasper variegatus (Spotted flounder)	GO:0005529	2e-28	43% (58/134)	ES781557
sb_gmnllfta_0004k04.t7	1		unclassified				ES781050
sb_gmnllfta_0004k14.t7	1		Cluster: PREDICTED: similar to Ribosomal protein L5b isoform 2; n=1; Danio rerio|Rep: PREDICTED: similar to Ribosomal protein L5b isoform 2 - Danio rerio		1e-05	61% (19/31)	ES781709
sb_gmnllfta_0004l14.t7	1		Cluster: Solute carrier family 35 member B1; n=1; Danio rerio|Rep: Solute carrier family 35 member B1 - Brachydanio rerio (Zebrafish) (Danio rerio)		3e-30	72% (54/74)	ES781760
sb_gmnllfta_0004m04.t7	1		Unassigned protein		7e-07	23% (35/147)	ES781678
sb_gmnllfta_0004m10.t7	1		Cluster: Hemoglobin subunit beta-2; n=3; Gadidae|Rep: Hemoglobin subunit beta-2 - Gadus morhua (Atlantic cod)	GO:0005344<br>GO:0005506<br>GO:0005833<br>GO:0006810<br>GO:0015671<br>GO:0019825<br>GO:0020037<br>GO:0046872	1e-12	97% (34/35)	ES780821
sb_gmnllfta_0004n05.t7	1		unclassified				ES781398

sb_gmnllfta_0004n09.t7	1		unclassified				ES780972
sb_gmnllfta_0004n19.t7	1		Cluster: Homolog of Homo sapiens "Apoptosis regulator; n=1; Takifugu rubripes|Rep: Homolog of Homo sapiens "Apoptosis regulator - Takifugu rubripes		1e-36	79% (69/87)	ES781786
sb_gmnllfta_0004n21.t7	1		Cluster: Isoform 2 of Q5RI56 ; n=3; Danio rerio|Rep: Isoform 2 of Q5RI56 - Brachydanio rerio (Zebrafish) (Danio rerio)		3e-27	57% (63/109)	ES781068
sb_gmnllfta_0004o08.t7	1		unclassified				ES781738
sb_gmnllfta_0004o19.t7	1		unclassified				ES781042
sb_gmnllfta_0004o21.t7	1		Cluster: Serotransferrin; n=1; Gadus morhua|Rep: Serotransferrin - Gadus morhua (Atlantic cod)	GO:0005506 GO:0005576 GO:0006810 GO:0006811 GO:0006826 GO:0006879 GO:0008199 GO:0046872	2e-23	62% (64/103)	ES781808
sb_gmnllfta_0004p16.t7	1		Cluster: Dci protein; n=5; Danio rerio|Rep: Dci protein - Brachydanio rerio (Zebrafish) (Danio rerio)		2e-16	74% (37/50)	ES781477
sb_gmnllfta_0004p22.t7	1		unclassified				ES780967
sb_gmnllfta_0004p23.t7	1		unclassified				ES780985
sb_gmnllfta_0005a01.t7	1		ncf1; neutrophil cytosolic factor 1	GO:0005515 GO:0006118 GO:0007154 GO:0035091	5e-23	53% (51/95)	ES781579

sb_gmnllfta_0005a03.t7	1		unclassified				ES781623
sb_gmnllfta_0005a04.t7	1		Cluster: Heat shock protein 90 beta; n=7; Euteleostomi|Rep: Heat shock protein 90 beta - Paralichthys olivaceus (Japanese flounder)	GO:0000166 GO:0005524 GO:0006457 GO:0051082	3e-28	94% (37/39)	ES781482
sb_gmnllfta_0005a06.t7	1		unclassified				ES781447
sb_gmnllfta_0005a07.t7	1		Cluster: Keratin, type I cytoskeletal 13; n=2; Oncorhynchus mykiss|Rep: Keratin, type I cytoskeletal 13 - Oncorhynchus mykiss (Rainbow trout) (Salmo gairdneri)	GO:0005198 GO:0005882	4e-10	55% (30/54)	ES781417
sb_gmnllfta_0005a14.t7	1		unclassified				ES781262
sb_gmnllfta_0005a16.t7	1		Cluster: Hemoglobin subunit alpha-2; n=3; Gadidae|Rep: Hemoglobin subunit alpha-2 - Gadus morhua (Atlantic cod)	GO:0005344 GO:0005506 GO:0005833 GO:0006810 GO:0015671 GO:0019825 GO:0020037 GO:0046872	1e-28	83% (64/77)	ES781377
sb_gmnllfta_0005a20.t7	1		unclassified				ES781793
sb_gmnllfta_0005a23.t7	1		unclassified				ES781683
sb_gmnllfta_0005a24.t7	1		unclassified				ES781837
sb_gmnllfta_0005b07.t7	1		unclassified				ES781046

sb_gmnllfta_0005b09.t7	1		unclassified				ES781353
sb_gmnllfta_0005b13.t7	1		unclassified				ES781926
sb_gmnllfta_0005b14.t7	1		LOC771528; similar to ribosomal protein L38		4e-08	100% (29/29)	ES781761
sb_gmnllfta_0005b18.t7	1		Cluster: Phosphogluconate hydrogenase; n=7; Clupeocephala|Rep: Phosphogluconate hydrogenase - Brachydanio rerio (Zebrafish) (Danio rerio)		3e-46	95% (92/96)	ES781449
sb_gmnllfta_0005b19.t7	1		unclassified				ES781415
sb_gmnllfta_0005b20.t7	1		Cluster: Translation elongation factor eEF-1 alpha chain; n=1; Anisakis simplex|Rep: Translation elongation factor eEF-1 alpha chain - Anisakis simplex (Herring worm)	GO:0000166 GO:0003746 GO:0003924 GO:0005525 GO:0005737 GO:0006412 GO:0006414	3e-24	96% (53/55)	ES781279
sb_gmnllfta_0005b23.t7	1		Cluster: Complement C3-1 [Contains: Complement C3 beta chain; Complement C3 alpha chain; C3a anaphylatoxin; Complement C3b alpha' chain; Complement C3c alpha' chain fragment 1; Complement C3dg fragment; Complement C3g fragment; Complement C3d fragment; Complement C3f fragment; Complement C3c alpha' chain fragment 2]; n=1; Oncorhynchus mykiss|Rep: Complement C3-1 [Contains: Complement C3 beta chain; Complement C3 alpha chain; C3a anaphylatoxin; Complement C3b alpha' chain; Complement C3c alpha' chain fragment 1; Complement C3dg fragment; Complement C3g fragment; Complement C3d fragment; Complement C3f fragment; Complement C3c alpha' chain fragment 2] - Oncorhynchus mykiss (Rainbow trout) (Salmo gairdneri)	GO:0004866 GO:0005576 GO:0006954 GO:0006955 GO:0006957 GO:0006958 GO:0045087	1e-10	64% (25/39)	ES781345
sb_gmnllfta_0005c05.t7	1		unclassified				ES781753
sb_gmnllfta_0005c06.t7	1		unclassified				ES781815

sb_gmnllfta_0005c08.t7	1		unclassified				ES781515
sb_gmnllfta_0005c09.t7	1		Cluster: Hemoglobin subunit beta-1; n=2; Gadidae|Rep: Hemoglobin subunit beta-1 - Gadus morhua (Atlantic cod)	GO:0005344<br>GO:0005506<br>GO:0005833<br>GO:0006810<br>GO:0015671<br>GO:0019825<br>GO:0020037<br>GO:0046872	1e-38	98% (78/79)	ES781494
sb_gmnllfta_0005c10.t7	1		unclassified				ES780819
sb_gmnllfta_0005c11.t7	1		rpl22l1; ribosomal protein L22-like 1; K02891 large subunit ribosomal protein L22e		1e-11	76% (32/42)	ES780839
sb_gmnllfta_0005c12.t7	1		Cluster: Serotransferrin; n=1; Gadus morhua|Rep: Serotransferrin - Gadus morhua (Atlantic cod)	GO:0005506<br>GO:0005576<br>GO:0006810<br>GO:0006811<br>GO:0006826<br>GO:0006879<br>GO:0008199<br>GO:0046872	6e-32	94% (67/71)	ES780930
sb_gmnllfta_0005c13.t7	1		Cluster: 3-hydroxyanthranilate 3,4-dioxygenase; n=1; Xenopus laevis|Rep: 3-hydroxyanthranilate 3,4-dioxygenase - Xenopus laevis (African clawed frog)	GO:0000334<br>GO:0005506<br>GO:0008152<br>GO:0016491<br>GO:0016702<br>GO:0046872	1e-13	54% (36/66)	ES780879
sb_gmnllfta_0005c15.t7	1		unclassified				ES781090
sb_gmnllfta_0005c16.t7	1		unclassified				ES780994
sb_gmnllfta_0005c17.t7	1		unclassified				ES780973

sb_gmnllfta_0005c21.t7	1		unclassified				ES781442
sb_gmnllfta_0005d02.t7	1		zf-C2H2 domain containing protein		3e-05	47% (11/23)	ES781966
sb_gmnllfta_0005d11.t7	1		unclassified				ES780927
sb_gmnllfta_0005d13.t7	1		rpl11; ribosomal protein L11		1e-35	98% (76/77)	ES780824
sb_gmnllfta_0005d14.t7	1		Cluster: Liver angiotensinogen; n=1; Rhabdosargus sarba|Rep: Liver angiotensinogen - Rhabdosargus sarba (goldlined seabream)	GO:0004867	8e-56	65% (112/170)	ES780975
sb_gmnllfta_0005d22.t7	1		Cluster: Homolog of Brachydanio rerio "Cyclin I.; n=1; Takifugu rubripes|Rep: Homolog of Brachydanio rerio "Cyclin I. - Takifugu rubripes		5e-55	57% (114/197)	ES781438
sb_gmnllfta_0005e02.t7	1		unclassified				ES780912
sb_gmnllfta_0005e04.t7	1		unclassified				ES781051
sb_gmnllfta_0005e05.t7	1		Cluster: PREDICTED: similar to ATP-binding cassette, sub-family D (ALD), member 3a; n=2; Danio rerio|Rep: PREDICTED: similar to ATP-binding cassette, sub-family D (ALD), member 3a - Danio rerio		7e-59	93% (107/114)	ES781070
sb_gmnllfta_0005e07.t7	1		LSU rRNA; Squalus acanthias		1e-110	99% (201/202)	ES780949
sb_gmnllfta_0005e11.t7	1		Cluster: Novel protein; n=3; Danio rerio|Rep: Novel protein - Brachydanio rerio (Zebrafish) (Danio rerio)		1e-25	55% (52/94)	ES781975

sb_gmnllfta_0005e12.t7	1		Cluster: Zgc:77665 protein; n=2; Metazoa|Rep: Zgc:77665 protein - Brachydanio rerio (Zebrafish) (Danio rerio)		3e-07	96% (24/25)	ES781889
sb_gmnllfta_0005e13.t7	1		Unassigned protein	GO:0000166 GO:0005198 GO:0005525 GO:0005874 GO:0007017 GO:0007018	3e-05	100% (16/16)	ES781877
sb_gmnllfta_0005e14.t7	1		unclassified				ES781708
sb_gmnllfta_0005e15.t7	1		unclassified				ES781734
sb_gmnllfta_0005e16.t7	1		unclassified				ES781822
sb_gmnllfta_0005e17.t7	1		unclassified				ES781766
sb_gmnllfta_0005e18.t7	1		Daxx domain containing protein		1e-04	75% (18/24)	ES781534
sb_gmnllfta_0005e22.t7	1		Cluster: PREDICTED: similar to integrin alpha M; n=1; Danio rerio|Rep: PREDICTED: similar to integrin alpha M - Danio rerio		1e-15	41% (43/103)	ES781291
sb_gmnllfta_0005e23.t7	1		Cluster: Rhamnose binding lectin STL2; n=1; Oncorhynchus mykiss|Rep: Rhamnose binding lectin STL2 - Oncorhynchus mykiss (Rainbow trout) (Salmo gairdneri)	GO:0005529 GO:0016020 GO:0016524	2e-23	54% (50/91)	ES781282
sb_gmnllfta_0005e24.t7	1		Cluster: Prostaglandin E synthase 3; n=2; Danio rerio|Rep: Prostaglandin E synthase 3 - Brachydanio rerio (Zebrafish) (Danio rerio)		4e-62	71% (109/152)	ES781125
sb_gmnllfta_0005f02.t7	1		Cluster: Homolog of Homo sapiens "ARMC6 protein; n=1; Takifugu rubripes|Rep: Homolog of Homo sapiens "ARMC6 protein - Takifugu		2e-53	62% (111/179)	ES781573

			rubripes				
sb_gmnllfta_0005f03.t7	1		unclassified				ES781563
sb_gmnllfta_0005f08.t7	1		Cluster: Homolog of Brachydanio rerio "Ceruloplasmin.; n=1; Takifugu rubripes|Rep: Homolog of Brachydanio rerio "Ceruloplasmin. - Takifugu rubripes		2e-45	78% (83/106)	ES781825
sb_gmnllfta_0005f11.t7	1		Cluster: PREDICTED: similar to 40S ribosomal protein S26; n=1; Rattus norvegicus|Rep: PREDICTED: similar to 40S ribosomal protein S26 - Rattus norvegicus		5e-12	69% (37/53)	ES781164
sb_gmnllfta_0005f20.t7	1		unclassified				ES781686
sb_gmnllfta_0005g04.t7	1		Cluster: PREDICTED: similar to Chain A, Crystal Structure Of Human Choline Kinase Alpha 2; n=1; Danio rerio|Rep: PREDICTED: similar to Chain A, Crystal Structure Of Human Choline Kinase Alpha 2 - Danio rerio		2e-46	70% (93/131)	ES781273
sb_gmnllfta_0005g09.t7	1		unclassified				ES781017
sb_gmnllfta_0005g16.t7	1		Cluster: Homolog of Homo sapiens "Ubiquinol-cytochrome c reductase complex 7.2 kDa protein; n=1; Takifugu rubripes|Rep: Homolog of Homo sapiens "Ubiquinol-cytochrome c reductase complex 7.2 kDa protein - Takifugu rubripes		3e-10	74% (26/35)	ES781472
sb_gmnllfta_0005h04.t7	1		unclassified				ES781582
sb_gmnllfta_0005h09.t7	1		unclassified				ES781983
sb_gmnllfta_0005h11.t7	1		Unassigned protein				ES781270

sb_gmnllfta_0005h14.t7	1		unclassified					ES781218
sb_gmnllfta_0005h16.t7	1		unclassified					ES781110
sb_gmnllfta_0005h19.t7	1		Cluster: PREDICTED: similar to Cytochrome c oxidase polypeptide VIIc, mitochondrial precursor; n=1; Macaca mulatta|Rep: PREDICTED: similar to Cytochrome c oxidase polypeptide VIIc, mitochondrial precursor - Macaca mulatta		4e-07	69% (18/26)	ES780859
sb_gmnllfta_0005i01.t7	1		unclassified					ES781349
sb_gmnllfta_0005i07.t7	1		Unassigned protein					ES781249
sb_gmnllfta_0005i15.t7	1		RCJMB04_13o11, LACTB; lactamase, beta		2e-42	56% (92/162)	ES781633
sb_gmnllfta_0005i16.t7	1		rpl22l1; ribosomal protein L22-like 1; K02891 large subunit ribosomal protein L22e		9e-14	73% (25/34)	ES781546
sb_gmnllfta_0005i21.t7	1		unclassified					ES780798
sb_gmnllfta_0005i22.t7	1		Cluster: Homolog of Homo sapiens "dodecenoyl-Coenzyme A delta isomerase (3,2 trans-enoyl-Coenzyme A isomerase); n=1; Takifugu rubripes|Rep: Homolog of Homo sapiens "dodecenoyl-Coenzyme A delta isomerase (3,2 trans-enoyl-Coenzyme A isomerase) - Takifugu rubripes		5e-42	78% (77/98)	ES780902
sb_gmnllfta_0005i23.t7	1		unclassified					ES780914
sb_gmnllfta_0005j01.t7	1		Cluster: Alpha-aminoadipic semialdehyde dehydrogenase; n=12; Eutheria|Rep: Alpha-aminoadipic semialdehyde dehydrogenase - Homo sapiens (Human)		5e-13	41% (47/114)	ES781689

sb_gmnllfta_0005j06.t7	1		Cluster: Zgc:153186; n=2; Danio rerio|Rep: Zgc:153186 - Brachydanio rerio (Zebrafish) (Danio rerio)		3e-17	81% (43/53)	ES781846
sb_gmnllfta_0005j10.t7	1		Unassigned protein		5e-32	61% (66/108)	ES781080
sb_gmnllfta_0005j15.t7	1		Cluster: Homolog of Oncorhynchus mykiss "Rhamnose binding lectin STL2.; n=1; Takifugu rubripes|Rep: Homolog of Oncorhynchus mykiss "Rhamnose binding lectin STL2. - Takifugu rubripes		3e-14	64% (25/39)	ES780816
sb_gmnllfta_0005j16.t7	1		Cluster: Hemoglobin subunit alpha-2; n=3; Gadidae|Rep: Hemoglobin subunit alpha-2 - Gadus morhua (Atlantic cod)	GO:0005344 GO:0005506 GO:0005833 GO:0006810 GO:0015671 GO:0019825 GO:0020037 GO:0046872	4e-22	75% (55/73)	ES780875
sb_gmnllfta_0005j21.t7	1		Cluster: NADPH oxidase flavocytochrome b small subunit p22phox; n=1; Oncorhynchus mykiss|Rep: NADPH oxidase flavocytochrome b small subunit p22phox - Oncorhynchus mykiss (Rainbow trout) (Salmo gairdneri)	GO:0006118 GO:0020037	4e-21	68% (48/70)	ES781628
sb_gmnllfta_0005j22.t7	1		unclassified				ES781562
sb_gmnllfta_0005j24.t7	1		Cluster: 60S ribosomal protein L9; n=4; Euteleostomi|Rep: 60S ribosomal protein L9 - Ictalurus punctatus (Channel catfish)	GO:0003735 GO:0005622 GO:0005840 GO:0006412 GO:0030529	2e-19	86% (50/58)	ES781446
sb_gmnllfta_0005k02.t7	1		unclassified				ES781057
sb_gmnllfta_0005k09.t7	1		Cluster: Dynein light chain roadblock-type 2; n=9; Tetrapoda|Rep: Dynein light chain roadblock-type 2 - Homo sapiens (Human)		8e-06	63% (26/41)	ES781171
sb_gmnllfta_0005k12.t7	1		unclassified				ES781715

sb_gmnllfta_0005k14.t7	1		unclassified				ES781894
sb_gmnllfta_0005k17.t7	1		LSU rRNA; Latimeria chalumnae		1e-94	97% (188/192)	ES781981
sb_gmnllfta_0005k20.t7	1		LSU rRNA; Latimeria chalumnae		5e-97	94% (251/267)	ES781244
sb_gmnllfta_0005k22.t7	1		Cluster: 60S ribosomal protein L23; n=29; Coelomata|Rep: 60S ribosomal protein L23 - Homo sapiens (Human)		4e-51	98% (101/103)	ES781130
sb_gmnllfta_0005k24.t7	1		Cluster: Hemoglobin subunit alpha-1; n=3; Gadidae|Rep: Hemoglobin subunit alpha-1 - Gadus morhua (Atlantic cod)	GO:0005344 GO:0005506 GO:0005833 GO:0006810 GO:0015671 GO:0019825 GO:0020037 GO:0046872	3e-51	95% (99/104)	ES781299
sb_gmnllfta_0005l02.t7	1		Cytochrome c oxidase subunit 4 isoform 1, mitochondrial precursor (Cytochrome c oxidase subunit IV isoform 1) (COX IV-1) gb|AAF79933.1|AF204870_1 cytochrome c oxidase subunit IV isoform 1 precursor [Thunnus obesus]		8e-40	50% (80/160)	ES780951
sb_gmnllfta_0005l03.t7	1		LOC771344; similar to Sm protein G		1e-30	88% (63/71)	ES781001
sb_gmnllfta_0005l05.t7	1		unclassified				ES780785
sb_gmnllfta_0005l08.t7	1		unclassified				ES781199
sb_gmnllfta_0005l12.t7	1		Cluster: ATPase, H+ transporting, lysosomal accessory protein 2; n=3; Danio rerio|Rep: ATPase, H+ transporting, lysosomal accessory protein 2 - Brachydanio rerio (Zebrafish) (Danio rerio)		6e-84	73% (159/216)	ES781768

sb_gmnllfta_0005l15.t7	1		LSU rRNA; Xenopus borealis		1e-134	95% (314/330)	ES781918
sb_gmnllfta_0005l18.t7	1		unclassified				ES781555
sb_gmnllfta_0005l24.t7	1		Cluster: 60S ribosomal protein L24; n=19; Tetrapoda|Rep: 60S ribosomal protein L24 - Homo sapiens (Human)		5e-12	51% (41/79)	ES781399
sb_gmnllfta_0005m01.t7	1		unclassified				ES781789
sb_gmnllfta_0005m05.t7	1		Cluster: 40S ribosomal protein S10; n=1; Oreochromis mossambicus|Rep: 40S ribosomal protein S10 - Oreochromis mossambicus (Mozambique tilapia) (Tilapia mossambica)		1e-10	60% (26/43)	ES781841
sb_gmnllfta_0005m12.t7	1		Cluster: Hemoglobin subunit alpha-1; n=3; Gadidae|Rep: Hemoglobin subunit alpha-1 - Gadus morhua (Atlantic cod)	GO:0005344 GO:0005506 GO:0005833 GO:0006810 GO:0015671 GO:0019825 GO:0020037 GO:0046872	2e-25	92% (58/63)	ES781018
sb_gmnllfta_0005m14.t7	1		Cluster: 60S ribosomal protein L11 (CLL-associated antigen KW-12).; n=1; Canis familiaris|Rep: 60S ribosomal protein L11 (CLL-associated antigen KW-12). - Canis familiaris		1e-23	88% (39/44)	ES780936
sb_gmnllfta_0005m18.t7	1		Cluster: Homolog of Homo sapiens "Fibrinogen beta chain precursor; n=1; Takifugu rubripes|Rep: Homolog of Homo sapiens "Fibrinogen beta chain precursor - Takifugu rubripes		3e-80	73% (134/182)	ES781120
sb_gmnllfta_0005m20.t7	1		Cluster: Elongation factor 1-gamma; n=7; Clupeocephala|Rep: Elongation factor 1-gamma - Brachydanio rerio (Zebrafish) (Danio rerio)		3e-06	76% (20/26)	ES781581
sb_gmnllfta_0005n04.t7	1		Unassigned protein		2e-07	54% (29/53)	ES781253

sb_gmnllfta_0005n10.t7	1		unclassified				ES781510
sb_gmnllfta_0005n11.t7	1		unclassified				ES781500
sb_gmnllfta_0005n13.t7	1		unclassified				ES781468
sb_gmnllfta_0005n15.t7	1		unclassified				ES781545
sb_gmnllfta_0005n16.t7	1		Cluster: Transcribed locus, weakly similar to XP_518227.1 desmoplakin [Pan troglodytes]; n=1; Takifugu rubripes|Rep: Transcribed locus, weakly similar to XP_518227.1 desmoplakin [Pan troglodytes] - Takifugu rubripes		4e-10	78% (29/37)	ES781632
sb_gmnllfta_0005n18.t7	1		unclassified				ES781944
sb_gmnllfta_0005n19.t7	1		Cluster: Elongation factor 1-gamma; n=7; Clupeocephala|Rep: Elongation factor 1-gamma - Brachydanio rerio (Zebrafish) (Danio rerio)		3e-22	50% (60/118)	ES781972
sb_gmnllfta_0005n20.t7	1		LSU rRNA; Latimeria chalumnae		5e-94	97% (187/191)	ES780917
sb_gmnllfta_0005n21.t7	1		unclassified				ES780901
sb_gmnllfta_0005n22.t7	1		Cluster: Putative ribosomal protein S8; n=1; Oncorhynchus mykiss|Rep: Putative ribosomal protein S8 - Oncorhynchus mykiss (Rainbow trout) (Salmo gairdneri)	GO:0005622 GO:0030529	8e-08	100% (24/24)	ES780793
sb_gmnllfta_0005o02.t7	1		unclassified				ES781486

sb_gmnllfta_0005o03.t7	1		unclassified				ES781533
sb_gmnllfta_0005o08.t7	1		Unassigned protein				ES781870
sb_gmnllfta_0005o13.t7	1		unclassified				ES781323
sb_gmnllfta_0005o14.t7	1		unclassified				ES781172
sb_gmnllfta_0005o17.t7	1		dck; dck protein		4e-12	60% (31/51)	ES781222
sb_gmnllfta_0005p05.t7	1		Cluster: Hemoglobin subunit beta-2; n=3; Gadidae|Rep: Hemoglobin subunit beta-2 - Gadus morhua (Atlantic cod)	GO:0005344 GO:0005506 GO:0005833 GO:0006810 GO:0015671 GO:0019825 GO:0020037 GO:0046872	5e-21	62% (61/97)	ES781231
sb_gmnllfta_0005p12.t7	1		unclassified				ES781453
sb_gmnllfta_0005p13.t7	1		unclassified				ES781434
sb_gmnllfta_0005p17.t7	1		unclassified				ES781611
sb_gmnllfta_0005p19.t7	1		Cluster: Pancreatic secretory trypsin inhibitor precursor (Tumor-associated trypsin inhibitor) (TATI) (Serine protease inhibitor Kazal-type 1).; n=1; Gallus gallus|Rep: Pancreatic secretory trypsin inhibitor precursor (Tumor-associated trypsin inhibitor) (TATI) (Serine protease inhibitor Kazal-type 1). - Gallus gallus		1e-06	56% (23/41)	ES781931

sb_gmnllfta_0005p22.t7	1		Cluster: Ribosomal protein S30; n=3; Percomorpha|Rep: Ribosomal protein S30 - Solea senegalensis (Sole)	GO:0003735 GO:0005622 GO:0005840 GO:0006412 GO:0006464	1e-19	85% (42/49)	ES780828
sb_gmnllfta_0006a01.t7	1		Cluster: Ribosomal protein S30; n=3; Percomorpha|Rep: Ribosomal protein S30 - Solea senegalensis (Sole)	GO:0003735 GO:0005622 GO:0005840 GO:0006412 GO:0006464	3e-21	86% (44/51)	ES781833
sb_gmnllfta_0006a05.t7	1		Cluster: Serotransferrin; n=1; Gadus morhua|Rep: Serotransferrin - Gadus morhua (Atlantic cod)	GO:0005506 GO:0005576 GO:0006810 GO:0006811 GO:0006826 GO:0006879 GO:0008199 GO:0046872	2e-41	100% (81/81)	ES781884
sb_gmnllfta_0006a06.t7	1		Cluster: Homolog of Brachydanio rerio "Glycoprotein, synaptic 2.; n=1; Takifugu rubripes|Rep: Homolog of Brachydanio rerio "Glycoprotein, synaptic 2. - Takifugu rubripes		2e-20	64% (22/34)	ES781997
sb_gmnllfta_0006a07.t7	1		unclassified				ES781932
sb_gmnllfta_0006a08.t7	1		unclassified				ES781666
sb_gmnllfta_0006a09.t7	1		Cluster: PREDICTED: similar to 40S ribosomal protein S17; n=1; Canis familiaris|Rep: PREDICTED: similar to 40S ribosomal protein S17 - Canis familiaris		8e-18	91% (32/35)	ES781612
sb_gmnllfta_0006a11.t7	1		unclassified				ES781011
sb_gmnllfta_0006a14.t7	1		SLC7A2, ATRC2; solute carrier family 7 (cationic amino acid transporter, y+ system), member 2		2e-30	31% (72/226)	ES780891

sb_gmnllfta_0006a18.t7	1		unclassified				ES781160
sb_gmnllfta_0006a21.t7	1		unclassified				ES781597
sb_gmnllfta_0006a24.t7	1		Cluster: Alpha tubulin; n=6; Bilateria|Rep: Alpha tubulin - Notothenia coriiceps (black rockcod)	GO:0003924 GO:0005198 GO:0005525 GO:0005874 GO:0007017 GO:0007018 GO:0043234 GO:0051258	4e-41	96% (80/83)	ES781502
sb_gmnllfta_0006b05.t7	1		unclassified				ES781229
sb_gmnllfta_0006b14.t7	1		Cluster: Homolog of Homo sapiens "Plasma serine protease inhibitor precursor; n=4; Takifugu rubripes|Rep: Homolog of Homo sapiens "Plasma serine protease inhibitor precursor - Takifugu rubripes		8e-44	58% (88/151)	ES781564
sb_gmnllfta_0006b15.t7	1		unclassified				ES781594
sb_gmnllfta_0006b16.t7	1		LOC617104; similar to growth and transformation-dependent protein		1e-24	59% (52/88)	ES781663
sb_gmnllfta_0006b17.t7	1		unclassified				ES781614
sb_gmnllfta_0006b18.t7	1		unclassified				ES781995
sb_gmnllfta_0006b20.t7	1		Cluster: Carnitine palmitoyltransferase 1B; n=1; Sparus aurata|Rep: Carnitine palmitoyltransferase 1B - Sparus aurata (Gilthead sea bream)	GO:0008415 GO:0016740	3e-09	62% (32/51)	ES780887

sb_gmnllfta_0006b23.t7	1		unclassified				ES780808
sb_gmnllfta_0006c02.t7	1		Cluster: Alpha-tubulin; n=1; Halichondria sp. AR-2003|Rep: Alpha-tubulin - Halichondria sp. AR-2003	GO:0000166 GO:0003924 GO:0005198 GO:0005525 GO:0005737 GO:0005874 GO:0007017 GO:0007018 GO:0043234 GO:0051258	1e-12	88% (31/35)	ES781520
sb_gmnllfta_0006c07.t7	1		Cluster: LOC496289 protein; n=6; Xenopus|Rep: LOC496289 protein - Xenopus laevis (African clawed frog)	GO:0004222 GO:0006508	3e-12	53% (35/65)	ES781541
sb_gmnllfta_0006c12.t7	1		unclassified				ES781304
sb_gmnllfta_0006c18.t7	1		unclassified				ES780945
sb_gmnllfta_0006c19.t7	1		Cluster: Fatty acid-binding protein, heart; n=3; Euteleostomi|Rep: Fatty acid-binding protein, heart - Oncorhynchus mykiss (Rainbow trout) (Salmo gairdneri)	GO:0005488 GO:0006810 GO:0008289	9e-51	72% (96/133)	ES780885
sb_gmnllfta_0006c23.t7	1		Cluster: Ribosomal protein S30; n=3; Percomorpha|Rep: Ribosomal protein S30 - Solea senegalensis (Sole)	GO:0003735 GO:0005622 GO:0005840 GO:0006412 GO:0006464	1e-25	88% (56/63)	ES781863
sb_gmnllfta_0006d02.t7	1		unclassified				ES781457
sb_gmnllfta_0006d03.t7	1		unclassified				ES781408
sb_gmnllfta_0006d07.t7	1		Cluster: Novel protein similar to human RAB3 GTPase-activating protein; n=2; Danio rerio|Rep: Novel protein similar to human RAB3 GTPase-activating protein - Brachydanio rerio (Zebrafish) (Danio		2e-26	65% (58/88)	ES781640

			rerio)				
sb_gmnllfta_0006d09.t7	1		unclassified				ES781939
sb_gmnllfta_0006d19.t7	1		unclassified				ES780806
sb_gmnllfta_0006e06.t7	1		unclassified				ES781226
sb_gmnllfta_0006e07.t7	1		Cluster: PREDICTED: similar to Actin, cytoplasmic 2 (Gamma-actin); n=1; Rattus norvegicus|Rep: PREDICTED: similar to Actin, cytoplasmic 2 (Gamma-actin) - Rattus norvegicus		6e-73	92% (136/147)	ES781209
sb_gmnllfta_0006e10.t7	1		unclassified				ES781435
sb_gmnllfta_0006e11.t7	1		Cluster: Glycoprotein, synaptic 2; n=5; Clupeocephala|Rep: Glycoprotein, synaptic 2 - Brachydanio rerio (Zebrafish) (Danio rerio)		1e-55	94% (73/77)	ES781452
sb_gmnllfta_0006e12.t7	1		Cluster: Homolog of Paralichthys olivaceus "Complement component C3.; n=1; Takifugu rubripes|Rep: Homolog of Paralichthys olivaceus "Complement component C3. - Takifugu rubripes		5e-13	52% (39/74)	ES781523
sb_gmnllfta_0006e17.t7	1		Cluster: UPI0000569862 related cluster; n=1; Danio rerio|Rep: UPI0000569862 UniRef100 entry - Danio rerio		8e-07	54% (19/35)	ES781566
sb_gmnllfta_0006e19.t7	1		Tetraspanin 8 [Xenopus tropicalis]		3e-10	29% (38/131)	ES781861
sb_gmnllfta_0006e22.t7	1		Cluster: PREDICTED: similar to IGF2 mRNA-binding protein 1 isoform isoform 1; n=1; Pan troglodytes|Rep: PREDICTED: similar to IGF2 mRNA-binding protein 1 isoform isoform 1 - Pan troglodytes		7e-47	83% (89/106)	ES780940

sb_gmnllfta_0006e24.t7	1		unclassified				ES781029
sb_gmnllfta_0006f01.t7	1		unclassified				ES781717
sb_gmnllfta_0006f02.t7	1		unclassified				ES781831
sb_gmnllfta_0006f03.t7	1		unclassified				ES781776
sb_gmnllfta_0006f10.t7	1		Cluster: Fibrinogen beta chain; n=1; Pseudosciaena crocea|Rep: Fibrinogen beta chain - Pseudosciaena crocea (Croceine croaker)	GO:0005102 GO:0005577 GO:0007165 GO:0030168 GO:0030674 GO:0051258	4e-15	64% (27/42)	ES781041
sb_gmnllfta_0006f11.t7	1		unclassified				ES781059
sb_gmnllfta_0006f12.t7	1		Cluster: PREDICTED: similar to Dual specificity mitogen-activated protein kinase kinase 4 (MAP kinase kinase 4) (JNK activating kinase 1) (c-Jun N-terminal kinase kinase 1) (JNKK) (SAPK/ERK kinase 1) (SEK1); n=1; Canis familiaris|Rep: PREDICTED: similar to Dual specificity mitogen-activated protein kinase kinase 4 (MAP kinase kinase 4) (JNK activating kinase 1) (c-Jun N-terminal kinase kinase 1) (JNKK) (SAPK/ERK kinase 1) (SEK1) - Canis familiaris		1e-134	92% (235/254)	ES781009
sb_gmnllfta_0006f13.t7	1		unclassified				ES780961
sb_gmnllfta_0006f15.t7	1		LOC579126; similar to sea urchin Arp2 (SUArp2)		4e-52	71% (97/135)	ES780867
sb_gmnllfta_0006f19.t7	1		unclassified				ES781208

sb_gmnllfta_0006g01.t7	1		unclassified				ES780982
sb_gmnllfta_0006g02.t7	1		Cluster: Homolog of Brachydanio rerio "Sly1 (Suppressor of ypt1) (Novel vesicle-transport related protein).; n=1; Takifugu rubripes|Rep: Homolog of Brachydanio rerio "Sly1 (Suppressor of ypt1) (Novel vesicle-transport related protein). - Takifugu rubripes		3e-24	95% (59/62)	ES781091
sb_gmnllfta_0006g07.t7	1		Cluster: PREDICTED: similar to F-box/LRR-repeat protein 17 (F-box and leucine-rich repeat protein 17) (F-box only protein 13); n=1; Danio rerio|Rep: PREDICTED: similar to F-box/LRR-repeat protein 17 (F-box and leucine-rich repeat protein 17) (F-box only protein 13) - Danio rerio		1e-44	80% (93/115)	ES780805
sb_gmnllfta_0006g11.t7	1		Cluster: Homolog of Homo sapiens "cell division cycle and apoptosis regulator 1 (CCAR1), mRNA; n=1; Takifugu rubripes|Rep: Homolog of Homo sapiens "cell division cycle and apoptosis regulator 1 (CCAR1), mRNA - Takifugu rubripes		4e-16	40% (53/131)	ES781798
sb_gmnllfta_0006g13.t7	1		Cluster: Eukaryotic translation initiation factor 3 subunit 7; n=19; Euteleostomi|Rep: Eukaryotic translation initiation factor 3 subunit 7 - Homo sapiens (Human)		3e-39	94% (69/73)	ES781693
sb_gmnllfta_0006g16.t7	1		Cluster: Macrophage migration inhibitory factor; n=1; Danio rerio|Rep: Macrophage migration inhibitory factor - Brachydanio rerio (Zebrafish) (Danio rerio)		3e-33	72% (70/97)	ES781959
sb_gmnllfta_0006g17.t7	1		Cluster: Prothrombin; n=3; Oncorhynchus mykiss|Rep: Prothrombin - Oncorhynchus mykiss (Rainbow trout) (Salmo gairdneri)	GO:0003809<br>GO:0004252<br>GO:0005509<br>GO:0005576<br>GO:0006508<br>GO:0007596<br>GO:0016787	7e-25	80% (52/65)	ES781940
sb_gmnllfta_0006g21.t7	1		unclassified				ES781223
sb_gmnllfta_0006g24.t7	1		unclassified				ES781259

sb_gmnllfta_0006h05.t7	1		Cluster: Huntingtin-associated protein 1 (HAP-1) (Neuroan 1).; n=2; Xenopus tropicalis|Rep: Huntingtin-associated protein 1 (HAP-1) (Neuroan 1). - Xenopus tropicalis		3e-11	81% (35/43)	ES781773
sb_gmnllfta_0006h07.t7	1		Cluster: Alcohol dehydrogenase 1; n=1; Gadus callarias|Rep: Alcohol dehydrogenase 1 - Gadus callarias (Baltic cod)	GO:0004022 GO:0008270 GO:0016491 GO:0046872	4e-37	89% (61/68)	ES781722
sb_gmnllfta_0006h10.t7	1		unclassified				ES780905
sb_gmnllfta_0006h11.t7	1		unclassified				ES780896
sb_gmnllfta_0006h17.t7	1		unclassified				ES781062
sb_gmnllfta_0006h18.t7	1		Cluster: Chaperonin containing TCP1, subunit 5; n=5; Clupeocephala|Rep: Chaperonin containing TCP1, subunit 5 - Brachydanio rerio (Zebrafish) (Danio rerio)		3e-84	94% (159/169)	ES781362
sb_gmnllfta_0006h20.t7	1		unclassified				ES781516
sb_gmnllfta_0006h21.t7	1		Cluster: Inositol oxygenase; n=2; Danio rerio|Rep: Inositol oxygenase - Brachydanio rerio (Zebrafish) (Danio rerio)		2e-70	72% (121/166)	ES781507
sb_gmnllfta_0006h24.t7	1		Cluster: 3-hydroxyanthranilate 3,4-dioxygenase; n=1; Xenopus laevis|Rep: 3-hydroxyanthranilate 3,4-dioxygenase - Xenopus laevis (African clawed frog)	GO:0000334 GO:0005506 GO:0008152 GO:0016491 GO:0016702 GO:0046872	2e-40	83% (65/78)	ES781599
sb_gmnllfta_0006i02.t7	1		Cluster: Complement factor H precursor; n=1; Oncorhynchus mykiss|Rep: Complement factor H precursor - Oncorhynchus mykiss (Rainbow trout) (Salmo gairdneri)		9e-35	48% (64/133)	ES780890

sb_gmnllfta_0006i04.t7	1		Cluster: PREDICTED: similar to mSUG1 protein isoform 5; n=1; Pan troglodytes|Rep: PREDICTED: similar to mSUG1 protein isoform 5 - Pan troglodytes		1e-13	97% (37/38)	ES781095
sb_gmnllfta_0006i09.t7	1		unclassified				ES781306
sb_gmnllfta_0006i14.t7	1		Cluster: Tyrosine 3-monooxygenase/tryptophan 5-monooxygenase activation protein, beta polypeptide like; n=2; Danio rerio|Rep: Tyrosine 3-monooxygenase/tryptophan 5-monooxygenase activation protein, beta polypeptide like - Brachydanio rerio (Zebrafish) (Danio rerio)		1e-43	77% (58/75)	ES781755
sb_gmnllfta_0006i17.t7	1		Cluster: Chaperone protein GP96 (Tumor rejection antigen (Gp96) 1) (Heat shock protein 90kDa beta (Grp94), member 1); n=2; Clupeocephala|Rep: Chaperone protein GP96 (Tumor rejection antigen (Gp96) 1) (Heat shock protein 90kDa beta (Grp94), member 1) - Brachydanio rerio (Zebrafish) (Danio rerio)		2e-20	80% (46/57)	ES781799
sb_gmnllfta_0006i22.t7	1		Cluster: Actin-5C; n=194; Eukaryota|Rep: Actin-5C - Drosophila melanogaster (Fruit fly)		3e-23	95% (47/49)	ES781261
sb_gmnllfta_0006i23.t7	1		Cluster: Homolog of Homo sapiens "E74-like factor 1; n=1; Takifugu rubripes|Rep: Homolog of Homo sapiens "E74-like factor 1 - Takifugu rubripes		1e-31	46% (85/181)	ES781310
sb_gmnllfta_0006j09.t7	1		Cluster: PREDICTED: similar to CREB/ATF bZIP transcription factor isoform 1; n=1; Danio rerio|Rep: PREDICTED: similar to CREB/ATF bZIP transcription factor isoform 1 - Danio rerio		2e-39	43% (58/132)	ES781800
sb_gmnllfta_0006j14.t7	1		Endod1; endonuclease domain containing 1 [EC:3.1.30.-]; K01173 endonuclease		9e-05	45% (27/59)	ES781385
sb_gmnllfta_0006j17.t7	1		unclassified				ES781305
sb_gmnllfta_0006j18.t7	1		Cluster: Transcribed locus, weakly similar to XP_518227.1 desmoplakin [Pan troglodytes]; n=1; Takifugu rubripes|Rep: Transcribed locus, weakly similar to XP_518227.1 desmoplakin [Pan troglodytes] - Takifugu rubripes		1e-15	75% (22/29)	ES780966

sb_gmnllfta_0006k01.t7	1		unclassified				ES781212
sb_gmnllfta_0006k06.t7	1		unclassified				ES781363
sb_gmnllfta_0006k12.t7	1		unclassified				ES781617
sb_gmnllfta_0006k15.t7	1		TMED9; transmembrane emp24 protein transport domain containing 9	GO:0006810 GO:0016021	2e-06	75% (22/29)	ES781478
sb_gmnllfta_0006k16.t7	1		Cluster: Zgc:66393 protein; n=1; Danio rerio|Rep: Zgc:66393 protein - Brachydanio rerio (Zebrafish) (Danio rerio)		7e-18	59% (38/64)	ES781432
sb_gmnllfta_0006k19.t7	1		unclassified				ES781723
sb_gmnllfta_0006k23.t7	1		unclassified				ES781092
sb_gmnllfta_0006l02.t7	1		Cluster: Ceruloplasmin; n=1; Pseudopleuronectes americanus|Rep: Ceruloplasmin - Pseudopleuronectes americanus (Winter flounder) (Pleuronectesamericanus)		6e-05	59% (16/27)	ES781210
sb_gmnllfta_0006l07.t7	1		unclassified				ES781317
sb_gmnllfta_0006l13.t7	1		Cluster: 60S ribosomal protein L35; n=1; Ictalurus punctatus|Rep: 60S ribosomal protein L35 - Ictalurus punctatus (Channel catfish)	GO:0003735 GO:0005622 GO:0005840 GO:0006412 GO:0030529	5e-13	86% (32/37)	ES781588
sb_gmnllfta_0006l19.t7	1		unclassified				ES781835

sb_gmnllfta_0006l24.t7	1		Cluster: Ubiquitin-protein ligase; n=1; Ostreococcus tauri|Rep: Ubiquitin-protein ligase - Ostreococcus tauri	GO:0006464<br>GO:0006512<br>GO:0016874<br>GO:0019787	7e-08	36% (26/71)	ES780833
sb_gmnllfta_0006m09.t7	1		unclassified				ES781756
sb_gmnllfta_0006m11.t7	1		Cluster: Homolog of Homo sapiens "leukocyte cell-derived chemotaxin 2 precursor; n=1; Takifugu rubripes|Rep: Homolog of Homo sapiens "leukocyte cell-derived chemotaxin 2 precursor - Takifugu rubripes		5e-25	75% (52/69)	ES781178
sb_gmnllfta_0006m14.t7	1		Unassigned protein				ES781308
sb_gmnllfta_0006m16.t7	1		unclassified				ES781332
sb_gmnllfta_0006m17.t7	1		Cluster: Homolog of Homo sapiens "Thimet oligopeptidase; n=1; Takifugu rubripes|Rep: Homolog of Homo sapiens "Thimet oligopeptidase - Takifugu rubripes		2e-35	74% (43/58)	ES781388
sb_gmnllfta_0006n09.t7	1		Cluster: PREDICTED: similar to 40S ribosomal protein S17; n=1; Canis familiaris|Rep: PREDICTED: similar to 40S ribosomal protein S17 - Canis familiaris		4e-07	92% (25/27)	ES781386
sb_gmnllfta_0006n10.t7	1		unclassified				ES781914
sb_gmnllfta_0006n11.t7	1		Cluster: Homolog of Brachydanio rerio "Ceruloplasmin.; n=1; Takifugu rubripes|Rep: Homolog of Brachydanio rerio "Ceruloplasmin. - Takifugu rubripes		7e-84	72% (111/154)	ES781857
sb_gmnllfta_0006n16.t7	1		vkorc1l1; vitamin K epoxide reductase complex, subunit 1-like 1		3e-24	75% (51/68)	ES781691
sb_gmnllfta_0006n17.t7	1		unclassified				ES781757

sb_gmnllfta_0006n19.t7	1		LOC690570; similar to keratin associated protein 4-2		2e-05	52% (20/38)	ES781463
sb_gmnllfta_0006n22.t7	1		unclassified				ES781364
sb_gmnllfta_0006n24.t7	1		Cluster: PREDICTED: similar to ribosomal protein S23; n=2; Homo/Pan/Gorilla group|Rep: PREDICTED: similar to ribosomal protein S23 - Homo sapiens		1e-36	95% (76/80)	ES781230
sb_gmnllfta_0006o03.t7	1		unclassified				ES781880
sb_gmnllfta_0006o05.t7	1		unclassified				ES781696
sb_gmnllfta_0006o09.t7	1		Thymosin domain containing protein		8e-14	81% (30/37)	ES781527
sb_gmnllfta_0006o15.t7	1		RNA_pol_Rpb5_C domain containing protein		9e-05	66% (14/21)	ES781036
sb_gmnllfta_0006o21.t7	1		unclassified				ES781404
sb_gmnllfta_0006o22.t7	1		unclassified				ES781505
sb_gmnllfta_0006o24.t7	1		Cluster: PREDICTED: immunoglobulin mu binding protein 2; n=1; Danio rerio|Rep: PREDICTED: immunoglobulin mu binding protein 2 - Danio rerio		2e-22	46% (59/127)	ES781657
sb_gmnllfta_0006p07.t7	1		Cluster: IgG immunoreactive antigen; n=1; Strongyloides stercoralis|Rep: IgG immunoreactive antigen - Strongyloides stercoralis		1e-06	58% (23/39)	ES781043

sb_gmnllfta_0006p15.t7	1		unclassified				ES781827
sb_gmnllfta_0006p17.t7	1		ZNF148; zinc finger protein 148 (pHZ-52)		9e-18	53% (40/75)	ES781707
sb_gmnllfta_0007a05.t7	1		unclassified				FL634064
sb_gmnllfta_0007a07.t7	1		unclassified				FL634066
sb_gmnllfta_0007a10.t7	1		Cluster: CD63 antigen (Melanoma-associated antigen ME491) (Ocular melanoma- associated antigen) (OMA81H) (Granulophysin) (Tetraspanin-30) (Tspan- 30).; n=1; Takifugu rubripes|Rep: CD63 antigen (Melanoma-associated antigen ME491) (Ocular melanoma-associated antigen) (OMA81H) (Granulophysin) (Tetraspanin-30) (Tspan- 30). - Takifugu rubripes		1e-19	54% (48/88)	FL634067
sb_gmnllfta_0007a11.t7	1		unclassified				FL634068
sb_gmnllfta_0007a12.t7	1		unclassified				FL634069
sb_gmnllfta_0007a13.t7	1		unclassified				FL634070
sb_gmnllfta_0007a16.t7	1		Cluster: Probable UDP-sugar transporter protein SLC35A5; n=1; Danio rerio|Rep: Probable UDP-sugar transporter protein SLC35A5 - Danio rerio (Zebrafish) (Brachydanio rerio)		2e-21	55% (49/88)	FL634072
sb_gmnllfta_0007a22.t7	1		Cluster: Protein mago nashi homolog 2; n=25; Coelomata|Rep: Protein mago nashi homolog 2 - Homo sapiens (Human)		3e-14	100% (36/36)	FL634075
sb_gmnllfta_0007a24.t7	1		unclassified				FL634076

sb_gmnllfta_0007b01.t7	1		Cluster: Lipoic acid synthetase, mitochondrial precursor (Lip-syn) (Lipoate synthase).; n=1; Takifugu rubripes|Rep: Lipoic acid synthetase, mitochondrial precursor (Lip-syn) (Lipoate synthase). - Takifugu rubripes		6e-34	88% (72/81)	FL634077
sb_gmnllfta_0007b02.t7	1		unclassified				FL634078
sb_gmnllfta_0007b04.t7	1		unclassified				FL634079
sb_gmnllfta_0007b05.t7	1		unclassified				FL634080
sb_gmnllfta_0007b10.t7	1		unclassified				FL634084
sb_gmnllfta_0007b11.t7	1		unclassified				FL634085
sb_gmnllfta_0007b13.t7	1		Cluster: Histone acetyltransferase 1; n=2; Danio rerio|Rep: Histone acetyltransferase 1 - Danio rerio (Zebrafish) (Brachydanio rerio)		1e-05	88% (22/25)	FL634086
sb_gmnllfta_0007b14.t7	1		unclassified				FL634087
sb_gmnllfta_0007b15.t7	1		unclassified				FL634088
sb_gmnllfta_0007b19.t7	1		unclassified				FL634092
sb_gmnllfta_0007b21.t7	1		unclassified				FL634093

sb_gmnllfta_0007c01.t7	1		Cluster: Ribosomal protein LP0; n=11; Euteleostomi|Rep: Ribosomal protein LP0 - Solea senegalensis (Sole)		4e-35	98% (75/76)	FL634095
sb_gmnllfta_0007c12.t7	1		Cluster: Cytochrome P450 3A; n=1; Dicentrarchus labrax|Rep: Cytochrome P450 3A - Dicentrarchus labrax (European sea bass)	GO:0004497 GO:0005506 GO:0006118 GO:0016491 GO:0016712 GO:0020037	5e-54	77% (95/122)	FL634101
sb_gmnllfta_0007c14.t7	1		unclassified				FL634102
sb_gmnllfta_0007c16.t7	1		Cluster: 40S ribosomal protein S20; n=27; Coelomata|Rep: 40S ribosomal protein S20 - Homo sapiens (Human)		3e-17	100% (42/42)	FL634104
sb_gmnllfta_0007c17.t7	1		Cluster: Fatty acid elongase; n=1; Gadus morhua|Rep: Fatty acid elongase - Gadus morhua (Atlantic cod)		1e-19	100% (44/44)	FL634105
sb_gmnllfta_0007c18.t7	1		Cluster: Heat shock protein 90; n=13; Euteleostomi|Rep: Heat shock protein 90 - Scophthalmus maximus (Turbot)	GO:0005524 GO:0006457 GO:0006950	4e-12	100% (25/25)	FL634106
sb_gmnllfta_0007c19.t7	1		Cluster: PREDICTED: similar to ribosomal protein S12 isoform 3; n=1; Canis lupus familiaris|Rep: PREDICTED: similar to ribosomal protein S12 isoform 3 - Canis familiaris		1e-11	94% (35/37)	FL634107
sb_gmnllfta_0007c20.t7	1		Cluster: Cytochrome P450 2P1; n=1; Fundulus heteroclitus|Rep: Cytochrome P450 2P1 - Fundulus heteroclitus (Killifish) (Mummichog)	GO:0004497 GO:0005506 GO:0006118 GO:0016491 GO:0016712 GO:0020037	5e-31	78% (63/80)	FL634108
sb_gmnllfta_0007c21.t7	1		Cluster: Ribosomal protein S7; n=1; Myxine glutinosa|Rep: Ribosomal protein S7 - Myxine glutinosa (Atlantic hagfish)	GO:0003735 GO:0005622 GO:0005840	9e-06	80% (24/30)	FL634109
sb_gmnllfta_0007d04.t7	1		Cluster: Ribosomal protein L18; n=5; Euteleostei|Rep: Ribosomal protein L18 - Oncorhynchus masou formosanus		1e-05	92% (24/26)	FL634112

sb_gmnllfta_0007d06.t7	1		Cluster: 40S ribosomal protein S8; n=3; Amniota|Rep: 40S ribosomal protein S8 - Oxyuranus scutellatus	GO:0005622<br>GO:0005840	1e-20	97% (46/47)	FL634114
sb_gmnllfta_0007d07.t7	1		Cluster: Hemoglobin subunit beta-2; n=3; Gadidae|Rep: Hemoglobin subunit beta-2 - Gadus morhua (Atlantic cod)	GO:0005344<br>GO:0005506<br>GO:0005833<br>GO:0006810<br>GO:0015671<br>GO:0019825<br>GO:0020037	2e-06	100% (25/25)	FL634115
sb_gmnllfta_0007d14.t7	1		Cluster: eukaryotic translation initiation factor 3, subunit 1 alpha; n=1; Danio rerio|Rep: eukaryotic translation initiation factor 3, subunit 1 alpha - Danio rerio		5e-23	56% (54/95)	FL634119
sb_gmnllfta_0007d16.t7	1		unclassified				FL634120
sb_gmnllfta_0007d17.t7	1		unclassified				FL634121
sb_gmnllfta_0007d18.t7	1		unclassified				FL634122
sb_gmnllfta_0007d20.t7	1		Unassigned protein		8e-06	47% (20/42)	FL634123
sb_gmnllfta_0007e06.t7	1		unclassified				FL634128
sb_gmnllfta_0007e07.t7	1		Cluster: Ribonucleoside-diphosphate reductase subunit M2; n=2; Otophysi|Rep: Ribonucleoside-diphosphate reductase subunit M2 - Danio rerio (Zebrafish) (Brachydanio rerio)		1e-05	60% (25/41)	FL634129
sb_gmnllfta_0007e08.t7	1		unclassified				FL634130
sb_gmnllfta_0007e09.t7	1		Cluster: PREDICTED: similar to reverse transcriptase-like protein; n=1; Strongylocentrotus purpuratus|Rep: PREDICTED: similar to		5e-05	65%	FL634131

			reverse transcriptase-like protein - Strongylocentrotus purpuratus			(19/29)	
sb_gmnllfta_0007e10.t7	1		Cluster: Bcl-XL-like protein 1; n=2; Clupeocephala|Rep: Bcl-XL-like protein 1 - Danio rerio (Zebrafish) (Brachydanio rerio)		5e-10	68% (26/38)	FL634132
sb_gmnllfta_0007e12.t7	1		Cluster: Hemoglobin subunit alpha-2; n=3; Gadidae|Rep: Hemoglobin subunit alpha-2 - Gadus morhua (Atlantic cod)	GO:0005344 GO:0005506 GO:0005833 GO:0006810 GO:0015671 GO:0019825 GO:0020037	9e-07	96% (24/25)	FL634133
sb_gmnllfta_0007e13.t7	1		unclassified				FL634134
sb_gmnllfta_0007e19.t7	1		unclassified				FL634139
sb_gmnllfta_0007e22.t7	1		Cluster: MHC class Ia antigen; n=9; Gadus morhua|Rep: MHC class Ia antigen - Gadus morhua (Atlantic cod)	GO:0006955 GO:0016020 GO:0019882	3e-50	88% (93/105)	FL634141
sb_gmnllfta_0007e24.t7	1		hspa5; heat shock 70kDa protein 5 (glucose-regulated protein)		3e-18	95% (44/46)	FL634142
sb_gmnllfta_0007f04.t7	1		unclassified				FL634144
sb_gmnllfta_0007f05.t7	1		unclassified				FL634145
sb_gmnllfta_0007f06.t7	1		PDIA3, LOC478279; protein disulfide isomerase family A, member 3 [EC:5.3.4.1]; K08056 protein disulfide isomerase family A, member 3		6e-57	76% (101/132)	FL634146
sb_gmnllfta_0007f19.t7	1		unclassified				FL634154

sb_gmnllfta_0007f21.t7	1		unclassified				FL634156
sb_gmnllfta_0007f22.t7	1		unclassified				FL634157
sb_gmnllfta_0007g03.t7	1		Cluster: Heat shock cognate 71 kDa protein; n=64; Euteleostomi|Rep: Heat shock cognate 71 kDa protein - Homo sapiens (Human)		6e-73	94% (135/143)	FL634160
sb_gmnllfta_0007g04.t7	1		unclassified				FL634161
sb_gmnllfta_0007g05.t7	1		unclassified				FL634162
sb_gmnllfta_0007g07.t7	1		unclassified				FL634163
sb_gmnllfta_0007g14.t7	1		unclassified				FL634170
sb_gmnllfta_0007g15.t7	1		unclassified				FL634171
sb_gmnllfta_0007g16.t7	1		unclassified				FL634172
sb_gmnllfta_0007g17.t7	1		Cluster: Inter-alpha trypsin inhibitor; n=1; Fundulus heteroclitus|Rep: Inter-alpha trypsin inhibitor - Fundulus heteroclitus (Killifish) (Mummichog)		3e-23	81% (52/64)	FL634173
sb_gmnllfta_0007g18.t7	1		Cluster: Bifunctional coenzyme A synthase (CoA synthase) (NBP) (POV-2) [Includes: Phosphopantetheine adenylyltransferase (EC 2.7.7.3) (Pantetheine-phosphate adenylyltransferase) (PPAT) (Dephospho-CoA pyrophosphorylase); Dephospho-CoA kinase (EC 2.7.1.24) (DPCK) (D; n=1; Takifugu rubripes|Rep: Bifunctional coenzyme A synthase (CoA synthase) (NBP) (POV-2) [Includes: Phosphopantetheine adenylyltransferase (EC 2.7.7.3) (Pantetheine-phosphate adenylyltransferase) (PPAT) (Dephospho-CoA		3e-21	81% (52/64)	FL634174

			pyrophosphorylase); Dephospho-CoA kinase (EC 2.7.1.24) (DPCK) (D - Takifugu rubripes				
sb_gmnllfta_0007g19.t7	1		unclassified				FL634175
sb_gmnllfta_0007g22.t7	1		unclassified				FL634176
sb_gmnllfta_0007h02.t7	1		Cluster: Cytochrome P450 3A; n=1; Dicentrarchus labrax|Rep: Cytochrome P450 3A - Dicentrarchus labrax (European sea bass)	GO:0004497 GO:0005506 GO:0006118 GO:0016491 GO:0016712 GO:0020037	6e-33	80% (64/80)	FL634178
sb_gmnllfta_0007h03.t7	1		Cluster: Eukaryotic translation elongation factor 1 beta 2; n=2; Danio rerio|Rep: Eukaryotic translation elongation factor 1 beta 2 - Danio rerio (Zebrafish) (Brachydanio rerio)		5e-09	85% (17/20)	FL634179
sb_gmnllfta_0007h04.t7	1		unclassified				FL634180
sb_gmnllfta_0007h06.t7	1		unclassified				FL634182
sb_gmnllfta_0007h08.t7	1		Cluster: Minor spike protein; n=40; Enterobacteria phage phiX174 sensu lato|Rep: Minor spike protein - Bacteriophage phi-X174	GO:0005198 GO:0016032 GO:0019012	2e-65	87% (111/127)	FL634183
sb_gmnllfta_0007h09.t7	1		Cluster: Thioredoxin interacting protein; n=1; Danio rerio|Rep: Thioredoxin interacting protein - Danio rerio (Zebrafish) (Brachydanio rerio)		6e-08	73% (25/34)	FL634184
sb_gmnllfta_0007h14.t7	1		Cluster: Fibrinogen beta chain precursor [Contains: Fibrinopeptide B].; n=1; Takifugu rubripes|Rep: Fibrinogen beta chain precursor [Contains: Fibrinopeptide B]. - Takifugu rubripes		7e-10	89% (26/29)	FL634187
sb_gmnllfta_0007h20.t7	1		unclassified				FL634191

sb_gmnllfta_0007h21.t7	1		Cluster: Zgc:158276; n=3; Danio rerio|Rep: Zgc:158276 - Danio rerio (Zebrafish) (Brachydanio rerio)		7e-26	78% (55/70)	FL634192
sb_gmnllfta_0007h22.t7	1		unclassified				FL634193
sb_gmnllfta_0007i10.t7	1		unclassified				FL634198
sb_gmnllfta_0007i13.t7	1		Cluster: Hemoglobin subunit alpha-2; n=3; Gadidae|Rep: Hemoglobin subunit alpha-2 - Gadus morhua (Atlantic cod)	GO:0005344 GO:0005506 GO:0005833 GO:0006810 GO:0015671 GO:0019825 GO:0020037	2e-10	100% (30/30)	FL634200
sb_gmnllfta_0007i14.t7	1		Cluster: Cathepsin L; n=2; Percomorpha|Rep: Cathepsin L - Lates calcarifer (Barramundi)	GO:0004197 GO:0006508 GO:0008233 GO:0008234	2e-27	86% (56/65)	FL634201
sb_gmnllfta_0007i15.t7	1		unclassified				FL634202
sb_gmnllfta_0007i18.t7	1		unclassified				FL634204
sb_gmnllfta_0007i19.t7	1		Cluster: Hemoglobin subunit beta; n=3; Gadidae|Rep: Hemoglobin subunit beta - Gadus morhua (Atlantic cod)	GO:0005344 GO:0005506 GO:0005833 GO:0006810 GO:0015671 GO:0019825 GO:0020037	1e-12	100% (35/35)	FL634205
sb_gmnllfta_0007i21.t7	1		unclassified				FL634207
sb_gmnllfta_0007j03.t7	1		Unassigned protein				FL634209

sb_gmnllfta_0007j05.t7	1		unclassified				FL634211
sb_gmnllfta_0007j07.t7	1		Cluster: CC chemokine; n=3; Paralichthys olivaceus|Rep: CC chemokine - Paralichthys olivaceus (Japanese flounder)	GO:0005125 GO:0005576 GO:0005615 GO:0006955	1e-13	47% (33/70)	FL634213
sb_gmnllfta_0007j10.t7	1		Cluster: Splicing factor 3B subunit 1; n=27; Euteleostomi|Rep: Splicing factor 3B subunit 1 - Homo sapiens (Human)		6e-08	100% (26/26)	FL634215
sb_gmnllfta_0007j14.t7	1		unclassified				FL634218
sb_gmnllfta_0007j16.t7	1		Cluster: Hydroxyacylglutathione hydrolase (EC 3.1.2.6) (Glyoxalase II) (GLX II).; n=1; Takifugu rubripes|Rep: Hydroxyacylglutathione hydrolase (EC 3.1.2.6) (Glyoxalase II) (GLX II). - Takifugu rubripes		2e-25	81% (50/61)	FL634219
sb_gmnllfta_0007j18.t7	1		unclassified				FL634221
sb_gmnllfta_0007j19.t7	1		Cluster: 1-aminocyclopropane-1-carboxylate synthase-like protein; n=2; Takifugu rubripes|Rep: 1-aminocyclopropane-1-carboxylate synthase-like protein - Fugu rubripes (Japanese pufferfish) (Takifugu rubripes)	GO:0003824 GO:0009058 GO:0016769 GO:0016847	2e-11	68% (33/48)	FL634222
sb_gmnllfta_0007j21.t7	1		unclassified				FL634224
sb_gmnllfta_0007j22.t7	1		Cluster: Ribosomal protein L10a; n=2; Euteleostei|Rep: Ribosomal protein L10a - Oncorhynchus masou formosanus		1e-27	96% (62/64)	FL634225
sb_gmnllfta_0007k04.t7	1		unclassified				FL634227
sb_gmnllfta_0007k05.t7	1		unclassified				FL634228

sb_gmnllfta_0007k06.t7	1		unclassified				FL634229
sb_gmnllfta_0007k09.t7	1		Cluster: PREDICTED: similar to ribosomal protein S5; n=1; Ornithorhynchus anatinus|Rep: PREDICTED: similar to ribosomal protein S5 - Ornithorhynchus anatinus		3e-16	97% (42/43)	FL634231
sb_gmnllfta_0007k10.t7	1		Cluster: PREDICTED: similar to ribosomal protein S5; n=1; Ornithorhynchus anatinus|Rep: PREDICTED: similar to ribosomal protein S5 - Ornithorhynchus anatinus		1e-16	86% (45/52)	FL634232
sb_gmnllfta_0007k13.t7	1		unclassified				FL634234
sb_gmnllfta_0007k17.t7	1		unclassified				FL634237
sb_gmnllfta_0007k19.t7	1		unclassified				FL634239
sb_gmnllfta_0007k20.t7	1		Cluster: Non-specific cytotoxic cell receptor protein-1; n=1; Gadus morhua|Rep: Non-specific cytotoxic cell receptor protein-1 - Gadus morhua (Atlantic cod)	GO:0004872 GO:0005515	6e-08	70% (28/40)	FL634240
sb_gmnllfta_0007l04.t7	1		unclassified				FL634243
sb_gmnllfta_0007l09.t7	1		unclassified				FL634247
sb_gmnllfta_0007l11.t7	1		unclassified				FL634248
sb_gmnllfta_0007l13.t7	1		unclassified				FL634250

sb_gmnllfta_0007l15.t7	1		Cluster: Eukaryotic translation initiation factor 3 subunit M; n=3; Clupeocephala|Rep: Eukaryotic translation initiation factor 3 subunit M - Danio rerio (Zebrafish) (Brachydanio rerio)		9e-45	96% (95/98)	FL634252
sb_gmnllfta_0007l18.t7	1		Unassigned protein				FL634254
sb_gmnllfta_0007l19.t7	1		Cluster: Heat shock protein HSP 90-alpha; n=8; Clupeocephala|Rep: Heat shock protein HSP 90-alpha - Danio rerio (Zebrafish) (Brachydanio rerio)		6e-29	95% (63/66)	FL634255
sb_gmnllfta_0007l20.t7	1		unclassified				FL634256
sb_gmnllfta_0007l22.t7	1		Cluster: Si:dkeyp-117h8.5; n=2; Danio rerio|Rep: Si:dkeyp-117h8.5 - Danio rerio (Zebrafish) (Brachydanio rerio)		1e-82	94% (150/158)	FL634257
sb_gmnllfta_0007m02.t7	1		unclassified				FL634258
sb_gmnllfta_0007m07.t7	1		Cluster: Hemoglobin subunit alpha-1; n=3; Gadidae|Rep: Hemoglobin subunit alpha-1 - Gadus morhua (Atlantic cod)	GO:0005344 GO:0005506 GO:0005833 GO:0006810 GO:0015671 GO:0019825 GO:0020037	1e-10	100% (33/33)	FL634260
sb_gmnllfta_0007m09.t7	1		LOC697495; similar to hyaluronan binding protein 2 [EC:3.4.21.-]; K08648 hyaluronan binding protein 2		1e-06	52% (24/46)	FL634262
sb_gmnllfta_0007m10.t7	1		Cluster: Elongation factor 1-alpha; n=1; Anisakis simplex|Rep: Elongation factor 1-alpha - Anisakis simplex (Herring worm)	GO:0000166 GO:0003746 GO:0003924 GO:0005525 GO:0005737 GO:0006412	9e-20	97% (46/47)	FL634263
sb_gmnllfta_0007m11.t7	1		Cluster: Hydroxyacylglutathione hydrolase (EC 3.1.2.6) (Glyoxalase II) (GLX II).; n=1; Takifugu rubripes|Rep: Hydroxyacylglutathione hydrolase (EC 3.1.2.6) (Glyoxalase II) (GLX II). - Takifugu rubripes		2e-25	81% (50/61)	FL634264

sb_gmnllfta_0007m14.t7	1		unclassified				FL634267
sb_gmnllfta_0007m16.t7	1		unclassified				FL634269
sb_gmnllfta_0007m18.t7	1		unclassified				FL634271
sb_gmnllfta_0007m19.t7	1		Cluster: Zgc:112358; n=1; Danio rerio|Rep: Zgc:112358 - Danio rerio (Zebrafish) (Brachydanio rerio)		4e-05	86% (25/29)	FL634272
sb_gmnllfta_0007m23.t7	1		unclassified				FL634274
sb_gmnllfta_0007n02.t7	1		unclassified				FL634275
sb_gmnllfta_0007n03.t7	1		unclassified				FL634276
sb_gmnllfta_0007n04.t7	1		unclassified				FL634277
sb_gmnllfta_0007n05.t7	1		unclassified				FL634278
sb_gmnllfta_0007n09.t7	1		unclassified				FL634279
sb_gmnllfta_0007n11.t7	1		unclassified				FL634280
sb_gmnllfta_0007n12.t7	1		unclassified				FL634281

sb_gmnllfta_0007n14.t7	1		Cluster: Hemoglobin subunit alpha-1; n=3; Gadidae|Rep: Hemoglobin subunit alpha-1 - Gadus morhua (Atlantic cod)	GO:0005344 GO:0005506 GO:0005833 GO:0006810 GO:0015671 GO:0019825 GO:0020037	5e-07	93% (27/29)	FL634282
sb_gmnllfta_0007n17.t7	1		unclassified				FL634285
sb_gmnllfta_0007o01.t7	1		Cluster: Proteasome (Prosome, macropain) 26S subunit, non-ATPase, 7; n=15; Euteleostomi|Rep: Proteasome (Prosome, macropain) 26S subunit, non-ATPase, 7 - Danio rerio (Zebrafish) (Brachydanio rerio)		1e-61	85% (122/142)	FL634290
sb_gmnllfta_0007o10.t7	1		Cluster: PREDICTED: similar to Tubulin beta-6 chain (Beta-tubulin class-VI); n=2; Danio rerio|Rep: PREDICTED: similar to Tubulin beta-6 chain (Beta-tubulin class-VI) - Danio rerio		4e-09	77% (28/36)	FL634295
sb_gmnllfta_0007o12.t7	1		unclassified				FL634297
sb_gmnllfta_0007o13.t7	1		unclassified				FL634298
sb_gmnllfta_0007o16.t7	1		Cluster: 60S ribosomal protein L7a; n=39; Euteleostomi|Rep: 60S ribosomal protein L7a - Fugu rubripes (Japanese pufferfish) (Takifugu rubripes)	GO:0003735 GO:0005622 GO:0005840 GO:0006412 GO:0030529	3e-10	82% (34/41)	FL634301
sb_gmnllfta_0007o18.t7	1		unclassified				FL634303
sb_gmnllfta_0007o23.t7	1		Unassigned protein				FL634305
sb_gmnllfta_0007o24.t7	1		Cluster: Zgc:77429 protein; n=1; Danio rerio|Rep: Zgc:77429 protein - Danio rerio (Zebrafish) (Brachydanio rerio)		9e-16	66% (24/36)	FL634306

sb_gmnllfta_0007p20.t7	1		Cluster: Fatty acid elongase; n=1; Gadus morhua|Rep: Fatty acid elongase - Gadus morhua (Atlantic cod)		1e-19	100% (44/44)	FL634317

[1]Annotations presented in the supplemental table were generated with AutoFACT [18], while annotations presented in the manuscript are recent BLASTx hits that reflect a more updated state of the NCBI's nr protein database.

Supplemental Table S1C. Contigs in library gmnlmfta (forward heat-shock skeletal muscle SSH library) with supporting annotations[1], statistics, and contributing EST accession numbers

Sequence	Count	Sub-sequences	AutoFACT Description	GO terms	E-value	Identity	Accession Number
sb_gmnlmfta.13.C1	66	sb_gmnlmfta_0003c16.t7<br>sb_gmnlmfta_0004b11.t7<br>sb_gmnlmfta_0002p01.t7<br>sb_gmnlmfta_0004n14.t7<br>sb_gmnlmfta_0004l20.t7<br>sb_gmnlmfta_0002a09.t7<br>sb_gmnlmfta_0004b10.t7<br>sb_gmnlmfta_0002d06.t7<br>sb_gmnlmfta_0005g19.t7<br>sb_gmnlmfta_0001c10.t7<br>sb_gmnlmfta_0004j17.t7<br>sb_gmnlmfta_0003a17.t7<br>sb_gmnlmfta_0003i08.t7<br>sb_gmnlmfta_0005c14.t7<br>sb_gmnlmfta_0004e03.t7<br>sb_gmnlmfta_0005h08.t7<br>sb_gmnlmfta_0005d21.t7<br>sb_gmnlmfta_0003n09.t7<br>sb_gmnlmfta_0002j07.t7<br>sb_gmnlmfta_0004l13.t7<br>sb_gmnlmfta_0002g02.t7<br>sb_gmnlmfta_0003n07.t7<br>sb_gmnlmfta_0004h23.t7<br>sb_gmnlmfta_0003c12.t7<br>sb_gmnlmfta_0005i10.t7<br>sb_gmnlmfta_0002k16.t7<br>sb_gmnlmfta_0004f11.t7<br>sb_gmnlmfta_0003k03.t7<br>sb_gmnlmfta_0003o10.t7<br>sb_gmnlmfta_0004h09.t7<br>sb_gmnlmfta_0004k02.t7<br>sb_gmnlmfta_0003l20.t7<br>sb_gmnlmfta_0001a12.t7<br>sb_gmnlmfta_0005b11.t7<br>sb_gmnlmfta_0005c22.t7<br>sb_gmnlmfta_0001b08.t7<br>sb_gmnlmfta_0001d12.t7<br>sb_gmnlmfta_0002c11.t7<br>sb_gmnlmfta_0002e04.t7<br>sb_gmnlmfta_0002g07.t7<br>sb_gmnlmfta_0002j12.t7<br>sb_gmnlmfta_0002l15.t7<br>sb_gmnlmfta_0002n01.t7<br>sb_gmnlmfta_0003a02.t7<br>sb_gmnlmfta_0003a14.t7<br>sb_gmnlmfta_0003b17.t7<br>sb_gmnlmfta_0003g10.t7<br>sb_gmnlmfta_0003l21.t7<br>sb_gmnlmfta_0003m08.t7<br>sb_gmnlmfta_0004f10.t7<br>sb_gmnlmfta_0005d01.t7<br>sb_gmnlmfta_0005d17.t7<br>sb_gmnlmfta_0005g24.t7<br>sb_gmnlmfta_0005i08.t7<br>sb_gmnlmfta_0003h01.t7<br>sb_gmnlmfta_0003n21.t7<br>sb_gmnlmfta_0004a16.t7<br>sb_gmnlmfta_0002m20.t7<br>sb_gmnlmfta_0003p03.t7<br>sb_gmnlmfta_0005c16.t7<br>sb_gmnlmfta_0004i18.t7<br>sb_gmnlmfta_0005p16.t7<br>sb_gmnlmfta_0004m21.t7<br>sb_gmnlmfta_0002b09.t7<br>sb_gmnlmfta_0004g23.t7	Cluster: Parvalbumin; n=1; Theragra chalcogramma|Rep: Parvalbumin - Theragra chalcogramma (Alaska pollock)		5e-32	86% (74/86)	ES783759<br>ES783757<br>ES783333<br>ES783517<br>ES784177<br>ES784039<br>ES783748<br>ES784015<br>FL634710<br>ES783319<br>ES783850<br>ES783418<br>ES783534<br>FL634625<br>ES783817<br>FL634722<br>FL634653<br>ES783625<br>ES783875<br>ES783581<br>ES783477<br>ES783362<br>ES783689<br>ES783587<br>FL634746<br>ES783845<br>ES784187<br>ES783699<br>ES783434<br>ES783716<br>ES783960<br>ES783374<br>ES783553<br>FL634606<br>FL634632<br>ES783722<br>ES783277<br>ES783741<br>ES783558<br>ES783307<br>ES783630<br>ES783848<br>ES783372<br>ES784263<br>ES783460<br>ES783886<br>ES784273<br>ES783340<br>ES784230<br>ES784138<br>FL634635<br>FL634649<br>FL634715<br>FL634744<br>ES784074<br>ES783538<br>ES784246<br>ES784151<br>ES783407<br>FL634627<br>ES783732<br>FL634888<br>ES783482<br>ES783299<br>ES783963

		sb_gmnlmfta_0002o03.t7					ES784203
sb_gmnlmfta.20.C1	61	sb_gmnlmfta_0005c19.t7<br>sb_gmnlmfta_0005j20.t7<br>sb_gmnlmfta_0005h09.t7<br>sb_gmnlmfta_0005d15.t7<br>sb_gmnlmfta_0004j03.t7<br>sb_gmnlmfta_0005n17.t7<br>sb_gmnlmfta_0002l08.t7<br>sb_gmnlmfta_0005o21.t7<br>sb_gmnlmfta_0002g10.t7<br>sb_gmnlmfta_0003k13.t7<br>sb_gmnlmfta_0003c15.t7<br>sb_gmnlmfta_0004b03.t7<br>sb_gmnlmfta_0002i10.t7<br>sb_gmnlmfta_0002j10.t7<br>sb_gmnlmfta_0004m07.t7<br>sb_gmnlmfta_0005f15.t7<br>sb_gmnlmfta_0004l02.t7<br>sb_gmnlmfta_0005k11.t7<br>sb_gmnlmfta_0003e11.t7<br>sb_gmnlmfta_0002f11.t7<br>sb_gmnlmfta_0003c18.t7<br>sb_gmnlmfta_0005g16.t7<br>sb_gmnlmfta_0003m13.t7<br>sb_gmnlmfta_0003j02.t7<br>sb_gmnlmfta_0004d11.t7<br>sb_gmnlmfta_0002g03.t7<br>sb_gmnlmfta_0001g09.t7<br>sb_gmnlmfta_0003d09.t7<br>sb_gmnlmfta_0003k21.t7<br>sb_gmnlmfta_0004n02.t7<br>sb_gmnlmfta_0005h11.t7<br>sb_gmnlmfta_0005p11.t7<br>sb_gmnlmfta_0005b02.t7<br>sb_gmnlmfta_0003i17.t7<br>sb_gmnlmfta_0004n23.t7<br>sb_gmnlmfta_0003d16.t7<br>sb_gmnlmfta_0002j22.t7<br>sb_gmnlmfta_0004j24.t7<br>sb_gmnlmfta_0002d14.t7<br>sb_gmnlmfta_0002i14.t7<br>sb_gmnlmfta_0003c05.t7<br>sb_gmnlmfta_0001c02.t7<br>sb_gmnlmfta_0003d12.t7<br>sb_gmnlmfta_0002d20.t7<br>sb_gmnlmfta_0003o11.t7<br>sb_gmnlmfta_0002a16.t7<br>sb_gmnlmfta_0003i06.t7<br>sb_gmnlmfta_0005f02.t7<br>sb_gmnlmfta_0003d03.t7<br>sb_gmnlmfta_0003a12.t7<br>sb_gmnlmfta_0002o17.t7<br>sb_gmnlmfta_0003f04.t7<br>sb_gmnlmfta_0002l22.t7<br>sb_gmnlmfta_0003c24.t7<br>sb_gmnlmfta_0001c11.t7<br>sb_gmnlmfta_0002f03.t7<br>sb_gmnlmfta_0003b04.t7<br>sb_gmnlmfta_0004b13.t7<br>sb_gmnlmfta_0004n01.t7<br>sb_gmnlmfta_0005l01.t7<br>sb_gmnlmfta_0001g01.t7	Cluster: Fast skeletal muscle troponin T; n=1; Gadus morhua|Rep: Fast skeletal muscle troponin T - Gadus morhua (Atlantic cod)		7e-64	63% (138/217)	FL634629<br>FL634776<br>FL634723<br>FL634647<br>ES783555<br>FL634852<br>ES783429<br>FL634874<br>ES784192<br>ES783899<br>ES783714<br>ES783872<br>ES784274<br>ES783528<br>ES783701<br>FL634690<br>ES784012<br>FL634790<br>ES783995<br>ES783456<br>ES783499<br>FL634707<br>ES783719<br>ES783810<br>ES783843<br>ES783501<br>ES783488<br>ES784137<br>ES783263<br>ES784208<br>FL634725<br>FL634884<br>FL634601<br>ES784323<br>ES783919<br>ES783709<br>ES784180<br>ES783366<br>ES783623<br>ES784094<br>ES783918<br>ES784221<br>ES783629<br>ES784262<br>ES783412<br>ES783292<br>ES783270<br>FL634679<br>ES783954<br>ES783385<br>ES783417<br>ES784163<br>ES783451<br>ES784209<br>ES783276<br>ES784181<br>ES783734<br>ES783659<br>ES784295<br>FL634802<br>ES783621
sb_gmnlmfta.0.C2	50	sb_gmnlmfta_0002k24.t7<br>sb_gmnlmfta_0004n24.t7<br>sb_gmnlmfta_0005e14.t7<br>sb_gmnlmfta_0004k22.t7<br>sb_gmnlmfta_0005c12.t7<br>sb_gmnlmfta_0002p18.t7	actc1l; actin, alpha, cardiac muscle 1 like; K10354 actin alpha/gamma 2		1e-114	97% (207/213)	ES783352<br>ES784062<br>FL634669<br>ES784164<br>FL634623<br>ES783789

		sb_gmnlmfta_0002a15.t7<br>sb_gmnlmfta_0004f02.t7<br>sb_gmnlmfta_0002b06.t7<br>sb_gmnlmfta_0004c13.t7<br>sb_gmnlmfta_0005i04.t7<br>sb_gmnlmfta_0005m23.t7<br>sb_gmnlmfta_0004l11.t7<br>sb_gmnlmfta_0005f21.t7<br>sb_gmnlmfta_0004e01.t7<br>sb_gmnlmfta_0002h15.t7<br>sb_gmnlmfta_0002k14.t7<br>sb_gmnlmfta_0005p20.t7<br>sb_gmnlmfta_0002n11.t7<br>sb_gmnlmfta_0003c17.t7<br>sb_gmnlmfta_0003g12.t7<br>sb_gmnlmfta_0003m06.t7<br>sb_gmnlmfta_0004f23.t7<br>sb_gmnlmfta_0004h04.t7<br>sb_gmnlmfta_0004j02.t7<br>sb_gmnlmfta_0004n05.t7<br>sb_gmnlmfta_0005a04.t7<br>sb_gmnlmfta_0005e23.t7<br>sb_gmnlmfta_0005g20.t7<br>sb_gmnlmfta_0005j09.t7<br>sb_gmnlmfta_0005k16.t7<br>sb_gmnlmfta_0005n15.t7<br>sb_gmnlmfta_0005n18.t7<br>sb_gmnlmfta_0005p10.t7<br>sb_gmnlmfta_0004o11.t7<br>sb_gmnlmfta_0005j10.t7<br>sb_gmnlmfta_0004l03.t7<br>sb_gmnlmfta_0002h22.t7<br>sb_gmnlmfta_0004h24.t7<br>sb_gmnlmfta_0002f06.t7<br>sb_gmnlmfta_0003f20.t7<br>sb_gmnlmfta_0005c09.t7<br>sb_gmnlmfta_0004o19.t7<br>sb_gmnlmfta_0003o19.t7<br>sb_gmnlmfta_0004h19.t7<br>sb_gmnlmfta_0003m20.t7<br>sb_gmnlmfta_0005n12.t7<br>sb_gmnlmfta_0005n02.t7<br>sb_gmnlmfta_0002e06.t7<br>sb_gmnlmfta_0005i23.t7					ES783325<br>ES783494<br>ES783544<br>ES783798<br>FL634740<br>FL634837<br>ES783616<br>FL634694<br>ES783909<br>ES783425<br>ES783890<br>FL634891<br>ES784247<br>ES783747<br>ES784245<br>ES783957<br>ES783549<br>ES783505<br>ES783550<br>ES784064<br>FL634585<br>FL634677<br>FL634711<br>FL634765<br>FL634795<br>FL634851<br>FL634853<br>FL634883<br>ES784229<br>FL634766<br>ES784052<br>ES783832<br>ES783567<br>ES784218<br>ES783867<br>FL634620<br>ES783834<br>ES783559<br>ES783924<br>ES784318<br>FL634848<br>FL634839<br>ES783604<br>FL634757
sb_gmnlmfta.0.C1	44	sb_gmnlmfta_0005n23.t7<br>sb_gmnlmfta_0005b05.t7<br>sb_gmnlmfta_0001c03.t7<br>sb_gmnlmfta_0005a10.t7<br>sb_gmnlmfta_0001a09.t7<br>sb_gmnlmfta_0002a19.t7<br>sb_gmnlmfta_0003l10.t7<br>sb_gmnlmfta_0004j08.t7<br>sb_gmnlmfta_0004l04.t7<br>sb_gmnlmfta_0005a12.t7<br>sb_gmnlmfta_0005d10.t7<br>sb_gmnlmfta_0004m23.t7<br>sb_gmnlmfta_0005g11.t7<br>sb_gmnlmfta_0005g21.t7<br>sb_gmnlmfta_0003i11.t7<br>sb_gmnlmfta_0001g03.t7<br>sb_gmnlmfta_0003d15.t7<br>sb_gmnlmfta_0005h23.t7<br>sb_gmnlmfta_0005m19.t7<br>sb_gmnlmfta_0002i08.t7<br>sb_gmnlmfta_0003f12.t7<br>sb_gmnlmfta_0004f06.t7<br>sb_gmnlmfta_0005c21.t7<br>sb_gmnlmfta_0003d13.t7<br>sb_gmnlmfta_0003j13.t7<br>sb_gmnlmfta_0003k09.t7<br>sb_gmnlmfta_0003h11.t7<br>sb_gmnlmfta_0004o12.t7<br>sb_gmnlmfta_0003k02.t7<br>sb_gmnlmfta_0003e14.t7	Cluster: Parvalbumin beta; n=3; Gadidae|Rep: Parvalbumin beta - Merlangius merlangus (Whiting)		5e-39	93% (81/87)	FL634857<br>FL634602<br>ES784235<br>FL634589<br>ES784128<br>ES783560<br>ES783902<br>ES783458<br>ES783927<br>FL634591<br>FL634642<br>ES783445<br>FL634703<br>FL634712<br>ES784134<br>ES783574<br>ES783755<br>FL634737<br>FL634834<br>ES783710<br>ES783300<br>ES783312<br>FL634631<br>ES783610<br>ES783762<br>ES783375<br>ES783457<br>ES784275<br>ES783671<br>ES783889

		sb_gmnlmfta_0003i15.t7<br>sb_gmnlmfta_0003l01.t7<br>sb_gmnlmfta_0004b08.t7<br>sb_gmnlmfta_0004c17.t7<br>sb_gmnlmfta_0004i03.t7<br>sb_gmnlmfta_0004l12.t7<br>sb_gmnlmfta_0005d23.t7<br>sb_gmnlmfta_0005o18.t7<br>sb_gmnlmfta_0004m16.t7<br>sb_gmnlmfta_0004c10.t7<br>sb_gmnlmfta_0002e22.t7<br>sb_gmnlmfta_0004e17.t7<br>sb_gmnlmfta_0002g19.t7<br>sb_gmnlmfta_0002p05.t7					ES784232<br>ES783668<br>ES784248<br>ES784020<br>ES784304<br>ES783531<br>FL634655<br>FL634872<br>ES783877<br>ES783883<br>ES783342<br>ES783527<br>ES784048<br>ES783408
sb_gmnlmfta.5.C1	40	sb_gmnlmfta_0002n18.t7<br>sb_gmnlmfta_0003p14.t7<br>sb_gmnlmfta_0005k10.t7<br>sb_gmnlmfta_0003p13.t7<br>sb_gmnlmfta_0005j08.t7<br>sb_gmnlmfta_0002k19.t7<br>sb_gmnlmfta_0003k11.t7<br>sb_gmnlmfta_0004i01.t7<br>sb_gmnlmfta_0002k12.t7<br>sb_gmnlmfta_0003d07.t7<br>sb_gmnlmfta_0005p15.t7<br>sb_gmnlmfta_0005b20.t7<br>sb_gmnlmfta_0001c06.t7<br>sb_gmnlmfta_0002o24.t7<br>sb_gmnlmfta_0003a16.t7<br>sb_gmnlmfta_0003f01.t7<br>sb_gmnlmfta_0004a01.t7<br>sb_gmnlmfta_0004g24.t7<br>sb_gmnlmfta_0003p20.t7<br>sb_gmnlmfta_0005m12.t7<br>sb_gmnlmfta_0003g24.t7<br>sb_gmnlmfta_0001e10.t7<br>sb_gmnlmfta_0005i09.t7<br>sb_gmnlmfta_0001g06.t7<br>sb_gmnlmfta_0004l23.t7<br>sb_gmnlmfta_0005f24.t7<br>sb_gmnlmfta_0003i16.t7<br>sb_gmnlmfta_0002g06.t7<br>sb_gmnlmfta_0003b06.t7<br>sb_gmnlmfta_0003j21.t7<br>sb_gmnlmfta_0005j19.t7<br>sb_gmnlmfta_0004g19.t7<br>sb_gmnlmfta_0005p13.t7<br>sb_gmnlmfta_0003a05.t7<br>sb_gmnlmfta_0003d22.t7<br>sb_gmnlmfta_0002c19.t7<br>sb_gmnlmfta_0002j21.t7<br>sb_gmnlmfta_0004n06.t7<br>sb_gmnlmfta_0003a18.t7<br>sb_gmnlmfta_0002c21.t7	Cluster: Myosin light chain 2; n=2; Holacanthopterygii|Rep: Myosin light chain 2 - Theragra chalcogramma (Alaska pollock)		2e-70	94% (136/144)	ES783827<br>ES784284<br>FL634789<br>ES784144<br>FL634764<br>ES784078<br>ES783793<br>ES784214<br>ES784043<br>ES783873<br>FL634887<br>FL634612<br>ES784143<br>ES784013<br>ES783428<br>ES784258<br>ES783498<br>ES783811<br>ES783522<br>FL634827<br>ES783552<br>ES784302<br>FL634745<br>ES783753<br>ES784116<br>FL634697<br>ES784279<br>ES783271<br>ES783695<br>ES784205<br>FL634775<br>ES783597<br>FL634886<br>ES784118<br>ES784178<br>ES783363<br>ES784121<br>ES784150<br>ES783693<br>ES784286
sb_gmnlmfta.0.C6	38	sb_gmnlmfta_0003i14.t7<br>sb_gmnlmfta_0005j14.t7<br>sb_gmnlmfta_0004g11.t7<br>sb_gmnlmfta_0003m07.t7<br>sb_gmnlmfta_0001g12.t7<br>sb_gmnlmfta_0004c09.t7<br>sb_gmnlmfta_0004n13.t7<br>sb_gmnlmfta_0005a23.t7<br>sb_gmnlmfta_0005i13.t7<br>sb_gmnlmfta_0002c13.t7<br>sb_gmnlmfta_0002d08.t7<br>sb_gmnlmfta_0003f11.t7<br>sb_gmnlmfta_0003i23.t7<br>sb_gmnlmfta_0002m07.t7	Cluster: Actin, alpha 1, skeletal muscle; n=2; Eukaryota|Rep: Actin, alpha 1, skeletal muscle - Homo sapiens (Human)		4e-77	97% (143/146)	ES784237<br>FL634770<br>ES783511<br>ES783970<br>ES784000<br>ES783283<br>ES783379<br>FL634598<br>FL634748<br>ES783720<br>ES784277<br>ES783384<br>ES783564<br>ES783814

		sb_gmnlmfta_0002m16.t7<br>sb_gmnlmfta_0003g06.t7<br>sb_gmnlmfta_0004b18.t7<br>sb_gmnlmfta_0001d10.t7<br>sb_gmnlmfta_0002a02.t7<br>sb_gmnlmfta_0002a10.t7<br>sb_gmnlmfta_0002l03.t7<br>sb_gmnlmfta_0002o13.t7<br>sb_gmnlmfta_0003c03.t7<br>sb_gmnlmfta_0003h17.t7<br>sb_gmnlmfta_0003j11.t7<br>sb_gmnlmfta_0003j20.t7<br>sb_gmnlmfta_0004i19.t7<br>sb_gmnlmfta_0004p18.t7<br>sb_gmnlmfta_0005g23.t7<br>sb_gmnlmfta_0005j22.t7<br>sb_gmnlmfta_0005n14.t7<br>sb_gmnlmfta_0003f21.t7<br>sb_gmnlmfta_0005n10.t7<br>sb_gmnlmfta_0003b20.t7<br>sb_gmnlmfta_0002l19.t7<br>sb_gmnlmfta_0002o08.t7<br>sb_gmnlmfta_0003k17.t7<br>sb_gmnlmfta_0004d05.t7					ES783760<br>ES783441<br>ES783371<br>ES783357<br>ES784068<br>ES783436<br>ES783594<br>ES783321<br>ES784001<br>ES783378<br>ES783656<br>ES784260<br>ES783780<br>ES783706<br>FL634714<br>FL634778<br>FL634850<br>ES783921<br>FL634846<br>ES783470<br>ES784162<br>ES783897<br>ES783989<br>ES783566
sb_gmnlmfta.36.C1	23	sb_gmnlmfta_0005d24.t7<br>sb_gmnlmfta_0003k16.t7<br>sb_gmnlmfta_0004g02.t7<br>sb_gmnlmfta_0004k11.t7<br>sb_gmnlmfta_0003m09.t7<br>sb_gmnlmfta_0003j09.t7<br>sb_gmnlmfta_0002n14.t7<br>sb_gmnlmfta_0003h22.t7<br>sb_gmnlmfta_0002n07.t7<br>sb_gmnlmfta_0004l06.t7<br>sb_gmnlmfta_0002e02.t7<br>sb_gmnlmfta_0004l15.t7<br>sb_gmnlmfta_0002o15.t7<br>sb_gmnlmfta_0005a03.t7<br>sb_gmnlmfta_0005a22.t7<br>sb_gmnlmfta_0004f13.t7<br>sb_gmnlmfta_0005d12.t7<br>sb_gmnlmfta_0004d10.t7<br>sb_gmnlmfta_0004h16.t7<br>sb_gmnlmfta_0002j23.t7<br>sb_gmnlmfta_0004a24.t7<br>sb_gmnlmfta_0004m01.t7<br>sb_gmnlmfta_0004l05.t7	Cluster: Myosin light chain 1; n=1; Theragra chalcogramma|Rep: Myosin light chain 1 - Theragra chalcogramma (Alaska pollock)		3e-81	99% (149/150)	FL634656<br>ES783942<br>ES784170<br>ES783533<br>ES784239<br>ES784326<br>ES784136<br>ES783950<br>ES783483<br>ES783803<br>ES783672<br>ES783653<br>ES783514<br>FL634584<br>FL634597<br>ES784096<br>FL634644<br>ES783802<br>ES784132<br>ES784156<br>ES783725<br>ES783546<br>ES783862
sb_gmnlmfta.22.C1	20	sb_gmnlmfta_0005m06.t7<br>sb_gmnlmfta_0004f03.t7<br>sb_gmnlmfta_0002d19.t7<br>sb_gmnlmfta_0002e07.t7<br>sb_gmnlmfta_0003l04.t7<br>sb_gmnlmfta_0005l21.t7<br>sb_gmnlmfta_0003a10.t7<br>sb_gmnlmfta_0003a13.t7<br>sb_gmnlmfta_0004i05.t7<br>sb_gmnlmfta_0003l17.t7<br>sb_gmnlmfta_0003i02.t7<br>sb_gmnlmfta_0005g03.t7<br>sb_gmnlmfta_0003k10.t7<br>sb_gmnlmfta_0005i03.t7<br>sb_gmnlmfta_0003a15.t7<br>sb_gmnlmfta_0002h01.t7<br>sb_gmnlmfta_0005k08.t7<br>sb_gmnlmfta_0005k23.t7<br>sb_gmnlmfta_0001h11.t7<br>sb_gmnlmfta_0002a18.t7	Cluster: Myosin heavy chain; n=3; Gadidae|Rep: Myosin heavy chain - Theragra chalcogramma (Alaska pollock)	GO:0003774<br>GO:0005524	1e-127	92% (232/252)	FL634823<br>ES783484<br>ES783305<br>ES783637<br>ES783642<br>FL634816<br>ES783284<br>ES783320<br>ES784157<br>ES784038<br>ES783479<br>FL634698<br>ES783851<br>FL634739<br>ES783513<br>ES784257<br>FL634787<br>FL634800<br>ES783704<br>ES783547

sb_gmnlmfta.1.C1	18	sb_gmnlmfta_0001c09.t7<br>sb_gmnlmfta_0005c01.t7<br>sb_gmnlmfta_0005b21.t7<br>sb_gmnlmfta_0003e13.t7<br>sb_gmnlmfta_0001b02.t7<br>sb_gmnlmfta_0001a02.t7<br>sb_gmnlmfta_0001a08.t7<br>sb_gmnlmfta_0002c01.t7<br>sb_gmnlmfta_0003m14.t7<br>sb_gmnlmfta_0004a23.t7<br>sb_gmnlmfta_0004k06.t7<br>sb_gmnlmfta_0005m24.t7<br>sb_gmnlmfta_0005e06.t7<br>sb_gmnlmfta_0005e05.t7<br>sb_gmnlmfta_0005d02.t7<br>sb_gmnlmfta_0001d07.t7<br>sb_gmnlmfta_0004m17.t7<br>sb_gmnlmfta_0002p21.t7	ckm; creatine kinase, muscle [EC:2.7.3.2]; K00933 creatine kinase		2e-55	82%<br>(97/118)	ES783907<br>FL634616<br>FL634613<br>ES784024<br>ES783279<br>ES784032<br>ES784085<br>ES783807<br>ES783579<br>ES783600<br>ES783869<br>FL634838<br>FL634662<br>FL634661<br>FL634636<br>ES784097<br>ES783903<br>ES783652
sb_gmnlmfta.18.C1	16	sb_gmnlmfta_0002l05.t7<br>sb_gmnlmfta_0005b22.t7<br>sb_gmnlmfta_0004i08.t7<br>sb_gmnlmfta_0005m09.t7<br>sb_gmnlmfta_0003b08.t7<br>sb_gmnlmfta_0005j17.t7<br>sb_gmnlmfta_0005i07.t7<br>sb_gmnlmfta_0005i24.t7<br>sb_gmnlmfta_0003p18.t7<br>sb_gmnlmfta_0003i22.t7<br>sb_gmnlmfta_0003i01.t7<br>sb_gmnlmfta_0005l04.t7<br>sb_gmnlmfta_0005j12.t7<br>sb_gmnlmfta_0005g06.t7<br>sb_gmnlmfta_0005b10.t7<br>sb_gmnlmfta_0003a23.t7	Cluster: Myosin heavy chain; n=3; Gadidae|Rep: Myosin heavy chain - Theragra chalcogramma (Alaska pollock)	GO:0003774<br>GO:0005524	1e-163	81%<br>(314/387)	ES783777<br>FL634614<br>ES783792<br>FL634825<br>ES783430<br>FL634773<br>FL634743<br>FL634758<br>ES783984<br>ES783540<br>ES783393<br>FL634804<br>FL634768<br>FL634700<br>FL634605<br>ES783861
sb_gmnlmfta.7.C1	14	sb_gmnlmfta_0005i22.t7<br>sb_gmnlmfta_0003f22.t7<br>sb_gmnlmfta_0004l07.t7<br>sb_gmnlmfta_0002p17.t7<br>sb_gmnlmfta_0003c14.t7<br>sb_gmnlmfta_0002a13.t7<br>sb_gmnlmfta_0004g13.t7<br>sb_gmnlmfta_0005m01.t7<br>sb_gmnlmfta_0002j17.t7<br>sb_gmnlmfta_0003c02.t7<br>sb_gmnlmfta_0002h17.t7<br>sb_gmnlmfta_0002k06.t7<br>sb_gmnlmfta_0002n09.t7<br>sb_gmnlmfta_0002l17.t7	Cluster: Creatine kinase muscle isoform 2; n=8; Holacanthopterygii|Rep: Creatine kinase muscle isoform 2 - Chaenocephalus aceratus (White crocodile fish)	GO:0003824<br>GO:0016301<br>GO:0016740	8e-67	95%<br>(116/121)	FL634756<br>ES783833<br>ES783835<br>ES784127<br>ES783661<br>ES783518<br>ES783419<br>FL634819<br>ES783665<br>ES784061<br>ES783463<br>ES783772<br>ES783749<br>ES783887
sb_gmnlmfta.16.C1	13	sb_gmnlmfta_0005o04.t7<br>sb_gmnlmfta_0004b04.t7<br>sb_gmnlmfta_0003n11.t7<br>sb_gmnlmfta_0004m06.t7<br>sb_gmnlmfta_0002f09.t7<br>sb_gmnlmfta_0002j08.t7<br>sb_gmnlmfta_0004d14.t7<br>sb_gmnlmfta_0004j13.t7<br>sb_gmnlmfta_0002i24.t7	Cluster: Enolase; n=1; Tetraodon nigroviridis|Rep: Enolase - Tetraodon nigroviridis (Green puffer)	GO:0000015<br>GO:0000287<br>GO:0004634<br>GO:0005737<br>GO:0006096	2e-82	85%<br>(148/174)	FL634861<br>ES784003<br>ES784086<br>ES783670<br>ES783988<br>ES784189<br>ES784023<br>ES784036<br>ES783551

		sb_gmnlmfta_0002i01.t7<br>sb_gmnlmfta_0005n05.t7<br>sb_gmnlmfta_0005d04.t7<br>sb_gmnlmfta_0004d08.t7					ES783313<br>FL634842<br>FL634637<br>ES783383
sb_gmnlmfta.65.C1	10	sb_gmnlmfta_0003c10.t7<br>sb_gmnlmfta_0002k09.t7<br>sb_gmnlmfta_0005a18.t7<br>sb_gmnlmfta_0004k13.t7<br>sb_gmnlmfta_0004l16.t7<br>sb_gmnlmfta_0002e23.t7<br>sb_gmnlmfta_0004b05.t7<br>sb_gmnlmfta_0005a15.t7<br>sb_gmnlmfta_0003a01.t7<br>sb_gmnlmfta_0002h18.t7	LSU rRNA; Neoceratodus forsteri		7e-55	94% (130/138)	ES783608<br>ES783465<br>FL634594<br>ES783622<br>ES783744<br>ES783368<br>ES784059<br>FL634592<br>ES784320<br>ES783775
sb_gmnlmfta.23.C1	9	sb_gmnlmfta_0001d01.t7<br>sb_gmnlmfta_0005e03.t7<br>sb_gmnlmfta_0003g02.t7<br>sb_gmnlmfta_0005l19.t7<br>sb_gmnlmfta_0003m18.t7<br>sb_gmnlmfta_0005c15.t7<br>sb_gmnlmfta_0003e18.t7<br>sb_gmnlmfta_0002j19.t7<br>sb_gmnlmfta_0002b16.t7	Cluster: Parvalbumin beta; n=1; Theragra chalcogramma|Rep: Parvalbumin beta - Theragra chalcogramma (Alaska pollock)		5e-49	96% (96/99)	ES784222<br>FL634659<br>ES783365<br>FL634814<br>ES783346<br>FL634626<br>ES784115<br>ES783404<br>ES784028
sb_gmnlmfta.76.C1	8	sb_gmnlmfta_0002h02.t7<br>sb_gmnlmfta_0004l09.t7<br>sb_gmnlmfta_0003d02.t7<br>sb_gmnlmfta_0003h08.t7<br>sb_gmnlmfta_0004f20.t7<br>sb_gmnlmfta_0002m14.t7<br>sb_gmnlmfta_0002l23.t7<br>sb_gmnlmfta_0002e24.t7	Cluster: Translationally-controlled tumor protein; n=6; Otophysi|Rep: Translationally-controlled tumor protein - Danio rerio (Zebrafish) (Brachydanio rerio)		4e-60	65% (112/170)	ES784315<br>ES784108<br>ES783975<br>ES783944<br>ES783605<br>ES783660<br>ES783390<br>ES783493
sb_gmnlmfta.31.C1	7	sb_gmnlmfta_0005m05.t7<br>sb_gmnlmfta_0004n16.t7<br>sb_gmnlmfta_0002d10.t7<br>sb_gmnlmfta_0003k22.t7<br>sb_gmnlmfta_0002b05.t7<br>sb_gmnlmfta_0004d03.t7<br>sb_gmnlmfta_0002g04.t7	Cluster: Chaperonin containing TCP1, subunit 5; n=4; Clupeocephala|Rep: Chaperonin containing TCP1, subunit 5 - Danio rerio (Zebrafish) (Brachydanio rerio)		9e-79	93% (149/159)	FL634822<br>ES783410<br>ES783717<br>ES783343<br>ES783601<br>ES783683<br>ES783359
sb_gmnlmfta.45.C1	7	sb_gmnlmfta_0005o15.t7<br>sb_gmnlmfta_0002p22.t7<br>sb_gmnlmfta_0003d20.t7<br>sb_gmnlmfta_0004d17.t7<br>sb_gmnlmfta_0004p09.t7<br>sb_gmnlmfta_0004k18.t7<br>sb_gmnlmfta_0004m24.t7	Cluster: Heat shock protein 47; n=1; Oncorhynchus mykiss|Rep: Heat shock protein 47 - Oncorhynchus mykiss (Rainbow trout) (Salmo gairdneri)	GO:0004867	9e-77	75% (132/174)	FL634869<br>ES783752<br>ES784072<br>ES783938<br>ES783857<br>ES783394<br>ES783318

sb_gmnlmfta.8.C1	7	sb_gmnlmfta_0003h07.t7<br>sb_gmnlmfta_0004d01.t7<br>sb_gmnlmfta_0003j16.t7<br>sb_gmnlmfta_0003i03.t7<br>sb_gmnlmfta_0002a06.t7<br>sb_gmnlmfta_0002b22.t7<br>sb_gmnlmfta_0002d01.t7	unclassified				ES784249<br>ES783774<br>ES783536<br>ES783502<br>ES784292<br>ES783261<br>ES783864
sb_gmnlmfta.0.C4	6	sb_gmnlmfta_0005k13.t7<br>sb_gmnlmfta_0005k07.t7<br>sb_gmnlmfta_0003i21.t7<br>sb_gmnlmfta_0005l22.t7<br>sb_gmnlmfta_0004h05.t7<br>sb_gmnlmfta_0004a13.t7	Cluster: Adenine nucleotide translocase; n=2; Tetrapoda|Rep: Adenine nucleotide translocase - Xenopus tropicalis (Western clawed frog) (Silurana tropicalis)	GO:0005215<br>GO:0005488<br>GO:0005739<br>GO:0005743<br>GO:0006810<br>GO:0006839<br>GO:0016020	2e-36	81% (75/92)	FL634792<br>FL634786<br>ES783596<br>FL634817<br>ES783475<br>ES784135
sb_gmnlmfta.32.C1	6	sb_gmnlmfta_0004k04.t7<br>sb_gmnlmfta_0002g15.t7<br>sb_gmnlmfta_0001e08.t7<br>sb_gmnlmfta_0003m21.t7<br>sb_gmnlmfta_0003f02.t7<br>sb_gmnlmfta_0001c04.t7	Cluster: Isocitrate dehydrogenase 2 (NADP+), mitochondrial; n=3; Clupeocephala|Rep: Isocitrate dehydrogenase 2 (NADP+), mitochondrial - Danio rerio (Zebrafish) (Brachydanio rerio)		9e-79	95% (141/147)	ES783831<br>ES784233<br>ES783685<br>ES784287<br>ES784314<br>ES784098
sb_gmnlmfta.41.C1	6	sb_gmnlmfta_0002m09.t7<br>sb_gmnlmfta_0003i04.t7<br>sb_gmnlmfta_0004h02.t7<br>sb_gmnlmfta_0003f09.t7<br>sb_gmnlmfta_0005a21.t7<br>sb_gmnlmfta_0005f22.t7	Cluster: LIM domain binding 3; n=1; Danio rerio|Rep: LIM domain binding 3 - Danio rerio		1e-24	90% (54/60)	ES784092<br>ES783360<br>ES783351<br>ES783801<br>FL634596<br>FL634695
sb_gmnlmfta.43.C1	6	sb_gmnlmfta_0003f13.t7<br>sb_gmnlmfta_0003d24.t7<br>sb_gmnlmfta_0004d23.t7<br>sb_gmnlmfta_0004k12.t7<br>sb_gmnlmfta_0002k17.t7<br>sb_gmnlmfta_0002f02.t7	ckm; creatine kinase, muscle [EC:2.7.3.2]; K00933 creatine kinase		1e-91	90% (163/181)	ES783287<br>ES784300<br>ES783354<br>ES783619<br>ES783799<br>ES784153
sb_gmnlmfta.52.C1	6	sb_gmnlmfta_0005b09.t7<br>sb_gmnlmfta_0004g20.t7<br>sb_gmnlmfta_0005k04.t7<br>sb_gmnlmfta_0002d03.t7<br>sb_gmnlmfta_0004k08.t7<br>sb_gmnlmfta_0004n09.t7	unclassified				FL634604<br>ES784014<br>FL634784<br>ES783830<br>ES784133<br>ES783901
sb_gmnlmfta.6.C1	6	sb_gmnlmfta_0002g17.t7<br>sb_gmnlmfta_0005p21.t7<br>sb_gmnlmfta_0004m11.t7<br>sb_gmnlmfta_0005h16.t7<br>sb_gmnlmfta_0002l16.t7	Cluster: Actin, aortic smooth muscle; n=565; Eukaryota|Rep: Actin, aortic smooth muscle - Homo sapiens (Human)		2e-20	100% (46/46)	ES784322<br>FL634892<br>ES784027<br>FL634730<br>ES783895

		sb_gmnlmfta_0003b18.t7					ES784175
sb_gmnlmfta.11.C1	5	sb_gmnlmfta_0005g22.t7<br>sb_gmnlmfta_0005e22.t7<br>sb_gmnlmfta_0003e08.t7<br>sb_gmnlmfta_0005f11.t7<br>sb_gmnlmfta_0003a09.t7	Cluster: Homolog of Makaira nigricans "Sarcoplasmic/endoplasmic reticulum calcium ATPase 1 (EC 3.6.3.8) (Calcium pump 1) (SERCA1) (SR Ca(2+)-ATPase 1) (Calcium-transporting ATPase sarcoplasmic reticulum type, fast twitch skeletal muscle isoform) (Endoplasmic reticulum class; n=1; Takifugu rubripes|Rep: Homolog of Makaira nigricans "Sarcoplasmic/endoplasmic reticulum calcium ATPase 1 (EC 3.6.3.8) (Calcium pump 1) (SERCA1) (SR Ca(2+)-ATPase 1) (Calcium-transporting ATPase sarcoplasmic reticulum type, fast twitch skeletal muscle isoform) (Endoplasmic reticulum class - Takifugu rubripes		2e-90	92% (172/186)	FL634713<br>FL634676<br>ES783510<br>FL634686<br>ES783884
sb_gmnlmfta.20.C2	5	sb_gmnlmfta_0005m21.t7<br>sb_gmnlmfta_0002h10.t7<br>sb_gmnlmfta_0004e16.t7<br>sb_gmnlmfta_0002h23.t7<br>sb_gmnlmfta_0004h08.t7	Cluster: Fast skeletal muscle troponin T; n=1; Gadus morhua|Rep: Fast skeletal muscle troponin T - Gadus morhua (Atlantic cod)		8e-48	52% (90/172)	FL634836<br>ES783324<br>ES783585<br>ES783808<br>ES783657
sb_gmnlmfta.27.C1	5	sb_gmnlmfta_0004b02.t7<br>sb_gmnlmfta_0005h12.t7<br>sb_gmnlmfta_0003m17.t7<br>sb_gmnlmfta_0003l18.t7<br>sb_gmnlmfta_0002m10.t7	Cluster: 6-phosphofructokinase, muscle type (EC 2.7.1.11) (Phosphofructokinase 1) (Phosphohexokinase) (Phosphofructo-1-kinase isozyme A) (PFK-A) (Phosphofructokinase-M).; n=1; Takifugu rubripes|Rep: 6-phosphofructokinase, muscle type (EC 2.7.1.11) (Phosphofructokinase 1) (Phosphohexokinase) (Phosphofructo-1-kinase isozyme A) (PFK-A) (Phosphofructokinase-M). - Takifugu rubripes		1e-101	90% (177/195)	ES783916<br>FL634726<br>ES783627<br>ES784296<br>ES783609
sb_gmnlmfta.29.C1	5	sb_gmnlmfta_0003j23.t7<br>sb_gmnlmfta_0002o07.t7<br>sb_gmnlmfta_0001h10.t7<br>sb_gmnlmfta_0001b09.t7<br>sb_gmnlmfta_0004n21.t7	Cluster: Myosin heavy chain; n=5; Holacanthopterygii|Rep: Myosin heavy chain - Notothenia coriiceps (yellowbelly rockcod)	GO:0000166<br>GO:0003774<br>GO:0005524	1e-137	85% (246/289)	ES784313<br>ES784159<br>ES783684<br>ES783768<br>ES783815
sb_gmnlmfta.24.C1	4	sb_gmnlmfta_0004f05.t7<br>sb_gmnlmfta_0002h20.t7<br>sb_gmnlmfta_0005i18.t7<br>sb_gmnlmfta_0005j03.t7	Cluster: UTP--glucose-1-phosphate uridylyltransferase 2 (EC 2.7.7.9) (UDP- glucose pyrophosphorylase 2) (UDPGP 2) (UGPase 2).; n=1; Takifugu rubripes|Rep: UTP--glucose-1-phosphate uridylyltransferase 2 (EC 2.7.7.9) (UDP- glucose pyrophosphorylase 2) (UDPGP 2) (UGPase 2). - Takifugu rubripes		2e-36	93% (75/80)	ES783369<br>ES783868<br>FL634752<br>FL634761
sb_gmnlmfta.33.C1	4	sb_gmnlmfta_0002i05.t7<br>sb_gmnlmfta_0002i23.t7<br>sb_gmnlmfta_0003j06.t7<br>sb_gmnlmfta_0003b21.t7	si:ch211-81a5.1; si:ch211-81a5.1		5e-32	53% (73/137)	ES783492<br>ES783674<br>ES784016<br>ES783507
sb_gmnlmfta.51.C1	4	sb_gmnlmfta_0004m12.t7<br>sb_gmnlmfta_0004c12.t7<br>sb_gmnlmfta_0004b23.t7<br>sb_gmnlmfta_0004b06.t7	Cluster: Ferritin, middle subunit; n=5; Euteleostei|Rep: Ferritin, middle subunit - Salmo salar (Atlantic salmon)	GO:0004322<br>GO:0005488<br>GO:0005506<br>GO:0006826<br>GO:0006879	7e-64	76% (118/154)	ES783941<br>ES783847<br>ES784212<br>ES783982

				GO:0008199<br>GO:0016491<br>GO:0046872			
sb_gmnlmfta.66.C2	4	sb_gmnlmfta_0005o07.t7<br>sb_gmnlmfta_0002c03.t7<br>sb_gmnlmfta_0003l16.t7<br>sb_gmnlmfta_0004j19.t7	hsp90a; heat shock protein 90-alpha		8e-65	93% (123/132)	FL634864<br>ES783928<br>ES784029<br>ES784119
sb_gmnlmfta.70.C1	4	sb_gmnlmfta_0002p12.t7<br>sb_gmnlmfta_0003j03.t7<br>sb_gmnlmfta_0002e14.t7<br>sb_gmnlmfta_0002a14.t7	Unassigned protein				ES784281<br>ES783829<br>ES784034<br>ES783380
sb_gmnlmfta.84.C1	4	sb_gmnlmfta_0002h07.t7<br>sb_gmnlmfta_0002k13.t7<br>sb_gmnlmfta_0002b07.t7<br>sb_gmnlmfta_0002o20.t7	Cluster: Zgc:112176 protein; n=2; Danio rerio|Rep: Zgc:112176 protein - Danio rerio (Zebrafish) (Brachydanio rerio)		1e-75	72% (134/184)	ES784081<br>ES784025<br>ES783562<br>ES783804
sb_gmnlmfta.9.C1	4	sb_gmnlmfta_0002h14.t7<br>sb_gmnlmfta_0004a22.t7<br>sb_gmnlmfta_0004g21.t7<br>sb_gmnlmfta_0002e12.t7	Cluster: Parvalbumin beta; n=2; Gadus morhua|Rep: Parvalbumin beta - Gadus morhua (Atlantic cod)		2e-53	100% (107/107)	ES783420<br>ES783641<br>ES784050<br>ES783881
sb_gmnlmfta.99.C1	4	sb_gmnlmfta_0003o03.t7<br>sb_gmnlmfta_0004e18.t7<br>sb_gmnlmfta_0004f15.t7<br>sb_gmnlmfta_0004g18.t7	Cluster: PREDICTED: similar to mitochondrial outer membrane protein 19; n=1; Equus caballus|Rep: PREDICTED: similar to mitochondrial outer membrane protein 19 - Equus caballus		2e-06	57% (30/52)	ES784123<br>ES783314<br>ES784243<br>ES783644
sb_gmnlmfta.102.C1	3	sb_gmnlmfta_0002a21.t7<br>sb_gmnlmfta_0002g05.t7<br>sb_gmnlmfta_0002m06.t7	Cluster: Thioredoxin-like 1; n=2; Danio rerio|Rep: Thioredoxin-like 1 - Danio rerio (Zebrafish) (Brachydanio rerio)		1e-61	86% (111/129)	ES783948<br>ES783353<br>ES783825
sb_gmnlmfta.110.C1	3	sb_gmnlmfta_0004b01.t7<br>sb_gmnlmfta_0002h21.t7<br>sb_gmnlmfta_0004e09.t7	Cluster: Collagen alpha-1(I) chain precursor.; n=1; Takifugu rubripes|Rep: Collagen alpha-1(I) chain precursor. - Takifugu rubripes		3e-61	91% (113/123)	ES783828<br>ES783923<br>ES784272
sb_gmnlmfta.121.C1	3	sb_gmnlmfta_0002n05.t7<br>sb_gmnlmfta_0002c05.t7<br>sb_gmnlmfta_0002a05.t7	LSU rRNA; Xenopus borealis		1e-179	97% (345/354)	ES783444<br>ES784009<br>ES784211

sb_gmnlmfta.127.C1	3	sb_gmnlmfta_0001c07.t7<br>sb_gmnlmfta_0005p22.t7<br>sb_gmnlmfta_0003g08.t7	Cluster: Fast skeletal muscle troponin I; n=1; Gadus morhua|Rep: Fast skeletal muscle troponin I - Gadus morhua (Atlantic cod)		8e-25	98% (56/57)	ES784199<br>FL634893<br>ES783711
sb_gmnlmfta.129.C1	3	sb_gmnlmfta_0002o10.t7<br>sb_gmnlmfta_0003m10.t7<br>sb_gmnlmfta_0004f12.t7	Cluster: Proteasome (Prosome, macropain) 26S subunit, non-ATPase, 7; n=18; Euteleostomi|Rep: Proteasome (Prosome, macropain) 26S subunit, non-ATPase, 7 - Danio rerio (Zebrafish) (Brachydanio rerio)		4e-66	84% (129/153)	ES783282<br>ES783765<br>ES784099
sb_gmnlmfta.145.C1	3	sb_gmnlmfta_0004j05.t7<br>sb_gmnlmfta_0001h03.t7<br>sb_gmnlmfta_0005n06.t7	unclassified				ES783673<br>ES783787<br>FL634843
sb_gmnlmfta.149.C1	3	sb_gmnlmfta_0003f10.t7<br>sb_gmnlmfta_0002n24.t7<br>sb_gmnlmfta_0003l23.t7	Cluster: Proteasome 26S subunit, non-ATPase, 13; n=5; Danio rerio|Rep: Proteasome 26S subunit, non-ATPase, 13 - Danio rerio (Zebrafish) (Brachydanio rerio)		1e-83	78% (153/194)	ES783322<br>ES783598<br>ES783316
sb_gmnlmfta.15.C1	3	sb_gmnlmfta_0002d18.t7<br>sb_gmnlmfta_0002f01.t7<br>sb_gmnlmfta_0002f15.t7	Cluster: Zgc:86701; n=3; Danio rerio|Rep: Zgc:86701 - Danio rerio (Zebrafish) (Brachydanio rerio)		5e-19	60% (45/74)	ES783275<br>ES784075<br>ES783294
sb_gmnlmfta.17.C1	3	sb_gmnlmfta_0001e09.t7<br>sb_gmnlmfta_0003h16.t7<br>sb_gmnlmfta_0005j04.t7	Cluster: Creatine kinase muscle isoform 2; n=8; Holacanthopterygii|Rep: Creatine kinase muscle isoform 2 - Chaenocephalus aceratus (White crocodile fish)	GO:0003824<br>GO:0016301<br>GO:0016740	1e-18	77% (51/66)	ES783703<br>ES783329<br>FL634762
sb_gmnlmfta.19.C1	3	sb_gmnlmfta_0004m04.t7<br>sb_gmnlmfta_0001e11.t7<br>sb_gmnlmfta_0004j21.t7	Cluster: Fish sorting nexin from oocyte; n=1; Carassius auratus gibelio|Rep: Fish sorting nexin from oocyte - Carassius auratus gibelio	GO:0005515<br>GO:0007154	3e-49	64% (100/156)	ES783785<br>ES784283<br>ES783403
sb_gmnlmfta.25.C1	3	sb_gmnlmfta_0005j24.t7<br>sb_gmnlmfta_0005l14.t7<br>sb_gmnlmfta_0005g12.t7	Cluster: SAR1 gene homolog A; n=2; Danio rerio|Rep: SAR1 gene homolog A - Danio rerio (Zebrafish) (Brachydanio rerio)		3e-90	88% (161/181)	FL634780<br>FL634811<br>FL634704
sb_gmnlmfta.26.C1	3	sb_gmnlmfta_0002j13.t7<br>sb_gmnlmfta_0003p12.t7<br>sb_gmnlmfta_0002f13.t7	Cluster: Syntaxin binding protein 3; n=2; Danio rerio|Rep: Syntaxin binding protein 3 - Danio rerio (Zebrafish) (Brachydanio rerio)		5e-53	73% (111/152)	ES783611<br>ES784169<br>ES783432

sb_gmnlmfta.28.C1	3	sb_gmnlmfta_0002e13.t7<br>sb_gmnlmfta_0002g20.t7<br>sb_gmnlmfta_0002o11.t7	unclassified				ES783898<br>ES783646<br>ES783301
sb_gmnlmfta.34.C1	3	sb_gmnlmfta_0005i19.t7<br>sb_gmnlmfta_0004g10.t7<br>sb_gmnlmfta_0005l07.t7	unclassified				FL634753<br>ES783464<br>FL634807
sb_gmnlmfta.35.C1	3	sb_gmnlmfta_0004h10.t7<br>sb_gmnlmfta_0005d20.t7<br>sb_gmnlmfta_0004m15.t7	Cluster: Calsequestrin; n=1; Tetraodon nigroviridis|Rep: Calsequestrin - Tetraodon nigroviridis (Green puffer)		3e-64	93% (114/122)	ES784325<br>FL634652<br>ES783791
sb_gmnlmfta.37.C1	3	sb_gmnlmfta_0005h07.t7<br>sb_gmnlmfta_0004o08.t7<br>sb_gmnlmfta_0004e08.t7	Cluster: PREDICTED: similar to Heat shock protein HSP 90-beta (HSP 84) (Tumor-specific transplantation 84 kDa antigen) (TSTA); n=1; Rattus norvegicus|Rep: PREDICTED: similar to Heat shock protein HSP 90-beta (HSP 84) (Tumor-specific transplantation 84 kDa antigen) (TSTA) - Rattus norvegicus		3e-08	95% (19/20)	FL634721<br>ES783742<br>ES784330
sb_gmnlmfta.48.C1	3	sb_gmnlmfta_0003l19.t7<br>sb_gmnlmfta_0003c08.t7<br>sb_gmnlmfta_0003k06.t7	unclassified				ES784308<br>ES784103<br>ES783606
sb_gmnlmfta.71.C1	3	sb_gmnlmfta_0003j05.t7<br>sb_gmnlmfta_0003o20.t7<br>sb_gmnlmfta_0005o05.t7	Cluster: Enolase; n=6; Euteleostomi|Rep: Enolase - Danio rerio (Zebrafish) (Brachydanio rerio)		1e-77	93% (144/154)	ES783962<br>ES783981<br>FL634862
sb_gmnlmfta.75.C1	3	sb_gmnlmfta_0003d19.t7<br>sb_gmnlmfta_0003f06.t7<br>sb_gmnlmfta_0003p06.t7	Cluster: Slow troponin T 1; n=1; Sparus aurata|Rep: Slow troponin T 1 - Sparus aurata (Gilthead sea bream)		1e-54	61% (111/180)	ES783405<br>ES784112<br>ES783387
sb_gmnlmfta.87.C1	3	sb_gmnlmfta_0004o14.t7<br>sb_gmnlmfta_0004k20.t7<br>sb_gmnlmfta_0004p17.t7	unclassified				ES784197<br>ES784111<br>ES783406
sb_gmnlmfta.90.C1	3	sb_gmnlmfta_0004o02.t7<br>sb_gmnlmfta_0002m18.t7<br>sb_gmnlmfta_0003a07.t7	unclassified				ES783268<br>ES783500<br>ES784158

sb_gmnlmfta.94.C1	3	sb_gmnlmfta_0001a10.t7<br>sb_gmnlmfta_0005k15.t7<br>sb_gmnlmfta_0005p24.t7	unclassified				ES783635<br>FL634794<br>FL634895
sb_gmnlmfta.0.C3	2	sb_gmnlmfta_0002l11.t7<br>sb_gmnlmfta_0002n08.t7	Cluster: Adenosine monophosphate deaminase; n=2; Platichthys flesus|Rep: Adenosine monophosphate deaminase - Platichthys flesus (European flounder)	GO:0003876<br>GO:0009168	1e-64	88%<br>(109/123)	ES784045<br>ES783758
sb_gmnlmfta.0.C5	2	sb_gmnlmfta_0002p04.t7<br>sb_gmnlmfta_0001e01.t7	Cluster: Parvalbumin beta; n=3; Gadidae|Rep: Parvalbumin beta - Merlangius merlangus (Whiting)		6e-43	88%<br>(88/100)	ES783423<br>ES783280
sb_gmnlmfta.10.C1	2	sb_gmnlmfta_0005o23.t7<br>sb_gmnlmfta_0005h10.t7	Cluster: Zgc:66097; n=3; Danio rerio|Rep: Zgc:66097 - Danio rerio (Zebrafish) (Brachydanio rerio)		7e-54	52%<br>(113/215)	FL634876<br>FL634724
sb_gmnlmfta.100.C1	2	sb_gmnlmfta_0004k24.t7<br>sb_gmnlmfta_0005h06.t7	Cluster: PREDICTED: similar to initiation factor 4AII isoform 9; n=1; Pan troglodytes|Rep: PREDICTED: similar to initiation factor 4AII isoform 9 - Pan troglodytes		2e-57	90%<br>(113/125)	ES784316<br>FL634720
sb_gmnlmfta.101.C1	2	sb_gmnlmfta_0004a10.t7<br>sb_gmnlmfta_0004o10.t7	Cluster: Stathmin; n=1; Danio rerio|Rep: Stathmin - Danio rerio (Zebrafish) (Brachydanio rerio)		4e-50	90%<br>(100/110)	ES784093<br>ES784240
sb_gmnlmfta.103.C1	2	sb_gmnlmfta_0003c11.t7<br>sb_gmnlmfta_0002m15.t7	Cluster: Twinfilin, actin-binding protein, homolog 1b; n=1; Danio rerio|Rep: Twinfilin, actin-binding protein, homolog 1b - Danio rerio (Zebrafish) (Brachydanio rerio)		8e-19	79%<br>(42/53)	ES783632<br>ES783713
sb_gmnlmfta.104.C1	2	sb_gmnlmfta_0005a11.t7<br>sb_gmnlmfta_0004c16.t7	Cluster: Putative rRNA methyltransferase 3 (EC 2.1.1.-) (rRNA (uridine-2'-O-)- methyltransferase 3).; n=1; Takifugu rubripes|Rep: Putative rRNA methyltransferase 3 (EC 2.1.1.-) (rRNA (uridine-2'-O-)- methyltransferase 3). - Takifugu rubripes		7e-40	58%<br>(81/139)	FL634590<br>ES784046
sb_gmnlmfta.105.C1	2	sb_gmnlmfta_0005i21.t7<br>sb_gmnlmfta_0003b15.t7	Cluster: UPF0466 protein C22orf32 homolog, mitochondrial precursor; n=1; Xenopus laevis|Rep: UPF0466 protein C22orf32 homolog, mitochondrial precursor - Xenopus laevis (African clawed frog)	GO:0005739<br>GO:0016020	1e-20	76%<br>(45/59)	FL634755<br>ES783849
sb_gmnlmfta.106.C1	2	sb_gmnlmfta_0004f08.t7<br>sb_gmnlmfta_0001d11.t7	Cluster: Sarcoplasmic/endoplasmic reticulum calcium ATPase 1 (EC 3.6.3.8) (Calcium pump 1) (SERCA1) (SR Ca(2+)-ATPase 1) (Calcium-transporting ATPase sarcoplasmic reticulum type, fast twitch skeletal muscle isoform) (Endoplasmic reticulum class 1/2	GO:0000166<br>GO:0000287<br>GO:0003824<br>GO:0005388	4e-37	88%<br>(56/63)	ES783583<br>ES783337

			Ca(2+) ATPase); n=6; Holacanthopterygii|Rep: Sarcoplasmic/endoplasmic reticulum calcium ATPase 1 (EC 3.6.3.8) (Calcium pump 1) (SERCA1) (SR Ca(2+)-ATPase 1) (Calcium-transporting ATPase sarcoplasmic reticulum type, fast twitch skeletal muscle isoform) (Endoplasmic reticulum class 1/2 Ca(2+) ATPase) - Makaira nigricans (Blue marlin)	GO:0005509 GO:0005515 GO:0005524 GO:0005783 GO:0005789 GO:0006810 GO:0006811 GO:0006812 GO:0006816 GO:0008152 GO:0015662 GO:0016020 GO:0016021 GO:0016529 GO:0016787 GO:0016820 GO:0031448 GO:0033017			
sb_gmnlmfta.107.C1	2	sb_gmnlmfta_0005l17.t7 sb_gmnlmfta_0004b12.t7	Cluster: ATP synthase subunit alpha; n=1; Tetraodon nigroviridis|Rep: ATP synthase subunit alpha - Tetraodon nigroviridis (Green puffer)	GO:0000166 GO:0005524 GO:0006754 GO:0006810 GO:0006811 GO:0015078 GO:0015986 GO:0015992 GO:0016469 GO:0016787 GO:0016820 GO:0045261 GO:0046872 GO:0046933	4e-50	100% (99/99)	FL634813 ES783715
sb_gmnlmfta.108.C1	2	sb_gmnlmfta_0005p12.t7 sb_gmnlmfta_0004j23.t7	Cluster: Myosin heavy chain 4; n=39; Clupeocephala|Rep: Myosin heavy chain 4 - Danio rerio (Zebrafish) (Brachydanio rerio)		4e-96	89% (154/173)	FL634885 ES783491
sb_gmnlmfta.109.C1	2	sb_gmnlmfta_0003n05.t7 sb_gmnlmfta_0003h19.t7	Cluster: Annexin max3; n=1; Oryzias latipes|Rep: Annexin max3 - Oryzias latipes (Medaka fish) (Japanese ricefish)	GO:0005509 GO:0005544	5e-76	78% (144/184)	ES783265 ES783639
sb_gmnlmfta.111.C1	2	sb_gmnlmfta_0004n12.t7 sb_gmnlmfta_0002m19.t7	Cluster: Fast muscle-specific myosin heavy chain; n=6; Clupeocephala|Rep: Fast muscle-specific myosin heavy chain - Danio rerio (Zebrafish) (Brachydanio rerio)		8e-57	81% (117/144)	ES783327 ES783481
sb_gmnlmfta.112.C1	2	sb_gmnlmfta_0003c13.t7 sb_gmnlmfta_0004o13.t7	unclassified				ES783526 ES784328
sb_gmnlmfta.113.C1	2	sb_gmnlmfta_0003n16.t7 sb_gmnlmfta_0003d18.t7	novel NACHT domain containing protein [Danio rerio]		3e-54	61% (108/175)	ES784228 ES783437

sb_gmnlmfta.114.C1	2	sb_gmnlmfta_0003k19.t7<br>sb_gmnlmfta_0002c12.t7	unclassified				ES784251<br>ES783655
sb_gmnlmfta.115.C1	2	sb_gmnlmfta_0004o04.t7<br>sb_gmnlmfta_0005e21.t7	Cluster: Poly(A) binding protein, cytoplasmic 4; n=4; Clupeocephala|Rep: Poly(A) binding protein, cytoplasmic 4 - Danio rerio (Zebrafish) (Brachydanio rerio)		1e-121	93% (214/229)	ES783450<br>FL634675
sb_gmnlmfta.116.C1	2	sb_gmnlmfta_0005n13.t7<br>sb_gmnlmfta_0004g22.t7	Cluster: Proteasome subunit alpha type-5; n=23; Euteleostomi|Rep: Proteasome subunit alpha type-5 - Homo sapiens (Human)		1e-120	95% (219/230)	FL634849<br>ES783968
sb_gmnlmfta.117.C1	2	sb_gmnlmfta_0003m05.t7<br>sb_gmnlmfta_0003e01.t7	Cluster: Zgc:86701; n=3; Danio rerio|Rep: Zgc:86701 - Danio rerio (Zebrafish) (Brachydanio rerio)		2e-30	53% (69/130)	ES784010<br>ES783645
sb_gmnlmfta.118.C1	2	sb_gmnlmfta_0002g23.t7<br>sb_gmnlmfta_0003h18.t7	unclassified				ES783565<br>ES783603
sb_gmnlmfta.119.C1	2	sb_gmnlmfta_0003n04.t7<br>sb_gmnlmfta_0002c02.t7	unclassified				ES783309<br>ES783859
sb_gmnlmfta.12.C1	2	sb_gmnlmfta_0004c19.t7<br>sb_gmnlmfta_0002o18.t7	Cluster: ATP synthase D chain, mitochondrial (EC 3.6.3.14).; n=1; Takifugu rubripes|Rep: ATP synthase D chain, mitochondrial (EC 3.6.3.14). - Takifugu rubripes		4e-53	72% (97/134)	ES784285<br>ES783692
sb_gmnlmfta.120.C1	2	sb_gmnlmfta_0003o12.t7<br>sb_gmnlmfta_0003p23.t7	unclassified				ES783455<br>ES783620
sb_gmnlmfta.122.C1	2	sb_gmnlmfta_0004o17.t7<br>sb_gmnlmfta_0003d10.t7	Cluster: 60S ribosomal protein L35a; n=2; Otophysi|Rep: 60S ribosomal protein L35a - Ictalurus punctatus (Channel catfish)	GO:0000049<br>GO:0003723<br>GO:0003735<br>GO:0005622<br>GO:0005840<br>GO:0006412	2e-27	91% (56/61)	ES784107<br>ES783529
sb_gmnlmfta.123.C1	2	sb_gmnlmfta_0005c24.t7<br>sb_gmnlmfta_0005b13.t7	unclassified				FL634634<br>FL634608

sb_gmnlmfta.124.C1	2	sb_gmnlmfta_0002o12.t7<br>sb_gmnlmfta_0004o15.t7	Cluster: PREDICTED: similar to alpha 3-actin; n=1; Monodelphis domestica|Rep: PREDICTED: similar to alpha 3-actin - Monodelphis domestica		7e-16	100% (36/36)	ES783386<br>ES784130
sb_gmnlmfta.125.C1	2	sb_gmnlmfta_0005m10.t7<br>sb_gmnlmfta_0005j01.t7	unclassified				FL634826<br>FL634759
sb_gmnlmfta.126.C1	2	sb_gmnlmfta_0002g21.t7<br>sb_gmnlmfta_0002i16.t7	Cluster: Homolog of Danio rerio "MID1 interacting protein 1; n=1; Takifugu rubripes|Rep: Homolog of Danio rerio "MID1 interacting protein 1 - Takifugu rubripes		2e-14	67% (35/52)	ES783595<br>ES784186
sb_gmnlmfta.128.C1	2	sb_gmnlmfta_0003l03.t7<br>sb_gmnlmfta_0003n02.t7	Cluster: Heat shock 70 kDa protein 1; n=7; Euteleostei|Rep: Heat shock 70 kDa protein 1 - Oryzias latipes (Medaka fish) (Japanese ricefish)	GO:0000166<br>GO:0005524	6e-77	80% (148/183)	ES783784<br>ES783388
sb_gmnlmfta.130.C1	2	sb_gmnlmfta_0005j21.t7<br>sb_gmnlmfta_0003f17.t7	CD20 domain containing protein		1e-05	23% (29/124)	FL634777<br>ES783462
sb_gmnlmfta.131.C1	2	sb_gmnlmfta_0005m14.t7<br>sb_gmnlmfta_0003b14.t7	unclassified				FL634829<br>ES783797
sb_gmnlmfta.132.C1	2	sb_gmnlmfta_0002n06.t7<br>sb_gmnlmfta_0005d11.t7	Cluster: Aktip protein; n=2; Danio rerio|Rep: Aktip protein - Danio rerio (Zebrafish) (Brachydanio rerio)		3e-25	88% (52/59)	ES783496<br>FL634643
sb_gmnlmfta.133.C1	2	sb_gmnlmfta_0003n08.t7<br>sb_gmnlmfta_0004j20.t7	unclassified				ES783615<br>ES783439
sb_gmnlmfta.134.C1	2	sb_gmnlmfta_0002f19.t7<br>sb_gmnlmfta_0002p06.t7	Cluster: Proactivator polypeptide-like 1 precursor [Contains: Saposin A-like; Saposin B-Val-like; Saposin B-like; Saposin C-like; Saposin D-like].; n=1; Takifugu rubripes|Rep: Proactivator polypeptide-like 1 precursor [Contains: Saposin A-like; Saposin B-Val-like; Saposin B-like; Saposin C-like; Saposin D-like]. - Takifugu rubripes		9e-48	78% (86/109)	ES783638<br>ES783466

sb_gmnlmfta.135.C1	2	sb_gmnlmfta_0002g08.t7<br>sb_gmnlmfta_0002i15.t7	Cluster: Novel protein similar to alpha 2 type V collagen col5a2; n=3; Danio rerio|Rep: Novel protein similar to alpha 2 type V collagen col5a2 - Danio rerio (Zebrafish) (Brachydanio rerio)		4e-57	80%<br>(106/132)	ES783535<br>ES784100
sb_gmnlmfta.136.C1	2	sb_gmnlmfta_0003e03.t7<br>sb_gmnlmfta_0005o14.t7	unclassified				ES783539<br>FL634868
sb_gmnlmfta.137.C1	2	sb_gmnlmfta_0004c08.t7<br>sb_gmnlmfta_0005p05.t7	unclassified				ES783303<br>FL634878
sb_gmnlmfta.138.C1	2	sb_gmnlmfta_0003a21.t7<br>sb_gmnlmfta_0005o12.t7	Cluster: Zgc:101880; n=1; Danio rerio|Rep: Zgc:101880 - Danio rerio (Zebrafish) (Brachydanio rerio)		2e-29	76%<br>(62/81)	ES783836<br>FL634867
sb_gmnlmfta.139.C1	2	sb_gmnlmfta_0002a03.t7<br>sb_gmnlmfta_0002f12.t7	Cluster: Ferritin, heavy subunit; n=2; Salmonidae|Rep: Ferritin, heavy subunit - Salmo salar (Atlantic salmon)	GO:0004322<br>GO:0005488<br>GO:0005506<br>GO:0006826<br>GO:0006879<br>GO:0008199<br>GO:0016491<br>GO:0046872	2e-83	89%<br>(146/164)	ES784124<br>ES783415
sb_gmnlmfta.14.C1	2	sb_gmnlmfta_0003o16.t7<br>sb_gmnlmfta_0003g16.t7	Cluster: Aldehyde dehydrogenase 2, like; n=9; Danio rerio|Rep: Aldehyde dehydrogenase 2, like - Danio rerio (Zebrafish) (Brachydanio rerio)		1e-102	79%<br>(175/220)	ES783291<br>ES784185
sb_gmnlmfta.140.C1	2	sb_gmnlmfta_0003l08.t7<br>sb_gmnlmfta_0004h20.t7	Cluster: PREDICTED: similar to Glypican 6; n=3; Mammalia|Rep: PREDICTED: similar to Glypican 6 - Ornithorhynchus anatinus		1e-11	55%<br>(40/72)	ES783288<br>ES783771
sb_gmnlmfta.141.C1	2	sb_gmnlmfta_0003h04.t7<br>sb_gmnlmfta_0003p02.t7	unclassified				ES784306<br>ES783424
sb_gmnlmfta.142.C1	2	sb_gmnlmfta_0005p07.t7<br>sb_gmnlmfta_0005n21.t7	CDC27 multi-domain protein		6e-13	25%<br>(46/183)	FL634880<br>FL634856

sb_gmnlmfta.143.C1	2	sb_gmnlmfta_0002e08.t7<br>sb_gmnlmfta_0002l20.t7	Cluster: Zgc:86810; n=2; Clupeocephala|Rep: Zgc:86810 - Danio rerio (Zebrafish) (Brachydanio rerio)		4e-20	100% (51/51)	ES783330<br>ES783471
sb_gmnlmfta.144.C1	2	sb_gmnlmfta_0003g03.t7<br>sb_gmnlmfta_0004a07.t7	unclassified				ES783344<br>ES783339
sb_gmnlmfta.146.C1	2	sb_gmnlmfta_0003b10.t7<br>sb_gmnlmfta_0005a24.t7	Cluster: 6-phosphofructokinase, muscle type (EC 2.7.1.11) (Phosphofructokinase 1) (Phosphohexokinase) (Phosphofructo-1-kinase isozyme A) (PFK-A) (Phosphofructokinase-M).; n=1; Takifugu rubripes|Rep: 6-phosphofructokinase, muscle type (EC 2.7.1.11) (Phosphofructokinase 1) (Phosphohexokinase) (Phosphofructo-1-kinase isozyme A) (PFK-A) (Phosphofructokinase-M). - Takifugu rubripes		3e-28	92% (61/66)	ES784021<br>FL634599
sb_gmnlmfta.147.C1	2	sb_gmnlmfta_0002c07.t7<br>sb_gmnlmfta_0005p08.t7	Cluster: Phosphorylase; n=3; Clupeocephala|Rep: Phosphorylase - Danio rerio (Zebrafish) (Brachydanio rerio)		4e-71	86% (113/130)	ES783971<br>FL634881
sb_gmnlmfta.148.C1	2	sb_gmnlmfta_0003h03.t7<br>sb_gmnlmfta_0003h05.t7	unclassified				ES784182<br>ES784299
sb_gmnlmfta.150.C1	2	sb_gmnlmfta_0004a21.t7<br>sb_gmnlmfta_0004m19.t7	unclassified				ES783563<br>ES784184
sb_gmnlmfta.151.C1	2	sb_gmnlmfta_0005n09.t7<br>sb_gmnlmfta_0004e05.t7	unclassified				FL634845<br>ES783977
sb_gmnlmfta.2.C1	2	sb_gmnlmfta_0005c18.t7<br>sb_gmnlmfta_0001f03.t7	Cluster: Heat shock protein HSP 90-alpha; n=8; Clupeocephala|Rep: Heat shock protein HSP 90-alpha - Danio rerio (Zebrafish) (Brachydanio rerio)		5e-32	80% (72/90)	FL634628<br>ES783985
sb_gmnlmfta.21.C1	2	sb_gmnlmfta_0002d05.t7<br>sb_gmnlmfta_0002a01.t7	Cluster: Zgc:85615; n=5; Clupeocephala|Rep: Zgc:85615 - Danio rerio (Zebrafish) (Brachydanio rerio)		8e-33	91% (72/79)	ES783964<br>ES784147
sb_gmnlmfta.3.C1	2	sb_gmnlmfta_0002f22.t7	Cluster: Ribosomal protein L13a; n=7; Clupeocephala|Rep: Ribosomal protein L13a - Danio rerio (Zebrafish) (Brachydanio		3e-52	85%	ES783951<br>ES783813

		sb_gmnlmfta_0002n19.t7	rerio)			(104/122)	
sb_gmnlmfta.30.C1	2	sb_gmnlmfta_0003p19.t7<br>sb_gmnlmfta_0004m10.t7	Cluster: Zgc:123326; n=2; Danio rerio|Rep: Zgc:123326 - Danio rerio (Zebrafish) (Brachydanio rerio)		2e-19	77%<br>(46/59)	ES783931<br>ES784041
sb_gmnlmfta.38.C1	2	sb_gmnlmfta_0004i06.t7<br>sb_gmnlmfta_0005l05.t7	Cluster: Peptidyl-prolyl cis-trans isomerase; n=2; Tetraodontidae|Rep: Peptidyl-prolyl cis-trans isomerase - Tetraodon nigroviridis (Green puffer)	GO:0003755<br>GO:0006457	6e-26	85%<br>(53/62)	ES784071<br>FL634805
sb_gmnlmfta.39.C1	2	sb_gmnlmfta_0002g14.t7<br>sb_gmnlmfta_0002b14.t7	unclassified				ES784238<br>ES783992
sb_gmnlmfta.4.C1	2	sb_gmnlmfta_0003d01.t7<br>sb_gmnlmfta_0003b23.t7	unclassified				ES784057<br>ES783392
sb_gmnlmfta.40.C1	2	sb_gmnlmfta_0003f03.t7<br>sb_gmnlmfta_0002a08.t7	Cluster: choline/ethanolaminephosphotransferase; n=1; Takifugu rubripes|Rep: choline/ethanolaminephosphotransferase - Takifugu rubripes		2e-20	58%<br>(54/92)	ES784288<br>ES784026
sb_gmnlmfta.42.C1	2	sb_gmnlmfta_0004o09.t7<br>sb_gmnlmfta_0004c04.t7	unclassified				ES783766<br>ES783591
sb_gmnlmfta.44.C1	2	sb_gmnlmfta_0001h01.t7<br>sb_gmnlmfta_0005k03.t7	Cluster: ETF1 protein; n=6; Mammalia|Rep: ETF1 protein - Homo sapiens (Human)		7e-53	82%<br>(106/129)	ES783906<br>FL634783
sb_gmnlmfta.46.C1	2	sb_gmnlmfta_0003h15.t7<br>sb_gmnlmfta_0002d16.t7	Cluster: Sarcoplasmic/endoplasmic reticulum calcium ATPase 1 (EC 3.6.3.8) (Calcium pump 1) (SERCA1) (SR Ca(2+)-ATPase 1) (Calcium-transporting ATPase sarcoplasmic reticulum type, fast twitch skeletal muscle isoform) (Endoplasmic reticulum class 1/2 Ca(2+) ATPase); n=6; Holacanthopterygii|Rep: Sarcoplasmic/endoplasmic reticulum calcium ATPase 1 (EC 3.6.3.8) (Calcium pump 1) (SERCA1) (SR Ca(2+)-ATPase 1) (Calcium-transporting ATPase sarcoplasmic reticulum type, fast twitch skeletal muscle isoform) (Endoplasmic reticulum class 1/2 Ca(2+) ATPase) - Makaira nigricans (Blue marlin)	GO:0000166<br>GO:0000287<br>GO:0003824<br>GO:0005388<br>GO:0005509<br>GO:0005515<br>GO:0005524<br>GO:0005783<br>GO:0005789<br>GO:0006810<br>GO:0006811<br>GO:0006812<br>GO:0006816<br>GO:0008152<br>GO:0015662<br>GO:0016020<br>GO:0016021<br>GO:0016529<br>GO:0016787<br>GO:0016820	6e-55	72%<br>(70/96)	ES783293<br>ES783537

				GO:0031448<br>GO:0033017			
sb_gmnlmfta.47.C1	2	sb_gmnlmfta_0004e14.t7<br>sb_gmnlmfta_0004m14.t7	unclassified				ES783612<br>ES783854
sb_gmnlmfta.49.C1	2	sb_gmnlmfta_0005o20.t7<br>sb_gmnlmfta_0004g01.t7	Cluster: PREDICTED: similar to Mid-1-related chloride channel 1; n=1; Equus caballus|Rep: PREDICTED: similar to Mid-1-related chloride channel 1 - Equus caballus		5e-32	44%<br>(71/160)	FL634873<br>ES784114
sb_gmnlmfta.50.C1	2	sb_gmnlmfta_0005n19.t7<br>sb_gmnlmfta_0002l06.t7	unclassified				FL634854<br>ES783694
sb_gmnlmfta.53.C1	2	sb_gmnlmfta_0004e13.t7<br>sb_gmnlmfta_0004j07.t7	Cluster: Homolog of Homo sapiens "N2B-Titin Isoform.; n=1; Takifugu rubripes|Rep: Homolog of Homo sapiens "N2B-Titin Isoform. - Takifugu rubripes		3e-67	81%<br>(121/148)	ES783751<br>ES783782
sb_gmnlmfta.54.C1	2	sb_gmnlmfta_0002e16.t7<br>sb_gmnlmfta_0005g09.t7	Cluster: Proteasome subunit alpha type-1; n=3; Catarrhini|Rep: Proteasome subunit alpha type-1 - Homo sapiens (Human)		5e-45	73%<br>(91/124)	ES783943<br>FL634701
sb_gmnlmfta.55.C1	2	sb_gmnlmfta_0004e21.t7<br>sb_gmnlmfta_0005g17.t7	Cluster: Fructose-bisphosphate aldolase; n=9; Clupeocephala|Rep: Fructose-bisphosphate aldolase - Danio rerio (Zebrafish) (Brachydanio rerio)		2e-48	93%<br>(91/97)	ES784250<br>FL634708
sb_gmnlmfta.56.C1	2	sb_gmnlmfta_0003k01.t7<br>sb_gmnlmfta_0002m02.t7	unclassified				ES783731<br>ES784063
sb_gmnlmfta.57.C1	2	sb_gmnlmfta_0002g13.t7<br>sb_gmnlmfta_0002k20.t7	unclassified				ES784106<br>ES783397
sb_gmnlmfta.58.C1	2	sb_gmnlmfta_0005e12.t7<br>sb_gmnlmfta_0002i07.t7	Cluster: Mucolipin 1; n=1; Danio rerio|Rep: Mucolipin 1 - Danio rerio (Zebrafish) (Brachydanio rerio)		7e-52	64%<br>(101/157)	FL634667<br>ES783402
sb_gmnlmfta.59.C1	2	sb_gmnlmfta_0003e17.t7<br>sb_gmnlmfta_0002c23.t7	unclassified				ES783800<br>ES784265

sb_gmnlmfta.60.C1	2	sb_gmnlmfta_0004g16.t7<br>sb_gmnlmfta_0002e18.t7	Cluster: Ribosomal protein L7; n=4; Danio rerio|Rep: Ribosomal protein L7 - Danio rerio (Zebrafish) (Brachydanio rerio)		5e-53	90% (97/107)	ES783382<br>ES784215
sb_gmnlmfta.61.C1	2	sb_gmnlmfta_0004e12.t7<br>sb_gmnlmfta_0003h21.t7	Cluster: PREDICTED: similar to ribosomal protein L10a, partial; n=1; Ornithorhynchus anatinus|Rep: PREDICTED: similar to ribosomal protein L10a, partial - Ornithorhynchus anatinus		3e-56	76% (111/146)	ES783754<br>ES784008
sb_gmnlmfta.62.C1	2	sb_gmnlmfta_0005d05.t7<br>sb_gmnlmfta_0003n13.t7	unclassified				FL634638<br>ES784196
sb_gmnlmfta.63.C1	2	sb_gmnlmfta_0002i04.t7<br>sb_gmnlmfta_0001c12.t7	Cluster: Ribosomal protein L12; n=1; Pagrus major|Rep: Ribosomal protein L12 - Pagrus major (Red sea bream) (Chrysophrys major)	GO:0003735<br>GO:0005622<br>GO:0005840<br>GO:0006412	9e-28	94% (55/58)	ES783486<br>ES783336
sb_gmnlmfta.64.C1	2	sb_gmnlmfta_0001b10.t7<br>sb_gmnlmfta_0003h13.t7	Cluster: EIF4G-related protein NAT1A; n=3; Danio rerio|Rep: EIF4G-related protein NAT1A - Danio rerio (Zebrafish) (Brachydanio rerio)		8e-21	59% (59/100)	ES784268<br>ES783431
sb_gmnlmfta.66.C1	2	sb_gmnlmfta_0005k05.t7<br>sb_gmnlmfta_0003c01.t7	Cluster: Myosin heavy chain; n=3; Gadidae|Rep: Myosin heavy chain - Theragra chalcogramma (Alaska pollock)	GO:0003774<br>GO:0005524	6e-44	95% (85/89)	FL634785<br>ES783980
sb_gmnlmfta.67.C1	2	sb_gmnlmfta_0003e07.t7<br>sb_gmnlmfta_0005j13.t7	unclassified				ES783739<br>FL634769
sb_gmnlmfta.68.C1	2	sb_gmnlmfta_0003e16.t7<br>sb_gmnlmfta_0003m16.t7	Cluster: Homolog of Homo sapiens "Photoreceptor outer segment all-trans retinol dehydrogenase; n=1; Takifugu rubripes|Rep: Homolog of Homo sapiens "Photoreceptor outer segment all-trans retinol dehydrogenase - Takifugu rubripes		1e-41	59% (79/132)	ES783846<br>ES783614
sb_gmnlmfta.69.C1	2	sb_gmnlmfta_0002j14.t7<br>sb_gmnlmfta_0005a17.t7	Cluster: Zgc:101050; n=5; Danio rerio|Rep: Zgc:101050 - Danio rerio (Zebrafish) (Brachydanio rerio)		1e-41	46% (50/107)	ES783750<br>FL634593
sb_gmnlmfta.72.C1	2	sb_gmnlmfta_0004j11.t7	unclassified				ES783945<br>ES784305

		sb_gmnlmfta_0004e22.t7					
sb_gmnlmfta.73.C1	2	sb_gmnlmfta_0002d02.t7<br>sb_gmnlmfta_0002i17.t7	PSCA; prostate stem cell antigen		1e-07	36%<br>(36/99)	ES783812<br>ES784141
sb_gmnlmfta.74.C1	2	sb_gmnlmfta_0003e15.t7<br>sb_gmnlmfta_0003f16.t7	Cluster: Putative fast skeletal muscle troponin; n=1; Paralichthys olivaceus|Rep: Putative fast skeletal muscle troponin - Paralichthys olivaceus (Japanese flounder)		2e-18	64%<br>(43/67)	ES783892<br>ES783512
sb_gmnlmfta.77.C1	2	sb_gmnlmfta_0005f10.t7<br>sb_gmnlmfta_0005h14.t7	Unassigned protein		7e-05	48%<br>(23/47)	FL634685<br>FL634728
sb_gmnlmfta.78.C1	2	sb_gmnlmfta_0005g14.t7<br>sb_gmnlmfta_0003f14.t7	Cluster: Mitochondrial import inner membrane translocase subunit Tim8 A; n=3; Xenopus|Rep: Mitochondrial import inner membrane translocase subunit Tim8 A - Xenopus laevis (African clawed frog)	GO:0005739<br>GO:0005743<br>GO:0006626<br>GO:0006810<br>GO:0008270<br>GO:0015031<br>GO:0016020<br>GO:0042719<br>GO:0045039<br>GO:0046872	2e-33	72%<br>(68/94)	FL634705<br>ES783421
sb_gmnlmfta.79.C1	2	sb_gmnlmfta_0001h12.t7<br>sb_gmnlmfta_0003b07.t7	Cluster: Zgc:100918; n=3; Euteleostomi|Rep: Zgc:100918 - Danio rerio (Zebrafish) (Brachydanio rerio)		1e-84	98%<br>(153/155)	ES783769<br>ES783678
sb_gmnlmfta.80.C1	2	sb_gmnlmfta_0005d07.t7<br>sb_gmnlmfta_0004g08.t7	Cluster: Zgc:91910; n=6; Euteleostomi|Rep: Zgc:91910 - Danio rerio (Zebrafish) (Brachydanio rerio)		2e-18	84%<br>(42/50)	FL634640<br>ES783996
sb_gmnlmfta.81.C1	2	sb_gmnlmfta_0002i06.t7<br>sb_gmnlmfta_0002c06.t7	Cluster: F-actin capping protein subunit beta (CapZ beta).; n=1; Xenopus tropicalis|Rep: F-actin capping protein subunit beta (CapZ beta). - Xenopus tropicalis		8e-05	85%<br>(24/28)	ES783440<br>ES783958
sb_gmnlmfta.82.C1	2	sb_gmnlmfta_0003e05.t7<br>sb_gmnlmfta_0003o09.t7	unclassified				ES783691<br>ES784040
sb_gmnlmfta.83.C1	2	sb_gmnlmfta_0005j11.t7<br>sb_gmnlmfta_0005l12.t7	unclassified				FL634767<br>FL634810

sb_gmnlmfta.85.C1	2	sb_gmnlmfta_0003i07.t7<br>sb_gmnlmfta_0002a22.t7	unclassified				ES783306<br>ES784004
sb_gmnlmfta.86.C1	2	sb_gmnlmfta_0004l10.t7<br>sb_gmnlmfta_0004p06.t7	Cluster: C20orf24 homolog; n=2; Danio rerio|Rep: C20orf24 homolog - Danio rerio (Zebrafish) (Brachydanio rerio)		3e-17	75%<br>(40/53)	ES783624<br>ES784200
sb_gmnlmfta.88.C1	2	sb_gmnlmfta_0003k08.t7<br>sb_gmnlmfta_0003m02.t7	unclassified				ES783332<br>ES783860
sb_gmnlmfta.89.C1	2	sb_gmnlmfta_0004a17.t7<br>sb_gmnlmfta_0005d09.t7	unclassified				ES784225<br>FL634641
sb_gmnlmfta.91.C1	2	sb_gmnlmfta_0002c14.t7<br>sb_gmnlmfta_0002n12.t7	Cluster: 60S ribosomal protein L31; n=7; Euteleostomi|Rep: 60S ribosomal protein L31 - Ictalurus punctatus (Channel catfish)	GO:0003735<br>GO:0005622<br>GO:0005840<br>GO:0006412	4e-37	93%<br>(74/79)	ES783580<br>ES784333
sb_gmnlmfta.92.C1	2	sb_gmnlmfta_0003l15.t7<br>sb_gmnlmfta_0003o18.t7	unclassified				ES783939<br>ES783545
sb_gmnlmfta.93.C1	2	sb_gmnlmfta_0004a14.t7<br>sb_gmnlmfta_0004o18.t7	unclassified				ES784269<br>ES783806
sb_gmnlmfta.95.C1	2	sb_gmnlmfta_0005h04.t7<br>sb_gmnlmfta_0005c13.t7	Cluster: Fatty acid-binding protein, heart; n=2; Salmonidae|Rep: Fatty acid-binding protein, heart - Oncorhynchus mykiss (Rainbow trout) (Salmo gairdneri)	GO:0005215<br>GO:0005488<br>GO:0005737<br>GO:0006810	4e-52	73%<br>(98/133)	FL634718<br>FL634624
sb_gmnlmfta.96.C1	2	sb_gmnlmfta_0002i22.t7<br>sb_gmnlmfta_0002l02.t7	unclassified				ES783696<br>ES783649
sb_gmnlmfta.97.C1	2	sb_gmnlmfta_0003m01.t7<br>sb_gmnlmfta_0002e20.t7	Cluster: Phosphoribosylaminoimidazole carboxylase, phosphoribosylaminoimidazole succinocarboxamide synthetase; n=2; Danio rerio|Rep: Phosphoribosylaminoimidazole carboxylase, phosphoribosylaminoimidazole succinocarboxamide synthetase -		4e-85	81%<br>(149/183)	ES783805<br>ES783311

			Danio rerio (Zebrafish) (Brachydanio rerio)				
sb_gmnlmfta.98.C1	2	sb_gmnlmfta_0004n11.t7<br>sb_gmnlmfta_0001a11.t7	Cluster: T-complex protein 1, alpha subunit; n=6; Danio rerio|Rep: T-complex protein 1, alpha subunit - Danio rerio (Zebrafish) (Brachydanio rerio)		7e-86	93% (163/174)	ES783289<br>ES783589
sb_gmnlmfta_0001a01.t7	1		unclassified				ES783930
sb_gmnlmfta_0001a05.t7	1		Unassigned protein				ES783891
sb_gmnlmfta_0001a07.t7	1		unclassified				ES783839
sb_gmnlmfta_0001b01.t7	1		unclassified				ES783334
sb_gmnlmfta_0001b06.t7	1		Cluster: Homolog of Cyprinus carpio "Alpha-2-macroglobulin-1.; n=1; Takifugu rubripes|Rep: Homolog of Cyprinus carpio "Alpha-2-macroglobulin-1. - Takifugu rubripes		7e-51	68% (99/145)	ES783467
sb_gmnlmfta_0001b12.t7	1		unclassified				ES784282
sb_gmnlmfta_0001c01.t7	1		unclassified				ES784280
sb_gmnlmfta_0001c08.t7	1		Atg8a; Autophagy-specific gene 8a; K08341 GABA(A) receptor-associated protein (autophagy-related protein 8)		9e-34	93% (59/63)	ES783858
sb_gmnlmfta_0001d03.t7	1		Cluster: 40S ribosomal protein S11; n=14; Euteleostomi|Rep: 40S ribosomal protein S11 - Xenopus laevis (African clawed frog)	GO:0003723<br>GO:0003735<br>GO:0005622<br>GO:0005840<br>GO:0006412<br>GO:0019843<br>GO:0030529	7e-34	87% (62/71)	ES784334

sb_gmnlmfta_0001d04.t7	1		unclassified				ES784198
sb_gmnlmfta_0001d08.t7	1		MGC82295; cytochrome c oxidase subunit Vb [EC:1.9.3.1]; K02265 cytochrome c oxidase subunit Vb		1e-21	74% (40/54)	ES783820
sb_gmnlmfta_0001d09.t7	1		unclassified				ES783837
sb_gmnlmfta_0001e06.t7	1		Cluster: UPI0000D8D6C0 related cluster; n=2; Danio rerio|Rep: UPI0000D8D6C0 UniRef100 entry - Danio rerio		2e-36	56% (76/135)	ES783409
sb_gmnlmfta_0001e12.t7	1		Cluster: PREDICTED: similar to Ribosomal protein S27-like; n=1; Monodelphis domestica|Rep: PREDICTED: similar to Ribosomal protein S27-like - Monodelphis domestica		5e-16	100% (23/23)	ES784219
sb_gmnlmfta_0001f07.t7	1		Cluster: PREDICTED: similar to T-complex protein 1 subunit alpha (TCP-1-alpha) (CCT-alpha) isoform 1; n=1; Apis mellifera|Rep: PREDICTED: similar to T-complex protein 1 subunit alpha (TCP-1-alpha) (CCT-alpha) isoform 1 - Apis mellifera		1e-36	80% (46/57)	ES783904
sb_gmnlmfta_0001f08.t7	1		Cluster: Sarcoplasmic/endoplasmic reticulum calcium ATPase 1 (EC 3.6.3.8) (Calcium pump 1) (SERCA1) (SR Ca(2+)-ATPase 1) (Calcium-transporting ATPase sarcoplasmic reticulum type, fast twitch skeletal muscle isoform) (Endoplasmic reticulum class 1/2 Ca(2+) ATPase); n=6; Holacanthopterygii|Rep: Sarcoplasmic/endoplasmic reticulum calcium ATPase 1 (EC 3.6.3.8) (Calcium pump 1) (SERCA1) (SR Ca(2+)-ATPase 1) (Calcium-transporting ATPase sarcoplasmic reticulum type, fast twitch skeletal muscle isoform) (Endoplasmic reticulum class 1/2 Ca(2+) ATPase) - Makaira nigricans (Blue marlin)	GO:0000166 GO:0000287 GO:0003824 GO:0005388 GO:0005509 GO:0005515 GO:0005524 GO:0005783 GO:0006810 GO:0006811 GO:0006812 GO:0006816 GO:0008152 GO:0015662 GO:0016020 GO:0016021 GO:0016529 GO:0016787 GO:0016820 GO:0031448 GO:0046872 GO:0005789 GO:0033017	3e-30	76% (40/52)	ES784146
sb_gmnlmfta_0001f09.t7	1		unclassified				ES784167

sb_gmnlmfta_0001f10.t7	1		Cluster: Type I cytokeratin; n=3; Danio rerio|Rep: Type I cytokeratin - Brachydanio rerio (Zebrafish) (Danio rerio)		1e-66	70% (132/186)	ES783573
sb_gmnlmfta_0001f11.t7	1		unclassified				ES783554
sb_gmnlmfta_0001f12.t7	1		Cluster: CCAAT/enhancer binding protein (C/EBP), delta; n=4; Danio rerio|Rep: CCAAT/enhancer binding protein (C/EBP), delta - Brachydanio rerio (Zebrafish) (Danio rerio)	GO:0003677 GO:0003700 GO:0005634 GO:0006355 GO:0043565	4e-24	76% (54/71)	ES783590
sb_gmnlmfta_0001g02.t7	1		unclassified				ES783523
sb_gmnlmfta_0001g07.t7	1		Cluster: Myosin heavy chain; n=3; Gadidae|Rep: Myosin heavy chain - Theragra chalcogramma (Alaska pollock)	GO:0003774 GO:0005524 GO:0016459	2e-17	100% (30/30)	ES783767
sb_gmnlmfta_0001g08.t7	1		unclassified				ES783469
sb_gmnlmfta_0001g10.t7	1		Cluster: Homolog of Homo sapiens "Palladin; n=1; Takifugu rubripes|Rep: Homolog of Homo sapiens "Palladin - Takifugu rubripes		1e-23	76% (36/47)	ES783983
sb_gmnlmfta_0001g11.t7	1		unclassified				ES783965
sb_gmnlmfta_0001h04.t7	1		Cluster: PREDICTED: similar to protein kinase PRK2; n=2; Gallus gallus|Rep: PREDICTED: similar to protein kinase PRK2 - Gallus gallus		2e-17	81% (45/55)	ES783932
sb_gmnlmfta_0001h06.t7	1		unclassified				ES784047
sb_gmnlmfta_0002a04.t7	1		Cluster: Carnitine octanoyltransferase; n=2; Takifugu rubripes|Rep: Carnitine octanoyltransferase - Fugu rubripes (Japanese pufferfish)	GO:0000062 GO:0005488 GO:0008415	2e-80	73% (104/141)	ES784255

			(Takifugu rubripes)	GO:0016740			
sb_gmnlmfta_0002a07.t7	1		SPRY domain containing protein		5e-08	27% (22/80)	ES784310
sb_gmnlmfta_0002a11.t7	1		unclassified				ES783413
sb_gmnlmfta_0002a17.t7	1		unclassified				ES783297
sb_gmnlmfta_0002a24.t7	1		unclassified				ES783915
sb_gmnlmfta_0002b03.t7	1		Cluster: Homolog of Brachydanio rerio "Solute carrier family 3, member 2.; n=1; Takifugu rubripes|Rep: Homolog of Brachydanio rerio "Solute carrier family 3, member 2. - Takifugu rubripes		2e-10	61% (33/54)	ES783783
sb_gmnlmfta_0002b04.t7	1		unclassified				ES783640
sb_gmnlmfta_0002b13.t7	1		unclassified				ES783856
sb_gmnlmfta_0002b15.t7	1		unclassified				ES783940
sb_gmnlmfta_0002b17.t7	1		Cluster: Cysteine-rich PDZ-binding protein; n=17; Euteleostomi|Rep: Cysteine-rich PDZ-binding protein - Homo sapiens (Human)		2e-42	89% (81/91)	ES784037
sb_gmnlmfta_0002b18.t7	1		unclassified				ES784294
sb_gmnlmfta_0002b19.t7	1		Cluster: Cytoplasmic dynein light chain; n=1; Aedes aegypti|Rep: Cytoplasmic dynein light chain - Aedes aegypti (Yellowfever mosquito)	GO:0003777 GO:0005875 GO:0007017	6e-10	83% (26/31)	ES784307

sb_gmnlmfta_0002c04.t7	1		unclassified				ES784053
sb_gmnlmfta_0002c08.t7	1		unclassified				ES784231
sb_gmnlmfta_0002c09.t7	1		unclassified				ES784241
sb_gmnlmfta_0002c10.t7	1		unclassified				ES783764
sb_gmnlmfta_0002c16.t7	1		unclassified				ES783613
sb_gmnlmfta_0002c20.t7	1		unclassified				ES784317
sb_gmnlmfta_0002d04.t7	1		Cluster: Alpha 2 actin; n=2; Bos taurus|Rep: Alpha 2 actin - Bos taurus (Bovine)		2e-79	96% (146/152)	ES783967
sb_gmnlmfta_0002d07.t7	1		unclassified				ES784049
sb_gmnlmfta_0002d11.t7	1		Cluster: Homolog of Homo sapiens "myosin binding protein C, fast type; n=1; Takifugu rubripes|Rep: Homolog of Homo sapiens "myosin binding protein C, fast type - Takifugu rubripes		5e-20	62% (49/78)	ES783658
sb_gmnlmfta_0002d12.t7	1		Unassigned protein				ES783745
sb_gmnlmfta_0002d15.t7	1		Cluster: PREDICTED: similar to lung cancer oncogene 7 isoform 1; n=1; Pan troglodytes|Rep: PREDICTED: similar to lung cancer oncogene 7 isoform 1 - Pan troglodytes		5e-66	92% (120/130)	ES783617

sb_gmnlmfta_0002d24.t7	1		Cluster: Elongation factor 1-beta; n=4; Xenopus|Rep: Elongation factor 1-beta - Xenopus tropicalis (Western clawed frog) (Silurana tropicalis)	GO:0003746 GO:0005853 GO:0006412 GO:0006414	4e-06	82% (23/28)	ES784171
sb_gmnlmfta_0002e05.t7	1		unclassified				ES783548
sb_gmnlmfta_0002e10.t7	1		Cluster: MKI67 FHA domain-interacting nucleolar phosphoprotein-like; n=2; Danio rerio|Rep: MKI67 FHA domain-interacting nucleolar phosphoprotein-like - Brachydanio rerio (Zebrafish) (Danio rerio)		4e-43	61% (92/150)	ES783853
sb_gmnlmfta_0002e11.t7	1		Cluster: Homolog of Brachydanio rerio "Plexin D1.; n=1; Takifugu rubripes|Rep: Homolog of Brachydanio rerio "Plexin D1. - Takifugu rubripes		2e-34	68% (64/94)	ES783794
sb_gmnlmfta_0002e15.t7	1		W2 domain containing protein		2e-05	51% (14/27)	ES784031
sb_gmnlmfta_0002f05.t7	1		unclassified				ES784298
sb_gmnlmfta_0002f14.t7	1		eIF-5a domain containing protein		1e-04	50% (14/28)	ES783295
sb_gmnlmfta_0002f16.t7	1		Cluster: Apolipoprotein B; n=1; Salmo salar|Rep: Apolipoprotein B - Salmo salar (Atlantic salmon)		6e-65	61% (114/186)	ES783328
sb_gmnlmfta_0002f17.t7	1		Cluster: Parvalbumin; n=1; Theragra chalcogramma|Rep: Parvalbumin - Theragra chalcogramma (Alaska pollock)	GO:0005509	1e-17	79% (53/67)	ES783377
sb_gmnlmfta_0002f20.t7	1		Cluster: Novel protein similar to vertebrate coenzyme Q9 homolog; n=3; Danio rerio|Rep: Novel protein similar to vertebrate coenzyme Q9 homolog - Brachydanio rerio (Zebrafish) (Danio rerio)		2e-62	56% (135/240)	ES784055
sb_gmnlmfta_0002f21.t7	1		Cluster: Zgc:114044; n=12; Euteleostomi|Rep: Zgc:114044 - Brachydanio rerio (Zebrafish) (Danio rerio)		1e-50	94% (94/100)	ES784006

sb_gmnlmfta_0002f23.t7	1		Cluster: 60S ribosomal protein L6; n=5; Percomorpha|Rep: 60S ribosomal protein L6 - Pagrus major (Red sea bream) (Chrysophrys major)	GO:0003735 GO:0005622 GO:0005840 GO:0006412 GO:0030529	8e-45	73% (97/132)	ES783978
sb_gmnlmfta_0002g09.t7	1		Cluster: Zgc:114044; n=12; Euteleostomi|Rep: Zgc:114044 - Brachydanio rerio (Zebrafish) (Danio rerio)		2e-33	91% (68/74)	ES783578
sb_gmnlmfta_0002g12.t7	1		unclassified				ES784089
sb_gmnlmfta_0002g22.t7	1		Cluster: Alpha 2 actin; n=2; Bos taurus|Rep: Alpha 2 actin - Bos taurus (Bovine)		2e-31	95% (61/64)	ES783542
sb_gmnlmfta_0002h03.t7	1		Cluster: Muscle creatine kinase; n=15; Euteleostomi|Rep: Muscle creatine kinase - Brachydanio rerio (Zebrafish) (Danio rerio)		6e-14	80% (38/47)	ES784289
sb_gmnlmfta_0002h05.t7	1		Cluster: PREDICTED: titin-like; n=1; Danio rerio|Rep: PREDICTED: titin-like - Danio rerio		9e-38	52% (93/177)	ES784173
sb_gmnlmfta_0002h06.t7	1		unclassified				ES784110
sb_gmnlmfta_0002h08.t7	1		unclassified				ES783842
sb_gmnlmfta_0002i02.t7	1		Cluster: PREDICTED: similar to Transforming growth factor, beta-induced; n=1; Danio rerio|Rep: PREDICTED: similar to Transforming growth factor, beta-induced - Danio rerio		7e-75	71% (137/192)	ES783367
sb_gmnlmfta_0002i03.t7	1		Cluster: Alpha 2 actin; n=2; Bos taurus|Rep: Alpha 2 actin - Bos taurus (Bovine)		2e-24	96% (29/30)	ES783345
sb_gmnlmfta_0002i09.t7	1		Cluster: PREDICTED: similar to Tropomyosin 1 alpha chain (Alpha-tropomyosin) isoform 7; n=5; Eutheria|Rep: PREDICTED: similar to Tropomyosin 1 alpha chain (Alpha-tropomyosin) isoform 7 - Canis familiaris		6e-24	88% (56/63)	ES783662

sb_gmnlmfta_0002i11.t7	1		unclassified				ES784331
sb_gmnlmfta_0002i12.t7	1		LOC479308; similar to step II splicing factor SLU7		7e-28	41% (64/154)	ES784244
sb_gmnlmfta_0002i19.t7	1		unclassified				ES783876
sb_gmnlmfta_0002i20.t7	1		Cluster: FK506 binding protein 3, 25kDa; n=3; Xenopus|Rep: FK506 binding protein 3, 25kDa - Xenopus tropicalis (Western clawed frog) (Silurana tropicalis)	GO:0006457 GO:0003755	7e-31	61% (66/107)	ES783730
sb_gmnlmfta_0002j02.t7	1		unclassified				ES783973
sb_gmnlmfta_0002j03.t7	1		Cluster: PREDICTED: similar to Myosin regulatory light chain 2, smooth muscle isoform (Myosin RLC) (LC20); n=1; Canis familiaris|Rep: PREDICTED: similar to Myosin regulatory light chain 2, smooth muscle isoform (Myosin RLC) (LC20) - Canis familiaris		6e-52	92% (96/104)	ES783953
sb_gmnlmfta_0002j04.t7	1		unclassified				ES783818
sb_gmnlmfta_0002j05.t7	1		Cluster: Ahnak protein; n=9; Murinae|Rep: Ahnak protein - Mus musculus (Mouse)		2e-15	32% (64/198)	ES783821
sb_gmnlmfta_0002j09.t7	1		unclassified				ES784139
sb_gmnlmfta_0002j11.t7	1		Cluster: FGF receptor-like protein 1a; n=2; Takifugu rubripes|Rep: FGF receptor-like protein 1a - Fugu rubripes (Japanese pufferfish) (Takifugu rubripes)	GO:0004872	3e-07	88% (24/27)	ES783582
sb_gmnlmfta_0002j15.t7	1		Cluster: Myosin heavy chain; n=3; Gadidae|Rep: Myosin heavy chain - Theragra chalcogramma (Alaska pollock)	GO:0003774 GO:0005524 GO:0016459	2e-89	90% (163/180)	ES783756

sb_gmnlmfta_0002j16.t7	1		Cluster: PREDICTED: similar to alpha 3-actin; n=1; Monodelphis domestica|Rep: PREDICTED: similar to alpha 3-actin - Monodelphis domestica		2e-33	98% (68/69)	ES783708
sb_gmnlmfta_0002j20.t7	1		unclassified				ES784070
sb_gmnlmfta_0002k02.t7	1		Unassigned protein				ES783568
sb_gmnlmfta_0002k11.t7	1		Unassigned protein				ES783994
sb_gmnlmfta_0002k15.t7	1		Cluster: Ubiquinol-cytochrome c reductase core protein II; n=3; Danio rerio|Rep: Ubiquinol-cytochrome c reductase core protein II - Brachydanio rerio (Zebrafish) (Danio rerio)		3e-20	70% (45/64)	ES783893
sb_gmnlmfta_0002k18.t7	1		MGC76326; similar to calsequestrin 2 (cardiac muscle)		5e-12	68% (31/45)	ES784113
sb_gmnlmfta_0002k21.t7	1		unclassified				ES783446
sb_gmnlmfta_0002k23.t7	1		Abhydrolase_2 domain containing protein		4e-11	32% (45/139)	ES783476
sb_gmnlmfta_0002l01.t7	1		unclassified				ES783571
sb_gmnlmfta_0002l04.t7	1		Cluster: Fast skeletal muscle troponin I; n=1; Gadus morhua|Rep: Fast skeletal muscle troponin I - Gadus morhua (Atlantic cod)		7e-21	87% (29/33)	ES783736
sb_gmnlmfta_0002l07.t7	1		PTPLA; protein tyrosine phosphatase-like (proline instead of catalytic arginine), member A		1e-60	68% (115/168)	ES783677

sb_gmnlmfta_0002l09.t7	1		Cluster: Heat shock cognate 71 kDa protein; n=23; Euteleostomi|Rep: Heat shock cognate 71 kDa protein - Ictalurus punctatus (Channel catfish)	GO:0000166 GO:0005524	4e-35	82% (80/97)	ES783416
sb_gmnlmfta_0002l10.t7	1		unclassified				ES784022
sb_gmnlmfta_0002l12.t7	1		Cluster: Fructose-bisphosphate aldolase A-1; n=2; Acipenser baerii|Rep: Fructose-bisphosphate aldolase A-1 - Acipenser baerii (Siberian sturgeon)	GO:0003824 GO:0004332 GO:0006096 GO:0008152 GO:0016829	1e-27	95% (39/41)	ES783998
sb_gmnlmfta_0002l13.t7	1		Cluster: Osteonectin; n=1; Hippoglossus hippoglossus|Rep: Osteonectin - Hippoglossus hippoglossus (Atlantic halibut)	GO:0005509	2e-24	88% (47/53)	ES783933
sb_gmnlmfta_0002l14.t7	1		unclassified				ES783796
sb_gmnlmfta_0002l21.t7	1		Cluster: Nol5 protein; n=8; Danio rerio|Rep: Nol5 protein - Brachydanio rerio (Zebrafish) (Danio rerio)		6e-44	79% (86/108)	ES783509
sb_gmnlmfta_0002l24.t7	1		Cluster: Proteasome activator subunit 2; n=1; Pseudosciaena crocea|Rep: Proteasome activator subunit 2 - Pseudosciaena crocea (Croceine croaker)	GO:0005829 GO:0008537 GO:0008538 GO:0043234	2e-35	53% (82/153)	ES783267
sb_gmnlmfta_0002m03.t7	1		Cluster: Homolog of Homo sapiens "Leiomodin 3 (fetal); n=1; Takifugu rubripes|Rep: Homolog of Homo sapiens "Leiomodin 3 (fetal) - Takifugu rubripes		2e-34	90% (70/77)	ES784002
sb_gmnlmfta_0002m05.t7	1		unclassified				ES783920
sb_gmnlmfta_0002m12.t7	1		Cluster: Homolog of Brachydanio rerio "Eukaryotic translation elongation factor 1 beta 2.; n=1; Takifugu rubripes|Rep: Homolog of Brachydanio rerio "Eukaryotic translation elongation factor 1 beta 2. - Takifugu rubripes		5e-45	88% (86/97)	ES783586

sb_gmnlmfta_0002m13.t7	1		unclassified				ES783525
sb_gmnlmfta_0002n03.t7	1		Cluster: Homolog of Homo sapiens "Zinc finger protein 364; n=1; Takifugu rubripes|Rep: Homolog of Homo sapiens "Zinc finger protein 364 - Takifugu rubripes		9e-07	85% (23/27)	ES783260
sb_gmnlmfta_0002n04.t7	1		unclassified				ES783400
sb_gmnlmfta_0002n10.t7	1		Cluster: Homolog of Paralichthys olivaceus "Type 1 collagen alpha 1.; n=1; Takifugu rubripes|Rep: Homolog of Paralichthys olivaceus "Type 1 collagen alpha 1. - Takifugu rubripes		5e-63	91% (116/127)	ES784224
sb_gmnlmfta_0002n13.t7	1		unclassified				ES784270
sb_gmnlmfta_0002n16.t7	1		unclassified				ES784105
sb_gmnlmfta_0002n17.t7	1		Cluster: 40S ribosomal protein S8; n=5; Amniota|Rep: 40S ribosomal protein S8 - Oxyuranus scutellatus	GO:0005622 GO:0030529	5e-46	93% (85/91)	ES784091
sb_gmnlmfta_0002n20.t7	1		unclassified				ES783669
sb_gmnlmfta_0002n23.t7	1		Cluster: Ribosomal protein L18; n=4; Euteleostomi|Rep: Ribosomal protein L18 - Pagrus major (Red sea bream) (Chrysophrys major)	GO:0003735 GO:0005622 GO:0005840 GO:0006412	3e-10	88% (31/35)	ES783723
sb_gmnlmfta_0002o01.t7	1		unclassified				ES784321
sb_gmnlmfta_0002o05.t7	1		Cluster: Questionable orf; n=1; Candida albicans|Rep: Questionable orf - Candida albicans (Yeast)		3e-07	55% (19/34)	ES784117

sb_gmnlmfta_0002o09.t7	1		unclassified				ES783885
sb_gmnlmfta_0002o14.t7	1		unclassified				ES783461
sb_gmnlmfta_0002o16.t7	1		unclassified				ES783427
sb_gmnlmfta_0002o19.t7	1		unclassified				ES783679
sb_gmnlmfta_0002o22.t7	1		unclassified				ES783926
sb_gmnlmfta_0002p07.t7	1		unclassified				ES783520
sb_gmnlmfta_0002p10.t7	1		MGC127606; similar to solute carrier family 25, member 35		3e-30	63% (60/95)	ES784267
sb_gmnlmfta_0002p13.t7	1		Cluster: Homolog of Homo sapiens "TiTin isoform novex-2; n=1; Takifugu rubripes|Rep: Homolog of Homo sapiens "TiTin isoform novex-2 - Takifugu rubripes		3e-43	49% (115/232)	ES784303
sb_gmnlmfta_0002p14.t7	1		unclassified				ES784168
sb_gmnlmfta_0002p15.t7	1		Cluster: Homolog of Brachydanio rerio "Eukaryotic translation elongation factor 2, like.; n=1; Takifugu rubripes|Rep: Homolog of Brachydanio rerio "Eukaryotic translation elongation factor 2, like. - Takifugu rubripes		2e-60	84% (123/145)	ES784145
sb_gmnlmfta_0002p19.t7	1		unclassified				ES783838

sb_gmnlmfta_0002p20.t7	1		unclassified				ES783707
sb_gmnlmfta_0003a03.t7	1		Cluster: Ribosomal protein L18; n=4; Euteleostomi|Rep: Ribosomal protein L18 - Pagrus major (Red sea bream) (Chrysophrys major)	GO:0003735 GO:0005622 GO:0005840 GO:0006412	1e-52	86% (101/117)	ES784204
sb_gmnlmfta_0003a04.t7	1		unclassified				ES784077
sb_gmnlmfta_0003a06.t7	1		unclassified				ES784176
sb_gmnlmfta_0003a11.t7	1		unclassified				ES783302
sb_gmnlmfta_0003a24.t7	1		unclassified				ES784011
sb_gmnlmfta_0003b01.t7	1		unclassified				ES783572
sb_gmnlmfta_0003b03.t7	1		unclassified				ES783593
sb_gmnlmfta_0003b05.t7	1		[J] COG2075 Ribosomal protein L24E		3e-32	49% (67/135)	ES783776
sb_gmnlmfta_0003b12.t7	1		Cluster: PREDICTED: similar to ribosomal protein S23; n=2; Homo/Pan/Gorilla group|Rep: PREDICTED: similar to ribosomal protein S23 - Homo sapiens		3e-14	97% (38/39)	ES783999
sb_gmnlmfta_0003b19.t7	1		unclassified				ES784161

sb_gmnlmfta_0003b22.t7	1		Cluster: Heat shock 70kDa protein 8 isoform 2 variant; n=48; Metazoa|Rep: Heat shock 70kDa protein 8 isoform 2 variant - Homo sapiens (Human)		9e-82	95% (153/161)	ES783452
sb_gmnlmfta_0003b24.t7	1		unclassified				ES783269
sb_gmnlmfta_0003c04.t7	1		unclassified				ES783870
sb_gmnlmfta_0003c06.t7	1		Cluster: Guanine nucleotide-binding protein G(q) subunit alpha; n=17; Euteleostomi|Rep: Guanine nucleotide-binding protein G(q) subunit alpha - Homo sapiens (Human)	GO:0000166 GO:0004871 GO:0005525 GO:0006471 GO:0007165 GO:0007186	6e-77	87% (145/166)	ES783826
sb_gmnlmfta_0003c07.t7	1		Cluster: PREDICTED: microfibrillar associated protein 5 isoform 2; n=2; Catarrhini|Rep: PREDICTED: microfibrillar associated protein 5 isoform 2 - Macaca mulatta		3e-12	48% (31/64)	ES783816
sb_gmnlmfta_0003c19.t7	1		unclassified				ES783480
sb_gmnlmfta_0003c20.t7	1		Cluster: COP9 signalosome complex subunit 3; n=22; Euteleostomi|Rep: COP9 signalosome complex subunit 3 - Homo sapiens (Human)		3e-75	92% (129/139)	ES784149
sb_gmnlmfta_0003c21.t7	1		Cluster: Homolog of Homo sapiens "Methylosome protein 50; n=1; Takifugu rubripes|Rep: Homolog of Homo sapiens "Methylosome protein 50 - Takifugu rubripes		8e-14	78% (32/41)	ES784183
sb_gmnlmfta_0003c22.t7	1		Cluster: Alpha-tropomyosin; n=1; Danio rerio|Rep: Alpha-tropomyosin - Brachydanio rerio (Zebrafish) (Danio rerio)		4e-12	90% (37/41)	ES784126
sb_gmnlmfta_0003c23.t7	1		unclassified				ES784066

sb_gmnlmfta_0003d04.t7	1		unclassified				ES783819
sb_gmnlmfta_0003d05.t7	1		Cluster: Myosin heavy chain; n=3; Gadidae|Rep: Myosin heavy chain - Theragra chalcogramma (Alaska pollock)	GO:0003774<br>GO:0005524<br>GO:0016459<br>GO:0000166	7e-38	96% (74/77)	ES783822
sb_gmnlmfta_0003d08.t7	1		Cluster: 60S ribosomal protein L7a; n=1; Ictalurus punctatus|Rep: 60S ribosomal protein L7a - Ictalurus punctatus (Channel catfish)	GO:0003735<br>GO:0005622<br>GO:0005840<br>GO:0006412<br>GO:0030529<br>GO:0042254	1e-36	91% (75/82)	ES784188
sb_gmnlmfta_0003d11.t7	1		Troponin multi-domain protein		3e-18	38% (30/77)	ES783584
sb_gmnlmfta_0003d23.t7	1		Cluster: Zgc:101755; n=3; Danio rerio|Rep: Zgc:101755 - Brachydanio rerio (Zebrafish) (Danio rerio)		2e-34	87% (67/77)	ES784155
sb_gmnlmfta_0003e02.t7	1		unclassified				ES783569
sb_gmnlmfta_0003e04.t7	1		unclassified				ES783682
sb_gmnlmfta_0003e06.t7	1		Cluster: PREDICTED: myelin basic protein isoform 2; n=3; Danio rerio|Rep: PREDICTED: myelin basic protein isoform 2 - Danio rerio		8e-08	38% (36/94)	ES783770
sb_gmnlmfta_0003e10.t7	1		Cluster: Annexin A6; n=5; Danio rerio|Rep: Annexin A6 - Brachydanio rerio (Zebrafish) (Danio rerio)		6e-91	78% (169/215)	ES783937
sb_gmnlmfta_0003e20.t7	1		unclassified				ES783398
sb_gmnlmfta_0003e21.t7	1		Cluster: 26S proteasome non-ATPase regulatory subunit 14; n=15; Coelomata|Rep: 26S proteasome non-ATPase regulatory subunit 14		2e-76	88%	ES783448

			- Homo sapiens (Human)			(123/139)	
sb_gmnlmfta_0003e24.t7	1		Cluster: Alpha 2 actin; n=2; Bos taurus|Rep: Alpha 2 actin - Bos taurus (Bovine)		1e-37	98% (76/77)	ES783350
sb_gmnlmfta_0003f05.t7	1		unclassified				ES784172
sb_gmnlmfta_0003f07.t7	1		Cluster: Homolog of Brachydanio rerio "Calcium channel, voltage-dependent, gamma subunit 1.; n=1; Takifugu rubripes|Rep: Homolog of Brachydanio rerio "Calcium channel, voltage-dependent, gamma subunit 1. - Takifugu rubripes		5e-33	80% (65/81)	ES784082
sb_gmnlmfta_0003f15.t7	1		unclassified				ES783426
sb_gmnlmfta_0003f18.t7	1		Cluster: Homolog of Homo sapiens "PREDICTED "zinc finger protein 292; n=1; Takifugu rubripes|Rep: Homolog of Homo sapiens "PREDICTED "zinc finger protein 292 - Takifugu rubripes		3e-53	65% (109/166)	ES783773
sb_gmnlmfta_0003f19.t7	1		Cluster: PREDICTED: connective tissue growth factor; n=1; Macaca mulatta|Rep: PREDICTED: connective tissue growth factor - Macaca mulatta		2e-30	69% (60/86)	ES783737
sb_gmnlmfta_0003f24.t7	1		Cluster: Ribosomal protein L7; n=1; Petromyzon marinus|Rep: Ribosomal protein L7 - Petromyzon marinus (Sea lamprey)	GO:0003735 GO:0005622 GO:0005840 GO:0006412 GO:0015934 GO:0030528 GO:0030529	2e-12	78% (30/38)	ES783961
sb_gmnlmfta_0003g04.t7	1		unclassified				ES783485
sb_gmnlmfta_0003g05.t7	1		gbas; glioblastoma amplified sequence		6e-71	91% (124/135)	ES783490
sb_gmnlmfta_0003g09.t7	1		unclassified				ES783664

sb_gmnlmfta_0003g11.t7	1		Cluster: Homolog of Brachydanio rerio "Unc-45 (C. elegans) related.; n=1; Takifugu rubripes|Rep: Homolog of Brachydanio rerio "Unc-45 (C. elegans) related. - Takifugu rubripes		2e-33	80% (68/84)	ES784329
sb_gmnlmfta_0003g13.t7	1		Cluster: Inducible heat shock protein 70; n=2; Sparidae|Rep: Inducible heat shock protein 70 - Rhabdosargus sarba (goldlined seabream)	GO:0000166 GO:0005524	2e-27	77% (58/75)	ES784226
sb_gmnlmfta_0003g14.t7	1		Cluster: Homolog of Brachydanio rerio "Phosphofructokinase, muscle.; n=1; Takifugu rubripes|Rep: Homolog of Brachydanio rerio "Phosphofructokinase, muscle. - Takifugu rubripes		7e-82	88% (150/169)	ES784095
sb_gmnlmfta_0003g15.t7	1		Cluster: PREDICTED: similar to Proteasome subunit beta type 6 precursor (Proteasome delta chain) (Macropain delta chain) (Multicatalytic endopeptidase complex delta chain) (Proteasome subunit Y); n=1; Tribolium castaneum|Rep: PREDICTED: similar to Proteasome subunit beta type 6 precursor (Proteasome delta chain) (Macropain delta chain) (Multicatalytic endopeptidase complex delta chain) (Proteasome subunit Y) - Tribolium castaneum	GO:0004175 GO:0004298 GO:0005737 GO:0005829 GO:0005839 GO:0006511 GO:0008233 GO:0016787	1e-26	63% (55/87)	ES784101
sb_gmnlmfta_0003g17.t7	1		Cluster: Homolog of Homo sapiens "Ubiquinol-cytochrome c reductase complex 7.2 kDa protein; n=1; Takifugu rubripes|Rep: Homolog of Homo sapiens "Ubiquinol-cytochrome c reductase complex 7.2 kDa protein - Takifugu rubripes	GO:0005740 GO:0006122	2e-19	71% (40/56)	ES784140
sb_gmnlmfta_0003g21.t7	1		unclassified				ES783781
sb_gmnlmfta_0003g22.t7	1		unclassified				ES783698
sb_gmnlmfta_0003g23.t7	1		unclassified				ES783675
sb_gmnlmfta_0003h09.t7	1		Unassigned protein				ES783987
sb_gmnlmfta_0003h20.t7	1		Cluster: 40S ribosomal protein S8; n=2; Gillichthys mirabilis|Rep: 40S ribosomal protein S8 - Gillichthys mirabilis (Long-jawed mudsucker)	GO:0005622	1e-23	78% (36/46)	ES784056

sb_gmnlmfta_0003h23.t7	1		unclassified				ES783976
sb_gmnlmfta_0003i05.t7	1		Unassigned protein				ES783355
sb_gmnlmfta_0003i10.t7	1		Cluster: Myosin heavy chain; n=3; Gadidae|Rep: Myosin heavy chain - Theragra chalcogramma (Alaska pollock)	GO:0003774 GO:0005524 GO:0016459	7e-42	100% (84/84)	ES784193
sb_gmnlmfta_0003i12.t7	1		unclassified				ES784088
sb_gmnlmfta_0003i18.t7	1		unclassified				ES784018
sb_gmnlmfta_0003i20.t7	1		unclassified				ES783648
sb_gmnlmfta_0003j01.t7	1		Cluster: 26S protease regulatory subunit 6B; n=30; Euteleostomi|Rep: 26S protease regulatory subunit 6B - Homo sapiens (Human)		2e-59	88% (118/133)	ES783866
sb_gmnlmfta_0003j04.t7	1		Cluster: Elongation factor 1-alpha; n=36; Vertebrata|Rep: Elongation factor 1-alpha - Brachydanio rerio (Zebrafish) (Danio rerio)		4e-08	96% (28/29)	ES783966
sb_gmnlmfta_0003j07.t7	1		unclassified				ES784051
sb_gmnlmfta_0003j15.t7	1		unclassified				ES783618
sb_gmnlmfta_0003j17.t7	1		Cluster: Homolog of Homo sapiens "Synaptopodin-2 (Myopodin) (Genethonin 2).; n=1; Takifugu rubripes|Rep: Homolog of Homo sapiens "Synaptopodin-2 (Myopodin) (Genethonin 2). - Takifugu rubripes		6e-13	50% (42/84)	ES783576

sb_gmnlmfta_0003j18.t7	1		unclassified				ES783274
sb_gmnlmfta_0003j19.t7	1		Cluster: Zgc:136473; n=3; Danio rerio|Rep: Zgc:136473 - Brachydanio rerio (Zebrafish) (Danio rerio)		7e-28	91% (51/56)	ES783304
sb_gmnlmfta_0003j22.t7	1		unclassified				ES784291
sb_gmnlmfta_0003k04.t7	1		Cluster: Polymerase delta interacting protein 38; n=2; Danio rerio|Rep: Polymerase delta interacting protein 38 - Brachydanio rerio (Zebrafish) (Danio rerio)		2e-13	82% (34/41)	ES783556
sb_gmnlmfta_0003k14.t7	1		MGC128797; similar to Nuclear protein 1 (Protein p8) (Candidate of metastasis 1)		2e-07	45% (27/59)	ES784035
sb_gmnlmfta_0003k23.t7	1		unclassified				ES783370
sb_gmnlmfta_0003k24.t7	1		unclassified				ES783495
sb_gmnlmfta_0003l02.t7	1		unclassified				ES783727
sb_gmnlmfta_0003l05.t7	1		unclassified				ES783602
sb_gmnlmfta_0003l06.t7	1		unclassified				ES783543
sb_gmnlmfta_0003l07.t7	1		Cluster: Parvalbumin beta; n=3; Gadiformes|Rep: Parvalbumin beta - Merlangius merlangus (Whiting)	GO:0005509 GO:0000166 GO:0000287 GO:0004550 GO:0005524 GO:0006183 GO:0006228 GO:0006241	2e-40	88% (86/97)	ES783561

				GO:0009117 GO:0016301 GO:0016740			
sb_gmnlmfta_0003l11.t7	1		Cluster: Homolog of Homo sapiens "Myomesin 2; n=1; Takifugu rubripes|Rep: Homolog of Homo sapiens "Myomesin 2 - Takifugu rubripes		5e-55	75% (104/137)	ES783878
sb_gmnlmfta_0003l12.t7	1		UBC; ubiquitin C; K08770 ubiquitin C		1e-114	98% (211/214)	ES783790
sb_gmnlmfta_0003l13.t7	1		Cluster: Electron-transfer-flavoprotein beta polypeptide; n=1; Scophthalmus maximus|Rep: Electron-transfer-flavoprotein beta polypeptide - Scophthalmus maximus (Turbot)	GO:0006118 GO:0009055	1e-52	77% (110/142)	ES783855
sb_gmnlmfta_0003m03.t7	1		TUSC2, PDAP2; tumor suppressor candidate 2		6e-22	76% (45/59)	ES783929
sb_gmnlmfta_0003m12.t7	1		Cluster: Thioredoxin interacting protein; n=1; Danio rerio|Rep: Thioredoxin interacting protein - Brachydanio rerio (Zebrafish) (Danio rerio)		2e-18	75% (43/57)	ES783654
sb_gmnlmfta_0003m15.t7	1		unclassified				ES783532
sb_gmnlmfta_0003m19.t7	1		PH domain containing protein		3e-07	22% (23/101)	ES783364
sb_gmnlmfta_0003m22.t7	1		Cluster: Small muscle protein, X-linked; n=2; Clupeocephala|Rep: Small muscle protein, X-linked - Brachydanio rerio (Zebrafish) (Danio rerio)		1e-09	91% (32/35)	ES784201
sb_gmnlmfta_0003m23.t7	1		LOC611134; similar to cytochrome c oxidase, subunit 7a 3 [EC:1.9.3.1]; K02270 cytochrome c oxidase subunit VIIa		9e-07	47% (23/48)	ES784264
sb_gmnlmfta_0003n06.t7	1		Cluster: Myeloid leukemia differentiation protein homologue; n=1; Salmo salar|Rep: Myeloid leukemia differentiation protein homologue - Salmo salar (Atlantic salmon)	GO:0042981	4e-22	37% (70/186)	ES783349

sb_gmnlmfta_0003n10.t7	1		Unassigned protein	GO:0005125<br>GO:0005576<br>GO:0006955<br>GO:0008009			ES784109
sb_gmnlmfta_0003n12.t7	1		unclassified				ES784131
sb_gmnlmfta_0003n15.t7	1		unclassified				ES784276
sb_gmnlmfta_0003n17.t7	1		Cluster: Ornithine decarboxylase antizyme small isoform; n=3; Percomorpha|Rep: Ornithine decarboxylase antizyme small isoform - Paralichthys olivaceus (Japanese flounder)	GO:0004857<br>GO:0008073	7e-46	80% (85/105)	ES784242
sb_gmnlmfta_0003n18.t7	1		Cluster: Sarcoplasmic/endoplasmic reticulum calcium ATPase 1 (EC 3.6.3.8) (Calcium pump 1) (SERCA1) (SR Ca(2+)-ATPase 1) (Calcium-transporting ATPase sarcoplasmic reticulum type, fast twitch skeletal muscle isoform) (Endoplasmic reticulum class 1/2 Ca(2+) ATPase); n=6; Holacanthopterygii|Rep: Sarcoplasmic/endoplasmic reticulum calcium ATPase 1 (EC 3.6.3.8) (Calcium pump 1) (SERCA1) (SR Ca(2+)-ATPase 1) (Calcium-transporting ATPase sarcoplasmic reticulum type, fast twitch skeletal muscle isoform) (Endoplasmic reticulum class 1/2 Ca(2+) ATPase) - Makaira nigricans (Blue marlin)	GO:0000166<br>GO:0000287<br>GO:0003824<br>GO:0005388<br>GO:0005509<br>GO:0005515<br>GO:0005524<br>GO:0005783<br>GO:0006810<br>GO:0006811<br>GO:0006812<br>GO:0006816<br>GO:0008152<br>GO:0015662<br>GO:0016020<br>GO:0016021<br>GO:0016529<br>GO:0016787<br>GO:0016820<br>GO:0031448<br>GO:0046872<br>GO:0005789<br>GO:0033017	1e-53	84% (102/121)	ES783956
sb_gmnlmfta_0003n19.t7	1		unclassified				ES783972
sb_gmnlmfta_0003n22.t7	1		unclassified				ES783592
sb_gmnlmfta_0003n23.t7	1		unclassified				ES783651

sb_gmnlmfta_0003o02.t7	1		unclassified				ES784067
sb_gmnlmfta_0003o05.t7	1		CHCHD6; coiled-coil-helix-coiled-coil-helix domain containing 6		6e-11	41% (34/81)	ES784210
sb_gmnlmfta_0003o13.t7	1		unclassified				ES783519
sb_gmnlmfta_0003o15.t7	1		Cluster: 3-oxoacid CoA transferase 1; n=4; Clupeocephala|Rep: 3-oxoacid CoA transferase 1 - Brachydanio rerio (Zebrafish) (Danio rerio)		4e-80	83% (138/165)	ES783326
sb_gmnlmfta_0003o17.t7	1		Cluster: Calpain 1, (Mu/I) large subunit; n=3; Danio rerio|Rep: Calpain 1, (Mu/I) large subunit - Brachydanio rerio (Zebrafish) (Danio rerio)		1e-87	82% (155/188)	ES783296
sb_gmnlmfta_0003o23.t7	1		Cluster: PREDICTED: similar to Rap2c protein; n=1; Pan troglodytes|Rep: PREDICTED: similar to Rap2c protein - Pan troglodytes		2e-13	85% (35/41)	ES784060
sb_gmnlmfta_0003o24.t7	1		Cluster: Zgc:77429 protein; n=1; Danio rerio|Rep: Zgc:77429 protein - Brachydanio rerio (Zebrafish) (Danio rerio)		3e-28	65% (55/84)	ES783917
sb_gmnlmfta_0003p01.t7	1		unclassified				ES783521
sb_gmnlmfta_0003p04.t7	1		Cluster: Heat shock protein HSP 90-alpha; n=6; Clupeocephala|Rep: Heat shock protein HSP 90-alpha - Brachydanio rerio (Zebrafish) (Danio rerio)		1e-26	87% (58/66)	ES783281
sb_gmnlmfta_0003p07.t7	1		Unassigned protein				ES783335
sb_gmnlmfta_0003p08.t7	1		unclassified				ES783634

sb_gmnlmfta_0003p10.t7	1		Unassigned protein				ES784083
sb_gmnlmfta_0003p11.t7	1		Cluster: Homolog of Homo sapiens "Placental protein 11 precursor; n=1; Takifugu rubripes|Rep: Homolog of Homo sapiens "Placental protein 11 precursor - Takifugu rubripes		2e-18	67% (43/64)	ES784129
sb_gmnlmfta_0003p15.t7	1		unclassified				ES784301
sb_gmnlmfta_0003p16.t7	1		Cluster: Homolog of Paralichthys olivaceus "Type 1 collagen alpha 1.; n=1; Takifugu rubripes|Rep: Homolog of Paralichthys olivaceus "Type 1 collagen alpha 1. - Takifugu rubripes		4e-34	93% (71/76)	ES784266
sb_gmnlmfta_0003p17.t7	1		unclassified				ES784220
sb_gmnlmfta_0003p21.t7	1		Cluster: Cyclin G1; n=5; Danio rerio|Rep: Cyclin G1 - Brachydanio rerio (Zebrafish) (Danio rerio)		2e-11	80% (32/40)	ES783575
sb_gmnlmfta_0004a02.t7	1		unclassified				ES783442
sb_gmnlmfta_0004a04.t7	1		Cluster: UPI0000E4DEC7 related cluster; n=2; Danio rerio|Rep: UPI0000E4DEC7 UniRef100 entry - Danio rerio		3e-58	78% (104/132)	ES783262
sb_gmnlmfta_0004a06.t7	1		Cluster: Parvalbumin beta; n=3; Gadiformes|Rep: Parvalbumin beta - Merlangius merlangus (Whiting)	GO:0005509	1e-25	89% (58/65)	ES783373
sb_gmnlmfta_0004a09.t7	1		unclassified				ES783607
sb_gmnlmfta_0004a11.t7	1		Cluster: PREDICTED: similar to Actin, alpha 2, smooth muscle, aorta isoform 1; n=1; Gallus gallus|Rep: PREDICTED: similar to Actin, alpha 2, smooth muscle, aorta isoform 1 - Gallus gallus		3e-17	100% (43/43)	ES784104

sb_gmnlmfta_0004a12.t7	1		unclassified				ES784191
sb_gmnlmfta_0004a18.t7	1		unclassified				ES783979
sb_gmnlmfta_0004a19.t7	1		unclassified				ES783949
sb_gmnlmfta_0004b07.t7	1		LOC479738; similar to COP9 signalosome subunit 6		8e-31	87% (63/72)	ES783947
sb_gmnlmfta_0004b14.t7	1		unclassified				ES783524
sb_gmnlmfta_0004b15.t7	1		Cluster: Fast skeletal muscle troponin T; n=1; Gadus morhua|Rep: Fast skeletal muscle troponin T - Gadus morhua (Atlantic cod)		8e-12	70% (35/50)	ES783588
sb_gmnlmfta_0004b16.t7	1		unclassified				ES783631
sb_gmnlmfta_0004b21.t7	1		unclassified				ES784309
sb_gmnlmfta_0004b24.t7	1		unclassified				ES784069
sb_gmnlmfta_0004c02.t7	1		unclassified				ES783779
sb_gmnlmfta_0004c03.t7	1		Cluster: Peroxiredoxin 6; n=2; Ictalurus punctatus|Rep: Peroxiredoxin 6 - Ictalurus punctatus (Channel catfish)	GO:0016209 GO:0016491	6e-69	82% (133/161)	ES783733
sb_gmnlmfta_0004c06.t7	1		unclassified				ES783570

sb_gmnlmfta_0004c11.t7	1		Cluster: Fas; n=1; Oryzias latipes|Rep: Fas - Oryzias latipes (Medaka fish) (Japanese ricefish)	GO:0004872 GO:0004888 GO:0005515 GO:0006915 GO:0006955 GO:0007165 GO:0016020	1e-27	31% (73/230)	ES783896
sb_gmnlmfta_0004c14.t7	1		Cluster: PREDICTED: similar to paraneoplastic antigen; MA1 isoform 1; n=1; Monodelphis domestica|Rep: PREDICTED: similar to paraneoplastic antigen; MA1 isoform 1 - Monodelphis domestica		3e-20	54% (51/94)	ES783935
sb_gmnlmfta_0004c15.t7	1		unclassified				ES783997
sb_gmnlmfta_0004c18.t7	1		Cluster: Homolog of Homo sapiens "Calcineurin-binding protein calsarcin-2; n=1; Takifugu rubripes|Rep: Homolog of Homo sapiens "Calcineurin-binding protein calsarcin-2 - Takifugu rubripes		4e-39	57% (76/133)	ES784319
sb_gmnlmfta_0004c20.t7	1		Cluster: Homolog of Brachydanio rerio "Caspase 8.; n=1; Takifugu rubripes|Rep: Homolog of Brachydanio rerio "Caspase 8. - Takifugu rubripes		2e-38	42% (96/226)	ES783348
sb_gmnlmfta_0004c21.t7	1		Cluster: Zgc:64202; n=3; Danio rerio|Rep: Zgc:64202 - Brachydanio rerio (Zebrafish) (Danio rerio)		2e-11	71% (38/53)	ES783361
sb_gmnlmfta_0004c22.t7	1		MYBPC3; myosin binding protein C, cardiac		8e-47	61% (88/143)	ES783310
sb_gmnlmfta_0004c23.t7	1		unclassified				ES783266
sb_gmnlmfta_0004d02.t7	1		Cluster: Myosin light chain 3; n=1; Theragra chalcogramma|Rep: Myosin light chain 3 - Theragra chalcogramma (Alaska pollock)	GO:0005509	8e-26	100% (37/37)	ES783688
sb_gmnlmfta_0004d04.t7	1		Cluster: Fast skeletal muscle troponin T; n=1; Gadus morhua|Rep: Fast skeletal muscle troponin T - Gadus morhua (Atlantic cod)		3e-11	94% (34/36)	ES783541

sb_gmnlmfta_0004d06.t7	1		Cluster: Novel protein similar to vertebrate thrombospondin 1. ; n=2; Danio rerio|Rep: Novel protein similar to vertebrate thrombospondin 1. - Brachydanio rerio (Zebrafish) (Danio rerio)		2e-71	76% (125/164)	ES783647
sb_gmnlmfta_0004d09.t7	1		Paf1 domain containing protein		2e-06	20% (20/96)	ES783323
sb_gmnlmfta_0004d12.t7	1		Unassigned protein				ES783894
sb_gmnlmfta_0004d13.t7	1		Cluster: Sarcoendoplasmic reticulum calcium ATPase; n=1; Silurus lanzhouensis|Rep: Sarcoendoplasmic reticulum calcium ATPase - Silurus lanzhouensis	GO:0003824 GO:0005388 GO:0005524 GO:0006810 GO:0006812 GO:0006816 GO:0008152 GO:0015662 GO:0015992 GO:0016020 GO:0016021 GO:0016820	1e-07	87% (21/24)	ES783888
sb_gmnlmfta_0004d16.t7	1		unclassified				ES783993
sb_gmnlmfta_0004d22.t7	1		unclassified				ES783358
sb_gmnlmfta_0004d24.t7	1		eef2l; eukaryotic translation elongation factor 2, like [EC:3.6.5.3]; K03234 elongation factor EF-2		4e-47	85% (87/102)	ES783478
sb_gmnlmfta_0004e04.t7	1		IFI35; interferon-induced protein 35		1e-11	39% (39/99)	ES783952
sb_gmnlmfta_0004e06.t7	1		Cluster: Parvalbumin; n=1; Theragra chalcogramma|Rep: Parvalbumin - Theragra chalcogramma (Alaska pollock)	GO:0005509	3e-32	81% (75/92)	ES784054
sb_gmnlmfta_0004e10.t7	1		Cluster: Aldolase a, fructose-bisphosphate, b; n=7; Clupeocephala|Rep: Aldolase a, fructose-bisphosphate, b - Brachydanio rerio (Zebrafish) (Danio rerio)	GO:0003824 GO:0004332 GO:0006096	2e-34	88% (68/77)	ES783663

				GO:0008152			
sb_gmnlmfta_0004e11.t7	1		unclassified				ES783712
sb_gmnlmfta_0004e20.t7	1		LOC506727; similar to nuclear factor of kappa light polypeptide gene enhancer in B-cells inhibitor, epsilon; K05872 nuclear factor of kappa light polypeptide gene enhancer in B-cells inhibitor, epsilon		5e-08	44% (37/84)	ES784217
sb_gmnlmfta_0004e23.t7	1		Cluster: Homolog of Brachydanio rerio "Similar to UPF3 regulator of nonsense transcripts homolog B.; n=1; Takifugu rubripes|Rep: Homolog of Brachydanio rerio "Similar to UPF3 regulator of nonsense transcripts homolog B. - Takifugu rubripes		7e-50	66% (103/154)	ES784297
sb_gmnlmfta_0004f01.t7	1		unclassified				ES783438
sb_gmnlmfta_0004f04.t7	1		unclassified				ES783341
sb_gmnlmfta_0004f07.t7	1		Unassigned protein				ES783264
sb_gmnlmfta_0004f14.t7	1		unclassified				ES784227
sb_gmnlmfta_0004f16.t7	1		unclassified				ES784332
sb_gmnlmfta_0004f17.t7	1		Cluster: RING finger protein 11; n=18; Euteleostomi|Rep: RING finger protein 11 - Homo sapiens (Human)		1e-22	68% (57/83)	ES784271
sb_gmnlmfta_0004f19.t7	1		unclassified				ES784005
sb_gmnlmfta_0004f21.t7	1		Cluster: Probable methylmalonate-semialdehyde dehydrogenase [acylating], mitochondrial precursor; n=3; Culicidae|Rep: Probable	GO:0004491 GO:0005739	2e-25	77%	ES783636

			methylmalonate-semialdehyde dehydrogenase [acylating], mitochondrial precursor - Anopheles gambiae (African malaria mosquito)	GO:0006573 GO:0008152 GO:0016491 GO:0018478		(37/48)	
sb_gmnlmfta_0004f22.t7	1		unclassified				ES783557
sb_gmnlmfta_0004g03.t7	1		Cluster: Elongation factor 1-gamma; n=7; Clupeocephala|Rep: Elongation factor 1-gamma - Brachydanio rerio (Zebrafish) (Danio rerio)		4e-18	48% (53/109)	ES784166
sb_gmnlmfta_0004g04.t7	1		Cluster: PREDICTED: similar to SSeCKS; n=2; Danio rerio|Rep: PREDICTED: similar to SSeCKS - Danio rerio		2e-15	31% (62/196)	ES784290
sb_gmnlmfta_0004g05.t7	1		Cluster: Myosin heavy chain; n=3; Gadidae|Rep: Myosin heavy chain - Theragra chalcogramma (Alaska pollock)	GO:0003774 GO:0005524 GO:0016459	6e-44	91% (85/93)	ES784311
sb_gmnlmfta_0004g06.t7	1		Cluster: ribosomal protein L27; n=2; Eutheria|Rep: ribosomal protein L27 - Canis familiaris		3e-18	97% (44/45)	ES784261
sb_gmnlmfta_0004g07.t7	1		unclassified				ES784206
sb_gmnlmfta_0004g09.t7	1		Cluster: PREDICTED: similar to Ubiquitin carboxyl-terminal hydrolase 47 (Ubiquitin thioesterase 47) (Ubiquitin-specific-processing protease 47) (Deubiquitinating enzyme 47), partial; n=3; Danio rerio|Rep: PREDICTED: similar to Ubiquitin carboxyl-terminal hydrolase 47 (Ubiquitin thioesterase 47) (Ubiquitin-specific-processing protease 47) (Deubiquitinating enzyme 47), partial - Danio rerio		2e-82	82% (149/181)	ES783936
sb_gmnlmfta_0004g14.t7	1		Cluster: Actin; n=10; Coelomata|Rep: Actin - Chasmagnathus granulata	GO:0005515	3e-09	80% (29/36)	ES783285
sb_gmnlmfta_0004h01.t7	1		unclassified				ES783273
sb_gmnlmfta_0004h06.t7	1		unclassified				ES783396

sb_gmnlmfta_0004h07.t7	1		unclassified				ES783447
sb_gmnlmfta_0004h13.t7	1		unclassified				ES784236
sb_gmnlmfta_0004h15.t7	1		unclassified				ES784090
sb_gmnlmfta_0004h21.t7	1		Cluster: Transaldolase; n=1; Ctenopharyngodon idella|Rep: Transaldolase - Ctenopharyngodon idella (Grass carp)	GO:0003824 GO:0004801 GO:0005737 GO:0005975 GO:0006098 GO:0008152	2e-45	73% (54/73)	ES783740
sb_gmnlmfta_0004h22.t7	1		DUF757 domain containing protein		4e-54	58% (79/134)	ES783681
sb_gmnlmfta_0004i04.t7	1		Cluster: PREDICTED: similar to ADP-ribosylation-like factor 6 interacting protein 6; n=1; Monodelphis domestica|Rep: PREDICTED: similar to ADP-ribosylation-like factor 6 interacting protein 6 - Monodelphis domestica		2e-06	44% (25/56)	ES784179
sb_gmnlmfta_0004i07.t7	1		Cluster: Eukaryotic translation initiation factor 3 subunit 7; n=19; Euteleostomi|Rep: Eukaryotic translation initiation factor 3 subunit 7 - Homo sapiens (Human)		9e-16	75% (40/53)	ES784120
sb_gmnlmfta_0004i09.t7	1		Cluster: Heat shock protein 4, like; n=3; Clupeocephala|Rep: Heat shock protein 4, like - Brachydanio rerio (Zebrafish) (Danio rerio)		4e-65	81% (119/146)	ES783852
sb_gmnlmfta_0004i10.t7	1		Cluster: Adenosine monophosphate deaminase; n=2; Platichthys flesus|Rep: Adenosine monophosphate deaminase - Platichthys flesus (European flounder)	GO:0003876 GO:0009168 GO:0019239	3e-42	79% (83/105)	ES783376
sb_gmnlmfta_0004i15.t7	1		unclassified				ES783414

sb_gmnlmfta_0004i16.t7	1		unclassified				ES783459
sb_gmnlmfta_0004i20.t7	1		Cluster: Actin, alpha 1, skeletal muscle; n=1; Homo sapiens|Rep: Actin, alpha 1, skeletal muscle - Homo sapiens (Human)		5e-34	97% (69/71)	ES783914
sb_gmnlmfta_0004i21.t7	1		unclassified				ES783874
sb_gmnlmfta_0004j06.t7	1		Cluster: Phosphoglucomutase 1; n=2; Danio rerio|Rep: Phosphoglucomutase 1 - Brachydanio rerio (Zebrafish) (Danio rerio)		4e-60	80% (92/115)	ES783729
sb_gmnlmfta_0004j09.t7	1		unclassified				ES783516
sb_gmnlmfta_0004j10.t7	1		unclassified				ES783986
sb_gmnlmfta_0004j12.t7	1		unclassified				ES784030
sb_gmnlmfta_0004j14.t7	1		unclassified				ES783900
sb_gmnlmfta_0004j15.t7	1		Cluster: Cytosolic malate dehydrogenase B; n=1; Oryzias latipes|Rep: Cytosolic malate dehydrogenase B - Oryzias latipes (Medaka fish) (Japanese ricefish)	GO:0004459<br>GO:0006096<br>GO:0006100<br>GO:0006108<br>GO:0016491<br>GO:0016615<br>GO:0030060<br>GO:0003824<br>GO:0005488<br>GO:0005975<br>GO:0006099<br>GO:0008152<br>GO:0016616	3e-67	88% (123/139)	ES783880
sb_gmnlmfta_0004j18.t7	1		Cluster: Adenosine monophosphate deaminase; n=2; Platichthys flesus|Rep: Adenosine monophosphate deaminase - Platichthys	GO:0003876<br>GO:0009168	1e-38	94% (72/76)	ES784073

			flesus (European flounder)	GO:0019239			
sb_gmnlmfta_0004j22.t7	1		unclassified				ES783487
sb_gmnlmfta_0004k01.t7	1		Cluster: Erythrocyte carbonic anhydrase; n=1; Oncorhynchus mykiss|Rep: Erythrocyte carbonic anhydrase - Oncorhynchus mykiss (Rainbow trout) (Salmo gairdneri)	GO:0004089 GO:0006730 GO:0008270 GO:0016829	3e-59	85% (107/125)	ES784019
sb_gmnlmfta_0004k03.t7	1		unclassified				ES783969
sb_gmnlmfta_0004k05.t7	1		unclassified				ES783809
sb_gmnlmfta_0004k07.t7	1		Cluster: Thioredoxin interacting protein; n=1; Danio rerio|Rep: Thioredoxin interacting protein - Brachydanio rerio (Zebrafish) (Danio rerio)		1e-105	71% (175/244)	ES783922
sb_gmnlmfta_0004k09.t7	1		Cluster: Ubiquitin-activating enzyme E1; n=4; Euteleostomi|Rep: Ubiquitin-activating enzyme E1 - Brachydanio rerio (Zebrafish) (Danio rerio)		1e-106	76% (168/220)	ES784194
sb_gmnlmfta_0004k14.t7	1		Unassigned protein				ES783761
sb_gmnlmfta_0004k21.t7	1		unclassified				ES784080
sb_gmnlmfta_0004l14.t7	1		unclassified				ES783721
sb_gmnlmfta_0004l17.t7	1		unclassified				ES783763
sb_gmnlmfta_0004l19.t7	1		unclassified				ES783506

sb_gmnlmfta_0004l21.t7	1		unclassified				ES784160
sb_gmnlmfta_0004m02.t7	1		Cluster: PREDICTED: similar to alpha 3-actin; n=1; Monodelphis domestica|Rep: PREDICTED: similar to alpha 3-actin - Monodelphis domestica		8e-10	93% (31/33)	ES783599
sb_gmnlmfta_0004m05.t7	1		Cluster: Putative fast skeletal muscle troponin; n=1; Paralichthys olivaceus|Rep: Putative fast skeletal muscle troponin - Paralichthys olivaceus (Japanese flounder)		4e-17	89% (44/49)	ES783724
sb_gmnlmfta_0004m08.t7	1		Cluster: Sarcoplasmic/endoplasmic reticulum calcium ATPase 1 (EC 3.6.3.8) (Calcium pump 1) (SERCA1) (SR Ca(2+)-ATPase 1) (Calcium-transporting ATPase sarcoplasmic reticulum type, fast twitch skeletal muscle isoform) (Endoplasmic reticulum class 1/2 Ca(2+) ATPase); n=6; Holacanthopterygii|Rep: Sarcoplasmic/endoplasmic reticulum calcium ATPase 1 (EC 3.6.3.8) (Calcium pump 1) (SERCA1) (SR Ca(2+)-ATPase 1) (Calcium-transporting ATPase sarcoplasmic reticulum type, fast twitch skeletal muscle isoform) (Endoplasmic reticulum class 1/2 Ca(2+) ATPase) - Makaira nigricans (Blue marlin)	GO:0000166 GO:0000287 GO:0003824 GO:0005388 GO:0005509 GO:0005515 GO:0005524 GO:0005783 GO:0006810 GO:0006811 GO:0006812 GO:0006816 GO:0008152 GO:0015662 GO:0016020 GO:0016021 GO:0016529 GO:0016787 GO:0016820 GO:0031448 GO:0046872	1e-27	85% (63/74)	ES783411
sb_gmnlmfta_0004m09.t7	1		unclassified				ES783435
sb_gmnlmfta_0004m13.t7	1		unclassified				ES783991
sb_gmnlmfta_0004m18.t7	1		unclassified				ES784148
sb_gmnlmfta_0004m20.t7	1		unclassified				ES783497

sb_gmnlmfta_0004m22.t7	1		unclassified				ES783399
sb_gmnlmfta_0004n03.t7	1		unclassified				ES784256
sb_gmnlmfta_0004n04.t7	1		Cluster: Kir6.2 protein; n=1; Danio rerio|Rep: Kir6.2 protein - Brachydanio rerio (Zebrafish) (Danio rerio)		2e-98	78% (178/226)	ES784125
sb_gmnlmfta_0004n08.t7	1		Cluster: Transcribed locus, weakly similar to NP_004137.2 NADH dehydrogenase (ubiquinone) 1 beta subcomplex, 7, 18kDa [Homo sapiens]; n=1; Takifugu rubripes|Rep: Transcribed locus, weakly similar to NP_004137.2 NADH dehydrogenase (ubiquinone) 1 beta subcomplex, 7, 18kDa [Homo sapiens] - Takifugu rubripes		1e-43	77% (73/94)	ES783879
sb_gmnlmfta_0004n10.t7	1		Cluster: Protein-tyrosine phosphatase-like member A; n=9; Eutheria|Rep: Protein-tyrosine phosphatase-like member A - Homo sapiens (Human)		1e-19	88% (44/50)	ES783298
sb_gmnlmfta_0004n18.t7	1		Cluster: Triosephosphate isomerase 1b; n=3; Danio rerio|Rep: Triosephosphate isomerase 1b - Brachydanio rerio (Zebrafish) (Danio rerio)		7e-43	91% (84/92)	ES783667
sb_gmnlmfta_0004n20.t7	1		Cluster: 60S acidic ribosomal protein P0; n=16; Theria|Rep: 60S acidic ribosomal protein P0 - Homo sapiens (Human)		3e-28	79% (59/74)	ES783824
sb_gmnlmfta_0004n22.t7	1		Cluster: Splicing factor 3B subunit 5; n=9; Euteleostomi|Rep: Splicing factor 3B subunit 5 - Homo sapiens (Human)		3e-34	92% (64/69)	ES783871
sb_gmnlmfta_0004o01.t7	1		unclassified				ES783347
sb_gmnlmfta_0004o03.t7	1		unclassified				ES783308
sb_gmnlmfta_0004o05.t7	1		Cluster: MGC69505 protein; n=2; Xenopus|Rep: MGC69505 protein - Xenopus tropicalis (Western clawed frog) (Silurana tropicalis)	GO:0004450 GO:0008152 GO:0016616	2e-12	84% (32/38)	ES783391

sb_gmnlmfta_0004o06.t7	1		Cluster: 40S ribosomal protein S3a; n=47; Eukaryota|Rep: 40S ribosomal protein S3a - Homo sapiens (Human)		1e-118	90% (210/232)	ES783472
sb_gmnlmfta_0004o07.t7	1		unclassified				ES783508
sb_gmnlmfta_0004o22.t7	1		unclassified				ES783735
sb_gmnlmfta_0004o24.t7	1		unclassified				ES783650
sb_gmnlmfta_0004p02.t7	1		unclassified				ES784234
sb_gmnlmfta_0004p03.t7	1		Cluster: Voltage-dependent anion channel 2; n=5; Clupeocephala|Rep: Voltage-dependent anion channel 2 - Brachydanio rerio (Zebrafish) (Danio rerio)		4e-38	90% (77/85)	ES784223
sb_gmnlmfta_0004p07.t7	1		Cluster: Ubiquitin specific protease 14; n=2; Danio rerio|Rep: Ubiquitin specific protease 14 - Brachydanio rerio (Zebrafish) (Danio rerio)		3e-39	71% (61/85)	ES784142
sb_gmnlmfta_0004p08.t7	1		serinc1; serine incorporator 1	GO:0016020	1e-26	70% (56/79)	ES783908
sb_gmnlmfta_0004p10.t7	1		Cluster: Pyrophosphatase (Inorganic) 1; n=2; Danio rerio|Rep: Pyrophosphatase (Inorganic) 1 - Brachydanio rerio (Zebrafish) (Danio rerio)		1e-67	74% (122/163)	ES783278
sb_gmnlmfta_0004p12.t7	1		Cluster: LIM domain binding 3 like; n=2; Danio rerio|Rep: LIM domain binding 3 like - Brachydanio rerio (Zebrafish) (Danio rerio)		5e-69	81% (126/154)	ES783356
sb_gmnlmfta_0004p14.t7	1		unclassified				ES783468

sb_gmnlmfta_0004p15.t7	1		C1_1 domain containing protein		2e-09	34% (16/46)	ES783489
sb_gmnlmfta_0004p21.t7	1		Cluster: Phosphoglucomutase 1; n=2; Danio rerio|Rep: Phosphoglucomutase 1 - Brachydanio rerio (Zebrafish) (Danio rerio)		2e-42	83% (61/73)	ES783840
sb_gmnlmfta_0005a01.t7	1		Cluster: Ubiquitin fusion degradation 1-like protein; n=1; Danio rerio|Rep: Ubiquitin fusion degradation 1-like protein - Danio rerio (Zebrafish) (Brachydanio rerio)		3e-10	68% (32/47)	FL634582
sb_gmnlmfta_0005a02.t7	1		Cluster: Creatine kinase muscle isoform 2; n=5; Percomorpha|Rep: Creatine kinase muscle isoform 2 - Chaenocephalus aceratus (White crocodile fish)	GO:0003824 GO:0016301 GO:0016740 GO:0016772	2e-24	70% (61/86)	FL634583
sb_gmnlmfta_0005a05.t7	1		Unassigned protein				FL634586
sb_gmnlmfta_0005a07.t7	1		Unassigned protein				FL634587
sb_gmnlmfta_0005a08.t7	1		Cluster: UNC45-related protein; n=2; Danio rerio|Rep: UNC45-related protein - Danio rerio (Zebrafish) (Brachydanio rerio)		4e-71	84% (130/154)	FL634588
sb_gmnlmfta_0005a20.t7	1		Unassigned protein				FL634595
sb_gmnlmfta_0005b01.t7	1		Cluster: PREDICTED: similar to elongation factor SIII p15 subunit; n=2; Eutheria|Rep: PREDICTED: similar to elongation factor SIII p15 subunit - Equus caballus		9e-45	86% (69/80)	FL634600
sb_gmnlmfta_0005b08.t7	1		Cluster: Polymerase delta interacting protein 38; n=3; Danio rerio|Rep: Polymerase delta interacting protein 38 - Danio rerio (Zebrafish) (Brachydanio rerio)		6e-68	73% (129/176)	FL634603
sb_gmnlmfta_0005b12.t7	1		Unassigned protein				FL634607

sb_gmnlmfta_0005b14.t7	1		Cluster: PREDICTED: similar to calcium-transporting ATPase; n=1; Monodelphis domestica|Rep: PREDICTED: similar to calcium-transporting ATPase - Monodelphis domestica		2e-05	100% (24/24)	FL634609
sb_gmnlmfta_0005b17.t7	1		Unassigned protein				FL634610
sb_gmnlmfta_0005b18.t7	1		Unassigned protein				FL634611
sb_gmnlmfta_0005b23.t7	1		Cluster: Histone methyltransferase SmyD1a; n=3; Danio rerio|Rep: Histone methyltransferase SmyD1a - Danio rerio (Zebrafish) (Brachydanio rerio)		2e-59	65% (105/160)	FL634615
sb_gmnlmfta_0005c05.t7	1		Cluster: Nars-prov protein; n=2; Xenopus|Rep: Nars-prov protein - Xenopus laevis (African clawed frog)	GO:0000166 GO:0003676 GO:0004812 GO:0004815 GO:0004816 GO:0005524 GO:0005737 GO:0006412 GO:0006418 GO:0006421 GO:0006422 GO:0008703 GO:0009231 GO:0016874 GO:0050661	4e-55	90% (100/110)	FL634617
sb_gmnlmfta_0005c07.t7	1		Unassigned protein				FL634618
sb_gmnlmfta_0005c08.t7	1		Cluster: Myosin light chain 1; n=1; Theragra chalcogramma|Rep: Myosin light chain 1 - Theragra chalcogramma (Alaska pollock)	GO:0005509	2e-20	54% (66/122)	FL634619
sb_gmnlmfta_0005c10.t7	1		Unassigned protein				FL634621
sb_gmnlmfta_0005c11.t7	1		Cluster: PREDICTED: similar to rat ribosomal protein L9 homologue isoform 1; n=2; Catarrhini|Rep: PREDICTED: similar to rat ribosomal protein L9 homologue isoform 1 - Pan troglodytes		9e-42	88% (80/90)	FL634622

sb_gmnlmfta_0005c20.t7	1		Cluster: Ribosomal protein S2; n=4; Euteleostomi|Rep: Ribosomal protein S2 - Danio rerio (Zebrafish) (Brachydanio rerio)		2e-30	100% (42/42)	FL634630
sb_gmnlmfta_0005c23.t7	1		Keratin_B2 domain containing protein		7e-06	30% (27/89)	FL634633
sb_gmnlmfta_0005d06.t7	1		Cluster: Fast skeletal muscle troponin I; n=1; Gadus morhua|Rep: Fast skeletal muscle troponin I - Gadus morhua (Atlantic cod)		3e-26	92% (58/63)	FL634639
sb_gmnlmfta_0005d13.t7	1		Unassigned protein				FL634645
sb_gmnlmfta_0005d14.t7	1		Unassigned protein				FL634646
sb_gmnlmfta_0005d16.t7	1		Unassigned protein				FL634648
sb_gmnlmfta_0005d18.t7	1		Unassigned protein				FL634650
sb_gmnlmfta_0005d19.t7	1		Cluster: 40S ribosomal protein S9; n=26; Gnathostomata|Rep: 40S ribosomal protein S9 - Homo sapiens (Human)		2e-96	97% (176/180)	FL634651
sb_gmnlmfta_0005d22.t7	1		Cluster: PREDICTED: similar to alpha 3-actin; n=1; Monodelphis domestica|Rep: PREDICTED: similar to alpha 3-actin - Monodelphis domestica	GO:0000166 GO:0003774 GO:0005524	7e-10	100% (31/31)	FL634654
sb_gmnlmfta_0005e01.t7	1		Cluster: Parvalbumin beta; n=4; Gadiformes|Rep: Parvalbumin beta - Merluccius bilinearis (Silver hake)	GO:0005509	9e-15	70% (38/54)	FL634657
sb_gmnlmfta_0005e02.t7	1		Unassigned protein				FL634658

sb_gmnlmfta_0005e04.t7	1		Unassigned protein				FL634660
sb_gmnlmfta_0005e07.t7	1		ADK domain containing protein		8e-06	60% (12/20)	FL634663
sb_gmnlmfta_0005e08.t7	1		Unassigned protein				FL634664
sb_gmnlmfta_0005e09.t7	1		Cluster: Peptidyl-prolyl cis-trans isomerase; n=1; Hemicentrotus pulcherrimus|Rep: Peptidyl-prolyl cis-trans isomerase - Hemicentrotus pulcherrimus (Sea urchin)	GO:0003755 GO:0005737 GO:0006457 GO:0016853 GO:0042277	5e-35	61% (74/121)	FL634665
sb_gmnlmfta_0005e11.t7	1		LSU rRNA; Neoceratodus forsteri		2e-27	91% (106/116)	FL634666
sb_gmnlmfta_0005e13.t7	1		Cluster: Homolog of Brachydanio rerio "ATP synthase, H+ transporting, mitochondrial F0 complex, subunit d.; n=1; Takifugu rubripes|Rep: Homolog of Brachydanio rerio "ATP synthase, H+ transporting, mitochondrial F0 complex, subunit d. - Takifugu rubripes		6e-22	64% (34/53)	FL634668
sb_gmnlmfta_0005e15.t7	1		Unassigned protein				FL634670
sb_gmnlmfta_0005e17.t7	1		Unassigned protein				FL634671
sb_gmnlmfta_0005e18.t7	1		Cluster: LOC564613 protein; n=2; Danio rerio|Rep: LOC564613 protein - Danio rerio (Zebrafish) (Brachydanio rerio)		2e-44	90% (83/92)	FL634672
sb_gmnlmfta_0005e19.t7	1		Cluster: Ubiquitin/40S ribosomal protein S27a fusion protein; n=16; Eukaryota|Rep: Ubiquitin/40S ribosomal protein S27a fusion protein - Argas monolakensis	GO:0003735 GO:0005622 GO:0005840 GO:0006412 GO:0006464	2e-30	97% (66/68)	FL634673

sb_gmnlmfta_0005e20.t7	1		unclassified				FL634674
sb_gmnlmfta_0005f01.t7	1		Unassigned protein				FL634678
sb_gmnlmfta_0005f03.t7	1		Unassigned protein				FL634680
sb_gmnlmfta_0005f04.t7	1		Cluster: Creatine kinase; n=1; Gillichthys mirabilis|Rep: Creatine kinase - Gillichthys mirabilis (Long-jawed mudsucker)	GO:0003824 GO:0016301 GO:0016740 GO:0016772	2e-15	83% (31/37)	FL634681
sb_gmnlmfta_0005f07.t7	1		Unassigned protein				FL634682
sb_gmnlmfta_0005f08.t7	1		Unassigned protein				FL634683
sb_gmnlmfta_0005f09.t7	1		selenoprotein S [Homo sapiens] ref|NP_982298.1| selenoprotein S [Homo sapiens] gb|AAP85541.1| selenoprotein S [Homo sapiens] gb|AAH05840.2| Selenoprotein S [Homo sapiens] gb|AAI07775.1| Selenoprotein S [Homo sapiens]		2e-16	42% (53/124)	FL634684
sb_gmnlmfta_0005f12.t7	1		Unassigned protein				FL634687
sb_gmnlmfta_0005f13.t7	1		Cluster: Leukemia inhibitory factor receptor; n=1; Carassius auratus|Rep: Leukemia inhibitory factor receptor - Carassius auratus (Goldfish)	GO:0004872	5e-13	26% (54/206)	FL634688
sb_gmnlmfta_0005f14.t7	1		Unassigned protein				FL634689
sb_gmnlmfta_0005f17.t7	1		Unassigned protein				FL634691

sb_gmnlmfta_0005f18.t7	1		F420_oxidored domain containing protein		1e-24	31% (50/158)	FL634692
sb_gmnlmfta_0005f20.t7	1		Unassigned protein				FL634693
sb_gmnlmfta_0005f23.t7	1		Unassigned protein				FL634696
sb_gmnlmfta_0005g05.t7	1		Cluster: Actin; n=1; Spirula spirula|Rep: Actin - Spirula spirula	GO:0005515 GO:0000166 GO:0005198 GO:0005524	6e-11	86% (25/29)	FL634699
sb_gmnlmfta_0005g10.t7	1		Unassigned protein				FL634702
sb_gmnlmfta_0005g15.t7	1		Unassigned protein				FL634706
sb_gmnlmfta_0005g18.t7	1		Unassigned protein				FL634709
sb_gmnlmfta_0005h01.t7	1		Cluster: Myosin light chain 2; n=2; Holacanthopterygii|Rep: Myosin light chain 2 - Theragra chalcogramma (Alaska pollock)	GO:0005509	2e-19	67% (53/78)	FL634716
sb_gmnlmfta_0005h02.t7	1		Cluster: Troponin C, slow skeletal and cardiac muscles; n=13; Amniota|Rep: Troponin C, slow skeletal and cardiac muscles - Homo sapiens (Human)		1e-05	100% (22/22)	FL634717
sb_gmnlmfta_0005h05.t7	1		Unassigned protein				FL634719
sb_gmnlmfta_0005h13.t7	1		unclassified				FL634727

sb_gmnlmfta_0005h15.t7	1		Unassigned protein				FL634729
sb_gmnlmfta_0005h17.t7	1		Unassigned protein				FL634731
sb_gmnlmfta_0005h18.t7	1		Unassigned protein				FL634732
sb_gmnlmfta_0005h19.t7	1		Unassigned protein				FL634733
sb_gmnlmfta_0005h20.t7	1		Cluster: ATP synthase delta chain; n=2; Tetraodontidae|Rep: ATP synthase delta chain - Tetraodon nigroviridis (Green puffer)	GO:0006754<br>GO:0006810<br>GO:0006811<br>GO:0015986<br>GO:0016469<br>GO:0016787<br>GO:0045261<br>GO:0046872<br>GO:0046933<br>GO:0046961	3e-70	75% (133/176)	FL634734
sb_gmnlmfta_0005h21.t7	1		Unassigned protein				FL634735
sb_gmnlmfta_0005h22.t7	1		Cluster: Proteasome (Prosome, macropain) 26S subunit, non-ATPase, 3; n=3; Danio rerio|Rep: Proteasome (Prosome, macropain) 26S subunit, non-ATPase, 3 - Danio rerio (Zebrafish) (Brachydanio rerio)		5e-63	91% (120/131)	FL634736
sb_gmnlmfta_0005h24.t7	1		I-set multi-domain protein		5e-15	25% (21/82)	FL634738
sb_gmnlmfta_0005i05.t7	1		Cluster: 40S ribosomal protein S29; n=33; Euteleostomi|Rep: 40S ribosomal protein S29 - Homo sapiens (Human)		2e-17	92% (39/42)	FL634741
sb_gmnlmfta_0005i06.t7	1		Unassigned protein				FL634742

sb_gmnlmfta_0005i12.t7	1		Unassigned protein				FL634747
sb_gmnlmfta_0005i15.t7	1		Unassigned protein				FL634749
sb_gmnlmfta_0005i16.t7	1		Cluster: Peptidyl-prolyl cis-trans isomerase; n=1; Chlamys farreri|Rep: Peptidyl-prolyl cis-trans isomerase - Chlamys farreri	GO:0003755 GO:0006457 GO:0016853	3e-32	78% (64/82)	FL634750
sb_gmnlmfta_0005i17.t7	1		Unassigned protein				FL634751
sb_gmnlmfta_0005i20.t7	1		Cluster: PREDICTED: similar to rapamycin insensitive companion of mTOR; rictor; n=1; Ornithorhynchus anatinus|Rep: PREDICTED: similar to rapamycin insensitive companion of mTOR; rictor - Ornithorhynchus anatinus		1e-101	80% (169/211)	FL634754
sb_gmnlmfta_0005j02.t7	1		Unassigned protein				FL634760
sb_gmnlmfta_0005j07.t7	1		Cluster: PREDICTED: similar to Ribosomal protein S2; n=1; Ornithorhynchus anatinus|Rep: PREDICTED: similar to Ribosomal protein S2 - Ornithorhynchus anatinus		3e-33	79% (73/92)	FL634763
sb_gmnlmfta_0005j15.t7	1		Cluster: PREDICTED: similar to lung cancer oncogene 7 isoform 1; n=1; Pan troglodytes|Rep: PREDICTED: similar to lung cancer oncogene 7 isoform 1 - Pan troglodytes		5e-34	97% (65/67)	FL634771
sb_gmnlmfta_0005j16.t7	1		Unassigned protein				FL634772
sb_gmnlmfta_0005j18.t7	1		Unassigned protein				FL634774
sb_gmnlmfta_0005j23.t7	1		Cluster: Homolog of Homo sapiens "TiTin isoform novex-2; n=1; Takifugu rubripes|Rep: Homolog of Homo sapiens "TiTin isoform novex-2 - Takifugu rubripes		2e-77	81% (128/157)	FL634779

sb_gmnlmfta_0005k01.t7	1		Unassigned protein				FL634781
sb_gmnlmfta_0005k02.t7	1		Unassigned protein				FL634782
sb_gmnlmfta_0005k09.t7	1		Cluster: PREDICTED: similar to Actin, alpha skeletal muscle (Alpha-actin 1) isoform 3; n=1; Canis lupus familiaris|Rep: PREDICTED: similar to Actin, alpha skeletal muscle (Alpha-actin 1) isoform 3 - Canis familiaris	GO:0000166 GO:0005198 GO:0005515 GO:0005524 GO:0005737	2e-24	84% (59/70)	FL634788
sb_gmnlmfta_0005k12.t7	1		Cluster: GTP-binding nuclear protein Ran; n=18; Tetrapoda|Rep: GTP-binding nuclear protein Ran - Homo sapiens (Human)	GO:0000166 GO:0003924 GO:0005515 GO:0005525 GO:0005634 GO:0006810 GO:0006886 GO:0006913 GO:0007165 GO:0007264	1e-72	91% (133/146)	FL634791
sb_gmnlmfta_0005k14.t7	1		Unassigned protein				FL634793
sb_gmnlmfta_0005k17.t7	1		Cluster: Actin, muscle; n=4; Branchiostoma|Rep: Actin, muscle - Branchiostoma lanceolatum (Common lancelet) (Amphioxus)	GO:0000166 GO:0005198 GO:0005515 GO:0005524 GO:0005737 GO:0005856	1e-09	61% (33/54)	FL634796
sb_gmnlmfta_0005k19.t7	1		Unassigned protein				FL634797
sb_gmnlmfta_0005k21.t7	1		Cluster: Actin, alpha 1, skeletal muscle; n=1; Homo sapiens|Rep: Actin, alpha 1, skeletal muscle - Homo sapiens (Human)		8e-29	95% (43/45)	FL634798
sb_gmnlmfta_0005k22.t7	1		Skp1 multi-domain protein		1e-32	79% (55/69)	FL634799

sb_gmnlmfta_0005k24.t7	1		Cluster: Annexin A4; n=3; Xenopus|Rep: Annexin A4 - Xenopus tropicalis (Western clawed frog) (Silurana tropicalis)	GO:0005509 GO:0005544	2e-15	61% (29/47)	FL634801
sb_gmnlmfta_0005l02.t7	1		Cluster: PREDICTED: similar to Ubiquitin carboxyl-terminal hydrolase 47 (Ubiquitin thioesterase 47) (Ubiquitin-specific-processing protease 47) (Deubiquitinating enzyme 47); n=1; Ornithorhynchus anatinus|Rep: PREDICTED: similar to Ubiquitin carboxyl-terminal hydrolase 47 (Ubiquitin thioesterase 47) (Ubiquitin-specific-processing protease 47) (Deubiquitinating enzyme 47) - Ornithorhynchus anatinus		9e-22	91% (44/48)	FL634803
sb_gmnlmfta_0005l06.t7	1		Unassigned protein				FL634806
sb_gmnlmfta_0005l08.t7	1		DUF1077 domain containing protein		3e-17	46% (35/75)	FL634808
sb_gmnlmfta_0005l09.t7	1		Cluster: Proliferating cell nuclear antigen; n=5; Elopocephala|Rep: Proliferating cell nuclear antigen - Anguilla japonica (Japanese eel)	GO:0003677 GO:0005634 GO:0006260 GO:0006275 GO:0030337 GO:0043626	3e-64	94% (125/132)	FL634809
sb_gmnlmfta_0005l15.t7	1		Cluster: Microsomal glutathione S-transferase; n=1; Platichthys flesus|Rep: Microsomal glutathione S-transferase - Platichthys flesus (European flounder)	GO:0016740	1e-21	70% (51/72)	FL634812
sb_gmnlmfta_0005l20.t7	1		UPF0041 domain containing protein		3e-29	30% (32/105)	FL634815
sb_gmnlmfta_0005l24.t7	1		LOC711021; similar to Protein C6orf142 homolog		7e-05	41% (26/63)	FL634818
sb_gmnlmfta_0005m02.t7	1		Cluster: Ubiquitin carrier protein; n=2; Clupeocephala|Rep: Ubiquitin carrier protein - Danio rerio (Zebrafish) (Brachydanio rerio)		9e-39	71% (77/108)	FL634820
sb_gmnlmfta_0005m04.t7	1		Unassigned protein				FL634821

sb_gmnlmfta_0005m08.t7	1		Unassigned protein				FL634824
sb_gmnlmfta_0005m13.t7	1		Cluster: Actin, gamma 1; n=1; Pan troglodytes verus|Rep: Actin, gamma 1 - Pan troglodytes verus	GO:0000166 GO:0005198 GO:0005515 GO:0005524 GO:0005856	1e-51	91% (98/107)	FL634828
sb_gmnlmfta_0005m15.t7	1		Unassigned protein				FL634830
sb_gmnlmfta_0005m16.t7	1		Unassigned protein				FL634831
sb_gmnlmfta_0005m17.t7	1		BUD22 domain containing protein		1e-04	19% (45/228)	FL634832
sb_gmnlmfta_0005m18.t7	1		Unassigned protein				FL634833
sb_gmnlmfta_0005m20.t7	1		Unassigned protein				FL634835
sb_gmnlmfta_0005n03.t7	1		unclassified				FL634840
sb_gmnlmfta_0005n04.t7	1		Cluster: Heat shock cognate 70 kDa protein 1; n=3; core eudicotyledons|Rep: Heat shock cognate 70 kDa protein 1 - Solanum lycopersicum (Tomato) (Lycopersicon esculentum)	GO:0000166 GO:0005524 GO:0006950	2e-56	80% (71/88)	FL634841
sb_gmnlmfta_0005n07.t7	1		Cluster: Serine/threonine-protein phosphatase 2A catalytic subunit alpha isoform; n=63; Bilateria|Rep: Serine/threonine-protein phosphatase 2A catalytic subunit alpha isoform - Homo sapiens (Human)		1e-57	84% (106/125)	FL634844
sb_gmnlmfta_0005n11.t7	1		Cluster: Twinfilin-2; n=2; Danio rerio|Rep: Twinfilin-2 - Danio rerio (Zebrafish) (Brachydanio rerio)		4e-60	76% (113/148)	FL634847

sb_gmnlmfta_0005n20.t7	1		Cluster: Heat shock protein 60; n=1; Pseudosciaena crocea|Rep: Heat shock protein 60 - Pseudosciaena crocea (Croceine croaker)	GO:0005488 GO:0006950	3e-35	90% (70/77)	FL634855
sb_gmnlmfta_0005n24.t7	1		Cluster: Homolog of Gallus gallus "Calsequestrin, skeletal muscle isoform precursor (Calsequestrin 1) (Aspartactin) (Laminin-binding protein).; n=1; Takifugu rubripes|Rep: Homolog of Gallus gallus "Calsequestrin, skeletal muscle isoform precursor (Calsequestrin 1) (Aspartactin) (Laminin-binding protein). - Takifugu rubripes		1e-105	82% (172/209)	FL634858
sb_gmnlmfta_0005o01.t7	1		DUF1421 multi-domain protein		2e-05	23% (31/130)	FL634859
sb_gmnlmfta_0005o03.t7	1		Unassigned protein				FL634860
sb_gmnlmfta_0005o06.t7	1		Cluster: Signal recognition particle 54 kDa protein; n=20; Euteleostomi|Rep: Signal recognition particle 54 kDa protein - Homo sapiens (Human)		4e-93	94% (167/176)	FL634863
sb_gmnlmfta_0005o08.t7	1		Cluster: Ribosomal protein L7a; n=2; Eutheria|Rep: Ribosomal protein L7a - Equus caballus (Horse)	GO:0003735 GO:0005622 GO:0005840 GO:0006412 GO:0030529 GO:0042254	7e-34	92% (70/76)	FL634865
sb_gmnlmfta_0005o11.t7	1		Unassigned protein				FL634866
sb_gmnlmfta_0005o16.t7	1		ubiquitin C-terminal hydrolase [Ictalurus punctatus]		3e-70	92% (132/142)	FL634870
sb_gmnlmfta_0005o17.t7	1		Unassigned protein				FL634871
sb_gmnlmfta_0005o22.t7	1		Cluster: Actin, alpha 1, skeletal muscle; n=1; Homo sapiens|Rep: Actin, alpha 1, skeletal muscle - Homo sapiens (Human)		2e-15	82% (32/39)	FL634875

sb_gmnlmfta_0005p02.t7	1		Cluster: Signal sequence receptor, delta; n=2; Danio rerio|Rep: Signal sequence receptor, delta - Danio rerio (Zebrafish) (Brachydanio rerio)		6e-46	79% (91/114)	FL634877
sb_gmnlmfta_0005p06.t7	1		Cluster: Homolog of Homo sapiens "proline dehydrogenase (oxidase) 1; n=1; Takifugu rubripes|Rep: Homolog of Homo sapiens "proline dehydrogenase (oxidase) 1 - Takifugu rubripes		5e-11	83% (20/24)	FL634879
sb_gmnlmfta_0005p09.t7	1		Unassigned protein				FL634882
sb_gmnlmfta_0005p18.t7	1		Unassigned protein				FL634889
sb_gmnlmfta_0005p19.t7	1		Unassigned protein				FL634890

[1]Annotations presented in the supplemental table were generated with AutoFACT [18], while annotations presented in the manuscript are recent BLASTx hits that reflect a more updated state of the NCBI's nr protein database.
