# Supplementary material for: Heat-shock responsive genes identified and validated in Atlantic cod (Gadus morhua) liver, head kidney and skeletal muscle using genomic techniques
Source: BMC Genomics. 2010 Jan 28;11:72. doi: 10.1186/1471-2164-11-72 (PMC2830189; doi:10.1186/1471-2164-11-72)
Supplement: Additional file 2 — Supplemental Table S2, assembled ESTs (contigs and singletons) in the reverse heat-shock SSH libraries. Contains 2 tables (S2A and S2B) with information such as supporting annotations, statistics, and contributing EST accession numbers of contigs and singletons found in the 2 reverse libraries that were sequenced (head kidney and liver). [file 1471-2164-11-72-S2.PDF]

Supplemental Table S2A. Contigs in library gmnlkrta (reverse heat-shock head kidney SSH library) with supporting annotations[1], statistics, and contributing EST accession numbers

Sequence	Count	Sub-sequences	AutoFACT Description	GO terms	E-value	Identity	Accession Number
sb_gmnlkrta.0.C1	26	sb_gmnlkrta_0001d08.t7<br>sb_gmnlkrta_0001f10.t7<br>sb_gmnlkrta_0001b11.t7<br>sb_gmnlkrta_0001c02.t7<br>sb_gmnlkrta_0001h04.t7<br>sb_gmnlkrta_0001g06.t7<br>sb_gmnlkrta_0001a09.t7<br>sb_gmnlkrta_0001e12.t7<br>sb_gmnlkrta_0001c05.t7<br>sb_gmnlkrta_0001a10.t7<br>sb_gmnlkrta_0001d09.t7<br>sb_gmnlkrta_0001a12.t7<br>sb_gmnlkrta_0001b01.t7<br>sb_gmnlkrta_0001c03.t7<br>sb_gmnlkrta_0001d03.t7<br>sb_gmnlkrta_0001d05.t7<br>sb_gmnlkrta_0001d12.t7<br>sb_gmnlkrta_0001e09.t7<br>sb_gmnlkrta_0001f04.t7<br>sb_gmnlkrta_0001f07.t7<br>sb_gmnlkrta_0001f09.t7<br>sb_gmnlkrta_0001h02.t7<br>sb_gmnlkrta_0001a08.t7<br>sb_gmnlkrta_0001h08.t7<br>sb_gmnlkrta_0001a07.t7<br>sb_gmnlkrta_0001g07.t7	Cluster: Hemoglobin subunit alpha-1; n=3; Gadidae|Rep: Hemoglobin subunit alpha-1 - Gadus morhua (Atlantic cod)	GO:0005344<br>GO:0005506<br>GO:0005833<br>GO:0006810<br>GO:0015671<br>GO:0019825<br>GO:0020037	2e-53	91% (103/113)	ES780720<br>ES780748<br>ES780704<br>ES780706<br>ES780732<br>ES780761<br>ES780696<br>ES780703<br>ES780693<br>ES780753<br>ES780721<br>ES780747<br>ES780771<br>ES780707<br>ES780716<br>ES780697<br>ES780765<br>ES780758<br>ES780724<br>ES780730<br>ES780700<br>ES780722<br>ES780692<br>ES780715<br>ES780723<br>ES780762
sb_gmnlkrta.5.C1	8	sb_gmnlkrta_0001b10.t7<br>sb_gmnlkrta_0001e01.t7<br>sb_gmnlkrta_0001g02.t7<br>sb_gmnlkrta_0001a01.t7<br>sb_gmnlkrta_0001b04.t7<br>sb_gmnlkrta_0001b12.t7<br>sb_gmnlkrta_0001a06.t7<br>sb_gmnlkrta_0001f03.t7	Cluster: Hemoglobin subunit beta-1; n=2; Gadidae|Rep: Hemoglobin subunit beta-1 - Gadus morhua (Atlantic cod)	GO:0005344<br>GO:0005506<br>GO:0005833<br>GO:0006810<br>GO:0015671<br>GO:0019825<br>GO:0020037	3e-73	97% (133/137)	ES780708<br>ES780767<br>ES780745<br>ES780734<br>ES780777<br>ES780713<br>ES780718<br>ES780737
sb_gmnlkrta.3.C1	7	sb_gmnlkrta_0001b08.t7<br>sb_gmnlkrta_0001c07.t7<br>sb_gmnlkrta_0001c12.t7<br>sb_gmnlkrta_0001e11.t7<br>sb_gmnlkrta_0001f06.t7<br>sb_gmnlkrta_0001f08.t7<br>sb_gmnlkrta_0001h03.t7	Cluster: Hemoglobin subunit beta-2; n=3; Gadidae|Rep: Hemoglobin subunit beta-2 - Gadus morhua (Atlantic cod)	GO:0005344<br>GO:0005506<br>GO:0005833<br>GO:0006810<br>GO:0015671<br>GO:0019825<br>GO:0020037	4e-50	96% (100/104)	ES780760<br>ES780701<br>ES780773<br>ES780712<br>ES780726<br>ES780699<br>ES780719
sb_gmnlkrta.1.C1	4	sb_gmnlkrta_0001c04.t7<br>sb_gmnlkrta_0001h11.t7<br>sb_gmnlkrta_0001g10.t7<br>sb_gmnlkrta_0001d01.t7	Cluster: Hemoglobin subunit alpha-2; n=3; Gadidae|Rep: Hemoglobin subunit alpha-2 - Gadus morhua (Atlantic cod)	GO:0005344<br>GO:0005506<br>GO:0005833<br>GO:0006810<br>GO:0015671<br>GO:0019825	7e-36	98% (75/76)	ES780694<br>ES780757<br>ES780736<br>ES780705

				GO:0020037			
sb_gmnlkrta.2.C1	4	sb_gmnlkrta_0001c06.t7<br>sb_gmnlkrta_0001a04.t7<br>sb_gmnlkrta_0001d07.t7<br>sb_gmnlkrta_0001g08.t7	Cluster: PREDICTED: similar to ribosomal protein L15; n=1; Macaca mulatta|Rep: PREDICTED: similar to ribosomal protein L15 - Macaca mulatta		1e-66	93% (119/127)	ES780698<br>ES780729<br>ES780695<br>ES780781
sb_gmnlkrta.4.C1	3	sb_gmnlkrta_0001d04.t7<br>sb_gmnlkrta_0001e05.t7<br>sb_gmnlkrta_0001g12.t7	LSU rRNA; Xenopus borealis		0.0	97% (346/354)	ES780702<br>ES780779<br>ES780739
sb_gmnlkrta.6.C1	3	sb_gmnlkrta_0001b09.t7<br>sb_gmnlkrta_0001f02.t7<br>sb_gmnlkrta_0001f12.t7	Cluster: PREDICTED: similar to Actin, cytoplasmic 2 (Gamma-actin); n=1; Rattus norvegicus|Rep: PREDICTED: similar to Actin, cytoplasmic 2 (Gamma-actin) - Rattus norvegicus		4e-75	94% (138/146)	ES780764<br>ES780733<br>ES780750
sb_gmnlkrta.7.C1	2	sb_gmnlkrta_0001b06.t7<br>sb_gmnlkrta_0001e03.t7	Cluster: 40S ribosomal protein S13; n=16; Euteleostomi|Rep: 40S ribosomal protein S13 - Homo sapiens (Human)		6e-58	96% (112/116)	ES780780<br>ES780774
sb_gmnlkrta_0001a02.t7	1		Cluster: PREDICTED: similar to 40S ribosomal protein S7 (S8); n=1; Macaca mulatta|Rep: PREDICTED: similar to 40S ribosomal protein S7 (S8) - Macaca mulatta		7e-16	93% (41/44)	ES780742
sb_gmnlkrta_0001a03.t7	1		Cluster: Hemoglobin subunit alpha-1; n=3; Gadidae|Rep: Hemoglobin subunit alpha-1 - Gadus morhua (Atlantic cod)		6e-53	95% (101/106)	ES780743
sb_gmnlkrta_0001a05.t7	1		unclassified				ES780727
sb_gmnlkrta_0001a11.t7	1		Cluster: Ubiquitin.; n=5; Amniota|Rep: Ubiquitin. - Canis familiaris		6e-25	98% (57/58)	ES780751
sb_gmnlkrta_0001b02.t7	1		LSU rRNA; Oncorhynchus mykiss		0.0	95% (416/436)	ES780768

sb_gmnlkrta_0001b03.t7	1		unclassified				ES780769
sb_gmnlkrta_0001b05.t7	1		unclassified				ES780776
sb_gmnlkrta_0001b07.t7	1		Cluster: Heat shock protein 90 beta; n=7; Euteleostomi|Rep: Heat shock protein 90 beta - Paralichthys olivaceus (Japanese flounder)		5e-30	76% (38/50)	ES780783
sb_gmnlkrta_0001c01.t7	1		Cluster: Transcribed locus, strongly similar to NP_002286.2 laminin receptor 1 (ribosomal protein SA, 67kDa) [Homo sapiens]; n=1; Takifugu rubripes|Rep: Transcribed locus, strongly similar to NP_002286.2 laminin receptor 1 (ribosomal protein SA, 67kDa) [Homo sapiens] - Takifugu rubripes		4e-68	93% (83/89)	ES780710
sb_gmnlkrta_0001c08.t7	1		Cluster: 3 beta-hydroxysteroid dehydrogenase; n=2; Salmonidae|Rep: 3 beta-hydroxysteroid dehydrogenase - Oncorhynchus mykiss (Rainbow trout) (Salmo gairdneri)		4e-33	66% (62/93)	ES780725
sb_gmnlkrta_0001c09.t7	1		LSU rRNA; Oncorhynchus mykiss		1e-130	94% (280/296)	ES780731
sb_gmnlkrta_0001c11.t7	1		Cluster: Ribosomal protein L18; n=3; Euteleostomi|Rep: Ribosomal protein L18 - Pagrus major (Red sea bream) (Chrysophrys major)		4e-48	77% (92/118)	ES780766
sb_gmnlkrta_0001d02.t7	1		unclassified				ES780709
sb_gmnlkrta_0001d11.t7	1		Cluster: Protein YIPF5; n=4; Clupeocephala|Rep: Protein YIPF5 - Brachydanio rerio (Zebrafish) (Danio rerio)		5e-37	92% (77/83)	ES780772

sb_gmnlkrta_0001e02.t7	1		unclassified				ES780770
sb_gmnlkrta_0001e04.t7	1		Cluster: Hemoglobin subunit alpha-1; n=3; Gadidae|Rep: Hemoglobin subunit alpha-1 - Gadus morhua (Atlantic cod)		6e-53	95% (101/106)	ES780784
sb_gmnlkrta_0001e06.t7	1		Cluster: Hemoglobin subunit alpha-1; n=3; Gadidae|Rep: Hemoglobin subunit alpha-1 - Arctogadus glacialis (Arctic cod)		1e-11	96% (31/32)	ES780775
sb_gmnlkrta_0001e07.t7	1		Cluster: Hemoglobin subunit beta-2; n=3; Gadidae|Rep: Hemoglobin subunit beta-2 - Arctogadus glacialis (Arctic cod)		7e-47	95% (93/97)	ES780778
sb_gmnlkrta_0001e08.t7	1		Cd53; CD53 antigen [KO:K06489]		8e-22	36% (45/123)	ES780755
sb_gmnlkrta_0001e10.t7	1		Cluster: Hemoglobin subunit beta-2; n=3; Gadidae|Rep: Hemoglobin subunit beta-2 - Arctogadus glacialis (Arctic cod)		2e-41	98% (60/61)	ES780714
sb_gmnlkrta_0001f01.t7	1		Cluster: 60S ribosomal protein L7a; n=1; Ictalurus punctatus|Rep: 60S ribosomal protein L7a - Ictalurus punctatus (Channel catfish)		2e-43	91% (86/94)	ES780741
sb_gmnlkrta_0001f05.t7	1		unclassified				ES780717
sb_gmnlkrta_0001f11.t7	1		Cluster: Hemoglobin subunit alpha-1; n=3; Gadidae|Rep: Hemoglobin subunit alpha-1 - Gadus morhua (Atlantic cod)		6e-53	95% (101/106)	ES780746
sb_gmnlkrta_0001g01.t7	1		unclassified				ES780752

sb_gmnlkrta_0001g03.t7	1		Cluster: 60S ribosomal protein L13; n=1; Ictalurus punctatus|Rep: 60S ribosomal protein L13 - Ictalurus punctatus (Channel catfish)		9e-43	67% (88/131)	ES780749
sb_gmnlkrta_0001g04.t7	1		Cluster: 60S ribosomal protein L31; n=45; Tetrapoda|Rep: 60S ribosomal protein L31 - Pongo pygmaeus (Orangutan)		2e-37	91% (72/79)	ES780759
sb_gmnlkrta_0001g05.t7	1		unclassified				ES780754
sb_gmnlkrta_0001g09.t7	1		Cluster: 60S ribosomal protein L27A; n=3; Percomorpha|Rep: 60S ribosomal protein L27A - Platichthys flesus (European flounder)		8e-50	93% (93/99)	ES780782
sb_gmnlkrta_0001g11.t7	1		Cluster: Homolog of Homo sapiens "Collagen alpha 1(IV) chain precursor; n=1; Takifugu rubripes|Rep: Homolog of Homo sapiens "Collagen alpha 1(IV) chain precursor - Takifugu rubripes		8e-51	95% (92/96)	ES780735
sb_gmnlkrta_0001h01.t7	1		Cluster: 60S ribosomal protein L10a (CSA-19).; n=3; Eutheria|Rep: 60S ribosomal protein L10a (CSA-19). - Homo sapiens		2e-73	79% (142/179)	ES780728
sb_gmnlkrta_0001h05.t7	1		Cluster: Hemoglobin subunit beta-2; n=3; Gadidae|Rep: Hemoglobin subunit beta-2 - Arctogadus glacialis (Arctic cod)		7e-47	95% (93/97)	ES780738
sb_gmnlkrta_0001h06.t7	1		LSU rRNA; Hydrolagus colliei		3e-62	98% (124/126)	ES780744
sb_gmnlkrta_0001h07.t7	1		Cluster: Elongation factor-1 alpha; n=57; Euteleostomi|Rep: Elongation factor-1 alpha - Leptodoras sp. 1-GM-2003		3e-70	88% (78/88)	ES780740
sb_gmnlkrta_0001h09.t7	1		Cluster: 60S ribosomal protein L35a; n=33; Tetrapoda|Rep: 60S ribosomal		4e-46	85% (85/99)	ES780711

			protein L35a - Homo sapiens (Human)				
sb_gmnlkrta_0001h10.t7	1		Cluster: Hemoglobin subunit alpha-1; n=3; Gadidae|Rep: Hemoglobin subunit alpha-1 - Gadus morhua (Atlantic cod)		2e-53	91% (103/113)	ES780756

[1]Annotations presented in the supplemental table were generated with AutoFACT [18], while annotations presented in the manuscript are recent BLASTx hits that reflect a more updated state of the NCBI's nr protein database.

Supplemental Table S2B. Contigs in library gmnllrta (reverse heat-shock liver SSH library) with supporting annotations[1], statistics, and contributing EST accession numbers

Sequence	Count	Sub-sequences	AutoFACT Description	GO terms	E-value	Identity	Accession Number
sb_gmnllrta.33.C1	25	sb_gmnllrta_0007f22.t7<br>sb_gmnllrta_0004n10.t7<br>sb_gmnllrta_0005n05.t7<br>sb_gmnllrta_0001h09.t7<br>sb_gmnllrta_0001g04.t7<br>sb_gmnllrta_0003c11.t7<br>sb_gmnllrta_0003c07.t7<br>sb_gmnllrta_0003f02.t7<br>sb_gmnllrta_0007h17.t7<br>sb_gmnllrta_0002e23.t7<br>sb_gmnllrta_0002g07.t7<br>sb_gmnllrta_0002h24.t7<br>sb_gmnllrta_0003c19.t7<br>sb_gmnllrta_0003d19.t7<br>sb_gmnllrta_0004j18.t7<br>sb_gmnllrta_0004k22.t7<br>sb_gmnllrta_0004l04.t7<br>sb_gmnllrta_0005c08.t7<br>sb_gmnllrta_0005l03.t7<br>sb_gmnllrta_0005n03.t7<br>sb_gmnllrta_0007b11.t7<br>sb_gmnllrta_0007c11.t7<br>sb_gmnllrta_0007j15.t7<br>sb_gmnllrta_0007o22.t7<br>sb_gmnllrta_0003i02.t7	Cluster: Hemoglobin subunit alpha-1; n=3; Gadidae|Rep: Hemoglobin subunit alpha-1 - Gadus morhua (Atlantic cod)	GO:0005344<br>GO:0005506<br>GO:0005833<br>GO:0006810<br>GO:0015671<br>GO:0019825<br>GO:0020037	1e-68	96% (130/135)	FL634412<br>ES782117<br>ES783255<br>ES783144<br>ES782618<br>ES782355<br>ES782733<br>ES783161<br>FL634446<br>ES782051<br>ES782146<br>ES782892<br>ES782186<br>ES782249<br>ES783037<br>ES782971<br>ES782684<br>ES782259<br>ES782633<br>ES783043<br>FL634340<br>FL634358<br>FL634475<br>FL634567<br>ES782173
sb_gmnllrta.1.C1	21	sb_gmnllrta_0007m21.t7<br>sb_gmnllrta_0004c18.t7<br>sb_gmnllrta_0007g13.t7<br>sb_gmnllrta_0005o13.t7<br>sb_gmnllrta_0002k19.t7<br>sb_gmnllrta_0005m11.t7<br>sb_gmnllrta_0005f24.t7<br>sb_gmnllrta_0005e08.t7<br>sb_gmnllrta_0004j17.t7<br>sb_gmnllrta_0006b21.t7<br>sb_gmnllrta_0003b08.t7<br>sb_gmnllrta_0002e10.t7<br>sb_gmnllrta_0003d11.t7<br>sb_gmnllrta_0003o20.t7<br>sb_gmnllrta_0006e15.t7<br>sb_gmnllrta_0007a04.t7<br>sb_gmnllrta_0007i23.t7<br>sb_gmnllrta_0007m16.t7<br>sb_gmnllrta_0004a21.t7<br>sb_gmnllrta_0006m10.t7<br>sb_gmnllrta_0004m21.t7	Cluster: Hemoglobin subunit beta-2; n=3; Gadidae|Rep: Hemoglobin subunit beta-2 - Gadus morhua (Atlantic cod)	GO:0020037<br>GO:0019825<br>GO:0015671<br>GO:0006810<br>GO:0005833<br>GO:0005506<br>GO:0005344	3e-42	94% (88/93)	FL634531<br>ES783156<br>FL634423<br>ES782959<br>ES783040<br>ES782669<br>ES782437<br>ES782952<br>ES782764<br>ES782880<br>ES782273<br>ES782768<br>ES782450<br>ES782916<br>ES782078<br>FL634321<br>FL634463<br>FL634526<br>ES782426<br>ES783192<br>ES782188
sb_gmnllrta.3.C1	21	sb_gmnllrta_0007o19.t7<br>sb_gmnllrta_0007g16.t7<br>sb_gmnllrta_0007h11.t7<br>sb_gmnllrta_0007g22.t7<br>sb_gmnllrta_0002g09.t7<br>sb_gmnllrta_0007f05.t7<br>sb_gmnllrta_0007h08.t7<br>sb_gmnllrta_0007n15.t7	Cluster: Hemoglobin subunit beta-1; n=2; Gadidae|Rep: Hemoglobin subunit beta-1 - Gadus morhua (Atlantic cod)	GO:0005344<br>GO:0005506<br>GO:0005833<br>GO:0006810<br>GO:0015671<br>GO:0019825	3e-71	98% (129/131)	FL634564<br>FL634426<br>FL634442<br>FL634431<br>ES782443<br>FL634402<br>FL634439<br>FL634544

		sb_gmnllrta_0002m21.t7<br>sb_gmnllrta_0002i05.t7<br>sb_gmnllrta_0007c01.t7<br>sb_gmnllrta_0004h17.t7<br>sb_gmnllrta_0002e22.t7<br>sb_gmnllrta_0001f06.t7<br>sb_gmnllrta_0001g06.t7<br>sb_gmnllrta_0006f03.t7<br>sb_gmnllrta_0006i24.t7<br>sb_gmnllrta_0006i15.t7<br>sb_gmnllrta_0006g08.t7<br>sb_gmnllrta_0006c08.t7<br>sb_gmnllrta_0006m15.t7		GO:0020037			ES782993<br>ES782199<br>FL634349<br>ES783027<br>ES782015<br>ES782642<br>ES782484<br>ES782617<br>ES783107<br>ES782536<br>ES783141<br>ES782320<br>ES783005
sb_gmnllrta.65.C1	14	sb_gmnllrta_0006p07.t7<br>sb_gmnllrta_0006a19.t7<br>sb_gmnllrta_0001e02.t7<br>sb_gmnllrta_0002k24.t7<br>sb_gmnllrta_0002n12.t7<br>sb_gmnllrta_0004b14.t7<br>sb_gmnllrta_0004p21.t7<br>sb_gmnllrta_0006i10.t7<br>sb_gmnllrta_0006m07.t7<br>sb_gmnllrta_0006p13.t7<br>sb_gmnllrta_0007b10.t7<br>sb_gmnllrta_0006c13.t7<br>sb_gmnllrta_0006f02.t7<br>sb_gmnllrta_0006g19.t7	zgc:73262; zgc:73262; K02978 small subunit ribosomal protein S27e		2e-33	87% (68/78)	ES782749<br>ES783172<br>ES781999<br>ES782024<br>ES783184<br>ES782387<br>ES782773<br>ES782405<br>ES782154<br>ES782439<br>FL634339<br>ES783001<br>ES782557<br>ES782134
sb_gmnllrta.0.C1	13	sb_gmnllrta_0006i05.t7<br>sb_gmnllrta_0007k20.t7<br>sb_gmnllrta_0007h10.t7<br>sb_gmnllrta_0005j06.t7<br>sb_gmnllrta_0003n19.t7<br>sb_gmnllrta_0006f13.t7<br>sb_gmnllrta_0002a22.t7<br>sb_gmnllrta_0002d24.t7<br>sb_gmnllrta_0005i03.t7<br>sb_gmnllrta_0005p08.t7<br>sb_gmnllrta_0007i14.t7<br>sb_gmnllrta_0007l13.t7<br>sb_gmnllrta_0007n10.t7	Unassigned protein				ES782781<br>FL634497<br>FL634441<br>ES782376<br>ES782909<br>ES782691<br>ES782786<br>ES782987<br>ES782973<br>ES782799<br>FL634458<br>FL634507<br>FL634540
sb_gmnllrta.6.C1	13	sb_gmnllrta_0002o20.t7<br>sb_gmnllrta_0002p16.t7<br>sb_gmnllrta_0004o09.t7<br>sb_gmnllrta_0004k10.t7<br>sb_gmnllrta_0005j12.t7<br>sb_gmnllrta_0005b10.t7<br>sb_gmnllrta_0005i10.t7<br>sb_gmnllrta_0002c16.t7<br>sb_gmnllrta_0002d09.t7<br>sb_gmnllrta_0003g06.t7<br>sb_gmnllrta_0006j15.t7<br>sb_gmnllrta_0006l20.t7<br>sb_gmnllrta_0006k20.t7	Cluster: UTP--glucose-1-phosphate uridylyltransferase 2 (EC 2.7.7.9) (UDP-glucose pyrophosphorylase 2) (UDPGP 2) (UGPase 2).; n=1; Takifugu rubripes|Rep: UTP--glucose-1-phosphate uridylyltransferase 2 (EC 2.7.7.9) (UDP-glucose pyrophosphorylase 2) (UDPGP 2) (UGPase 2). - Takifugu rubripes		3e-39	94% (79/84)	ES782723<br>ES783031<br>ES782518<br>ES782442<br>ES782672<br>ES782347<br>ES782167<br>ES782337<br>ES783181<br>ES782297<br>ES783090<br>ES782714<br>ES782643
sb_gmnllrta.8.C1	13	sb_gmnllrta_0005c19.t7<br>sb_gmnllrta_0006d20.t7<br>sb_gmnllrta_0007g09.t7<br>sb_gmnllrta_0005f03.t7	unclassified				ES782995<br>ES782344<br>FL634419<br>ES782043

		sb_gmnllrta_0005p09.t7<br>sb_gmnllrta_0006g13.t7<br>sb_gmnllrta_0007g15.t7<br>sb_gmnllrta_0005n10.t7<br>sb_gmnllrta_0007c09.t7<br>sb_gmnllrta_0006e03.t7<br>sb_gmnllrta_0005l10.t7<br>sb_gmnllrta_0006a18.t7<br>sb_gmnllrta_0006c03.t7					ES782859<br>ES782533<br>FL634425<br>ES782260<br>FL634356<br>ES783010<br>ES782501<br>ES783110<br>ES782280
sb_gmnllrta.1.C2	12	sb_gmnllrta_0006p10.t7<br>sb_gmnllrta_0006m19.t7<br>sb_gmnllrta_0006k05.t7<br>sb_gmnllrta_0006o06.t7<br>sb_gmnllrta_0007h07.t7<br>sb_gmnllrta_0006n02.t7<br>sb_gmnllrta_0005g07.t7<br>sb_gmnllrta_0002a20.t7<br>sb_gmnllrta_0005j03.t7<br>sb_gmnllrta_0007k10.t7<br>sb_gmnllrta_0004e04.t7<br>sb_gmnllrta_0003h04.t7	Cluster: Hemopexin-like protein; n=1; Gillichthys mirabilis|Rep: Hemopexin-like protein - Gillichthys mirabilis (Long-jawed mudsucker)	GO:0003824	2e-37	58% (78/133)	ES782350<br>ES782710<br>ES783016<br>ES782619<br>FL634438<br>ES782821<br>ES783104<br>ES782918<br>ES782578<br>FL634490<br>ES782887<br>ES783151
sb_gmnllrta.2.C1	8	sb_gmnllrta_0004l18.t7<br>sb_gmnllrta_0005d14.t7<br>sb_gmnllrta_0006c16.t7<br>sb_gmnllrta_0006f05.t7<br>sb_gmnllrta_0006i06.t7<br>sb_gmnllrta_0006i16.t7<br>sb_gmnllrta_0006j05.t7<br>sb_gmnllrta_0006j12.t7	unclassified				ES782180<br>ES782676<br>ES783190<br>ES782397<br>ES782666<br>ES782587<br>ES782224<br>ES783245
sb_gmnllrta.25.C1	8	sb_gmnllrta_0007k03.t7<br>sb_gmnllrta_0005m24.t7<br>sb_gmnllrta_0002b15.t7<br>sb_gmnllrta_0005m03.t7<br>sb_gmnllrta_0005p14.t7<br>sb_gmnllrta_0007g17.t7<br>sb_gmnllrta_0007p20.t7<br>sb_gmnllrta_0001b03.t7	Cluster: Heat shock cognate 71 kDa protein; n=25; Clupeocephala|Rep: Heat shock cognate 71 kDa protein - Danio rerio (Zebrafish) (Brachydanio rerio)		2e-46	73% (99/135)	FL634485<br>ES782243<br>ES782870<br>ES782472<br>ES782056<br>FL634427<br>FL634578<br>ES782132
sb_gmnllrta.50.C1	8	sb_gmnllrta_0004i23.t7<br>sb_gmnllrta_0003j11.t7<br>sb_gmnllrta_0006j09.t7<br>sb_gmnllrta_0005h15.t7<br>sb_gmnllrta_0005k14.t7<br>sb_gmnllrta_0003e14.t7<br>sb_gmnllrta_0007k12.t7<br>sb_gmnllrta_0001c01.t7	Cluster: Ferritin, heavy subunit; n=2; Salmonidae|Rep: Ferritin, heavy subunit - Salmo salar (Atlantic salmon)	GO:0004322<br>GO:0005488<br>GO:0005506<br>GO:0006826<br>GO:0006879<br>GO:0008199<br>GO:0016491<br>GO:0046872	1e-89	89% (157/176)	ES782743<br>ES782540<br>ES782562<br>ES783232<br>ES782329<br>ES782656<br>FL634492<br>ES783109
sb_gmnllrta.57.C1	8	sb_gmnllrta_0003o18.t7<br>sb_gmnllrta_0003i17.t7<br>sb_gmnllrta_0006c14.t7<br>sb_gmnllrta_0007e10.t7<br>sb_gmnllrta_0006k12.t7<br>sb_gmnllrta_0005f18.t7	unclassified				ES782418<br>ES783179<br>ES783166<br>FL634392<br>ES782137<br>ES782623

		sb_gmnllrta_0007p07.t7<br>sb_gmnllrta_0005b13.t7					FL634570<br>ES782440
sb_gmnllrta.78.C1	8	sb_gmnllrta_0001c06.t7<br>sb_gmnllrta_0005i02.t7<br>sb_gmnllrta_0006h09.t7<br>sb_gmnllrta_0006k23.t7<br>sb_gmnllrta_0007a07.t7<br>sb_gmnllrta_0007g20.t7<br>sb_gmnllrta_0007n13.t7<br>sb_gmnllrta_0007m18.t7	unclassified				ES782947<br>ES782988<br>ES782190<br>ES782712<br>FL634322<br>FL634429<br>FL634542<br>FL634528
sb_gmnllrta.10.C1	7	sb_gmnllrta_0005m09.t7<br>sb_gmnllrta_0002d05.t7<br>sb_gmnllrta_0003b07.t7<br>sb_gmnllrta_0002h17.t7<br>sb_gmnllrta_0002l17.t7<br>sb_gmnllrta_0005e13.t7<br>sb_gmnllrta_0005b23.t7	LSU rRNA; Oncorhynchus mykiss		1e-158	94% (346/367)	ES782100<br>ES782896<br>ES782580<br>ES782165<br>ES782659<br>ES782351<br>ES783091
sb_gmnllrta.141.C1	6	sb_gmnllrta_0003n06.t7<br>sb_gmnllrta_0005f08.t7<br>sb_gmnllrta_0007h21.t7<br>sb_gmnllrta_0004m12.t7<br>sb_gmnllrta_0006a23.t7<br>sb_gmnllrta_0007n23.t7	Cluster: Ferritin, middle subunit; n=5; Euteleostei|Rep: Ferritin, middle subunit - Salmo salar (Atlantic salmon)	GO:0004322<br>GO:0005488<br>GO:0005506<br>GO:0006826<br>GO:0006879<br>GO:0008199<br>GO:0016491<br>GO:0046872	3e-22	70% (51/72)	ES782032<br>ES782542<br>FL634449<br>ES782871<br>ES782130<br>FL634551
sb_gmnllrta.17.C1	6	sb_gmnllrta_0007m24.t7<br>sb_gmnllrta_0007i08.t7<br>sb_gmnllrta_0006l09.t7<br>sb_gmnllrta_0006i20.t7<br>sb_gmnllrta_0001a12.t7<br>sb_gmnllrta_0007h05.t7	LSU rRNA; Xenopus borealis		1e-165	97% (318/326)	FL634534<br>FL634454<br>ES782635<br>ES783063<br>ES782404<br>FL634436
sb_gmnllrta.22.C1	6	sb_gmnllrta_0007h19.t7<br>sb_gmnllrta_0005a23.t7<br>sb_gmnllrta_0004a20.t7<br>sb_gmnllrta_0001b08.t7<br>sb_gmnllrta_0005k12.t7<br>sb_gmnllrta_0005o01.t7	Cluster: Hemoglobin subunit alpha-2; n=3; Gadidae|Rep: Hemoglobin subunit alpha-2 - Gadus morhua (Atlantic cod)	GO:0005344<br>GO:0005506<br>GO:0005833<br>GO:0006810<br>GO:0015671<br>GO:0019825<br>GO:0020037	1e-34	98% (71/72)	FL634447<br>ES782521<br>ES782416<br>ES782478<br>ES782512<br>ES782197
sb_gmnllrta.36.C1	6	sb_gmnllrta_0006d01.t7<br>sb_gmnllrta_0006e11.t7<br>sb_gmnllrta_0006k24.t7<br>sb_gmnllrta_0006m12.t7<br>sb_gmnllrta_0002m11.t7<br>sb_gmnllrta_0007o24.t7	unclassified				ES782267<br>ES782192<br>ES782874<br>ES783163<br>ES782356<br>FL634568

sb_gmnllrta.61.C1	6	sb_gmnllrta_0005a18.t7<br>sb_gmnllrta_0005f20.t7<br>sb_gmnllrta_0005h08.t7<br>sb_gmnllrta_0005k11.t7<br>sb_gmnllrta_0006f01.t7<br>sb_gmnllrta_0007k24.t7	unclassified				ES782728<br>ES782522<br>ES782448<br>ES782608<br>ES782483<br>FL634500
sb_gmnllrta.9.C1	6	sb_gmnllrta_0002a18.t7<br>sb_gmnllrta_0003k17.t7<br>sb_gmnllrta_0005a13.t7<br>sb_gmnllrta_0005e11.t7<br>sb_gmnllrta_0005j20.t7<br>sb_gmnllrta_0005p02.t7	Cluster: Cathepsin L; n=2; Percomorpha|Rep: Cathepsin L - Lates calcarifer (Barramundi)		3e-32	86% (63/73)	ES782419<br>ES782927<br>ES783177<br>ES782390<br>ES782090<br>ES783066
sb_gmnllrta.104.C1	5	sb_gmnllrta_0005j05.t7<br>sb_gmnllrta_0001g05.t7<br>sb_gmnllrta_0003h24.t7<br>sb_gmnllrta_0006b05.t7<br>sb_gmnllrta_0002a23.t7	si:ch211-234p6.13; si:ch211-234p6.13		1e-13	45% (33/73)	ES782435<br>ES782556<br>ES782747<br>ES783217<br>ES782847
sb_gmnllrta.117.C1	5	sb_gmnllrta_0006p15.t7<br>sb_gmnllrta_0006p02.t7<br>sb_gmnllrta_0005e07.t7<br>sb_gmnllrta_0004a09.t7<br>sb_gmnllrta_0005i19.t7	unclassified				ES782544<br>ES782793<br>ES782686<br>ES782330<br>ES782308
sb_gmnllrta.23.C1	5	sb_gmnllrta_0006g06.t7<br>sb_gmnllrta_0006l08.t7<br>sb_gmnllrta_0006n08.t7<br>sb_gmnllrta_0006o09.t7<br>sb_gmnllrta_0007l08.t7	unclassified				ES782802<br>ES782692<br>ES783089<br>ES782237<br>FL634504
sb_gmnllrta.24.C1	5	sb_gmnllrta_0006j14.t7<br>sb_gmnllrta_0004o24.t7<br>sb_gmnllrta_0005n16.t7<br>sb_gmnllrta_0002a07.t7<br>sb_gmnllrta_0006l14.t7	Cluster: PREDICTED: similar to alpha-NAC, muscle-specific form gp220; n=1; Monodelphis domestica|Rep: PREDICTED: similar to alpha-NAC, muscle-specific form gp220 - Monodelphis domestica		4e-37	100% (81/81)	ES783028<br>ES782371<br>ES782125<br>ES783148<br>ES782191
sb_gmnllrta.35.C1	5	sb_gmnllrta_0005m14.t7<br>sb_gmnllrta_0005g10.t7<br>sb_gmnllrta_0005l13.t7<br>sb_gmnllrta_0005o21.t7<br>sb_gmnllrta_0005p23.t7	Cluster: PREDICTED: similar to Actin, cytoplasmic 2 (Gamma-actin); n=1; Rattus norvegicus|Rep: PREDICTED: similar to Actin, cytoplasmic 2 (Gamma-actin) - Rattus norvegicus		6e-45	92% (88/95)	ES782865<br>ES782068<br>ES782549<br>ES782425<br>ES782820

sb_gmnllrta.37.C1	5	sb_gmnllrta_0007e16.t7<br>sb_gmnllrta_0003c05.t7<br>sb_gmnllrta_0004f22.t7<br>sb_gmnllrta_0007a10.t7<br>sb_gmnllrta_0004i02.t7	Cluster: Serotransferrin; n=1; Gadus morhua|Rep: Serotransferrin - Gadus morhua (Atlantic cod)	GO:0005506<br>GO:0005576<br>GO:0006810<br>GO:0006811<br>GO:0006826<br>GO:0006879<br>GO:0008199	2e-74	96% (137/142)	FL634395<br>ES782678<br>ES782429<br>FL634324<br>ES783136
sb_gmnllrta.7.C1	5	sb_gmnllrta_0003c21.t7<br>sb_gmnllrta_0007c14.t7<br>sb_gmnllrta_0004b07.t7<br>sb_gmnllrta_0004o05.t7<br>sb_gmnllrta_0005d01.t7	unclassified				ES782992<br>FL634361<br>ES782891<br>ES782245<br>ES782311
sb_gmnllrta.112.C1	4	sb_gmnllrta_0006l05.t7<br>sb_gmnllrta_0006a15.t7<br>sb_gmnllrta_0006l16.t7<br>sb_gmnllrta_0006k16.t7	unclassified				ES783081<br>ES782883<br>ES782289<br>ES782217
sb_gmnllrta.113.C1	4	sb_gmnllrta_0004a16.t7<br>sb_gmnllrta_0003c20.t7<br>sb_gmnllrta_0003n20.t7<br>sb_gmnllrta_0007b15.t7	Cluster: PREDICTED: similar to Actin, cytoplasmic 2 (Gamma-actin); n=1; Rattus norvegicus|Rep: PREDICTED: similar to Actin, cytoplasmic 2 (Gamma-actin) - Rattus norvegicus		5e-24	96% (54/56)	ES783230<br>ES782967<br>ES782431<br>FL634343
sb_gmnllrta.124.C1	4	sb_gmnllrta_0003l09.t7<br>sb_gmnllrta_0006a14.t7<br>sb_gmnllrta_0007o18.t7<br>sb_gmnllrta_0006b12.t7	SPRY domain containing protein		5e-06	33% (18/53)	ES782118<br>ES782905<br>FL634563<br>ES782194
sb_gmnllrta.13.C1	4	sb_gmnllrta_0002g05.t7<br>sb_gmnllrta_0007o09.t7<br>sb_gmnllrta_0005p06.t7<br>sb_gmnllrta_0005b17.t7	Cluster: Ribosomal protein Sa; n=2; Percomorpha|Rep: Ribosomal protein Sa - Solea senegalensis (Sole)	GO:0003735<br>GO:0005622<br>GO:0005840<br>GO:0006412<br>GO:0015935	5e-57	97% (105/108)	ES782025<br>FL634555<br>ES783111<br>ES782514
sb_gmnllrta.159.C1	4	sb_gmnllrta_0002g22.t7<br>sb_gmnllrta_0002k16.t7<br>sb_gmnllrta_0004m11.t7<br>sb_gmnllrta_0007p23.t7	unclassified				ES782407<br>ES782760<br>ES782817<br>FL634580
sb_gmnllrta.164.C1	4	sb_gmnllrta_0006h04.t7<br>sb_gmnllrta_0007h20.t7<br>sb_gmnllrta_0006j23.t7<br>sb_gmnllrta_0006m18.t7	unclassified				ES782551<br>FL634448<br>ES782555<br>ES782777

sb_gmnllrta.38.C1	4	sb_gmnllrta_0004a17.t7<br>sb_gmnllrta_0002g14.t7<br>sb_gmnllrta_0005b08.t7<br>sb_gmnllrta_0007c20.t7	Unassigned protein				ES783208<br>ES783229<br>ES783049<br>FL634367
sb_gmnllrta.47.C1	4	sb_gmnllrta_0005i23.t7<br>sb_gmnllrta_0005d07.t7<br>sb_gmnllrta_0007j17.t7<br>sb_gmnllrta_0004j05.t7	unclassified				ES782894<br>ES782530<br>FL634477<br>ES782568
sb_gmnllrta.5.C1	4	sb_gmnllrta_0002n13.t7<br>sb_gmnllrta_0003a19.t7<br>sb_gmnllrta_0005l02.t7<br>sb_gmnllrta_0007f18.t7	Cluster: PREDICTED: similar to reverse transcriptase-like; n=1; Strongylocentrotus purpuratus|Rep: PREDICTED: similar to reverse transcriptase-like - Strongylocentrotus purpuratus		5e-10	38% (35/91)	ES783120<br>ES782581<br>ES782677<br>FL634410
sb_gmnllrta.60.C1	4	sb_gmnllrta_0002k09.t7<br>sb_gmnllrta_0003e16.t7<br>sb_gmnllrta_0004e13.t7<br>sb_gmnllrta_0001d03.t7	Cluster: Ceruloplasmin; n=1; Chionodraco rastrospinosus|Rep: Ceruloplasmin - Chionodraco rastrospinosus (Ocellated icefish)	GO:0004322<br>GO:0005507<br>GO:0016491	5e-44	66% (84/126)	ES782168<br>ES782761<br>ES782497<br>ES783175
sb_gmnllrta.62.C1	4	sb_gmnllrta_0006k21.t7<br>sb_gmnllrta_0005d05.t7<br>sb_gmnllrta_0005f09.t7<br>sb_gmnllrta_0002n01.t7	unclassified				ES782663<br>ES782570<br>ES782613<br>ES782045
sb_gmnllrta.67.C1	4	sb_gmnllrta_0006b23.t7<br>sb_gmnllrta_0006d06.t7<br>sb_gmnllrta_0004a10.t7<br>sb_gmnllrta_0005l05.t7	LSU rRNA; Hydrolagus colliei		1e-102	99% (187/188)	ES782822<br>ES782109<br>ES783059<br>ES782850
sb_gmnllrta.71.C1	4	sb_gmnllrta_0006h22.t7<br>sb_gmnllrta_0005a08.t7<br>sb_gmnllrta_0003e17.t7<br>sb_gmnllrta_0006f21.t7	unclassified				ES782220<br>ES782491<br>ES782702<br>ES782113
sb_gmnllrta.72.C1	4	sb_gmnllrta_0006b14.t7<br>sb_gmnllrta_0006m08.t7<br>sb_gmnllrta_0002n20.t7<br>sb_gmnllrta_0006o15.t7	Cluster: Zgc:56419; n=1; Danio rerio|Rep: Zgc:56419 - Danio rerio (Zebrafish) (Brachydanio rerio)		3e-25	87% (57/65)	ES782055<br>ES782535<br>ES782572<br>ES782737

sb_gmnllrta.88.C1	4	sb_gmnllrta_0005b11.t7<br>sb_gmnllrta_0005c07.t7<br>sb_gmnllrta_0006e02.t7<br>sb_gmnllrta_0007p18.t7	Cluster: Transaldolase; n=1; Ctenopharyngodon idella|Rep: Transaldolase - Ctenopharyngodon idella (Grass carp)	GO:0003824<br>GO:0004801<br>GO:0005737<br>GO:0005975<br>GO:0006098<br>GO:0008152	9e-21	82% (46/56)	ES782339<br>ES782600<br>ES782946<br>FL634576
sb_gmnllrta.96.C1	4	sb_gmnllrta_0004d20.t7<br>sb_gmnllrta_0005b06.t7<br>sb_gmnllrta_0005a22.t7<br>sb_gmnllrta_0007c06.t7	Cluster: Putative flavin-monooxygenase; n=1; Takifugu rubripes|Rep: Putative flavin-monooxygenase - Fugu rubripes (Japanese pufferfish) (Takifugu rubripes)	GO:0003824<br>GO:0004497<br>GO:0004499<br>GO:0005488<br>GO:0005792<br>GO:0006118<br>GO:0008152<br>GO:0016491<br>GO:0031227<br>GO:0050660	9e-43	74% (84/113)	ES782092<br>ES782724<br>ES782473<br>FL634353
sb_gmnllrta.101.C1	3	sb_gmnllrta_0003a10.t7<br>sb_gmnllrta_0002b11.t7<br>sb_gmnllrta_0003i11.t7	unclassified				ES782103<br>ES782653<br>ES782954
sb_gmnllrta.106.C1	3	sb_gmnllrta_0006g18.t7<br>sb_gmnllrta_0006h18.t7<br>sb_gmnllrta_0006j18.t7	Cluster: Zgc:77098; n=2; Danio rerio|Rep: Zgc:77098 - Danio rerio (Zebrafish) (Brachydanio rerio)		5e-71	69% (133/192)	ES782108<br>ES783082<br>ES782668
sb_gmnllrta.109.C1	3	sb_gmnllrta_0007o15.t7<br>sb_gmnllrta_0004i18.t7<br>sb_gmnllrta_0003a11.t7	Cluster: Complement component C3; n=1; Anarhichas minor|Rep: Complement component C3 - Anarhichas minor (Arctic spotted wolffish)	GO:0005515<br>GO:0004866	2e-54	48% (90/186)	FL634560<br>ES782462<br>ES782124
sb_gmnllrta.110.C1	3	sb_gmnllrta_0007p17.t7<br>sb_gmnllrta_0003n17.t7<br>sb_gmnllrta_0007m17.t7	Cluster: Fibrinogen, B beta polypeptide; n=2; Danio rerio|Rep: Fibrinogen, B beta polypeptide - Danio rerio (Zebrafish) (Brachydanio rerio)		8e-28	86% (52/60)	FL634575<br>ES783225<br>FL634527
sb_gmnllrta.118.C1	3	sb_gmnllrta_0004g18.t7<br>sb_gmnllrta_0005c14.t7<br>sb_gmnllrta_0005k13.t7	Cluster: Transmembrane protein 50A; n=1; Danio rerio|Rep: Transmembrane protein 50A - Danio rerio (Zebrafish) (Brachydanio rerio)		5e-46	65% (103/157)	ES782373<br>ES782762<br>ES782498
sb_gmnllrta.12.C1	3	sb_gmnllrta_0006k03.t7<br>sb_gmnllrta_0004c06.t7<br>sb_gmnllrta_0006o18.t7	unclassified				ES783114<br>ES782432<br>ES783015

sb_gmnllrta.120.C1	3	sb_gmnllrta_0005m04.t7<br>sb_gmnllrta_0006j03.t7<br>sb_gmnllrta_0006f14.t7	Cluster: PREDICTED: similar to Antiquitin; n=1; Gallus gallus|Rep: PREDICTED: similar to Antiquitin - Gallus gallus		1e-44	90% (83/92)	ES782310<br>ES782001<br>ES782860
sb_gmnllrta.125.C1	3	sb_gmnllrta_0002a12.t7<br>sb_gmnllrta_0003e07.t7<br>sb_gmnllrta_0007p12.t7	Cluster: High mobility group protein B2 (High mobility group protein 2) (HMG- 2).; n=1; Takifugu rubripes|Rep: High mobility group protein B2 (High mobility group protein 2) (HMG- 2). - Takifugu rubripes		2e-10	54% (31/57)	ES782164<br>ES782468<br>FL634572
sb_gmnllrta.132.C1	3	sb_gmnllrta_0004c14.t7<br>sb_gmnllrta_0006a20.t7<br>sb_gmnllrta_0005p16.t7	Lss; lanosterol synthase [EC:5.4.99.7]; K01852 lanosterol synthase		5e-27	72% (51/70)	ES782866<br>ES782059<br>ES782081
sb_gmnllrta.135.C1	3	sb_gmnllrta_0002k21.t7<br>sb_gmnllrta_0005b12.t7<br>sb_gmnllrta_0006g03.t7	Cluster: MHC class Ia antigen; n=2; Gadus morhua|Rep: MHC class Ia antigen - Gadus morhua (Atlantic cod)	GO:0006955<br>GO:0016020<br>GO:0019882	2e-37	97% (72/74)	ES782299<br>ES782394<br>ES782776
sb_gmnllrta.136.C1	3	sb_gmnllrta_0003m06.t7<br>sb_gmnllrta_0003h09.t7<br>sb_gmnllrta_0003n05.t7	Cluster: Zgc:112265 protein; n=7; Danio rerio|Rep: Zgc:112265 protein - Danio rerio (Zebrafish) (Brachydanio rerio)		3e-12	30% (48/159)	ES782899<br>ES782925<br>ES782094
sb_gmnllrta.139.C1	3	sb_gmnllrta_0006a12.t7<br>sb_gmnllrta_0002g24.t7<br>sb_gmnllrta_0007m02.t7	Cluster: Ribosomal protein L7; n=4; Danio rerio|Rep: Ribosomal protein L7 - Danio rerio (Zebrafish) (Brachydanio rerio)		1e-66	83% (123/147)	ES782720<br>ES782595<br>FL634515
sb_gmnllrta.140.C1	3	sb_gmnllrta_0005i08.t7<br>sb_gmnllrta_0005g08.t7<br>sb_gmnllrta_0007o13.t7	Cluster: 60S ribosomal protein L35a; n=42; Euteleostomi|Rep: 60S ribosomal protein L35a - Homo sapiens (Human)		6e-43	87% (82/94)	ES782932<br>ES782697<br>FL634558
sb_gmnllrta.145.C1	3	sb_gmnllrta_0006c05.t7<br>sb_gmnllrta_0002l12.t7<br>sb_gmnllrta_0006o10.t7	Cluster: MOSC domain-containing protein 2, mitochondrial precursor (EC 1.-.-.-).; n=1; Takifugu rubripes|Rep: MOSC domain-containing protein 2, mitochondrial precursor (EC 1.-.-.-). - Takifugu rubripes		8e-41	65% (81/123)	ES782112<br>ES782929<br>ES782800

sb_gmnllrta.149.C1	3	sb_gmnllrta_0003k23.t7 sb_gmnllrta_0003l23.t7 sb_gmnllrta_0003a21.t7	unclassified				ES782052 ES782148 ES782753
sb_gmnllrta.151.C1	3	sb_gmnllrta_0005n04.t7 sb_gmnllrta_0007n11.t7 sb_gmnllrta_0005b19.t7	unclassified				ES783196 FL634541 ES782203
sb_gmnllrta.152.C1	3	sb_gmnllrta_0003k11.t7 sb_gmnllrta_0005c17.t7 sb_gmnllrta_0001e04.t7	Cluster: Ubiquitin/40S ribosomal protein S27a fusion protein; n=16; Metazoa|Rep: Ubiquitin/40S ribosomal protein S27a fusion protein - Argas monolakensis	GO:0003735 GO:0005622 GO:0005840 GO:0006412	5e-38	100% (80/80)	ES782696 ES782674 ES782219
sb_gmnllrta.154.C1	3	sb_gmnllrta_0003j09.t7 sb_gmnllrta_0006b03.t7 sb_gmnllrta_0006c24.t7	Cluster: PREDICTED: similar to mitogen-activated protein kinase kinase kinase kinase 4 isoform 1; n=1; Rattus norvegicus|Rep: PREDICTED: similar to mitogen-activated protein kinase kinase kinase kinase 4 isoform 1 - Rattus norvegicus		8e-06	88% (24/27)	ES783183 ES783079 ES782505
sb_gmnllrta.156.C1	3	sb_gmnllrta_0006m11.t7 sb_gmnllrta_0002b19.t7 sb_gmnllrta_0006j02.t7	unclassified				ES783244 ES783146 ES782062
sb_gmnllrta.16.C1	3	sb_gmnllrta_0006d03.t7 sb_gmnllrta_0007i24.t7 sb_gmnllrta_0007m09.t7	unclassified				ES782221 FL634464 FL634521
sb_gmnllrta.160.C1	3	sb_gmnllrta_0004f13.t7 sb_gmnllrta_0003k16.t7 sb_gmnllrta_0007e20.t7	unclassified				ES783055 ES782867 FL634398
sb_gmnllrta.166.C1	3	sb_gmnllrta_0003a01.t7 sb_gmnllrta_0003d12.t7 sb_gmnllrta_0004n09.t7	Cluster: Homolog of Pseudopleuronectes americanus "Renal organic anion transporter.; n=1; Takifugu rubripes|Rep: Homolog of Pseudopleuronectes americanus "Renal organic anion transporter. - Takifugu rubripes		9e-20	66% (57/86)	ES783157 ES782359 ES782673

sb_gmnllrta.19.C1	3	sb_gmnllrta_0005g13.t7<br>sb_gmnllrta_0007p24.t7<br>sb_gmnllrta_0004e14.t7	unclassified				ES782121<br>FL634581<br>ES782328
sb_gmnllrta.29.C1	3	sb_gmnllrta_0002k04.t7<br>sb_gmnllrta_0002m24.t7<br>sb_gmnllrta_0005g01.t7	Cluster: Ribosomal protein S2; n=5; Euteleostomi|Rep: Ribosomal protein S2 - Danio rerio (Zebrafish) (Brachydanio rerio)		1e-23	98% (55/56)	ES782574<br>ES783203<br>ES783199
sb_gmnllrta.32.C1	3	sb_gmnllrta_0006k02.t7<br>sb_gmnllrta_0004k04.t7<br>sb_gmnllrta_0004m16.t7	unclassified				ES783170<br>ES782755<br>ES782652
sb_gmnllrta.40.C1	3	sb_gmnllrta_0002m09.t7<br>sb_gmnllrta_0002n09.t7<br>sb_gmnllrta_0005n13.t7	Cluster: Antizyme inhibitor 1 (AZI) (Ornithine decarboxylase antizyme inhibitor).; n=1; Takifugu rubripes|Rep: Antizyme inhibitor 1 (AZI) (Ornithine decarboxylase antizyme inhibitor). - Takifugu rubripes		4e-51	64% (106/165)	ES783058<br>ES782500<br>ES782169
sb_gmnllrta.42.C1	3	sb_gmnllrta_0006a17.t7<br>sb_gmnllrta_0006g12.t7<br>sb_gmnllrta_0006g15.t7	unclassified				ES782858<br>ES782477<br>ES782319
sb_gmnllrta.44.C1	3	sb_gmnllrta_0006p14.t7<br>sb_gmnllrta_0005d02.t7<br>sb_gmnllrta_0005m01.t7	unclassified				ES782610<br>ES782413<br>ES782588
sb_gmnllrta.45.C1	3	sb_gmnllrta_0004o08.t7<br>sb_gmnllrta_0003d24.t7<br>sb_gmnllrta_0004p07.t7	unclassified				ES782492<br>ES783134<br>ES782942
sb_gmnllrta.51.C1	3	sb_gmnllrta_0007c04.t7<br>sb_gmnllrta_0004h10.t7<br>sb_gmnllrta_0005l06.t7	LOC562970; similar to polycystic kidney disease 1-like 2 short form		1e-24	79% (55/69)	FL634351<br>ES783182<br>ES782917
sb_gmnllrta.53.C1	3	sb_gmnllrta_0002f19.t7<br>sb_gmnllrta_0003i05.t7<br>sb_gmnllrta_0006h16.t7	Unassigned protein		1e-07	36% (28/76)	ES782369<br>ES782027<br>ES782738

sb_gmnllrta.56.C1	3	sb_gmnllrta_0005i22.t7<br>sb_gmnllrta_0005n12.t7<br>sb_gmnllrta_0003f05.t7	Cluster: Translationally-controlled tumor protein; n=1; Lateolabrax japonicus|Rep: Translationally-controlled tumor protein - Lateolabrax japonicus (Japanese sea perch) (Japanese sea bass)	GO:0005509	2e-36	65% (71/109)	ES782911<br>ES782225<br>ES782990
sb_gmnllrta.68.C1	3	sb_gmnllrta_0002h13.t7<br>sb_gmnllrta_0007d12.t7<br>sb_gmnllrta_0002h09.t7	Unassigned protein				ES782098<br>FL634378<br>ES782703
sb_gmnllrta.70.C1	3	sb_gmnllrta_0004p01.t7<br>sb_gmnllrta_0004a18.t7<br>sb_gmnllrta_0005l16.t7	Cluster: Peroxisomal sarcosine oxidase (EC 1.5.3.1) (PSO) (L-pipecolate oxidase) (EC 1.5.3.7) (L-pipecolic acid oxidase).; n=1; Takifugu rubripes|Rep: Peroxisomal sarcosine oxidase (EC 1.5.3.1) (PSO) (L-pipecolate oxidase) (EC 1.5.3.7) (L-pipecolic acid oxidase). - Takifugu rubripes		4e-15	79% (38/48)	ES783169<br>ES782914<br>ES782354
sb_gmnllrta.75.C1	3	sb_gmnllrta_0006c02.t7<br>sb_gmnllrta_0006p04.t7<br>sb_gmnllrta_0007b09.t7	unclassified				ES782270<br>ES782685<br>FL634338
sb_gmnllrta.77.C1	3	sb_gmnllrta_0002a01.t7<br>sb_gmnllrta_0003h18.t7<br>sb_gmnllrta_0002j16.t7	unclassified				ES782965<br>ES782313<br>ES782605
sb_gmnllrta.82.C1	3	sb_gmnllrta_0002e15.t7<br>sb_gmnllrta_0005f16.t7<br>sb_gmnllrta_0005n23.t7	unclassified				ES782814<br>ES782950<br>ES782792
sb_gmnllrta.97.C1	3	sb_gmnllrta_0006l12.t7<br>sb_gmnllrta_0006c15.t7<br>sb_gmnllrta_0007b03.t7	Cluster: 60S ribosomal protein L37a; n=36; Craniata|Rep: 60S ribosomal protein L37a - Homo sapiens (Human)		8e-40	100% (78/78)	ES782053<br>ES783143<br>FL634334
sb_gmnllrta.100.C1	2	sb_gmnllrta_0005m06.t7<br>sb_gmnllrta_0007l03.t7	Cluster: Keratin 15; n=6; Danio rerio|Rep: Keratin 15 - Danio rerio (Zebrafish) (Brachydanio rerio)		8e-05	85% (23/27)	ES782430<br>FL634501

sb_gmnllrta.102.C1	2	sb_gmnllrta_0005k08.t7<br>sb_gmnllrta_0007c10.t7	unclassified				ES783186<br>FL634357
sb_gmnllrta.103.C1	2	sb_gmnllrta_0007c12.t7<br>sb_gmnllrta_0006e04.t7	unclassified				FL634359<br>ES783174
sb_gmnllrta.105.C1	2	sb_gmnllrta_0007k22.t7<br>sb_gmnllrta_0007n06.t7	unclassified				FL634498<br>FL634538
sb_gmnllrta.107.C1	2	sb_gmnllrta_0006m05.t7<br>sb_gmnllrta_0007j08.t7	unclassified				ES782282<br>FL634468
sb_gmnllrta.108.C1	2	sb_gmnllrta_0004k08.t7<br>sb_gmnllrta_0004l07.t7	unclassified				ES782953<br>ES782752
sb_gmnllrta.11.C1	2	sb_gmnllrta_0005c02.t7<br>sb_gmnllrta_0005e17.t7	unclassified				ES782315<br>ES782615
sb_gmnllrta.111.C1	2	sb_gmnllrta_0007a17.t7<br>sb_gmnllrta_0007n24.t7	Cluster: similar to glutathione peroxidase 3 (LOC798788), mRNA; n=2; Danio rerio|Rep: similar to glutathione peroxidase 3 (LOC798788), mRNA - Danio rerio		6e-10	61% (29/47)	FL634330<br>FL634552
sb_gmnllrta.114.C1	2	sb_gmnllrta_0002i23.t7<br>sb_gmnllrta_0006p08.t7	unclassified				ES782569<br>ES783050
sb_gmnllrta.115.C1	2	sb_gmnllrta_0003e19.t7<br>sb_gmnllrta_0006i12.t7	unclassified				ES783041<br>ES782378
sb_gmnllrta.116.C1	2	sb_gmnllrta_0002f23.t7<br>sb_gmnllrta_0003p12.t7	Cluster: Protein transport protein Sec61 subunit alpha isoform A; n=48; Eumetazoa|Rep: Protein transport protein Sec61 subunit alpha isoform A - Danio		2e-25	98% (56/57)	ES782923<br>ES783004

			rerio (Zebrafish) (Brachydanio rerio)				
sb_gmnllrta.119.C1	2	sb_gmnllrta_0004l17.t7<br>sb_gmnllrta_0007a14.t7	unclassified				ES782515<br>FL634328
sb_gmnllrta.121.C1	2	sb_gmnllrta_0006f24.t7<br>sb_gmnllrta_0005k15.t7	Cluster: Tetraspanin-6 (Tspan-6) (Transmembrane 4 superfamily member 6) (T245 protein) (Tetraspanin TM4-D) (A15 homolog).; n=1; Takifugu rubripes|Rep: Tetraspanin-6 (Tspan-6) (Transmembrane 4 superfamily member 6) (T245 protein) (Tetraspanin TM4-D) (A15 homolog). - Takifugu rubripes		8e-09	93% (27/29)	ES782223<br>ES782358
sb_gmnllrta.122.C1	2	sb_gmnllrta_0002n14.t7<br>sb_gmnllrta_0003l16.t7	Cluster: Tetranectin precursor (TN) (C-type lectin domain family 3 member B) (Plasminogen kringle 4-binding protein).; n=1; Takifugu rubripes|Rep: Tetranectin precursor (TN) (C-type lectin domain family 3 member B) (Plasminogen kringle 4-binding protein). - Takifugu rubripes		8e-23	79% (47/59)	ES782960<br>ES782819
sb_gmnllrta.123.C1	2	sb_gmnllrta_0006d09.t7<br>sb_gmnllrta_0007o14.t7	Cluster: CCAAT/enhancer binding protein delta1; n=1; Oncorhynchus mykiss|Rep: CCAAT/enhancer binding protein delta1 - Oncorhynchus mykiss (Rainbow trout) (Salmo gairdneri)	GO:0006355<br>GO:0005634<br>GO:0003700<br>GO:0003677<br>GO:0043565	4e-15	71% (42/59)	ES782422<br>FL634559
sb_gmnllrta.126.C1	2	sb_gmnllrta_0006b07.t7<br>sb_gmnllrta_0007g21.t7	LSU rRNA; Hydrolagus colliei		2e-95	96% (200/208)	ES783171<br>FL634430
sb_gmnllrta.127.C1	2	sb_gmnllrta_0004h19.t7<br>sb_gmnllrta_0003g23.t7	Cluster: Sialate O-acetylesterase precursor (EC 3.1.1.53) (Sialic acid-specific 9-O-acetylesterase) (H-Lse).; n=1; Takifugu rubripes|Rep: Sialate O-acetylesterase precursor (EC 3.1.1.53) (Sialic acid-specific 9-O-acetylesterase) (H-Lse). - Takifugu rubripes		8e-16	45% (42/92)	ES782688<br>ES782571
sb_gmnllrta.128.C1	2	sb_gmnllrta_0001c08.t7<br>sb_gmnllrta_0007k08.t7	P8; similar to Nuclear protein 1 (Protein p8) (Candidate of metastasis 1)		5e-06	68% (22/32)	ES782638<br>FL634488
sb_gmnllrta.129.C1	2	sb_gmnllrta_0006o02.t7<br>sb_gmnllrta_0006l23.t7	Cluster: Nuclear factor interleukin-3-regulated protein; n=2; Danio rerio|Rep: Nuclear factor interleukin-3-regulated protein - Danio rerio (Zebrafish)		2e-08	73% (31/42)	ES782343<br>ES782646

			(Brachydanio rerio)				
sb_gmnllrta.130.C1	2	sb_gmnllrta_0003n13.t7<br>sb_gmnllrta_0002d08.t7	Cluster: Hemoglobin subunit alpha-2; n=3; Gadidae|Rep: Hemoglobin subunit alpha-2 - Gadus morhua (Atlantic cod)	GO:0005344<br>GO:0005506<br>GO:0005833<br>GO:0006810<br>GO:0015671<br>GO:0019825<br>GO:0020037	2e-12	96% (32/33)	ES783022<br>ES783121
sb_gmnllrta.131.C1	2	sb_gmnllrta_0006c11.t7<br>sb_gmnllrta_0006g07.t7	Cluster: Transgelin (Smooth muscle protein 22-alpha) (SM22-alpha) (WS3-10) (22 kDa actin-binding protein).; n=1; Takifugu rubripes|Rep: Transgelin (Smooth muscle protein 22-alpha) (SM22-alpha) (WS3-10) (22 kDa actin-binding protein). - Takifugu rubripes		8e-07	62% (23/37)	ES783032<br>ES782827
sb_gmnllrta.133.C1	2	sb_gmnllrta_0003m07.t7<br>sb_gmnllrta_0004c22.t7	Cluster: Apolipoprotein B; n=1; Salmo salar|Rep: Apolipoprotein B - Salmo salar (Atlantic salmon)		7e-17	59% (29/49)	ES782908<br>ES782143
sb_gmnllrta.134.C1	2	sb_gmnllrta_0006f23.t7<br>sb_gmnllrta_0006n12.t7	unclassified				ES782061<br>ES782420
sb_gmnllrta.137.C1	2	sb_gmnllrta_0007f23.t7<br>sb_gmnllrta_0007m03.t7	Cluster: Alcohol dehydrogenase 1; n=1; Gadus callarias|Rep: Alcohol dehydrogenase 1 - Gadus callarias (Baltic cod)	GO:0003824<br>GO:0004022<br>GO:0005488<br>GO:0005737<br>GO:0008152<br>GO:0008270<br>GO:0016491	8e-68	100% (121/121)	FL634413<br>FL634516
sb_gmnllrta.138.C1	2	sb_gmnllrta_0007f17.t7<br>sb_gmnllrta_0004i09.t7	Cluster: Alanine-glyoxylate aminotransferase; n=1; Platichthys flesus|Rep: Alanine-glyoxylate aminotransferase - Platichthys flesus (European flounder)	GO:0003824<br>GO:0004760<br>GO:0008152<br>GO:0008483<br>GO:0016740	5e-45	86% (85/98)	FL634409<br>ES782767
sb_gmnllrta.14.C1	2	sb_gmnllrta_0002i14.t7<br>sb_gmnllrta_0005j17.t7	unclassified				ES783052<br>ES782864
sb_gmnllrta.142.C1	2	sb_gmnllrta_0005p13.t7<br>sb_gmnllrta_0005o18.t7	unclassified				ES782212<br>ES782912

sb_gmnllrta.143.C1	2	sb_gmnllrta_0006i09.t7<br>sb_gmnllrta_0006f08.t7	Cluster: Chaperonin containing TCP1, subunit 3; n=2; Danio rerio|Rep: Chaperonin containing TCP1, subunit 3 - Danio rerio (Zebrafish) (Brachydanio rerio)		1e-66	93% (125/134)	ES782977<br>ES782033
sb_gmnllrta.144.C1	2	sb_gmnllrta_0005l09.t7<br>sb_gmnllrta_0003p16.t7	unclassified				ES783206<br>ES783243
sb_gmnllrta.147.C1	2	sb_gmnllrta_0007i15.t7<br>sb_gmnllrta_0007c15.t7	unclassified				FL634459<br>FL634362
sb_gmnllrta.148.C1	2	sb_gmnllrta_0005j18.t7<br>sb_gmnllrta_0005e22.t7	Cluster: Heat shock protein 94c.; n=1; Takifugu rubripes|Rep: Heat shock protein 94c. - Takifugu rubripes		1e-47	86% (93/108)	ES783257<br>ES782970
sb_gmnllrta.15.C1	2	sb_gmnllrta_0007e14.t7<br>sb_gmnllrta_0006c01.t7	Cluster: PREDICTED: similar to ribosomal protein L10a; n=1; Pan troglodytes|Rep: PREDICTED: similar to ribosomal protein L10a - Pan troglodytes		3e-34	71% (73/102)	FL634394<br>ES782158
sb_gmnllrta.150.C1	2	sb_gmnllrta_0006e06.t7<br>sb_gmnllrta_0006l15.t7	Cluster: Zgc:92599; n=1; Danio rerio|Rep: Zgc:92599 - Danio rerio (Zebrafish) (Brachydanio rerio)		1e-31	55% (66/119)	ES783215<br>ES782215
sb_gmnllrta.153.C1	2	sb_gmnllrta_0006c06.t7<br>sb_gmnllrta_0006m03.t7	Cluster: Glutathione S-transferase pi; n=2; Cyprinidae|Rep: Glutathione S-transferase pi - Danio rerio (Zebrafish) (Brachydanio rerio)		2e-14	75% (37/49)	ES782000<br>ES782115
sb_gmnllrta.155.C1	2	sb_gmnllrta_0003c12.t7<br>sb_gmnllrta_0004h23.t7	Cluster: Ribonucleoside-diphosphate reductase large subunit (EC 1.17.4.1) (Ribonucleoside-diphosphate reductase M1 subunit) (Ribonucleotide reductase large chain).; n=1; Takifugu rubripes|Rep: Ribonucleoside-diphosphate reductase large subunit (EC 1.17.4.1) (Ribonucleoside-diphosphate reductase M1 subunit) (Ribonucleotide reductase large chain). - Takifugu rubripes		3e-26	82% (34/41)	ES782446<br>ES782596
sb_gmnllrta.157.C1	2	sb_gmnllrta_0001h08.t7<br>sb_gmnllrta_0001c04.t7	Cluster: Eukaryotic elongation factor 1 alpha; n=1; Gadus morhua|Rep: Eukaryotic elongation factor 1 alpha -		8e-54	98% (73/74)	ES783165<br>ES783078

			Gadus morhua (Atlantic cod)				
sb_gmnllrta.158.C1	2	sb_gmnllrta_0002p23.t7<br>sb_gmnllrta_0004i19.t7	Cluster: Novel protein similar to human NADH dehydrogenase (Ubiquinone) 1 beta subcomplex, 11, 17.3kDa; n=1; Danio rerio|Rep: Novel protein similar to human NADH dehydrogenase (Ubiquinone) 1 beta subcomplex, 11, 17.3kDa - Danio rerio (Zebrafish) (Brachydanio rerio)		7e-29	67% (56/83)	ES782506<br>ES782529
sb_gmnllrta.161.C1	2	sb_gmnllrta_0007h06.t7<br>sb_gmnllrta_0007j09.t7	Cluster: Beta2-microglobulin precursor; n=3; Gadus morhua|Rep: Beta2-microglobulin precursor - Gadus morhua (Atlantic cod)		2e-31	100% (63/63)	FL634437<br>FL634469
sb_gmnllrta.162.C1	2	sb_gmnllrta_0006e08.t7<br>sb_gmnllrta_0006k15.t7	Unassigned protein				ES782881<br>ES782290
sb_gmnllrta.163.C1	2	sb_gmnllrta_0003k19.t7<br>sb_gmnllrta_0002l20.t7	unclassified				ES783252<br>ES782176
sb_gmnllrta.165.C1	2	sb_gmnllrta_0006a01.t7<br>sb_gmnllrta_0005h22.t7	unclassified				ES782559<br>ES782428
sb_gmnllrta.167.C1	2	sb_gmnllrta_0005l01.t7<br>sb_gmnllrta_0005h07.t7	DJ-1_PfpI domain containing protein		9e-14	30% (39/129)	ES782741<br>ES782085
sb_gmnllrta.168.C1	2	sb_gmnllrta_0006h08.t7<br>sb_gmnllrta_0006i11.t7	Cluster: Alpha-1-microglobulin/bikunin; n=1; Oncorhynchus mykiss|Rep: Alpha-1-microglobulin/bikunin - Oncorhynchus mykiss (Rainbow trout) (Salmo gairdneri)	GO:0004867<br>GO:0005215<br>GO:0005488	6e-05	55% (21/38)	ES782218<br>ES782421
sb_gmnllrta.169.C1	2	sb_gmnllrta_0004h21.t7<br>sb_gmnllrta_0007k14.t7	LSU rRNA; Neoceratodus forsteri		7e-58	91% (156/170)	ES782471<br>FL634494
sb_gmnllrta.170.C1	2	sb_gmnllrta_0004n07.t7<br>sb_gmnllrta_0005j07.t7	unclassified				ES782994<br>ES782306

sb_gmnllrta.171.C1	2	sb_gmnllrta_0003e08.t7<br>sb_gmnllrta_0003j08.t7	ATP1G1_PLM_MAT8 domain containing protein		1e-05	29% (18/61)	ES782228<br>ES783122
sb_gmnllrta.172.C1	2	sb_gmnllrta_0006o13.t7<br>sb_gmnllrta_0002f10.t7	Cluster: ADP-ribosylation factor 6; n=30; Coelomata|Rep: ADP-ribosylation factor 6 - Homo sapiens (Human)		2e-12	66% (44/66)	ES782885<br>ES782234
sb_gmnllrta.173.C1	2	sb_gmnllrta_0002f17.t7<br>sb_gmnllrta_0005h21.t7	unclassified				ES782069<br>ES782368
sb_gmnllrta.174.C1	2	sb_gmnllrta_0007p22.t7<br>sb_gmnllrta_0005d16.t7	unclassified				FL634579<br>ES782699
sb_gmnllrta.175.C1	2	sb_gmnllrta_0006h07.t7<br>sb_gmnllrta_0007i12.t7	unclassified				ES782479<br>FL634457
sb_gmnllrta.176.C1	2	sb_gmnllrta_0007f15.t7<br>sb_gmnllrta_0007k16.t7	unclassified				FL634407<br>FL634495
sb_gmnllrta.177.C1	2	sb_gmnllrta_0003o19.t7<br>sb_gmnllrta_0007b18.t7	unclassified				ES782424<br>FL634346
sb_gmnllrta.178.C1	2	sb_gmnllrta_0005g04.t7<br>sb_gmnllrta_0006h01.t7	Cluster: Novel protein similar to vertebrate adaptor-related protein complex 2, mu 1 subunit; n=3; Euteleostomi|Rep: Novel protein similar to vertebrate adaptor-related protein complex 2, mu 1 subunit - Danio rerio (Zebrafish) (Brachydanio rerio)		1e-12	89% (35/39)	ES782996<br>ES782345
sb_gmnllrta.179.C1	2	sb_gmnllrta_0001h04.t7<br>sb_gmnllrta_0001g07.t7	unclassified				ES782878<br>ES782503
sb_gmnllrta.18.C1	2	sb_gmnllrta_0007g10.t7	unclassified				FL634420<br>ES782520

		sb_gmnllrta_0002d13.t7					
sb_gmnllrta.20.C1	2	sb_gmnllrta_0002j12.t7<br>sb_gmnllrta_0007a22.t7	unclassified				ES782360<br>FL634332
sb_gmnllrta.21.C1	2	sb_gmnllrta_0006m24.t7<br>sb_gmnllrta_0006o12.t7	unclassified				ES782325<br>ES782903
sb_gmnllrta.26.C1	2	sb_gmnllrta_0003h16.t7<br>sb_gmnllrta_0006l01.t7	unclassified				ES782010<br>ES783168
sb_gmnllrta.27.C1	2	sb_gmnllrta_0003p18.t7<br>sb_gmnllrta_0007j04.t7	Cluster: Signal peptidase complex subunit 3; n=6; Amniota|Rep: Signal peptidase complex subunit 3 - Gallus gallus (Chicken)		4e-24	79% (49/62)	ES782934<br>FL634467
sb_gmnllrta.28.C1	2	sb_gmnllrta_0006o04.t7<br>sb_gmnllrta_0006g11.t7	unclassified				ES782481<br>ES782564
sb_gmnllrta.30.C1	2	sb_gmnllrta_0006d02.t7<br>sb_gmnllrta_0006n09.t7	Cluster: Rhamnose binding lectin STL2; n=1; Oncorhynchus mykiss|Rep: Rhamnose binding lectin STL2 - Oncorhynchus mykiss (Rainbow trout) (Salmo gairdneri)	GO:0005529<br>GO:0016020	1e-13	50% (34/67)	ES782155<br>ES783029
sb_gmnllrta.31.C1	2	sb_gmnllrta_0007o08.t7<br>sb_gmnllrta_0003i04.t7	Cluster: Histamine N-methyltransferase (EC 2.1.1.8) (HMT).; n=1; Takifugu rubripes|Rep: Histamine N-methyltransferase (EC 2.1.1.8) (HMT). - Takifugu rubripes		5e-38	49% (74/149)	FL634554<br>ES782042
sb_gmnllrta.34.C1	2	sb_gmnllrta_0004f07.t7<br>sb_gmnllrta_0005k09.t7	unclassified				ES782086<br>ES783117
sb_gmnllrta.39.C1	2	sb_gmnllrta_0005j15.t7<br>sb_gmnllrta_0004n06.t7	unclassified				ES782839<br>ES782968

sb_gmnllrta.4.C1	2	sb_gmnllrta_0003k06.t7<br>sb_gmnllrta_0007o21.t7	unclassified				ES782314<br>FL634566
sb_gmnllrta.41.C1	2	sb_gmnllrta_0003m20.t7<br>sb_gmnllrta_0003g24.t7	unclassified				ES783154<br>ES782414
sb_gmnllrta.43.C1	2	sb_gmnllrta_0005f06.t7<br>sb_gmnllrta_0005k22.t7	unclassified				ES782242<br>ES783152
sb_gmnllrta.46.C1	2	sb_gmnllrta_0006e16.t7<br>sb_gmnllrta_0006h05.t7	unclassified				ES782034<br>ES782621
sb_gmnllrta.48.C1	2	sb_gmnllrta_0005e14.t7<br>sb_gmnllrta_0006h15.t7	Cluster: Proteasome activator subunit 2; n=2; Clupeocephala|Rep: Proteasome activator subunit 2 - Danio rerio (Zebrafish) (Brachydanio rerio)		5e-12	80% (32/40)	ES782519<br>ES782694
sb_gmnllrta.49.C1	2	sb_gmnllrta_0005a20.t7<br>sb_gmnllrta_0007e17.t7	Cluster: MGC80906 protein; n=1; Xenopus laevis|Rep: MGC80906 protein - Xenopus laevis (African clawed frog)		1e-21	83% (46/55)	ES782593<br>FL634396
sb_gmnllrta.52.C1	2	sb_gmnllrta_0006f12.t7<br>sb_gmnllrta_0006o23.t7	Cluster: Cell division cycle 20 homolog; n=3; Danio rerio|Rep: Cell division cycle 20 homolog - Danio rerio (Zebrafish) (Brachydanio rerio)		9e-59	66% (94/142)	ES782636<br>ES782268
sb_gmnllrta.54.C1	2	sb_gmnllrta_0005m16.t7<br>sb_gmnllrta_0002h12.t7	unclassified				ES782836<br>ES782126
sb_gmnllrta.55.C1	2	sb_gmnllrta_0007j10.t7<br>sb_gmnllrta_0007k05.t7	unclassified				FL634470<br>FL634487
sb_gmnllrta.58.C1	2	sb_gmnllrta_0006d07.t7<br>sb_gmnllrta_0005l14.t7	unclassified				ES782133<br>ES782386

sb_gmnllrta.59.C1	2	sb_gmnllrta_0007f03.t7<br>sb_gmnllrta_0006e05.t7	Cluster: Ribosomal protein L18; n=5; Euteleostomi|Rep: Ribosomal protein L18 - Pagrus major (Red sea bream) (Chrysophrys major)	GO:0003735<br>GO:0005622<br>GO:0005840<br>GO:0006412	8e-33	82%<br>(66/80)	FL634401<br>ES783108
sb_gmnllrta.63.C1	2	sb_gmnllrta_0004l20.t7<br>sb_gmnllrta_0007d06.t7	Cluster: MHC class Ia antigen; n=6; Gadus morhua|Rep: MHC class Ia antigen - Gadus morhua (Atlantic cod)	GO:0006955<br>GO:0016020<br>GO:0019882	2e-55	80%<br>(105/130)	ES782985<br>FL634372
sb_gmnllrta.64.C1	2	sb_gmnllrta_0007m08.t7<br>sb_gmnllrta_0007a16.t7	Cluster: Coiled-coil domain-containing protein 111.; n=1; Takifugu rubripes|Rep: Coiled-coil domain-containing protein 111. - Takifugu rubripes		3e-59	54%<br>(122/222)	FL634520<br>FL634329
sb_gmnllrta.66.C1	2	sb_gmnllrta_0002g13.t7<br>sb_gmnllrta_0006g16.t7	unclassified				ES783076<br>ES782402
sb_gmnllrta.69.C1	2	sb_gmnllrta_0006k06.t7<br>sb_gmnllrta_0007i11.t7	Ig heavy chain, secreted form - Atlantic cod emb|CAA41680.1| immunoglobulin heavy chain secretory form [Gadus morhua]		7e-71	92%<br>(130/140)	ES783083<br>FL634456
sb_gmnllrta.73.C1	2	sb_gmnllrta_0007d07.t7<br>sb_gmnllrta_0007f24.t7	unclassified				FL634373<br>FL634414
sb_gmnllrta.76.C1	2	sb_gmnllrta_0003d01.t7<br>sb_gmnllrta_0002k15.t7	Cluster: Cell surface protein; n=1; Lactobacillus plantarum|Rep: Cell surface protein - Lactobacillus plantarum	GO:0005618	5e-06	46%<br>(34/73)	ES782853<br>ES782671
sb_gmnllrta.79.C1	2	sb_gmnllrta_0006d05.t7<br>sb_gmnllrta_0006o16.t7	unclassified				ES781998<br>ES782693
sb_gmnllrta.80.C1	2	sb_gmnllrta_0007h09.t7<br>sb_gmnllrta_0007j12.t7	Cluster: Ribosomal protein S8; n=22; Euteleostomi|Rep: Ribosomal protein S8 - Mus musculus (Mouse)		3e-32	97%<br>(65/67)	FL634440<br>FL634472
sb_gmnllrta.81.C1	2	sb_gmnllrta_0005h19.t7<br>sb_gmnllrta_0003i07.t7	Cluster: Poly(RC) binding protein 3; n=3; Tetrapoda|Rep: Poly(RC) binding protein 3 - Xenopus tropicalis (Western clawed		5e-19	71%<br>(47/66)	ES782783<br>ES782145

			frog) (Silurana tropicalis)				
sb_gmnllrta.83.C1	2	sb_gmnllrta_0007p19.t7<br>sb_gmnllrta_0007a08.t7	Unassigned protein				FL634577<br>FL634323
sb_gmnllrta.84.C1	2	sb_gmnllrta_0007d04.t7<br>sb_gmnllrta_0007n01.t7	unclassified				FL634371<br>FL634535
sb_gmnllrta.85.C1	2	sb_gmnllrta_0004h14.t7<br>sb_gmnllrta_0002e21.t7	unclassified				ES783073<br>ES782087
sb_gmnllrta.86.C1	2	sb_gmnllrta_0007j13.t7<br>sb_gmnllrta_0004m10.t7	Cluster: PREDICTED: similar to dynein, cytoplasmic, heavy polypeptide 1; n=2; Amniota|Rep: PREDICTED: similar to dynein, cytoplasmic, heavy polypeptide 1 - Gallus gallus		4e-06	61%<br>(26/42)	FL634473<br>ES782831
sb_gmnllrta.87.C1	2	sb_gmnllrta_0006d04.t7<br>sb_gmnllrta_0007l14.t7	Cluster: FACT complex subunit SSRP1 (Facilitates chromatin transcription complex subunit SSRP1) (FACT 80 kDa subunit) (FACTp80) (Chromatin- specific transcription elongation factor 80 kDa subunit) (Structure- specific recognition protein 1) (hSSRP1) (Recombination; n=1; Takifugu rubripes|Rep: FACT complex subunit SSRP1 (Facilitates chromatin transcription complex subunit SSRP1) (FACT 80 kDa subunit) (FACTp80) (Chromatin- specific transcription elongation factor 80 kDa subunit) (Structure- specific recognition protein 1) (hSSRP1) (Recombination - Takifugu rubripes		9e-21	90%<br>(47/52)	ES782065<br>FL634508
sb_gmnllrta.90.C1	2	sb_gmnllrta_0003d03.t7<br>sb_gmnllrta_0006d12.t7	Cluster: High mobility group-T protein; n=5; Salmonidae|Rep: High mobility group-T protein - Oncorhynchus mykiss (Rainbow trout) (Salmo gairdneri)	GO:0000785<br>GO:0003677<br>GO:0005634<br>GO:0005694	6e-53	78%<br>(66/84)	ES782889<br>ES783030
sb_gmnllrta.91.C1	2	sb_gmnllrta_0003p21.t7<br>sb_gmnllrta_0007a18.t7	Cluster: Lectin; n=1; Oncorhynchus mykiss|Rep: Lectin - Oncorhynchus mykiss (Rainbow trout) (Salmo gairdneri)		5e-14	48%<br>(25/52)	ES782456<br>FL634331
sb_gmnllrta.92.C1	2	sb_gmnllrta_0004f16.t7<br>sb_gmnllrta_0006h19.t7	Cluster: Eukaryotic elongation factor 1 alpha; n=1; Gadus morhua|Rep: Eukaryotic elongation factor 1 alpha -		6e-13	97%<br>(34/35)	ES783187<br>ES783061

			Gadus morhua (Atlantic cod)				
sb_gmnllrta.93.C1	2	sb_gmnllrta_0006m04.t7<br>sb_gmnllrta_0006m13.t7	Cluster: Rab effector MyRIP (Myosin-VIIa- and Rab-interacting protein) (Exophilin-8) (Slp homolog lacking C2 domains c) (SlaC2-c).; n=1; Danio rerio|Rep: Rab effector MyRIP (Myosin-VIIa- and Rab-interacting protein) (Exophilin-8) (Slp homolog lacking C2 domains c) (SlaC2-c). - Danio rerio		3e-32	43% (98/226)	ES782265<br>ES783145
sb_gmnllrta.94.C1	2	sb_gmnllrta_0003l15.t7<br>sb_gmnllrta_0007f11.t7	unclassified				ES782869<br>FL634405
sb_gmnllrta.95.C1	2	sb_gmnllrta_0003i08.t7<br>sb_gmnllrta_0005l12.t7	ptgs1; prostaglandin-endoperoxide synthase 1 [EC:1.14.99.1]; K00509 prostaglandin-endoperoxide synthase	GO:0004601<br>GO:0006118<br>GO:0006979	5e-14	60% (28/46)	ES782389<br>ES782604
sb_gmnllrta.98.C1	2	sb_gmnllrta_0007m05.t7<br>sb_gmnllrta_0006l17.t7	unclassified				FL634517<br>ES782236
sb_gmnllrta.99.C1	2	sb_gmnllrta_0003i20.t7<br>sb_gmnllrta_0007m07.t7	Cluster: Fibrinogen, B beta polypeptide; n=2; Danio rerio|Rep: Fibrinogen, B beta polypeptide - Danio rerio (Zebrafish) (Brachydanio rerio)		9e-07	75% (21/28)	ES782375<br>FL634519
sb_gmnllrta_0001a01.t7	1		MGC81978; MGC81978 protein [EC:2.3.1.48]; K00653 histone acetyltransferase		4e-48	67% (90/134)	ES782876
sb_gmnllrta_0001a02.t7	1		Cluster: PREDICTED: similar to Protein tyrosine phosphatase, receptor type, B; n=2; Gallus gallus|Rep: PREDICTED: similar to Protein tyrosine phosphatase, receptor type, B - Gallus gallus		6e-45	63% (89/141)	ES782803
sb_gmnllrta_0001a03.t7	1		Cluster: Homolog of Homo sapiens "Adaptor-related protein complex 1, mu 1 subunit; n=1; Takifugu rubripes|Rep: Homolog of Homo sapiens "Adaptor-related protein complex 1, mu 1 subunit - Takifugu rubripes		9e-26	83% (54/65)	ES782826

sb_gmnllrta_0001a04.t7	1		unclassified				ES782660
sb_gmnllrta_0001a05.t7	1		unclassified				ES782645
sb_gmnllrta_0001a06.t7	1		Cluster: Elongation factor 1-gamma; n=7; Clupeocephala|Rep: Elongation factor 1-gamma - Brachydanio rerio (Zebrafish) (Danio rerio)		1e-42	83% (51/61)	ES782715
sb_gmnllrta_0001a07.t7	1		unclassified				ES782772
sb_gmnllrta_0001a08.t7	1		unclassified				ES783033
sb_gmnllrta_0001a09.t7	1		UPF0005 domain containing protein	GO:0016021 GO:0043066	3e-07	30% (15/50)	ES783084
sb_gmnllrta_0001a10.t7	1		Cluster: Ribophorin I; n=2; Danio rerio|Rep: Ribophorin I - Brachydanio rerio (Zebrafish) (Danio rerio)		7e-42	83% (78/93)	ES782379
sb_gmnllrta_0001b02.t7	1		Cluster: similar to splicing factor, arginine/serine-rich 2 (Sfrs2), mRNA; n=1; Rattus norvegicus|Rep: similar to splicing factor, arginine/serine-rich 2 (Sfrs2), mRNA - Rattus norvegicus		3e-07	61% (21/34)	ES782111
sb_gmnllrta_0001b05.t7	1		unclassified				ES782269
sb_gmnllrta_0001b06.t7	1		Cluster: Zgc:112282; n=2; Danio rerio|Rep: Zgc:112282 - Brachydanio rerio (Zebrafish) (Danio rerio)		1e-52	81% (103/127)	ES782159
sb_gmnllrta_0001c03.t7	1		unclassified				ES783239

sb_gmnllrta_0001c05.t7	1		Cluster: Receptor of Activated Kinase C 1; n=1; Mya arenaria|Rep: Receptor of Activated Kinase C 1 - Mya arenaria	GO:0004872 GO:0016301	4e-29	80% (32/40)	ES783067
sb_gmnllrta_0001c07.t7	1		Cluster: Myeloperoxidase; n=1; Siniperca chuatsi|Rep: Myeloperoxidase - Siniperca chuatsi (Chinese perch)	GO:0004601 GO:0046872	7e-14	55% (37/67)	ES783008
sb_gmnllrta_0001c09.t7	1		unclassified				ES782689
sb_gmnllrta_0001d02.t7	1		unclassified				ES783106
sb_gmnllrta_0001d05.t7	1		Cluster: PREDICTED: similar to Myosin regulatory light chain 2, smooth muscle isoform (Myosin RLC) (LC20); n=1; Canis familiaris|Rep: PREDICTED: similar to Myosin regulatory light chain 2, smooth muscle isoform (Myosin RLC) (LC20) - Canis familiaris		3e-33	91% (66/72)	ES782944
sb_gmnllrta_0001d08.t7	1		unclassified				ES782718
sb_gmnllrta_0001d09.t7	1		Cluster: Cathepsin D precursor; n=1; Chionodraco hamatus|Rep: Cathepsin D precursor - Chionodraco hamatus (Antarctic teleost icefish)	GO:0004190 GO:0004192 GO:0004194 GO:0005575 GO:0005764 GO:0006508 GO:0008233 GO:0016787	3e-52	89% (95/106)	ES782736
sb_gmnllrta_0001e01.t7	1		Cluster: Homolog of Brachydanio rerio "Hydroxyacyl glutathione hydrolase (Zgc:73161 protein).; n=1; Takifugu rubripes|Rep: Homolog of Brachydanio rerio "Hydroxyacyl glutathione hydrolase (Zgc:73161 protein). - Takifugu rubripes		2e-43	85% (78/91)	ES782107
sb_gmnllrta_0001e03.t7	1		LOC584434; similar to Hect domain and		1e-10	43%	ES782064

			RLD 4			(30/69)	
sb_gmnllrta_0001e06.t7	1		Unassigned protein				ES782266
sb_gmnllrta_0001e07.t7	1		Unassigned protein				ES782281
sb_gmnllrta_0001e08.t7	1		Cluster: Hepcidin; n=1; Pseudosciaena crocea|Rep: Hepcidin - Pseudosciaena crocea (Croceine croaker)		9e-25	54% (53/98)	ES782565
sb_gmnllrta_0001e09.t7	1		Cluster: PREDICTED: similar to ATP-binding cassette, sub-family D (ALD), member 3a; n=2; Danio rerio|Rep: PREDICTED: similar to ATP-binding cassette, sub-family D (ALD), member 3a - Danio rerio		2e-48	96% (52/54)	ES782582
sb_gmnllrta_0001f02.t7	1		unclassified				ES782875
sb_gmnllrta_0001f03.t7	1		unclassified				ES782938
sb_gmnllrta_0001f04.t7	1		Cluster: Mantle gene 8; n=1; Pinctada fucata|Rep: Mantle gene 8 - Pinctada fucata (Pearl oyster)		6e-06	51% (20/39)	ES782775
sb_gmnllrta_0001f05.t7	1		Cluster: Integral membrane protein 2B (Transmembrane protein BRI) [Contains: ABri/ADan amyloid peptide]; n=19; Eutheria|Rep: Integral membrane protein 2B (Transmembrane protein BRI) [Contains: ABri/ADan amyloid peptide] - Homo sapiens (Human)		6e-05	88% (16/18)	ES782713
sb_gmnllrta_0001f07.t7	1		Cluster: Chaperone protein GP96 (Tumor rejection antigen (Gp96) 1) (Heat shock protein 90kDa beta (Grp94), member 1); n=2; Clupeocephala|Rep: Chaperone protein GP96 (Tumor rejection antigen (Gp96) 1) (Heat shock protein 90kDa beta (Grp94), member 1) - Brachydanio rerio		8e-56	80% (103/128)	ES782662

			(Zebrafish) (Danio rerio)				
sb_gmnllrta_0001f08.t7	1		unclassified				ES782979
sb_gmnllrta_0001f09.t7	1		Cluster: Titin; n=4; Homo sapiens|Rep: Titin - Homo sapiens (Human)		3e-75	95% (102/107)	ES783003
sb_gmnllrta_0001f10.t7	1		unclassified				ES782423
sb_gmnllrta_0001g02.t7	1		Cluster: Zgc:66080; n=2; Danio rerio|Rep: Zgc:66080 - Brachydanio rerio (Zebrafish) (Danio rerio)		2e-19	83% (44/53)	ES782396
sb_gmnllrta_0001g03.t7	1		unclassified				ES782458
sb_gmnllrta_0001g09.t7	1		unclassified				ES782213
sb_gmnllrta_0001g10.t7	1		Cluster: Ubiquitin/60S ribosomal fusion protein, putative; n=5; root|Rep: Ubiquitin/60S ribosomal fusion protein, putative - Theileria annulata	GO:0003735 GO:0005622 GO:0005840 GO:0006412 GO:0006464	9e-28	98% (50/51)	ES782907
sb_gmnllrta_0001g11.t7	1		Cluster: Homolog of Paralichthys olivaceus "Complement component C3.; n=1; Takifugu rubripes|Rep: Homolog of Paralichthys olivaceus "Complement component C3. - Takifugu rubripes		6e-26	48% (64/132)	ES782882
sb_gmnllrta_0001g12.t7	1		Cluster: Homolog of Homo sapiens "Maltase-glucoamylase, intestinal; n=1; Takifugu rubripes|Rep: Homolog of Homo sapiens "Maltase-glucoamylase, intestinal - Takifugu rubripes		5e-17	44% (45/102)	ES782797
sb_gmnllrta_0001h01.t7	1		unclassified				ES782667

sb_gmnllrta_0001h02.t7	1		unclassified				ES782779
sb_gmnllrta_0001h03.t7	1		Unassigned protein				ES782708
sb_gmnllrta_0001h06.t7	1		unclassified				ES782825
sb_gmnllrta_0001h10.t7	1		Cluster: Hemoglobin subunit beta-2; n=3; Gadidae|Rep: Hemoglobin subunit beta-2 - Gadus morhua (Atlantic cod)	GO:0005344 GO:0005506 GO:0005833 GO:0006810 GO:0015671 GO:0019825 GO:0020037 GO:0046872	3e-09	80% (32/40)	ES782561
sb_gmnllrta_0001h11.t7	1		unclassified				ES782586
sb_gmnllrta_0001h12.t7	1		Cluster: 60S ribosomal protein L9; n=4; Euteleostomi|Rep: 60S ribosomal protein L9 - Ictalurus punctatus (Channel catfish)	GO:0003735 GO:0005622 GO:0005840 GO:0006412 GO:0030529	5e-15	93% (27/29)	ES782537
sb_gmnllrta_0002a14.t7	1		allergen V5/TPX-1 related		2e-06	50% (33/65)	ES782066
sb_gmnllrta_0002b06.t7	1		unclassified				ES782417
sb_gmnllrta_0002b13.t7	1		unclassified				ES782763
sb_gmnllrta_0002b14.t7	1		unclassified				ES782924

sb_gmnllrta_0002b16.t7	1		Cluster: 40S ribosomal protein S21; n=5; Euteleostomi|Rep: 40S ribosomal protein S21 - Ictalurus punctatus (Channel catfish)	GO:0003735<br>GO:0005622<br>GO:0005840<br>GO:0006412<br>GO:0030529	4e-33	90% (68/75)	ES782818
sb_gmnllrta_0002b18.t7	1		unclassified				ES783138
sb_gmnllrta_0002b22.t7	1		unclassified				ES782089
sb_gmnllrta_0002c06.t7	1		unclassified				ES782902
sb_gmnllrta_0002c08.t7	1		unclassified				ES783214
sb_gmnllrta_0002c17.t7	1		Cluster: Cytochrome P450 3A; n=1; Dicentrarchus labrax|Rep: Cytochrome P450 3A - Dicentrarchus labrax (European sea bass)	GO:0004497<br>GO:0005506<br>GO:0006118<br>GO:0016712<br>GO:0020037	7e-98	73% (173/234)	ES782349
sb_gmnllrta_0002c19.t7	1		Cluster: Alpha tubulin; n=6; Bilateria|Rep: Alpha tubulin - Notothenia coriiceps (black rockcod)	GO:0003924<br>GO:0005198<br>GO:0005525<br>GO:0005874<br>GO:0007017<br>GO:0007018<br>GO:0043234<br>GO:0051258	2e-20	83% (41/49)	ES782040
sb_gmnllrta_0002c20.t7	1		Cluster: Homolog of Gallus gallus "Ig lambda chain C region.; n=4; Takifugu rubripes|Rep: Homolog of Gallus gallus "Ig lambda chain C region. - Takifugu rubripes		1e-28	56% (66/117)	ES783153
sb_gmnllrta_0002d10.t7	1		unclassified				ES782614

sb_gmnllrta_0002d14.t7	1		unclassified				ES782352
sb_gmnllrta_0002d16.t7	1		STMN1; stathmin 1/oncoprotein 18		6e-43	83% (89/106)	ES782393
sb_gmnllrta_0002d20.t7	1		unclassified				ES783259
sb_gmnllrta_0002e07.t7	1		Cluster: PREDICTED: similar to RB1-inducible coiled-coil 1; n=1; Monodelphis domestica|Rep: PREDICTED: similar to RB1-inducible coiled-coil 1 - Monodelphis domestica		1e-61	73% (122/165)	ES782367
sb_gmnllrta_0002e09.t7	1		unclassified				ES782067
sb_gmnllrta_0002e18.t7	1		MGC86432; MGC86432 protein [EC:1.9.3.1]; K02271 cytochrome c oxidase subunit VIIb	GO:0004129 GO:0005746 GO:0006118	5e-13	57% (28/49)	ES783198
sb_gmnllrta_0002e20.t7	1		unclassified				ES782151
sb_gmnllrta_0002e24.t7	1		unclassified				ES782200
sb_gmnllrta_0002f02.t7	1		unclassified				ES782961
sb_gmnllrta_0002f03.t7	1		unclassified				ES782999
sb_gmnllrta_0002f13.t7	1		Unassigned protein				ES782278

sb_gmnllrta_0002f14.t7	1		unclassified				ES782119
sb_gmnllrta_0002f24.t7	1		unclassified				ES782748
sb_gmnllrta_0002g03.t7	1		Unassigned protein				ES782207
sb_gmnllrta_0002g10.t7	1		Cluster: 60S ribosomal protein L22; n=2; Holacanthopterygii|Rep: 60S ribosomal protein L22 - Gadus morhua (Atlantic cod)	GO:0003723 GO:0003735 GO:0005622 GO:0005840 GO:0006412 GO:0030529	1e-21	97% (38/39)	ES783023
sb_gmnllrta_0002g12.t7	1		unclassified				ES783046
sb_gmnllrta_0002g17.t7	1		unclassified				ES783178
sb_gmnllrta_0002g19.t7	1		MGC52584; similar to protein tyrosine phosphatase, non-receptor type 9 [EC:3.1.3.48]; K01104 protein-tyrosine phosphatase		1e-74	81% (140/171)	ES782844
sb_gmnllrta_0002g21.t7	1		Gal-bind_lectin domain containing protein		6e-06	29% (17/58)	ES782305
sb_gmnllrta_0002h02.t7	1		unclassified				ES783162
sb_gmnllrta_0002h03.t7	1		unclassified				ES783126
sb_gmnllrta_0002h04.t7	1		unclassified				ES782972

sb_gmnllrta_0002h11.t7	1		unclassified				ES782077
sb_gmnllrta_0002h15.t7	1		unclassified				ES782274
sb_gmnllrta_0002h18.t7	1		DUF850 domain containing protein		1e-42	64% (68/105)	ES782525
sb_gmnllrta_0002h20.t7	1		LSU rRNA; Oncorhynchus mykiss		1e-106	95% (226/236)	ES782625
sb_gmnllrta_0002i02.t7	1		unclassified				ES782050
sb_gmnllrta_0002i04.t7	1		unclassified				ES782185
sb_gmnllrta_0002i08.t7	1		Cluster: TLR22; n=1; Takifugu rubripes|Rep: TLR22 - Fugu rubripes (Japanese pufferfish) (Takifugu rubripes)	GO:0004872 GO:0004888 GO:0005515 GO:0006954 GO:0006955 GO:0016020 GO:0016021 GO:0045087	1e-47	88% (59/67)	ES782607
sb_gmnllrta_0002i20.t7	1		Cluster: Hsp90 co-chaperone Cdc37; n=3; Tetraodontidae|Rep: Hsp90 co-chaperone Cdc37 - Tetraodon fluviatilis (Puffer fish)	GO:0000074 GO:0006457	1e-39	96% (64/66)	ES782461
sb_gmnllrta_0002i24.t7	1		Cluster: Homolog of Brachydanio rerio "Putative heparin-binding growth factor 1 (HBGF-1) (Fibroblast growth factor 1) (FGF-1) (Acidic fibroblast growth factor) (aFGF).; n=1; Takifugu rubripes|Rep: Homolog of Brachydanio rerio "Putative heparin-binding growth factor 1 (HBGF-1) (Fibroblast growth factor 1) (FGF-1) (Acidic fibroblast growth factor) (aFGF). - Takifugu rubripes		6e-16	60% (42/69)	ES782412

sb_gmnllrta_0002j01.t7	1		unclassified				ES782854
sb_gmnllrta_0002j02.t7	1		Cluster: PREDICTED: similar to ribosomal protein L26 isoform 1; n=1; Gallus gallus|Rep: PREDICTED: similar to ribosomal protein L26 isoform 1 - Gallus gallus		1e-35	94% (73/77)	ES782919
sb_gmnllrta_0002j03.t7	1		unclassified				ES782888
sb_gmnllrta_0002j08.t7	1		unclassified				ES783021
sb_gmnllrta_0002j11.t7	1		Cluster: 60S ribosomal protein L13; n=2; Clupeocephala|Rep: 60S ribosomal protein L13 - Brachydanio rerio (Zebrafish) (Danio rerio)		3e-22	91% (53/58)	ES782449
sb_gmnllrta_0002j18.t7	1		Cluster: Ghitm-prov protein; n=2; Xenopus|Rep: Ghitm-prov protein - Xenopus laevis (African clawed frog)		3e-07	63% (23/36)	ES782294
sb_gmnllrta_0002j22.t7	1		Cluster: Homolog of Brachydanio rerio "Transgelin 2 (Tagln2 protein).; n=1; Takifugu rubripes|Rep: Homolog of Brachydanio rerio "Transgelin 2 (Tagln2 protein). - Takifugu rubripes		2e-05	85% (24/28)	ES782998
sb_gmnllrta_0002k06.t7	1		unclassified				ES782523
sb_gmnllrta_0002k07.t7	1		Cluster: Zgc:63838; n=2; Danio rerio|Rep: Zgc:63838 - Brachydanio rerio (Zebrafish) (Danio rerio)		2e-27	70% (41/58)	ES782470
sb_gmnllrta_0002k10.t7	1		Cluster: Homolog of Brachydanio rerio "Phospholipase C gamma 1.; n=1; Takifugu rubripes|Rep: Homolog of Brachydanio rerio "Phospholipase C		4e-73	79% (131/164)	ES782862

			gamma 1. - Takifugu rubripes				
sb_gmnllrta_0002k12.t7	1		unclassified				ES782838
sb_gmnllrta_0002k13.t7	1		unclassified				ES782809
sb_gmnllrta_0002k22.t7	1		unclassified				ES782210
sb_gmnllrta_0002n17.t7	1		unclassified				ES783056
sb_gmnllrta_0002n19.t7	1		unclassified				ES782731
sb_gmnllrta_0002n23.t7	1		unclassified				ES782464
sb_gmnllrta_0002o10.t7	1		unclassified				ES782101
sb_gmnllrta_0002o11.t7	1		Trypsin domain containing protein		4e-10	37% (30/79)	ES782122
sb_gmnllrta_0002o13.t7	1		Cluster: Zgc:101691; n=1; Danio rerio|Rep: Zgc:101691 - Brachydanio rerio (Zebrafish) (Danio rerio)		3e-10	37% (24/64)	ES782009
sb_gmnllrta_0002o16.t7	1		unclassified				ES782271
sb_gmnllrta_0002o17.t7	1		Cluster: Protoporphyrinogen oxidase; n=4; Danio rerio|Rep: Protoporphyrinogen oxidase - Brachydanio rerio (Zebrafish) (Danio rerio)		3e-26	71% (56/78)	ES782261

sb_gmnllrta_0002o18.t7	1		unclassified				ES782589
sb_gmnllrta_0002o24.t7	1		Cluster: Ribosomal protein S14; n=5; Euteleostomi|Rep: Ribosomal protein S14 - Bos taurus (Bovine)		1e-34	100% (71/71)	ES782796
sb_gmnllrta_0002p19.t7	1		Unassigned protein				ES782771
sb_gmnllrta_0002p22.t7	1		unclassified				ES782482
sb_gmnllrta_0003a05.t7	1		Cluster: Keratin 18; n=3; Danio rerio|Rep: Keratin 18 - Brachydanio rerio (Zebrafish) (Danio rerio)		8e-08	70% (33/47)	ES783092
sb_gmnllrta_0003a06.t7	1		Cluster: PREDICTED: similar to cytoplasmic beta-actin; n=2; Homo/Pan/Gorilla group|Rep: PREDICTED: similar to cytoplasmic beta-actin - Homo sapiens		1e-05	88% (16/18)	ES782984
sb_gmnllrta_0003a13.t7	1		Cluster: Serum lectin isoform 1 precursor; n=3; Verasper variegatus|Rep: Serum lectin isoform 1 precursor - Verasper variegatus (Spotted flounder)	GO:0005529	3e-33	45% (71/157)	ES782008
sb_gmnllrta_0003a15.t7	1		Cluster: PREDICTED: similar to ribosomal protein L21; n=1; Canis familiaris|Rep: PREDICTED: similar to ribosomal protein L21 - Canis familiaris		2e-16	73% (22/30)	ES782226
sb_gmnllrta_0003a20.t7	1		unclassified				ES782725
sb_gmnllrta_0003a23.t7	1		PREDICTED: p8 protein (candidate of metastasis 1) [Macaca mulatta]		1e-09	73% (28/38)	ES782629

sb_gmnllrta_0003b01.t7	1		unclassified				ES782434
sb_gmnllrta_0003b11.t7	1		LSU rRNA; Neoceratodus forsteri		6e-48	97% (106/109)	ES782833
sb_gmnllrta_0003b19.t7	1		unclassified				ES782974
sb_gmnllrta_0003b21.t7	1		Cluster: Homolog of Brachydanio rerio "Ornithine decarboxylase antizyme inhibitor.; n=1; Takifugu rubripes|Rep: Homolog of Brachydanio rerio "Ornithine decarboxylase antizyme inhibitor. - Takifugu rubripes		3e-24	53% (42/78)	ES782204
sb_gmnllrta_0003b23.t7	1		unclassified				ES782246
sb_gmnllrta_0003c01.t7	1		unclassified				ES782915
sb_gmnllrta_0003c18.t7	1		unclassified				ES782196
sb_gmnllrta_0003d05.t7	1		Unassigned protein				ES782744
sb_gmnllrta_0003d20.t7	1		unclassified				ES783035
sb_gmnllrta_0003d21.t7	1		unclassified				ES783105
sb_gmnllrta_0003e05.t7	1		Cluster: Homolog of Homo sapiens "Inter-alpha (globulIn) InhIbItor H3; n=1; Takifugu rubripes|Rep: Homolog of Homo sapiens "Inter-alpha (globulIn) InhIbItor		3e-49	66% (100/151)	ES782598

			H3 - Takifugu rubripes				
sb_gmnllrta_0003e11.t7	1		Cluster: Homolog of Homo sapiens "vitronectin precursor; n=1; Takifugu rubripes|Rep: Homolog of Homo sapiens "vitronectin precursor - Takifugu rubripes		3e-22	73% (28/38)	ES782933
sb_gmnllrta_0003e12.t7	1		unclassified				ES782837
sb_gmnllrta_0003e13.t7	1		f2; coagulation factor II (thrombin) [EC:3.4.21.5]; K01313 coagulation factor II (thrombin)	GO:0003809 GO:0004252 GO:0005509 GO:0005576 GO:0006508 GO:0007596 GO:0016787	8e-11	79% (27/34)	ES782808
sb_gmnllrta_0003e15.t7	1		Cluster: Homolog of Homo sapiens "Gamma-glutamyltransferase-like activity 1; n=1; Takifugu rubripes|Rep: Homolog of Homo sapiens "Gamma-glutamyltransferase-like activity 1 - Takifugu rubripes		3e-21	56% (52/92)	ES782670
sb_gmnllrta_0003e21.t7	1		unclassified				ES782301
sb_gmnllrta_0003e22.t7	1		unclassified				ES782208
sb_gmnllrta_0003e23.t7	1		Cluster: PREDICTED: similar to 40S ribosomal protein S26; n=1; Rattus norvegicus|Rep: PREDICTED: similar to 40S ribosomal protein S26 - Rattus norvegicus		4e-16	97% (33/34)	ES782170
sb_gmnllrta_0003f03.t7	1		unclassified				ES783125
sb_gmnllrta_0003f10.t7	1		Cluster: PREDICTED: similar to ADP-ribosylation factor; n=2; Strongylocentrotus purpuratus|Rep: PREDICTED: similar to ADP-ribosylation		8e-18	92% (36/39)	ES782004

			factor - Strongylocentrotus purpuratus				
sb_gmnllrta_0003f11.t7	1		vtn; vitronectin		3e-25	61% (44/72)	ES782075
sb_gmnllrta_0003f18.t7	1		Cluster: Calreticulin; n=3; Oncorhynchus mykiss|Rep: Calreticulin - Oncorhynchus mykiss (Rainbow trout) (Salmo gairdneri)	GO:0005509 GO:0005529 GO:0005783 GO:0006457 GO:0008270 GO:0046872 GO:0051082	2e-37	80% (67/83)	ES782524
sb_gmnllrta_0003f22.t7	1		Cluster: Lypla3 protein; n=4; Danio rerio|Rep: Lypla3 protein - Brachydanio rerio (Zebrafish) (Danio rerio)		6e-28	48% (45/93)	ES782756
sb_gmnllrta_0003f23.t7	1		Cluster: Homolog of Oncorhynchus mykiss "Plasminogen precursor (EC 3.4.21.7).; n=1; Takifugu rubripes|Rep: Homolog of Oncorhynchus mykiss "Plasminogen precursor (EC 3.4.21.7). - Takifugu rubripes		1e-54	64% (77/120)	ES782721
sb_gmnllrta_0003g02.t7	1		unclassified				ES782049
sb_gmnllrta_0003g03.t7	1		unclassified				ES782017
sb_gmnllrta_0003g05.t7	1		unclassified				ES782198
sb_gmnllrta_0003g07.t7	1		unclassified				ES782247
sb_gmnllrta_0003g08.t7	1		unclassified				ES782609
sb_gmnllrta_0003g09.t7	1		unclassified				ES782548

sb_gmnllrta_0003g10.t7	1		Cluster: Heat shock protein 4, like; n=3; Clupeocephala|Rep: Heat shock protein 4, like - Brachydanio rerio (Zebrafish) (Danio rerio)		1e-62	75% (62/82)	ES783116
sb_gmnllrta_0003g14.t7	1		unclassified				ES783053
sb_gmnllrta_0003g15.t7	1		Cluster: Glutathione S-transferase pi; n=2; Cyprinidae|Rep: Glutathione S-transferase pi - Brachydanio rerio (Zebrafish) (Danio rerio)		2e-12	54% (40/73)	ES783068
sb_gmnllrta_0003g17.t7	1		Cluster: Carboxypeptidase N, polypeptide 1; n=2; Danio rerio|Rep: Carboxypeptidase N, polypeptide 1 - Brachydanio rerio (Zebrafish) (Danio rerio)		2e-52	84% (61/72)	ES782958
sb_gmnllrta_0003g19.t7	1		Cluster: Electron-transfer-flavoprotein, beta polypeptide; n=4; Clupeocephala|Rep: Electron-transfer-flavoprotein, beta polypeptide - Brachydanio rerio (Zebrafish) (Danio rerio)		8e-41	91% (42/46)	ES782631
sb_gmnllrta_0003h03.t7	1		Cluster: NADH dehydrogenase [ubiquinone] 1 alpha subcomplex subunit 6; n=2; Bos taurus|Rep: NADH dehydrogenase [ubiquinone] 1 alpha subcomplex subunit 6 - Bos taurus (Bovine)		1e-29	80% (37/46)	ES783000
sb_gmnllrta_0003h06.t7	1		Cluster: Hemoglobin subunit alpha-2; n=3; Gadidae|Rep: Hemoglobin subunit alpha-2 - Gadus morhua (Atlantic cod)	GO:0005344 GO:0005506 GO:0005833 GO:0006810 GO:0015671 GO:0019825 GO:0020037 GO:0046872	3e-30	95% (66/69)	ES783201
sb_gmnllrta_0003h07.t7	1		unclassified				ES783250
sb_gmnllrta_0003h17.t7	1		unclassified				ES782070

sb_gmnllrta_0003h19.t7	1		unclassified				ES782370
sb_gmnllrta_0003h23.t7	1		unclassified				ES782922
sb_gmnllrta_0003i13.t7	1		Unassigned protein				ES783075
sb_gmnllrta_0003i14.t7	1		Cluster: UPI0000D8E0CE related cluster; n=1; Danio rerio|Rep: UPI0000D8E0CE UniRef100 entry - Danio rerio		3e-08	29% (39/134)	ES783228
sb_gmnllrta_0003j04.t7	1		Cluster: Homolog of Homo sapiens "Mammalian ependymin related protein-1 precursor; n=1; Takifugu rubripes|Rep: Homolog of Homo sapiens "Mammalian ependymin related protein-1 precursor - Takifugu rubripes		2e-26	58% (36/62)	ES782910
sb_gmnllrta_0003j07.t7	1		ptgs1; prostaglandin-endoperoxide synthase 1 [EC:1.14.99.1]; K00509 prostaglandin-endoperoxide synthase	GO:0001516 GO:0004601 GO:0005506 GO:0006633 GO:0008610 GO:0016020 GO:0016491 GO:0016702 GO:0046872	4e-05	69% (16/23)	ES782845
sb_gmnllrta_0003j14.t7	1		Cluster: KIAA0682-like; n=7; Danio rerio|Rep: KIAA0682-like - Brachydanio rerio (Zebrafish) (Danio rerio)		3e-13	58% (32/55)	ES782353
sb_gmnllrta_0003j16.t7	1		Cluster: Homolog of Homo sapiens "Galectin-3 binding protein precursor; n=1; Takifugu rubripes|Rep: Homolog of Homo sapiens "Galectin-3 binding protein precursor - Takifugu rubripes		7e-09	47% (29/61)	ES782392
sb_gmnllrta_0003j22.t7	1		Cluster: 40S ribosomal protein S21; n=7; Murinae|Rep: 40S ribosomal protein S21 - Mus musculus (Mouse)		2e-26	89% (33/37)	ES783127

sb_gmnllrta_0003k02.t7	1		[J] COG0097 Ribosomal protein L6P/L9E		6e-08	35% (15/42)	ES782566
sb_gmnllrta_0003k05.t7	1		unclassified				ES782411
sb_gmnllrta_0003k08.t7	1		Cluster: Ghitm-prov protein; n=2; Xenopus|Rep: Ghitm-prov protein - Xenopus laevis (African clawed frog)		2e-08	64% (24/37)	ES782012
sb_gmnllrta_0003k10.t7	1		Unassigned protein				ES782766
sb_gmnllrta_0003k12.t7	1		Cluster: Reticulocalbin 3, EF-hand calcium binding domain; n=2; Danio rerio|Rep: Reticulocalbin 3, EF-hand calcium binding domain - Brachydanio rerio (Zebrafish) (Danio rerio)		7e-10	83% (26/31)	ES782650
sb_gmnllrta_0003k15.t7	1		Unassigned protein	GO:0003735 GO:0005622 GO:0005840 GO:0006412 GO:0015934			ES782815
sb_gmnllrta_0003k20.t7	1		Thymosin domain containing protein		8e-14	81% (30/37)	ES782149
sb_gmnllrta_0003l02.t7	1		unclassified				ES782466
sb_gmnllrta_0003l03.t7	1		DENND2D; DENN/MADD domain containing 2D		6e-08	47% (28/59)	ES782527
sb_gmnllrta_0003l04.t7	1		unclassified				ES782362
sb_gmnllrta_0003l05.t7	1		unclassified				ES782318

sb_gmnllrta_0003l12.t7	1		unclassified				ES782700
sb_gmnllrta_0003l21.t7	1		unclassified				ES782018
sb_gmnllrta_0003m04.t7	1		Cluster: Homolog of Brachydanio rerio "Beta-ureidopropionase.; n=1; Takifugu rubripes|Rep: Homolog of Brachydanio rerio "Beta-ureidopropionase. - Takifugu rubripes		3e-58	90% (90/100)	ES782843
sb_gmnllrta_0003m08.t7	1		Cluster: Gapdh protein; n=17; Clupeocephala|Rep: Gapdh protein - Brachydanio rerio (Zebrafish) (Danio rerio)		2e-36	89% (53/59)	ES783212
sb_gmnllrta_0003m12.t7	1		unclassified				ES782545
sb_gmnllrta_0003m14.t7	1		unclassified				ES782438
sb_gmnllrta_0003m18.t7	1		unclassified				ES782029
sb_gmnllrta_0003m23.t7	1		unclassified				ES783254
sb_gmnllrta_0003n03.t7	1		Cluster: Cysteine dioxygenase type 1; n=2; Danio rerio|Rep: Cysteine dioxygenase type 1 - Brachydanio rerio (Zebrafish) (Danio rerio)		1e-14	77% (34/44)	ES782298
sb_gmnllrta_0003n07.t7	1		Unassigned protein				ES782039

sb_gmnllrta_0003n10.t7	1		Cluster: 60S ribosomal protein L35.; n=1; Homo sapiens|Rep: 60S ribosomal protein L35. - Homo sapiens		8e-13	68% (46/67)	ES783072
sb_gmnllrta_0003n12.t7	1		Cluster: Translocase of outer mitochondrial membrane 34; n=3; Danio rerio|Rep: Translocase of outer mitochondrial membrane 34 - Brachydanio rerio (Zebrafish) (Danio rerio)		2e-49	65% (97/149)	ES782956
sb_gmnllrta_0003n14.t7	1		Cluster: Creatine kinase, testis isozyme; n=1; Oncorhynchus mykiss|Rep: Creatine kinase, testis isozyme - Oncorhynchus mykiss (Rainbow trout) (Salmo gairdneri)	GO:0000166 GO:0003824 GO:0004111 GO:0005524 GO:0016301 GO:0016740 GO:0016772	7e-21	68% (50/73)	ES783176
sb_gmnllrta_0003n23.t7	1		Cluster: PREDICTED: similar to polytropic murine leukamia virus receptor SYG1 isoform 1; n=2; Danio rerio|Rep: PREDICTED: similar to polytropic murine leukamia virus receptor SYG1 isoform 1 - Danio rerio		2e-13	97% (33/34)	ES782372
sb_gmnllrta_0003o06.t7	1		unclassified				ES783137
sb_gmnllrta_0003o09.t7	1		unclassified				ES782830
sb_gmnllrta_0003o11.t7	1		unclassified				ES782258
sb_gmnllrta_0003o22.t7	1		unclassified				ES782788
sb_gmnllrta_0003o23.t7	1		unclassified				ES782849
sb_gmnllrta_0003p11.t7	1		unclassified				ES783087

sb_gmnllrta_0003p20.t7	1		unclassified				ES782398
sb_gmnllrta_0003p24.t7	1		Cluster: Aldolase B; n=3; Cyprinodontoidei|Rep: Aldolase B - Poecilia reticulata (Guppy)	GO:0003824 GO:0004332 GO:0006096 GO:0008152	3e-57	81% (68/83)	ES782487
sb_gmnllrta_0004a22.t7	1		unclassified				ES782361
sb_gmnllrta_0004b01.t7	1		unclassified				ES782742
sb_gmnllrta_0004b05.t7	1		Cluster: Ribosomal protein Sa; n=2; Percomorpha|Rep: Ribosomal protein Sa - Solea senegalensis (Sole)	GO:0003735 GO:0005622 GO:0005840 GO:0006412 GO:0015935	4e-28	95% (38/40)	ES782848
sb_gmnllrta_0004b13.t7	1		unclassified				ES782550
sb_gmnllrta_0004b17.t7	1		unclassified				ES782333
sb_gmnllrta_0004b23.t7	1		unclassified				ES783204
sb_gmnllrta_0004b24.t7	1		unclassified				ES783039
sb_gmnllrta_0004c01.t7	1		unclassified				ES782590

sb_gmnllrta_0004c07.t7	1		Cluster: Homolog of Salmo salar "Delta-6 fatty acyl desaturase.; n=1; Takifugu rubripes|Rep: Homolog of Salmo salar "Delta-6 fatty acyl desaturase. - Takifugu rubripes		3e-16	60% (45/74)	ES782410
sb_gmnllrta_0004c09.t7	1		unclassified				ES782102
sb_gmnllrta_0004c12.t7	1		unclassified				ES782758
sb_gmnllrta_0004c13.t7	1		unclassified				ES782707
sb_gmnllrta_0004c17.t7	1		unclassified				ES782810
sb_gmnllrta_0004d06.t7	1		Cluster: Embryonic alpha-type globin; n=1; Oncorhynchus mykiss|Rep: Embryonic alpha-type globin - Oncorhynchus mykiss (Rainbow trout) (Salmo gairdneri)	GO:0005344 GO:0005506 GO:0005833 GO:0006810 GO:0015671 GO:0019825 GO:0020037 GO:0046872	4e-05	53% (24/45)	ES782374
sb_gmnllrta_0004d09.t7	1		Cluster: Calreticulin; n=1; Paralichthys olivaceus|Rep: Calreticulin - Paralichthys olivaceus (Japanese flounder)	GO:0005509 GO:0005783 GO:0006457 GO:0051082	1e-78	88% (134/151)	ES782005
sb_gmnllrta_0004d10.t7	1		MGC86432; MGC86432 protein [EC:1.9.3.1]; K02271 cytochrome c oxidase subunit VIIb	GO:0004129 GO:0005746 GO:0006118	4e-06	65% (15/23)	ES782705
sb_gmnllrta_0004d14.t7	1		Cluster: Homolog of Danio rerio "MID1 interacting protein 1; n=1; Takifugu rubripes|Rep: Homolog of Danio rerio "MID1 interacting protein 1 - Takifugu rubripes		3e-37	80% (76/94)	ES782807

sb_gmnllrta_0004d16.t7	1		unclassified				ES782931
sb_gmnllrta_0004d23.t7	1		unclassified				ES782026
sb_gmnllrta_0004e03.t7	1		unclassified				ES782730
sb_gmnllrta_0004e07.t7	1		unclassified				ES782784
sb_gmnllrta_0004e09.t7	1		unclassified				ES783115
sb_gmnllrta_0004e10.t7	1		Cluster: Dynein heavy chain, cytosolic; n=19; Euteleostomi|Rep: Dynein heavy chain, cytosolic - Homo sapiens (Human)		4e-08	75% (31/41)	ES782547
sb_gmnllrta_0004e12.t7	1		unclassified				ES782511
sb_gmnllrta_0004e17.t7	1		Cluster: MHC class I antigene; n=1; Salmo salar|Rep: MHC class I antigene - Salmo salar (Atlantic salmon)	GO:0006955 GO:0016020 GO:0019882 GO:0042612	2e-12	47% (35/74)	ES782383
sb_gmnllrta_0004e23.t7	1		unclassified				ES783131
sb_gmnllrta_0004f03.t7	1		unclassified				ES782181
sb_gmnllrta_0004f09.t7	1		unclassified				ES782385

sb_gmnllrta_0004f15.t7	1		unclassified				ES783233
sb_gmnllrta_0004f19.t7	1		unclassified				ES782782
sb_gmnllrta_0004f21.t7	1		Unassigned protein				ES782366
sb_gmnllrta_0004g01.t7	1		unclassified				ES783100
sb_gmnllrta_0004g05.t7	1		unclassified				ES783159
sb_gmnllrta_0004g09.t7	1		unclassified				ES782863
sb_gmnllrta_0004g10.t7	1		Cluster: Reticulocalbin 3, EF-hand calcium binding domain; n=2; Danio rerio|Rep: Reticulocalbin 3, EF-hand calcium binding domain - Brachydanio rerio (Zebrafish) (Danio rerio)		1e-10	47% (33/69)	ES782166
sb_gmnllrta_0004g12.t7	1		unclassified				ES782276
sb_gmnllrta_0004g15.t7	1		unclassified				ES782128
sb_gmnllrta_0004g16.t7	1		unclassified				ES782073
sb_gmnllrta_0004h02.t7	1		unclassified				ES782023

sb_gmnllrta_0004h03.t7	1		Cluster: PREDICTED: similar to rat ribosomal protein L9 homologue; n=1; Gallus gallus|Rep: PREDICTED: similar to rat ribosomal protein L9 homologue - Gallus gallus		1e-24	66% (66/100)	ES782044
sb_gmnllrta_0004h05.t7	1		unclassified				ES782171
sb_gmnllrta_0004h09.t7	1		Idh3b; isocitrate dehydrogenase 3 (NAD+) beta		1e-44	87% (68/78)	ES782612
sb_gmnllrta_0004h12.t7	1		Cluster: PREDICTED: similar to glypican-6; n=1; Gallus gallus|Rep: PREDICTED: similar to glypican-6 - Gallus gallus		3e-17	75% (41/54)	ES783209
sb_gmnllrta_0004h22.t7	1		Cluster: Homolog of Homo sapiens "Eukaryotic translation initiation factor 3 subunit 3; n=1; Takifugu rubripes|Rep: Homolog of Homo sapiens "Eukaryotic translation initiation factor 3 subunit 3 - Takifugu rubripes		1e-38	100% (39/39)	ES782575
sb_gmnllrta_0004h24.t7	1		unclassified				ES782436
sb_gmnllrta_0004i04.t7	1		unclassified				ES782997
sb_gmnllrta_0004i05.t7	1		Unassigned protein				ES782964
sb_gmnllrta_0004i08.t7	1		unclassified				ES782695
sb_gmnllrta_0004i13.t7	1		unclassified				ES782120

sb_gmnllrta_0004i15.t7	1		Cluster: Proteasome subunit; n=2; Oncorhynchus mykiss|Rep: Proteasome subunit - Oncorhynchus mykiss (Rainbow trout) (Salmo gairdneri)	GO:0004175 GO:0004298 GO:0005829 GO:0005839 GO:0006511 GO:0008233 GO:0016787 GO:0043234	3e-15	66% (30/45)	ES782252
sb_gmnllrta_0004i16.t7	1		Cluster: Hemoglobin subunit alpha-2; n=3; Gadidae|Rep: Hemoglobin subunit alpha-2 - Gadus morhua (Atlantic cod)	GO:0005344 GO:0005506 GO:0005833 GO:0006810 GO:0015671 GO:0019825 GO:0020037 GO:0046872	2e-21	91% (42/46)	ES782163
sb_gmnllrta_0004i17.t7	1		Cluster: Homolog of Homo sapiens "Transmembrane protein 43; n=1; Takifugu rubripes|Rep: Homolog of Homo sapiens "Transmembrane protein 43 - Takifugu rubripes		2e-41	60% (82/135)	ES782232
sb_gmnllrta_0004j01.t7	1		GRP multi-domain protein		1e-08	45% (28/62)	ES782312
sb_gmnllrta_0004j07.t7	1		unclassified				ES782531
sb_gmnllrta_0004j15.t7	1		unclassified				ES782649
sb_gmnllrta_0004k11.t7	1		Cluster: PREDICTED: similar to 60S ribosomal protein L17 (L23) isoform 3; n=3; Eutheria|Rep: PREDICTED: similar to 60S ribosomal protein L17 (L23) isoform 3 - Canis familiaris		1e-24	91% (45/49)	ES782388
sb_gmnllrta_0004k19.t7	1		Cluster: Ppia protein; n=6; Danio rerio|Rep: Ppia protein - Brachydanio rerio (Zebrafish) (Danio rerio)		1e-45	89% (75/84)	ES782303
sb_gmnllrta_0004l01.t7	1		Cluster: Rps13 protein; n=8; Euteleostomi|Rep: Rps13 protein - Mus		1e-05	81% (27/33)	ES782897

			musculus (Mouse)				
sb_gmnllrta_0004l03.t7	1		Cluster: Ribosomal protein L21; n=2; Otophysi|Rep: Ribosomal protein L21 - Brachydanio rerio (Zebrafish) (Danio rerio)		8e-06	88% (23/26)	ES782840
sb_gmnllrta_0004l09.t7	1		unclassified				ES783071
sb_gmnllrta_0004l11.t7	1		unclassified				ES782341
sb_gmnllrta_0004m05.t7	1		unclassified				ES782465
sb_gmnllrta_0004m07.t7	1		unclassified				ES782599
sb_gmnllrta_0004m08.t7	1		Cluster: Homolog of Homo sapiens "Apolipoprotein B-100 precursor; n=1; Takifugu rubripes|Rep: Homolog of Homo sapiens "Apolipoprotein B-100 precursor - Takifugu rubripes		3e-11	43% (35/80)	ES782257
sb_gmnllrta_0004m17.t7	1		unclassified				ES782675
sb_gmnllrta_0004n08.t7	1		unclassified				ES782655
sb_gmnllrta_0004n11.t7	1		unclassified				ES782106
sb_gmnllrta_0004o06.t7	1		unclassified				

sb_gmnllrta_0004o07.t7	1		Cluster: PREDICTED: similar to Electron-transfer-flavoprotein, beta polypeptide; n=3; Strongylocentrotus purpuratus|Rep: PREDICTED: similar to Electron-transfer-flavoprotein, beta polypeptide - Strongylocentrotus purpuratus		9e-20	61% (35/57)	ES782205
sb_gmnllrta_0004o11.t7	1		Ribosomal_L19e domain containing protein		2e-06	76% (19/25)	ES783211
sb_gmnllrta_0004o19.t7	1		unclassified				ES782750
sb_gmnllrta_0004o20.t7	1		unclassified				ES782592
sb_gmnllrta_0004p02.t7	1		unclassified				ES783234
sb_gmnllrta_0004p11.t7	1		unclassified				ES782135
sb_gmnllrta_0004p15.t7	1		Cluster: PREDICTED: similar to Chromatin modifying protein 5; n=1; Danio rerio|Rep: PREDICTED: similar to Chromatin modifying protein 5 - Danio rerio		9e-13	89% (34/38)	ES782216
sb_gmnllrta_0004p18.t7	1		Cluster: MHC class Ia antigen; n=1; Gadus morhua|Rep: MHC class Ia antigen - Gadus morhua (Atlantic cod)	GO:0006955 GO:0016020 GO:0019882 GO:0042612	1e-14	82% (38/46)	ES782620
sb_gmnllrta_0004p19.t7	1		unclassified				ES782553
sb_gmnllrta_0004p24.t7	1		unclassified				ES782805

sb_gmnllrta_0005a03.t7	1		Cluster: Gapdh protein; n=17; Clupeocephala|Rep: Gapdh protein - Brachydanio rerio (Zebrafish) (Danio rerio)		2e-16	97% (42/43)	ES782141
sb_gmnllrta_0005a06.t7	1		unclassified				ES782175
sb_gmnllrta_0005a09.t7	1		unclassified				ES782516
sb_gmnllrta_0005a12.t7	1		unclassified				ES783124
sb_gmnllrta_0005a15.t7	1		Cluster: Hemoglobin subunit beta-2; n=3; Gadidae|Rep: Hemoglobin subunit beta-2 - Gadus morhua (Atlantic cod)	GO:0005344 GO:0005506 GO:0005833 GO:0006810 GO:0015671 GO:0019825 GO:0020037 GO:0046872	6e-29	94% (65/69)	ES782955
sb_gmnllrta_0005a19.t7	1		Cluster: PREDICTED: similar to ATP synthase, H+ transporting, mitochondrial F0 complex, subunit c isoform 2a precursor; n=1; Canis familiaris|Rep: PREDICTED: similar to ATP synthase, H+ transporting, mitochondrial F0 complex, subunit c isoform 2a precursor - Canis familiaris		2e-22	100% (54/54)	ES782751
sb_gmnllrta_0005a21.t7	1		unclassified				ES782579
sb_gmnllrta_0005b01.t7	1		Cluster: Pituitary tumor-transforming gene 1 protein-interacting protein precursor (Pituitary tumor-transforming gene protein-binding factor) (PTTG-binding factor) (PBF).; n=1; Canis familiaris|Rep: Pituitary tumor-transforming gene 1 protein-interacting protein precursor (Pituitary tumor-transforming gene protein-binding factor) (PTTG-binding factor) (PBF). - Canis familiaris		1e-27	42% (62/146)	ES782898

sb_gmnllrta_0005b02.t7	1		Cluster: Abhydrolase domain-containing protein 2-B; n=2; Danio rerio|Rep: Abhydrolase domain-containing protein 2-B - Brachydanio rerio (Zebrafish) (Danio rerio)		2e-28	75% (58/77)	ES782794
sb_gmnllrta_0005b04.t7	1		Cluster: Hemoglobin subunit alpha-1; n=3; Gadidae|Rep: Hemoglobin subunit alpha-1 - Gadus morhua (Atlantic cod)	GO:0005344 GO:0005506 GO:0005833 GO:0006810 GO:0015671 GO:0019825 GO:0020037 GO:0046872	1e-36	91% (77/84)	ES782683
sb_gmnllrta_0005b05.t7	1		unclassified				ES782628
sb_gmnllrta_0005b14.t7	1		Cluster: PREDICTED: similar to transcriptional regulator; n=4; Danio rerio|Rep: PREDICTED: similar to transcriptional regulator - Danio rerio		2e-46	72% (89/122)	ES782611
sb_gmnllrta_0005b16.t7	1		unclassified				ES782494
sb_gmnllrta_0005c03.t7	1		Cluster: PREDICTED: similar to polytropic murine leukamia virus receptor SYG1 isoform 1; n=2; Danio rerio|Rep: PREDICTED: similar to polytropic murine leukamia virus receptor SYG1 isoform 1 - Danio rerio		4e-13	94% (32/34)	ES782364
sb_gmnllrta_0005c04.t7	1		unclassified				ES782528
sb_gmnllrta_0005c05.t7	1		unclassified				ES782463
sb_gmnllrta_0005c06.t7	1		Cluster: Hemoglobin subunit alpha-2; n=3; Gadidae|Rep: Hemoglobin subunit alpha-2 - Gadus morhua (Atlantic cod)	GO:0005344 GO:0005506 GO:0005833 GO:0006810 GO:0015671 GO:0019825 GO:0020037	1e-27	94% (37/39)	ES782573

				GO:0046872			
sb_gmnllrta_0005c09.t7	1		Cluster: Integrator complex subunit 6; n=2; Danio rerio|Rep: Integrator complex subunit 6 - Brachydanio rerio (Zebrafish) (Danio rerio)		9e-57	75% (108/144)	ES782277
sb_gmnllrta_0005c10.t7	1		unclassified				ES782829
sb_gmnllrta_0005c11.t7	1		Cluster: PREDICTED: similar to ribosomal protein L21; n=4; Murinae|Rep: PREDICTED: similar to ribosomal protein L21 - Mus musculus		1e-22	70% (38/54)	ES782816
sb_gmnllrta_0005c12.t7	1		unclassified				ES782872
sb_gmnllrta_0005c15.t7	1		unclassified				ES782701
sb_gmnllrta_0005c16.t7	1		Unassigned protein				ES782651
sb_gmnllrta_0005c18.t7	1		Cluster: Hemoglobin subunit alpha-1; n=3; Gadidae|Rep: Hemoglobin subunit alpha-1 - Gadus morhua (Atlantic cod)	GO:0005344 GO:0005506 GO:0005833 GO:0006810 GO:0015671 GO:0019825 GO:0020037 GO:0046872	3e-33	91% (71/78)	ES782966
sb_gmnllrta_0005c23.t7	1		unclassified				ES782293
sb_gmnllrta_0005d03.t7	1		Cluster: PREDICTED: similar to DEAD (Asp-Glu-Ala-Asp) box polypeptide 50; n=1; Macaca mulatta|Rep: PREDICTED: similar to DEAD (Asp-Glu-Ala-Asp) box polypeptide 50 - Macaca mulatta		6e-21	62% (30/48)	ES782427

sb_gmnllrta_0005d08.t7	1		unclassified				ES782161
sb_gmnllrta_0005d09.t7	1		unclassified				ES782233
sb_gmnllrta_0005d11.t7	1		Cluster: Hemoglobin subunit beta-2; n=3; Gadidae|Rep: Hemoglobin subunit beta-2 - Gadus morhua (Atlantic cod)	GO:0005344 GO:0005506 GO:0005833 GO:0006810 GO:0015671 GO:0019825 GO:0020037 GO:0046872	3e-29	91% (67/73)	ES782868
sb_gmnllrta_0005d12.t7	1		Unassigned protein				ES782813
sb_gmnllrta_0005d13.t7	1		unclassified				ES782832
sb_gmnllrta_0005d15.t7	1		unclassified				ES782648
sb_gmnllrta_0005d17.t7	1		Cluster: Elongation factor 2; n=2; Caenorhabditis|Rep: Elongation factor 2 - Caenorhabditis elegans		9e-40	82% (74/90)	ES782765
sb_gmnllrta_0005d19.t7	1		HTH_psq domain containing protein		3e-05	37% (17/45)	ES783102
sb_gmnllrta_0005d20.t7	1		unclassified				ES782296
sb_gmnllrta_0005d22.t7	1		unclassified				ES782184
sb_gmnllrta_0005e01.t7	1		unclassified				ES782790

sb_gmnllrta_0005e05.t7	1		unclassified				ES782722
sb_gmnllrta_0005e06.t7	1		Cluster: Hemoglobin subunit alpha-2; n=3; Gadidae|Rep: Hemoglobin subunit alpha-2 - Gadus morhua (Atlantic cod)	GO:0005344<br>GO:0005506<br>GO:0005833<br>GO:0006810<br>GO:0015671<br>GO:0019825<br>GO:0020037<br>GO:0046872	2e-33	95% (70/73)	ES782624
sb_gmnllrta_0005e09.t7	1		Cluster: PREDICTED: similar to ribosomal protein S10; n=1; Pan troglodytes|Rep: PREDICTED: similar to ribosomal protein S10 - Pan troglodytes		9e-05	77% (24/31)	ES783024
sb_gmnllrta_0005e10.t7	1		Cluster: PREDICTED: similar to ribosomal protein L36a; n=1; Pan troglodytes|Rep: PREDICTED: similar to ribosomal protein L36a - Pan troglodytes		4e-37	97% (74/76)	ES782444
sb_gmnllrta_0005e15.t7	1		unclassified				
sb_gmnllrta_0005e16.t7	1		unclassified				ES782539
sb_gmnllrta_0005e19.t7	1		Cluster: Alpha-2-macroglobulin; n=1; Sparus aurata|Rep: Alpha-2-macroglobulin - Sparus aurata (Gilthead sea bream)		2e-34	63% (69/109)	ES782304
sb_gmnllrta_0005e20.t7	1		unclassified				ES783098
sb_gmnllrta_0005e21.t7	1		Cluster: Hemoglobin subunit beta-1; n=2; Gadidae|Rep: Hemoglobin subunit beta-1 - Gadus morhua (Atlantic cod)	GO:0005344<br>GO:0005506<br>GO:0005833<br>GO:0006810<br>GO:0015671<br>GO:0019825<br>GO:0020037	6e-25	97% (45/46)	ES783042

				GO:0046872			
sb_gmnllrta_0005e23.t7	1		unclassified				ES782991
sb_gmnllrta_0005f07.t7	1		Cluster: Simple type II keratin K8a; n=3; Oncorhynchus|Rep: Simple type II keratin K8a - Oncorhynchus mykiss (Rainbow trout) (Salmo gairdneri)	GO:0005198<br>GO:0005882	2e-39	91% (81/89)	ES782302
sb_gmnllrta_0005f10.t7	1		unclassified				
sb_gmnllrta_0005f12.t7	1		Cluster: Coagulation factor V; n=2; Takifugu rubripes|Rep: Coagulation factor V - Fugu rubripes (Japanese pufferfish) (Takifugu rubripes)	GO:0005507<br>GO:0005509<br>GO:0007155<br>GO:0007596<br>GO:0016491	6e-39	69% (72/103)	ES783210
sb_gmnllrta_0005f13.t7	1		unclassified				ES783227
sb_gmnllrta_0005f14.t7	1		Cluster: Glutathione S-transferase M; n=3; Cyprinidae|Rep: Glutathione S-transferase M - Brachydanio rerio (Zebrafish) (Danio rerio)		2e-20	78% (44/56)	ES783074
sb_gmnllrta_0005f15.t7	1		Cluster: Hemoglobin subunit alpha-1; n=3; Gadidae|Rep: Hemoglobin subunit alpha-1 - Gadus morhua (Atlantic cod)	GO:0005344<br>GO:0005506<br>GO:0005833<br>GO:0006810<br>GO:0015671<br>GO:0019825<br>GO:0020037<br>GO:0046872	6e-31	91% (68/74)	ES783047
sb_gmnllrta_0005f17.t7	1		unclassified				ES783026
sb_gmnllrta_0005f22.t7	1		unclassified				ES782577

sb_gmnllrta_0005g03.t7	1		unclassified				ES783150
sb_gmnllrta_0005g15.t7	1		unclassified				ES782253
sb_gmnllrta_0005g16.t7	1		Cluster: Homolog of Homo sapiens "nucleoporin 133kDa; n=1; Takifugu rubripes|Rep: Homolog of Homo sapiens "nucleoporin 133kDa - Takifugu rubripes		3e-09	67% (27/40)	ES782162
sb_gmnllrta_0005g20.t7	1		unclassified				ES782681
sb_gmnllrta_0005g23.t7	1		Cluster: FK506-binding protein; n=3; Obtectomera|Rep: FK506-binding protein - Bombyx mori (Silk moth)	GO:0006457	4e-15	61% (46/75)	ES782745
sb_gmnllrta_0005h02.t7	1		Cluster: Vitronectin protein 1; n=1; Oncorhynchus mykiss|Rep: Vitronectin protein 1 - Oncorhynchus mykiss (Rainbow trout) (Salmo gairdneri)		4e-09	43% (34/79)	ES782202
sb_gmnllrta_0005h03.t7	1		unclassified				ES782182
sb_gmnllrta_0005h04.t7	1		unclassified				ES782016
sb_gmnllrta_0005h06.t7	1		Cluster: PREDICTED: similar to Ribosomal protein L10; n=1; Danio rerio|Rep: PREDICTED: similar to Ribosomal protein L10 - Danio rerio		1e-27	94% (54/57)	ES782150
sb_gmnllrta_0005h09.t7	1		Cluster: 60S ribosomal protein L27A; n=7; Euteleostei|Rep: 60S ribosomal protein L27A - Platichthys flesus (European flounder)	GO:0003735 GO:0005622 GO:0005840 GO:0006412 GO:0030529	2e-48	94% (91/96)	ES782384

sb_gmnllrta_0005h10.t7	1		Cluster: Ribosomal protein L10; n=7; Euteleostomi|Rep: Ribosomal protein L10 - Brachydanio rerio (Zebrafish) (Danio rerio)		3e-34	91% (65/71)	ES782957
sb_gmnllrta_0005h13.t7	1		unclassified				ES783054
sb_gmnllrta_0005h16.t7	1		Cluster: Keratin type IIE; n=1; Acipenser baerii|Rep: Keratin type IIE - Acipenser baerii (Siberian sturgeon)	GO:0005198 GO:0005882	7e-12	87% (35/40)	ES783188
sb_gmnllrta_0005i01.t7	1		Cluster: Transcribed locus, weakly similar to XP_518227.1 desmoplakin [Pan troglodytes]; n=1; Takifugu rubripes|Rep: Transcribed locus, weakly similar to XP_518227.1 desmoplakin [Pan troglodytes] - Takifugu rubripes		2e-30	81% (58/71)	ES783099
sb_gmnllrta_0005i05.t7	1		Cluster: RuvB-like 1; n=6; Clupeocephala|Rep: RuvB-like 1 - Brachydanio rerio (Zebrafish) (Danio rerio)		3e-74	92% (137/148)	ES783160
sb_gmnllrta_0005i06.t7	1		Cluster: 60S ribosomal protein L19; n=18; Amniota|Rep: 60S ribosomal protein L19 - Homo sapiens (Human)		1e-32	90% (46/51)	ES783258
sb_gmnllrta_0005i12.t7	1		unclassified				ES782275
sb_gmnllrta_0005i14.t7	1		unclassified				ES782097
sb_gmnllrta_0005i15.t7	1		unclassified				ES782127
sb_gmnllrta_0005i16.t7	1		unclassified				ES782074

sb_gmnllrta_0005i17.t7	1		unclassified				ES782007
sb_gmnllrta_0005i21.t7	1		Cluster: Type I cytokeratin; n=3; Danio rerio|Rep: Type I cytokeratin - Brachydanio rerio (Zebrafish) (Danio rerio)		3e-55	77% (85/110)	ES782846
sb_gmnllrta_0005j01.t7	1		unclassified				ES782526
sb_gmnllrta_0005j02.t7	1		unclassified				ES782594
sb_gmnllrta_0005j08.t7	1		unclassified				ES782076
sb_gmnllrta_0005j09.t7	1		Cluster: Novel protein similar to vertebrate zinc finger, DHHC domain containing 14; n=2; Danio rerio|Rep: Novel protein similar to vertebrate zinc finger, DHHC domain containing 14 - Brachydanio rerio (Zebrafish) (Danio rerio)		4e-09	61% (27/44)	ES782006
sb_gmnllrta_0005j10.t7	1		unclassified				ES782704
sb_gmnllrta_0005j11.t7	1		Cluster: Kinesin-associated protein 3; n=25; Amniota|Rep: Kinesin-associated protein 3 - Homo sapiens (Human)		7e-32	63% (51/80)	ES782759
sb_gmnllrta_0005j19.t7	1		mrpl35; mitochondrial ribosomal protein L35		8e-23	73% (49/67)	ES783195
sb_gmnllrta_0005j21.t7	1		unclassified				ES782144
sb_gmnllrta_0005j22.t7	1		Unassigned protein				ES782041

sb_gmnllrta_0005j23.t7	1		Cluster: Ribosomal protein S30; n=3; Percomorpha|Rep: Ribosomal protein S30 - Solea senegalensis (Sole)	GO:0003735 GO:0005622 GO:0005840 GO:0006412 GO:0006464	4e-25	83% (56/67)	ES782028
sb_gmnllrta_0005j24.t7	1		[O] COG0625 Glutathione S-transferase		1e-04	38% (23/60)	ES782174
sb_gmnllrta_0005k01.t7	1		LSU rRNA; Neoceratodus forsteri		7e-45	96% (108/112)	ES782680
sb_gmnllrta_0005k02.t7	1		Cluster: 40S ribosomal protein S11; n=14; Euteleostomi|Rep: 40S ribosomal protein S11 - Xenopus laevis (African clawed frog)	GO:0003723 GO:0003735 GO:0005622 GO:0005840 GO:0006412 GO:0019843 GO:0030529	4e-39	82% (81/98)	ES782746
sb_gmnllrta_0005k03.t7	1		Cluster: Hemoglobin subunit alpha-1; n=3; Gadidae|Rep: Hemoglobin subunit alpha-1 - Gadus morhua (Atlantic cod)	GO:0005344 GO:0005506 GO:0005833 GO:0006810 GO:0015671 GO:0019825 GO:0020037 GO:0046872	2e-07	86% (26/30)	ES782729
sb_gmnllrta_0005k06.t7	1		unclassified				ES782852
sb_gmnllrta_0005k07.t7	1		Cluster: Homolog of Homo sapiens "CLN8 protein; n=1; Takifugu rubripes|Rep: Homolog of Homo sapiens "CLN8 protein - Takifugu rubripes		3e-09	78% (30/38)	ES782785
sb_gmnllrta_0005k17.t7	1		LOC536558; similar to heat shock 70kDa protein 4; K09485 heat shock protein 110kDa		2e-31	85% (66/77)	ES782382
sb_gmnllrta_0005k18.t7	1		Cluster: Capsid protein; n=41; Enterobacteria phage phiX174 sensu lato|Rep: Capsid protein - Bacteriophage	GO:0005198	2e-57	93%	ES782152

			phi-X174	GO:0019028		(60/64)	
sb_gmnllrta_0005k20.t7	1		Unassigned protein				ES783200
sb_gmnllrta_0005k23.t7	1		Cluster: PREDICTED: similar to putative acetyltransferase; n=1; Gallus gallus|Rep: PREDICTED: similar to putative acetyltransferase - Gallus gallus		7e-16	64% (32/50)	ES783132
sb_gmnllrta_0005k24.t7	1		unclassified				ES782962
sb_gmnllrta_0005l04.t7	1		unclassified				ES782787
sb_gmnllrta_0005l07.t7	1		Cluster: Brain-type fatty-acid binding protein; n=2; Danio rerio|Rep: Brain-type fatty-acid binding protein - Brachydanio rerio (Zebrafish) (Danio rerio)		2e-10	85% (30/35)	ES782890
sb_gmnllrta_0005l08.t7	1		brp44; brain protein 44		5e-16	79% (38/48)	ES783231
sb_gmnllrta_0005l11.t7	1		Cluster: JunDLa; n=2; Tetraodontidae|Rep: JunDLa - Fugu rubripes (Japanese pufferfish) (Takifugu rubripes)	GO:0003677 GO:0003700 GO:0005634 GO:0006355 GO:0043565 GO:0046983	9e-48	86% (93/107)	ES782510
sb_gmnllrta_0005l17.t7	1		unclassified				ES782334
sb_gmnllrta_0005l18.t7	1		Cluster: Elongation factor 1-alpha; n=36; Vertebrata|Rep: Elongation factor 1-alpha - Brachydanio rerio (Zebrafish) (Danio rerio)		4e-08	96% (28/29)	ES782046
sb_gmnllrta_0005l21.t7	1		Cluster: Ribosomal protein S30; n=3; Percomorpha|Rep: Ribosomal protein S30	GO:0003735 GO:0005622 GO:0005840	4e-25	87% (55/63)	ES783149

			- Solea senegalensis (Sole)	GO:0006412 GO:0006464			
sb_gmnllrta_0005m07.t7	1		unclassified				ES782409
sb_gmnllrta_0005m08.t7	1		unclassified				ES782123
sb_gmnllrta_0005m10.t7	1		unclassified				ES782658
sb_gmnllrta_0005m12.t7	1		Cluster: 60S ribosomal protein L35; n=17; Tetrapoda|Rep: 60S ribosomal protein L35 - Rattus norvegicus (Rat)		3e-19	98% (49/50)	ES782757
sb_gmnllrta_0005m15.t7	1		Cluster: Elongation factor 1-alpha; n=36; Vertebrata|Rep: Elongation factor 1-alpha - Brachydanio rerio (Zebrafish) (Danio rerio)		5e-33	92% (63/68)	ES782928
sb_gmnllrta_0005m18.t7	1		ckb; creatine kinase, brain [EC:2.7.3.2]; K00933 creatine kinase		4e-30	81% (57/70)	ES783158
sb_gmnllrta_0005m19.t7	1		unclassified				ES783129
sb_gmnllrta_0005m20.t7	1		Cluster: Aldehyde dehydrogenase 8 family, member A1; n=5; Danio rerio|Rep: Aldehyde dehydrogenase 8 family, member A1 - Brachydanio rerio (Zebrafish) (Danio rerio)		2e-74	73% (137/187)	ES782031
sb_gmnllrta_0005m22.t7	1		unclassified				ES782142
sb_gmnllrta_0005n01.t7	1		LOC607198; similar to Acyl-CoA-binding protein (ACBP) (Diazepam binding inhibitor) (DBI) (Endozepine) (EP)		2e-05	68% (22/32)	ES782982

sb_gmnllrta_0005n07.t7	1		unclassified				ES783130
sb_gmnllrta_0005n08.t7	1		unclassified				ES782834
sb_gmnllrta_0005n11.t7	1		Cluster: Xgly4 protein; n=3; Xenopus|Rep: Xgly4 protein - Xenopus laevis (African clawed frog)	GO:0005578	3e-32	69% (63/91)	ES782272
sb_gmnllrta_0005n15.t7	1		Cluster: Ubiquitin fusion degradation 1-like protein; n=2; Danio rerio|Rep: Ubiquitin fusion degradation 1-like protein - Brachydanio rerio (Zebrafish) (Danio rerio)		2e-96	85% (180/210)	ES782071
sb_gmnllrta_0005n19.t7	1		unclassified				ES782408
sb_gmnllrta_0005n20.t7	1		LSU rRNA; Squalus acanthias		2e-57	95% (127/133)	ES782901
sb_gmnllrta_0005n22.t7	1		Cluster: Procathepsin L; n=2; Oncorhynchus mykiss|Rep: Procathepsin L - Oncorhynchus mykiss (Rainbow trout) (Salmo gairdneri)	GO:0004197<br>GO:0006508<br>GO:0008233<br>GO:0008234<br>GO:0016787	2e-15	90% (38/42)	ES782842
sb_gmnllrta_0005n24.t7	1		Cluster: Heat shock protein 90 beta; n=7; Euteleostomi|Rep: Heat shock protein 90 beta - Paralichthys olivaceus (Japanese flounder)	GO:0000166<br>GO:0005524<br>GO:0006457<br>GO:0051082	3e-31	97% (48/49)	ES782627
sb_gmnllrta_0005o04.t7	1		Cluster: 60S ribosomal protein L6; n=6; Rattus norvegicus|Rep: 60S ribosomal protein L6 - Rattus norvegicus (Rat)		1e-48	83% (87/104)	ES782088
sb_gmnllrta_0005o06.t7	1		unclassified				ES782047

sb_gmnllrta_0005o10.t7	1		unclassified				ES783057
sb_gmnllrta_0005o12.t7	1		unclassified				ES783018
sb_gmnllrta_0005o14.t7	1		unclassified				ES783119
sb_gmnllrta_0005o15.t7	1		Cluster: 60S ribosomal protein L34; n=2; Rattus norvegicus|Rep: 60S ribosomal protein L34 - Rattus norvegicus (Rat)		3e-07	83% (26/31)	ES783185
sb_gmnllrta_0005o20.t7	1		unclassified				ES782415
sb_gmnllrta_0005o22.t7	1		Cluster: Fibrinogen, B beta polypeptide; n=2; Danio rerio|Rep: Fibrinogen, B beta polypeptide - Brachydanio rerio (Zebrafish) (Danio rerio)		2e-23	68% (47/69)	ES782363
sb_gmnllrta_0005o23.t7	1		Cluster: Hemoglobin subunit alpha-2; n=3; Gadidae|Rep: Hemoglobin subunit alpha-2 - Gadus morhua (Atlantic cod)	GO:0005344 GO:0005506 GO:0005833 GO:0006810 GO:0015671 GO:0019825 GO:0020037 GO:0046872	2e-22	96% (31/32)	ES782317
sb_gmnllrta_0005p03.t7	1		Cluster: PREDICTED: adaptor-related protein complex 2, mu 1 subunit isoform 9; n=18; Eutheria|Rep: PREDICTED: adaptor-related protein complex 2, mu 1 subunit isoform 9 - Pan troglodytes		1e-16	90% (40/44)	ES783077
sb_gmnllrta_0005p04.t7	1		Cluster: Homolog of Brachydanio rerio "Mcl1b.; n=1; Takifugu rubripes|Rep: Homolog of Brachydanio rerio "Mcl1b. - Takifugu rubripes		6e-16	64% (36/56)	ES783238

sb_gmnllrta_0005p05.t7	1		Cluster: Heat shock protein 90 beta; n=7; Euteleostomi|Rep: Heat shock protein 90 beta - Paralichthys olivaceus (Japanese flounder)	GO:0000166 GO:0005524 GO:0006457 GO:0051082	4e-42	79% (87/110)	ES783218
sb_gmnllrta_0005p07.t7	1		unclassified				ES783173
sb_gmnllrta_0005p11.t7	1		Cluster: Complement component C3; n=1; Gadus morhua|Rep: Complement component C3 - Gadus morhua (Atlantic cod)		3e-31	68% (64/94)	ES782240
sb_gmnllrta_0005p12.t7	1		unclassified				ES782193
sb_gmnllrta_0005p15.t7	1		unclassified				ES782035
sb_gmnllrta_0005p17.t7	1		Cluster: Homolog of Brachydanio rerio "Ddx5 protein.; n=1; Takifugu rubripes|Rep: Homolog of Brachydanio rerio "Ddx5 protein. - Takifugu rubripes		2e-31	95% (47/49)	ES782138
sb_gmnllrta_0005p18.t7	1		Cluster: CD36 antigen; n=1; Oncorhynchus mykiss|Rep: CD36 antigen - Oncorhynchus mykiss (Rainbow trout) (Salmo gairdneri)	GO:0004872 GO:0005764 GO:0007155 GO:0016020	1e-23	37% (64/170)	ES782399
sb_gmnllrta_0005p20.t7	1		Cluster: Glucose phosphate isomerase a; n=4; Danio rerio|Rep: Glucose phosphate isomerase a - Brachydanio rerio (Zebrafish) (Danio rerio)		3e-37	90% (54/60)	ES782935
sb_gmnllrta_0005p21.t7	1		Cluster: Novel protein similar to vertebrate organic cation transporter; n=1; Danio rerio|Rep: Novel protein similar to vertebrate organic cation transporter - Brachydanio rerio (Zebrafish) (Danio rerio)		1e-10	66% (30/45)	ES782879

sb_gmnllrta_0005p24.t7	1		unclassified				ES782665
sb_gmnllrta_0006a04.t7	1		unclassified				ES782346
sb_gmnllrta_0006a08.t7	1		Cluster: PREDICTED: similar to ribosomal protein L19; n=1; Rattus norvegicus|Rep: PREDICTED: similar to ribosomal protein L19 - Rattus norvegicus		1e-06	56% (32/57)	ES782082
sb_gmnllrta_0006a09.t7	1		unclassified				ES782139
sb_gmnllrta_0006a10.t7	1		Ribosomal_L19e domain containing protein		2e-06	76% (19/25)	ES782690
sb_gmnllrta_0006a11.t7	1		Cluster: Homolog of Brachydanio rerio "Intestinal fatty acid binding protein 2 (Fabp2 protein).; n=1; Takifugu rubripes|Rep: Homolog of Brachydanio rerio "Intestinal fatty acid binding protein 2 (Fabp2 protein). - Takifugu rubripes		2e-60	82% (109/132)	ES782637
sb_gmnllrta_0006a13.t7	1		Cluster: Homolog of Gallus gallus "FK-506 binding protein 51.; n=1; Takifugu rubripes|Rep: Homolog of Gallus gallus "FK-506 binding protein 51. - Takifugu rubripes		3e-40	75% (78/104)	ES782734
sb_gmnllrta_0006a21.t7	1		Unassigned protein				ES782003
sb_gmnllrta_0006a22.t7	1		Cluster: Similar to ribosomal protein S27; n=1; Homo sapiens|Rep: Similar to ribosomal protein S27 - Homo sapiens (Human)		9e-11	56% (44/78)	ES782114
sb_gmnllrta_0006a24.t7	1		Cluster: Ribosomal protein Sa; n=2; Percomorpha|Rep: Ribosomal protein Sa - Solea senegalensis (Sole)	GO:0003735 GO:0005622 GO:0005840 GO:0006412	6e-39	96% (73/76)	ES782283

				GO:0015935			
sb_gmnllrta_0006b04.t7	1		unclassified				ES783240
sb_gmnllrta_0006b09.t7	1		Cluster: Homolog of Oncorhynchus mykiss "Rhamnose binding lectin STL2.; n=1; Takifugu rubripes|Rep: Homolog of Oncorhynchus mykiss "Rhamnose binding lectin STL2. - Takifugu rubripes		9e-16	52% (45/86)	ES782857
sb_gmnllrta_0006b10.t7	1		Cluster: Warm temperature acclimation-related 65 kDa protein; n=1; Dicentrarchus labrax|Rep: Warm temperature acclimation-related 65 kDa protein - Dicentrarchus labrax (European sea bass)		5e-13	60% (29/48)	ES782286
sb_gmnllrta_0006b11.t7	1		unclassified				ES782239
sb_gmnllrta_0006b13.t7	1		unclassified				ES782211
sb_gmnllrta_0006b15.t7	1		unclassified				ES782036
sb_gmnllrta_0006b19.t7	1		unclassified				ES782457
sb_gmnllrta_0006b22.t7	1		unclassified				ES782806
sb_gmnllrta_0006c07.t7	1		unclassified				ES782063
sb_gmnllrta_0006c09.t7	1		unclassified				ES782380

sb_gmnllrta_0006c10.t7	1		Thymosin domain containing protein		8e-14	81% (30/37)	ES783085
sb_gmnllrta_0006c12.t7	1		unclassified				ES782981
sb_gmnllrta_0006c21.t7	1		Cluster: translation elongation factor 2; n=3; Endopterygota|Rep: translation elongation factor 2 - Bombyx mori		1e-14	92% (38/41)	ES782401
sb_gmnllrta_0006d08.t7	1		unclassified				ES782403
sb_gmnllrta_0006d10.t7	1		unclassified				ES783002
sb_gmnllrta_0006d11.t7	1		Cluster: Drebrin-like protein (SH3 domain-containing protein 7) (Drebrin-F) (Cervical SH3P7) (HPK1-interacting protein of 55 kDa) (HIP-55) (Cervical mucin-associated protein).; n=2; Gallus gallus|Rep: Drebrin-like protein (SH3 domain-containing protein 7) (Drebrin-F) (Cervical SH3P7) (HPK1-interacting protein of 55 kDa) (HIP-55) (Cervical mucin-associated protein). - Gallus gallus		5e-39	82% (58/70)	ES782980
sb_gmnllrta_0006d13.t7	1		Cluster: Homolog of Homo sapiens "inter-alpha (globulin) inhibitor H4 (plasma Kallikrein-sensitive glycoprotein); n=2; Takifugu rubripes|Rep: Homolog of Homo sapiens "inter-alpha (globulin) inhibitor H4 (plasma Kallikrein-sensitive glycoprotein) - Takifugu rubripes		6e-37	65% (78/120)	ES783086
sb_gmnllrta_0006d16.t7	1		unclassified				ES783140
sb_gmnllrta_0006d17.t7	1		unclassified				ES783167

sb_gmnllrta_0006d22.t7	1		LSU rRNA; Hydrolagus colliei		6e-61	99% (118/119)	ES782400
sb_gmnllrta_0006d23.t7	1		unclassified				ES782455
sb_gmnllrta_0006d24.t7	1		Cluster: Myeloperoxidase; n=1; Siniperca chuatsi|Rep: Myeloperoxidase - Siniperca chuatsi (Chinese perch)	GO:0004601 GO:0046872	4e-05	57% (22/38)	ES782622
sb_gmnllrta_0006e07.t7	1		Cluster: Nucleolar complex protein 4 homolog; n=2; Danio rerio|Rep: Nucleolar complex protein 4 homolog - Brachydanio rerio (Zebrafish) (Danio rerio)		1e-17	90% (39/43)	ES783242
sb_gmnllrta_0006e12.t7	1		unclassified				ES782238
sb_gmnllrta_0006e13.t7	1		Cluster: 40S ribosomal protein S23.; n=5; Amniota|Rep: 40S ribosomal protein S23. - Rattus norvegicus		9e-53	96% (104/108)	ES782287
sb_gmnllrta_0006e18.t7	1		unclassified				ES782324
sb_gmnllrta_0006e20.t7	1		Cluster: Proteasome subunit; n=2; Oncorhynchus mykiss|Rep: Proteasome subunit - Oncorhynchus mykiss (Rainbow trout) (Salmo gairdneri)	GO:0004175 GO:0004298 GO:0005829 GO:0005839 GO:0006511 GO:0008233 GO:0016787 GO:0043234	1e-18	66% (36/54)	ES782824
sb_gmnllrta_0006e23.t7	1		unclassified				ES782936
sb_gmnllrta_0006e24.t7	1		Cluster: Tetraspanin 7; n=1; Danio rerio|Rep: Tetraspanin 7 - Brachydanio rerio (Zebrafish) (Danio rerio)		1e-10	72% (39/54)	ES782780

sb_gmnllrta_0006f09.t7	1		Cluster: Warm temperature acclimation-related 65kDa protein; n=1; Xiphophorus hellerii|Rep: Warm temperature acclimation-related 65kDa protein - Xiphophorus helleri		2e-31	73% (58/79)	ES782058
sb_gmnllrta_0006f10.t7	1		unclassified				ES782735
sb_gmnllrta_0006f17.t7	1		Cluster: Flavin-containing monooxygenase FMO1; n=1; Oncorhynchus mykiss|Rep: Flavin-containing monooxygenase FMO1 - Oncorhynchus mykiss (Rainbow trout) (Salmo gairdneri)	GO:0004497 GO:0004499 GO:0005792 GO:0006118 GO:0016491 GO:0031227 GO:0050660 GO:0050661	2e-19	70% (34/48)	ES782906
sb_gmnllrta_0006f22.t7	1		MGC79028; MGC79028 protein [EC:1.1.1.41]; K00030 isocitrate dehydrogenase (NAD+)		2e-36	81% (73/90)	ES782002
sb_gmnllrta_0006g01.t7	1		unclassified				ES782644
sb_gmnllrta_0006g02.t7	1		Mucin multi-domain protein		2e-06	30% (21/68)	ES782711
sb_gmnllrta_0006g04.t7	1		unclassified				ES782939
sb_gmnllrta_0006g05.t7	1		unclassified				ES782873
sb_gmnllrta_0006g10.t7	1		Cluster: 3-hydroxyanthranilate 3,4-dioxygenase; n=3; Danio rerio|Rep: 3-hydroxyanthranilate 3,4-dioxygenase - Brachydanio rerio (Zebrafish) (Danio rerio)		6e-33	60% (61/101)	ES782583
sb_gmnllrta_0006g20.t7	1		Cluster: Hemoglobin subunit alpha-1; n=3; Gadidae|Rep: Hemoglobin subunit alpha-1	GO:0005344 GO:0005506 GO:0005833	7e-10	88%	ES783236

			- Gadus morhua (Atlantic cod)	GO:0006810 GO:0015671 GO:0019825 GO:0020037 GO:0046872		(32/36)	
sb_gmnllrta_0006g21.t7	1		LOC607198; similar to Acyl-CoA-binding protein (ACBP) (Diazepam binding inhibitor) (DBI) (Endozepine) (EP)		2e-21	71% (47/66)	ES783221
sb_gmnllrta_0006g24.t7	1		Cluster: Elongation factor 1-gamma; n=7; Clupeocephala|Rep: Elongation factor 1-gamma - Brachydanio rerio (Zebrafish) (Danio rerio)		2e-24	80% (51/63)	ES783017
sb_gmnllrta_0006h06.t7	1		Mucin domain containing protein		2e-06	30% (21/68)	ES782509
sb_gmnllrta_0006h10.t7	1		LSU rRNA; Neoceratodus forsteri		1e-18	86% (91/105)	ES782884
sb_gmnllrta_0006h11.t7	1		Cluster: 60S ribosomal protein L12; n=11; Euteleostomi|Rep: 60S ribosomal protein L12 - Brachydanio rerio (Zebrafish) (Danio rerio)		5e-51	93% (100/107)	ES782904
sb_gmnllrta_0006h17.t7	1		NAP domain containing protein		9e-08	36% (17/46)	ES782717
sb_gmnllrta_0006i01.t7	1		Cluster: PREDICTED: similar to Ribosomal protein L10; n=1; Danio rerio|Rep: PREDICTED: similar to Ribosomal protein L10 - Danio rerio		9e-29	98% (56/57)	ES782823
sb_gmnllrta_0006i02.t7	1		unclassified				ES782937
sb_gmnllrta_0006i03.t7	1		Cluster: PREDICTED: similar to mitotic centromere-associated kinesin; n=2; Danio rerio|Rep: PREDICTED: similar to mitotic centromere-associated kinesin - Danio rerio		1e-30	51% (74/143)	ES782877

sb_gmnllrta_0006i08.t7	1		unclassified				ES783006
sb_gmnllrta_0006i17.t7	1		Cluster: Homolog of Homo sapiens "Plasma serine protease inhibitor precursor; n=4; Takifugu rubripes|Rep: Homolog of Homo sapiens "Plasma serine protease inhibitor precursor - Takifugu rubripes		3e-25	57% (52/90)	ES782560
sb_gmnllrta_0006i18.t7	1		Cluster: Serotransferrin; n=1; Gadus morhua|Rep: Serotransferrin - Gadus morhua (Atlantic cod)	GO:0005506 GO:0005576 GO:0006810 GO:0006811 GO:0006826 GO:0006879 GO:0008199 GO:0046872	3e-37	97% (78/80)	ES782285
sb_gmnllrta_0006i19.t7	1		unclassified				ES782263
sb_gmnllrta_0006i21.t7	1		Cluster: PREDICTED: similar to DEAH (Asp-Glu-Ala-His) box polypeptide 33,; n=1; Monodelphis domestica|Rep: PREDICTED: similar to DEAH (Asp-Glu-Ala-His) box polypeptide 33, - Monodelphis domestica		4e-28	60% (39/65)	ES783080
sb_gmnllrta_0006i22.t7	1		unclassified				ES783012
sb_gmnllrta_0006i23.t7	1		Cluster: Glypican-6 precursor; n=10; Amniota|Rep: Glypican-6 precursor - Homo sapiens (Human)		1e-14	82% (19/23)	ES782945
sb_gmnllrta_0006j04.t7	1		unclassified				ES782153
sb_gmnllrta_0006j06.t7	1		unclassified				ES782284

sb_gmnllrta_0006j11.t7	1		Cluster: Hemoglobin subunit alpha-2; n=3; Gadidae|Rep: Hemoglobin subunit alpha-2 - Gadus morhua (Atlantic cod)	GO:0005344 GO:0005506 GO:0005833 GO:0006810 GO:0015671 GO:0019825 GO:0020037 GO:0046872	8e-06	75% (25/33)	ES783164
sb_gmnllrta_0006j13.t7	1		unclassified				ES783191
sb_gmnllrta_0006j17.t7	1		unclassified				ES782976
sb_gmnllrta_0006j20.t7	1		unclassified				ES782485
sb_gmnllrta_0006j21.t7	1		unclassified				ES782504
sb_gmnllrta_0006k01.t7	1		unclassified				ES783237
sb_gmnllrta_0006k04.t7	1		Cluster: Solute carrier family 25 (Mitochondrial carrier; citrate transporter), member 1; n=2; Xenopus|Rep: Solute carrier family 25 (Mitochondrial carrier; citrate transporter), member 1 - Xenopus tropicalis (Western clawed frog) (Silurana tropicalis)	GO:0005215 GO:0005488 GO:0005743 GO:0006810 GO:0016020 GO:0016021	1e-16	88% (40/45)	ES782940
sb_gmnllrta_0006k07.t7	1		SAMHD1; SAM domain and HD domain 1		3e-40	50% (90/179)	ES783060
sb_gmnllrta_0006k08.t7	1		Cluster: MHC class I antigen precursor; n=1; Barbus intermedius|Rep: MHC class I antigen precursor - Barbus intermedius (Lake tana barbels)	GO:0006955 GO:0016020 GO:0019882 GO:0042612	3e-40	53% (76/142)	ES782739
sb_gmnllrta_0006k10.t7	1		Cluster: Cathepsin L; n=1; Oryzias latipes|Rep: Cathepsin L - Oryzias latipes	GO:0004197 GO:0006508 GO:0008233	6e-64	75%	ES782037

			(Medaka fish) (Japanese ricefish)	GO:0008234 GO:0016787		(56/74)	
sb_gmnllrta_0006k11.t7	1		PXMP2; peroxisomal membrane protein 2, 22kDa		4e-36	47% (74/156)	ES782054
sb_gmnllrta_0006k14.t7	1		Cluster: Hemoglobin subunit alpha-1; n=3; Gadidae|Rep: Hemoglobin subunit alpha-1 - Gadus morhua (Atlantic cod)	GO:0005344 GO:0005506 GO:0005833 GO:0006810 GO:0015671 GO:0019825 GO:0020037 GO:0046872	7e-25	90% (57/63)	ES782235
sb_gmnllrta_0006k18.t7	1		Cluster: NMDA receptor-regulated gene 1a; n=4; Danio rerio|Rep: NMDA receptor-regulated gene 1a - Brachydanio rerio (Zebrafish) (Danio rerio)		5e-14	92% (35/38)	ES782508
sb_gmnllrta_0006k19.t7	1		unclassified				ES782480
sb_gmnllrta_0006l10.t7	1		unclassified				ES782084
sb_gmnllrta_0006l21.t7	1		unclassified				ES782770
sb_gmnllrta_0006l22.t7	1		unclassified				ES782661
sb_gmnllrta_0006m02.t7	1		Cluster: 60S ribosomal protein L13; n=2; Clupeocephala|Rep: 60S ribosomal protein L13 - Brachydanio rerio (Zebrafish) (Danio rerio)		1e-21	89% (52/58)	ES782129
sb_gmnllrta_0006m14.t7	1		unclassified				ES782978
sb_gmnllrta_0006m16.t7	1		unclassified				ES783088

sb_gmnllrta_0006m21.t7	1		Cluster: Zgc:55845; n=3; Danio rerio|Rep: Zgc:55845 - Brachydanio rerio (Zebrafish) (Danio rerio)		1e-05	79% (23/29)	ES782616
sb_gmnllrta_0006m22.t7	1		Unassigned protein				ES782502
sb_gmnllrta_0006m23.t7	1		Cluster: Ferritin heavy subunit; n=1; Ictalurus punctatus|Rep: Ferritin heavy subunit - Ictalurus punctatus (Channel catfish)	GO:0005488 GO:0005506 GO:0006826 GO:0006879 GO:0008199 GO:0016491 GO:0046872 GO:0046914	4e-06	73% (25/34)	ES782486
sb_gmnllrta_0006n04.t7	1		Cluster: Homolog of Homo sapiens "nucleoporin 133kDa; n=1; Takifugu rubripes|Rep: Homolog of Homo sapiens "nucleoporin 133kDa - Takifugu rubripes		1e-19	60% (42/70)	ES782641
sb_gmnllrta_0006n05.t7	1		Unassigned protein				ES782664
sb_gmnllrta_0006n06.t7	1		unclassified				ES782778
sb_gmnllrta_0006n11.t7	1		LOC761080; similar to reverse transcriptase-like protein		9e-38	43% (69/157)	ES782377
sb_gmnllrta_0006n14.t7	1		Cluster: Complement component C3; n=1; Anarhichas minor|Rep: Complement component C3 - Anarhichas minor (Arctic spotted wolffish)	GO:0004866 GO:0005576	5e-40	58% (75/128)	ES782563
sb_gmnllrta_0006n15.t7	1		unclassified				ES782584
sb_gmnllrta_0006n16.t7	1		unclassified				ES782534

sb_gmnllrta_0006n21.t7	1		Cluster: Homolog of Homo sapiens "nucleoporin 133kDa; n=1; Takifugu rubripes|Rep: Homolog of Homo sapiens "nucleoporin 133kDa - Takifugu rubripes		5e-10	79% (19/24)	ES783009
sb_gmnllrta_0006o01.t7	1		LSU rRNA; Homo sapiens		4e-85	94% (189/200)	ES782452
sb_gmnllrta_0006o07.t7	1		Cluster: Homolog of Brachydanio rerio "Intestinal fatty acid binding protein 2 (Fabp2 protein).; n=1; Takifugu rubripes|Rep: Homolog of Brachydanio rerio "Intestinal fatty acid binding protein 2 (Fabp2 protein). - Takifugu rubripes		2e-60	82% (109/132)	ES782554
sb_gmnllrta_0006o08.t7	1		unclassified				ES782288
sb_gmnllrta_0006o17.t7	1		Cluster: Eukaryotic initiation factor 4A-III; n=24; Eukaryota|Rep: Eukaryotic initiation factor 4A-III - Homo sapiens (Human)		9e-88	86% (167/194)	ES782634
sb_gmnllrta_0006o19.t7	1		unclassified				ES782941
sb_gmnllrta_0006o20.t7	1		unclassified				ES782160
sb_gmnllrta_0006o24.t7	1		unclassified				ES782110
sb_gmnllrta_0006p01.t7	1		Cluster: Ribosomal protein Sa; n=2; Percomorpha|Rep: Ribosomal protein Sa - Solea senegalensis (Sole)	GO:0003735 GO:0005622 GO:0005840 GO:0006412 GO:0015935	7e-35	94% (66/70)	ES782900
sb_gmnllrta_0006p06.t7	1		unclassified				ES782727

sb_gmnllrta_0006p09.t7	1		unclassified				ES783069
sb_gmnllrta_0006p11.t7	1		unclassified				ES782338
sb_gmnllrta_0006p12.t7	1		Ribosomal_L19e domain containing protein		5e-06	72% (18/25)	ES782395
sb_gmnllrta_0006p17.t7	1		Cluster: MHC class I; n=1; Gadus morhua|Rep: MHC class I - Gadus morhua (Atlantic cod)		3e-08	96% (28/29)	ES782517
sb_gmnllrta_0006p21.t7	1		unclassified				ES782975
sb_gmnllrta_0007a01.t7	1		unclassified				FL634320
sb_gmnllrta_0007a11.t7	1		unclassified				FL634325
sb_gmnllrta_0007a12.t7	1		unclassified				FL634326
sb_gmnllrta_0007a13.t7	1		unclassified				FL634327
sb_gmnllrta_0007a24.t7	1		unclassified				FL634333
sb_gmnllrta_0007b04.t7	1		unclassified				FL634335

sb_gmnllrta_0007b05.t7	1		unclassified				FL634336
sb_gmnllrta_0007b08.t7	1		Cluster: Hemoglobin subunit alpha-2; n=3; Gadidae|Rep: Hemoglobin subunit alpha-2 - Gadus morhua (Atlantic cod)	GO:0005344 GO:0005506 GO:0005833 GO:0006810 GO:0015671 GO:0019825 GO:0020037	8e-11	100% (31/31)	FL634337
sb_gmnllrta_0007b13.t7	1		unclassified				FL634341
sb_gmnllrta_0007b14.t7	1		Cluster: PREDICTED: similar to mtprd; n=1; Gallus gallus|Rep: PREDICTED: similar to mtprd - Gallus gallus		6e-08	72% (26/36)	FL634342
sb_gmnllrta_0007b16.t7	1		Unassigned protein				FL634344
sb_gmnllrta_0007b17.t7	1		unclassified				FL634345
sb_gmnllrta_0007b19.t7	1		Cluster: GatC-like protein (Protein 15E1.2).; n=1; Takifugu rubripes|Rep: GatC-like protein (Protein 15E1.2). - Takifugu rubripes		3e-16	70% (28/40)	FL634347
sb_gmnllrta_0007b23.t7	1		Cluster: Novel protein similar to human pre-mRNA cleavage complex II protein Pcf11; n=1; Danio rerio|Rep: Novel protein similar to human pre-mRNA cleavage complex II protein Pcf11 - Danio rerio (Zebrafish) (Brachydanio rerio)		2e-14	90% (39/43)	FL634348
sb_gmnllrta_0007c03.t7	1		unclassified				FL634350
sb_gmnllrta_0007c05.t7	1		unclassified				FL634352

sb_gmnllrta_0007c07.t7	1		unclassified				FL634354
sb_gmnllrta_0007c08.t7	1		Cluster: UPI000056791E related cluster; n=1; Danio rerio|Rep: UPI000056791E UniRef100 entry - Danio rerio		2e-08	23% (31/134)	FL634355
sb_gmnllrta_0007c13.t7	1		unclassified				FL634360
sb_gmnllrta_0007c16.t7	1		tm4sf3; transmembrane 4 superfamily member 3		4e-13	30% (41/135)	FL634363
sb_gmnllrta_0007c17.t7	1		unclassified				FL634364
sb_gmnllrta_0007c18.t7	1		unclassified				FL634365
sb_gmnllrta_0007c19.t7	1		unclassified				FL634366
sb_gmnllrta_0007c21.t7	1		unclassified				FL634368
sb_gmnllrta_0007c24.t7	1		Cluster: Thioredoxin-like 1; n=2; Danio rerio|Rep: Thioredoxin-like 1 - Danio rerio (Zebrafish) (Brachydanio rerio)		9e-62	86% (111/129)	FL634369
sb_gmnllrta_0007d02.t7	1		Cluster: Hemoglobin subunit alpha-1; n=3; Gadidae|Rep: Hemoglobin subunit alpha-1 - Gadus morhua (Atlantic cod)	GO:0005344 GO:0005506 GO:0005833 GO:0006810 GO:0015671 GO:0019825 GO:0020037	7e-09	96% (30/31)	FL634370
sb_gmnllrta_0007d08.t7	1		unclassified				FL634374

sb_gmnllrta_0007d09.t7	1		unclassified				FL634375
sb_gmnllrta_0007d10.t7	1		Cluster: Prostaglandine D synthase; n=2; Oncorhynchus|Rep: Prostaglandine D synthase - Oncorhynchus masou formosanus		3e-06	64% (24/37)	FL634376
sb_gmnllrta_0007d11.t7	1		unclassified				FL634377
sb_gmnllrta_0007d14.t7	1		unclassified				FL634379
sb_gmnllrta_0007d16.t7	1		unclassified				FL634380
sb_gmnllrta_0007d17.t7	1		Cluster: Fibrinogen, B beta polypeptide; n=2; Danio rerio|Rep: Fibrinogen, B beta polypeptide - Danio rerio (Zebrafish) (Brachydanio rerio)		2e-21	87% (43/49)	FL634381
sb_gmnllrta_0007d19.t7	1		unclassified				FL634382
sb_gmnllrta_0007d23.t7	1		unclassified				FL634383
sb_gmnllrta_0007d24.t7	1		unclassified				FL634384
sb_gmnllrta_0007e01.t7	1		unclassified				FL634385
sb_gmnllrta_0007e02.t7	1		unclassified				FL634386

sb_gmnllrta_0007e03.t7	1		unclassified				FL634387
sb_gmnllrta_0007e05.t7	1		unclassified				FL634388
sb_gmnllrta_0007e07.t7	1		unclassified				FL634389
sb_gmnllrta_0007e08.t7	1		unclassified				FL634390
sb_gmnllrta_0007e09.t7	1		Cluster: 40S ribosomal protein Sa; n=4; Euteleostomi|Rep: 40S ribosomal protein Sa - Pagrus major (Red sea bream) (Chrysophrys major)	GO:0003735 GO:0005622 GO:0005840 GO:0006412 GO:0015935	3e-06	95% (21/22)	FL634391
sb_gmnllrta_0007e13.t7	1		Cluster: Heat shock 70 kDa protein 1; n=7; Euteleostei|Rep: Heat shock 70 kDa protein 1 - Oryzias latipes (Medaka fish) (Japanese ricefish)	GO:0000166 GO:0005524	2e-70	76% (138/180)	FL634393
sb_gmnllrta_0007e19.t7	1		unclassified				FL634397
sb_gmnllrta_0007e22.t7	1		unclassified				FL634399
sb_gmnllrta_0007f02.t7	1		unclassified				FL634400
sb_gmnllrta_0007f06.t7	1		Cluster: Lipoprotein lipase precursor (EC 3.1.1.34) (LPL).; n=1; Danio rerio|Rep: Lipoprotein lipase precursor (EC 3.1.1.34) (LPL). - Danio rerio		2e-09	56% (18/32)	FL634403

sb_gmnllrta_0007f09.t7	1		unclassified				FL634404
sb_gmnllrta_0007f13.t7	1		Cluster: MGC85384 protein; n=3; Xenopus|Rep: MGC85384 protein - Xenopus laevis (African clawed frog)	GO:0003735 GO:0005622 GO:0005840	4e-08	57% (31/54)	FL634406
sb_gmnllrta_0007f16.t7	1		unclassified				FL634408
sb_gmnllrta_0007f20.t7	1		unclassified				FL634411
sb_gmnllrta_0007g04.t7	1		Cluster: Pyrophosphatase (Inorganic) 1; n=2; Danio rerio|Rep: Pyrophosphatase (Inorganic) 1 - Danio rerio (Zebrafish) (Brachydanio rerio)		3e-64	68% (66/97)	FL634415
sb_gmnllrta_0007g06.t7	1		unclassified				FL634416
sb_gmnllrta_0007g07.t7	1		Cluster: PREDICTED: glutaminyl-peptide cyclotransferase; n=1; Macaca mulatta|Rep: PREDICTED: glutaminyl-peptide cyclotransferase - Macaca mulatta		2e-09	81% (27/33)	FL634417
sb_gmnllrta_0007g08.t7	1		Cluster: Villin 2 (Ezrin) like; n=5; Danio rerio|Rep: Villin 2 (Ezrin) like - Danio rerio (Zebrafish) (Brachydanio rerio)		2e-06	78% (25/32)	FL634418
sb_gmnllrta_0007g11.t7	1		unclassified				FL634421
sb_gmnllrta_0007g12.t7	1		Cluster: Selenophosphate synthetase 2; n=3; Danio rerio|Rep: Selenophosphate synthetase 2 - Danio rerio (Zebrafish) (Brachydanio rerio)		2e-24	72% (55/76)	FL634422

sb_gmnllrta_0007g14.t7	1		unclassified				FL634424
sb_gmnllrta_0007g18.t7	1		unclassified				FL634428
sb_gmnllrta_0007g23.t7	1		LSU rRNA; Squalus acanthias		4e-33	96% (77/80)	FL634432
sb_gmnllrta_0007g24.t7	1		unclassified				FL634433
sb_gmnllrta_0007h01.t7	1		unclassified				FL634434
sb_gmnllrta_0007h02.t7	1		unclassified				FL634435
sb_gmnllrta_0007h12.t7	1		Cluster: Serine--pyruvate aminotransferase (EC 2.6.1.51) (SPT) (Alanine-- glyoxylate aminotransferase) (EC 2.6.1.44) (AGT).; n=1; Takifugu rubripes|Rep: Serine--pyruvate aminotransferase (EC 2.6.1.51) (SPT) (Alanine-- glyoxylate aminotransferase) (EC 2.6.1.44) (AGT). - Takifugu rubripes		4e-13	66% (24/36)	FL634443
sb_gmnllrta_0007h13.t7	1		unclassified				FL634444
sb_gmnllrta_0007h15.t7	1		Cluster: 40S ribosomal protein S18; n=38; Euteleostomi|Rep: 40S ribosomal protein S18 - Homo sapiens (Human)		2e-18	97% (47/48)	FL634445
sb_gmnllrta_0007h23.t7	1		Cluster: Zgc:152951; n=2; Danio rerio|Rep: Zgc:152951 - Danio rerio (Zebrafish) (Brachydanio rerio)		2e-05	65% (23/35)	FL634450

sb_gmnllrta_0007i04.t7	1		unclassified				FL634451
sb_gmnllrta_0007i05.t7	1		unclassified				FL634452
sb_gmnllrta_0007i07.t7	1		unclassified				FL634453
sb_gmnllrta_0007i10.t7	1		unclassified				FL634455
sb_gmnllrta_0007i19.t7	1		unclassified				FL634460
sb_gmnllrta_0007i20.t7	1		unclassified				FL634461
sb_gmnllrta_0007i21.t7	1		unclassified				FL634462
sb_gmnllrta_0007j01.t7	1		Cluster: Villin 2 (Ezrin) like; n=5; Danio rerio|Rep: Villin 2 (Ezrin) like - Danio rerio (Zebrafish) (Brachydanio rerio)		3e-05	75% (24/32)	FL634465
sb_gmnllrta_0007j03.t7	1		Cluster: Prothrombin; n=1; Takifugu rubripes|Rep: Prothrombin - Fugu rubripes (Japanese pufferfish) (Takifugu rubripes)	GO:0003809 GO:0003824 GO:0004252 GO:0005509 GO:0005576 GO:0006508 GO:0007596 GO:0008233	5e-18	87% (41/47)	FL634466
sb_gmnllrta_0007j11.t7	1		Cluster: Si:rp71-39b20.7 protein; n=3; Danio rerio|Rep: Si:rp71-39b20.7 protein - Danio rerio (Zebrafish) (Brachydanio rerio)		5e-17	76% (39/51)	FL634471

sb_gmnllrta_0007j14.t7	1		Cluster: Zgc:123297; n=2; Danio rerio|Rep: Zgc:123297 - Danio rerio (Zebrafish) (Brachydanio rerio)		2e-06	40% (31/76)	FL634474
sb_gmnllrta_0007j16.t7	1		unclassified				FL634476
sb_gmnllrta_0007j19.t7	1		unclassified				FL634478
sb_gmnllrta_0007j20.t7	1		unclassified				FL634479
sb_gmnllrta_0007j21.t7	1		unclassified				FL634480
sb_gmnllrta_0007j23.t7	1		unclassified				FL634481
sb_gmnllrta_0007j24.t7	1		Cluster: Lysosomal-associated protein transmembrane 4 alpha; n=4; Clupeocephala|Rep: Lysosomal-associated protein transmembrane 4 alpha - Danio rerio (Zebrafish) (Brachydanio rerio)		3e-52	94% (99/105)	FL634482
sb_gmnllrta_0007k01.t7	1		unclassified				FL634483
sb_gmnllrta_0007k02.t7	1		Cluster: Thioredoxin interacting protein; n=1; Danio rerio|Rep: Thioredoxin interacting protein - Danio rerio (Zebrafish) (Brachydanio rerio)		5e-08	73% (25/34)	FL634484
sb_gmnllrta_0007k04.t7	1		unclassified				FL634486
sb_gmnllrta_0007k09.t7	1		unclassified				FL634489

sb_gmnllrta_0007k11.t7	1		unclassified				FL634491
sb_gmnllrta_0007k13.t7	1		unclassified				FL634493
sb_gmnllrta_0007k17.t7	1		Cluster: Ribosomal protein S13; n=7; Euteleostomi|Rep: Ribosomal protein S13 - Siniperca chuatsi (Chinese perch)	GO:0003735 GO:0005622 GO:0005840	3e-37	98% (78/79)	FL634496
sb_gmnllrta_0007k23.t7	1		unclassified				FL634499
sb_gmnllrta_0007l04.t7	1		unclassified				FL634502
sb_gmnllrta_0007l07.t7	1		unclassified				FL634503
sb_gmnllrta_0007l09.t7	1		Cluster: PREDICTED: similar to cathepsin L; n=1; Ornithorhynchus anatinus|Rep: PREDICTED: similar to cathepsin L - Ornithorhynchus anatinus		1e-09	69% (27/39)	FL634505
sb_gmnllrta_0007l11.t7	1		unclassified				FL634506
sb_gmnllrta_0007l15.t7	1		Cluster: Zgc:153988; n=1; Danio rerio|Rep: Zgc:153988 - Danio rerio (Zebrafish) (Brachydanio rerio)		4e-28	54% (59/109)	FL634509
sb_gmnllrta_0007l16.t7	1		unclassified				FL634510
sb_gmnllrta_0007l20.t7	1		Cluster: PREDICTED: similar to ATP synthase lipid binding protein p3; n=1; Ornithorhynchus anatinus|Rep: PREDICTED: similar to ATP synthase lipid binding protein p3 - Ornithorhynchus		3e-06	100% (26/26)	FL634511

			anatinus				
sb_gmnllrta_0007l21.t7	1		unclassified				FL634512
sb_gmnllrta_0007l22.t7	1		unclassified				FL634513
sb_gmnllrta_0007l24.t7	1		unclassified				FL634514
sb_gmnllrta_0007m06.t7	1		Cluster: Proteasome subunit beta type; n=3; Danio rerio|Rep: Proteasome subunit beta type - Danio rerio (Zebrafish) (Brachydanio rerio)		1e-35	97% (73/75)	FL634518
sb_gmnllrta_0007m10.t7	1		unclassified				FL634522
sb_gmnllrta_0007m11.t7	1		Cluster: Heat shock protein 90; n=13; Euteleostomi|Rep: Heat shock protein 90 - Scophthalmus maximus (Turbot)	GO:0005524 GO:0006457 GO:0006950	4e-10	96% (32/33)	FL634523
sb_gmnllrta_0007m13.t7	1		unclassified				FL634524
sb_gmnllrta_0007m15.t7	1		Unassigned protein				FL634525
sb_gmnllrta_0007m19.t7	1		unclassified				FL634529
sb_gmnllrta_0007m20.t7	1		Cluster: Interleukin-8; n=1; Melanogrammus aeglefinus|Rep: Interleukin-8 - Melanogrammus aeglefinus (Haddock)	GO:0005125 GO:0005576 GO:0005615 GO:0006955	9e-36	82% (63/76)	FL634530

sb_gmnllrta_0007m22.t7	1		unclassified				FL634532
sb_gmnllrta_0007m23.t7	1		unclassified				FL634533
sb_gmnllrta_0007n02.t7	1		unclassified				FL634536
sb_gmnllrta_0007n05.t7	1		unclassified				FL634537
sb_gmnllrta_0007n07.t7	1		Cluster: Zgc:56585 protein; n=3; Danio rerio|Rep: Zgc:56585 protein - Danio rerio (Zebrafish) (Brachydanio rerio)		2e-44	61% (86/139)	FL634539
sb_gmnllrta_0007n14.t7	1		unclassified				FL634543
sb_gmnllrta_0007n16.t7	1		unclassified				FL634545
sb_gmnllrta_0007n17.t7	1		unclassified				FL634546
sb_gmnllrta_0007n18.t7	1		unclassified				FL634547
sb_gmnllrta_0007n19.t7	1		Cluster: Zgc:86599; n=3; Clupeocephala|Rep: Zgc:86599 - Danio rerio (Zebrafish) (Brachydanio rerio)		2e-10	86% (26/30)	FL634548
sb_gmnllrta_0007n20.t7	1		Cluster: Homolog of Homo sapiens "Receptor-type tyrosine-protein phosphatase beta precursor; n=1; Takifugu rubripes|Rep: Homolog of Homo sapiens "Receptor-type tyrosine-protein phosphatase beta precursor - Takifugu		3e-08	65% (25/38)	FL634549

			rubripes				
sb_gmnllrta_0007n21.t7	1		unclassified				FL634550
sb_gmnllrta_0007o07.t7	1		Itm1; similar to intergral membrane protein 1; K07151		9e-53	98% (98/99)	FL634553
sb_gmnllrta_0007o10.t7	1		unclassified				FL634556
sb_gmnllrta_0007o11.t7	1		unclassified				FL634557
sb_gmnllrta_0007o16.t7	1		unclassified				FL634561
sb_gmnllrta_0007o17.t7	1		unclassified				FL634562
sb_gmnllrta_0007o20.t7	1		Cluster: PASG; n=6; Danio rerio|Rep: PASG - Danio rerio (Zebrafish) (Brachydanio rerio)		1e-09	100% (31/31)	FL634565
sb_gmnllrta_0007p04.t7	1		Cluster: UDP-glucose 4-epimerase (EC 5.1.3.2) (Galactowaldenase) (UDP-galactose 4-epimerase).; n=1; Takifugu rubripes|Rep: UDP-glucose 4-epimerase (EC 5.1.3.2) (Galactowaldenase) (UDP-galactose 4-epimerase). - Takifugu rubripes		3e-16	88% (37/42)	FL634569
sb_gmnllrta_0007p10.t7	1		Cluster: PREDICTED: similar to ribosomal protein S5; n=1; Ornithorhynchus anatinus|Rep: PREDICTED: similar to ribosomal protein S5 - Ornithorhynchus anatinus		2e-13	97% (35/36)	FL634571
sb_gmnllrta_0007p13.t7	1		Cluster: TNFAIP3 interacting protein 1; n=3; Danio rerio|Rep: TNFAIP3 interacting protein 1 - Danio rerio		5e-35	86% (74/86)	FL634573

			(Zebrafish) (Brachydanio rerio)				

[1]Annotations presented in the supplemental table were generated with AutoFACT [18], while annotations presented in the manuscript are recent BLASTx hits that reflect a more updated state of the NCBI's nr protein database
