# Supplementary material for: Heat-shock responsive genes identified and validated in Atlantic cod (Gadus morhua) liver, head kidney and skeletal muscle using genomic techniques
Source: BMC Genomics. 2010 Jan 28;11:72. doi: 10.1186/1471-2164-11-72 (PMC2830189; doi:10.1186/1471-2164-11-72)
Supplement: Additional file 3 — Supplemental Table S3, summary of GO annotation. Contains 4 tables (S3 A-D) with summaries of percentages of total ESTs with GO (biological process) terms for each of 4 SSH libraries. [file 1471-2164-11-72-S3.PDF]

**Supplemental Table S3A. Head Kidney Forward SSH Library: biological process (BP) Gene Ontology (GO)**

| Term | Description | # of assembled ESTs | Percentage of total ESTs with GO (BP) |
|---|---|---|---|
| GO:0006412 | protein biosynthesis | 21 | 17.65% |
| GO:0006810 | transport | 11 | 9.24% |
| GO:0006955 | immune response | 10 | 8.40% |
| GO:0006457 | protein folding | 7 | 5.88% |
| GO:0015671 | oxygen transport | 7 | 5.88% |
| GO:0019882 | antigen presentation | 6 | 5.04% |
| GO:0006508 | proteolysis | 5 | 4.20% |
| GO:0006118 | electron transport | 4 | 3.36% |
| GO:0006511 | ubiquitin-dependent protein catabolism | 4 | 3.36% |
| GO:0006950 | response to stress | 3 | 2.52% |
| GO:0008152 | metabolism | 3 | 2.52% |
| GO:0006099 | tricarboxylic acid cycle | 2 | 1.68% |
| GO:0006355 | regulation of transcription, DNA-dependent | 2 | 1.68% |
| GO:0006464 | protein modification | 2 | 1.68% |
| GO:0006813 | potassium ion transport | 2 | 1.68% |
| GO:0006814 | sodium ion transport | 2 | 1.68% |
| GO:0006826 | iron ion transport | 2 | 1.68% |
| GO:0006879 | iron ion homeostasis | 2 | 1.68% |
| GO:0044267 | cellular protein metabolism | 2 | 1.68% |
| GO:0000074 | regulation of progression through cell cycle | 1 | 0.84% |
| GO:0002474 | antigen processing and presentation of peptide antigen via MHC class I | 1 | 0.84% |
| GO:0005975 | carbohydrate metabolism | 1 | 0.84% |
| GO:0006098 | pentose-phosphate shunt | 1 | 0.84% |
| GO:0006100 | tricarboxylic acid cycle intermediate metabolism | 1 | 0.84% |
| GO:0006108 | malate metabolism | 1 | 0.84% |
| GO:0006352 | transcription initiation | 1 | 0.84% |
| GO:0006413 | translational initiation | 1 | 0.84% |
| GO:0006414 | translational elongation | 1 | 0.84% |
| GO:0006417 | regulation of protein biosynthesis | 1 | 0.84% |
| GO:0006418 | tRNA aminoacylation for protein translation | 1 | 0.84% |
| GO:0006422 | aspartyl-tRNA aminoacylation | 1 | 0.84% |
| GO:0006730 | one-carbon compound metabolism | 1 | 0.84% |
| GO:0006801 | superoxide metabolism | 1 | 0.84% |
| GO:0006811 | ion transport | 1 | 0.84% |
| GO:0007165 | signal transduction | 1 | 0.84% |
| GO:0007186 | G-protein coupled receptor protein signaling pathway | 1 | 0.84% |
| GO:0009253 | peptidoglycan catabolism | 1 | 0.84% |
| GO:0016032 | viral life cycle | 1 | 0.84% |
| GO:0018149 | peptide cross-linking | 1 | 0.84% |
| GO:0019082 | viral protein processing | 1 | 0.84% |
| GO:0050819 | negative regulation of coagulation | 1 | 0.84% |
| | Total | 119 | 100.00% |

**Supplemental Table S3B. Liver Forward SSH Library: biological process (BP) Gene Ontology (GO)**

| Term | Description | # of assembled ESTs | Percentage of total ESTs with GO (BP) |
|---|---|---|---|
| GO:0006810 | transport | 33 | 18.97% |
| GO:0015671 | oxygen transport | 22 | 12.64% |
| GO:0006412 | protein biosynthesis | 16 | 9.20% |
| GO:0006118 | electron transport | 8 | 4.60% |
| GO:0006508 | proteolysis | 6 | 3.45% |
| GO:0006826 | iron ion transport | 6 | 3.45% |
| GO:0006879 | iron ion homeostasis | 6 | 3.45% |
| GO:0008152 | metabolism | 6 | 3.45% |
| GO:0006811 | ion transport | 5 | 2.87% |
| GO:0006464 | protein modification | 4 | 2.30% |
| GO:0006955 | immune response | 4 | 2.30% |
| GO:0007017 | microtubule-based process | 4 | 2.30% |
| GO:0007018 | microtubule-based movement | 4 | 2.30% |
| GO:0051258 | protein polymerization | 4 | 2.30% |
| GO:0006457 | protein folding | 3 | 1.72% |
| GO:0006096 | glycolysis | 2 | 1.15% |
| GO:0006954 | inflammatory response | 2 | 1.15% |
| GO:0007165 | signal transduction | 2 | 1.15% |
| GO:0007596 | blood coagulation | 2 | 1.15% |
| GO:0019882 | antigen presentation | 2 | 1.15% |
| GO:0006094 | gluconeogenesis | 1 | 0.57% |
| GO:0006099 | tricarboxylic acid cycle | 1 | 0.57% |
| GO:0006355 | regulation of transcription, DNA-dependent | 1 | 0.57% |
| GO:0006414 | translational elongation | 1 | 0.57% |
| GO:0006512 | ubiquitin cycle | 1 | 0.57% |
| GO:0006605 | protein targeting | 1 | 0.57% |
| GO:0006626 | protein targeting to mitochondrion | 1 | 0.57% |
| GO:0006801 | superoxide metabolism | 1 | 0.57% |
| GO:0006812 | cation transport | 1 | 0.57% |
| GO:0006869 | lipid transport | 1 | 0.57% |
| GO:0006915 | apoptosis | 1 | 0.57% |
| GO:0006950 | response to stress | 1 | 0.57% |
| GO:0006956 | complement activation | 1 | 0.57% |
| GO:0006957 | complement activation, alternative pathway | 1 | 0.57% |
| GO:0006958 | complement activation, classical pathway | 1 | 0.57% |
| GO:0006979 | response to oxidative stress | 1 | 0.57% |
| GO:0007049 | cell cycle | 1 | 0.57% |
| GO:0007154 | cell communication | 1 | 0.57% |
| GO:0007268 | synaptic transmission | 1 | 0.57% |
| GO:0008643 | carbohydrate transport | 1 | 0.57% |
| GO:0009058 | biosynthesis | 1 | 0.57% |
| GO:0009405 | pathogenesis | 1 | 0.57% |
| GO:0015031 | protein transport | 1 | 0.57% |
| GO:0015672 | monovalent inorganic cation transport | 1 | 0.57% |
| GO:0016032 | viral life cycle | 1 | 0.57% |

| | | | |
|---|---|---|---|
| GO:0017187 | peptidyl-glutamic acid carboxylation | 1 | 0.57% |
| GO:0019069 | viral capsid assembly | 1 | 0.57% |
| GO:0030168 | platelet activation | 1 | 0.57% |
| GO:0042157 | lipoprotein metabolism | 1 | 0.57% |
| GO:0042309 | homoiothermy | 1 | 0.57% |
| GO:0045039 | protein import into mitochondrial inner membrane | 1 | 0.57% |
| GO:0045087 | innate immune response | 1 | 0.57% |
| GO:0045454 | cell redox homeostasis | 1 | 0.57% |
| | Total | 174 | 100.00% |

**Supplemental Table S3C. Skeletal Muscle Forward SSH Library: biological process (BP) Gene Ontology (GO)**

| Term | Description | # of assembled ESTs | Percentage of total ESTs with GO (BP) |
|---|---|---|---|
| GO:0006412 | protein biosynthesis | 13 | 10.16% |
| GO:0006810 | transport | 12 | 9.38% |
| GO:0008152 | metabolism | 12 | 9.38% |
| GO:0006811 | ion transport | 7 | 5.47% |
| GO:0006812 | cation transport | 6 | 4.69% |
| GO:0006816 | calcium ion transport | 6 | 4.69% |
| GO:0031448 | positive regulation of striated fast muscle contraction | 5 | 3.91% |
| GO:0006096 | glycolysis | 4 | 3.13% |
| GO:0006457 | protein folding | 4 | 3.13% |
| GO:0007165 | signal transduction | 3 | 2.34% |
| GO:0009168 | purine ribonucleoside monophosphate biosynthesis | 3 | 2.34% |
| GO:0005975 | carbohydrate metabolism | 2 | 1.56% |
| GO:0006754 | ATP biosynthesis | 2 | 1.56% |
| GO:0006826 | iron ion transport | 2 | 1.56% |
| GO:0006879 | iron ion homeostasis | 2 | 1.56% |
| GO:0006950 | response to stress | 2 | 1.56% |
| GO:0006955 | immune response | 2 | 1.56% |
| GO:0015986 | ATP synthesis coupled proton transport | 2 | 1.56% |
| GO:0015992 | proton transport | 2 | 1.56% |
| GO:0042254 | ribosome biogenesis and assembly | 2 | 1.56% |
| GO:0006098 | pentose-phosphate shunt | 1 | 0.78% |
| GO:0006099 | tricarboxylic acid cycle | 1 | 0.78% |
| GO:0006100 | tricarboxylic acid cycle intermediate metabolism | 1 | 0.78% |
| GO:0006108 | malate metabolism | 1 | 0.78% |
| GO:0006118 | electron transport | 1 | 0.78% |
| GO:0006122 | mitochondrial electron transport, ubiquinol to cytochrome c | 1 | 0.78% |
| GO:0006183 | GTP biosynthesis | 1 | 0.78% |
| GO:0006228 | UTP biosynthesis | 1 | 0.78% |
| GO:0006241 | CTP biosynthesis | 1 | 0.78% |
| GO:0006260 | DNA replication | 1 | 0.78% |
| GO:0006275 | regulation of DNA replication | 1 | 0.78% |
| GO:0006355 | regulation of transcription, DNA-dependent | 1 | 0.78% |
| GO:0006414 | translational elongation | 1 | 0.78% |
| GO:0006418 | tRNA aminoacylation for protein translation | 1 | 0.78% |
| GO:0006421 | asparaginyl-tRNA aminoacylation | 1 | 0.78% |
| GO:0006422 | aspartyl-tRNA aminoacylation | 1 | 0.78% |
| GO:0006464 | protein modification | 1 | 0.78% |
| GO:0006471 | protein amino acid ADP-ribosylation | 1 | 0.78% |
| GO:0006511 | ubiquitin-dependent protein catabolism | 1 | 0.78% |
| GO:0006573 | valine metabolism | 1 | 0.78% |
| GO:0006626 | protein targeting to mitochondrion | 1 | 0.78% |
| GO:0006730 | one-carbon compound metabolism | 1 | 0.78% |
| GO:0006839 | mitochondrial transport | 1 | 0.78% |
| GO:0006886 | intracellular protein transport | 1 | 0.78% |
| GO:0006913 | nucleocytoplasmic transport | 1 | 0.78% |

| | | | |
|---|---|---|---|
| GO:0006915 | apoptosis | 1 | 0.78% |
| GO:0007017 | microtubule-based process | 1 | 0.78% |
| GO:0007154 | cell communication | 1 | 0.78% |
| GO:0007186 | G-protein coupled receptor protein signaling pathway | 1 | 0.78% |
| GO:0007264 | small GTPase mediated signal transduction | 1 | 0.78% |
| GO:0009117 | nucleotide metabolism | 1 | 0.78% |
| GO:0009231 | riboflavin biosynthesis | 1 | 0.78% |
| GO:0015031 | protein transport | 1 | 0.78% |
| GO:0042981 | regulation of apoptosis | 1 | 0.78% |
| GO:0045039 | protein import into mitochondrial inner membrane | 1 | 0.78% |
| | Total | 128 | 100.00% |

**Supplemental Table S3D. Liver Reverse SSH Library: biological process (BP) Gene Ontology (GO)**

| Term | Description | # of assembled ESTs | Percentage of total ESTs with GO (BP) |
|---|---|---|---|
| GO:0006810 | transport | 27 | 19.29% |
| GO:0015671 | oxygen transport | 24 | 17.14% |
| GO:0006412 | protein biosynthesis | 16 | 11.43% |
| GO:0006457 | protein folding | 7 | 5.00% |
| GO:0006955 | immune response | 7 | 5.00% |
| GO:0006118 | electron transport | 6 | 4.29% |
| GO:0006508 | proteolysis | 5 | 3.57% |
| GO:0006826 | iron ion transport | 5 | 3.57% |
| GO:0006879 | iron ion homeostasis | 5 | 3.57% |
| GO:0008152 | metabolism | 5 | 3.57% |
| GO:0019882 | antigen presentation | 5 | 3.57% |
| GO:0006464 | protein modification | 3 | 2.14% |
| GO:0007596 | blood coagulation | 3 | 2.14% |
| GO:0006355 | regulation of transcription, DNA-dependent | 2 | 1.43% |
| GO:0006511 | ubiquitin-dependent protein catabolism | 2 | 1.43% |
| GO:0006811 | ion transport | 2 | 1.43% |
| GO:0007155 | cell adhesion | 2 | 1.43% |
| GO:0000074 | regulation of progression through cell cycle | 1 | 0.71% |
| GO:0001516 | prostaglandin biosynthesis | 1 | 0.71% |
| GO:0005975 | carbohydrate metabolism | 1 | 0.71% |
| GO:0006096 | glycolysis | 1 | 0.71% |
| GO:0006098 | pentose-phosphate shunt | 1 | 0.71% |
| GO:0006633 | fatty acid biosynthesis | 1 | 0.71% |
| GO:0006950 | response to stress | 1 | 0.71% |
| GO:0006954 | inflammatory response | 1 | 0.71% |
| GO:0006979 | response to oxidative stress | 1 | 0.71% |
| GO:0007017 | microtubule-based process | 1 | 0.71% |
| GO:0007018 | microtubule-based movement | 1 | 0.71% |
| GO:0008610 | lipid biosynthesis | 1 | 0.71% |
| GO:0043066 | negative regulation of apoptosis | 1 | 0.71% |
| GO:0045087 | innate immune response | 1 | 0.71% |
| | Total | 140 | |
